# An Open-Label Trial of 12-Week Simeprevir plus Peginterferon/Ribavirin (PR) in Treatment-Naïve Patients with Hepatitis C Virus (HCV) Genotype 1 (GT1)

**DOI:** 10.1371/journal.pone.0158526

**Published:** 2016-07-18

**Authors:** Tarik Asselah, Christophe Moreno, Christoph Sarrazin, Michael Gschwantler, Graham R. Foster, Antonio Craxí, Peter Buggisch, Robert Ryan, Oliver Lenz, Jane Scott, Gino Van Dooren, Isabelle Lonjon-Domanec, Michael Schlag, Maria Buti

**Affiliations:** 1 Service d'Hépatologie, Beaujon Hospital, INSERM UMR 1149, Université Paris Diderot, Paris, France; 2 CUB Hôpital Erasme, Université Libre de Bruxelles, Brussels, Belgium; 3 Johann Wolfgang Goethe University Hospital, Frankfurt am Main, Germany; 4 Wilhelminenspital, Vienna, Austria; 5 Queen Mary Hospital, University of London, Barts Health, London, United Kingdom; 6 University of Palermo, Palermo, Italy; 7 Institute for Interdisciplinary Medicine, Hamburg, Germany; 8 Janssen Research & Development, Titusville, New Jersey, United States of America; 9 Janssen Infectious Diseases BVBA, Beerse, Belgium; 10 Janssen Global Services, High Wycombe, United Kingdom; 11 Janssen Pharmaceuticals, Paris, France; 12 Janssen-Cilag, Vienna, Austria; 13 Hospital Valle Hebron and Ciberehd del Institut Carlos III, Barcelona, Spain; University of New South Wales, AUSTRALIA

## Abstract

**Background:**

Shortening duration of peginterferon-based HCV treatment reduces associated burden for patients. Primary objectives of this study were to assess the efficacy against the minimally acceptable response rate 12 weeks post-treatment (SVR12) and safety of simeprevir plus PR in treatment-naïve HCV GT1 patients treated for 12 weeks. Additional objectives included the investigation of potential associations of rapid viral response and baseline factors with SVR12.

**Methods:**

In this Phase III, open-label study in treatment-naïve HCV GT1 patients with F0–F2 fibrosis, patients with HCV-RNA <25 IU/mL (detectable/undetectable) at Week 2, and undetectable HCV-RNA at Weeks 4 and 8, stopped all treatment at Week 12. All other patients continued PR for a further 12 weeks. Baseline factors significantly associated with SVR12 were identified through logistic regression.

**Results:**

Of 163 patients who participated in the study, 123 (75%) qualified for 12-week treatment; of these, 81 (66%) achieved SVR12. Baseline factors positively associated with SVR12 rates in patients receiving the 12-week regimen were: *IL28B* CC genotype: (94% SVR12); HCV RNA ≤800,000 IU/mL (82%); F0–F1 fibrosis (74%). Among all 163 patients, 94% experienced ≥1 adverse event (AE), 4% a serious AE, and 2.5% discontinued due to an AE. Reduced impairment in patient-reported outcomes was observed in the 12-week *vs* >12-week regimen.

**Conclusions:**

Overall SVR12 rate (66%) was below the target of 80%, indicating that shortening of treatment with simeprevir plus PR to 12 weeks based on very early response is not effective. However, baseline factors associated with higher SVR12 rates were identified. Therefore, while Week 2 response alone is insufficient to predict efficacy, GT1 patients with favourable baseline factors may benefit from a shortened simeprevir plus PR regimen.

**Trial Registration:**

ClinicalTrials.gov NCT01846832

## Introduction

The desired outcome of treatment for hepatitis C virus (HCV) infection is sustained virologic response (SVR), typically measured 12 weeks after end of treatment (SVR12). This endpoint strongly correlates with permanent clearance of the virus [1,2].

Treatment with peginterferon (peg-IFN; P)/ribavirin (RBV; R) in combination with a direct-acting antiviral (DAA) is associated with markedly improved SVR rates and shortened treatment durations compared with PR alone [3,4]. Simeprevir is a one-pill, once-daily HCV NS3/4A second-generation protease inhibitor (PI) approved in Europe, the USA, Canada, Japan and Russia for the treatment of chronic HCV genotype 1 (GT1) and GT4 infection [5,6].

The QUEST-1 and -2 Phase III trials investigated efficacy and safety of simeprevir in combination with PR in treatment-naïve HCV GT1 patients, and demonstrated SVR12 rates of 80–81% in patients receiving 24 or 48 weeks of treatment [7,8]. Eligibility for 24-week treatment in these trials was determined by assessment against response-guided therapy (RGT) criteria, whereby HCV RNA was required to be <25 IU/mL detectable or undetectable at Week 4, and undetectable at Week 12. Of the 88% (459/521) patients receiving simeprevir treatment who met these criteria, 88% (405/459) achieved SVR12. Furthermore, in a similar study conducted in Japanese GT1 (98% GT1b) patients, the CONCERTO-1 Phase III trial, treatment-naïve patients demonstrated an SVR12 rate of 89% with a 12-week regimen of simeprevir (100 mg once daily) plus PR [9].

Based on these results, the European and US label for simeprevir recommends a fixed 24-week simeprevir plus PR triple therapy regimen comprising 12 weeks of treatment with simeprevir plus PR followed by 12 weeks of PR, for GT1 and GT4 treatment-naïve patients and prior relapsers (including cirrhotic patients).

Shortening of peg-IFN-based treatment regimens is of interest for a number of reasons, including: reduced incidence of treatment-associated adverse events (AEs); better treatment adherence; reductions in costs; and reduced duration of impact on patient quality of life [10,11]. A 12-week simeprevir plus PR treatment regimen may be sufficiently effective in some patients. Previously, an SVR12 rate of 87% was demonstrated with a 12-week interferon (IFN)-based telaprevir regimen in non-cirrhotic GT1 patients with favorable baseline characteristics demonstrating a rapid virologic response (RVR) at Week 4 [12]. More recently, 90% SVR12 (95% CI: 87–93%) has been achieved in treatment-naïve patients with a 12-week regimen of sofosbuvir plus PR in patients with GT1 or GT4–6 [13].

In our Phase III study of treatment-naïve patients with chronic GT1 infection and F0-F2 fibrosis, very rapid (Week 2) virologic response (vRVR; HCV RNA <25 IU/mL [detectable/undetectable]) was used to determine eligibility for a 12-week simeprevir plus PR regimen. Objectives were to assess efficacy and safety in the 12-week and >12-week groups, determine whether Week 2 response was predictive of SVR12, and identify baseline factors associated with SVR with the 12-week regimen.

## Methods

### Patients and study design

This multicenter, open-label, single-arm, Phase III clinical trial (NCT01846832), which took place between 3 September 2013, when the first patient was screened, and 31 August 2015 (date of last patient contact) evaluated the efficacy and safety of a 12-week regimen of simeprevir plus PR in treatment-naïve patients with HCV GT1 or GT4. Results in patients with GT4 have been reported separately [14].

The study was approved by each center’s institutional review board and conducted in accordance with the Declaration of Helsinki and Good Clinical Practice guidelines. The review boards are listed in **S1 Text**. Written informed consent was obtained from all patients at screening prior to the performance of any study-specific assessments or procedures. Investigators at sites in seven European countries invited patients to participate in the study. Each patient received an identification code to permit easy identification of each subject during and after the study. The code list was treated as confidential and filed by the investigator in the study file. The study protocol and supporting TREND checklist are available as supporting information (**S1 Protocol**; **S1 TREND Checklist**).

Eligible patients were aged 18 to 70 years with confirmed chronic GT1 infection at least 6 months prior to screening, plasma HCV RNA >10,000 IU/mL and had received no prior treatment for HCV. Patients’ liver fibrosis score was F0–F2 (METAVIR or equivalent) as confirmed by liver biopsy or non-invasive assessment [15]. Patients with F3–F4 fibrosis were excluded, as were patients co-infected with HBV and/or HIV. Further details of the inclusion/exclusion criteria are provided in **S1 Appendix**. Patients were stratified according to HCV genotype/subtype and *IL28B* genotype.

All patients received simeprevir (150 mg once daily, orally) for 12 weeks in combination with Peg-IFNα2a (180 μg/week, subcutaneously) and RBV (1000 mg/day <75 kg body weight, or 1200 mg/day ≥75 kg, orally). Treatments were stored and administered by participants in accordance with instructions provided by study site personnel. The investigator or designated study site personnel maintained a log of all study treatments dispensed and returned for compliance assessment purposes.

Treatment concluded at Week 12 if HCV RNA was <25 IU/mL (undetectable or detectable) at Week 2 (vRVR), and was <25 IU/mL undetectable at Week 4 and Week 8 (12-week group). Patients not meeting these criteria continued PR for a further 12 weeks (>12-week group). Patients in France could receive up to 36 further weeks of PR, but only one patient did so. For analysis purposes, patients discontinuing treatment before Week 8 were considered as not having met the eligibility criteria for the shortened treatment regimen, as Week 8 viral load was part of these criteria. As such, these patients were assigned to the >12-week group.

HCV RNA was measured by Roche Cobas TaqMan v2.0 assay (lower limit of quantification [LLOQ]: 25 IU/mL, limit of detection: 15 IU/mL).

Virologic stopping rules, requiring the discontinuation of all treatment, were: HCV RNA ≥25 IU/mL at Week 4; HCV RNA ≥25 IU/mL or <25 IU/mL detectable at Week 12; or viral breakthrough (confirmed increase of >1 log_10_ IU/mL in HCV RNA concentration from the lowest level, or confirmed HCV RNA level of >100 IU/mL following achievement of <25 IU/mL [detectable or undetectable] while on study treatment).

### Objectives

The primary objective of the study was to assess efficacy, as measured by SVR12 (HCV RNA <25 IU/mL undetectable 12 weeks after planned end of treatment), and safety of the 12-week regimen in patients with GT1. The study hypothesis was that the proportion of patients achieving SVR12 in the 12-week group would be superior to the minimally acceptable response rate of 80%.

Secondary and other objectives included: proportion of patients achieving vRVR at Week 2, and RVR at Week 4; relationship between Week 2 response, Week 4 response and SVR12; SVR12 among patients receiving >12-week treatment; SVR24 in patients receiving 12- or >12-week treatment; and patient-reported outcomes. Exploratory univariate and multivariate analyses (12-week group only) investigated factors influencing SVR12 and viral relapse.

### Evaluations

Blood samples for the determination of HCV RNA levels and for viral sequencing were collected at screening, at baseline and at each study visit throughout treatment (Weeks 4, 8, 12, 16, 20 and 24) and follow-up (Weeks 1, 2, 4, 8, 12, 16, 20, 24, 28, 36, and 48) as pre-specified in the time and events schedule. The HCV NS3/4A region was sequenced at baseline to test for the Q80K polymorphism, and at treatment failure to determine emergence of resistant viral variants. Additionally, *IL28B* genotype was determined at screening.

AEs reported by the patient or appropriate caregiver were coded using the Medical Dictionary for Regulatory Activities and recorded throughout the study, and blood samples were used for biochemical and hematological analyses. AEs of special interest were reported as described in previous publications [16]. Electrocardiograms, vital sign assessments and physical examinations were also performed throughout the study period.

Patient-reported outcomes questionnaires were completed at baseline and at pre-specified timepoints throughout treatment (Weeks 4, 8, 12, 16, 20 and 24) and follow-up (Weeks 4, 8, 12, 16, 20,24, 28, 36 and 48).

### Statistical methods

Efficacy analyses were conducted in the intent-to-treat population (all patients receiving ≥1 dose of study medication). Treatment of 150 eligible patients, of whom 69% (104/150) were expected to be eligible for the 12-week group, was based on an assumed estimated *IL28B* genotype breakdown of 30% CC, 55% CT and 15% TT, and a 50% G1a/G1b split. The sample size was determined based on the minimally acceptable response rate (80%) and target response rate of 90%. With α = 0.05 and a sample size of 104, the power to show the minimally acceptable response rate was estimated by Z-test as 91%.

All reported AEs with onset during the treatment phase were included in the safety analysis.

*Post-hoc* univariate and step-wise multivariate logistic regression analyses were performed to determine prognostic factors associated with SVR12 or viral relapse among patients in the 12-week group (all patients; patients with *IL28B* non-CC genotype). The significance of these prognostic factors was assessed using Wald’s test. Statistical analysis was conducted using SAS statistical analysis software (SAS Institute Inc). Classification and regression tree analysis of factors associated with SVR12 in patients receiving 12 weeks of treatment was performed using SAS^®^ JMP. Only those factors that were included in the final multivariate model were included in the regression tree analysis: *IL28B* genotype, HCV RNA at baseline, and METAVIR fibrosis score.

For measures of patient-reported outcomes, descriptive statistics are reported according to treatment group and achievement or otherwise of SVR12.

## Results

### Patient disposition

**Fig 1:**
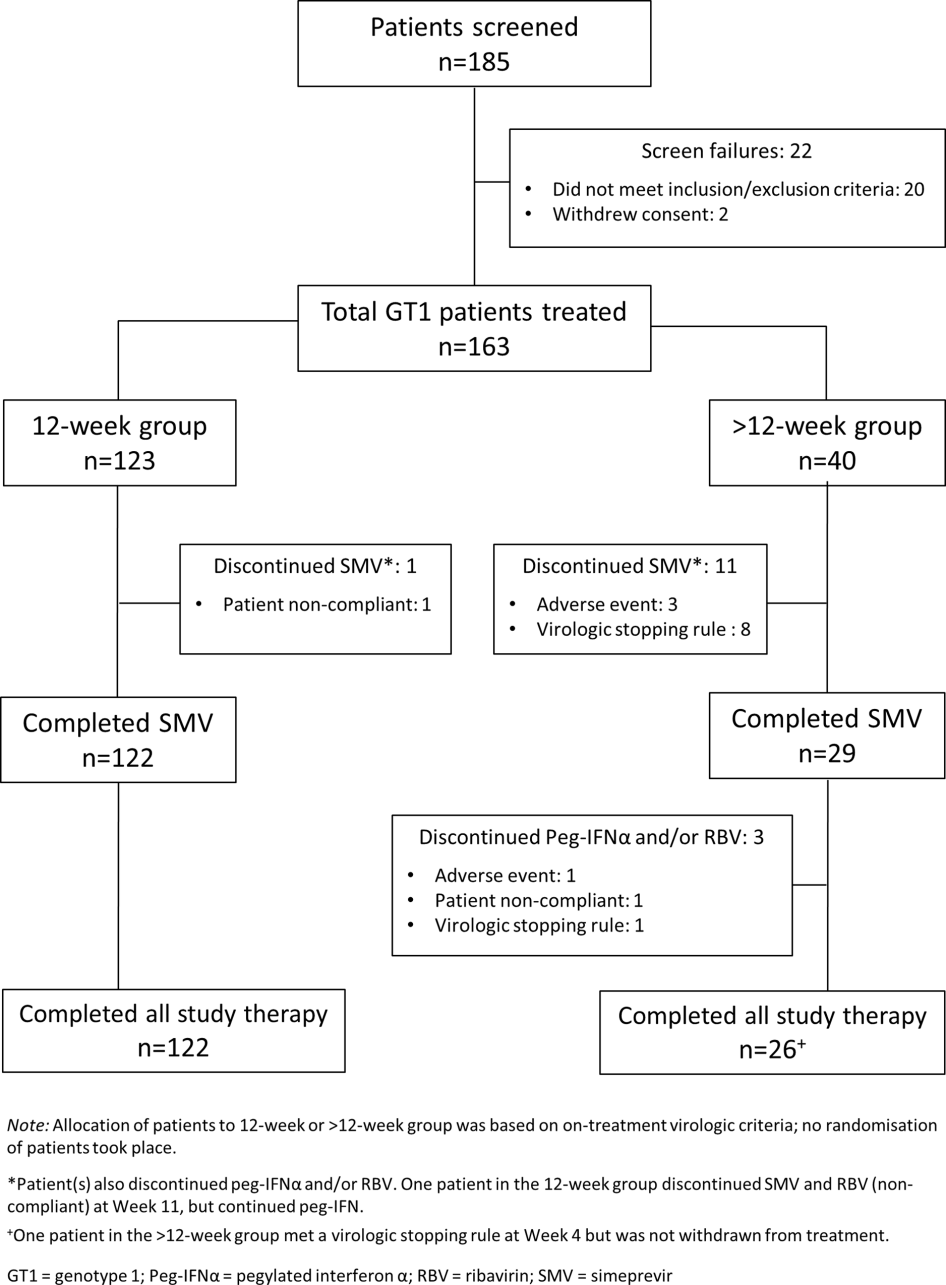
Patient disposition flow chart.

Of 185 patients screened, 88% (163) were treated. Of these, 91% (148/163) completed all study therapy. During the entire treatment period, 7% (12/163) patients discontinued simeprevir, and 9% (15/163) discontinued Peg-IFNα and/or RBV for any reason (**Fig 1**). Seventy-five per cent (123/163) of patients met the criteria for shortening treatment to 12 weeks. Of these, all except one patient, who discontinued simeprevir and RBV at Week 11 due to non-compliance, completed all study treatment. A total of 40 patients were assigned to the >12 week group for analysis; these patients did not met the eligibility criteria for shortened treatment. Of these 40 patients, 73% (29/40) completed the 12 weeks of simeprevir therapy and 65% (26/40) completed all study treatment (**Fig 1**).

### Baseline characteristics and eligibility for the 12-week treatment regimen

Most patients were male and white; median age was 47 years. More patients with *IL28B* CC (32/40; 80%) and CT (73/93; 78%) genotype than TT (18/30; 60%) were eligible for 12-week treatment. More patients in the 12-week *vs*. >12-week group had HCV RNA ≤800,000 IU/mL at baseline (33/123 [27%] *vs*. 3/40 [8%]) (**Table 1**).

**Table 1:** Patient baseline demographics and disease characteristics. Data are n (%) unless otherwise stated. HCV GT1 subtype (Coalesce) is based on the *NS5B* assay, and if not available on LIPA HCV II or Trugene results.

	**12-week group (n = 123)**	**>12-week group (n = 40)**	**Overall (N = 163)**
Male	65 (53)	28 (70)	93 (57)
Race			
White	98/107 (92)	32/33 (97)	130/140 (93)
Age, years, median (min, max)	47 (23, 68)	50 (26, 64)	47 (23, 68)
Body mass index, kg/m^2^, median (range)	25 (17–39)	25 (16–34)	25 (16–39)
HCV GT1 subtype			
1a	49 (40)	18 (45)	67 (41)
With Q80K*	6/47 (13)	5/18 (28)	11/65 (17)
1b	74 (60)	22 (55)	96 (59)
Baseline HCV RNA			
≤800,000 IU/mL	33 (27)	3 (8)	36 (22)
METAVIR fibrosis score^+^			
F0–1	93 (76)	25 (63)	118 (72)
F2	29 (24)	15 (38)	44 (27)
Missing	1 (1)	0	1 (1)
*IL28B* genotype			
CC	32 (26)	8 (20)	40 (25)
CT	73 (59)	20 (50)	93 (57)
TT	18 (15)	12 (30)	30 (18)

*Q80K data unavailable for two patients with GT1a in the 12-week group

^+^Patients were assessed with invasive (liver biopsy; (n = 34)) and/or non-invasive assessment of fibrosis (transient elastography; n = 128), with the worst assessment being used for stage of fibrosis. SD: standard deviation.

Among patients with GT1a HCV (n = 67), the Q80K polymorphism was identified in 17% (11/65) of those with available sequence data. Of these, 55% (6/11) (5/6 with *IL28B* CC genotype) were eligible for 12-week treatment.

The minimal data set of the present study is available in **S2 Text**.

### Efficacy

**Fig 2:**
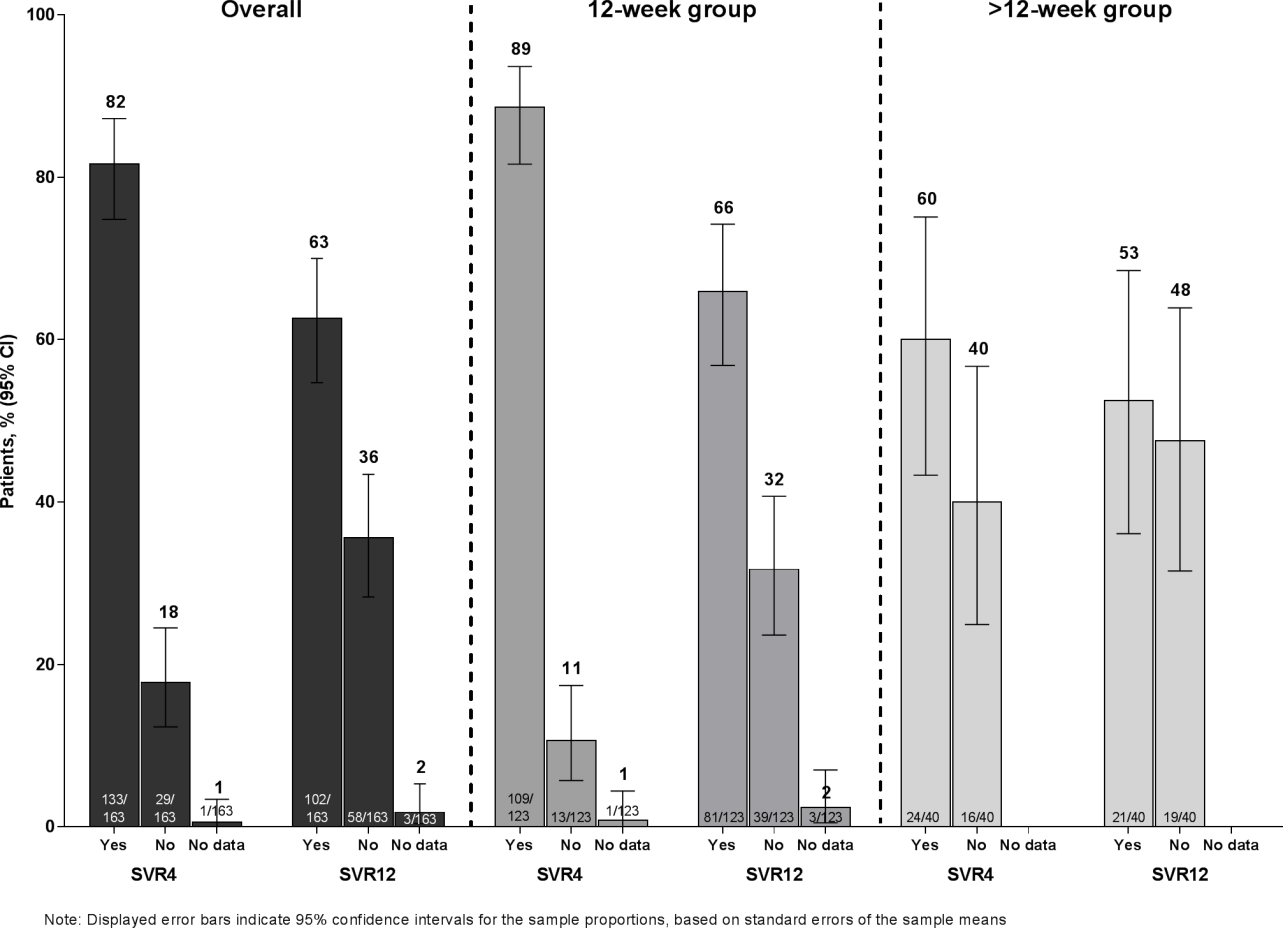
SVR4 and SVR12 rates for all patients, patients receiving 12 weeks of treatment, and patients receiving >12 weeks of treatment.

Overall, 63% (102/163) of patients achieved SVR12. A greater proportion of patients in the 12-week treatment group (81/123 [66%]) than in the >12-week group (21/40 [53%]) achieved SVR12 (**Fig 2**). The study hypothesis of ≥80% SVR12 in the 12-week group was rejected.

**Fig 3:**
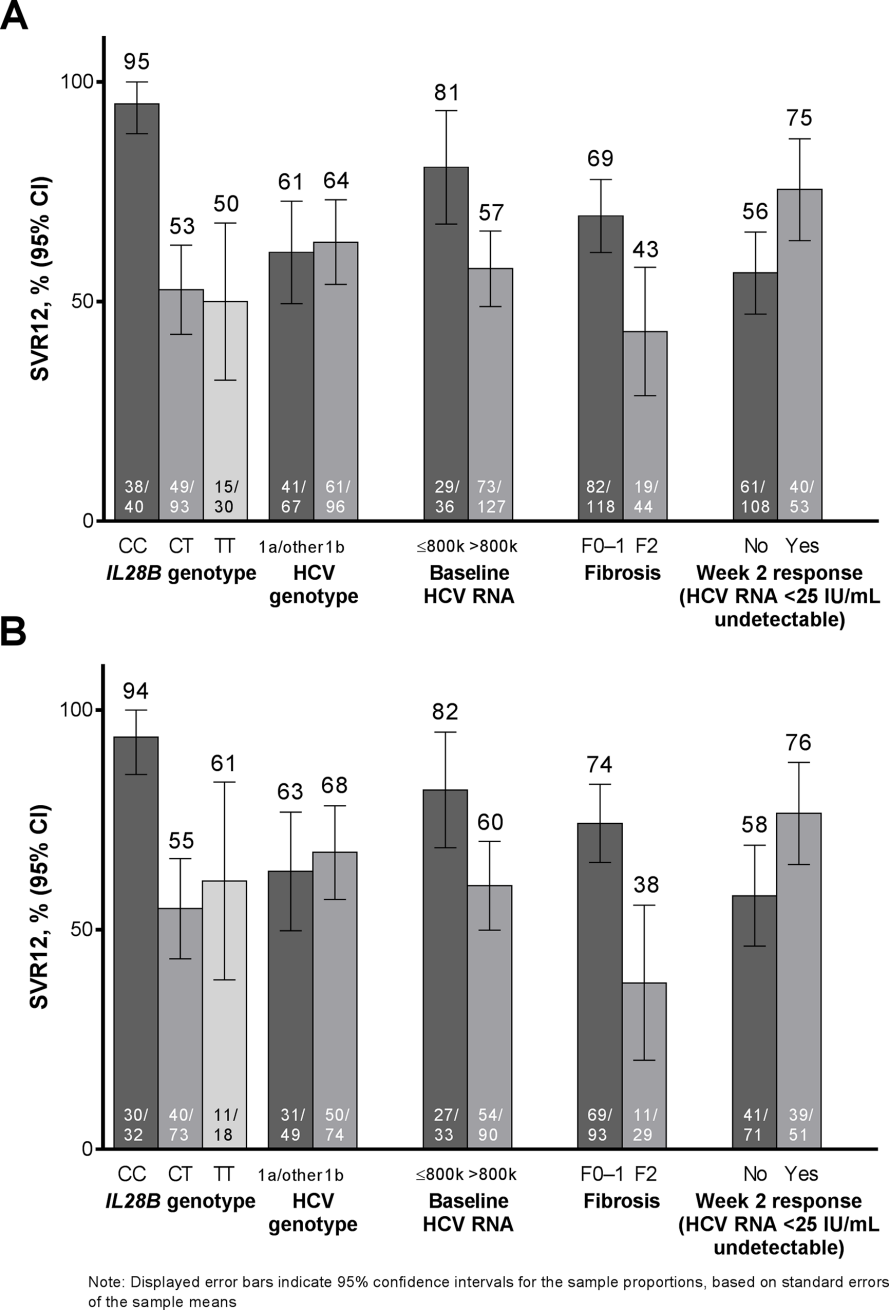
Percentage of **[A]** all patients, and **[B]** patients receiving 12 weeks of treatment who achieved SVR12, according to baseline characteristics and on-treatment factors.

Substantial variation in SVR12 rates according to patient baseline characteristics was observed (**Fig 3A**). Overall, 95% (38/40) of patients with *IL28B* CC genotype achieved SVR12 compared with 52% (64/123) patients with *IL28B* non-CC. Of the *IL28B* CC patients who were treated with the 12-week regimen, 94% (30/32) achieved SVR12. All (8/8) CC patients receiving >12 weeks of treatment achieved SVR12. Overall, 81% (29/36) of patients with baseline HCV RNA ≤800,000 IU/mL achieved SVR12 (27/33 [82%] in the 12-week group) compared with 57% (73/127) of patients with baseline HCV RNA >800,000 IU/mL. Patients with F0–F1 fibrosis achieved higher SVR12 rates compared with patients with F2 fibrosis (69% *vs*. 43% overall) **(Fig 3A**). The difference was more pronounced in the 12-week group (74% *vs*. 38%) (**Fig 3B**). In the 12-week group, 76% of patients with undetectable HCV RNA at Week 2 achieved SVR12, versus 58% of patients with <25 IU/mL detectable (**Fig 3B**). GT1 subtype had no impact on likelihood of achieving SVR12, overall or in the 12-week group.

No patients in the 12-week group experienced viral breakthrough, and all achieved undetectable HCV RNA at the end of treatment. During the 12-week follow-up period, 32% (38/123) of patients experienced viral relapse; another 4 patients relapsed during the 24-week follow-up period. Three patients in the 12-week group had missing SVR12 and 24 data.

In the >12-week group, which notably included all patients with on-treatment virologic failure or those discontinuing treatment due to an AE, on-treatment failure occurred in 25% (10/40) of patients, and 23% (9/40) of patients experienced viral relapse.

Paired baseline and time of failure sequencing data were available in 26 patients who experienced treatment failure in the 12-week group, and six in the >12-week group (32 in total). Of these, 97% (31/32) had a treatment-emergent *NS3* mutation at the time of failure. Consistent with previous data [17] mutations were most frequently observed at positions 168 (n = 24, in total) and 155 (n = 10) (**S2 Appendix**). Of patients with the Q80K polymorphism at baseline, 100% (6/6) in the 12-week group and 40% (2/5) in the >12-week group achieved SVR12.

### Factors associated with SVR12, and with incidence of viral relapse (12-week group only)

**Fig 4:**
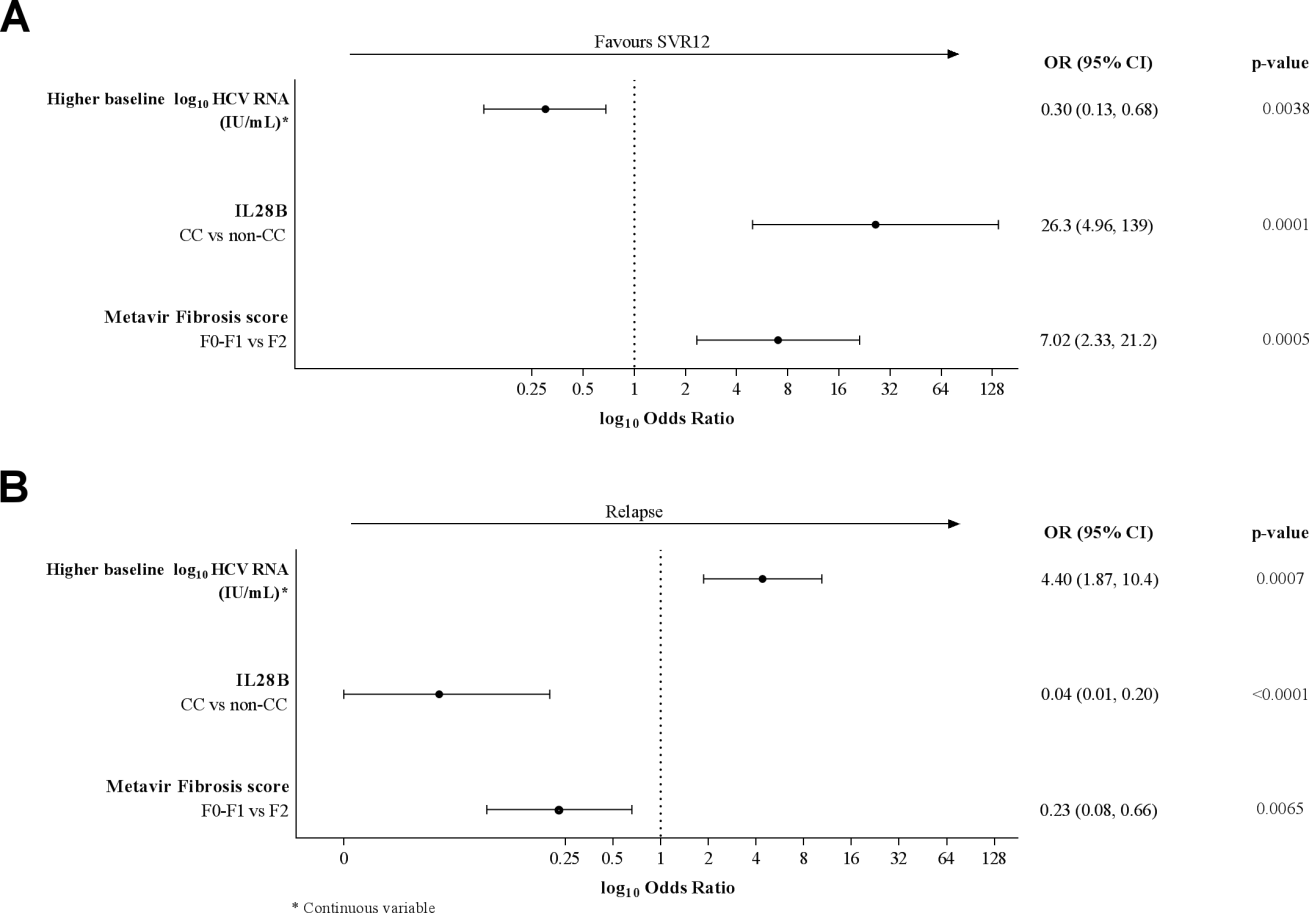
Forest plot showing the results of the final multivariate logistic regression analyses of factors associated with **[A]** SVR12 and **[B]** viral relapse in patients receiving 12 weeks of treatment.

Univariate and multivariate logistic regression analyses were conducted to identify factors associated with SVR12 rates and viral relapse. *IL28B* CC genotype was the factor most strongly associated with SVR12 (Odds ratio [OR], 95% confidence interval [CI]: 26.3 [4.96, 139]) and low relapse rates (OR, 95% CI: 0.04 [0.01, 0.20]) in the final multivariate model (**Fig 4**). High baseline viral load was negatively associated with SVR12 (OR, 95% CI: 0.30 [0.13, 0.68]) and positively associated with viral relapse (OR, 95% CI: 4.40 [1.87, 10.4]). F0–F1 *vs*. F2 fibrosis was positively associated with SVR12 (OR, 95% CI: 7.02 [2.33, 21.2]) and negatively associated with viral relapse (OR, 95% CI: 0.23 [0.08, 0.66]) (**Fig 4**). Week 2 virologic response (<25 IU/mL undetectable *vs*. detectable) was positively associated with SVR12 in the univariate analysis but not the final multivariate model.

When multivariate analysis was conducted in patients with *IL28B* non-CC genotype only (n = 91), the findings were consistent with the overall model (**S1 Fig**): high baseline viral load and fibrosis stage F2 (*vs*. F0-F1) remained significantly associated with viral relapse.

HCV genotype subtype, race, gender and baseline body mass index were not found to be significantly associated with SVR12 rates in all patients or in the non-CC subset.

Classification and regression tree analysis findings supported those of the multivariate analysis (**S3 Appendix**).

### Safety

Among all patients who received 12 weeks or >12 weeks of treatment (n = 163), 94% (154/163) experienced ≥1 AE; 4% (6/163) a serious AE during the entire treatment phase. Only one of the serious AEs, a case of rash in a >12-week patient that started during the PR-only treatment phase, was considered possibly related to simeprevir. Grade 3/4 AEs were experienced by 21% (34/163) of patients (**Table 2**); of these, eight, all Grade 3, were considered possibly related to simeprevir. Four patients, all in the >12-week group, discontinued ≥1 study drug because of an AE; two of these (urinary incontinence; rash) were considered at least possibly related to simeprevir. No clear difference in overall AE rates was observed between patients treated for 12 *vs*. >12 weeks. AEs for which there was a marked difference between the 12- and >12-week groups were: fatigue (31% *vs*. 18%), asthenia (24% *vs*. 35%) and insomnia (18% *vs*. 8%), and the AEs of special interest anemia (11% *vs*. 23%) and rash (17% *vs*. 33%) (**Table 3**). Laboratory analysis findings were consistent with previously-reported data for simeprevir plus PR. Notably, two patients had grade 2 alanine aminotransferase (ALT) elevations during the first 12 weeks of treatment, but no patient had grade 3 or 4 ALT elevation (**S4 Appendix**).

**Table 2:** Summary of patients experiencing AEs (on-treatment) during the entire treatment phase, overall and according to treatment group (12- or >12-week treatment).

	**12-week group (n = 123)**	**>12-week group (n = 40)**	**All patients (N = 163)**
**AE**			
Any AE	117 (95)	37 (93)	154 (94)
Any serious AE	4 (3)	2 (5)	6 (4)
AE leading to discontinuation of ≥1 study drug	0	4 (10)*	4 (2)
**Treatment-related AEs**^**‡**^			
Any study drug	110 (89)	36 (90)	146 (90)
Simeprevir^¶^	68 (55)	20 (50)	88 (54)
Ribavirin	84 (68)	30 (75)	114 (70)
Peginterferon	105 (85)	33 (83)	138 (85)
**Worst grade AE**			
Grade 1	54 (44)	13 (33)	67 (41)
Grade 2	37 (30)	16 (40)	53 (33)
Grade 3	23 (19)	5 (13)	28 (17)
Grade 4	3 (2)	3 (8)	6 (4)

*3 patients discontinued all treatment during the simeprevir+PR phase. One patient discontinued PR during the PR-only phase.

^‡^AEs considered by the investigator to be at least possibly related to specified study drug

^¶^Grade 3 AEs considered by the investigator to be at least possibly related to simeprevir were experienced by 8 (5%) patients, as follows: rash (n = 2), depression (n = 1), asthenia (n = 1), hyponatremia (n = 1), hyperbilirubinemia (n = 1), neutropenia (n = 1), and increase in blood bilirubin (n = 1). No Grade 4 AEs were considered by the investigator to be at least possibly related to simeprevir. AE, adverse event.

**Table 3:** Listing of most frequently-reported AEs (>15% overall) and all AEs of special interest. AE, adverse event; GI, gastrointestinal.

	**12-week group (n = 123)**	**>12-week group (n = 40)**	**All patients (N = 163)**
**Most frequently-reported AEs (>15% of patients overall)**			
Influenza-like illness	47 (38)	12 (30)	59 (36)
Pruritus	43 (35)	12 (30)	55 (34)
Fatigue	38 (31)	7 (18)	45 (28)
Asthenia	29 (24)	14 (35)	43 (26)
Headache	32 (26)	9 (23)	41 (25)
Neutropenia	24 (20)	8 (20)	32 (20)
Dry skin	24 (20)	5 (13)	29 (18)
Rash	17 (14)	7 (18)	24 (15)
Insomnia	22 (18)	3 (8)	25 (15)
**AEs of special interest (groupings of preferred terms)**			
Pruritus (any type)	44 (36)	12 (30)	56 (34)
Upper GI symptoms	27 (22)	8 (20)	35 (21)
Rash (any type)	21 (17)	13 (33)	34 (21)
Neutrophils decrease	25 (20)	8 (20)	33 (20)
Anemia	14 (11)	9 (23)	23 (14)
Dyspnea	19 (15)	4 (10)	23 (14)
Bilirubin increase	10 (8)	1 (3)	11 (7)
Photosensitivity	1 (<1)	0	1 (<1)

### Patient-reported outcomes

Patient-reported outcomes scores at 24 weeks were improved in patients treated for 12 weeks compared with patients treated for >12 weeks, indicative of improved health-related quality of life. These scores were also improved, relative to baseline, in patients achieving *vs*. not achieving SVR12 (**S5 Appendix**).

## Discussion

The observed SVR12 rate among patients in this study who met the criteria and received 12 weeks of treatment with simeprevir plus PR was 66%, and so the primary endpoint of superiority over the minimally acceptable response rate of 80% was not met. Current guidelines recommend 12 weeks of simeprevir plus PR followed by another 12 weeks of PR in treatment-naïve patients and prior PR relapsers. This recommendation is based on the findings of three Phase III studies (QUEST-1, QUEST-2 and PROMISE [7,8,18]) that showed high SVR12 rates (83–91%) in patients treated for 24 weeks with simeprevir plus PR.

Although this study did not find Week 2 response to be sufficient to select patients in whom reduction of simeprevir plus PR treatment duration to 12 weeks is likely to be effective, several baseline host and viral factors (namely mild fibrosis, *IL28B* CC genotype and low baseline viral load) were found to be associated with high efficacy of this shortened regimen.

Among patients with *IL28B* CC genotype, very high SVR rates of 94%, and 100%, respectively were observed in both the 12-week and >12-week groups. This variant is a well-established predictor of SVR with IFN-containing regimens, [19] and this observation is consistent with reports from the QUEST studies [7,8]. Previously, a 12-week telaprevir triple therapy regimen was shown to achieve ~87% SVR in non-cirrhotic patients with *IL28B* CC genotype achieving rapid virologic response at Week 4 [12]. Our analysis also found low HCV RNA at baseline and F0–F1 fibrosis to be associated with an increased likelihood of achieving SVR. An association of advanced fibrosis and cirrhosis with poor response to IFN-based treatment is well-established; [20] our findings support the proposition of a graded impact of fibrosis on treatment efficacy [21].

In IFN-free regimens combining at least two direct-acting antivirals, impact of *IL28B* genotype on efficacy is almost exclusively observed where the treatment duration is too short: for example, the C-SWIFT study showed lower SVR rates with the 6-week grazoprevir/elbasvir/sofosbuvir regimen in patients with non-CC *vs*. CC allele [22].

In the current analysis, aside from three patients with missing data at the SVR12 timepoint, all instances of treatment failure in the 12-week group resulted from viral relapse, mainly occurring after the SVR4 timepoint. On-treatment virologic response data alone are therefore not sufficient to identify patients in whom shortening of treatment to 12 weeks is appropriate.

Baseline characteristics identified by multivariate logistic regression as associated with relapse were consistent with those associated with increased SVR12 rates: *IL28B* genotype, baseline HCV RNA, and fibrosis score.

In the >12-week group, 53% (21/40) patients achieved SVR12. This low rate may partly be explained by the assignment to the >12-week group of those patients experiencing on-treatment virologic failure on or before Week 8, or discontinuing treatment due to AEs. Thus, findings in the 24-week treatment group in this study cannot be compared with patients treated with simeprevir plus PR for 24 weeks in the QUEST studies.

As previously discussed, *IL28B* CC genotype was strongly associated with SVR. Furthermore, by combining response data with relevant patient-specific factors it is possible to identify individuals with GT1 in whom shortening of treatment would be beneficial. For instance, per the findings of our classification and regression tree analysis, among non-CC patients with F0-F1 fibrosis and HCV RNA <1,020,000 IU/mL who received 12 weeks of treatment, 89% (24/27) achieved SVR12, although this finding should be interpreted with caution due to the small sample size.

Our safety and laboratory analysis findings were similar to those of other simeprevir studies, with a simeprevir plus PR AE profile similar to that of PR [16]. Most patients (94%) experienced at least one on-treatment AE. However, only 34 (21%) patients experienced a Grade 3 or 4 AE, of whom eight (5%) were potentially related to simeprevir (all Grade 3). No clear trend in AE frequency between the 12- and >12-week groups was seen, although patient-reported findings did differ between the groups. This may be due to the limited sample size or higher discontinuation rate in the >12-week group, or the inclusion in the study of patients with relatively mild fibrosis, who may tolerate PR better than patients with more advanced disease [16].

This study represents the first assessment of a 12-week total treatment duration for simeprevir plus PR, with eligibility for shortened therapy determined by on-treatment virologic response. The inclusion of findings from a multivariate analysis of baseline factors potentially associated with SVR12 rates and viral relapse, demonstrates the informative value of these baseline factors. However, the logistic regression analysis was *post-hoc* and therefore not considered in the power calculations; hence, some subgroups included in this analysis comprised few patients and should be interpreted with caution. Another potential limitation is the sensitivity of the assay used (LLOQ 25 IU/mL). Further investigation of the possibility that using a more sensitive assay could enable better-informed selection of patients to receive a 12-week regimen may be worthwhile [23].

Although approved short-duration DAA regimens are currently available, there remains a need for cost-effective, short-duration treatment options for HCV GT1 patients. Due to the high costs of currently approved DAA regimens, cost-effective treatment options are limited in low socioeconomic areas. Therefore, this regimen may be of particular benefit in these regions until further DAAs are approved. Although the *a priori* defined SVR12 threshold of 80% was not met in patients who received the 12-week regimen of simeprevir plus PR treatment, the findings of this study suggest that treatment-naïve patients with HCV GT1 and favourable baseline characteristics–particularly *IL28B* CC genotype–who display early virologic response, may benefit from this shortened treatment duration.

## Supporting Information

S1 TREND Checklist TREND checklist. (PDF)

S1 Text List of Institutional review boards. (DOCX)

S1 Appendix Patient inclusion/exclusion criteria. (DOCX)

S2 Appendix Emerging mutations at time of virologic failure of treatment. (DOCX)

S3 Appendix Classification and regression tree analysis. (DOCX)

S4 Appendix Laboratory tests. (DOCX)

S5 Appendix Patient-reported outcomes. (DOCX)

S1 Protocol Study protocol. (PDF)

S1 Dataset Minimal data set. (ZIP)

S1 Fig Forest plot showing the results of the final multivariate logistic regression analyses of factors associated with [A] SVR12 (*n =* 91) and [B] viral relapse (*n =* 90) in patients with *IL28B* CT or TT genotype receiving 12 weeks of treatment. (DOCX)
