# Supplementary material for: An Open-Label Trial of 12-Week Simeprevir plus Peginterferon/Ribavirin (PR) in Treatment-Naïve Patients with Hepatitis C Virus (HCV) Genotype 1 (GT1)
Source: PLoS One. 2016 Jul 18;11(7):e0158526. doi: 10.1371/journal.pone.0158526 (PMC4948848; doi:10.1371/journal.pone.0158526)
Supplement: S1 Text — (DOCX) [file pone.0158526.s002.docx]

**S1 Text – List of Institutional review boards**

1. Ethikkommission der Medizinischen Universität Wien, Vienna, Austria

2. Commission d’Éthique Biomédicale Hospitalo-Facultaire, Brussels, Belgium

3. Comité de Protection des Personnes Ile-de-France IV, Paris, France

4. Segreteria Tecnico Scientifica Comitato di Bioetica, Palermo, Italy

5. Comitato Etico Policlinico Umberto I, Roma, Italy

6. Comitato Etico Indipendente Fondazione Policlinico Tor Vergata, Roma, Italy

7. Comitato Etico Azienda Ospedaliero-Universitaria di Parma, Parma, Italy

8. Comitato Etico di Area Vasta Nord-Ovest per la sperimentazione clinica, Pisa, Italy

9. Ethik-Kommission des Landes Berlin, Berlin, Germany

10. Ethik-Kommission der Ärztekammer Hamburg, Hamburg, Germany

11. Hospital Universitari Vall D’Hebrón - Unidad de Soporte al CEIC (SCEI) - Vall d’Hebron Institut de Recerca (VHIR), Barcelona, Spain

12. West of Scotland Research Ethics Service, Glasgow, UK

13. Centralna Komisja Bioetyczna - Komisja Bioetyczna Uniwersytetu Mikołaja Kopernika w Toruniu przy Collegium Medicum im, Bydgoszcz, Poland
