# Supplementary material for: An Open-Label Trial of 12-Week Simeprevir plus Peginterferon/Ribavirin (PR) in Treatment-Naïve Patients with Hepatitis C Virus (HCV) Genotype 1 (GT1)
Source: PLoS One. 2016 Jul 18;11(7):e0158526. doi: 10.1371/journal.pone.0158526 (PMC4948848; doi:10.1371/journal.pone.0158526)
Supplement: S1 Appendix — (DOCX) [file pone.0158526.s003.docx]

**S1 Appendix – Patient Inclusion/Exclusion Criteria**

**Inclusion Criteria**

Eligible patients had fibrosis score F0–F2 (METAVIR or equivalent) confirmed by liver biopsy or non-invasive assessment (transient elastography). For non-invasive fibrosis assessment, the following cut-offs were defined: FibroScan™ F0–F1 <6.9 kPa, F2 <8 kPa;^1,2^ Magnetic Resonance (MR) elastography F0–F1 <2.5 kPa; F2 <3.1 kPa;^3^ for other methods, such as Shear Wave Elastography, the advice of the sponsor was taken.

Patients were included irrespective of NS3 Q80K status and *IL28B* genotype.

**Exclusion Criteria**

Exclusion criteria included: liver disease stage F3/F4 or previous or current hepatic decompensation; chronic liver disease of a non-HCV etiology; co-infection with hepatitis B virus or HIV; significant laboratory abnormalities (including platelet count below lower limit of laboratory normal range) or other medical disorders; pregnancy or breast-feeding; and male or female patients planning on conceiving within 6 months of the last dose of RBV.

[1] Castéra L, Foucher J, Bernard PH, Carvalho F, Allaix D, Merrouche W et al. Pitfalls of liver stiffness measurement: a 5-year prospective study of 13,369 examinations. Hepatology 2010; 51: 828–35.

[2] Ferraioli G, Tinelli C, Dal Bello B, Zicchetti M, Lissandrin R, Filice G et al. Performance of liver stiffness measurements by transient elastography in chronic hepatitis. World J Gastroenterol 2013; 7: 49–56.

[3] Huwart L, Salameh N, Annet L, Peeters F, ter Beek L, Sinkus R et al. Comparison of MR elastography and FibroScan for the non-invasive assessment of liver fibrosis. Proc Intl Soc Mag Reson Med 2007; 15: 216.
