# Supplementary material for: An Open-Label Trial of 12-Week Simeprevir plus Peginterferon/Ribavirin (PR) in Treatment-Naïve Patients with Hepatitis C Virus (HCV) Genotype 1 (GT1)
Source: PLoS One. 2016 Jul 18;11(7):e0158526. doi: 10.1371/journal.pone.0158526 (PMC4948848; doi:10.1371/journal.pone.0158526)
Supplement: S2 Appendix — (DOCX) [file pone.0158526.s004.docx]

**S2 Appendix**: Emerging *NS3* mutations at time of virologic failure of treatment for patients receiving **A** 12 weeks of treatment; **B** >12 weeks of treatment; for whom paired baseline and time of failure sequencing data are available

**A**

| **Genotype** | **1a (n = 49)** | **1b (n = 74)** | **Overall (N = 123)** |
| --- | --- | --- | --- |
| Failure, n | 19 | 26 | 45 |
| Sequencing data available, n | 11 | 15 | 26 |
| Any emerging mutation, n (%) | 10 (91) | 15 (100) | 25 (96) |
| **Without Q80K at baseline, n (%)** | 10 (91) | 15 (100) | 25 (96) |
| **Specific emerging mutations, n (%)** |  |  |  |
| D168V | 2 (18) | 8 (53) | 10 (38) |
| D168A | 2 (18) | 1 (7) | 3 (12) |
| R155K | 3 (27) | 0 | 3 (12) |
| Q80R + D168E/V | 0 | 1 (7) | 1 (4) |
| R155Q + D168V | 0 | 2 (13) | 2 (8) |
| D168A + V170I | 0 | 1 (7) | 1 (4) |
| D168H | 0 | 1 (7) | 1 (4) |
| Q80K + R155K + D168A | 1 (9) | 0 | 1 (4) |
| Q80L + R155K | 1 (9) | 0 | 1 (4) |
| S122T | 0 | 1 (7) | 1 (4) |
| T54S + R155K | 1 (9) | 0 | 1 (4) |

**B**

| **Genotype** | **1a (n = 18)** | **1b (n = 22)** | **Overall (N = 40)** |
| --- | --- | --- | --- |
| Failure, n | 8 | 11 | 19 |
| Sequencing data available, n | 3 | 3 | 6 |
| Any emerging mutation, n (%) | 3 (100) | 3 (100) | 6 (100) |
| **Without Q80K at baseline, n (%)** | 2 (67) | 3 (100) | 5 (83) |
| **Specific emerging mutations, n (%)** |  |  |  |
| D168V | 1 (33) |  | 1 (17) |
| Q80R + D168E/V |  | 1 (33) | 1 (17) |
| D168E/V |  | 1 (33) | 1 (17) |
| Q80R + D168E |  | 1 (33) | 1 (17) |
| R155K + D168E + L175F | 1 (33) |  | 1 (17) |
| **With Q80K at baseline, n (%)** | 1 (33) |  | 1 (17) |
| R155K | 1 (33) |  | 1 (17) |

*Note:* Mutations at the following *NS3* amino acid positions were considered: 36, 41, 43, 54, 55, 80, 107, 122, 132, 138, 155, 156, 158, 168, 169, 170, 174, 175.
