# Supplementary material for: An Open-Label Trial of 12-Week Simeprevir plus Peginterferon/Ribavirin (PR) in Treatment-Naïve Patients with Hepatitis C Virus (HCV) Genotype 1 (GT1)
Source: PLoS One. 2016 Jul 18;11(7):e0158526. doi: 10.1371/journal.pone.0158526 (PMC4948848; doi:10.1371/journal.pone.0158526)
Supplement: S3 Appendix — (DOCX) [file pone.0158526.s005.docx]

**S3 Appendix – Classification and Regression Tree Analysis**

Classification and regression tree analysis of factors associated with SVR12 in patients receiving 12 weeks of treatment was performed using SAS^®^ JMP. Only those factors that were included in the final multivariate model were included in the regression tree analysis: *IL28B* genotype, HCV RNA at baseline, and METAVIR fibrosis score.

**Figure:** Outcomes of classification and regression tree analysis.


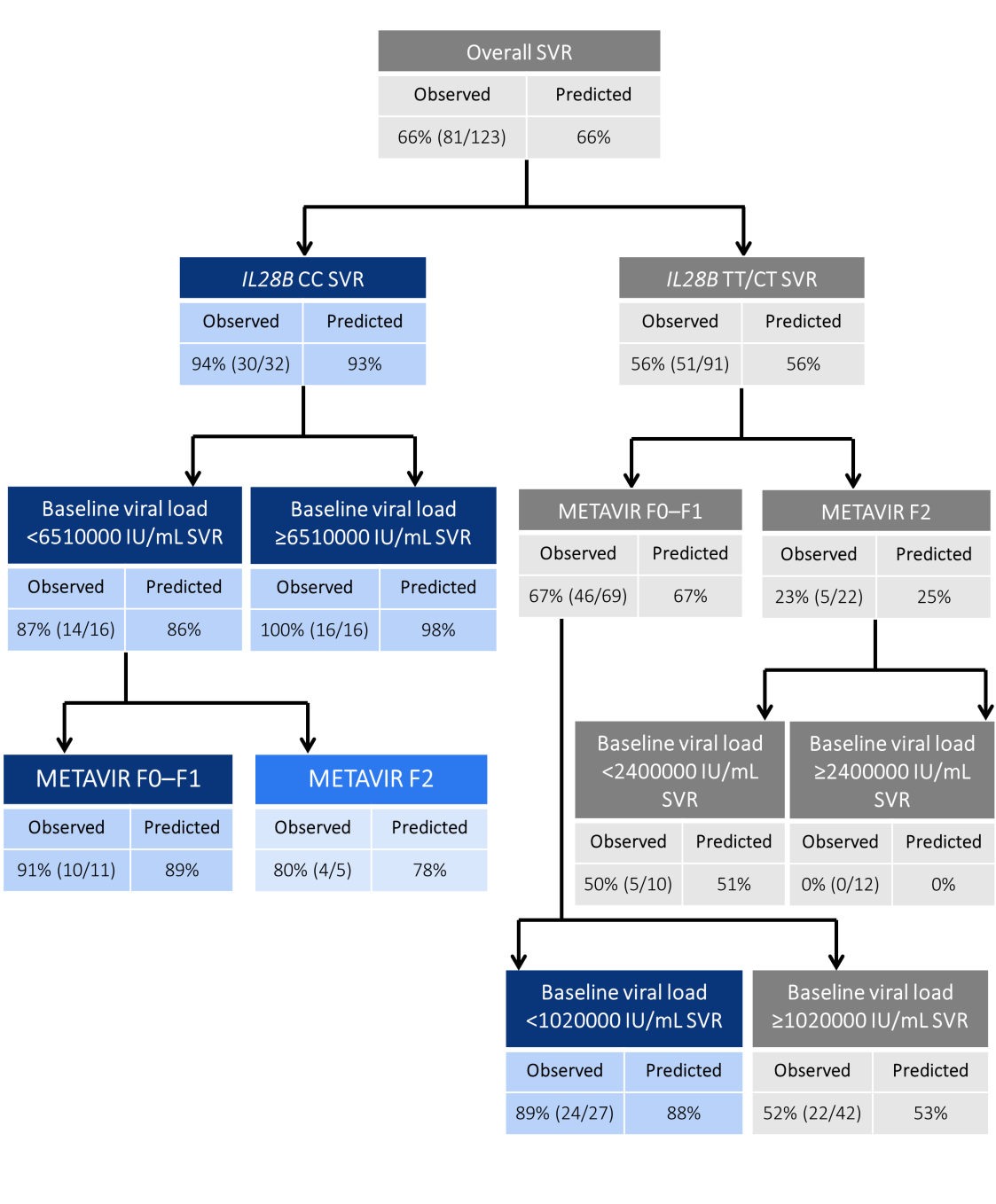


Note: Dark blue boxes indicate predicted SVR12 ≥80%; light blue boxes indicate predicted SVR12 70–79%; grey boxes SVR <70%

Of 16 patients with *IL28B* CC genotype and baseline viral load ≥6,510,000 IU/mL SVR, all of whom achieved SVR12, 13 were F0–F1, two were F2, and one was unknown.
