# Supplementary material for: An Open-Label Trial of 12-Week Simeprevir plus Peginterferon/Ribavirin (PR) in Treatment-Naïve Patients with Hepatitis C Virus (HCV) Genotype 1 (GT1)
Source: PLoS One. 2016 Jul 18;11(7):e0158526. doi: 10.1371/journal.pone.0158526 (PMC4948848; doi:10.1371/journal.pone.0158526)

**S4 Appendix – Laboratory tests**

A reduction in mean levels of neutrophils and precursors below the lower limit of normal (LLN), which persisted throughout most of the study period, was observed. Over the entire treatment period, neutropenia was reported as an adverse event in 20% (33/163) of patients; all but one of these reports occurred during the first 12 weeks of treatment. Around half of all reports of neutropenia (18/33) were Grade 3/4 adverse events.

Anemia was reported as an adverse event in 14% (23/163) of patients. In all but three of these, this occurred during the simeprevir plus PR phase. Incidence of anemia was lower in the 12-week group than in the >12-week group. No cases of Grade 3/4 anemia were observed. Five instances of thrombocytopenia were reported (3%), of which one was deemed to be a Grade 3 adverse event; all of these occurring during the first 12 weeks of treatment. Overall reductions in hemoglobin and platelet levels from baseline were observed, but mean levels stayed within the normal range.

Increases in bilirubin were reported during the first 12 weeks of treatment in 10 (8%) patients in the 12-week treatment group and one (3%) in the >12-week group. Two Grade 3 cases of increased bilirubin were reported.

Mean on-treatment and end-of-treatment (EOT) laboratory values are shown in the **Figure**.

**Figure:** Mean (SE) on-treatment and end-of-treatment (EOT) laboratory measures in patients receiving 12 and >12 weeks’ treatment: **A** hemoglobin (g/L); **B** neutrophils and precursors (x10^9^/L); **C** platelets (x10^9^/L); **D** bilirubin (µmol/L)

**A**

*
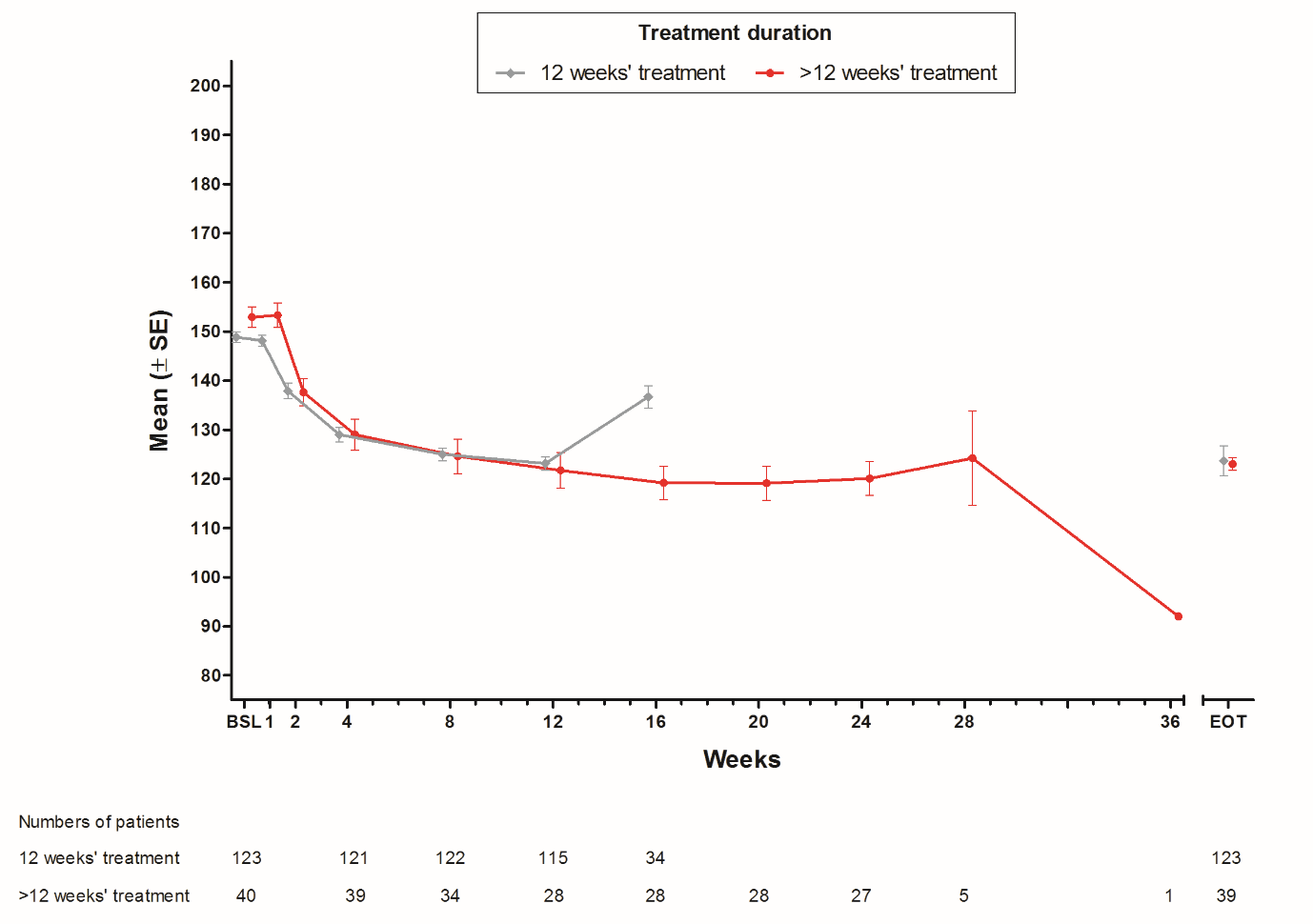
*

**B**

*
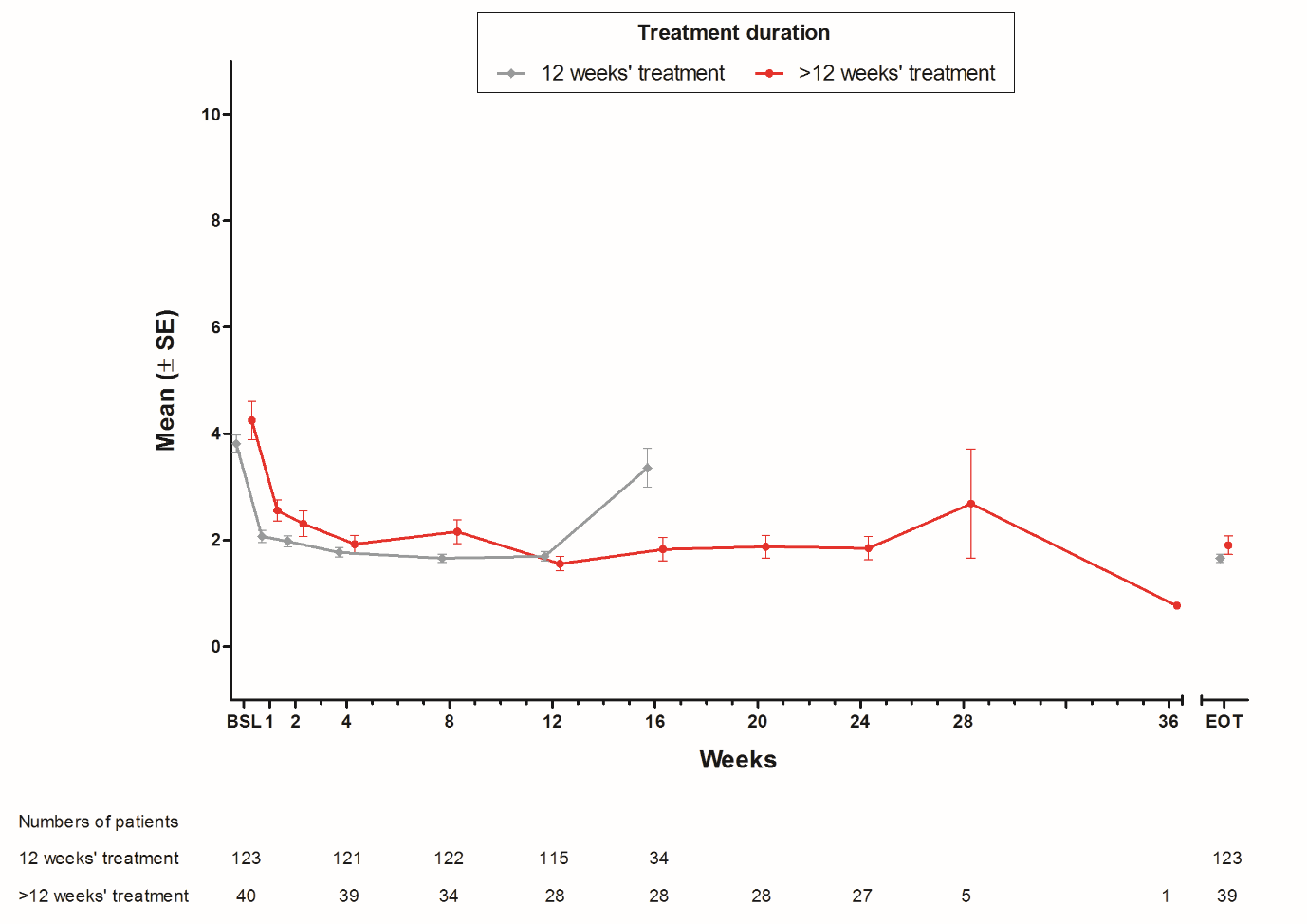
*

**C**

*
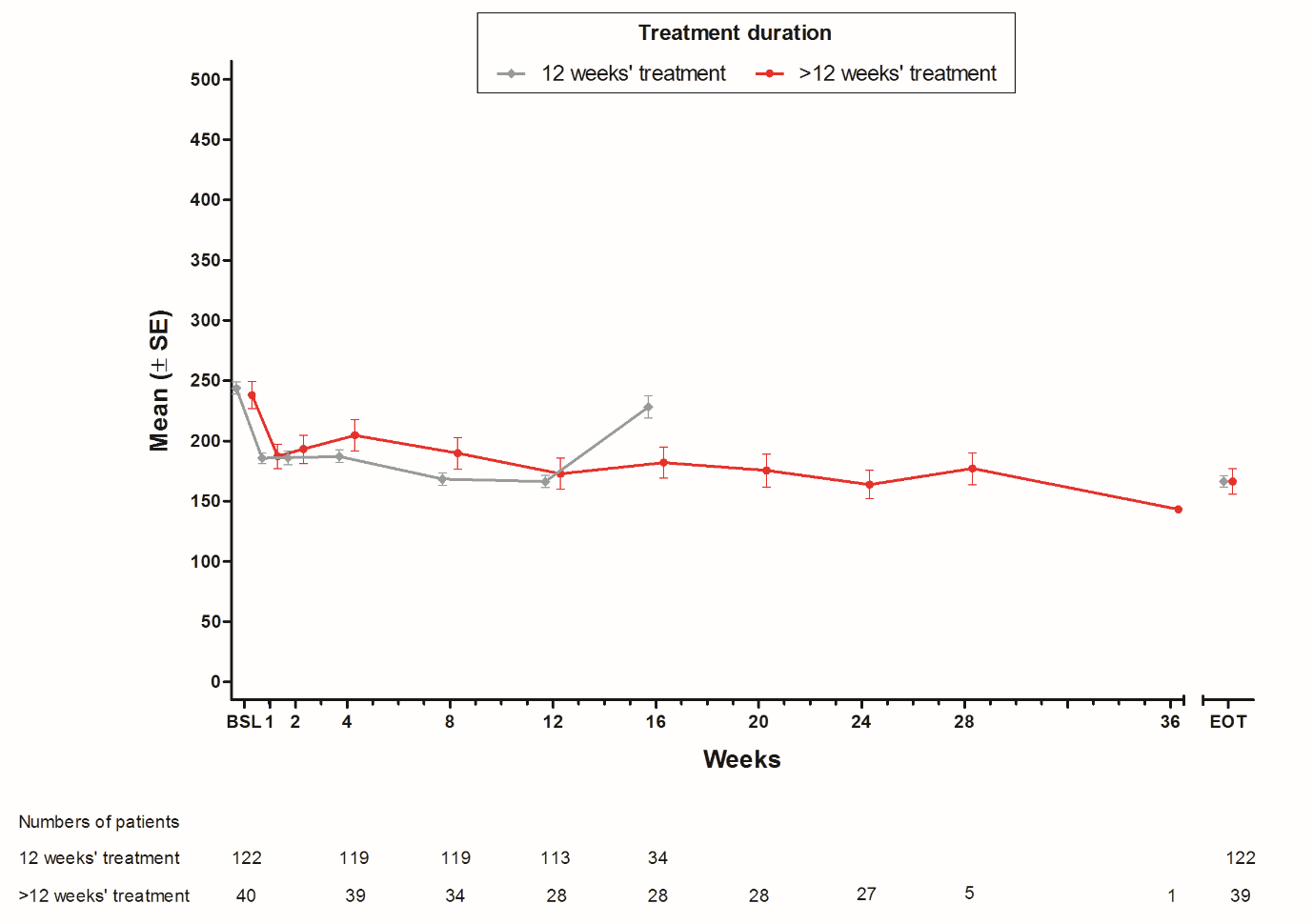
*

**D**


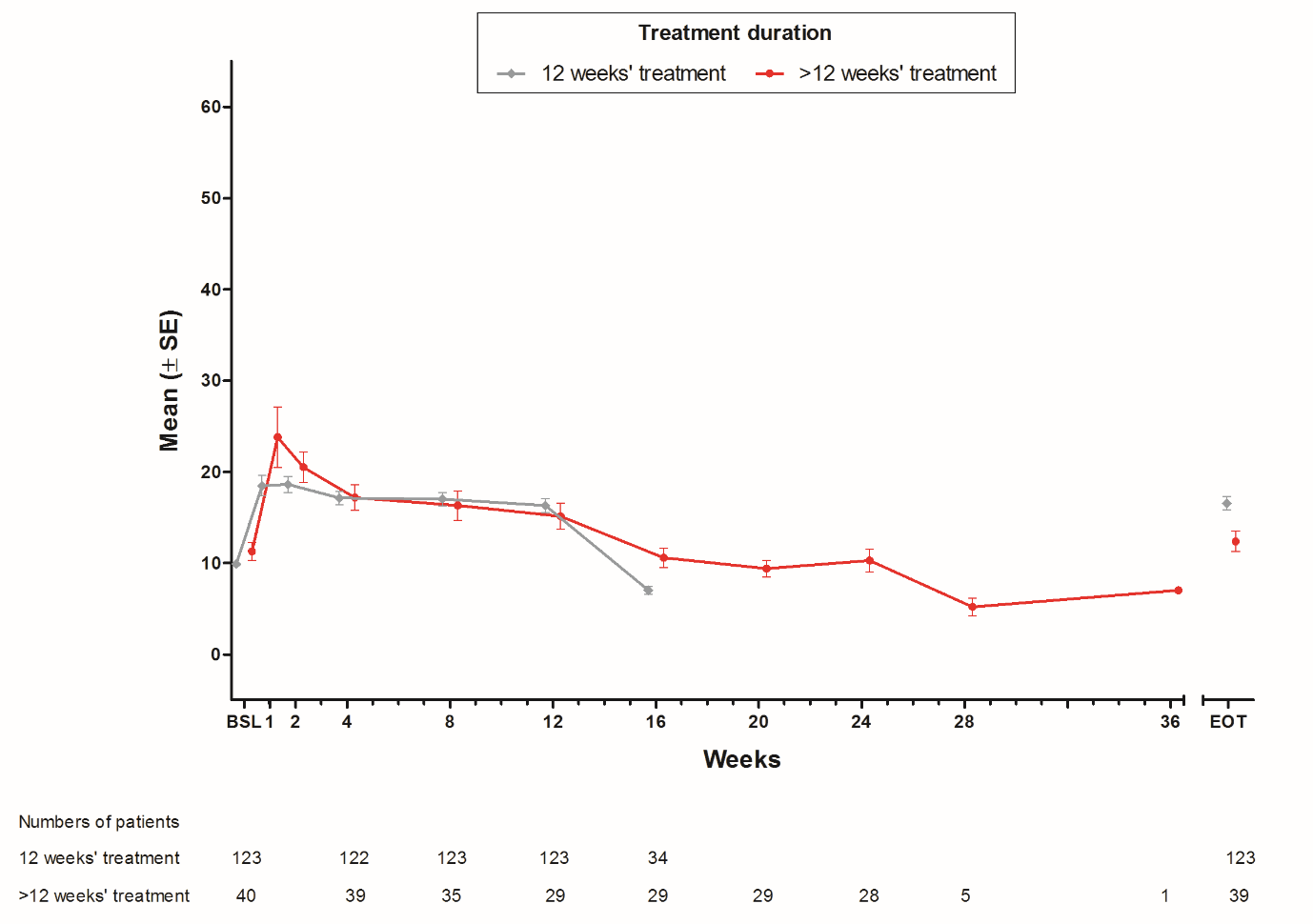

Supplement: S4 Appendix — (DOCX) [file pone.0158526.s006.docx]
