# Supplementary material for: An Open-Label Trial of 12-Week Simeprevir plus Peginterferon/Ribavirin (PR) in Treatment-Naïve Patients with Hepatitis C Virus (HCV) Genotype 1 (GT1)
Source: PLoS One. 2016 Jul 18;11(7):e0158526. doi: 10.1371/journal.pone.0158526 (PMC4948848; doi:10.1371/journal.pone.0158526)
Supplement: S5 Appendix — (DOCX) [file pone.0158526.s007.docx]

**S5 Appendix – Patient-reported Outcomes**

*Methods*

Patients completed questionnaires (Fatigue Severity Score (FSS)^1^ and Center for Epidemiologic Studies Depression Scale (CES-D)^2^ to document changes in the severity of symptoms associated with HCV and its treatment. Patients also rated their degree of impairment in daily activities, work productivity and absenteeism (using the Work Productivity and Activity Impairment (WPAI) questionnaire for hepatitis C^3^), and rated their health status and quality of life (using the EuroQol 5-dimension questionnaire [EQ-5D]^4^). Finally, severity of HCV-related symptoms and their impact on work/school attendance and daily activities were assessed as an exploratory objective of this study using a newly developed HCV Symptom & Impact Questionnaire (HCV-SIQ version 3). Psychometric analysis to develop scoring and evaluate the measurement properties of the HCV-SIQ scores will be reported in a separate publication. Descriptive statistics at baseline, and changes from baseline during and at the end of the treatment period are reported for each treatment group and for patients who achieved SVR12 compared with those who did not.

*Results*

Baseline PRO scores were indicative of high levels of fatigue, depressive symptoms and impairment in work and daily activities. Scores across the treatment period for the FSS, CES-D, WPAI, and EQ-5D questionnaires displayed a similar pattern; each worsening between baseline and Week 4 in both treatment groups. In patients who received the 12-week treatment regimen, mean Week 24 scores were equivalent to, or improved from, baseline (**Figure 1**). The findings were consistent with those of an analysis of pooled data from previous clinical studies of simeprevir plus PR,^5^ and confirmed that the addition of simeprevir to PR is not associated with impaired tolerability compared with PR alone.

Patients who achieved SVR12 were more likely to report improved scores relative to baseline than those without SVR12 (**Figure 2**).

[1] Krupp LB, LaRocca NG, Muir-Nash J, Steinberg AD. The fatigue severity scale. Application to patients with multiple sclerosis and systemic lupus erythematosus. Arch Neurol 1989; 46: 1121–3.

[2] Radloff LS. The CES-D scale: a self-report depressionn scale for research in the general population. Appl Psych Measur 1977; 1: 385–401.

[3] Reilly MC, Zbrozek AS, Dukes EM. The validity and reproducibility of a work productivity and activity impairment instrument. Pharmacoeconomics 1993;4:353–65.

[4] Euroqol Group. Euroqol–a new facility for the measurement of health-related quality of life. *Health Pol* 1990; 16: 199–208.

[5] Scott J, et al. Simeprevir added to peginterferon and ribavirin lessens time with fatigue, depressive symptoms and functional limitations in patients with chronic hepatitis C compared with peginterferon and ribavirin: results from 1161 patients in the QUEST-1, QUEST-2 and PROMISE studies. J Viral Hepat 2014; 22: 639–650.

**Figure 1**: Change from baseline in mean (± SE) scores for PROs in patients receiving 12 and >12 weeks’ treatment: **A** FSS; **B** CES-D; **C** WPAI daily activity impairment; **D** WPAI total work impairment; **E** EQ-5D VAS, **F** EQ-5D Valuation Index

**A**

*
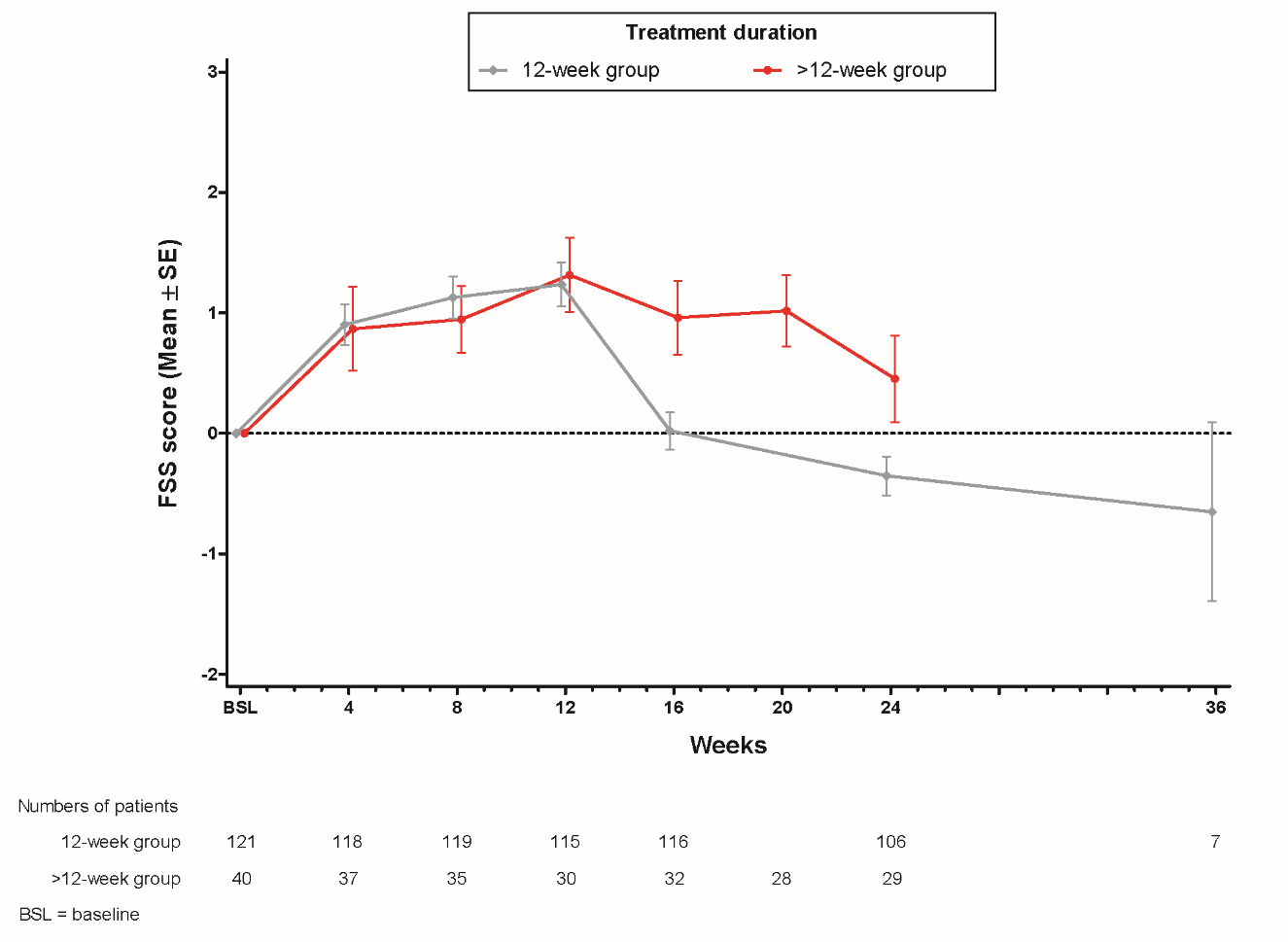
*

**B**

*
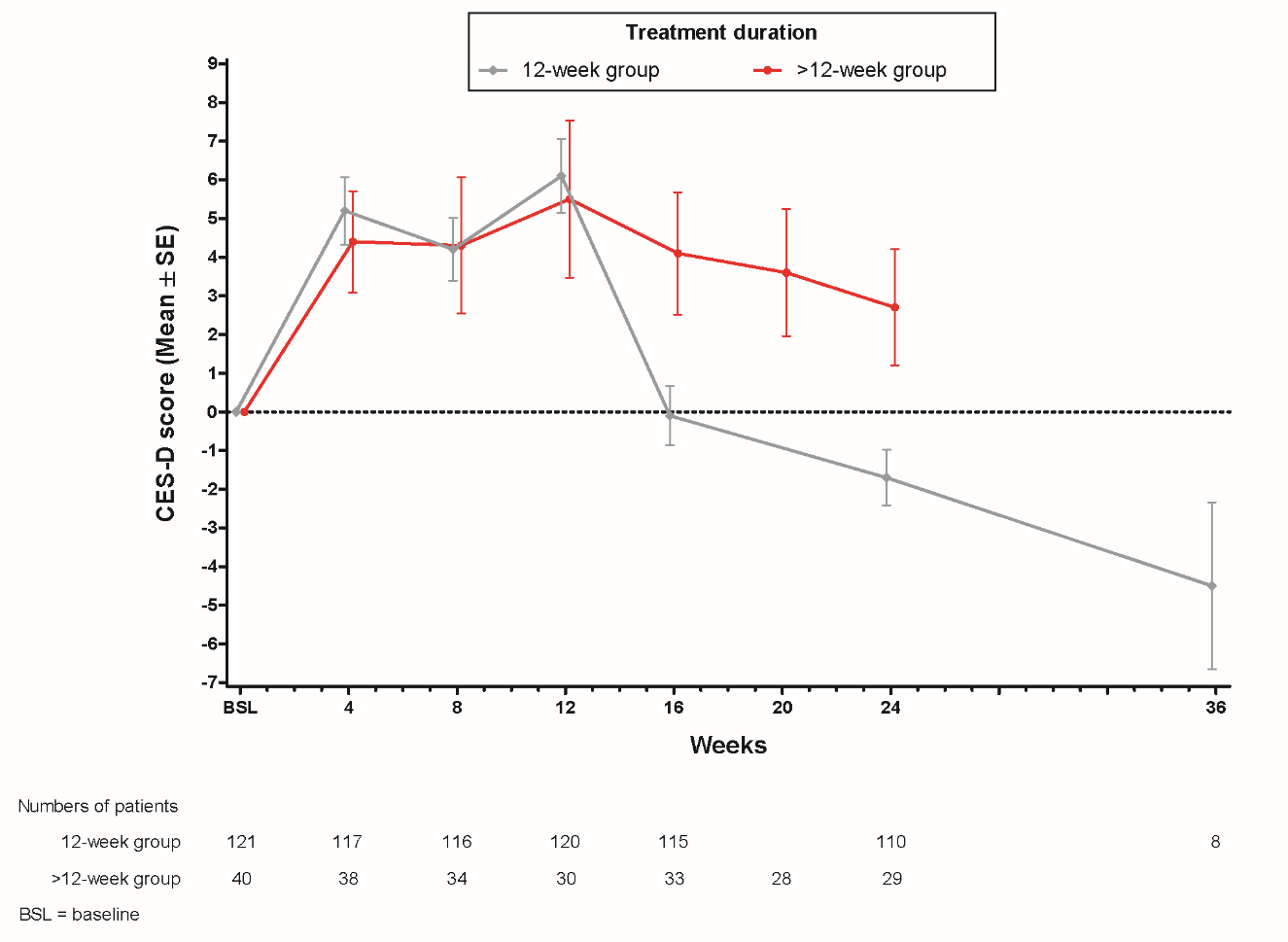
*

**C**

*
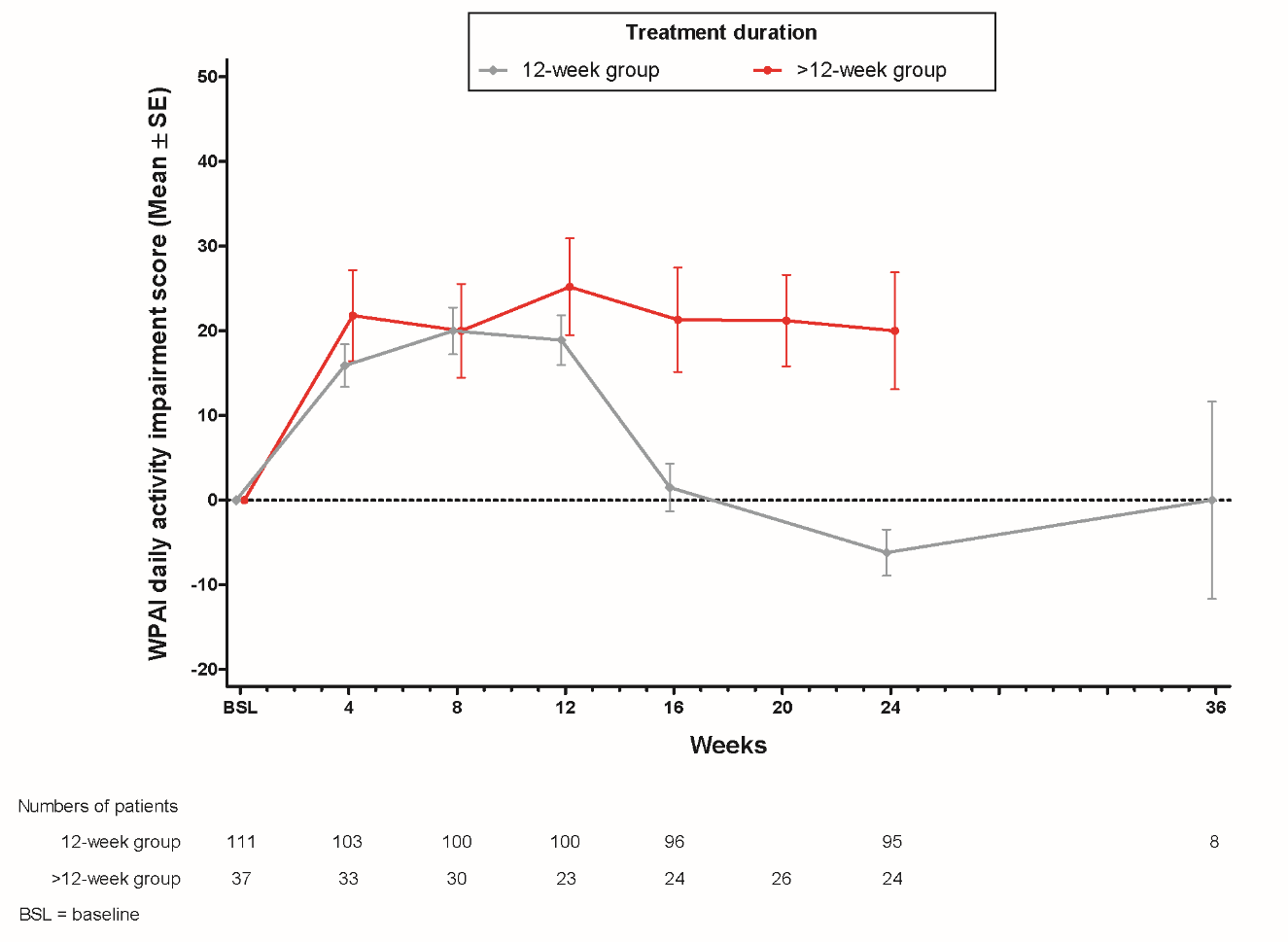
*

**D**

*
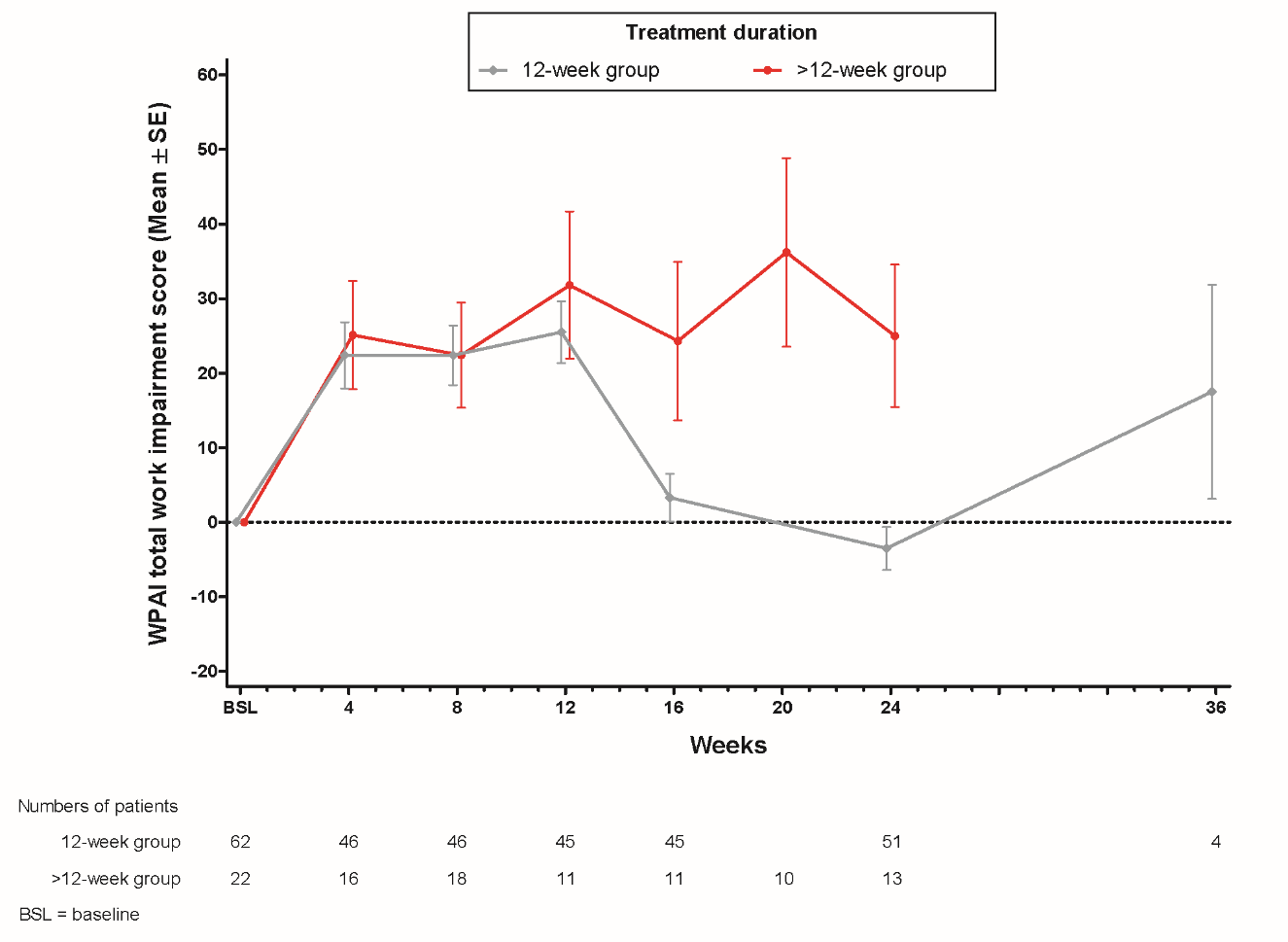
*

**E**

*
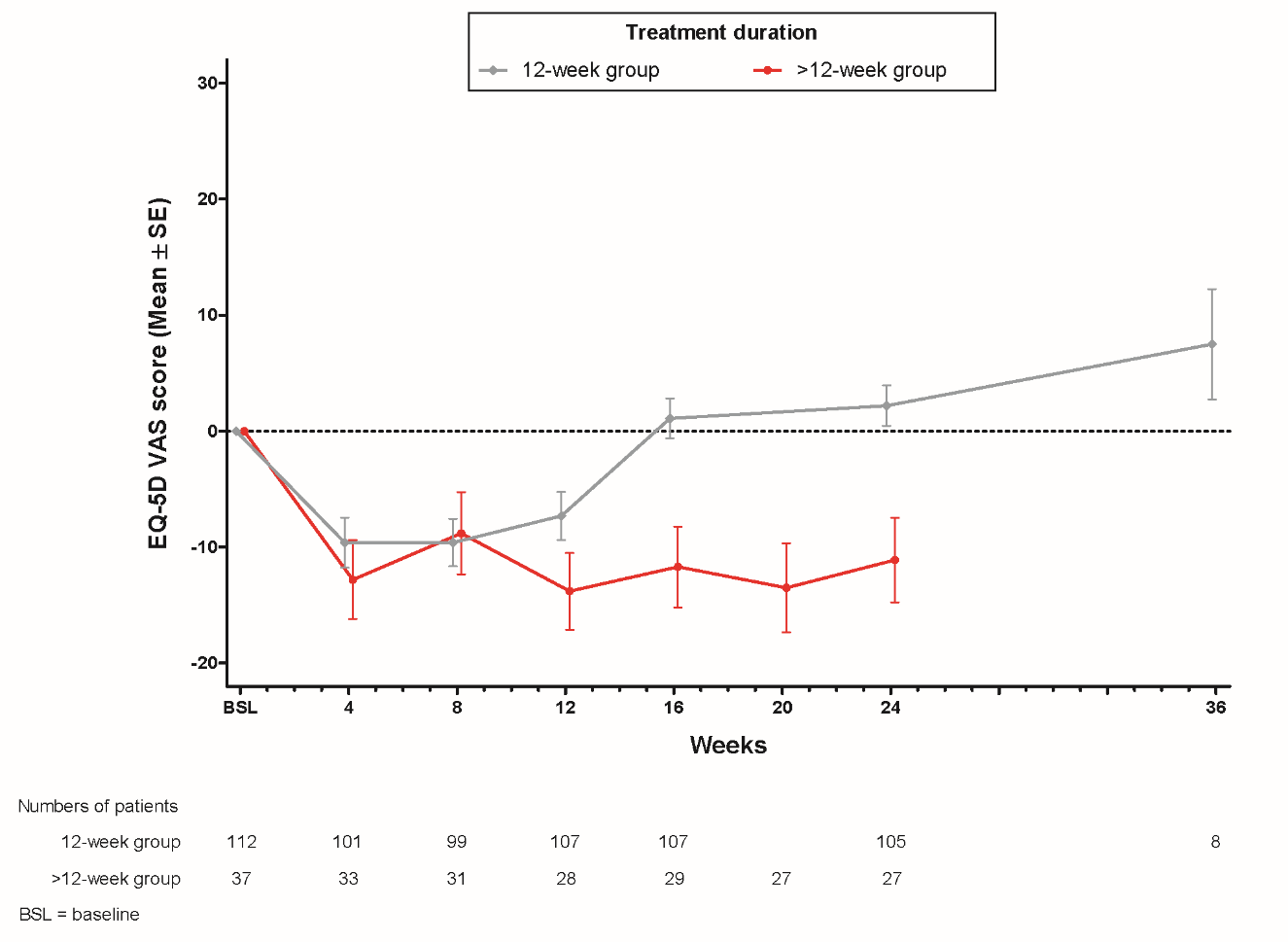
*

**F**

*
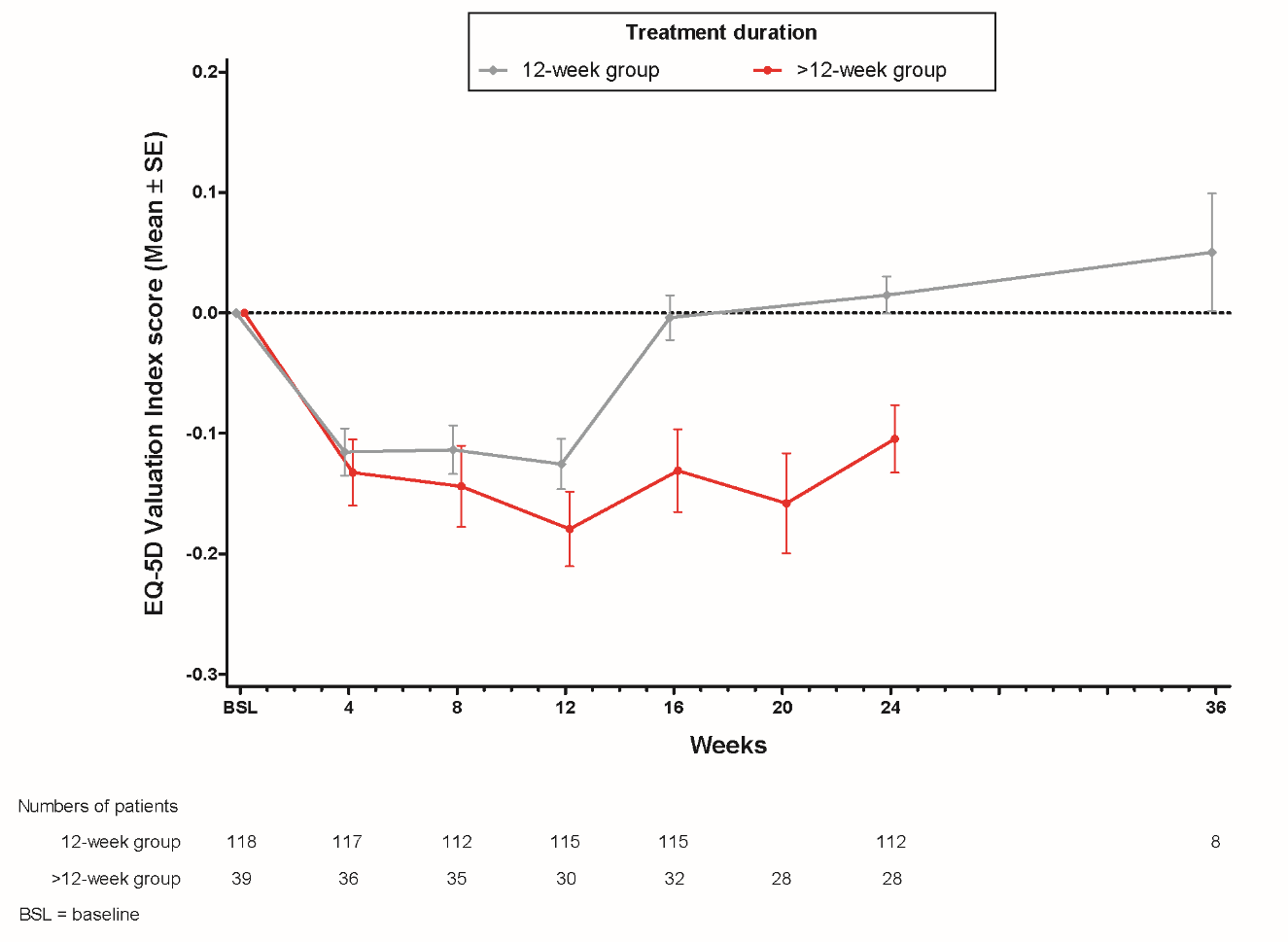
*

CES-D: center for epidemiologic studies depression scale; EQ-5D: EuroQol 5-dimensional scale; FSS: fatigue severity scale; PRO: patient-reported outcome; VAS: visual analog scale; WPAI: work productivity and activity impairment scale

**Figure 2**: Change from baseline in mean (±SE) PRO scores according to achievement or otherwise of SVR12, among patients receiving 12 weeks of treatment: **A** FSS score; **B** CES-D score; **C** WPAI daily activity impairment score **D** WPAI total work impairment score; **E** EQ-5D VAS score, **F** EQ-5D Valuation Index score

**A**

*
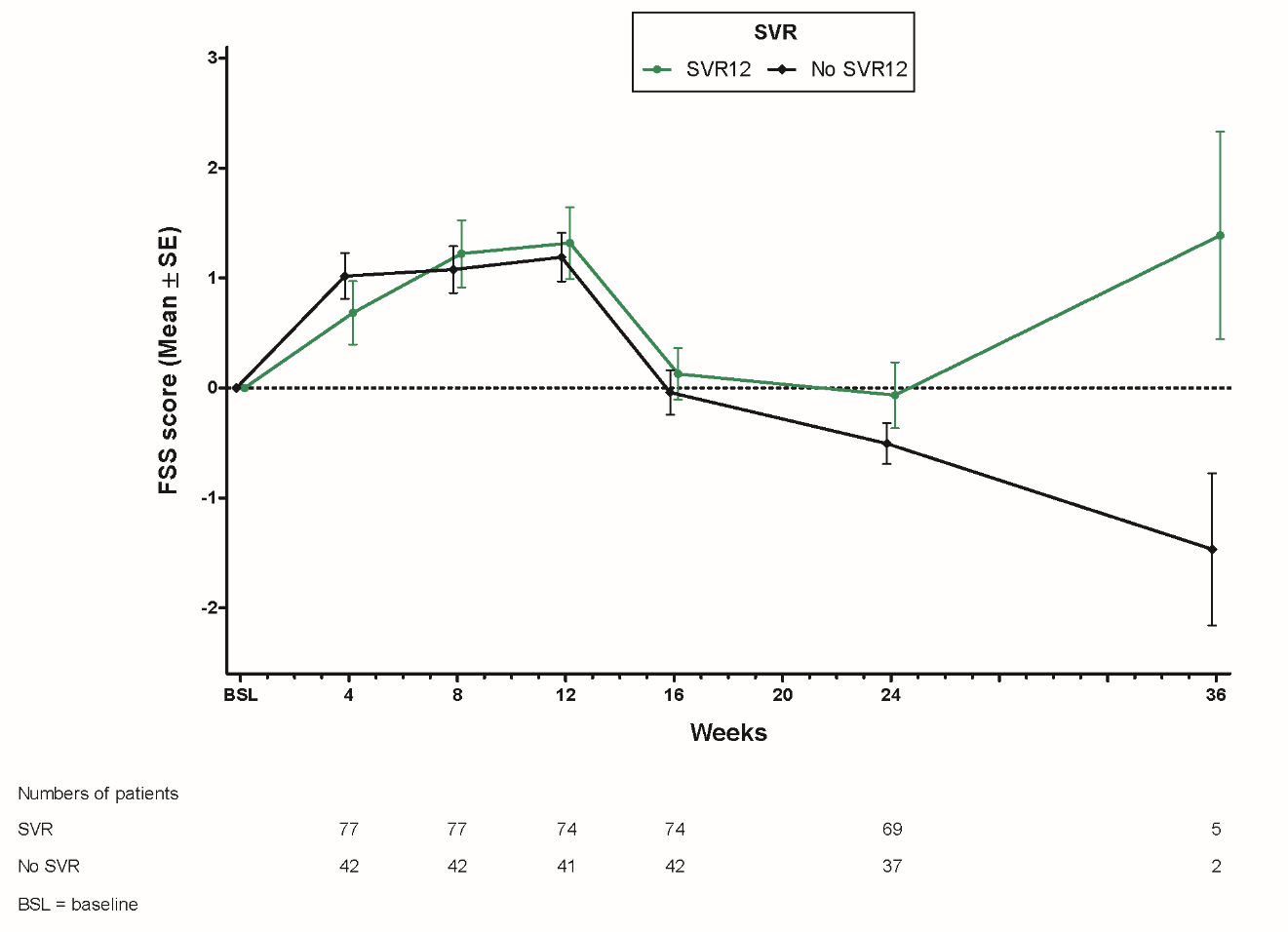
*

**B**

*
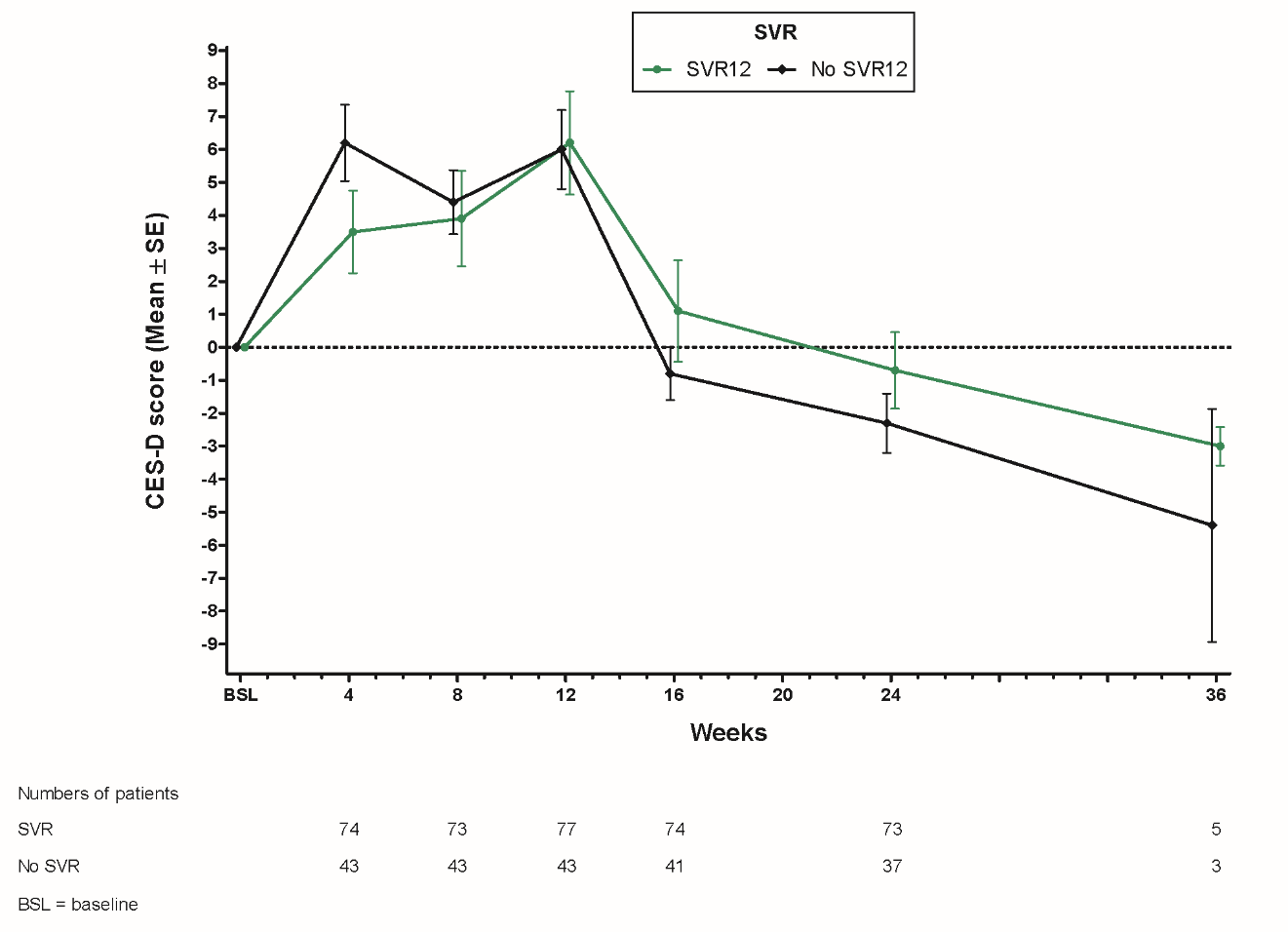
*

**C**

*
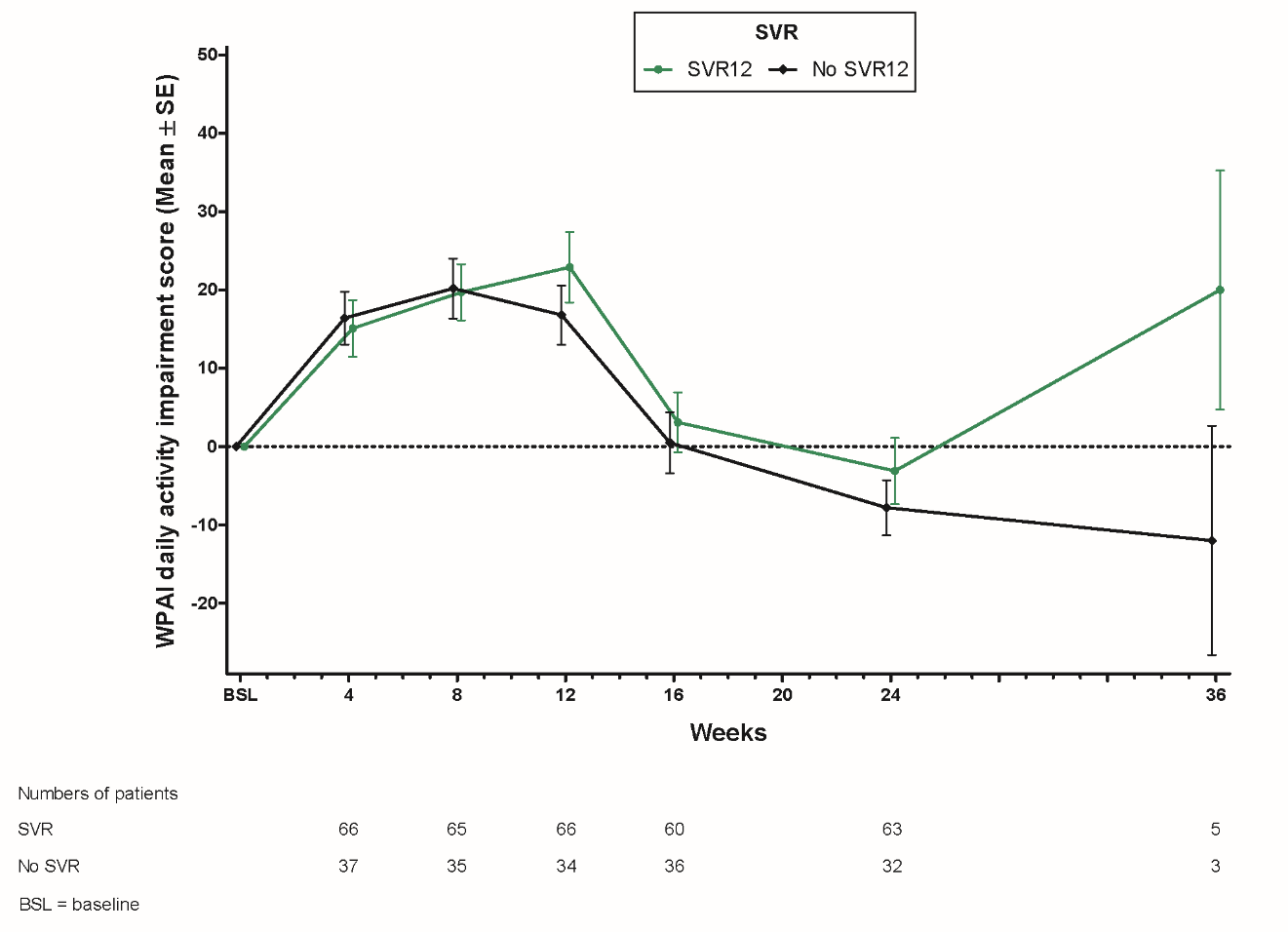
*

**D**

*
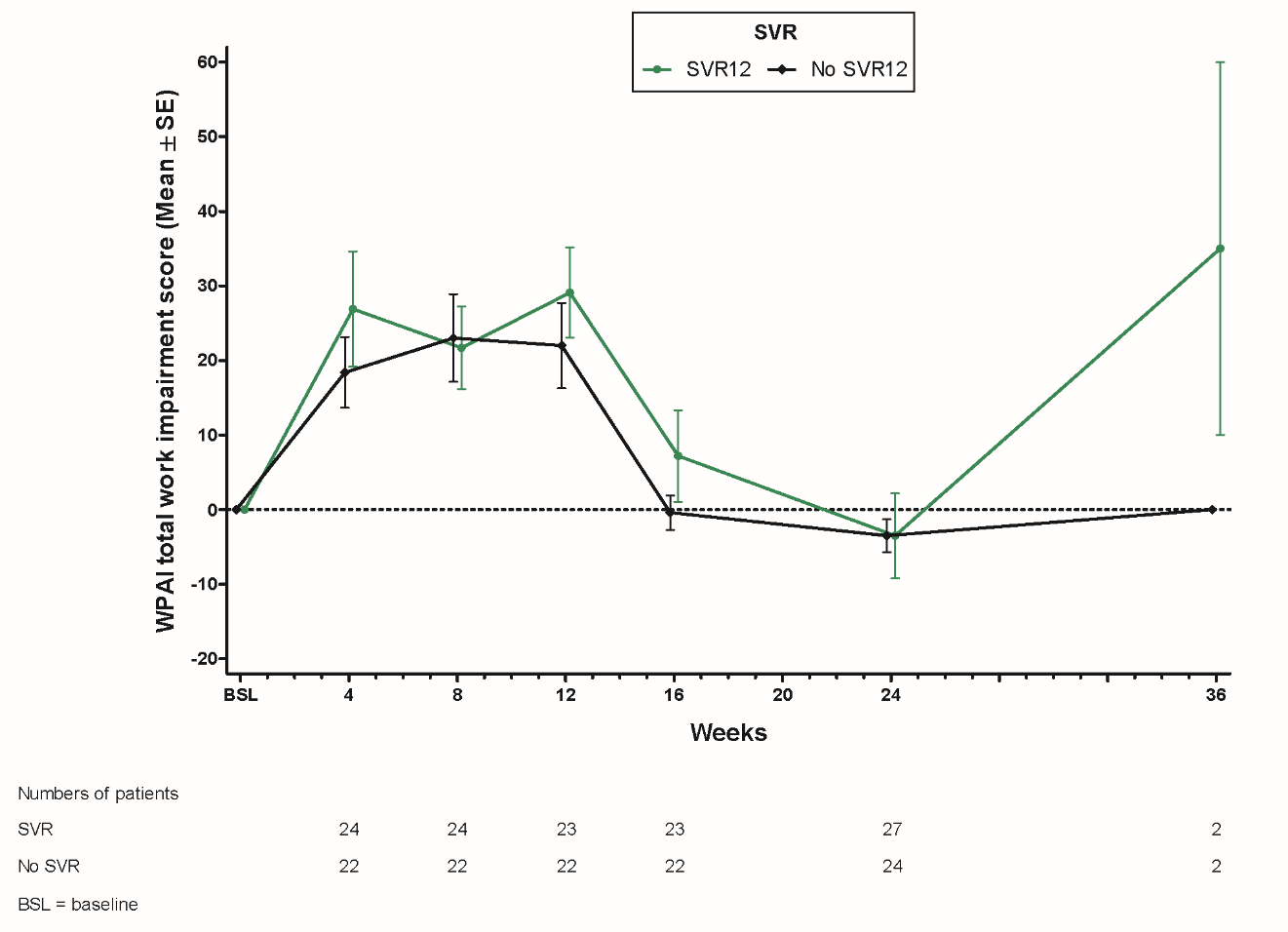
*

**E**

*
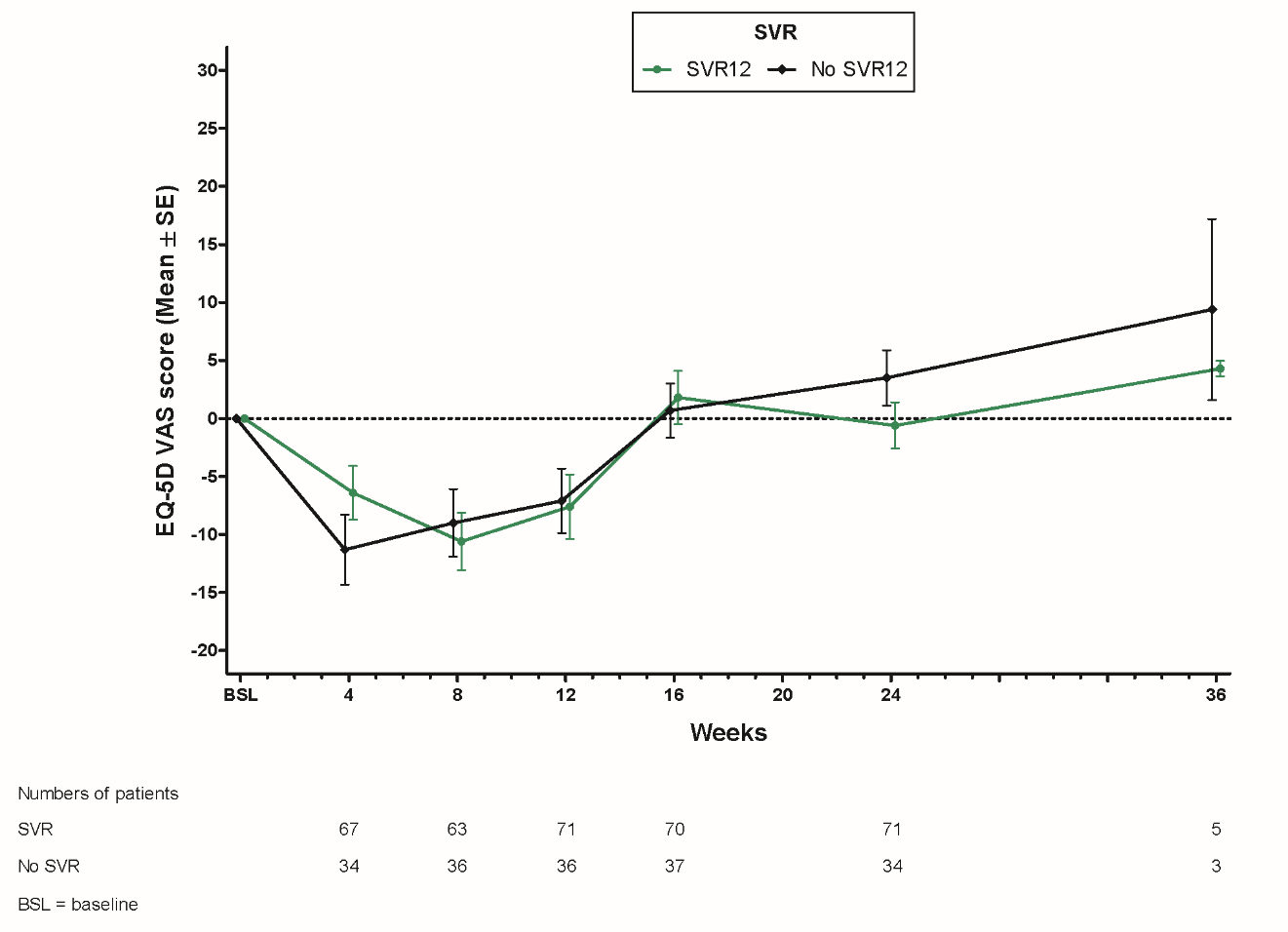
*

**F**

*
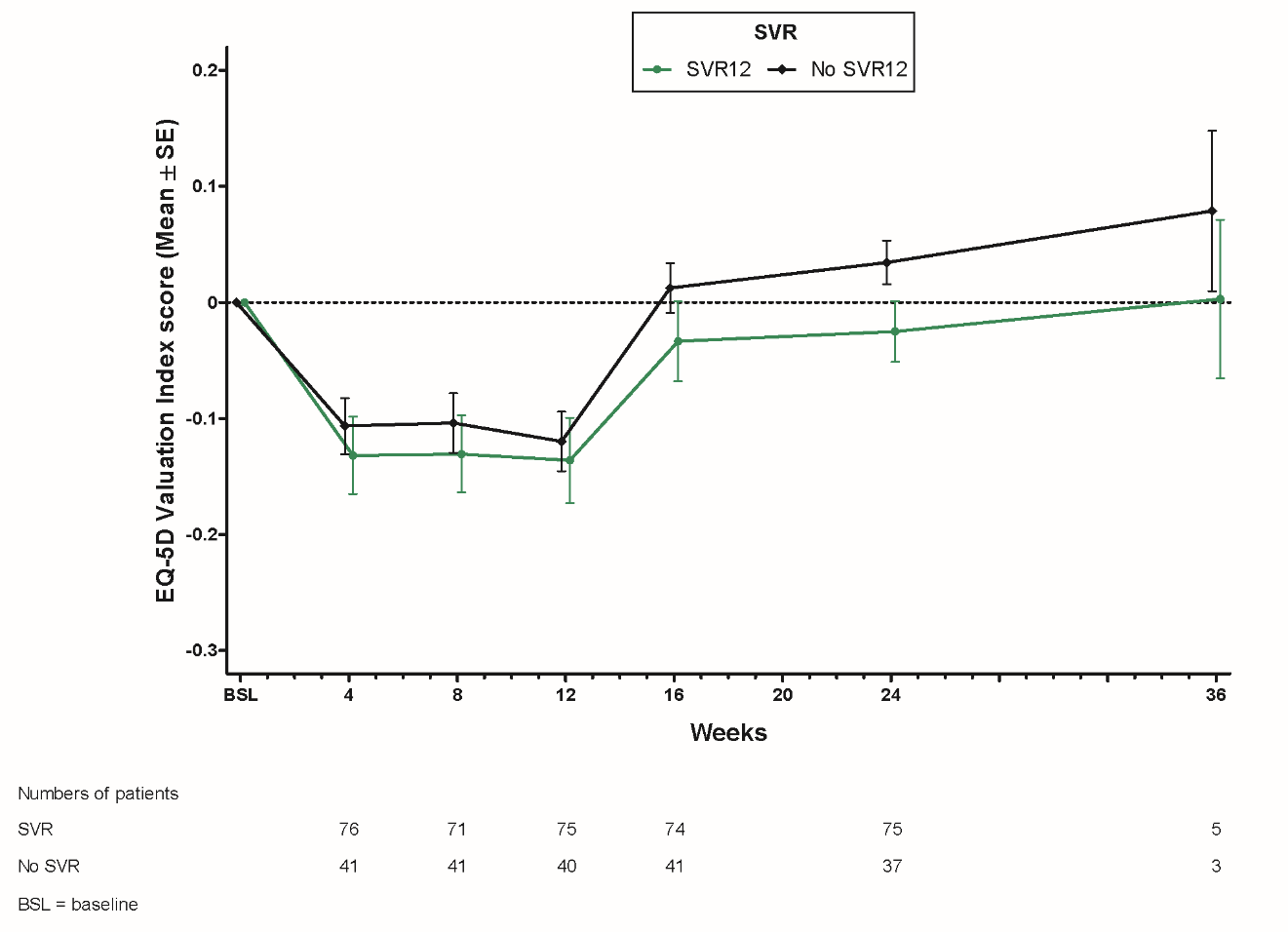
*

CES-D: center for epidemiologic studies depression scale; EQ-5D: EuroQol 5-dimensional scale; FSS: fatigue severity scale; PRO: patient-reported outcome; VAS: visual analog scale; WPAI: work productivity and activity impairment scale
