# Supplementary material for: An Open-Label Trial of 12-Week Simeprevir plus Peginterferon/Ribavirin (PR) in Treatment-Naïve Patients with Hepatitis C Virus (HCV) Genotype 1 (GT1)
Source: PLoS One. 2016 Jul 18;11(7):e0158526. doi: 10.1371/journal.pone.0158526 (PMC4948848; doi:10.1371/journal.pone.0158526)
Supplement: S1 Protocol — (PDF) [file pone.0158526.s008.pdf]

Janssen Therapeutics EMEA*

Clinical Protocol

A Phase 3, Open-Label Study to Evaluate the Safety and Efficacy of TMC435 plus Pegylated Interferon alfa-2a and Ribavirin Administered for 12 Weeks in Treatment-Naïve Subjects with Chronic Genotype 1 or Genotype 4 HCV Infection

Protocol TMC435HPC3014; Phase 3b
AMENDMENT INT-3
TMC435 (simeprevir)

* Janssen Therapeutics EMEA, a division of Janssen Pharmaceutica N.V. , is an organization that operates through different legal entities in various countries. Therefore, the legal entity acting as the sponsor for Janssen Therapeutics EMEA studies may vary, such as, but not limited to Janssen-Cilag International NV or Janssen Research and Development, LLC. The term “sponsor” is used throughout the protocol to represent these various legal entities; the sponsor is identified on the Contact Information page that accompanies the protocol.

EudraCT NUMBER: 2012-004905-29

Status: Approved
Date: 19 November 2013
Prepared by: Janssen Therapeutics EMEA
EDMS No. & version: EDMS-ERI-72508845 2.0

GCP Compliance: This study will be conducted in compliance with Good Clinical Practice, and applicable regulatory requirements.

Confidentiality Statement
The information in this document contains trade secrets and commercial information that are privileged or confidential and may not be disclosed unless such disclosure is required by applicable law or regulations. In any event, persons to whom the information is disclosed must be informed that the information is privileged or confidential and may not be further disclosed by them. These restrictions on disclosure will apply equally to all future information supplied to you that is indicated as privileged or confidential.

Protocol History[a]
TMC435HPC3014

Document Type and *File Name*	Issued Date	Amendment Type	Comments
Initial Clinical Trial Protocol *TMC435HPC3014-CTP-v1*	21-Mar-2013	-	-
Revised Clinical Trial Protocol *TMC435HPC3014-CTP-v2*	23-Apr-2013	-	Inclusion of new HCV-SIQ version
CTP Amendment I *TMC435HPC3014-CTPA-INT-I*	2- Sep-2013	Substantial	For details, please refer to Section Amendment INT-1
CTP Amendment II *TMC435HPC3014-CTPA-INT-2*	10-Oct-2013	Substantial	For details, please refer to Section Amendment INT-2
CTP Amendment III *TMC435HPC3014-CTPA-INT-3*	19-Nov-2013	Substantial	For details, please refer to Section Amendment INT-3

[a] This overview lists general amendments to the protocol only. Site and country specific amendments to the protocol are not included.

TABLE OF CONTENTS

TABLE OF CONTENTS 3
LIST OF ATTACHMENTS 6
LIST OF IN-TEXT TABLES AND FIGURES 6
TABLES 6
FIGURES 6
PROTOCOL AMENDMENTS 7
SYNOPSIS 12
TIME AND EVENTS SCHEDULE - PART 1: FOR ALL SUBJECTS (UP TO WEEK 12) 19
TIME AND EVENTS SCHEDULE - PART 2: FROM WEEK 12 TO WEEK 36 FOR SUBJECTS ELIGIBLE FOR A TOTAL TREATMENT DURATION OF 12 WEEKS 22
TIME AND EVENTS SCHEDULE - PART 3: FROM WEEK 12 TO WEEK 48 FOR SUBJECTS ELIGIBLE FOR A TOTAL TREATMENT DURATION OF 24 WEEKS (EXTENSION) 24
TIME AND EVENTS SCHEDULE - PART 4: IN CASE OF PREMATURE DISCONTINUATION OF ALL STUDY MEDICATION (TMC435/PEGIFNα-2A/RBV DURING THE FIRST 12 WEEKS OR PEGIFNα-2A/RBV AFTER WEEK 12) 26
ABBREVIATIONS 28
DEFINITION OF TERMS 30
1. INTRODUCTION 31
1.1. Background 31
1.1.1. Genotype 1 31
1.1.2. Genotype 4 32
1.2. Genotype 1 32
1.3. Genotype 4 36
1.4. Combination Therapy 37
1.5. Benefits and Risk Assessment 37
1.5.1. Known Benefits 37
1.5.2. Potential Benefits 38
1.5.3. Known Risks 38
1.5.4. Potential Risks 39
1.5.5. Overall Benefit/Risk Assessment 40
1.6. Overall Rationale for the Study 42
2. OBJECTIVES AND HYPOTHESIS 44
2.1. Objectives 44
2.2. Hypothesis 46
3. STUDY DESIGN AND RATIONALE 46
3.1. Overview of Study Design 46
3.2. Study Design Rationale 49
4. SUBJECT POPULATION 50
4.1. Inclusion Criteria 50
4.2. Exclusion Criteria 52
4.3. Prohibitions and Restrictions 55

5. TREATMENT ALLOCATION AND BLINDING ... 56

6. DOSAGE AND ADMINISTRATION ... 57
6.1. Treatment Overview and Response-Guided Treatment Duration ... 57
6.2. Timing of Dosing ... 58
6.3. Virologic Stopping Criteria ... 59

7. TREATMENT COMPLIANCE ... 60

8. PRESTUDY AND CONCOMITANT THERAPY ... 60

9. STUDY EVALUATIONS ... 63
9.1. Study Procedures ... 63
9.1.1. Overview ... 63
9.1.2. Screening Phase ... 64
9.1.3. Open-Label Treatment Phase ... 65
9.1.4. Posttreatment Phase (Follow-Up) ... 65
9.2. Efficacy ... 66
9.2.1. Evaluations ... 66
9.2.2. Endpoints ... 66
9.3. Resistance Determinations ... 67
9.4. Pharmacogenomic (DNA) Evaluations ... 68
9.5. Safety Evaluations ... 68
9.5.1. Adverse Events ... 68
9.5.2. Clinical Laboratory Tests ... 68
9.5.3. Electrocardiogram (ECG) ... 71
9.5.4. Vital Signs ... 71
9.5.5. Physical Examination ... 71
9.5.6. Patient-reported Symptoms, Functioning, and Quality of Life ... 71
9.5.6.1. Hepatitis C Symptom & Impact Questionnaire ... 72
9.5.6.2. Impact on Work and Daily Activities ... 72
9.5.6.3. Health Status and Quality of Life ... 73
9.5.6.4. Fatigue ... 73
9.5.6.5. Depressive Symptoms ... 73
9.5.7. Specific Toxicities ... 74
9.5.7.1. Rash ... 74
9.5.7.2. Acute Systemic Allergic Reaction ... 77
9.5.7.3. AST and ALT and/or Bilirubin Elevation ... 78
9.5.7.4. Clinical Hepatitis ... 80
9.5.7.5. Other Toxicities Considered to be at Least Possibly Related to TMC435 ... 81
9.5.7.6. Toxicity Management for Specific Adverse Events Considered to be at Least Possibly Related to PegIFNα-2a and Ribavirin ... 81
9.5.7.6.1. Neuropsychiatric Symptoms ... 82
9.5.7.6.2. Anemia ... 83
9.5.7.6.3. Hyperglycemia ... 83
9.5.7.6.4. Renal Complications ... 83
9.5.7.6.5. Decreased White Blood Cell Count ... 84
9.5.7.6.6. Decreased Platelet Count ... 84
9.5.7.6.7. Thyroid Abnormalities ... 84
9.6. Sample Collection and Handling ... 84

10. SUBJECT COMPLETION/WITHDRAWAL ... 85
10.1. Completion ... 85
10.2. Discontinuation of Study Treatment ... 85
10.3. Withdrawal From the Study ... 86

11. STATISTICAL METHODS ... 86
11.1. Subject Information ... 87
11.2. Sample Size Determination ... 87

11.3. Efficacy Analyses 88
11.4. Safety Analyses 88
11.5. Interim Analyses 89
11.6. Data Monitoring Committee 90

12. ADVERSE EVENT REPORTING 90
12.1. Definitions 90
12.1.1. Adverse Event Definitions and Classifications 90
12.1.2. Attribution Definitions 91
12.1.3. Severity Criteria 92
12.2. Special Reporting Situations 92
12.3. Procedures 92
12.3.1. All Adverse Events 92
12.3.2. Serious Adverse Events 93
12.3.3. Pregnancy 94
12.4. Contacting Sponsor Regarding Safety 95

13. PRODUCT QUALITY COMPLAINT HANDLING 95
13.1. Procedures 95
13.2. Contacting Sponsor Regarding Product Quality 95

14. STUDY DRUG INFORMATION 95
14.1. Physical Description of Study Drug(s) 95
14.2. Packaging 96
14.3. Labeling 96
14.4. Preparation, Handling, and Storage 96
14.5. Drug Accountability 97

15. STUDY-SPECIFIC MATERIALS 98

16. ETHICAL ASPECTS 98
16.1. Study-Specific Design Considerations 98
16.2. Regulatory Ethics Compliance 98
16.2.1. Investigator Responsibilities 98
16.2.2. Independent Ethics Committee or Institutional Review Board 99
16.2.3. Informed Consent 100
16.2.4. Privacy of Personal Data 101
16.2.5. Long-Term Retention of Samples for Additional Future Research 101
16.2.6. Country Selection 102

17. ADMINISTRATIVE REQUIREMENTS 102
17.1. Protocol Amendments 102
17.2. Regulatory Documentation 102
17.2.1. Regulatory Approval/Notification 102
17.2.2. Required Prestudy Documentation 102
17.3. Subject Identification, Enrollment, and Screening Logs 103
17.4. Source Documentation 103
17.5. Case Report Form Completion 104
17.6. Data Quality Assurance/Quality Control 105
17.7. Record Retention 105
17.8. Monitoring 106
17.9. Study Completion/Termination 106
17.9.1. Study Completion 106
17.9.2. Study Termination 106
17.10. On-Site Audits 107
17.11. Use of Information and Publication 107

REFERENCES 109

ATTACHMENTS **112**

INVESTIGATOR AGREEMENT **128**

LAST PAGE **128**

LIST OF ATTACHMENTS

Attachment 1: Metavir Scoring System, 1997 112
Attachment 2: WHO Toxicity Grading Scale for Determining the Severity of Adverse Events, February 2003 113
Attachment 3: Hepatitis C Symptom & Impact Questionnaire (HCV-SIQ v3) 118
Attachment 4: Work Productivity and Activity Impairment:Hepatitis C Questionnaire (WPAI:Hepatitis C) 120
Attachment 5: EuroQol 5-Dimension Questionnaire (EQ-5D) 122
Attachment 6: Fatigue Severity Scale (FSS) 125
Attachment 7: Center For Epidemiologic Studies-Depression Scale (CES-D) 126
Attachment 8: Visit Schedule for Rash Management 127

LIST OF IN-TEXT TABLES AND FIGURES

TABLES

Table 1: Treatment Overview 58
Table 2: Guidelines for Subjects Developing Allergic Reactions 78
Table 3: Treatment-emergent Grade 1 to Grade 3 ALT or AST elevation or Grade 1 to Grade 3 total bilirubin elevation 78
Table 4: Treatment-emergent Grade 4 ALT or AST elevation or Grade 4 total bilirubin elevation 79
Table 5: Guidelines for Subjects Developing ALT/AST Elevations 80
Table 6: Guidelines for Subjects Developing Bilirubin Elevations 80
Table 7: Guidelines for Subjects Developing Psychiatric Symptoms/Depression 82
Table 8: Guidelines for Subjects Developing Anemia 83
Table 9: Guidelines for Subjects Developing Decreased White Blood Cell Count 84
Table 10: Guidelines for Subjects Developing Decreased Platelet Count 84

FIGURES

Figure 1: Schematic Overview of the Study 48

PROTOCOL AMENDMENTS

Protocol Version	Issue Date
Original Protocol	23 Apr 2013
Amendment INT-1	2 Sep 2013
Amendment INT-2	10 Oct 2013
Amendment INT-3	19 Nov 2013

Amendments are listed beginning with the most recent amendment.

Amendment INT-3 (19 Nov 2013)

This amendment is considered to be substantial based on the criteria set forth in Article 10(a) of Directive 2001/20/EC of the European Parliament and the Council of the European Union.

The overall reason for the amendment: This amendment was created to include subjects with genotype 4 HCV infection in the trial population.

Applicable Section(s)	Description of Change(s)
**Rationale:** HCV genotype 4 and genotype 1 show similar response rates to treatment with interferon and ribavirin. Thus, international treatment guidelines recommend identical treatment algorithms for genotype 1 and 4 HCV. In both genotypes rapid viral response to PegIFN/RBV has been shown to be associated with higher SVR rates and allows shortening of treatment duration to 24 weeks. A recent study also demonstrated high SVR rates in genotype 4 HCV infection (96%) after 12 weeks of triple therapy with the polymerase inhibitor sofosbuvir in combination with PegIFN/RBV. TMC435 showed similar antiviral activity in genotype 1 and 4 in a monotherapy study. An interim analysis from an ongoing open-label phase 3 clinical trial with TMC435 (150 mg qd administered for 12 weeks) in combination with PegIFN/RBV in genotype 4 showed that > 80% of treatment-naïve subjects and prior relapsers were able to shorten treatment to 24 weeks based on response-guided treatment criteria. Moreover, preliminary SVR12 rates in genotype 4 from this ongoing phase 3 study are very similar to those from the phase 3 studies in genotype 1. This suggests that patients with genotype 4 HCV infection who have very rapid viral response could benefit from a 12-week treatment duration with TMC435 in combination with PegIFN/RBV.	
Throughout the protocol	Added a second arm for subjects with genotype-4 HCV infection
**Rationale:** Correction of an error: insulin and glucose need to be tested in plasma samples (not serum samples) for the HOMA IR calculations. This was specified correctly in the laboratory manual but not in the protocol. Central laboratory tests for insulin and glucose have been performed on plasma samples to date.	
9.5.2. Clinical Laboratory Tests	Removed the specification 'serum' for the panel of chemistry tests. Correct information on which samples need to be taken is available in the laboratory manual.
**Rationale:** Correction of an error: crossreference was made to the wrong footnote	
Time and Events Schedule Part 3 and Part 4	Inserted reference to correct footnote for viral sequencing

Applicable Section(s)	Description of Change(s)
**Rationale:** Correction of an inconsistency: statement " If TMC435 is discontinued for the management of an AE or because a subject meets a virologic stopping rule, it must not be restarted." was not consistent with text allowing temporary interruptions of TMC435 for tolerability reasons	
6.2 Timing of Dosing, 9.5.7.6.2 Anemia	deleted inconsistent text

<u>Amendment INT-2</u> (10 Oct 2013)

This amendment is considered to be substantial based on the criteria set forth in Article 10(a) of Directive 2001/20/EC of the European Parliament and the Council of the European Union.

The overall reason for the amendment: The Sponsor amends the protocol to introduce precautionary language on photosensitivity. The changes made to the clinical protocol TMC435HPC3014-CTPA INT-1, dd. 2 Sep 2013, are listed below.

Applicable Section(s)	Description of Change(s)
**Rationale:** The Phase 1 photosensitivity study C125 concluded that the photosensitizing potential of TMC435 is similar to that of placebo. Accordingly, formal recommendations for sun-protective measures were removed from the ongoing TMC435 studies at the time when these results became available, and were not included in future TMC435 study protocols. After analysis of the Phase 3 (C208/C216/HPC3007) studies, in which subjects were dosed with TMC435 prior to removal of recommendation for sun-protective measures, photosensitivity conditions were nevertheless identified as adverse drug reaction of TMC435 as detailed in the updated Investigator Brochure Edition 7, issued in June 2013. As a follow up to a recommendation by US FDA, it was decided to reintroduce sun-protective language in TMC435 studies in which subjects are being dosed with TMC435 and in future TMC435 studies.	
4.3 Prohibitions and restrictions	Added wording to inform subjects of possible photosensitivity reactions when taking TMC435 and introduce restrictions regarding exposure to sun during TMC435 treatment

Amendment INT-1 (2 Sep 2013)

This amendment is considered to be substantial based on the criteria set forth in Article 10(a) of Directive 2001/20/EC of the European Parliament and the Council of the European Union.

The overall reason for the amendment: The overall reasons for the amendment are to implement comments received from ethics committees (EC) and HA and to update the benefit-risk assessment with Phase 3 data that have become available.

Applicable Section(s)	Description of Change(s)
**Rationale:** The hypothesis test (which stated no hypothesis testing would occur) was made consistent with the primary efficacy analysis (which planned to compare response to a predefined minimally acceptable response rate).	
Synopsis Hypothesis; 2.2 Hypothesis	Hypothesis formulation added to ensure consistency of hypothesis wording with the planned primary efficacy analysis.
Synopsis Statistical Methods; 11.3 Efficacy Analyses	Text added to specify that the primary parameter will be assessed using a one-sided test with alpha=0.05 for consistency with the calculated power specified in the sample size determination.
Synopsis Statistical Methods; 11.3 Efficacy Analyses	Moved text on 2-sided confidence intervals to make it clear this is applicable for secondary efficacy parameters
**Rationale:** A Data Monitoring Committee (DMC) will be installed for this study at the request of EC and HA.	
11.6 DMC	Added new section to specify set up of a DMC
**Rationale:** At the request of EC, the exclusion criteria were adjusted so that imprisoned subjects cannot be enrolled.	
4.2 Exclusion criteria	Updated exclusion criterion 15 to exclude vulnerable subjects
**Rationale:** The introduction and benefit-risk assessment have been updated with Phase 3 data that have become available.	
1. Introduction; 1.1 Background; 1.3 Benefits and risk assessment	Revision to include Phase 3 data that have become available.
Throughout protocol	Included reference to most recent Investigator's Brochure (IB), which was updated to include Phase 3 data
Throughout protocol	Removed reference to previous edition of IB
References	Deleted references that are no longer used after the update

Applicable Section(s)	Description of Change(s)
**Rationale:** In view of the capping for IL28B genotypes, a procedure will be put in place so that subjects who have an IL28B genotype for which maximum enrollment has been reached do not need to undergo all screening assessments.	
Time and Events Schedule; 9.1.2 Screening	Specified that IL28B screening can be done first and further screening assessments can be undertaken after the results of IL28B screening have been received.
**Rationale:** Adverse events will be reported until 4 weeks after last study medication intake (previously until last study related activity) to align AE reporting across Sponsor's studies. Thereafter, only adverse events considered related to TMC435 will be reported. SAEs will be reported until 30 days after last study-related procedure.	
Synopsis Safety; Time and Events Schedule; 9.1.3 Treatment phase; 12.3.1 Adverse events	Adverse events will be reported until 4 weeks after last study medication intake (previously until last study related activity). Thereafter, only adverse events considered related to TMC435 will be reported.
12.3.1 Adverse events	Serious AEs will be reported until 30 days after last study related activity
**Rationale:** Requirement to do a home urine pregnancy test when visits are more than 4 weeks apart.	
Time and Events Schedule; 9.1.3, 9.5.2 Pregnancy test	Added to footnote: extra pregnancy test kits will be given if more than 4 weeks between two consecutive study visits to perform a pregnancy test at home.
**Rationale:** Clarification of allowed and disallowed systemic corticosteroid use: dexamethasone is a CYP3A4 inducer and should not be used; therapeutic index of prednisone and methylprednisone is not too narrow to allow their use	
8. Concomitant therapy	Specified that dexamethasone should not be used, not even for < 24 h in case of rash
8. Concomitant therapy	Deleted prednisone and methylprednisone as their use can be allowed
**Rationale:** Clarification of treatment guidelines in case of specific PegIFN-related AEs to make it clear that RBV monotherapy is not allowed.	
9.5.7.6.1 Neuropsychiatric Symptoms; 9.5.7.6.5 Decreased WBC 9.5.7.6.5 Decreased platelets	Stated explicitly that if PegIFNα-2a has to be discontinued for management of the AE, then TMC435 and RBV must also be discontinued

Applicable Section(s)	Description of Change(s)
**Rationale:** Correction of an error: both male and female subjects must discontinue study medication if they fail to comply with contraception requirements	
1.3.5 Overall benefit/risk assessment	Deletion of erroneous text
**Rationale:** Correction of an error: SVR12 time point for subjects receiving treatment for 24 weeks is Week 36 (instead of Week 24)	
Figure 1	Moved SVR12 time point to correct location on study timeline
**Rationale:** Correction of an error: creatinine is not part of a total blood cell count	
9.5.2 Laboratory tests	Moved creatinine out of brackets clarifying total blood cell count
**Rationale:** Correction of an inconsistency: if telephone contact is not allowed for Grade 1 rash, it should not be allowed for more severe Grade 2 rash	
9.5.7.1 Rash	Removed the option of replacing an unscheduled visit with a telephone contact if a visit cannot be scheduled
**Rationale:** To Include reference to new edition of the Diagnostic and Statistical Manual of Mental Disorders	
9.5.7.6.1 Neuropsychiatric Symptoms	Updated reference to DSM-V
**Rationale:** To specify in the protocol the time points of the interim analyses for the DMC review	
Synopsis<br>11.5 Interim Analyses	An extra interim analysis at an earlier time point was added
**Rationale:** To include safety criteria for discontinuation	
17.9.2 Study termination	Added safety criteria for discontinuation
**Rationale:** Clarified that study will be included in yearly DSUR until availability of final CSR (which is a more specific description than 'study closure')	
17.11 Information	specified that study will be included in yearly DSUR until availability of final CSR (previously until 'study closure')

SYNOPSIS

A Phase 3, Open-Label Study to Evaluate the Safety and Efficacy of TMC435 plus Pegylated Interferon alfa-2a and Ribavirin Administered for 12 Weeks in Treatment-Naïve Subjects with Chronic Genotype 1 or Genotype 4 HCV Infection

EUDRACT number: 2012-004905-29

TMC435 (simeprevir), formerly known as TMC435350, is an NS3/4A protease inhibitor (PI) in development for treatment of chronic hepatitis C virus (HCV) infection (clinical development stage: Phase 3).

OBJECTIVES AND HYPOTHESIS

Primary Objectives

The primary objectives are:

- To determine the efficacy of TMC435 plus pegylated interferon alfa-2a (PegIFNα-2a) and ribavirin (RBV) when administered for 12 weeks in treatment-naïve subjects with chronic genotype 1 HCV infection, as measured by the proportion of subjects with sustained virologic response (SVR) 12 weeks after planned end of treatment (SVR12).
- To assess the safety and tolerability of TMC435 plus PegIFNα-2a and RBV when administered for 12 weeks in treatment-naïve subjects with chronic genotype 1 HCV infection

Major Secondary Objectives

Major secondary objectives are:

- To determine the efficacy of TMC435 plus PegIFNα-2a and RBV when administered for 12 weeks in treatment-naïve subjects with chronic genotype 4 HCV infection, as measured by the proportion of subjects with SVR12
- To assess the safety and tolerability of TMC435 plus PegIFNα-2a and RBV when administered for 12 weeks in treatment-naïve subjects with chronic genotype 4 HCV infection

Further major secondary objectives are in subjects with genotype 1 or genotype 4 HCV infection (separately per genotype):

- To determine the proportion of subjects who achieve virologic response at Week 2 (W2VR) and the proportion of subjects who achieve rapid virologic response (RVR).
- To determine the relationship between W2VR and SVR12 and between RVR and SVR12.
- To determine the efficacy of TMC435 plus PegIFNα-2a and RBV for 12 weeks followed by 12 weeks of PegIFNα-2a and RBV (i.e., a total treatment duration of 24 weeks), as measured by the proportion of subjects with SVR12.
- To determine the efficacy of TMC435 plus PegIFNα-2a and RBV after a total treatment duration of 12 weeks, as measured by the proportion of subjects with sustained virologic response 24 weeks after planned end of treatment (SVR24).
- To determine the efficacy of TMC435 plus PegIFNα-2a and RBV for 12 weeks followed by 12 weeks of PegIFNα-2a and RBV (i.e., a total treatment duration of 24 weeks), as measured by the proportion of subjects with SVR24.
- To evaluate the evolution of HCV RNA levels at regular intervals during treatment and after planned end of treatment.

and for both genotypes combined:

- To evaluate impact of HCV or its treatment on patient reported symptoms and functioning using a new patient reported outcomes tool, the Hepatitis C Symptom & Impact Questionnaire (HCV-SIQ) and four well-validated PRO instruments measuring severity and impact of fatigue (Fatigue Severity Scale, FSS), depressive symptoms (Center for Epidemiologic Studies Depression Scale, CES-D), time missed from work and impairment in daily activities (Work Productivity and Activity Impairment, WPAI: Hepatitis C), and health status (EuroQol 5 Dimension, EQ5D).

Definitions

Sustained virologic response 12 weeks after planned end of treatment (SVR12) will be defined as undetectable HCV RNA (< 25 IU/mL undetectable) at the actual end of treatment and 12 weeks after the planned end of treatment.

Rapid virologic response (RVR) will be defined as HCV RNA < 25 IU/mL undetectable measured 4 weeks after start of treatment.

Virologic response at Week 2 (W2VR) will be defined as HCV RNA < 25 IU/mL (detectable or undetectable) measured 2 weeks after start of treatment.

Exploratory Objectives

The exploratory objectives in subjects with genotype 1 or genotype 4 HCV infection are, for both genotypes combined:

- to evaluate the measurement properties of the HCV-SIQ
- to explore alternate scoring of the HCV-SIQ (e.g., total score, symptom scores by body system).

Hypothesis

In this study, the response rate (SVR12) in subjects with genotype 1 HCV infection eligible for and assigned to 12 weeks of treatment is expected to be superior to the minimally acceptable response rate of 80%.

OVERVIEW OF STUDY DESIGN

Shortening treatment duration in HCV infected patients is one of the key objectives for patients on triple therapy (direct-acting anti-viral agent [DAA] plus PegIFN/RBV). It may decrease side effects associated with longer medication use and could mean better medication adherence as well. The sponsor aims to investigate the possibility to shorten the treatment duration to 12 weeks triple therapy (PegIFNα-2a/RBV/TMC435) in treatment-naïve subjects with chronic genotype 1 or genotype 4 HCV infection.

This is a multicenter, international study to evaluate the efficacy, tolerability, and safety of 12-week triple therapy with TMC435 plus PegIFNα-2a and RBV in treatment-naïve adult subjects with genotype 1 or genotype 4 chronic HCV infection and fibrosis stage equivalent to Metavir F0-F2. *IL28B* genotyping is mandatory for participating subjects and will be included in the main Informed Consent Form.

In an attempt to enroll similar percentages of the 3 host *IL28B* genotypes (CC, CT or TT) as were enrolled in the ongoing Phase 3 TMC435 studies, the sponsor will make all effort to respect the following maximum caps for enrolment of each *IL28B* genotype for subjects with genotype 1 or genotype 4 HCV infection separately:

- a maximum of 35% of subjects with host *IL28B* genotype CC (i.e., maximum 35% *IL28B* CC among subjects with genotype 1 HCV infection and maximum 35% *IL28B* CC among subjects with genotype 4 HCV infection);

- a maximum of 55% of subjects with host *IL28B* genotype CT (i.e., maximum 55% *IL28B* CT among subjects with genotype 1 HCV infection and maximum 55% *IL28B* CT among subjects with genotype 4 HCV infection);
- a maximum of 18% of subjects with host *IL28B* genotype TT (i.e., maximum 18% *IL28B* TT among subjects with genotype 1 HCV infection and maximum 18% *IL28B* TT among subjects with genotype 4 HCV infection).

In addition, within subjects infected with genotype 1 HCV, the sponsor seeks a balance between HCV genotypes 1a and 1b, therefore all effort will be made to cap enrolment of HCV genotype 1a at:

- a maximum of 50% of subjects infected with HCV genotype 1a.

Procedures to manage the distribution of the HCV genotype 1 subtypes and the *IL28B* subtypes will be communicated to participating investigators at study initiation.

All subjects will start treatment at baseline (Day 1) and will receive triple therapy consisting of TMC435 plus PegIFNα-2a and RBV for 12 weeks. Total anti-HCV treatment duration will be response-guided based on HCV RNA levels at Week 2, Week 4, and Week 8 as follows:

- Subjects will qualify for a total treatment duration of only 12 weeks (ie all anti-HCV treatment is discontinued after week 12) if the following criteria are met: if HCV RNA value is
    - o < 25 IU/mL (detectable or undetectable) at Week 2,

    AND
    - o < 25 IU/mL undetectable at Week 4,

    AND
    - o < 25 IU/mL undetectable at Week 8.
- if HCV RNA is above the threshold defined above at any of the 3 time points, subjects will stop TMC435 treatment at Week 12 and will continue PegIFNα-2a and RBV until Week 24.

The above treatment continuation criteria will at all times be overruled if a subject meets any of the virologic stopping criteria. Subjects meeting any of the following criteria will be required to discontinue all treatment:

- Week 4: HCV RNA ≥ 25 IU/mL
- Week 12: HCV RNA ≥ 25 IU/mL OR < 25 IU/mL detectable
- a confirmed increase of > 1 $\log_{10}$ IU/mL in HCV RNA level from the lowest level reached, or a confirmed HCV RNA level of > 100 IU/mL in subjects whose HCV RNA level had previously been < 25 IU/mL while on study drugs.

The study will be conducted in 3 phases: a screening phase of maximum 6 weeks, a treatment phase extending from Day 1 (baseline) up to 12 or 24 weeks depending on the response to treatment, and a posttreatment follow-up period of 24 weeks after the subject's last planned dose of study drug. The duration of the subject's participation (excluding screening phase) will vary between 36 weeks and 48 weeks, depending on the response to treatment. The study will be considered completed with the last visit of the last subject.

SUBJECT POPULATION

A target of 150 subjects infected with genotype 1 HCV and 75 subjects infected with genotype 4 HCV will be assigned to treatment in this study.

Main selection criteria

Males or females aged between 18 and 70 years (extremes included); treatment-naïve with confirmed chronic HCV infection; liver biopsy performed within 2 years prior to screening or non-invasive confirmation of the liver disease stage (by transient elastography) performed within 6 months prior to screening; liver disease stage equivalent to Metavir Score F0-F2 (no fibrosis, or portal fibrosis without or with few septa); genotype 1 or genotype 4 HCV infection (confirmed at screening). Subjects with liver disease stage equivalent to Metavir Score F2 and without liver biopsy performed within 2 years prior to screening must have undergone liver imaging within 6 months prior to the Screening visit (or between the Screening and Baseline/Day 1 visits) with no findings suspicious of hepatocellular carcinoma.

Subjects with advanced liver disease equivalent to Metavir score F3-F4 (bridging fibrosis or cirrhosis), with hepatic decompensation, with any liver disease of non-HCV etiology, and/or with a non-genotype 1 or non-genotype 4 hepatitis C, hepatitis B or HIV co-infection will be excluded. Subjects willing to participate, having signed the informed consent form (ICF), and found eligible for the study, may be required to discontinue if disallowed concomitant medication needs to be started.

DOSAGE AND ADMINISTRATION

TMC435 will be taken as oral capsules at a once daily (q.d.) dose of 150 mg with food.

PegIFNα-2a and RBV will be given as Pegasys® and Copegus®, respectively. Pegasys and Copegus will be administered according to the manufacturer's prescribing information. Pegasys (180 µg once weekly) will be administered as weekly subcutaneous (SC) injections of 0.5 mL. If the subject's baseline body weight is < 75 kg, the total daily dose of Copegus will be 1000 mg, administered as 400 mg (2 oral tablets of 200 mg, intake with food) in the morning and 600 mg (3 oral tablets of 200 mg, intake with food) in the evening. If the baseline body weight is ≥ 75 kg, the total daily dose will be 1200 mg, administered as 600 mg in the morning and evening (3 oral tablets of 200 mg per intake, with food).

EFFICACY EVALUATIONS

Samples for the determination of HCV RNA levels will be taken at predefined time points and processed in real-time (see the Time and Events Schedule that follows the Synopsis).

RESISTANCE DETERMINATIONS

Samples for sequencing of HCV NS3/4A will be collected at predefined time points (see the Time and Events Schedule that follows the Synopsis). Sequencing of the baseline sample will be done in real-time. Sequencing of samples taken after baseline will occur at the discretion of the Study Responsible Scientist.

PHARMACOGENOMIC (DNA) EVALUATIONS

A pharmacogenomic blood sample will be collected to allow for host *IL28B* genotyping. This pharmacogenomic blood sample is mandatory and will be collected from all subjects who consent to participate in the study.

SAFETY EVALUATIONS

Safety and tolerability will be evaluated throughout the study from signing of the informed consent onwards until the last study-related visit. Adverse events will be reported from signing of ICF until 4 weeks after last intake of study medication. Thereafter, only adverse events considered related to TMC435 will be reported. Serious adverse events will be reported until 30 days after last study-related procedure. The evaluations of safety and tolerability will include monitoring of adverse events (AEs), clinical laboratory tests, vital signs, physical examination, hepatitis C symptoms and impact (HCV-SIQ), impact on work and daily activities (WPAI:Hepatitis C), health status and quality of life (EQ-5D), fatigue (FSS), and depressive symptoms (CES-D) according to the time points indicated in the Time and Events

Schedule that follows the Synopsis. Specific toxicity management plans are incorporated in line with known toxicities for the medicinal products evaluated in this study.

STATISTICAL METHODS

The primary analysis will be performed when all subjects infected with genotype 1 HCV who were eligible for and assigned to a total treatment duration of 12 weeks have completed the Week 24 visit (SVR12) or discontinued earlier.

The final analysis will be performed when all subjects have completed the last study-related visit (Week 36 or Week 48 depending on total treatment duration) or discontinued earlier.

Interim analyses will be done for subjects infected with genotype 1 HCV:

- when the first 35 subjects that were eligible for a total treatment duration of 12 weeks and completed their treatment have reached the time point of SVR4 (4 weeks after planned end of treatment, corresponding to Week 16).
- when all subjects eligible for a total treatment duration of 12 weeks have completed the last study-related visit (Week 36) or discontinued earlier.

An additional interim analysis may be done when all subjects infected with genotype 1 HCV who were eligible for and assigned to a total treatment duration of 12 weeks have reached the time point of SVR4 or have discontinued earlier.

An interim analysis will be done for subjects infected with genotype 4 HCV:

- when all subjects eligible for and assigned to a total treatment duration of 12 weeks have completed the Week 24 visit (SVR12) or discontinued earlier.

An additional interim analysis may be conducted when the first 20 subjects infected with genotype 4 HCV who were eligible for a total treatment duration of 12 weeks and completed their treatment have reached the time point of SVR4.

Given the timing of the above-listed interim analyses, if 2 of the planned analyses are close in meeting their cut-off point, one may be slightly delayed to allow them to be done concurrently.

Sample Size Determination

The primary efficacy parameter is SVR12 for subjects infected with genotype 1 HCV who were eligible for and assigned to a total treatment duration of 12 weeks. One hundred and fifty subjects infected with genotype 1 HCV and with fibrosis stages ranging from equivalent to F0 to equivalent to F2 will be treated. With the assumption 50% will have genotype 1a HCV infection and 50% genotype 1b, and with estimated *IL28B* genotype breakdown being 30% CC, 55% CT and 15% TT, it is expected 69% (104 subjects) will have a Week 2 response and reach RVR and be eligible for a total treatment duration of 12 weeks.

The minimally acceptable response rate (SVR12 in subjects eligible for and assigned to 12 weeks of treatment) is taken as 80%, whilst the target response rate is 90%. With an alpha=0.05, and a sample size of 104, based on these assumptions, the calculated power would be 90.6%. As the N is assumed, several scenarios are presented below:

N	Target Response	Power
96	90%	83.9%
104	90%	90.6%
112	90%	90.8%

In addition, 75 subjects with genotype 4 HCV infection and with fibrosis stages ranging from equivalent to F0 to equivalent to F2 will be treated. With the assumption the estimated *IL28B* genotype breakdown will be 30% CC, 55% CT and 15% TT, it is expected 69% (52 subjects) will have a Week 2 response and reach RVR and be eligible for a total treatment duration of 12 weeks. With a target response rate of 90%, 52 subjects will allow the response rate to be estimated with a 2-sided 95% CI width of 18.1%: 90% (78.5%, 96.6%).

Efficacy

The primary efficacy endpoint is the proportion of subjects infected with genotype 1 HCV with SVR12, i.e., subjects with undetectable HCV RNA (< 25 IU/mL undetectable) at the actual end of treatment and 12 weeks after the planned end of treatment, in subjects infected with genotype 1 HCV who were eligible for and assigned to a total treatment duration of 12 weeks.

For the primary endpoint, SVR12 in subjects infected with genotype 1 HCV who were eligible for and assigned to 12 weeks of treatment, a one-sided test, with alpha=0.05 will be used to compare the response rate to the minimally acceptable response rate of 80%.

Secondary efficacy parameters will be analyzed as described below. Two-sided 95% confidence intervals will be constructed around the observed response rate.

Secondary endponts are:

In subjects infected with genotype 4 HCV who were eligible for and assigned to a total treatment duration of 12 weeks:

- the proportion of subjects with SVR12

For all subjects per assigned total treatment duration and per HCV genotype (separately):

- the proportion of subjects who achieve RVR;
- the proportion of subjects who achieve virologic response at Week 2 (W2VR);
- the proportion of subjects with SVR12;
- the proportion of subjects with SVR24;
- the proportion of subjects with ≥ 2 log decrease in HCV RNA at each time point;
- the proportion of subjects with HCV RNA < 25 IU/mL undetectable at each time point;
- the proportion of subjects with viral breakthrough;
- the proportion of subjects with viral relapse;
- the proportion of subjects with normalized ALT levels at the end of study and at time points SVR is assessed.
- Change in liver disease stage assessment between screening assessment and assessment at SVR24 time point

For all subjects per assigned total treatment duration and combined for both HCV genotypes (subanalyses for each genotype separately will also be done):

- mean change from baseline at each study visit throughout treatment and follow-up for each of the PRO measures (HCV-SIQ symptom score, HCV impact score, FSS total score, CES-D score, WPAI missed work time, WPAI daily activity impairment, WPAI productivity score, EQ5D VAS, EQ5D valuation index, EQ5D Descriptive System scores);

For definitions of RVR, W2VR, viral breakthrough and relapse, see Definition of Terms.

Exploratory PRO endpoints

In subjects infected with genotype 1 or genotype 4 HCV for both genotypes combined (subanalyses for each genotype separately will also be done):

- Mean change from baseline at each study visit throughout treatment and follow-up for the HCV-SIQ total score
- Mean change from baseline at each study visit throughout treatment and follow-up for the HCV-SIQ symptom scores by body system

Safety

Safety endpoints will be analysed in subjects infected with genotype 1 or genotype 4 HCV separately per genotype and for both genotypes combined:

Adverse Events: The verbatim terms used in the CRF by investigators to identify AEs will be coded using the Medical Dictionary for Regulatory Activities (MedDRA). All reported AEs with onset during the treatment phase (i.e., treatment-emergent AEs, and AEs that have worsened since baseline) will be included in the analysis. For each AE, the percentage of subjects who experience at least 1 occurrence of the given event will be summarized by treatment group. Summaries, listings, datasets, or subject narratives may be provided, as appropriate, for those subjects who die, who discontinue treatment due to an AE, or who experience a severe or a serious adverse event (SAE).

Clinical Laboratory Tests: Laboratory data will be summarized by type of laboratory test. Descriptive statistics (actual values and changes from reference) will be calculated for each laboratory analyte at baseline and at each scheduled time point. Laboratory abnormalities will be determined according to the WHO grading table and in accordance with the normal ranges of the clinical laboratory.

Vital Signs: Descriptive statistics of pulse rate and blood pressure (systolic and diastolic) values and changes from baseline will be summarized at each scheduled time point. The percentage of subjects with values beyond clinically important limits will be summarized.

Physical Examination: Findings and changes from baseline will be summarized at each scheduled time point. Abnormalities will be listed.

TIME AND EVENTS SCHEDULE - PART 1: FOR ALL SUBJECTS (UP TO WEEK 12)

Period	Scr[a]	BL[b]	Study Visits[c]				
Visit Number	V1	V2	V3	V4	V5	V6	V7
Study Day		1	7	14	28		
Study Week	-6	0	1	2	4	8	12
**Study Procedure**							
Informed consent (ICF)[d]	X						
Demographics	X						
Medical history/Concomitant diseases	X						
Inclusion/exclusion criteria[e]	X						
Liver disease status[f]	X						
ECG (performed locally)	X						
Pharmacogenomics							
Host *IL28B* polymorphism analysis	X[g]						
Clinical Laboratory Tests							
Hepatitis A/B/C test	X						
Hepatitis C virus genotype-subtype determination	X						
HIV-1/HIV-2 test	X						
Schistosomiasis serology test[h]	X						
Alpha-fetoprotein (AFP) and follicle-stimulating hormone (FSH)[i]	X						
Glycosylated hemoglobin (HbA1c) and antinuclear antibody (ANA)	X						
Thyroid-stimulating hormone (TSH)[j]	X	X[k]					X
Hematology/biochemistry[l]	X	X[k]	X	X	X	X	X
Local urinalysis[m]	X[n]	X[k]	X	X	X	X	X
Pregnancy test[o]	X	X			X	X	X
Study Drug Administration							
Dispense TMC435/PegIFNα-2a/RBV: all subjects		X			X	X	
Dispense PegIFNα-2a/RBV: Extension[p]							X
Efficacy Assessments							
HCV RNA quantification	X	X[k]	X	X	X	X	X
Viral sequencing[q]		X[k]	X	X	X	X	X
Other Safety Assessments							
Physical examination[r]	X	X	X	X	X	X	X
Vital signs	X	X	X	X	X	X	X

Period	Scr[a]	BL[b]	Study Visits[c]				
Visit Number	V1	V2	V3	V4	V5	V6	V7
Study Day		1	7	14	28		
Study Week	-6	0	1	2	4	8	12
**Study Procedure**							
Patient reported outcomes (PRO)							
Hepatitis C Symptom & Impact Questionnaire (HCV-SIQ)[s]	X	X	X	X	X	X	X
Fatigue (Fatigue severity scale [FSS])[s]		X			X	X	X
Productivity (Work Productivity and Activity Impairment [WPAI])[s]		X			X	X	X
Depressive symptom severity (Center for Epidemiologic Studies Depression Scale [CES-D])[s]		X			X	X	X
Health status (EuroQol 5-dimension questionnaire [EQ-5D])[s]		X			X	X	X
Ongoing Subject Review							
Concomitant therapy	X	X	X	X	X	X	X
Observe/interview for (serious) adverse events[t]	X	X	X	X	X	X	X

Prescr: Prescreening; Scr: screening; BL: baseline;

Footnotes

[a] Screening within 6 weeks of planned baseline visit. Prolongation of this period for legitimate reasons may be allowed upon written approval from the sponsor. Screening assessments can be done during the screening period and need not necessarily be done at the same moment. When the cap for enrolment has been reached for a specific IL28B genotype, stepwise screening can be performed, ie after signing of the ICF, IL28B screening can be done first. If the lab results show that the subject has another IL28B genotype than the one for which the enrolment cap was reached, further screening assessments can be undertaken.. Upon confirmation of all eligibility parameters, the baseline visit can take place.

[b] The baseline visit should take place as soon as all details to confirm eligibility have been obtained.

[c] Subjects who prematurely discontinue all study medication (TMC435/PegIFNα-2a/RBV) should switch to the visit schedule as laid out in Time and Events Schedule - Part 4 (unless the subject withdraws consent).

[d] Including integrated consent for mandatory *IL28B* polymorphism analysis

[e] Minimum criteria for the availability of documentation supporting the eligibility criteria are described in Section 17.4, Source Documentation.

[f] Confirmation of liver disease status either by liver biopsy or non-invasive method as described in Section 4.1, Inclusion Criteria. If results are available from either a liver biopsy performed within 2 years prior to the screening visit or from non-invasive confirmation of the liver condition (by transient elastography) performed within 6 months prior to the screening visit, this assessment does not need to be repeated.

[g] After the results have been obtained, all remaining blood samples for *IL28B* genotyping will be destroyed.

[h] Only for subjects infected with genotype 4 HCV. If the schistosomiasis antibody test is positive, a schistosomiasis antigen blood test or rectal snip should be performed. Subjects with a positive schistosomiasis antigen blood test or positive rectal snip are excluded from study participation .

[i] Alpha-fetoprotein (AFP) will be tested for all subjects. Follicle-stimulating hormone (FSH) will be tested for female subjects who are postmenopausal for less than 2 years.

[j] Free thyroxine (T4) and free triiodothyronine (T3) will be assessed if TSH is abnormal.

[k] Samples to be taken predose

[l] The biochemistry sample at screening must be taken after the subject fasted for at least 12 hours. The biochemistry samples at baseline (Day 1) and Weeks 4 and 12 must be taken after fasting for at least 10 hours. It is recommended that biochemistry samples at all other time points also be taken after fasting for at least 10h. The homeostasis model assessment for insulin resistance (HOMA IR) index will be derived from insulin and glucose (fasted) test results at baseline and Weeks 4 and 12.

[m] Urinalysis performed locally by dipstick. If there are clinically relevant abnormalities in the investigator's opinion, further urinalysis should be done locally (e.g., sediment analysis including RBC, WBC, casts). Clinically relevant findings from local urinalysis will be reported as AEs.

[n] Including drug screen at central laboratory

[o] Serum test at screening for all female subjects, urine test at other visits for women of childbearing potential. Pregnancy test should be performed prior to dosing.

[p] See Time and Events Schedule - Part 3

[q] Sequencing of the HCV NS3/4A region will be done in real-time for the baseline sample. Sequencing of the other samples will be at the discretion of the Study Responsible Scientist.

[r] Including height and weight at screening. Physical examination at screening should include a fundoscopic eye examination, as per the manufacturer's prescribing information for PegIFNα-2a. Note: Baseline fundoscopic pathology which would require routine follow up on PegIFNα-2a and RBV therapy is considered exclusionary, as per exclusion criterion 7.

[s] PRO scales should be completed prior to any other study-related activity at each visit.

[t] From signing the Informed Consent Form (ICF) until 4 weeks after last study medication intake. Thereafter, only AEs considered related to TMC435 will be reported. SAEs will be reported until 30 days after last study-related procedure.

TIME AND EVENTS SCHEDULE - PART 2: FROM WEEK 12 TO WEEK 36 FOR SUBJECTS ELIGIBLE FOR A TOTAL TREATMENT DURATION OF 12 WEEKS

Period	Study Visits			
Visit Number	V8	V9	V10	V11
Study Week	16	20	24	36
**Study Procedure**				
Clinical Laboratory Tests				
TSH[a]			X	X
Hematology/biochemistry[b]	X	X	X	X
Local urinalysis[c]	X	X	X	X
Pregnancy test[d]	X	X	X	X
Efficacy Assessments				
HCV RNA quantification	X	X	X	X
Viral sequencing[e]	X	X	X	X
Liver disease status[f]				X
Other Safety Assessments				
Physical examination	X	X	X	X
Vital signs	X	X	X	X
Patient reported outcomes (PRO)				
Hepatitis C Symptom & Impact Questionnaire (HCV-SIQ)[g]	X	X	X	X
Fatigue (Fatigue severity scale [FSS])[g]	X		X	X
Productivity (Work Productivity and Activity Impairment [WPAI])[g]	X		X	X
Depressive symptom severity (Center for Epidemiologic Studies Depression Scale [CES-D])[g]	X		X	X
Health status (EuroQol 5-dimension questionnaire [EQ-5D])[g]	X		X	X
Ongoing Subject Review				
Concomitant therapy	X	X	X	X
Observe/interview for (serious) adverse events[h]	X	X	X	X

Footnotes

a Free thyroxine (T4) and free triiodothyronine (T3) will be assessed if TSH is abnormal.

b The biochemistry sample at Weeks 24 and 36 must be taken after fasting for at least 10 hours. It is recommended that biochemistry samples at all other time points also be taken after fasting for at least 10h. The homeostasis model assessment for insulin resistance (HOMA IR) index will be derived from insulin and glucose (fasted) test results at Weeks 24 and 36.

c Urinalysis performed locally by dipstick. If there are clinically relevant abnormalities in the investigator's opinion, further urinalysis should be done locally (e.g., sediment analysis including RBC, WBC, casts). Clinically relevant findings from local urinalysis will be reported as AEs.

d Urine test for women of childbearing potential. Extra pregnancy test kits will be given if more than 4 weeks between two consecutive study visits to perform a pregnancy test at home.

[e] Sequencing of the HCV NS3/4A region will be done at the discretion of the Study Responsible Scientist.

[f] Non-invasive confirmation of the liver condition (by transient elastography). All effort should be made to use the same assessment method as used to assess fibrosis stage at inclusion.

[g] PRO scales should be completed prior to any other study-related activity at each visit.

[h] From signing the Informed Consent Form (ICF) until 4 weeks after last study medication intake. Thereafter, only AEs considered related to TMC435 will be reported. SAEs will be reported until 30 days after last study-related procedure.

TIME AND EVENTS SCHEDULE - PART 3: FROM WEEK 12 TO WEEK 48 FOR SUBJECTS ELIGIBLE FOR A TOTAL TREATMENT DURATION OF 24 WEEKS (EXTENSION)

Period	Study Visits[a]					
Visit Number	V8	V9	V10	V11	V12	V13
Study Week	16	20	24	28	36	48
**Study Procedure**						
Clinical Laboratory Tests						
TSH[b]			X		X	X
Hematology/biochemistry[c]	X	X	X	X	X	X
Local urinalysis[d]	X	X	X	X	X	X
Pregnancy test[e]	X	X	X	X	X	X
Study Drug Administration						
Dispense PegIFNα-2a/RBV: Extension	X	X				
Efficacy Assessments						
HCV RNA quantification	X	X	X	X	X	X
Viral sequencing[f]	X	X	X	X	X	X
Liver disease status[g]						X
Other Safety Assessments						
Physical examination	X	X	X	X	X	X
Vital signs	X	X	X	X	X	X
Patient reported outcomes (PRO)			X			
Hepatitis C Symptom & Impact Questionnaire (HCV-SIQ)[h]	X	X	X	X	X	X
Fatigue (Fatigue severity scale [FSS])[h]	X	X	X		X	X
Productivity (Work Productivity and Activity Impairment [WPAI][h]	X	X	X		X	X
Depressive symptom severity (Center for Epidemiologic Studies Depression Scale [CES-D])[h]	X	X	X		X	X
Health status (EuroQol 5-dimension questionnaire [EQ-5D])[h]	X	X	X		X	X
Ongoing Subject Review						
Concomitant therapy	X	X	X	X	X	X
Observe/interview for (serious) adverse events[i]	X	X	X	X	X	X

Footnotes

[a] Subjects who prematurely discontinue all study medication (PegIFNα-2a/RBV) should switch to the visit schedule as laid out in Time and Events Schedule - Part 4 (unless the subject withdraws consent).

[b] Free thyroxine (T4) and free triiodothyronine (T3) will be assessed if TSH is abnormal.

[c] The biochemistry sample at Weeks 24, 36, and 48 must be taken after fasting for at least 10 hours. It is recommended that biochemistry samples at all other time points also be taken after fasting for at least 10h. The homeostasis model assessment for insulin resistance (HOMA IR) index will be derived from insulin and glucose (fasted) test results at Weeks 24, 36, and 48.

[d] Urinalysis performed locally by dipstick. If there are clinically relevant abnormalities in the investigator's opinion, further urinalysis should be done locally (e.g., sediment analysis including RBC, WBC, casts). Clinically relevant findings from local urinalysis will be reported as AEs.

[e] Urine test for women of childbearing potential. Extra pregnancy test kits will be given if more than 4 weeks between two consecutive study visits to perform a pregnancy test at home.

[f] Sequencing of the HCV NS3/4A region will be done at the discretion of the Study Responsible Scientist.

[g] Non-invasive confirmation of the liver condition (by transient elastography). All effort should be made to use the same assessment method as used to assess fibrosis stage at inclusion.

[h] PRO scales should be completed prior to any other study-related activity at each visit.

[i] From signing the ICF until 4 weeks after last study medication intake. Thereafter, only AEs considered related to TMC435 will be reported. SAEs will be reported until 30 days after last study-related procedure.

TIME AND EVENTS SCHEDULE - PART 4: IN CASE OF PREMATURE DISCONTINUATION OF ALL STUDY MEDICATION (TMC435/PEGIFNα-2A/RBV DURING THE FIRST 12 WEEKS OR PEGIFNα-2A/RBV AFTER WEEK 12)

Visit Type	Early Withdrawal Visit	Follow-up after early withdrawal[a]	
Study Week	At time of early discontinuation of all study medication	4 weeks after study medication withdrawal	Every 12 weeks (counting from baseline) until Week 36 or Week 48[b]
**Study Procedure**			
Clinical Laboratory Tests			
TSH[c]	X	X	
Hematology/biochemistry[d]	X	X	X
Local urinalysis[e]	X	X	X
Pregnancy test[f]	X	X	X
Efficacy Assessments			
HCV RNA quantification	X	X	X
Viral sequencing[g]	X	X	X
Other Safety Assessments			
Physical examination	X	X	X
Vital signs	X	X	X
Patient reported outcomes (PRO)			
Hepatitis C Symptom & Impact Questionnaire (HCV-SIQ)[h]	X	X	X
Fatigue (Fatigue severity scale [FSS])[h]	X		X
Productivity (Work Productivity and Activity Impairment [WPAI])[h]	X		X
Depressive symptom severity (Center for Epidemiologic Studies Depression Scale [CES-D])[h]	X		X
Health status (EuroQol 5-dimension questionnaire [EQ-5D])[h]	X		X
Ongoing Subject Review			
Concomitant therapy	X	X	X
Observe/interview for (serious) adverse eventsi	X	X	X

Footnotes

[a] Duration of follow-up after early withdrawal will depend on the planned duration of treatment (see Section 3.1):

- subjects who prematurely discontinue all study medication before Week 8 will be followed until Week 36.
- subjects who would have qualified for a total treatment duration of 12 weeks but prematurely discontinued after Week 8 will be followed until Week 36.
- subjects who would have qualified for total treatment duration of 24 weeks but prematurely discontinued after Week 8 will be followed until Week 48.
- subjects who discontinue treatment because they meet a virologic stopping rule at Week 4 or Week 12 will be followed until Week 36.
- subjects who discontinue treatment due to viral breakthrough will be followed until 4 weeks after withdrawal (i.e., they will come back for visits at time of discontinuation and 4 weeks after study medication withdrawal)

[b] The first scheduled visit (every 12 weeks from baseline) after study medication withdrawal will not be performed if it coincides (with a time window of ± 7 calendar days) with the Week 4 visit after study medication withdrawal. In such a case, every effort should be made to ensure that for subsequent visits the schedule every 12 weeks from baseline is adhered to.

[c] Free thyroxine (T4) and free triiodothyronine (T3) will be assessed if TSH is abnormal.

[d] The biochemistry sample at 12-weekly intervals (counting from baseline) must be taken after fasting for at least 10 hours. It is recommended that biochemistry samples at all other time points also be taken after fasting for at least 10h. The homeostasis model assessment for insulin resistance (HOMA IR) index will be derived from insulin and glucose (fasted) test results at 12-weekly intervals (counting from baseline).

[e] Urinalysis performed locally by dipstick. If there are clinically relevant abnormalities in the investigator's opinion, further urinalysis should be done locally (e.g., sediment analysis including RBC, WBC, casts). Clinically relevant findings from local urinalysis will be reported as AEs.

[f] Urine test for women of childbearing potential. Extra pregnancy test kits will be given if more than 4 weeks between two consecutive study visits to perform a pregnancy test at home.

[g] Sequencing of the HCV NS3/4A region will be done at the discretion of the Study Responsible Scientist.

[h] PRO scales should be completed prior to any other study-related activity at each visit.

[i] From signing the ICF until 4 weeks after last study medication intake. Thereafter, only AEs considered related to TMC435 will be reported. SAEs will be reported until 30 days after last study-related procedure.

ABBREVIATIONS

β-HCG	β-human chorionic gonadotropin
AE	adverse event
AFP	alpha-fetoprotein
ALT	alanine aminotransferase
ANA	antinuclear antibody
ANC	absolute neutrohil count
AST	aspartate aminotransferase
AUC	area under concentration-time curve
b.i.d.	bis in die; twice daily
BMI	Body Mass Index
BUN	blood urea nitrogen
CC_{50}	50% cytotoxic concentration in cell-based assays
CES-D	Center for Epidemiologic Studies Depression Scale
CLcr	creatinine clearance
C_{max}	maximum plasma concentration
CPK	creatine phosphokinase
CRF	case report form (paper or electronic as appropriate for this study)
CT	computed tomography
CYP	cytochrome P450
DAA	direct-acting antiviral
DBP	diastolic blood pressure
DNA	deoxyribonucleic acid
DMC	Data Monitoring Committee
DSM-IV	Diagnostic and Statistical Manual of Mental Disorders (4th edition)
DSUR	development safety update report
EC	Ethics Committee
EC_{50}	50% effective concentration in cell-based assays
EC_{90}	90% effective concentration in cell-based assays
ECG	electrocardiogram
eCRF	electronic case report form
eDC	electronic data capture
EQ-5D	EuroQol 5-dimension questionnaire
FDA	US Food and Drug Administration
FSH	follicle stimulating hormone
FSS	Fatigue Severity Scale
GCP	Good Clinical Practice
GGT	gamma glutamyl transferase
HA	Health Authorities
HbA1c	glycosylated hemoglobin
HBsAg	hepatitis B surface antigen
HCV	hepatitis C virus
HCV-SIQ	Hepatitis C Symptom & Impact Questionnaire
HDL	high-density lipoprotein
HIV	human immunodeficiency virus
HOMA-IR	homeostasis model assessment for insulin resistance
HRQoL	health-related quality of life
IB	Investigator's Brochure
ICF	Informed Consent Form
ICH	International Conference on Harmonisation
IEC	Independent Ethics Committee
IgM	immunoglobulin M
IL	interleukin
IL28B	interleukin 28B
INR	international normalized ratio
IRB	Institutional Review Board
IUD	intrauterine device

IUS	intrauterine system
IWRS	interactive web-based response system
Ki	kinetic inhibition constant
LDH	lactic acid dehydrogenase
LDL	low-density lipoprotein
LLN	lower limit of normal range
MCH	mean corpuscular hemoglobin
MCHC	mean corpuscular hemoglobin concentration
MCV	mean corpuscular volume
MedDRA	Medical Dictionary for Regulatory Activities
MRI	magnetic resonance imaging
MRP2	multidrug resistance-associated protein 2
OATP1B1	organic anion transporting polypeptide 1B1
P-gp	P-glycoprotein
PegIFNα-2a	pegylated interferon alfa-2a
PI	protease inhibitor
PQC	Product Quality Complaint
PRO	patient-reported outcome
PT	prothrombin time
q.d.	quaque die, once daily
QTc	QT interval corrected for heart rate
RBC	red blood cell
RBV	ribavirin
RGT	response-guided treatment
RNA	ribonucleic acid
RVR	rapid virologic response
SAE	serious adverse event
SBP	systolic blood pressure
SC	subcutaneous
SD	standard deviation
SJS	Stevens-Johnson syndrome
SVR	sustained virologic response
$t_{1/2,term}$	terminal elimination half-life
T_3	triiodothyronine
T_4	thyroxine
t_{max}	time to reach the maximum plasma concentration
TMC	Tibotec Medicinal Compound
TSH	thyroid stimulating hormone
ULN	upper limit of normal range
vs	versus
W2VR	virologic response at Week 2
WBC	white blood cell
WHO	World Health Organisation
WPAI	Work Productivity and Activity Impairment questionnaire

DEFINITION OF TERMS

RVR	HCV RNA < 25 IU/mL undetectable measured 4 weeks after start of treatment
SVR12	sustained virologic response 12 weeks after planned end of treatment<br>Subjects are considered to have reached SVR12 if both conditions below are met:<br>1) at the actual end of treatment<br>• HCV RNA levels < 25 IU/mL undetectable,<br>AND<br>2) at the time point of SVR12 (i.e., 12 weeks after the planned EOT)<br>• HCV RNA levels < 25 IU/mL undetectable.
SVR24	sustained virologic response 24 weeks after planned end of treatment<br>Subjects are considered to have reached SVR24 if both conditions below are met:<br>1) at the actual end of treatment<br>• HCV RNA levels < 25 IU/mL undetectable,<br>AND<br>2) at the time point of SVR24 (i.e., 24 weeks after the planned EOT)<br>• HCV RNA levels < 25 IU/mL undetectable.
Triple therapy	Treatment for HCV infection consisting of a direct-acting anti-viral agent (DAA) plus PegIFN/RBV
viral breakthrough	Confirmed increase of > 1 $\log_{10}$ IU/mL in HCV RNA level from the lowest level reached, or a confirmed HCV RNA level of > 100 IU/mL in subjects whose HCV RNA levels had previously been below the limit of quantification (< 25 IU/mL detectable) or undetectable (< 25 IU/mL undetectable) while on study treatment.
W2VR	HCV RNA < 25 IU/mL (detectable or undetectable) measured 2 weeks after start of treatment.
viral relapse	Subjects are considered to have a viral relapse if both conditions below are met:<br>At actual end of treatment:<br>• HCV RNA levels < 25 IU/mL undetectable,<br>AND<br>During the follow-up period:<br>• HCV RNA levels ≥ 25 IU/mL.

1. INTRODUCTION

TMC435 (simeprevir, formerly known as TMC435350) is a NS3/4A protease inhibitor (PI) in development for treatment of chronic hepatitis C virus (HCV) infection. Based on the available data from Phase 3 clinical studies, it is anticipated that TMC435 may provide added benefit in terms of a better adverse event (AE) profile and potentially higher efficacy in some subpopulations than the currently approved PIs (telaprevir and boceprevir).

The term "sponsor" used throughout this document refers to the entities listed in the Contact Information page(s), which will be provided as a separate document.

1.1. Background

Infection with HCV is a leading cause of liver disease worldwide and has become a focus of considerable medical research. An estimated 170 million people (3% of the global population) are currently infected with HCV, with approximately 2 to 4 million people newly infected each year[27,46]. More than 50% of HCV infections become chronic and may lead to the development of liver cirrhosis, chronic liver failure and hepatocellular carcinoma. Complications of liver disease due to HCV are the leading cause for liver transplantation.

1.1.1. Genotype 1

In the past 10 years, treatment of chronic hepatitis C had been exclusively based on pegylated interferon alfa (PegIFNα) in combination with ribavirin (RBV). However, this combination therapy has limited efficacy on genotype-1 HCV, yielding sustained virologic response (SVR) rates of approximately 45% in treatment-naïve subjects, 20% to 30% in prior relapsers, and 5% to 15% in prior non-responders (null and partial responders)[2,12,26,31,41,45]. Treatment options for patients who fail PegIFNα/RBV therapy remain limited. In addition to the inadequate efficacy in chronic genotype-1 HCV infection, the combination PegIFNα/RBV requires 48 weeks of treatment for chronic genotype-1 HCV infection, has significant side effects, and is poorly tolerated in some subjects. Treatment-related side effects include influenza-like symptoms, hematological abnormalities, and neuropsychiatric symptoms[12,21,24,26,31,41].

The treatment paradigm has changed to triple therapy including PegIFNα and RBV in combination with a direct-acting anti-viral (DAA) agent. The HCV encoded NS3/4A protease is essential for viral replication, and multiple NS3/4A PIs, including TMC435, are currently in clinical development. Recently, the HCV NS3/4A PIs telaprevir and boceprevir obtained marketing approval. In studies with telaprevir and boceprevir, it was demonstrated that substantially improved SVR rates can be achieved when these agents are administered in combination with PegIFNα and RBV in chronic HCV genotype-1 infected treatment-naïve and treatment-experienced subjects[3,17,20,28,34,35,40,44,47]. In addition to the improved SVR rates, these studies demonstrated that a significant proportion of treatment-naïve subjects achieved high cure rates with shorter overall treatment duration. However, adverse effects and complicated treatment schedules with a high pill burden may lead to poor adherence and SVR rates remain low in difficult-to-cure populations, such as patients with null response to previous IFN-based therapy or patients with advanced liver disease. This highlights the persistent unmet need for new HCV inhibitors that could further improve outcomes and shorten treatment duration, have a

lower pill burden to increase compliance, have increased efficacy in difficult-to-cure subgroups and without any substantial increase in treatment-related toxicity.

1.1.2. Genotype 4

Hepatitis C virus genotype 4 (HCV genotype 4) is the most prevalent genotype in the Middle East, North Africa and sub-Saharan Africa. Egypt has the highest worldwide incidence and prevalence of HCV, where HCV genotype 4 is the cause of 90% of HCV infections. HCV genotype 4 has recently spread to Southern Europe through immigration and injection drug use. In France, for example, the prevalence rate of HCV genotype 4 has increased from 4% in 1990 to more than 11% in a decade[22].

Although approximately 20% of the worldwide HCV population is infected with genotype 4, these patients have been underrepresented in large multicentre clinical studies because of limited prevalence of this genotype in Europe and the United States. As a result, data regarding the responsiveness of genotype 4 are limited.

PegIFNα/RBV 48-week therapy for HCV genotype 4 results in sustained virologic response (SVR) rates of around 60% (ranging between 50% and 79%)[22]. Shorter treatment of chronic HCV genotype 4 patients with rapid and early virologic responses has been associated with high SVR rates, better compliance, fewer AEs and lower costs[22]. Despite this recent progress, the treatment of HCV genotype 4 in certain patient groups, such as patients who failed PegIFNα/RBV therapy, still represents a significant therapeutic challenge. In addition, more research is required to optimize current therapy for patients infected with genotype 4 HCV, which requires inclusion of genotype 4 patients in clinical studies evaluating direct-acting antiviral agents (DAAs) with activity against genotype 4 such as PIs, NS5A or NS5B inhibitors.

The following summarizes TMC435 findings at the time of initial protocol writing. For a more comprehensive nonclinical and clinical information regarding TMC435, refer to the latest version of the Investigator's Brochure (IB) for TMC435[18] and its addendum[19].

1.2. Genotype 1

Nonclinical Studies[18]

TMC435 is an inhibitor of the HCV NS3/4A protease, with median in vitro kinetic inhibition constant (Ki) values of 0.5 nM and 1.4 nM against the HCV genotype 1a (H77) and 1b (con1) proteases, respectively. The in vitro anti-HCV median 50% and 90% effective concentrations (EC_{50} and EC_{90}) of TMC435 were 9.4 and 19 nM, respectively, in a genotype 1b replicon system. In vitro characterization of the resistance profile of TMC435 showed that some amino acid changes at positions 43, 80, 122, 155, 156, and 168 of the NS3 region resulted in reductions in TMC435 activity, with D168V having the largest effect (2,830-fold increase in TMC435 EC_{50}). Q80K reduced TMC435 activity by 7.7- and 9.3-fold when tested as a site-directed mutant in a genotype 1b and 1a replicon, respectively. In vitro combination of TMC435 with anti-HCV agents with a different mode of action resulted in additive or synergistic effects.

TMC435 safety pharmacology studies did not detect any significant signals that are considered causes for concern. Therefore, TMC435 is considered to have very limited potential for

cardiovascular, pulmonary or nervous system effects at the proposed clinical dose of TMC435. After oral repeated dosing, the liver was the primary target organ of TMC435. TMC435 was not genotoxic or clastogenic and had no effect on fertility, embryofetal or pre- and postnatal development. TMC435 was mildly irritant to the eyes, but was not likely to cause skin sensitization and was not irritating to the skin. TMC435 was phototoxic after ultraviolet A exposure in vitro.

Clinical Studies[18,19]

At the time of initial protocol writing, data were available from 38 core clinical studies: 7 Phase 3 studies, 2 Phase 2b studies, 2 Phase 2a studies, and 27 Phase 1 studies. In these 38 studies, a total of 2,652 subjects were exposed to TMC435, including 1,846 HCV-infected and 806 healthy subjects. Of these, 1,153 HCV-infected subjects were treated with TMC435 150 mg once daily for 12 weeks in combination with pegylated interferon and ribavirin (PegIFN/RBV) for 24 or 48 weeks.

Ongoing development includes the evaluations of TMC435 as part of IFN-free regimens. Data are available from the ongoing Phase 2a study HPC2002 investigating the safety and efficacy of 12 or 24 weeks of TMC435 150 mg once daily combined with GS-7977 (sofosbuvir) 400 mg once daily with or without RBV in subjects with chronic HCV genotype 1 infection.

In parallel to the core clinical development program and the IFN-free studies, a separate clinical development program with 100 mg TMC435 in combination with PegIFN/RBV is being conducted in Japan. This program includes 7 studies: 2 Phase 1 studies in healthy Japanese subjects and 5 studies (1 phase 2b and 4 phase 3 studies) in subjects infected with HCV genotype 1.

Human Pharmacokinetics[18]

TMC435 has an oral bioavailability of 62% after a single dose of 150 mg. The maximum plasma concentration (C_{max}) is attained approximately 4 to 6 hours after administration and the mean terminal elimination half-life ($t_{1/2,term}$) of TMC435 was approximately 10 to 13 hours in healthy subjects. In HCV-infected subjects, the mean $t_{1/2,term}$ of TMC435 was 41.3 hours after multiple dosing at 200 mg once daily. Steady-state conditions are reached after 7 days of dosing. The exposure of TMC435 was generally higher (approximately 2- to 3-fold) in HCV-infected subjects compared with healthy subjects.

Compared to fasting conditions, the exposure (area under concentration-time curve [AUC]) of TMC435 administered as the Phase 3 150 mg capsule was 1.69-fold and 1.61-fold higher following a normal breakfast (21 g fat, 533 kcal) and a high-fat, high caloric breakfast (56 g fat, 928 kcal), respectively. The median time to C_{max} was delayed by 1 to 1.5 hours under fed conditions.

TMC435 is predominantly excreted in feces. Renal excretion of TMC435 is negligible.

Cytochrome P450 (CYP) 3A enzymes are mainly involved in the metabolism of TMC435. TMC435 is a mild inhibitor of intestinal (but not hepatic) CYP3A activity and a mild inhibitor of

CYP1A2. In addition, drug-drug interaction studies with transporter substrates suggest that TMC435 likely inhibits P-glycoprotein (P-gp) and organic anion transporting polypeptide (OATP) 1B1 in vivo.

Efficacy Data[18]

In subjects infected with HCV genotype 1, treatment with TMC435 in combination with PegIFN/RBV resulted in statistically significantly higher SVR rates compared with PegIFN/RBV alone regardless of pretreatment category (i.e., treatment-naïve subjects, prior relapsers, prior partial and prior null responders).

- **Treatment-Naïve Subjects**

  In treatment-naïve subjects (Phase 3 studies C208 and C216 combined), SVR at 12 weeks after the planned end of treatment (SVR12) was achieved in 80.4% of subjects treated with TMC435 150 mg once daily for 12 weeks in combination with PegIFN/RBV, compared to 50.0% of subjects treated with PegIFN/RBV alone.

- **Prior Relapsers**

  In subjects with relapse to prior IFN-based therapy (Phase 3 study HPC3007), SVR12 was achieved in 79.2% of subjects treated with TMC435 150 mg once daily for 12 weeks in combination with PegIFN/RBV compared to 36.8% of subjects treated with PegIFN/RBV alone.

  In subjects with relapse to prior PegIFN/RBV-based therapy (Phase 2b study C206), SVR at 24 weeks after planned end of treatment (SVR24) was achieved in 84.8% of subjects treated with TMC435 150 mg once daily in combination with PegIFN/RBV compared to 37.0% of subjects treated with PegIFN/RBV alone.

- **Prior Partial and Null Responders**

  In prior partial and null responders to PegIFN/RBV (Phase 2b study C206), SVR24 was achieved in a statistically significantly higher percentage of subjects treated with TMC435 150 mg once daily in combination with PegIFN/RBV (pooled data from 3 treatment duration groups: 12, 24 and 48 weeks of TMC435 treatment) compared to subjects treated with PegIFN/RBV alone: 75.4% (52/69) versus (vs) 8.7% (2/23) in prior partial responders and 51.0% (26/51) vs 18.8% (3/16) in prior null responders.

Shortening of treatment duration was evaluated in treatment-naïve and prior relapser subjects. A high proportion of treatment-naïve subjects (88.1%, pooled studies C208 and C216) and prior relapsers (92.7%, study HPC3007) who were treated with TMC435 in combination with PegIFN/RBV were eligible for shorter treatment duration of PegIFN/RBV therapy (24 weeks instead of 48 weeks) according to the protocol-defined response-guided treatment (RGT) criteria.

Overall, significantly higher SVR rates with TMC435 compared to PegIFN/RBV alone were observed across subgroups of demographic factors and baseline disease characteristics including sex, age, race, body mass index (BMI), HCV genotype, baseline HCV RNA (≤800,000 IU/mL, ≥800,000 IU/mL), Metavir fibrosis score, and interleukin-28B (*IL28B)* genotype (Phase 3 studies C208, C216, HPC3007).

Multivariate as well as subgroup analyses across studies C208, C216, and HPC3007 identified several baseline characteristics that affected SVR rates. The strongest prognostic factors for treatment outcome of TMC435 in combination with PegIFN/RBV included *IL28B* genotype (with TT resulting in the lowest SVR rates), Metavir fibrosis score (with F4 resulting in the lowest SVR rates) and the presence of baseline Q80K polymorphism in HCV genotype 1a infected subjects (Q80K polymorphism was only rarely observed in HCV genotype 1b).

***Safety and Tolerability Data*[18]**

Overall, in the pooled Phase 3 studies C208, C216 and HPC3007, similar incidences of AEs by type and preferred term were seen for TMC435-treated subjects and subjects receiving placebo. By preferred term, the most frequent AEs (>25% of TMC435-treated subjects) were fatigue (35.6%), headache (33.2%) and influenza-like illness (26.0%), all of which are common adverse reactions of PegIFN/RBV treatment. The incidence of these AEs was similar in subjects receiving placebo. By preferred term, pruritus was the only AE with a notably (>5%) higher incidence in TMC435-treated subjects than in subjects receiving placebo (20.6% vs 13.6%). Adverse events considered by the investigator to be related to study drug were reported in 69.4% of TMC435-treated subjects and in 57.7% of subjects receiving placebo.

Most AEs were grade 1 or grade 2. Grade 3 AEs were reported in 20.0% of TMC435-treated subjects and 21.9% of subjects receiving placebo. Grade 4 AEs were reported in 2.9% and 2.8%, respectively. By preferred term, all grade 3 or grade 4 AEs were reported in <5.0% of subjects, with the exception of neutropenia, which was reported in 9.2% of TMC435-treated subjects and in 8.6% of subjects receiving placebo.

Serious adverse events (SAEs) were reported in 2.0% of TMC435-treated subjects and 2.5% of subjects receiving placebo. There were no deaths during the first 12 weeks of treatment, but 3 TMC435-treated subjects died during the entire treatment phase, after completion of TMC435 treatment. None of these deaths were considered related to TMC435 or placebo.

TMC435/placebo (without regard to PegIFN and RBV) was discontinued prematurely due to the occurrence of AE in 1.8% of TMC435-treated subjects and 1.3% of subjects receiving placebo. Rash was the most common preferred term reported as AE leading to premature discontinuation of TMC435/placebo (5 [0.6%] TMC435-treated subjects).

Certain grouped terms were considered of special (increased bilirubin) and clinical interest (rash [any type], pruritus, anemia, neutropenia, and photosensitivity conditions). The incidence in TMC435-treated subjects was higher than in subjects receiving placebo for increased bilirubin (7.9% vs 2.8%), rash (any type) (23.2% vs 16.9%), photosensitivity conditions (3.3% vs 0.5%), and pruritus (22.0% vs 14.9%). For anemia and neutropenia, the incidences were similar in both treatment groups.

At the time of initial protocol writing, the following adverse reactions had been identified for TMC435: constipation, blood bilirubin increased, photosensitivity reaction, pruritus, and rash.

Most adverse reactions were grade 1 or 2 in severity. Overall, there was no relevant difference in severity between TMC435 and placebo treatment groups.

The elevations in direct and indirect bilirubin were mostly of mild or moderate severity and were reversible. Bilirubin elevations were generally not associated with elevations in transaminases and are attributed to a decrease in bilirubin elimination related to inhibition of the hepatocyte transporters OATP1B1 and multidrug resistance-associated protein 2 (MRP2) by TMC435. These changes are not considered to be clinically relevant.

Subgroup analysis of AEs in general and events of special/clinical interest by age, sex, race, region, BMI and Metavir fibrosis score indicated that, overall, the safety and tolerability profile of TMC435 was similar, regardless of demographic and disease characteristics.

1.3. Genotype 4

Efficacy Data[18]

An open-label Phase 2a study (C202)[6] in treatment-naïve subjects with genotype 2 (n=6), 3 (n=8), 4 (n=8), 5 (n=7) or 6 (n=8) HCV infection provided evidence that TMC435 has a spectrum of activity broader than genotype 1 infection[6]. Subjects received TMC435 200 mg qd for 7 consecutive days as monotherapy. TMC435 antiviral activity against HCV genotype 4 was comparable to that observed in HCV genotype 1 infection in early Phase 1/2a studies with TMC435 200 mg qd monotherapy.

Pharmacokinetic parameters for TMC435 in subjects infected with HCV genotype 4 were consistent with those in genotype 1 infected subjects[6].

A Phase 3 study in chronic HCV genotype 4 infected subjects is currently ongoing (HPC3011) and evaluates the efficacy, safety and tolerability of TMC435 (150 mg qd administered for 12 weeks) in combination with PegIFNα-2a/RBV, in treatment naïve and treatment-experienced subjects with HCV genotype 4 infection. Results from an interim analysis with cut-off date 17 January 2013 suggest that the efficacy of TMC435 in the HCV genotype 4 infected subjects is in line with what is observed in HCV genotype 1 infected subjects.

At the time of interim analysis, 107 subjects had received study treatment, of whom 35 were HCV treatment-naïve, 22 prior viral relapser, 10 prior partial responders and 40 prior null-responders. Of those 107 subjects, 86 (80.4%) had completed treatment with TMC435 and 20 (18.7%) subjects had completed the planned treatment period with PegIFN/RBV. The preliminary SVR12 rates in treatment-naïve subjects and prior relapsers with HCV genotype 4 in study HPC3011 were similar to rates observed in subjects infected with HCV genotype 1. SVR12 rates are not yet available for prior non-responders. Overall, 77.8% of treatment-naïve subjects or prior relapsers who completed treatment and reached the SVR12 assessment time point achieved SVR12: 3 of 3 evaluable treatment-naïve subjects and 4 of 6 evaluable prior relapsers.

A large majority of RGT-eligible subjects (i.e., treatment-naïve subjects and prior relapsers; all fibrosis stages including compensated cirrhosis) met the protocol-stipulated RGT criteria for shortening duration of PegIFN/RBV treatment (82.5%). In the subjects meeting the RGT criteria and reaching the SVR time point, the preliminary SVR12 rate was 87.5%[36].

Fifteen subjects experienced on-treatment failure, of whom 3 were HCV treatment-naïve, 2 prior viral relapser, and 10 prior null-responders. To date, no viral relapse was observed in this study.

Safety Data[18]

The available data of the Phase 3 study HPC3011 and final data of the Phase 2a study C202 suggest that the safety of TMC435 is comparable in the HCV genotype 4 and HCV genotype 1 infected subjects.

Results from an interim analysis with cut-off date 17 January 2013 of the ongoing HPC3011 study suggest that the safety and tolerability of TMC435 in the HCV genotype 4 infected subjects are in line with what is observed in HCV genotype 1 infected subjects. During the entire treatment phase up to the cut-off date of the interim analysis, most AEs were grade 1 or 2 in severity. Serious AEs were reported for 4 (3.7%) subjects. None of the AEs were fatal. Adverse events leading to treatment discontinuation of TMC435 were reported in one (1.1%) subject. The most common AEs by preferred term (in >25% of the subjects) were influenza-like illness, asthenia, and fatigue. There were no unexpected observations with regard to the frequency or severity of reported events of interest reported as AEs: increased bilirubin was observed in 2 subjects (1.9%), rash (any type) in 20 subjects (18.7%), and pruritus in 25 subjects (23.4%).

1.4. Combination Therapy

TMC435 will be administered in combination with PegIFNα-2a (Pegasys®) and RBV (Copegus®).

For further information regarding Pegasys and Copegus, refer to the Summary of Product Characteristics (SmPC)[9,38].

1.5. Benefits and Risk Assessment

This benefit and risk assessment is primarily based on clinical data generated in a HCV genotype 1 infected population as only limited clinical data in a HCV genotype 4 infected population are currently available. However, based on the consistency of in vitro activity and available viral response data, including early viral kinetics and SVR rates, between genotype 1 and genotype 4 as well as the comparable safety and tolerability profile in both genotypes, it is expected that benefits and risks will be comparable in subjects with genotype 1 and genotype 4.

1.5.1. Known Benefits

- TMC435 is an inhibitor of the HCV NS3/4A protease which is essential for viral replication. The in vitro anti-HCV EC_{50} and EC_{90} of TMC435 were 9.4 nM and 19 nM, respectively, in a genotype 1b replicon system.

- In HCV genotype 1 treatment-naïve subjects in Phase 3 studies C208 and C216, TMC435 150 mg once daily administered for 12 weeks with PegIFNα-2a/RBV or PegIFNα-2b/RBV (C216 only) for response-guided 24 or 48 weeks was superior (based on the primary endpoint, SVR12) to placebo with PegIFN/RBV alone for 48 weeks. Observed difference between the TMC435 and PegIFN/RBV alone treatment groups for these pooled studies was 30.4%.
- Significantly higher SVR rates with TMC435 in combination with PegIFN/RBV compared to PegIFN/RBV alone were observed across subgroups of demographic factors and baseline disease characteristics including sex, age, race, BMI, HCV genotype, baseline HCV RNA levels (≤800,000 IU/mL, ≥800,000 IU/mL), Metavir fibrosis score, and *IL28B* genotype (Phase 3 studies C208, C216, HPC3007).
- Study HPC3011 is currently ongoing and evaluates the efficacy, safety and tolerability of TMC435 (150 mg qd administered for 12 weeks), in combination with PegIFNα-2a/RBV, in treatment naïve and treatment-experienced subjects with HCV genotype 4 infection. Available interim results showed that TMC435 in combination with PegIFNα-2a/RBV was associated with high preliminary SVR12 rates in treatment-naïve subjects and prior relapsers that are comparable to those reported for the HCV genotype 1 population.

For more detailed information refer to the specific Clinical Study Reports.

1.5.2. Potential Benefits

For the subjects in this study, the status of their HCV infection and general health will be closely monitored. It is possible that by participating in this study the subject's general health and HCV disease status will improve. As the study investigates the possibility to shorten the treatment duration to 12 weeks triple therapy (PegIFNα-2a/RBV/TMC435), it is also possible that the subject may receive HCV treatment for a shorter total duration than current treatment guidelines indicate. Results from this study may be useful in developing a new therapy for HCV-infected subjects.

1.5.3. Known Risks

Every medication can have **undesirable effects**.

Adverse reactions of TMC435 were identified from the AEs reported in the placebo-controlled Phase 2b and 3 studies in which TMC435 was dosed in combination with PegIFN/RBV (C205, C206, C208, C216, HPC3007) since this is the largest dataset, in accordance with a systematic and well-documented structured approach with finalization date 11 February 2013. The adverse reactions assessment was performed for placebo-controlled studies because the AE rate is driven by PegIFN/RBV. The grouped terms identified as TMC435 adverse reactions are constipation, blood bilirubin increased, photosensitivity reaction, pruritus, and rash.

In the pooled data set from the 3 placebo-controlled Phase 3 studies (TMC435-C208, -C216, and HPC3007) at least 1 TMC435 adverse reaction was reported in 44.7% of TMC435-treated subjects and in 31.2% of subjects receiving placebo, during the first 12 weeks phase. Adverse reactions reported in TMC435-treated subjects and subjects receiving placebo were pruritus (21.9% vs 14.6%), rash (21.8% vs 16.6%), blood bilirubin increased (7.4% vs 2.8%),

photosensitivity reaction (4.7% vs 0.8%), and constipation (2.6% vs 2.5%). With exception of constipation, incidences were higher in the TMC435-treated subjects, but the differences between the treatment groups became smaller when considering the entire treatment phase. For constipation, the time to onset was shorter in the TMC435 group than in the placebo group

Most adverse reactions were grade 1 or 2 in severity. During the first 12 weeks phase, the incidence of grade 3 or 4 adverse reactions (2.8% in TMC435-treated subjects and 0.5% [2 subjects] in subjects on placebo), serious adverse reactions (0.3% [2 subjects] vs 0.0% [none]), and adverse reactions leading to TMC435/placebo discontinuation (0.9% [7 subjects] vs 0.3% [1 subject]) was low.

In conclusion, TMC435 150 mg once daily was generally safe and well tolerated when administered for a duration of 12 weeks in combination with PegIFN/RBV to adults with compensated liver disease with or without HIV co-infection.

1.5.4. Potential Risks

The following potential AEs or toxicities will be carefully monitored during the study and are specified in this protocol:

Reproductive Risks and Pregnancy:

No studies with TMC435 were performed in pregnant women. In animal studies, TMC435 had no effect on fertility and early embryonic and fetal development in rats up to doses of 500 mg/kg corresponding to an exposure of 221 µg.h/mL in plasma. No human data on the effect of TMC435 on fertility are available. Therefore, pregnancy and breastfeeding have been exclusion criteria for all clinical studies with TMC435 conducted to date. Women of childbearing potential and men included in studies with TMC435 have been required to use effective methods of birth control.

Significant teratogenic and/or embryocidal effects have been demonstrated in animals exposed to RBV. Ribavirin may persist in non-plasma compartments for as long as 6 months. Ribavirin is contraindicated in females who are pregnant or breastfeeding and in male partners of females who are pregnant. Ribavirin should not be started unless a report of a negative pregnancy test has been obtained immediately prior to initiation of therapy. In addition, extreme care must be taken to avoid pregnancy in females of childbearing potential or males having a female partner of childbearing potential taking RBV for at least 6 months after completion of PegIFNα-2a and RBV therapy (or longer, if dictated by local regulations). Subjects must consent to utilize 2 effective methods of birth control (as specified in Section 4.3), and male adolescents should not donate sperm while participating in this study and for at least 6 months after receiving the last dose of RBV (or longer, if dictated by local regulations; see Section 4.3 for more detail).

Drug Interactions:

TMC435 is mainly metabolized by CYP3A enzymes. Co-administration of TMC435 and drugs that induce CYP3A enzymes may decrease TMC435 plasma concentrations and reduce its therapeutic effect. Conversely, co-administration of TMC435 and drugs that inhibit CYP3A

enzymes may increase TMC435 plasma concentrations and increase or prolong its therapeutic and adverse effects.

The impact of TMC435 on drug metabolizing enzymes is limited to mild inhibition of intestinal (not hepatic) CYP3A and mild inhibition of CYP1A2. In addition, interaction potential with P-gp and OATP1B1 substrates has been identified.

An overview of disallowed concomitant medication is presented in Section 8 of this protocol.

Development of drug resistance:

Pooled data for the Phase 2b and 3 studies in subjects infected with genotype 1 HCV showed that treatment failure (not achieving SVR12 or relapsing thereafter) occurred in 245 of 1136 subjects who received TMC435 150 mg once daily in combination with PegIFN/RBV, of whom 197 subjects had NS3 sequence information available. The majority (91.4%, 180 of 197) of these subjects had emerging mutations at position 80, 122, 155 and/or 168 at time of failure that reduce susceptibility to TMC435 in vitro.

Follow-up of the subjects with emerging mutations at time of failure within in the Phase 2b and 3 studies showed that emerging resistant variants were no longer detectable by standard population sequencing in 50.0% (90/180 subjects) of subjects within the trial period (median follow-up time 28 weeks; range: 0 to 70 weeks).

The long-term persistence of emergent TMC435-resistant variants could potentially impair a subject's future treatment options with respect to retreatment with a HCV PI due to potential cross-resistance within this class. However, this potential risk may be mitigated by the availability of other antiviral agents with a different mechanism of action.

Continued exposure to TMC435 in subjects who experienced viral breakthrough and/or on-treatment failure could potentially lead to further accumulation of mutations and improvement of viral fitness. These factors could prolong the time that resistant variants persist in subjects and therefore potentially impair future treatment options. Implementation of virologic stopping rules (as described in Section 6.3) may minimize the accumulation of mutations.

At present, data on drug resistance in subjects infected with genotype 4 HCV are very limited.

1.5.5. Overall Benefit/Risk Assessment

Based on the available data and proposed safety measures, the overall risk/benefit assessment for this clinical study is acceptable for the following reasons:

- Safety data from Phase 1, 2, and 3 studies showed that TMC435 was generally safe and well tolerated in healthy and HCV-infected adults at all doses tested, including the 150 mg once daily dose that is currently used in the ongoing Phase 3 studies.
- Only subjects who meet all of the inclusion criteria and none of the exclusion criteria (as specified in the protocol) will be allowed to participate in this study. The selection criteria

include adequate provisions to minimize the risk and protect the well-being of subjects in the study.

- Safety will be closely monitored by the investigator throughout the study. Safety and tolerability assessments (including vital signs, physical examination, and clinical laboratory tests) will be performed at scheduled visits throughout the study. Adverse events will be monitored from signing of the informed consent onwards until 4 weeks after last study medication intake. Thereafter, only AEs considered related to TMC435 will be reported. SAEs will be reported until 30 days after last study-related procedure.
- Several safety measures have been proposed to minimize potential risk to subjects, including:
  - The safety monitoring and toxicity management plan in this study (see Section 9.5.7) takes into account the known toxicities of the PI class, the safety and tolerability profile of TMC435 as determined in studies in healthy subjects and in (ongoing) studies in HCV-infected subjects, the findings in nonclinical studies, and the safety and tolerability profile of PegIFNα-2a and RBV.
  - Subjects must discontinue all study medication (TMC435, PegIFNα-2a and RBV) for any of the following reasons:
    - withdrawal of consent.
    - The subject meets a virologic stopping criterion at Week 4 or 12 or experiences viral breakthrough at any time point during treatment (see Section 6.3).
    - The investigator believes that for safety reasons (e.g., AE) it is in the best interest of the subject to discontinue study treatment.
    - The subject becomes pregnant.
    - The subject fails to comply with the contraception requirements (refer to Section 4.3).
    - The subject experiences a grade 3 or 4 rash/allergic reaction (see Sections 9.5.7.1 and 9.5.7.2).
    - The subject experiences severe worsening of hepatic disease (see Section 9.5.7.4).
  - Subjects must discontinue investigational medication (TMC435) and may continue PegIFNα-2a and/or RBV alone if:
    - They require treatment with one of the medications included in the list of disallowed concomitant medications (see Section 8).
    - The subject experiences grade 4 ALT/AST elevations > 2 x baseline value and concurrent grade 4 bilirubin values, and the ALT/AST and bilirubin values continue to meet grade 4 criteria at re-testing performed 72 hours after receipt of the results (see Section 9.5.7.3). Subjects who have known Gilbert's disease and experience isolated grade 4 elevated indirect bilirubin can continue the investigational medication at the investigator's discretion.
    - The subject experiences a grade 3 or 4 AE (other than the rash/allergic reaction AE mentioned above) or confirmed grade 3 or 4 laboratory abnormality (other than grade 3 isolated increases in AST/ALT or bilirubin) considered to be at least

possibly related to TMC435 (2 consecutive measurements no more than 72 hours after receipt of first results). This excludes AEs or laboratory abnormalities that are not considered to be at least possibly related to TMC435.

- There will be a post-treatment follow-up of 24 weeks after end of treatment. All AEs occurring at any time during the study (including the follow-up period) will be followed until satisfactory resolution (e.g., value back to baseline value) or stabilization (to be agreed upon in collaboration with the sponsor).
- If a subject withdraws from the study (i.e., withdrawal of consent), he/she maintains the option to participate in the safety follow-up procedures.

1.6. Overall Rationale for the Study

Interferon-based HCV therapy is associated with a high side effect burden. Moreover it requires high adherence to dosing frequency as well as dose to obtain optimal response rates[33]. It therefore requires high patient motivation to initiate and remain adherent to treatment over its entire course. Research has therefore sought to identify patient groups that could benefit from shorter treatment duration without losing efficacy. Early response to treatment has been shown to reliably predict the likelihood of achieving SVR[13,15]. This has led to the concept of response guided therapy (RGT) that determines treatment duration and futility of treatment in the individual patient by assessing the response to treatment at defined time points[11].

Study C205[7] was designed to evaluate the safety and efficacy of TMC435 in previously untreated patients chronically infected with genotype 1 HCV (see also Section 1.5.1). A total of 156 subjects were assigned to receive TMC435 150 mg q.d. for either 12 weeks (77) or 24 weeks (79) of triple therapy with PegIFNα/RBV followed by PegIFNα/RBV alone. Subjects treated with TMC435 were eligible for shortening of total treatment to 24 weeks if their HCV RNA levels were < 25 IU/mL (detectable or undetectable) at Week 4 and were undetectable at Weeks 12, 16, and 20. Among subjects treated with 150 mg TMC435, 79.2% in the 12-week TMC435 treatment arm and 86.1% in the 24-week TMC435 treatment arm met the RGT criteria and completed therapy after a total treatment duration of 24 weeks. Of these, 93.4% and 95.6% of patients achieved SVR24[14]. These very high response rates support the question whether treatment could be shortened even further, i.e., to 12 weeks to avoid side effects and costs associated with longer treatment.

There is an emerging body of evidence that in selected patient groups demonstrating very fast on-treatment response, 12 weeks of treatment with a combination of P/R and direct-acting antivirals will result in very high response rates. Two recent studies compared 12 with 24 weeks of such combinations in patients with fast viral response and found no significant difference in response rates[32, 37]. CONCISE included previously untreated or relapsed patients with GT1 HCV, with no to severe liver fibrosis (F0-3) and the IL28B CC genotype. Patients that had undetectable viral load after 4 weeks of triple therapy with telaprevir + P/R were randomized to either stop all treatment after 12 weeks or to extend treatment with P/R to 24 weeks. Very high cure rates were seen in both arms (SVR12: 87% vs 97%) with no statistical significant difference between arms[37]. Another study included treatment-naïve HCV GT1 patients with no to severe liver fibrosis regardless of IL28B genotype. Patients were treated with a combination of 2 DAAs

and P/R. Patients with HCV RNA below the lower limit of detection (LLOD; 25 IU/ml) at Weeks 2 and 4 of treatment and undetected HCV RNA at Week 8 were randomized to stop all treatment at Week 12 or extend treatment to 24 weeks. Both arms showed very high response rates that did not significantly differ from each other (SVR12: 86% vs 92%)[32]. The single-arm study NEUTRINO investigated the safety and efficacy of a fixed treatment duration of 12 weeks of triple therapy with the polymerase inhibitor sofosbuvir in combination with PegIFN/RBV in patients with HCV genotype 1, 4, 5, or 6. SVR12 rates in genotype 1 and 4 were 89% and 96%, respectively[29]. Patients responded very fast to therapy with 91% of patients having <25 IU/ml at treatment week 2. This observation further underscores the association between rapid viral response and the ability to shorten treatment without compromising efficacy and cure rates.

The current study will investigate whether 12 weeks of TMC435-based triple therapy will be sufficient to achieve SVR in genotype 1 or genotype 4 HCV-infected patients. Eligibility for shortened treatment duration is based on early viral response at Week 2 and undetectable HCV RNA at Weeks 4 and 8. The study will include patients with different *IL28B* genotypes but will exclude patients with advanced fibrosis (equivalent to F3 or F4). While no data yet exists for 12-week TMC435-based triple therapy, the eligibility criteria for shortening of treatment have been selected to ensure a high probability of SVR. A multivariate analysis of prognostic factors on SVR24 in C205[7] that included baseline demographic and viral parameters as well as early viral response parameters during treatment, found only smoking, extended RVR, and complete early virologic response to be significantly associated with SVR. This indicates that early viral response under treatment is a stronger predictor of SVR than baseline parameters. It has also been shown that RVR overcomes the effect of *IL28B* genotype on SVR in genotype 1 HCV-infected patients[43].

It is expected that in the current study a large majority of patients will be eligible for a 12-week course of TMC435-based treatment. In C205[7], TMC435 reduced the impact of *IL28B* genotype on viral response and high RVR rates were observed. In the combined 150 mg TMC435 groups, 77.1%, 80.0%, and 91.7% of patients with CC, CT, and TT genotypes, respectively, achieved RVR[1]. In contrast, the corresponding values in the placebo group were 8.3%, 3.6%, and 0%.

Measures will be implemented to ensure that the recruited patient sample will reflect the population in the ongoing Phase 3 studies. Thus, there are upper limits for the 3 *IL28B* genotypes CC, CT, and TT, as well as for the genotype 1 HCV subtype 1a. Patients with advanced liver fibrosis (equivalent to F3-F4) will be excluded from the study. In the combined 150 mg TMC435 treatment groups in C205[7], more patients with fibrosis stage Metavir F0-F2 achieved RVR than patients with F3 (75.7% versus 70.0%). Given the low likelihood that patients with fibrosis stage equivalent to Metavir F3 or F4 will be eligible for shorter treatment durations and to reduce the risk for relapse in these patients, it has been decided not to include this population in this study specifically investigating the possibility for a 12-week treatment duration.

HCV genotype 4 and genotype 1 show similar response rates to treatment with interferon and ribavirin. Thus, international treatment guidelines[11] recommend identical treatment algorithms for genotype 1 and 4. Current standard treatment of chronic HCV genotype 4 infection consists of 48 weeks of PegIFN/RBV. In both genotypes rapid viral response to PegIFN/RBV has been

shown to be associated with higher SVR rates and allows shortening of treatment duration to 24 weeks[23,43]. In the NEUTRINO study, 12 weeks of triple therapy with sofosbuvir plus PegIFN/RBV resulted in equally high SVR rates in genotype 1 (89%) and genotype 4 (96%)[29]. In an exploratory study[6], TMC435 has been shown to have antiviral activity in genotype 4 infected subjects comparable to that in genotype 1 infected subjects, when administered as 200 mg qd monotherapy for 7 days. Results from an interim analysis of the ongoing HPC3011 study suggest that the efficacy and safety of TMC435 in the 107 HCV genotype 4 infected subjects who had received study treatment at the time of interim analysis is in line with what is observed in HCV genotype 1 infected subjects, when administered as 150 mg qd for 12 weeks. Of note, a large majority of RGT-eligible subjects (82.5%) in HPC3011 (treatment-naïve and prior relapsers; all stages of fibrosis including compensated cirrhosis) met the protocol-defined criteria for shortening of treatment duration. This suggests that TMC435 in combination with PegIFN/RBV has the potential to increase SVR rates and shorten overall treatment duration in genotype 4 infected subjects as has been shown for genotype 1 (see Section 1.3). In order to further increase the available efficacy and safety data with TMC435 in HCV genotype 4 infected subjects, the current open-label study will evaluate the efficacy, safety and tolerability of TMC435 in combination with PegIFNα-2a and RBV in chronic HCV genotype 4 infected subjects using a response-guided treatment duration as specified (See Section 6.2). Similar to the genotype 1 subjects included in this trial, only genotype 4 subjects with mild to moderate fibrosis will be included. Patients with low fibrosis have a better chance for faster viral clearance. The results in genotype 4 will be important from a global perspective as the majority of patients with HCV genotype 4 live in resource-limited countries. The ability to shorten treatment from 48 or 24 weeks to 12 weeks would thus address a high unmet need in these countries and allow more patients to be treated.

2. OBJECTIVES AND HYPOTHESIS

2.1. Objectives

Primary Objectives

The primary objectives are:

- To determine the efficacy of TMC435 plus PegIFNα-2a and RBV when administered for 12 weeks in treatment-naïve subjects with chronic genotype 1 HCV infection, as measured by the proportion of subjects with sustained virologic response 12 weeks after planned end of treatment (SVR12).
- To assess the safety and tolerability of TMC435 plus PegIFNα-2a and RBV when administered for 12 weeks in treatment-naïve subjects with chronic genotype 1 HCV infection

Major Secondary Objectives

Major secondary objectives are:

- To determine the efficacy of TMC435 plus PegIFNα-2a and RBV when administered for 12 weeks in treatment-naïve subjects with chronic genotype 4 HCV infection, as measured

by the proportion of subjects with sustained virologic response 12 weeks after planned end of treatment (SVR12).

- To assess the safety and tolerability of TMC435 plus PegIFNα-2a and RBV when administered for 12 weeks in treatment-naïve subjects with chronic genotype 4 HCV infection

Further major secondary objectives are in subjects with genotype 1 or genotype 4 HCV infection (separately per genotype):

- To determine the proportion of subjects who achieve virologic response at Week 2 (W2VR) and the proportion of subjects who achieve RVR.
- To determine the relationship between W2VR and SVR12 and between RVR and SVR12.
- To determine the efficacy of TMC435 plus PegIFNα-2a and RBV for 12 weeks followed by 12 weeks of PegIFNα-2a and RBV (i.e., a total treatment duration of 24 weeks), as measured by the proportion of subjects with SVR12.
- To determine the efficacy of TMC435 plus PegIFNα-2a and RBV after a total treatment duration of 12 weeks, as measured by the proportion of subjects with sustained virologic response 24 weeks after planned end of treatment (SVR24).
- To determine the efficacy of TMC435 plus PegIFNα-2a and RBV for 12 weeks followed by 12 weeks of PegIFNα-2a and RBV (i.e., a total treatment duration of 24 weeks), as measured by the proportion of subjects with SVR24.
- To evaluate the evolution of HCV RNA levels at regular intervals during treatment and after planned end of treatment.

and for both genotypes combined:

- To evaluate impact of HCV or its treatment on patient reported symptoms and functioning using a new patient reported outcomes tool, the Hepatitis C Symptom & Impact Questionnaire (HCV-SIQ) and four well-validated PRO instruments measuring severity and impact of fatigue (Fatigue Severity Scale, FSS), depressive symptoms (Center for Epidemiologic Studies Depression Scale, CES-D), time missed from work and impairment in daily activities (Work Productivity and Activity Impairment, WPAI: Hepatitis C), and health status (EuroQol 5 Dimension, EQ5D).

Definitions

Sustained virologic response 12 weeks after planned end of treatment (SVR12) will be defined as undetectable HCV RNA (< 25 IU/mL undetectable) at the actual end of treatment and 12 weeks after the planned end of treatment.

Rapid virologic response (RVR) will be defined as HCV RNA < 25 IU/mL undetectable measured 4 weeks after start of treatment.

Virologic response at Week 2 (W2VR) will be defined as HCV RNA < 25 IU/mL (detectable or undetectable) measured 2 weeks after start of treatment.

Exploratory Objectives

The exploratory objectives in subjects with genotype 1 or genotype 4 HCV infection are:

- to evaluate the measurement properties of the HCV-SIQ
- to explore alternate scoring of the HCV-SIQ (e.g., total score, symptom scores by body system).

2.2. Hypothesis

In this study, the response rate (SVR12) in subjects with genotype 1 HCV infection eligible for and assigned to 12 weeks of treatment is expected to be superior to the minimally acceptable response rate of 80%.

3. STUDY DESIGN AND RATIONALE

3.1. Overview of Study Design

This, multicenter, international study will evaluate the efficacy, tolerability, and safety of 12-week triple therapy with TMC435 plus PegIFNα-2a and RBV in treatment-naïve adult subjects with genotype 1 or genotype 4 chronic HCV infection and fibrosis stage equivalent to F0-F2. A target of 150 subjects with genotype 1 chronic HCV infection and 75 subjects with genotype 4 chronic HCV infection will be assigned to treatment in this study.

In an attempt to enroll similar percentages of the 3 host *IL28B* genotypes (CC, CT or TT) as were enrolled in the ongoing Phase 3 TMC435 studies, the sponsor will make all effort to respect the following maximum caps for enrolment of each *IL28B* genotype for subjects with genotype 1 or genotype 4 HCV infection separately:

- a maximum of 35% of subjects with host *IL28B* genotype CC (i.e., maximum 35% *IL28B* CC among subjects with genotype 1 HCV infection and maximum 35% *IL28B* CC among subjects with genotype 4 HCV infection);
- a maximum of 55% of subjects with host *IL28B* genotype CT (i.e., maximum 55% *IL28B* CT among subjects with genotype 1 HCV infection and maximum 55% *IL28B* CT among subjects with genotype 4 HCV infection);
- a maximum of 18% of subjects with host *IL28B* genotype TT (i.e., maximum 18% *IL28B* TT among subjects with genotype 1 HCV infection and maximum 18% *IL28B* TT among subjects with genotype 4 HCV infection).

In addition, within subjects infected with genotype 1 HCV, the sponsor seeks a balance between HCV genotypes 1a and 1b, therefore all effort will be made to cap enrolment of HCV genotype 1a at:

- a maximum of 50% of subjects infected with HCV genotype 1a.

Procedures to manage the distribution of the HCV genotype 1 subtypes and the host *IL28B* subtypes will be communicated to participating investigators at study initiation.

All subjects will start treatment at baseline (Day 1) and will receive triple therapy consisting of TMC435 plus PegIFNα-2a and RBV for 12 weeks. Total anti-HCV treatment duration will be response-guided based on HCV RNA levels at Week 2, Week 4, and Week 8 as follows:

- Subjects will qualify for a total treatment duration of only 12 weeks (ie all anti-HCV treatment is discontinued after week 12) if the following criteria are met: if HCV RNA value is
    - < 25 IU/mL (detectable or undetectable) at Week 2,

    AND
    - < 25 IU/mL undetectable at Week 4,

    AND
    - < 25 IU/mL undetectable at Week 8.
- if HCV RNA is above the threshold defined above at any of the 3 time points, subjects will continue PegIFNα-2a and RBV until Week 24.

A diagram of the study design is provided in Figure 1.

Regardless of treatment duration, endpoint to evaluate efficacy will be SVR12.

The treatment continuation criteria will at all times be overruled if a subject meets any of the virologic stopping criteria outlined below. Subjects meeting any of the following criteria will be required to discontinue all treatment:

- Week 4: HCV RNA ≥ 25 IU/mL
- Week 12: HCV RNA ≥ 25 IU/mL OR < 25 IU/mL detectable
- a confirmed increase of > 1 $\log_{10}$ IU/mL in HCV RNA level from the lowest level reached, or a confirmed HCV RNA level of > 100 IU/mL in subjects whose HCV RNA level had previously been < 25 IU/mL while on study drugs.

Detectable HCV RNA after previous undetectability should be confirmed by a confirmatory HCV RNA measurement within 2 weeks after receipt of the detectable HCV RNA value. Treatment should only be discontinued after HCV RNA detectability is confirmed (see Section 6.3, Virologic Stopping Criteria for further details).

HCV RNA will be measured centrally and the results will be communicated to the investigational sites. It will be the investigator’s responsibility to take the necessary actions regarding the discontinuation of therapy in case a virologic stopping rule has been met.

Figure 1: Schematic Overview of the Study

All subjects: 12 weeks of triple therapy[a]; stop all therapy after Week 12 if
HCV RNA < 25 IU/mL (detectable or undetectable) at Week 2 AND
< 25 IU/mL undetectable at Week 4 AND
< 25 IU/mL undetectable at Week 8;
otherwise continue treatment in Extension

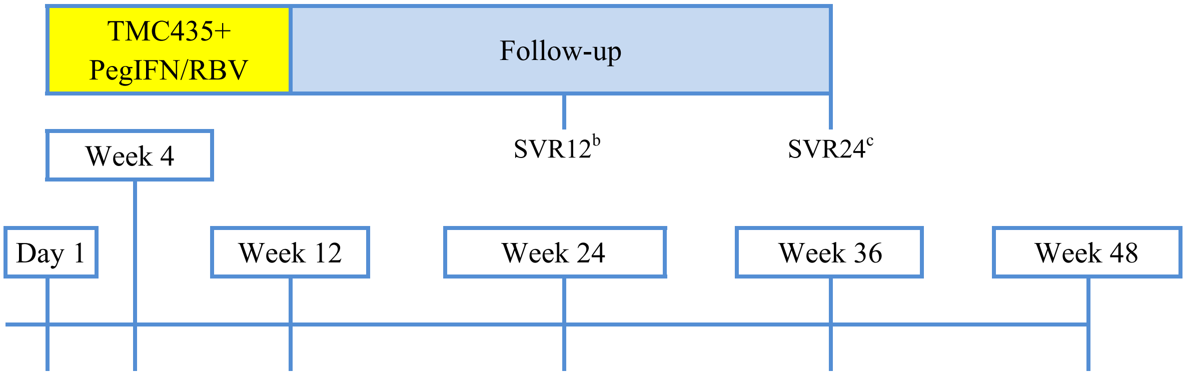

Treatment Extension
If HCV RNA at Week 2, 4 and 8 does not meet the above-outlined criteria for treatment stop at Week 12, the subject should continue PegIFN/RBV until Week 24[a]

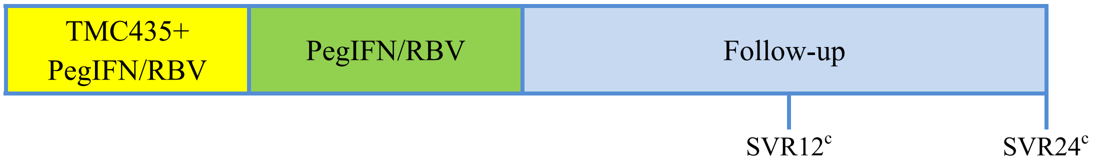

a unless a virologic stopping rule is met, see Section 6.3, Virologic Stopping Criteria for further details
b primary endpoint
c secondary endpoint

TMC435 will be taken as oral capsules of 150 mg. PegIFNα-2a and RBV will be given as Pegasys and Copegus (see Section 6.2, Timing of Dosing for further details). Pegasys and Copegus will be administered according to the manufacturer's prescribing information. Pegasys (180 µg once weekly) will be administered as weekly subcutaneous (SC) injections of 0.5 mL. The total daily dose of Copegus is weight-dependent. If body weight is < 75 kg, the total daily dose is 1000 mg, administered as 400 mg (2 tablets of 200 mg, morning intake) and 600 mg (3 tablets of 200 mg, evening intake). If body weight is ≥ 75 kg, the total daily dose is 1200 mg, administered as 2 x 600 mg (3 tablets of 200 mg per intake, morning and evening).

The study will be conducted in 3 phases: a screening phase of maximum 6 weeks, a treatment phase extending from Day 1 (baseline) up to 12 or 24 weeks depending on the response to treatment, and a posttreatment follow-up period of 24 weeks after the subject's last planned dose of study drug. The duration of the subject's participation (excluding screening phase) will vary between 36 weeks and 48 weeks, depending on the response to treatment.

Subjects should maintain the study visit schedule during treatment interruptions or after premature treatment discontinuation as outlined in the Time and Events Schedule. Additional unscheduled visits may be performed for safety or tolerability reasons if needed.

Interim analyses will be conducted (refer to Section 11.5, Interim Analyses).

The key efficacy evaluation will be plasma HCV RNA levels (see Section 9.2.1, Evaluations). Safety evaluations will include the monitoring of AEs, physical examinations, vital sign measurements, clinical laboratory tests, and pregnancy testing. Patient-reported outcomes (PROs) will also be assessed.

A pharmacogenomic blood sample will be collected to allow for host *IL28B* genotyping. This pharmacogenomic blood sample is mandatory and will be collected from all subjects who consent to participate in the study.

3.2. Study Design Rationale

Blinding, Control, Study Phase/Periods, Treatment Groups

This is a study without an active or placebo control. No randomization or blinding procedures are applicable.

Patient Reported Symptoms and their Impact

Severity and impact of symptoms before, during and after treatment provide important information for patients and physicians about the tolerability of treatments beyond what is known from AE reports. Prospectively collecting information about symptoms and their impact gives physicians better information to advise patients about what to expect during treatment and how long any symptoms or functional problems that may occur during treatment are likely to last. Patients are better able to anticipate and persist with treatment when symptoms occur if information about duration, severity and impact of symptoms from clinical studies can be provided.

Adding TMC435 to PegIFNα-2a/RBV has not been found to increase symptoms or functional limitations beyond what patients experience with PegIFNα-2a and RBV[16]. TMC435 Phase 2 and Phase 3 studies used PRO measures to study two of the most common symptoms associated with PegIFNα-2a/RBV treatment: fatigue and depressive symptoms. Data from the PRO measures documented the duration and intensity of symptoms subjects experienced based on their own ratings throughout treatment and follow-up. The prospective collection of the PRO symptom data provided valuable information beyond what was known based on AE frequency and severity reports.

For this study, 5 PRO measures will be administered during study visits to document quality of life and symptom onset, duration, severity, and impact from the subject's perspective. The HCV-SIQ asks subjects to rate 26 symptoms associated with HCV or its treatment and how symptoms impacted the subjects' life during the prior week. This questionnaire provides a simple tool for monitoring symptoms during HCV treatment and follow-up that may be suitable for clinical

management of patients as well as for clinical research. To help evaluate the validity of the HCV-SIQ, 4 PRO questionnaires that have been used extensively in prior HCV studies will also be collected. The Fatigue Severity Scale (FSS) will be used to document fatigue severity and impact of fatigue on subjects' daily lives. The Center for Epidemiologic Studies Depression Scale (CES-D), a brief assessment that asks subjects to rate how often in the past week they experienced 20 symptoms associated with depressive illness, will be used to assess depressive symptom severity. The Work Productivity and Activity Index for Hepatitis C (WPAI: Hepatitis C) will be used to measure the impact of HCV on time missed from work (absenteeism), reduced performance while at work (productivity impairment), and impairment in daily activities without regard to employment status. Finally, the EuroQol 5 Dimension (EQ5D) scale and overall health status will be used to measure the impact of HCV and its treatment on health-related quality of life (HRQoL). Together, the PRO instruments collected in this study are included to help better characterize the symptoms patients experience with HCV treatment, the impact symptoms have on patient's lives, and to evaluate the validity and reliability of HCV-SIQ for use in future HCV studies.

4. SUBJECT POPULATION

Screening for eligible subjects will be performed within 6 weeks before administration of the study drug.

The inclusion and exclusion criteria for enrolling subjects in this study are described in the following 2 subsections. If there is a question about the inclusion or exclusion criteria below, the investigator should consult with the appropriate sponsor representative before enrolling a subject in the study.

For a discussion of the statistical considerations of subject selection, refer to Section 11.2, Sample Size Determination.

4.1. Inclusion Criteria

Each potential subject must satisfy all of the following criteria to be enrolled in the study. Exceptional and limited retesting of abnormal screening values that lead to exclusion is allowed only once using an unscheduled visit during the screening period (to reassess eligibility). This should only be considered if there is no anticipated impact on subject safety. Refer to Section 9.1.2 for full details.

1. Subject must be a man or woman 18 to 70 years of age, inclusive, prior to the baseline visit.

2. Subject must have confirmed chronic HCV infection as documented by a positive anti-HCV antibody test, or positive HCV RNA test at least 6 months prior to screening, or positive HCV genotyping test at least 6 months prior to screening.

3. Subject's liver disease stage must be equivalent to Metavir Score F0-F2 (no fibrosis, or portal fibrosis without or with few septa) as confirmed by a liver biopsy performed within 2 years prior to screening or by non-invasive determination of liver disease stage (by transient elastography) performed within 6 months prior to screening. Non-invasive staging assessments include FibroScan™ or MR-Elastography.

   Note: The Metavir scoring system is provided in Attachment 1. For non-invasive fibrosis assessment, FibroScan™ should be < 8 kPa and MR-Elastography should be < 3.1 kPa. For any other transient elastography method (eg, Shear Wave Elastography), please contact the sponsor for advice.

4. Subject with liver disease stage equivalent to Metavir Score F2 and without liver biopsy performed within 2 years prior to screening must have undergone liver imaging (ultrasound, computed tomography [CT], or magnetic resonance imaging [MRI]) within 6 months prior to the Screening visit (or between the Screening and Baseline/Day 1 visits) with no findings suspicious of hepatocellular carcinoma.

5. Subject must have genotype 1 or genotype 4 HCV infection (confirmed at screening).

6. Subject's plasma HCV RNA must be > 10000 IU/mL at screening.

   Note: Retesting of HCV RNA to assess eligibility will be allowed once, using an unscheduled visit during the screening period.

7. Subject has not received prior treatment for hepatitis C with any approved or investigational drug.

8. Before screening, a woman must be either:

   - Not of childbearing potential: postmenopausal (> 45 years of age with amenorrhea for at least 12 months or any age with amenorrhea for at least 6 months and a serum follicle stimulating hormone (FSH) level > 40 IU/mL); permanently sterilized (e.g., tubal occlusion, hysterectomy, bilateral salpingectomy); or otherwise be incapable of pregnancy,

   - Of childbearing potential and practicing two different highly effective methods of birth control (including at least one barrier method) consistent with local regulations regarding the use of birth control methods for subjects participating in clinical studies: e.g., established use of oral, injected or implanted hormonal methods of contraception; placement of an intrauterine device (IUD) or intrauterine system (IUS); barrier methods: condom with spermicidal foam/gel/film/cream/suppository or occlusive cap (diaphragm or cervical/vault caps) with spermicidal foam/gel/film/cream/suppository; male partner sterilization (the vasectomized partner should be the sole partner for that subject); true abstinence (when this is in line with the preferred and usual lifestyle of the subject).

     Note: If the childbearing potential changes after start of the study (e.g., woman who is not heterosexually active becomes active, premenarchal woman experiences menarche) a woman must begin 2 highly effective methods of birth control, as described above.

9. A woman must have a negative serum β-human chorionic gonadotropin (β-hCG) test at screening and women of childbearing potential must have a negative urine pregnancy test before first dose of study drug.

10. A man who is sexually active with a woman of childbearing potential and has not had a vasectomy must agree to use two different highly effective methods of birth control (including at least one barrier method). A man who is sexually active with a woman of childbearing potential and has had a vasectomy must agree to use a barrier method of birth control e.g., either condom with spermicidal foam/gel/film/cream/suppository or partner with occlusive cap (diaphragm or cervical/vault caps) with spermicidal foam/gel/film/cream/suppository. All men must also not donate sperm during the study and for 6 months (or longer if dictated by local regulations) after the last dose of RBV.

11. Subject must be willing and able to adhere to the prohibitions and restrictions specified in this protocol.

12. Each subject must sign an informed consent form (ICF) indicating that he or she understands the purpose of and procedures required for the study and are willing to participate in the study.

13. Subject with genotype 4 HCV infection must have a body mass index (BMI) of $\leq$32 kg/m^2.

4.2. Exclusion Criteria

Any potential subject who meets any of the following criteria will be excluded from participating in the study.

1. Subject has been previously exposed to anti-HCV treatment, including any approved or investigational DAA therapy.

2. Subject has chronic liver disease of a non-HCV etiology (including but not limited to hemochromatosis, Wilson's disease, α1-antitrypsin deficiency, cholangitis, drug- or alcohol-related liver disease, primary biliary cirrhosis).

   Note: Subjects with Gilbert's disease will be allowed to participate provided they satisfy criteria outlined in exclusion criterion 8.

3. Subject is infected with hepatitis B virus (hepatitis B surface antigen [HBsAg] positive) or human immunodeficiency virus (HIV-1 or HIV-2) (HIV-1 or HIV-2 antibody test positive at screening).

4. Subject is infected with non-genotype 1 or non-genotype 4 HCV.

5. Subject has advanced liver disease equivalent to Metavir status F3-F4 (bridging fibrosis

or cirrhosis).

6. Subject has current or past history of clinical hepatic decompensation (e.g., ascites, variceal hemorrhage, hepatic encephalopathy, hepatorenal syndrome, or hepatopulmonary syndrome).

7. Subject has contraindications to the use of PegIFNα-2a or RBV per local prescribing information:

   – Major uncontrolled depressive illness.
   – Organ transplant (other than cornea or hair transplant or skin graft).
   – Severe concurrent medical disease including but not limited to severe hypertension, significant coronary heart disease requiring therapy, poorly controlled diabetes, untreated thyroid disease, chronic obstructive pulmonary disease, severe retinopathy or clinically relevant ophthalmological disorder, severe infections (bacterial, viral, fungal, including acute tuberculosis and active schistosomiasis), or hemoglobinopathies (thalassemia major or sickle-cell anemia).
   – Autoimmune hepatitis or other autoimmune conditions (except thyroid disease) known to be exacerbated by PegIFNα-2a and RBV.
   – Subject has evidence of severe retinopathy or clinically relevant ophthalmological disorder.
   – Subject has any other clinically significant disease that in the opinion of the investigator would be exacerbated by the known effects of PegIFNα-2a and RBV.

   Note: This is a non-exhaustive list. For details, refer to the manufacturer's prescribing information or contact the sponsor. Baseline fundoscopic pathology which would require routine follow up on PegIFNα-2a and RBV therapy is considered exclusionary.

8. Subject has any of the following laboratory abnormalities:

   – Platelet count < lower limit of laboratory normal range (LLN);
   – Absolute neutrophil count (ANC) < 1500 cells/mm^3 (subject of black African [Sub-Saharan African] descent: < 1000 cells/mm^3);
   – Hemoglobin < 12 g/dL for women and < 13 g/dL for men;
   – Creatinine > 1.5 mg/dL;
   – ALT and/or AST > 10 x upper limit of laboratory normal range (ULN);
   – Total serum bilirubin > 2 x ULN;

     Note: Subjects with total serum bilirubin > 2 x ULN are allowed in case of elevated indirect bilirubin due to Gilbert's disease and direct serum bilirubin below ULN.
   – Alpha-fetoprotein (AFP) > 50 ng/mL;

- Thyroid-stimulating hormone (TSH):
  - < 1 LLN or > 1.5 x ULN, or
  - ≥1 and ≤ 1.5 x ULN if free thyroxine (T_4) or free triiodothyronine (T_3) are outside of normal range;
- Antinuclear antibody (ANA) > 1:160.

9. Subject has a history of clinically significant renal, cardiac, vascular, pulmonary, gastrointestinal, endocrine, neurologic, hematologic, rheumatologic, psychiatric, or metabolic disturbances or any other major medical disorder that may interfere with the subject's treatment, assessment or compliance with the protocol.

10. Subject has a history of malignancy within 5 years before screening (exceptions are squamous and non-invasive basal cell carcinomas of the skin and carcinoma in situ of the cervix, or malignancy that in the opinion of the investigator, with concurrence with the sponsor's medical monitor, is considered cured with minimal risk of recurrence). Subjects under evaluation for malignancy are not eligible.

11. Subject has known allergies, hypersensitivity, or intolerance to TMC435 , PegIFNα-2a or RBV or its excipients (refer to IB or the respective SmPCs)[18,9,38].

12. Subject has taken any disallowed therapies as noted in Section 8, Prestudy and Concomitant Therapy before the planned first dose of study drug.

13. Subject is a woman who is pregnant, or breast-feeding, or planning to become pregnant while enrolled in this study or within 6 months after the last dose of RBV or a man who plans to father a child while enrolled in this study or within 6 months after the last dose of RBV (or longer when dictated by local regulations), or has a pregnant female partner.

14. Subject has a history of drug or alcohol abuse that in the investigator's opinion would compromise the subject's safety and/or compliance with the study procedures.

    Note: Urine will be tested at screening to check the current use of amphetamines, cocaine, opioids, benzodiazepines, cannabinoids and barbiturates. Subjects with a positive drug test may be included at the investigator's discretion. Evidence of the investigator's assessment with regard to the subject's safety and compliance must be in place prior to the start of treatment.

15. Subject has any condition for which, in the opinion of the investigator, participation would not be in the best interest of the subject (e.g., compromise the well-being) or that could prevent, limit, or confound the protocol-specified assessments. Vulnerable subjects (e.g., imprisoned subjects) should be excluded.

16. Subject is an employee of the investigator or study site, with direct involvement in the proposed study or other studies under the direction of that investigator or study site, as

well as family members of the employees or the investigator.

17. Subject with genotype 4 HCV infection has currently active schistosomiasis (i.e., positive Ag blood test or positive rectal snip). Subjects with a history of schistosomiasis may be included at the discretion of the Investigator.

18. Subject is infected with genotype 1 **and** genotype 4 HCV.

NOTE: Investigators should ensure that all study enrollment criteria have been met at screening, prior to declaring a subject eligible to start treatment. If a subject's status changes (including laboratory results or receipt of additional medical records) after screening but before the first dose of study drug is given such that they no longer meet all eligibility criteria, they should be excluded from participation in the study. Section 17.4, Source Documentation, describes the required documentation to support meeting the enrollment criteria.

4.3. Prohibitions and Restrictions

Potential subjects must be willing and able to adhere to the following prohibitions and restrictions during the course of the study to be eligible for participation.

Significant teratogenic and/or embryocidal effects have been shown in animals exposed to RBV. Ribavirin may persist in non-plasma compartments for as long as 6 months. It is contraindicated in women who are pregnant or breastfeeding and in male partners of women who are pregnant. In addition, extreme care must be taken to avoid pregnancy in female subjects and in female partners of male subjects taking RBV for at least 6 months after completion of PegIFNα-2a/RBV therapy (or longer, if dictated by local regulations). Ribavirin should not be started unless a report of a negative pregnancy test is obtained immediately prior to initiation of therapy. Therefore, the following precautions must be adhered to:

1. A woman of childbearing potential who is heterosexually active must remain on two different highly effective methods of birth control (see inclusion criteria in Section 4.1) from screening and up to 6 months after the last dose of RBV (or longer if dictated by local regulations).

2. A woman using oral contraceptives must use an additional birth control method (see inclusion criterion in Section 4.1).

3. A man who is sexually active with a woman of childbearing potential and has not had a vasectomy must agree to use two different highly effective methods of birth control (including at least one barrier method). A man who is sexually active with a woman of childbearing potential and has had a vasectomy must agree to use a barrier method of birth control e.g., either condom with spermicidal foam/gel/film/cream/suppository or partner with occlusive cap (diaphragm or cervical/vault caps) with spermicidal foam/gel/film/cream/suppository. All men must also not donate sperm during the study and for 6 months (or longer if dictated by local regulations) after the last dose of RBV.

For male subjects, it is the responsibility of the subject to ensure that his partner (or partners) is (are) not pregnant prior to entry into the study or becomes pregnant during the treatment and post-therapy follow-up phase of the study. During treatment with RBV, female partners of male participants should follow the guidelines as outlined in the manufacturer's prescribing information for RBV.

4. Subjects should be informed that during TMC435 administration photosensitivity reactions (rash confined to sun-exposed areas) have been reported and should be informed on the importance of sun protection during TMC435 treatment. Extreme exposure to the sun or sunbathing should be avoided, as well as the use of tanning devices (e.g., sunbed, solarium) from baseline until the last intake of TMC435.

5. TREATMENT ALLOCATION AND BLINDING

Randomization will not be used in this study. All subjects will be assigned to the same treatment. Duration of treatment will be response-guided (see Section 3.1).

In an attempt to enroll similar percentages of the 3 host *IL28B* genotypes (CC, CT or TT) as were enrolled in the ongoing TMC435 Phase 3 studies, the sponsor will make all effort to respect the following maximum caps for enrolment of each *IL28B* genotype for subjects with genotype 1 or genotype 4 HCV infection separately:

- a maximum of 35% of subjects with host *IL28B* genotype CC (i.e., maximum 35% *IL28B* CC among subjects with genotype 1 HCV infection and maximum 35% *IL28B* CC among subjects with genotype 4 HCV infection);
- a maximum of 55% of subjects with host *IL28B* genotype CT (i.e., maximum 55% *IL28B* CT among subjects with genotype 1 HCV infection and maximum 55% *IL28B* CT among subjects with genotype 4 HCV infection);
- a maximum of 18% of subjects with host *IL28B* genotype TT (i.e., maximum 18% *IL28B* TT among subjects with genotype 1 HCV infection and maximum 18% *IL28B* TT among subjects with genotype 4 HCV infection).

In addition, within subjects infected with genotype 1 HCV, the sponsor seeks a balance between HCV genotypes 1a and 1b, therefore all effort will be made to cap enrolment of HCV genotype 1a at:

- a maximum of 50% of subjects infected with HCV genotype 1a.

Procedures to manage the distribution of the HCV genotype 1 subtypes and the *IL28B* subtypes will be communicated to the investigators at study initiation.

As this is an open-label study, blinding procedures are not applicable.

6. DOSAGE AND ADMINISTRATION

6.1. Treatment Overview and Response-Guided Treatment Duration

All subjects will receive triple therapy consisting of TMC435 plus PegIFNα-2a and RBV for 12 weeks. Total anti-HCV treatment duration will be response-guided based on HCV RNA levels at Week 2, Week 4, and Week 8 as follows:

- Subjects will qualify for a total treatment duration of only 12 weeks (ie all anti-HCV treatment is discontinued after week 12) if the following criteria are met: HCV RNA value is
    - < 25 IU/mL (detectable or undetectable) at Week 2,

    AND
    - < 25 IU/mL undetectable at Week 4,

    AND
    - < 25 IU/mL undetectable at Week 8.
- if HCV RNA is above the threshold defined above at any of the 3 time points, subjects will continue PegIFNα-2a and RBV until Week 24.

The above treatment continuation criteria will at all times be overruled if a subject meets any of the virologic stopping criteria outlined in Section 6.3.

Table 1 provides an overview of treatment dosage.

The HCV RNA samples will be processed in real-time and results will be communicated to the investigators and sponsor throughout the study.

The investigator will make treatment decisions in line with these treatment duration criteria and virologic stopping criteria (as mentioned in Section 6.3).

Table 1: Treatment Overview

Subjects and timing	Treatment and Dosage	Number of capsules/volume
**All subjects**<br>stop all therapy after Week 12 if<br>HCV RNA < 25 IU/mL (detectable or undetectable) at Week 2 AND<br>< 25 IU/mL undetectable at Week 4 AND<br>< 25 IU/mL undetectable at Week 8;<br>otherwise continue treatment in Extension		
Week 1 (Day 1) to Week 12 (Day 84) (inclusive)	• TMC435: 150 mg q.d., oral<br>• PegIFNα-2a: 180 µg once weekly, SC<br>• RBV[a]: 1000 mg or 1200 mg in a b.i.d. regimen, oral	• TMC435: one 150-mg capsule<br>• PegIFNα-2a: 1 SC injection of 0.5 mL<br>• RBV: 200-mg tablets as needed according to the subject's weight[a]
**Treatment extension**<br>If HCV RNA at Week 2, 4 and 8 does not meet the above-outlined criteria for treatment stop at Week 12, continue treatment as follows:		
Week 13 (Day 85) to Week 24 (Day 168) (inclusive)	• PegIFNα-2a: 180 µg once weekly, SC<br>• RBV[a]: 1000 mg or 1200 mg in a b.i.d. regimen, oral	• PegIFNα-2a: 1 SC injection of 0.5 mL<br>• RBV: 200-mg tablets as needed according to the subject's weight[a]
SC: subcutaneous<br>[a] RBV dosage is weight-based:<br>Body weight < 75 kg: total daily dose is 1000 mg, administered as 400 mg (2 tablets of 200 mg, morning intake) and 600 mg (3 tablets of 200 mg, evening intake);<br>Body weight ≥ 75 kg: total daily dose is 1200 mg, administered as 2 x 600 mg (3 tablets of 200 mg per intake, morning and evening).		

6.2. Timing of Dosing

Subjects should take the investigational medication (TMC435) once a day, starting the morning or evening of the Baseline visit on Day 1. The investigational medication should be taken at the same time each day throughout the entire treatment period with food. PegIFNα-2a will be administered once weekly in the morning or evening following local practice. Ribavirin should be taken twice daily with food.

The following applies if a scheduled dose of TMC435 is missed:

- if the missed dose is remembered within 12 hours of the scheduled dose time, the dose should be taken as soon as possible.
- if the missed dose is remembered more than 12 hours after the scheduled dose time, the dose should be skipped and the next dose taken at the appropriate time.

If a subject accidentally misses a scheduled dose of PegIFNα-2a or RBV, the investigator should formulate an advice according to the manufacturer's prescribing information for the medication. One missed day of TMC435 or RBV regimen and one missed injection of PegIFNα-2a are not considered as treatment interruptions. All missed doses should be documented.

Dose adjustments or treatment interruptions of PegIFNα-2a and RBV are allowed for tolerability and toxicity issues, considering recommendations in the manufacturer's prescribing information

and the investigator's clinical judgment, and should be clearly linked to a reported AE or SAE. Treatment interruptions of PegIFNα-2a and/or RBV may result in viral breakthrough and treatment failures associated with emergence of HCV PI resistant viral variants. As PegIFNα-2a and/or RBV interruptions may be unavoidable for management of AEs, a maximum interruption of 2 consecutive PegIFNα-2a injections and/or 14 consecutive days of RBV are allowed before the investigational medication must be discontinued. In particular during the phase when the 3 drugs (TMC435/PegIFNα-2a/RBV) are administered (and especially during the first 4 weeks), every effort should be made to avoid or minimize PegIFNα-2a and/or RBV treatment interruptions.

Dose adjustments of TMC435 are not allowed. **If TMC435 is discontinued because a subject meets a virologic stopping rule, it must not be restarted.** If PegIFNα-2a is discontinued for tolerability or toxicity issues or if > 2 consecutive weekly injections are missed, RBV and TMC435 must be discontinued. If RBV is interrupted for more than 14 consecutive days, TMC435 must be discontinued; PegIFNα-2a may be continued at the investigator's discretion. Any additional interruption of RBV or PegIFNα-2a should be discussed with the sponsor on a case-by-case basis.

Temporary interruption of TMC435 during the treatment period will be allowed in the event of suspected toxicity, as long as the temporary interruption is associated with and can be linked to an AE or SAE (permanent discontinuation of one or more study medications may be required, see Section 9.5.7 and Section 10.2). One single treatment interruption for toxicity reasons of TMC435 will be allowed with a maximum duration of 4 days. Treatment with TMC435 should be reinstituted as soon as the AE is stabilized. Reinstitution of TMC435 treatment after interruption for longer than 4 days or after repeated interruption is generally disallowed unless approval by the sponsor has been obtained after discussion on a case-to-case basis.

All modifications, interruptions, or discontinuations of TMC435, PegIFNα-2a, and RBV will be recorded in the eCRF.

Study-site personnel will instruct subjects on how to store study drug for at-home use as indicated for this protocol.

6.3. Virologic Stopping Criteria

Subjects will discontinue medication as soon as possible to avoid continued exposure of subjects to a failing treatment and to limit the risk for evolution of resistant HCV variants.

Subjects meeting any of the following criteria will be required to discontinue all treatment:

- Week 4: HCV RNA ≥ 25 IU/mL
- Week 12: HCV RNA ≥ 25 IU/mL OR < 25 IU/mL detectable
- a confirmed increase of > 1 log_{10} IU/mL in HCV RNA level from the lowest level reached, or a confirmed HCV RNA level of > 100 IU/mL in subjects whose HCV RNA level had previously been < 25 IU/mL while on study drugs.

Detectable HCV RNA after previous undetectability should be confirmed by a confirmatory HCV RNA measurement within 2 weeks after receipt of the detectable HCV RNA value. HCV RNA will be measured centrally and the results will be communicated to the investigational sites. It will be the investigator's responsibility to take the necessary actions regarding the discontinuation of therapy in case a virologic stopping criterion has been met.

Virologic stopping criteria overrule treatment continuation criteria at all times. If a stopping rule is met, treatment should be discontinued and a study medication withdrawal visit should be scheduled as soon as possible. For details on the assessments during the withdrawal visit, see Time and Events Schedule - Part 4.

7. TREATMENT COMPLIANCE

The investigator or designated study-site personnel will maintain a log of all study drug dispensed and returned. Drug supplies for each subject will be inventoried and accounted for at each study visit throughout the study.

Compliance to study medication intake will be assessed by pill counts for TMC435 and RBV, and by full/empty check for PegIFNα-2a boxes. In order to do so, subjects will bring their used and unused medication packages with them at each visit. For further information, refer to Section 14.5, Drug Accountability.

Subjects will receive instructions on compliance with study drug administration at the Baseline/Day 1 visit. During the course of the study, the investigator or designated study-site personnel will be responsible for providing additional instruction to reeducate any subject who is not compliant with taking the study drug.

8. PRESTUDY AND CONCOMITANT THERAPY

Prestudy therapies (prescriptions or over-the-counter medications, including vitamins and herbal supplements, nonpharmacologic therapies such as electrical stimulation, acupuncture, special diets, exercise regimens) administered within 30 days before the start of screening must be recorded at screening.

All therapies (prescription or over-the-counter medications, including vaccines, vitamins, herbal supplements; non-pharmacologic therapies such as electrical stimulation, acupuncture, special diets, exercise regimens) different from the study drug (TMC435 plus PegIFNα-2a and RBV until the end of Week 12, or PegIFNα-2a and RBV alone from Week 13 until Week 24 depending on individual response to treatment) must be recorded in the CRF throughout the study, i.e., from 30 days prior to screening until the last study-related activity. Recorded information will include a description of the type of the drug, treatment period, dosing regimen, route of administration, and its indication.

For study-specific rules on the use of contraceptive methods, refer to Section 4.3, Prohibitions and Restrictions.

TMC435 is a mild inhibitor of intestinal CYP3A. Coadministration of TMC435 with drugs that are primarily metabolized by CYP3A may result in mild increases in plasma concentrations of such drugs, which could increase or prolong therapeutic effect and adverse reactions of CYP3A substrates with narrow therapeutic index.

Drugs that induce CYP3A may decrease TMC435 plasma concentrations and reduce the therapeutic effect of TMC435. Coadministration of TMC435 with inhibitors of CYP3A may increase TMC435 plasma concentrations.

An overview of disallowed concomitant medication is presented below.

1. **Disallowed at any time prior to screening and during the study period, including post-treatment follow-up**
    - Any approved or investigational anti-HCV drug (including vaccines; other than PegIFNα-2a and RBV as of the Baseline/Day 1 visit)

      Notes:

      ♦ Any herbal or nutritional products used for the treatment of liver disease or liver-disease related complications are allowed prior to screening but should be stopped at screening.

      ♦ Subjects who have previously used anti-HCV drug, including any DAA (e.g., NS5B polymerase, NS3/4A protease or NS5A inhibitors, or cyclophilin inhibitors) for the treatment of chronic HCV will be excluded from the study (see Section 4.2).
2. **Disallowed within 30 days of screening, during screening and during the entire treatment period:**
    - All non-HCV investigational drugs or invasive investigational medical devices
    - Experimental non-HCV vaccines

      Note: Approved vaccines are allowed.
    - Praziquantel
3. **Disallowed from screening onwards and during the entire treatment period:**
    - Immunomodulators (e.g., interleukins, systemic corticosteroids[a] in immunosuppressive dose) except for PegIFNα-2a as of the Baseline/Day 1 visit
4. **Disallowed from baseline onwards, during the TMC435 treatment period:**
    - CYP3A4 inducers, such as:

      ♦ The anti-epileptics: carbamazepine, phenytoin and phenobarbital

      ♦ The anti-tuberculosis drugs: rifabutin, rifampin

      ♦ Systemic dexamethasone

      ♦ Miscellaneous: products containing *Hypericum perforatum* (St. John's Wort)
    - CYP3A4 inhibitors, such as:

      ♦ The antifungals (systemic): ketoconazole, itraconazole, posaconazole, voriconazole

- ♦ The macrolide antibiotics: clarithromycin, erythromycin, telithromycin (azithromycin is the preferred option)

- o Drug metabolized only by CYP3A substrates only if they have a small therapeutic index, for example:
  - ♦ The anti-arrhythmics: amiodarone, disopyramide, flecainide, mexiletine, systemic lidocaine, propafenone, quinidine
  - ♦ The antihistamines: astemizole, terfenadine
  - ♦ The gastrointestinal/gastroesophageal reflux disease drugs: cisapride

5. **Allowed but use with caution, i.e., lowest possible dose and monitor for AEs:**
   - o Analgesics: ergaloid mesylates, ergotamine tartrate, dihydroergotamine
   - o Calcium channel blockers: felodipine, nifedipine, nicardipine, amlodipine, bepridil
   - o Lipid-lowering drugs: lovastatin, simvastatin, pravastatin, rosuvastatin, atorvastatin, fluvastatin. A recent drug-drug interaction study indicated that co-administration of TMC435 with rosuvastatin resulted in a 3-fold increase in rosuvastatin levels, most likely due to inhibition of the hepatic uptake transporter OATP1B1 by TMC435. Other statins such as simvastatin and atorvastatin may be less sensitive to an interaction with TMC435, however, clinical data with other statins are not available. If needed, physicians should use the lowest dose possible of statins and carefully monitor patients for any signs or symptoms of AEs that may accompany an increase in statin exposure.
   - o Phosphodiesterase 5 inhibitors: sildenafil, vardenafil, tadalafil
   - o Sedatives/anxiolytics: midazolam, triazolam when orally administered
6. **Allowed but use with special attention as doses may need to be titrated in either direction to achieve the desired therapeutic effect:**
   - o Opioid substitution drugs: buprenorphine, methadone

a Oral corticosteroids < 24h (other than dexamethasone) are allowed in case of rash or acute systemic allergic reaction as outlined in Sections 9.5.7.1 and 9.5.7.2. All other use must be discussed with the sponsor on a case-by-case basis prior to corticosteroid administration.

The list of disallowed concomitant medication is not exhaustive; for drugs falling in one of the categories defined by respective CYP interaction and not mentioned by name, the sponsor should be contacted to determine whether the drug can be allowed.

The approved product information for PegIFNα-2a (Pegasys) and RBV (Copegus) should be consulted for any (additional) prohibited medications.

The sponsor must be notified in advance (or as soon as possible thereafter) of any instances in which prohibited therapies are administered.

For any concomitant therapy given as a treatment for a new condition or a worsening of an existing condition, the condition must be documented in the Adverse Event section of the eCRF.

9. STUDY EVALUATIONS

9.1. Study Procedures

9.1.1. Overview

The Time and Events Schedule summarizes the frequency and timing of efficacy, safety, and other measurements applicable to this study.

Subjects are screened at Visit 1, and within 6 weeks, the site will have received all results to determine the eligibility of the subject. Legitimate delays in starting treatment (e.g., additional medical examinations) may be approved by the sponsor through upfront written communication/motivation by site and approval in writing by sponsor. If eligible, the subject will come for the baseline visit (Day 1). He/she will also start the study medication (TMC435 and PegIFNα-2a/RBV) at that time.

All subjects will follow the visit schedule outlined in the Time and Events Schedule - Part 1. Subjects who meet the criterion to stop all treatment at Week 12 will follow the visit schedule in Time and Events Schedule - Part 2. Subjects who do not meet the criterion to stop all treatment at Week 12 will follow the visit schedule in the Time and Events Schedule - Part 3.

Subjects prematurely discontinuing all study medication (i.e., TMC435, PegIFNα-2a and RBV prior to Week 12, or PegIFNα-2a and RBV after Week 12) for reasons other than withdrawal of consent, will come to the clinic for visits at study medication withdrawal and will thereafter follow the visit schedule as outlined in the Time and Events Schedule - Part 4 without taking study medication.

Investigators and site personnel should inform subjects of the importance to complete the study visit schedule, also in case of discontinuation of study medication intake (for reasons other than withdrawal of consent).

In addition to the scheduled visits and assessments as detailed in the Time and Events Schedule, unscheduled visits may be performed for safety and tolerability reasons, for confirmation of detectable HCV RNA and to communicate the change in treatment in case of virologic failure. Findings during these unscheduled visits need to be reported on special sections of the eCRF.

Study personnel will explain to subjects how to self-administer the PRO measures prior to the first administration of each PRO measure (i.e., during screening visit for the HCV-SIQ and during Baseline/Day 1 visit for the FSS, CES-D, WPAI, and EQ5D). At each visit, subjects should be asked to complete the PRO measures prior to any other study procedures.

Additional serum or urine pregnancy tests may be performed, as determined necessary by the investigator or required by local regulation, to establish the absence of pregnancy at any time during the subject's participation in the study.

The following time windows are recommended:

- For all visits at which study medication is distributed up to Week 12 (Weeks 4, 8, and 12): ± 1 day.
- For all other visits: ± 2 days.

The timing of visits during the treatment period should be based on the start date of medication intake (i.e., on Day 1). The subject should be encouraged to come within these time windows. In the exceptional case that this would not be possible, it might be necessary to request the delivery of the next medication kit ahead of schedule to avoid that the subject runs out of medication.

The total blood volume to be collected from each subject will be included in the patient information sheet/ICF.

Repeat or unscheduled samples may be taken for safety reasons or for technical issues with the samples.

9.1.2. Screening Phase

The screening period will last maximum 6 weeks. Legitimate delays in starting treatment may be approved by the sponsor through upfront written communication/motivation by the site and written approval by the sponsor.

Screening assessments as outlined in the Time and Events Schedule can be done during the screening period and need not necessarily be done at the same moment. When the cap for enrolment has been reached for a specific IL28B genotype, stepwise screening can be performed: after signing of the ICF, IL28B screening can be done first. If the lab results show that the subject has another IL28B genotype than the one for which the enrolment cap was reached, further screening assessments can be undertaken. This way subjects who have an IL28B genotype for which maximum enrollment has been reached do not need to undergo all screening assessments.

Upon confirmation of all eligibility parameters, including availability of all central laboratory parameters, the baseline visit can take place. The locally approved product information of PegIFNα-2a (Pegasys) and RBV (Copegus) should be taken into account to determine eligibility (see also Section 4.1, Inclusion Criteria and Section 4.2, Exclusion Criteria).

Except the tests specified in the Section 4.2, Exclusion Criteria, if the results of other clinical laboratory tests are outside the normal reference ranges, the subject may be included only if the investigator judges the abnormalities or deviations from normal to be not clinically significant or to be appropriate and reasonable for the population under study. This determination must be recorded in the subject's source documents and initialed by the investigator.

Host *IL28B* genotyping is mandated for all subjects who participate. This will be specified on the ICF: subjects who consent to participate in the study also consent to *IL28B* genotyping.

9.1.3. Open-Label Treatment Phase

All subjects will receive TMC435 plus PegIFNα-2a and RBV triple therapy in an open-label fashion according to the Treatment Overview as shown in Table 1 (Section 6), the treatment duration rules (Section 6.1) and virologic stopping rules (Section 6.3).

Blood sampling for HCV RNA determinations and viral sequencing will be done at predefined time points. Sequencing of the HCV NS3/4A region will be done in real-time for the baseline sample. Sequencing of other samples will be at the discretion of the Study Responsible Scientist. Unscheduled HCV RNA assessments may be required.

Safety and tolerability will be assessed at predefined time points as outlined in the Time and Events Schedule. Pregnancy tests will be performed on a regular basis (as specified in the Time and Events Schedule) for female subjects of childbearing potential. Extra pregnancy test kits will be given if more than 4 weeks between two consecutive study visits to perform a pregnancy test at home. Concomitant medication will be recorded throughout the study from signing of ICF until the last study-related visit. The occurrence of AEs will be recorded from signing of ICF until 4 weeks after last study medication intake. Thereafter, only adverse events considered related to TMC435 will be reported. SAEs will be reported until 30 days after last study-related procedure. Unscheduled visits during the treatment period may be performed for safety/tolerability reasons.

The visit-specific PRO assessments (including the HCV-SIQ, WPAI:Hepatitis C, FSS, CES-D, and EQ-5D questionnaires) will be completed at scheduled visits prior to all other study-related procedures planned during these visits. Data will only be collected in countries and at study sites where a translation exists and has been approved by the Independent Ethics Committee (IEC)/ Institutional Review Board (IRB) and Health Authority (if applicable as per local regulations). It is preferable that subjects are able to read and write to complete the questionnaires by themselves. Subjects should not receive any help from anyone accompanying them (such as, family members and friends) or study staff in interpreting or responding to the questions. If a subject is unable to read, or has visual or other physical limitations that make it difficult to read or complete the questionnaires, trained study staff may read the questions and response options aloud, exactly as they appear on the questionnaire, and record the subject’s responses. Study site staff will review the questionnaires after completion to ensure that all questions were completed. If required, subjects will be asked to complete missing items on these questionnaires. A manual with detailed instructions for the completion of PRO questionnaires will be provided separately. The subjects’ responses to the questionnaires will not be used to determine reporting of (serious) AEs.

9.1.4. Posttreatment Phase (Follow-Up)

After the end of the treatment period, all subjects will be followed up for at least 24 weeks, except for those who prematurely discontinue due to withdrawal of consent or viral breakthrough.

9.2. Efficacy

9.2.1. Evaluations

Samples for the determination of HCV RNA will be taken at the time points indicated in the Time and Events Schedule and will be processed in real-time. Procedures for sample collection, processing and storage will be provided in the laboratory manual.

Plasma HCV RNA levels will be determined using an in vitro nucleic acid amplification test for the quantification of HCV RNA in human plasma (Roche COBAS Taqman v2.0)[8,10]. HCV RNA determination will be performed at a central laboratory.

The HCV RNA results will be communicated to the site and the sponsor throughout the study. The investigator will make treatment decisions in line with the treatment duration criteria and virologic stopping rules as outlined in Sections 6.1 and 6.3, respectively.

Changes in HCV RNA plasma levels will not be reported as AEs or SAEs.

9.2.2. Endpoints

Primary Endpoint

In subjects infected with genotype 1 HCV who were eligible for and assigned to a total treatment duration of 12 weeks:

- the proportion of subjects with sustained virologic response 12 weeks after planned end of treatment (SVR12)

For definitions, see Definition of Terms.

Major Secondary Endpoints

In subjects infected with genotype 4 HCV who were eligible for and assigned to a total treatment duration of 12 weeks:

- the proportion of subjects with SVR12

For all subjects per assigned total treatment duration and per HCV genotype (separately):

- the proportion of subjects who achieve RVR;
- the proportion of subjects who achieve virologic response at Week 2 (W2VR);
- the proportion of subjects with SVR12;
- the proportion of subjects with SVR24;
- the proportion of subjects with ≥ 2 log decrease in HCV RNA at each time point;
- the proportion of subjects with HCV RNA < 25 IU/mL undetectable at each time point;
- the proportion of subjects with viral breakthrough;
- the proportion of subjects with viral relapse.

Other Secondary Endpoints

In subjects with genotype 1 or genotype 4 HCV infection for both genotypes combined (subanalyses for each genotype separately will also be done):

- Mean change from baseline at each study visit specified in the Time and Events Schedule throughout treatment and follow-up for each of the following PRO measures:
  - HCV-SIQ symptom score
  - HCV impact score
  - FSS total score
  - CES-D score
  - WPAI missed work time
  - WPAI daily activity impairment
  - WPAI productivity score
  - EQ5D VAS
  - EQ5D valuation index
  - EQ5D Descriptive System scores

In subjects with genotype 1 or genotype 4 HCV infection (separately per genotype):

- the proportion of subjects with normalized ALT levels at the end of study and at time points SVR is assessed.
- Change in liver disease stage assessment between screening assessment and assessment at SVR24 time point

Exploratory Endpoints

In subjects with genotype 1 or genotype 4 HCV infection for both genotypes combined (subanalyses for each genotype separately will also be done):

- Mean change from baseline at each study visit throughout treatment and follow-up for the HCV-SIQ total score
- Mean change from baseline at each study visit throughout treatment and follow-up for the HCV-SIQ symptom scores by body system

For definitions, see Definition of Terms.

For other information, see Section 11.3, Efficacy Analyses.

9.3. Resistance Determinations

Sequencing of the HCV NS3/4A region can be performed to monitor HCV variants present at the time points indicated in the Time and Events Schedule. Sequencing of the baseline sample will be done in real-time. Changes in sequence compared to baseline may indicate the presence of a drug resistant virus. In samples taken after baseline, the NS3/4A region may be sequenced at the

discretion of the Study Responsible Scientist based on the changes in HCV RNA levels observed in each individual subject and the limits of the sequencing assay.

Changes in the viral sequence may be evaluated by the Study Responsible Scientist but will not be regarded as AEs or SAEs.

These samples may be used by the sponsor for additional studies analyzing the genotypic and phenotypic characteristics of HCV present in these samples, upon explicit consent of the subject. No human DNA analysis will be performed on these samples.

9.4. Pharmacogenomic (DNA) Evaluations

Determination of the subject's *IL28B* genotype (single nucleotide polymorphism rs12979860) will be performed on human genomic DNA by PCR. After the results are obtained, these samples will be destroyed.

9.5. Safety Evaluations

Any clinically relevant changes occurring during the study must be recorded on the Adverse Event section of the CRF.

Any clinically significant abnormalities persisting at the end of the study/early withdrawal will be followed by the investigator until resolution or until a clinically stable endpoint is reached.

The study will include the following evaluations of safety and tolerability according to the time points provided in the Time and Events Schedule.

9.5.1. Adverse Events

Adverse events will be reported by the subject (or, when appropriate, by a caregiver, surrogate, or the subject's legally acceptable representative) for the duration of the study.Adverse events will be reported from signing of ICF until 4 weeks after last intake of study medication. Thereafter, only adverse events considered related to TMC435 will be reported. SAEs will be reported until 30 days after last study-related procedure. Adverse events will be followed by the investigator as specified in Section 12, Adverse Event Reporting.

9.5.2. Clinical Laboratory Tests

Blood samples for chemistry and hematology and a random urine sample for urinalysis will be collected. The investigator must review the laboratory report, document this review, and record any clinically relevant changes occurring during the study in the AE section of the CRF. The laboratory reports must be filed with the source documents.

For the biochemistry sample taken at screening, the subjects must have fasted overnight for at least 12 hours. For biochemistry samples taken on Day 1 and at Weeks 4, 12, and 24, the subjects must have fasted overnight for at least 10 hours. At other time points and in case the subject prematurely discontinues study medication (TMC435, PegIFNα-2a and RBV), please refer to the Time and Events Schedule as to whether samples need or are recommended to be taken after a

fast. In the event that values for parameters that are affected by fasting status are outside the normal range, a retest will be required after fasting.

Laboratory abnormalities will be classified by severity according to the WHO grading scale (see Attachment 2). In case a **grade 3 or grade 4** laboratory abnormality occurs, a confirmatory test should be performed by the central laboratory (or, if this is not feasible, by the local laboratory) preferably within 48 hours after the results have become available. All grade 3 and grade 4 laboratory abnormalities and laboratory abnormalities resulting in an increase of 2 grades from baseline will be followed until return to baseline or within 1 grade from baseline.

The following tests will be performed by the central laboratory:

- Hematology Panel

-hemoglobin	-WBC differential
-hematocrit	Neutrophils
- RBC count	Lymphocytes
-RBC parameters:	Monocytes
mean corpuscular hemoglobin (MCH)	Eosinophils
MCH concentration (MCHC)	Basophils
mean corpuscular volume (MCV)	-platelet count
- WBC count	

The samples will also be used for coagulation tests. Prothrombin time (PT) and activated partial thromboplastin time (aPTT) will be assessed, and the INR will be calculated.

- Chemistry Panel

-sodium	-uric acid
-potassium	-calcium (corrected for albumin)
-chloride	- phosphate
-alkaline phosphatase	-albumin
-lactic acid dehydrogenase (LDH)	-total protein
-blood urea nitrogen (BUN)	- total cholesterol
-creatinine	- high-density lipoprotein (HDL) cholesterol
-glucose	- low-density lipoprotein (LDL) cholesterol
-aspartate aminotransferase (AST)	-triglycerides
-alanine aminotransferase (ALT)	-serum magnesium
-gamma-glutamyltransferase (GGT)	-pancreatic amylase
-total, direct, indirect bilirubin	-total lipase
-insulin[a]	- glycosylated hemoglobin (HbA1c)[c]
-creatine phosphokinase (CPK)	- ANA[c]
-TSH (+free T_3 and free T_4 if TSH is abnormal)[b]	

[a] The homeostasis model assessment for insulin resistance (HOMA IR) index will be derived from insulin and glucose (fasted) test results at baseline, Week 4, Week 12, Week 24, Week 36, and Week 48 (as applicable).

[b] Safety sampling will also include a TSH test at screening, and thereafter every 12 weeks after the start of study treatment until the end of PegIFNα-2a and RBV treatment. In case of clinically significant abnormal TSH levels, reflex testing for free T_3 and free T_4 will be performed.

[c] The biochemistry sample taken at screening will include testing for glycosylated hemoglobin (HbA1c) and ANA.

The central laboratory will estimate the creatinine clearance (CLcr) according to the formula derived from the Modification of Diet in Renal Diseases (MDRD) study[30].

A midstream urine sample must be provided for local urinalysis by dipstick. If there are clinically relevant abnormalities in the investigator’s opinion, further urinalysis should be done locally (e.g., sediment analysis including RBC, WBC, casts). Any clinically relevant findings from this local analysis should be reported as an AE.

- Urinalysis

  Dipstick
  -specific gravity
  -pH
  -glucose
  -protein
  -blood
  -ketones
  -bilirubin
  -urobilinogen
  -nitrite
  -leukocyte esterase

- Serum (at screening, all female subjects) and urine (at other time points, female subjects of childbearing potential only) pregnancy testing will be performed. Extra pregnancy test kits will be given if more than 4 weeks between two consecutive study visits to perform a pregnancy test at home. The results of the urine pregnancy test should be recorded in the eCRF and in the subject’s medical records.
- At screening, serology for HIV-1 and HIV-2 antibody, hepatitis A immunoglobulin M (IgM), HBsAg, and anti-HCV antibody will be performed.
- At the screening visit, an additional midstream urine sample must be provided for a urine drug screen. The drug screening involves analysis for amphetamines, benzodiazepines, cocaine, cannabinoids, barbiturates and opioids.
- At screening a schistosomiasis antibody test will be performed. If the schistosomiasis antibody test is positive, a schistosomiasis antigen blood test or rectal snip should be performed.
- At the screening visit, AFP will be tested for all subjects. FSH will be tested for female subjects who are postmenopausal for less than 2 years.
- In case of rash, safety blood samples must be taken, at unscheduled visits, as described in Section 9.5.7.1, and are to be processed by the local laboratory. The following parameters need to be tested: AST, ALT, sedimentation rate and a complete blood cell count (including hemoglobin, hematocrit, RBC count, WBC count, neutrophils, lymphocytes, monocytes, eosinophils, basophils, platelet count) and creatinine.

9.5.3. Electrocardiogram (ECG)

ECG will be performed locally for screening purposes only. If clinically indicated, additional ECG measurements can be performed at the discretion of the investigator.

During the collection of ECGs, subjects should be in a quiet setting without distractions (e.g., television, cell phones). Subjects should rest in a supine position for at least 5 minutes before ECG collection and should refrain from talking or moving arms or legs. If blood sampling or vital sign measurement is scheduled for the same time point as ECG recording, the procedures should be performed in the following order: ECG(s), vital signs, blood draw.

9.5.4. Vital Signs

Systolic and diastolic blood pressure (SBP, DBP) and pulse rate will be recorded according to the Time and Events Schedule.

Blood pressure and pulse/heart rate measurements will be assessed supine with a completely automated device. Manual techniques will be used only if an automated device is not available.

Blood pressure and pulse/heart rate measurements should be preceded by at least 5 minutes of rest in a quiet setting without distractions (e.g., television, cell phones).

9.5.5. Physical Examination

To evaluate the subject's eligibility, a physical examination will be performed at screening. In addition, a physical examination will be performed at several time points throughout the study.

A physical examination includes review of the following systems: general appearance, gastrointestinal system, skin and mucous membranes, eyes, ears, nose, throat, cardiovascular system, respiratory system and genito-urinary system. A neurological and musculo-skeletal examination will be performed as well as a check of the lymph nodes. Other findings will be reported in case the physical examination reveals a clinically significant abnormality not present in this list.

Physical examination at screening should include a fundoscopic eye examination, as per the manufacturer's prescribing information for PegIFNα-2a. Note: Baseline fundoscopic pathology which would require routine follow up on PegIFNα-2a and RBV therapy is considered exclusionary, as per exclusion criterion 7.

To obtain the actual body weight, subjects must be weighed lightly clothed. The height should be measured barefoot at the screening visit.

Any clinically relevant changes occurring during the study must be recorded on the Adverse Event section of the eCRF.

9.5.6. Patient-reported Symptoms, Functioning, and Quality of Life

Five PRO instruments included in this study will be used to prospectively assess symptoms, functional impairments, and HRQoL that subjects experience during and after HCV treatment.

The HCV-SIQ, FSS, CES-D, WPAI:Hepatitis C, and the EQ5D will be completed by subjects at time points indicated in the Time and Events Schedule. Study personnel will instruct subjects in how to self-administer the PRO instruments. If subjects require assistance reading the PRO questionnaires, trained study personnel may read the questions and responses aloud to the subject and record the subject's response on the questionnaire form. Subjects should not be advised by anyone (study personnel, family or others) regarding which answer to give on the PRO questionnaires. For more detailed information about administration of the PRO, please refer to the study manual.

For further guidance, see also Section 9.1.3.

9.5.6.1. Hepatitis C Symptom & Impact Questionnaire

The HCV-SIQ (Attachment 3) is a self-administered questionnaire containing 30 items developed to assess severity or frequency of 26 symptoms associated with HCV or its treatment and 4 questions regarding the impact of symptoms on work/school attendance and daily activities.

Based on information from screening and baseline assessments, a scoring system will be developed for comparison of symptoms and their impact over the course of treatment and follow-up. Three scores will be evaluated as secondary endpoints in this study: a symptom severity score consisting of the sum of responses to the 26 symptom items, a missed work/school score, and a daily activity impairment score. Higher scores indicate worse symptom severity, more time missed from work/school, and more impairment in daily activities, respectively. Measurement properties of the HCV-SIQ have not been evaluated; psychometric evaluation of the HCV-SIQ scores including possible alternative scoring to improve utility of HCV-SIQ scores will be explored as part of this study.

HCV-SIQ symptom scores based on body systems may better characterize symptom experiences during treatment and follow-up. Preliminary scoring by body system will be developed based on data from the screening and baseline visit.

9.5.6.2. Impact on Work and Daily Activities

Impairment associated with HCV infection or its treatment in terms of missed time from work, reduced effectiveness while at work, and impairment in daily activities will be assessed at baseline and at study visits throughout treatment and follow-up as specified in the Time and Events Schedule. The WPAI:Hepatitis C questionnaire (see Attachment 4) includes 6 questions that have been successfully used to compare work productivity and daily activity impairment in patients with chronic HCV infection who received PegIFNα-2a versus the combination of PegIFNα-2b and RBV[39]. Two primary scores are provided in the standard scoring of the WPAI:Hepatitis C: a productivity score (combining missed time and reduced effectiveness at work for subjects who are employed), and a daily activity impairment score for all subjects. In addition, the impact of HCV and its treatment can be examined in terms of missed time from work and reduced effectiveness while at work as separate outcomes.

9.5.6.3. Health Status and Quality of Life

Impact of treatment and of HCV infection on health status and quality of life will be assessed using the EQ5D. Subjects will self-administer the EQ5D at baseline and during study visits throughout treatment and follow-up as specified in the Time and Events Schedule. The EQ-5D questionnaire (see Attachment 5) is an instrument designed to assess overall health status using 5 health dimension scores (mobility, self-care, usual activities, pain/discomfort, anxiety/depression) and a "thermometer" visual analog scale (VAS) ranging from 0 (worst imaginable health state) to 100 (best imaginable health state). It takes approximately 8 minutes to complete and was designed for self-completion by respondents. The EQ-5D questionnaire is commonly used as a brief, generic HRQoL assessment and to incorporate patient preference values into health economic evaluations.

Scores for the EQ5D include the Valuation Index, the visual analog scale (VAS) and 5 health dimension scores (also known as the EQ5D Descriptive System scores).

9.5.6.4. Fatigue

The FSS will be completed by the subject at time points indicated in the Time and Events Schedule. The FSS (Attachment 6) is a self-administered questionnaire with 9 items developed to assess disabling fatigue that has been used extensively in studies of chronic HCV infection[25]. Item responses are measured on a 7-point Likert scale ranging from strongly disagree (1 point) to strongly agree (7 points). The 9 items are averaged to produce a total score; a lower total score indicates less effect of fatigue on everyday life. The FSS has demonstrated good internal consistency, test-retest reliability and construct validity in patients with chronic HCV infection[25]. The recall period of the FSS used in this study is 2 weeks (the past 14 days).

For further guidance, see also Section 9.1.3.

9.5.6.5. Depressive Symptoms

The Center for Epidemiologic Studies Depression Scale (CES-D) will be completed by the subject at time points indicated in the Time and Events Schedule. The CES-D (Attachment 7) is a self-administered, 20-item questionnaire developed based upon extensive in-depth interviews with patients[42]. The CES-D has been used extensively to measure the impact of HCV treatment on depressive symptom severity[12]. A total score for the CES-D is available and is based upon the assessment of the dimensions of depressed affect, positive affect, somatic impact and interpersonal impact of depression. The total score ranges from 0 to 60, with lower scores indicating a lower depressive symptom severity during the past week. The CES-D has demonstrated good internal consistency, test-retest reliability and construct validity in a sample of patients with chronic HCV infection[5].

For further guidance, see also Section 9.1.3.

9.5.7. Specific Toxicities

The safety monitoring and toxicity management plan described below takes into account AEs based on the clinical safety data of TMC435 and toxicities reported with PegIFNα-2a and RBV. For guidance on discontinuation of TMC435 during the treatment period, refer to Section 6.

Note that the following definitions apply:

Investigational medication: TMC435

Study medication: Before Week 12 of the study:
TMC435 plus PegIFNα-2a and RBV triple therapy

After Week 12 of the study:
PegIFNα-2a and RBV

9.5.7.1. Rash

Subjects should be informed that they should contact their study doctor immediately if they notice any rash.

The rash event should be captured in the Adverse Event section of the eCRF.

Monitoring of the evolution of rash will be performed based on the grade (severity) of the rash (see Attachment 8). At the investigator's discretion, additional visits and assessments can be performed.

When safety blood samples are drawn, these should be processed by the local laboratory. The following parameters will need to be tested: AST, ALT, sedimentation rate, and complete blood cell count (including hemoglobin, hematocrit, RBC count, WBC count, platelet count, neutrophils, lymphocytes, monocytes, eosinophils, basophils and creatinine). The values of the local laboratory assessments need to be transcribed in the eCRF by the site staff.

The subject may be treated symptomatically until the rash resolves. Oral antihistamines (e.g., cetirizine, levocetirizine) and/or topical corticosteroids may provide symptomatic relief but effectiveness of these measures has not been established. If systemic corticosteroids for longer than 24h are required for treatment of rash, the investigational medication needs to be permanently discontinued. If the rash is considered to be most likely due to concomitant illness or nonstudy medication, standard management, including discontinuation of the likely causative agent, should be undertaken.

Dermatologist fees for evaluating subjects who experience a rash will be reimbursed by the sponsor.

The following grades are based on the WHO grading table (see Attachment 2), with adaptations made by the sponsor.

Grade 1 Rash (with or without pruritus)

A grade 1 rash is defined as **erythema**.

- Subjects may continue the intake of study medication (at the investigator's discretion). If only TMC435 is discontinued, PegIFNα-2a/RBV can be continued at the investigator's discretion.
- An unscheduled visit including a safety laboratory evaluation should be performed as soon as possible after the subject contacts the investigator to report the AE.
- Assessment of safety blood samples by the local laboratory is required. The values of the local laboratory assessments need to be transcribed in the eCRF by the site staff.

Unscheduled visits will also be performed after the initial rash assessment at the investigator's discretion for appropriate follow-up until resolution of the rash. At these visits, safety blood samples can be taken at the investigator's discretion. For these and all subsequent local laboratory blood sample assessments, the values of the assessments need to be transcribed in the eCRF by the site staff.

The subject should be advised to contact the investigator immediately if there is any worsening of the rash, if any systemic signs or symptoms appear, or if mucosal involvement develops.

In case the rash evolves from a grade 1 to a higher grade, management of the rash should follow the guidelines indicated for grade 2 or grade 3-4 rash, respectively.

Grade 2 Rash (with or without pruritus)

A grade 2 rash is defined as **diffuse, maculopapular rash, OR dry desquamation**.

- Subjects may continue the intake of study medication (at the investigator's discretion). If only TMC435 is discontinued, PegIFNα-2a/RBV can be continued at the investigator's discretion.
- An unscheduled visit including a safety laboratory evaluation should be performed as soon as possible after the subject contacts the investigator to report the AE.
- Assessment of safety blood samples by the local laboratory is required. The values of the local laboratory assessments need to be transcribed in the eCRF by the site staff.
- Referral to a dermatologist is optional but, when done, should occur preferably within 24 hours after the onset of the rash. A copy of the dermatologist's report should be made anonymous and will be collected by the monitor.

Unscheduled visits will also be performed after the initial rash assessment at the investigator's discretion for appropriate follow-up until resolution of the rash. At these visits, safety blood samples must be taken. For these and all subsequent local laboratory blood sample assessments, the values of the assessments need to be transcribed in the eCRF by the site staff.

The subject should be advised to contact the investigator immediately if there is any worsening of the rash, if any systemic signs or symptoms appear, or if mucosal involvement develops.

In case the rash evolves from a grade 2 to a grade 3-4 rash, management of the rash should follow the guidelines specified for grade 3-4 rash.

Grade 3 or Grade 4 Rash

A grade 3 rash is defined as a rash associated with:

- **vesiculation, moist desquamation, or ulceration OR**
- **Any cutaneous event with 1 of the following:**
  - **elevations in AST/ALT more than 2 x baseline value;**
  - **fever > 38°C or 100°F;**
  - **eosinophils > 1000/mm^3;**
  - **serum sickness-like reaction.**

A grade 4 rash is defined as:

- exfoliative dermatitis OR
- mucous membrane involvement OR
- erythema multiforme OR
- Stevens-Johnson Syndrome (SJS) OR
- necrosis requiring surgery.

Subjects will permanently discontinue the intake of study medication. No rechallenge is allowed.

An unscheduled visit including a safety laboratory evaluation should be performed as soon as possible after the subject contacts the investigator to report the AE.

Assessment of safety blood samples by the local laboratory is required on the day of initial rash evaluation and the day thereafter (Days 0 and 1). The values of the local laboratory assessments need to be transcribed in the eCRF by the site staff.

Referral to a dermatologist is required, preferably within 24 hours after the onset of the rash. A copy of the dermatologist's report should be made anonymous and will be collected by the monitor.

A biopsy is to be performed at the discretion of the dermatologist for grade 3 rash and should be performed in case of a grade 4 rash. A copy of the dermatologist's report, and the biopsy if performed, should be made anonymous and will be collected by the monitor.

Appropriate management should be undertaken and subjects should be followed until resolution of the rash or until clinical stability is reached.

A complete summary of the guidelines for rash management is given in Attachment 8.

9.5.7.2. Acute Systemic Allergic Reaction

Oral antihistamines (e.g., cetirizine, levocetirizine) and/or topical corticosteroids may provide symptomatic relief but effectiveness of these measures has not been established. If systemic corticosteroids for longer than 24h are required for treatment of acute systemic allergic reaction, the investigational medication needs to be permanently discontinued.

Grade 1 (Pruritus Suggestive of an Allergic Reaction Without Rash)

Subjects may continue the intake of investigational medication or have their investigational medication discontinued at the investigator's discretion. Subjects may continue treatment with PegIFNα-2a or RBV at the investigator's discretion. Close clinical follow-up is recommended to monitor for any progression of the AE. Subjects should be advised to contact the investigator immediately if there is any worsening of symptoms.

Grade 2 (Localized Urticaria)

Subjects may continue the intake of investigational medication or have their investigational medication discontinued at the investigator's discretion. Subjects may continue treatment with PegIFNα-2a or RBV at the investigator's discretion. Close clinical follow-up is recommended to monitor for any progression of the AE. Subjects should be advised to contact the investigator immediately if there is any worsening of symptoms, in which case the subject will permanently discontinue the study medication. Rechallenge is not allowed.

Grade 3 (Generalized Urticaria, Angioedema) and Grade 4 (Anaphylaxis)

Subjects will immediately and permanently discontinue the intake of study medication. Rechallenge is not allowed. Subjects will be treated as clinically appropriate and should be followed until resolution of the AE.

Refer to Table 2 for an overview of the management scheme in case of an allergic reaction.

Table 2: Guidelines for Subjects Developing Allergic Reactions

WHO Toxicity Grade	Definitions	Investigator Action
Grade 1	Pruritus suggestive of an allergic reaction without rash	May continue investigational medication (TMC435) or have their investigational medication (TMC435) discontinued at the investigator's discretion.<br>May continue treatment with PegIFNα-2a or RBV at the investigator's discretion.
Grade 2	Localized urticaria	May continue investigational medication (TMC435) or have their investigational medication (TMC435) discontinued at the investigator's discretion. Rechallenge is not allowed.<br>May continue treatment with PegIFNα-2a or RBV at the investigator's discretion.
Grade 3	Generalized urticaria, or Angioedema	Permanently discontinue study medication (TMC435, PegIFNα-2a and RBV) (see Section 10.2).
Grade 4	Anaphylaxis	Permanently discontinue study medication (TMC435, PegIFNα-2a and RBV) (see Section 10.2).

9.5.7.3. AST and ALT and/or Bilirubin Elevation

Although an AST and ALT elevation of up to grade 3 is common in chronic HCV due to disease activity, treatment-emergent changes from baseline in ALT and AST levels should be carefully evaluated and closely monitored with unscheduled visits if needed. Increases in bilirubin (both direct and indirect) have been observed during the first weeks of TMC435 therapy. The bilirubin elevations are caused by a benign competitive inhibition of biliary transporter systems in hepatocytes. Bilirubin elevations following initiation of TMC435 therapy are typically not associated with increases in ALT/AST levels and rapidly resolve after completion of TMC435 treatment.

Management of treatment-emergent AST and ALT and/or bilirubin elevations will take into account the following protocol-defined procedures (see also Table 5 and Table 6 below).

In case the investigational medication is discontinued due to increased AST/ALT or bilirubin levels, PegIFNα-2a and RBV therapy may be continued at the investigator's discretion if the increased values are not signs of liver decompensation, but in subjects with progressive ALT increases, the dose of PegIFNα-2a should be reduced to 135 µg and more frequent monitoring of liver function should be performed in line with the manufacturer's prescribing information.

Table 3: Treatment-emergent Grade 1 to Grade 3 ALT or AST elevation or Grade 1 to Grade 3 total bilirubin elevation

WHO Toxicity Grade	AST or ALT, Ranges	Total Bilirubin, Ranges
Grade 1	≥ 1.25 to ≤ 2.5 x ULN	≥ 1.1 to ≤ 1.5 x ULN
Grade 2	> 2.5 to ≤ 5.0 x ULN	> 1.5 to ≤ 2.5 x ULN
Grade 3	> 5.0 to ≤ 10.0 x ULN	> 2.5 to ≤ 5.0 x ULN

Subjects may continue intake of investigational medication or have their investigational medication discontinued at the investigator's discretion. Close clinical follow-up is

recommended to monitor for any progressive increase in ALT/AST or bilirubin levels. In order to continue the study medication, in case of a grade 3 ALT/AST increase, this laboratory abnormality must be judged by the investigator to be either "not related" or "doubtfully related" to the investigational medication. The investigational medication may be continued upon agreement with the sponsor.

Table 4: Treatment-emergent Grade 4 ALT or AST elevation or Grade 4 total bilirubin elevation

WHO Toxicity Grade	AST or ALT, Ranges	Total Bilirubin, Ranges
Grade 4	> 10.0 x ULN	> 5.0 x ULN

If the grade 4 ALT/AST value is > 2 x baseline value, a confirmatory measurement should be performed within 72 hours after receipt of the results. In case of a confirmed value, the investigational medication should be discontinued.

For isolated grade 4 bilirubin values, subjects should have a confirmatory measurement within 72 hours after receipt of the results. In case of a confirmed grade 4 value, the investigational medication should be discontinued. Treatment with PegIFNα-2a and RBV may be continued at the investigator's discretion if the increased values are not accompanied by signs of liver decompensation. Subjects who have known Gilbert's disease and experience isolated grade 4 elevated indirect bilirubin can continue the investigational medication at the investigator's discretion.

For concurrent grade 4 ALT/AST elevations > 2 x baseline value and grade 4 bilirubin values, subjects should have a confirmatory measurement within 72 hours after receipt of the results. In case of confirmed grade 4 elevations, the investigational medication should be discontinued. Treatment with PegIFNα-2a and RBV may be continued at the investigator's discretion if the increased values are not accompanied by signs of liver decompensation.

Subjects should be followed until return to predose baseline or stabilization of ALT/AST and/or bilirubin elevation.

Table 5: Guidelines for Subjects Developing ALT/AST Elevations

WHO Toxicity Grade	Ranges	Investigator Action
Grade 1<br>Grade 2<br>Grade 3	≥ 1.25 to ≤ 2.5 x ULN<br>> 2.5 to ≤ 5.0 x ULN<br>> 5.0 to ≤ 10.0 x ULN	May continue intake of investigational medication or have their investigational medication discontinued at the investigator's discretion.<br>Monitor for progressive increase in ALT/AST levels.<br>In order to continue the study medication, in case of a grade 3 ALT/AST increase, this laboratory abnormality should be considered "not related" or "doubtfully related" to the investigational medication. The investigational medication may be continued upon agreement with the sponsor.
Grade 4	> 10.0 x ULN	May continue study medication or have study medication discontinued at the investigator's discretion if value is < 2 times the baseline value and the event is considered "not related or "doubtfully related" to the study medication. Subjects who continue should be carefully evaluated and close follow-up is recommended to monitor for progressive increase in ALT/AST levels.<br>If the grade 4 ALT/AST value is > 2 x baseline value, a confirmatory measurement should be performed within 72 hours after receipt of the results. In case of a confirmed value, the investigational medication should be discontinued (see also Section 10.2). PegIFNα-2a and RBV may be continued at the investigator's discretion if increased values are not signs of liver decompensation.

Table 6: Guidelines for Subjects Developing Bilirubin Elevations

WHO Toxicity Grade	Ranges	Investigator Action
Grade 1<br>Grade 2<br>Grade 3	≥ 1.1 to ≤ 1.5 x ULN<br>> 1.5 to ≤ 2.5 x ULN<br>> 2.5 to ≤ 5.0 x ULN	May continue intake of investigational medication or have their investigational medication discontinued at the investigator's discretion.<br>Monitor for progressive increase in bilirubin levels.
Grade 4	> 5.0 x ULN	A confirmatory measurement should be performed within 72 hours after receipt of the results. In case of a confirmed value, the investigational medication should be discontinued (see also Section 10.2)[a]. PegIFNα-2a and RBV may be continued at the investigator's discretion if increased values are not signs of liver decompensation.

[a] Subjects who have known Gilbert's disease and experience isolated grade 4 elevated indirect bilirubin can continue the investigational medication at the investigator's discretion.

9.5.7.4. Clinical Hepatitis

Subjects should be monitored for any worsening of their hepatic disease and development of overt signs and symptoms (including increased fatigue, malaise, anorexia, nausea, dark urine, clay colored stools, bilirubinemia, jaundice, liver tenderness, hepatomegaly, or severely increased serum transaminase levels).

Subjects with these signs and symptoms must seek medical attention immediately and have their hepatic parameters assessed. If severe worsening of hepatic disease is evident, all study medication must be discontinued and the sponsor should be contacted.

9.5.7.5. Other Toxicities Considered to be at Least Possibly Related to TMC435

Note: For grade 3 or 4 treatment-emergent laboratory abnormalities, subjects should have a confirmatory measurement within 72 hours after receipt of the results. The management scheme below is for confirmed treatment-emergent laboratory abnormalities and not for isolated and/or non-confirmed events.

Note: If the AE or laboratory abnormality is considered “not related” or “doubtfully related” to the investigational medication, the following rules do not apply and the investigational medication may be continued.

For AEs and laboratory abnormalities considered at least possibly related to TMC435, the following rules should be followed (except for the toxicities detailed in Sections 9.5.7.1 to 9.5.7.4).

Grade 1

Subjects who develop a grade 1 AE or grade 1 laboratory abnormality may continue intake of investigational medication.

Grade 2

Subjects who develop a grade 2 AE or grade 2 laboratory abnormality may continue or discontinue intake of investigational medication based on the investigator’s clinical judgment.

Grade 3

Subjects who develop a grade 3 treatment-emergent AE or confirmed grade 3 laboratory abnormality, considered at least possibly related to the investigational medication, must permanently discontinue intake of investigational medication (TMC435) and should be followed as appropriate until resolution of the AE or laboratory abnormality or stabilization (to be agreed upon in collaboration with the sponsor).

Grade 4

Subjects who develop a grade 4 AE or treatment-emergent confirmed grade 4 laboratory abnormality must permanently discontinue the investigational medication (TMC435) and should be followed as appropriate until resolution of the AE or laboratory abnormality or stabilization (to be agreed upon in collaboration with the sponsor).

9.5.7.6. Toxicity Management for Specific Adverse Events Considered to be at Least Possibly Related to PegIFNα-2a and Ribavirin

Note: the information below does not imply that the following AEs are caused by PegIFNα-2a or RBV therapy alone, they may also be due to TMC435.

Management of AEs associated with the use of PegIFNα-2a and RBV will be at the discretion of the investigator, taking into account generally accepted medical standards for patient monitoring and management, and the investigator's clinical judgment and clinical practice. General guidance is provided in the sections below. In addition, the manufacturer's prescribing information for PegIFNα-2a (Pegasys) and RBV (Copegus) should be consulted.

9.5.7.6.1. Neuropsychiatric Symptoms

Severe psychiatric symptoms, in particular severe depression (including suicidal ideation or attempts), have been reported with PegIFNα-2a/RBV. These reactions may occur in subjects with and without previous psychiatric illness. Subjects who experience psychiatric symptoms should contact the investigator immediately because discontinuation of study medication may be required. investigators should refer subjects reporting such symptoms for immediate psychiatric evaluation/medical intervention.

Study medication must be permanently discontinued in case the alterations in personality behavior or mood prevent the subject from performing usual social and functional activities, are associated with behavior potentially harmful to self or others (e.g., suicidal and homicidal ideation or attempt, acute psychosis), or cause inability to perform basic self-care functions; see Table 7 and Section 10.2).

Table 7: Guidelines for Subjects Developing Psychiatric Symptoms/Depression

**Severity**[a]	**Initial Management** (4-8 weeks)	**Evolution of symptoms**		
		**Remains stable**	**Improves**	**Worsens**
Mild	May continue study medication or have their study medication discontinued at the investigator's discretion.<br>Evaluate once weekly by visit and/or phone.	Continue weekly visit schedule.	Resume normal visit schedule.	See grade 2 or grade 3 symptoms.
Moderate	May continue study medication or have their study medication discontinued at the investigator's discretion.<br>When study medication is continued, reduce to PegIFNα-2a dose to 135 μg (in some cases, a second dose reduction to 90 μg may be needed).<br>Evaluate once weekly (office visit at least every other week).	Consider psychiatric consultation.<br><br>Continue reduced dosing.	If symptoms improve and are stable for 4 weeks, may resume normal visit schedule.<br>Continue reduced dosing or return to normal dose.	See grade 3 symptoms
Severe	Permanently discontinue PegIFNα-2a[b] (see Section 10.2).<br>Obtain immediate psychiatric consultation.	Psychiatric therapy necessary.		

[a] Refer to the Diagnostic and Statistical Manual of Mental Disorders – fifth edition (DSM-V) for definitions.
[b] In case PegIFNα-2a has to be discontinued, TMC435 and RBV must be discontinued as well.

9.5.7.6.2. Anemia

RBV may cause hemolytic anemia, and PegIFNα-2a can suppress bone marrow production of RBCs. Anemia can be serious and subjects should be monitored closely. Anemia should be managed with RBV dose adjustments and general guidelines for RBV dosage modifications (Table 8).

If RBV dose reductions are insufficient for management of anemia, discontinuation of the investigational medication should be considered. If RBV is discontinued for the management of anemia, TMC435 should be discontinued as well. RBV may be restarted per the dosing modification guidelines. Adjustment of the investigational medication dose is not allowed. Use of erythropoiesis-stimulating agents is not disallowed.

Table 8: Guidelines for Subjects Developing Anemia

Laboratory values	Reduce only RBV dose to 600 mg/day[a] if hemoglobin level is	Discontinue RBV[b] if hemoglobin level is
Hemoglobin in subjects with no cardiac disease	< 10 g/dL	< 8.5 g/dL
Hemoglobin in subjects with history of stable cardiac disease	≥ 2 g/dL decrease in hemoglobin during any 4-week treatment period (permanent dose reduction)	< 12 g/dL despite 4 weeks at reduced dose

[a] One 200 mg tablet in the morning and two 200 mg tablets in the evening.
[b] If the abnormality is reversed, RBV may be restarted at 600 mg daily and increased further to 800 mg daily at the discretion of the treating physician. However, returning to higher doses is not recommended. If RBV has to be discontinued, TMC435 must be discontinued as well; PegIFNα-2a may be continued at the investigator's discretion.

9.5.7.6.3. Hyperglycemia

Grade 3, 13.89-27.75 mmol/L (251-500 mg/dL); Grade 4, > 27.75 mmol/L (> 500 mg/dL)

Management decisions must be based on fasted results. If elevated glucose levels are from a non-fasting blood draw, repeat the draw after a 10-hour fast. Subjects with persistent grade 3 or 4 glucose elevations despite anti-hyperglycemic treatment should be permanently discontinued with the exception of subjects with pre-existing diabetes. Appropriate clinical management of hyperglycemia must be started in a timely fashion.

9.5.7.6.4. Renal Complications

Renal safety will be monitored by evaluating serum creatinine levels, estimate CLcr (according to the formula derived from the MDRD study[30] and serum chemistry results. The investigator should closely monitor for disturbances in serum creatinine. If renal complications develop, subjects must be treated as clinically appropriate. The study medication may be continued if the renal complication is considered not to be related to the study medication in the opinion of the investigator. RBV should be discontinued in subjects with creatinine > 2mg/dL or CLcr < 50 mL/min.

9.5.7.6.5. Decreased White Blood Cell Count

PegIFNα-2a may suppress bone marrow production of leukocytes. General guidelines for PegIFNα-2a dosage modification are provided in Table 9. Use of leukopoiesis-stimulating agents is not disallowed.

Table 9: Guidelines for Subjects Developing Decreased White Blood Cell Count

Laboratory values	PegIFNα-2a dose	Discontinue PegIFNα-2a[a] if
ANC ≥ 750/mm^3	Maintain 180 µg	ANC < 500/mm^3; treatment should be suspended until ANC values return to > 1000/ mm^3. Reinstitute at 90 µg and monitor ANC.
ANC < 750/mm^3	Reduce to 135 µg	

ANC = absolute neutrophil count
[a] In case PegIFNα-2a has to be discontinued, TMC435 and RBV must be discontinued as well.

9.5.7.6.6. Decreased Platelet Count

Platelet counts may drop in the setting of PegIFNα-2a and RBV therapy, due at least in part to PegIFNα-2a suppression of platelet production in the bone marrow. General guidelines for PegIFNα-2a dosage modification is provided in Table 10.

Table 10: Guidelines for Subjects Developing Decreased Platelet Count

Laboratory values	PegIFNα-2a dose	Discontinue PegIFNα-2a[a] if
Platelet count ≥ 50,000/mm^3	Maintain 180 µg	Platelet count < 25,000/mm^3
Platelet count < 50,000/mm^3	Reduce to 90 µg	

[a] In case PegIFNα-2a has to be discontinued, TMC435 and RBV must be discontinued as well.

9.5.7.6.7. Thyroid Abnormalities

PegIFNα-2a therapy can be associated with changes in thyroid function, with both hypo- and hyperthyroidism occurring. Such changes are more common in subjects with a history of thyroid dysfunction.

Serum TSH levels should be checked before, during, and after therapy. T3 and free T4 levels should be assessed if changes in TSH values are noted. Subjects should be evaluated by a clinician if biochemical thyroid abnormalities are noted.

Therapy can be stopped and thyroid hormone replacement can be initiated if indicated.

9.6. Sample Collection and Handling

The actual dates and times of sample collection must be recorded in the CRF and laboratory requisition form.

Refer to the Time and Events Schedule for the timing and frequency of all sample collections.

Instructions for the collection, handling, storage, and shipment of samples are found in the laboratory manual that will be provided. Collection, handling, storage, and shipment of samples must be under the specified, and where applicable, controlled temperature conditions as indicated in the laboratory manual.

10. SUBJECT COMPLETION/WITHDRAWAL

10.1. Completion

A subject will be considered to have completed the study if he or she has completed assessments at:

- Week 36 for subjects receiving treatment for a total duration of 12 weeks;
- Week 48 for subjects receiving treatment for a total duration of 24 weeks;
- in case of premature discontinuation of all study medication, refer to Time and Events Schedule - Part 4.

10.2. Discontinuation of Study Treatment

If a subject's study treatment must be discontinued before the end of the treatment regimen, this will not result in automatic withdrawal of the subject from the study.

At the discretion of the investigator, all study medication (TMC435, PegIFNα-2a and RBV) may be discontinued for any of the following reasons:

- An SAE occurs.
- The subject fails to comply with the protocol or study staff requirements.

All study medication (TMC435, PegIFNα-2a and RBV) must be discontinued for any of the following reasons:

- withdrawal of consent.
- The subject meets a virologic stopping criterion at Week 4 or 12 or experiences viral breakthrough at any time point during treatment (see Section 6.3).
- The investigator believes that for safety reasons (e.g., AE) it is in the best interest of the subject to discontinue study treatment.
- The subject becomes pregnant.
- A subject fails to comply with the contraception requirements (in female subjects; refer to Section 4.3).
- The subject experiences a grade 3 or 4 rash/allergic reaction (see Sections 9.5.7.1 and 9.5.7.2).
- The subject experiences severe worsening of hepatic disease (see Section 9.5.7.4).

Subjects must discontinue investigational medication (TMC435) and may continue PegIFNα-2a and/or RBV alone if:

- They require treatment with one of the medications included in the list of disallowed concomitant medications (see Section 8).

- The subject experiences grade 4 ALT/AST elevations > 2 x baseline value and concurrent grade 4 bilirubin values and the ALT/AST and bilirubin values continue to meet grade 4 criteria at re-testing performed 72 hours after receipt of the results (see Section 9.5.7.3).
- The subject experiences a grade 3 or 4 AE (other than the rash/allergic reaction AE mentioned above) or confirmed grade 3 or 4 laboratory abnormality (other than grade 3 isolated increases in AST/ALT or bilirubin) considered to be at least possibly related to TMC435 (2 consecutive measurements no more than 72 hours after receipt of first results). This excludes AEs or laboratory abnormalities that are not considered to be at least possibly related to TMC435. Subjects who have known Gilbert's disease and experience isolated grade 4 elevated indirect bilirubin can continue the investigational medication at the investigator's discretion.

If a subject discontinues study treatment before the end of the treatment regimen, end-of-treatment and follow-up assessments should be obtained and scheduled assessments should be continued as outlined in Time and Events Schedule - Part 4.

The date and the reason for treatment discontinuation must be noted on the eCRF.

10.3. Withdrawal From the Study

A subject will be withdrawn from the study for any of the following reasons:

- Lost to follow-up
- Withdrawal of consent
- Death

If a subject is lost to follow-up, every reasonable effort must be made by the study site personnel to contact the subject and determine the reason for discontinuation/withdrawal. The measures taken to follow up must be documented.

When a subject withdraws before completing the study, the reason for withdrawal is to be documented in the CRF and in the source document. Study drug assigned and dispensed to the withdrawn subject may not be assigned to another subject. Subjects who withdraw will not be replaced.

Withdrawal From the Use of Samples in Future Research

The subject may withdraw consent for use of samples for research (refer to Section 16.2.5, Long-Term Retention of Samples for Additional Future Research). In such a case, samples will be destroyed after they are no longer needed for the clinical study. Details of the sample retention for research are presented in the main ICF.

11. STATISTICAL METHODS

Statistical analysis will be done by the sponsor or under the authority of the sponsor. A general description of the statistical methods to be used to analyze the efficacy and safety data is outlined below. Specific details will be provided in the Statistical Analysis Plan.

The analysis population is the intent-to-treat (ITT) population. The ITT population will include all subjects who took at least one dose of study medication.

Planned interim analyses are described in Section 11.5.

The primary analysis will be performed when all subjects infected with genotype 1 HCV who were eligible for and assigned to a total treatment duration of 12 weeks have completed the Week 24 visit (SVR12) or discontinued earlier.

The final analysis will be performed when all subjects have completed the last study-related visit (Week 36 or Week 48 depending on total treatment duration) or discontinued earlier.

For definitions, see Definition of Terms.

11.1. Subject Information

For all subjects who receive at least 1 dose of study drug descriptive statistics will be provided. All demographic characteristics (e.g., age, height, weight, BMI, race, ethnic origin) and other initial subject characteristics (physical examination, medical and surgical history, concomitant diseases, baseline HCV RNA) will be tabulated and analyzed descriptively.

11.2. Sample Size Determination

The primary efficacy parameter is SVR12 for subjects infected with genotype 1 HCV who were eligible for and assigned to a total treatment duration of 12 weeks.

One hundred and fifty subjects with genotype 1 HCV infection and with fibrosis stages ranging from equivalent to F0 to equivalent to F2 will be treated. With the assumption 50% will have genotype 1a HCV infection and 50% genotype 1b, and with estimated *IL28B* genotype breakdown being 30% CC, 55% CT and 15% TT, it is expected 69% (104 subjects) will have a Week 2 response and reach RVR and be eligible for a total treatment duration of 12 weeks.

The minimally acceptable response rate (SVR12 in subjects eligible for and assigned to 12 weeks of treatment) is taken as 80%, whilst the target response rate is 90%. With an alpha=0.05, and a sample size of 104, based on these assumptions, the calculated power would be 90.6%. As the N is assumed, several scenarios are presented below:

N	Target Response	Power
96	90%	83.9%
104	90%	90.6%
112	90%	90.8%

In addition, 75 subjects with genotype 4 HCV infection and with fibrosis stages ranging from equivalent to F0 to equivalent to F2 will be treated. With the assumption the estimated *IL28B* genotype breakdown will be 30% CC, 55% CT and 15% TT, it is expected 69% (52 subjects) will have a Week 2 response and reach RVR and be eligible for a total treatment duration of

12 weeks. With a target response rate of 90%, 52 subjects will allow the response rate to be estimated with a 2-sided 95% CI width of 18.1%: 90% (78.5%, 96.6%).

11.3. Efficacy Analyses

For a list of primary and secondary endpoints, see Section 9.2.2, Endpoints.

For the primary endpoint, SVR12 in subjects with genotype 1 HCV infection who were eligible for and assigned to 12 weeks of treatment, a one-sided test with alpha=0.05 will be used to compare the observed response rate to the minimally acceptable response rate of 80%.

For all proportions listed in Section 9.2.2, Endpoints, 2-sided 95% confidence intervals will be constructed around the observed response rate.

11.4. Safety Analyses

Safety endpoints will be analysed in subjects infected with genotype 1 or genotype 4 HCV separately per genotype and for both genotypes combined.

Adverse Events

The verbatim terms used in the CRF by investigators to identify AEs will be coded using the Medical Dictionary for Regulatory Activities (MedDRA). All reported AEs with onset during the treatment phase (i.e., treatment-emergent AEs, and AEs that have worsened since baseline) will be included in the analysis. For each AE, the percentage of subjects who experience at least 1 occurrence of the given event will be summarized by treatment group.

Summaries, listings, datasets, or subject narratives may be provided, as appropriate, for those subjects who die, who discontinue treatment due to an AE, or who experience a severe or a serious AE.

All events of special interest will be evaluated in conjunction with other systemic symptoms and laboratory abnormalities: information on time of onset, duration of events, time to resolution, concomitant therapies, and relationship to TMC435, PegIFNα-2a, RBV, and HCV infection will be tabulated.

Clinical Laboratory Tests

The laboratory abnormalities will be determined according to the WHO grading table (see Attachment 1) and in accordance with the normal ranges of the clinical laboratory. Laboratory abnormalities will be tabulated by the scheduled time points and summarized by type of laboratory test. For the clinical laboratory data, descriptive statistics (actual values and changes from baseline) will be generated for all tests performed

A listing of subjects with any markedly abnormal laboratory results will also be provided.

Vital Signs

Descriptive statistics of pulse/heart rate and blood pressure (systolic and diastolic) values and changes from baseline will be summarized at each scheduled time point. The percentage of subjects with values beyond clinically important limits will be summarized.

Physical Examination

Physical examination findings and changes from baseline will be summarized at each scheduled time point. Physical examination results will be tabulated and abnormalities will be listed.

BMI will be calculated using the recording of height at screening. Body weight and BMI results will be tabulated and analyzed descriptively.

Patient-reported Symptoms, Functioning, and Quality of Life

The Statistical Analysis Plan will specify analyses to evaluate impact of HCV or its treatment on the 5 PRO instruments, and to propose scoring for the HCV-SIQ and evaluate its measurement properties.

11.5. Interim Analyses

An interim analysis of PRO data from the screening and baseline visits will be conducted to establish and test the scoring of the HCV-SIQ.

Interim analyses will be done for subjects infected with genotype 1 HCV:

- when the first 35 subjects that were eligible for a total treatment duration of 12 weeks and completed their treatment have reached the time point of SVR4 (4 weeks after planned end of treatment, corresponding to Week 16).
- when all subjects eligible for a total treatment duration of 12 weeks have completed the last study-related visit (Week 36) or discontinued earlier.

An additional interim analysis may be done when all subjects infected with genotype 1 HCV who were eligible for and assigned to a total treatment duration of 12 weeks have reached the time point of SVR4 or have discontinued earlier.

An interim analysis will be done for subjects infected with genotype 4 HCV:

- when all subjects eligible for and assigned to a total treatment duration of 12 weeks have completed the Week 24 visit (SVR12) or discontinued earlier.

An additional interim analysis may be conducted when the first 20 subjects infected with genotype 4 HCV who were eligible for a total treatment duration of 12 weeks and completed their treatment have reached the time point of SVR4.

Given the timing of the above-listed interim analyses, if 2 of the planned analyses are close in meeting their cut-off point, one may be slightly delayed to allow them to be done concurrently.

11.6. Data Monitoring Committee

An independent Data Monitoring Committee (DMC) will be established to monitor data on an ongoing basis to ensure the continuing safety of the subjects enrolled in this study and to meet efficacy objectives. The committee will meet periodically to review interim data. After the review, the DMC will make recommendations regarding the continuation of the study. The details will be provided in a separate DMC charter.

The DMC will consist of at least one medical expert in the relevant therapeutic area and at least one statistician. The DMC responsibilities, authorities, and procedures will be documented in its charter.

12. ADVERSE EVENT REPORTING

Timely, accurate, and complete reporting and analysis of safety information from clinical studies are crucial for the protection of subjects, investigators, and the sponsor, and are mandated by regulatory agencies worldwide. The sponsor has established Standard Operating Procedures in conformity with regulatory requirements worldwide to ensure appropriate reporting of safety information; all clinical studies conducted by the sponsor or its affiliates will be conducted in accordance with those procedures.

12.1. Definitions

12.1.1. Adverse Event Definitions and Classifications

Adverse Event

An AE is any untoward medical occurrence in a clinical study subject administered a medicinal (investigational or non-investigational) product. An AE does not necessarily have a causal relationship with the treatment. An AE can therefore be any unfavorable and unintended sign (including an abnormal finding), symptom, or disease temporally associated with the use of a medicinal (investigational or non-investigational) product, whether or not related to that medicinal (investigational or non-investigational) product. (Definition per International Conference on Harmonisation [ICH])

This includes any occurrence that is new in onset or aggravated in severity or frequency from the baseline condition, or abnormal results of diagnostic procedures, including laboratory test abnormalities.

Note: The sponsor collects AEs starting with the signing of the ICF (refer to Section 12.3.1, All Adverse Events, for time of last AE recording).

Serious Adverse Event

A SAE based on ICH and EU Guidelines on Pharmacovigilance for Medicinal Products for Human Use is any untoward medical occurrence that at any dose:

- Results in death

- Is life-threatening
(The subject was at risk of death at the time of the event. It does not refer to an event that hypothetically might have caused death if it were more severe.)
- Requires inpatient hospitalization or prolongation of existing hospitalization
- Results in persistent or significant disability/incapacity
- Is a congenital anomaly/birth defect
- Is a suspected transmission of any infectious agent via a medicinal product
- Is Medically Important*

*Medical and scientific judgment should be exercised in deciding whether expedited reporting is also appropriate in other situations, such as important medical events that may not be immediately life threatening or result in death or hospitalization but may jeopardize the subject or may require intervention to prevent one of the other outcomes listed in the definition above. These should usually be considered serious.

Unlisted (Unexpected) Adverse Event/Reference Safety Information

An AE is considered unlisted if the nature or severity is not consistent with the applicable product reference safety information. For TMC435, the expectedness of an AE will be determined by whether or not it is listed in the Investigator's Brochure. For a non-sponsor investigational medicinal product (e.g., a comparator product) with a marketing authorization, the expectedness of an AE will be determined by whether or not it is listed in the manufacturer's prescribing information.

Adverse Event Associated With the Use of the Drug

An AE is considered associated with the use of the drug if the attribution is possible, probable, or very likely by the definitions listed in Section 12.1.2.

12.1.2. Attribution Definitions

Not Related

An AE that is not related to the use of the drug.

Doubtful

An AE for which an alternative explanation is more likely, e.g., concomitant drug(s), concomitant disease(s), or the relationship in time suggests that a causal relationship is unlikely.

Possible

An AE that might be due to the use of the drug. An alternative explanation, e.g., concomitant drug(s), concomitant disease(s), is inconclusive. The relationship in time is reasonable; therefore, the causal relationship cannot be excluded.

Probable

An AE that might be due to the use of the drug. The relationship in time is suggestive (e.g., confirmed by dechallenge). An alternative explanation is less likely, e.g., concomitant drug(s), concomitant disease(s).

Very Likely

An AE that is listed as a possible adverse reaction and cannot be reasonably explained by an alternative explanation, e.g., concomitant drug(s), concomitant disease(s). The relationship in time is very suggestive (e.g., it is confirmed by dechallenge and rechallenge).

12.1.3. Severity Criteria

An assessment of severity grade will be made using the general categorical descriptors as outlined in the WHO Toxicity Grading Scale in Attachment 2.

The investigator should use clinical judgment in assessing the severity of events not directly experienced by the subject (e.g., laboratory abnormalities).

12.2. Special Reporting Situations

Safety events of interest on a sponsor study drug that may require expedited reporting and/or safety evaluation include, but are not limited to:

- Overdose of a sponsor study drug
- Suspected abuse/misuse of a sponsor study drug
- Inadvertent or accidental exposure to a sponsor study drug
- Medication error involving a sponsor product (with or without subject/patient exposure to the sponsor study drug, e.g., name confusion)

Special reporting situations should be recorded in the CRF. Any special reporting situation that meets the criteria of a SAE should be recorded on the serious adverse event page of the CRF.

12.3. Procedures

12.3.1. All Adverse Events

All AEs and special reporting situations, whether serious or non-serious, will be reported from the time a signed and dated ICF is obtained until 4 weeks after last study medication intake. Thereafter, only AEs considered related to TMC435 will be reported. Serious adverse events, including those spontaneously reported to the investigator within 30 days after the last study-related procedure, must be reported using the Serious Adverse Event Form. The sponsor will evaluate any safety information that is spontaneously reported by an investigator beyond the time frame specified in the protocol.

All AEs, regardless of seriousness, severity, or presumed relationship to study drug, must be recorded using medical terminology in the source document and the CRF. Whenever possible, diagnoses should be given when signs and symptoms are due to a common etiology (e.g., cough,

runny nose, sneezing, sore throat, and head congestion should be reported as "upper respiratory infection"). Investigators must record in the CRF their opinion concerning the relationship of the AE to study therapy. All measures required for AE management must be recorded in the source document and reported according to sponsor instructions.

The sponsor assumes responsibility for appropriate reporting of AEs to the regulatory authorities. The sponsor will also report to the investigator (and the head of the investigational institute where required) all SAEs that are unlisted (unexpected) and associated with the use of the study drug. The investigator (or sponsor where required) must report these events to the appropriate Independent Ethics Committee/Institutional Review Board (IEC/IRB) that approved the protocol unless otherwise required and documented by the IEC/IRB.

Subjects (or their designees, if appropriate) must be provided with a "study card" indicating the following:

- Subject's name
- Study number
- Investigator's name and 24-hour contact telephone number
- Sponsor's name and 24-hour contact telephone number
- Statement, in the local language(s), that the subject is participating in a clinical study.

12.3.2. Serious Adverse Events

All SAEs occurring during the study must be reported to the appropriate sponsor contact person by study-site personnel within 24 hours of their knowledge of the event.

Information regarding SAEs will be transmitted to the sponsor using the Serious Adverse Event Form, which must be completed and signed by a physician from the study site, and transmitted to the sponsor within 24 hours. The initial and follow-up reports of a SAE should be made by facsimile (fax).

All SAEs that have not resolved by the end of the study, or that have not resolved upon discontinuation of the subject's participation in the study, must be followed until any of the following occurs:

- The event resolves
- The event stabilizes
- The event returns to baseline, if a baseline value/status is available
- The event can be attributed to agents other than the study drug or to factors unrelated to study conduct
- It becomes unlikely that any additional information can be obtained (subject or health care practitioner refusal to provide additional information, lost to follow-up after demonstration of due diligence with follow-up efforts)

Suspected transmission of an infectious agent by a medicinal product will be reported as a SAE. Any event requiring hospitalization (or prolongation of hospitalization) that occurs during the course of a subject's participation in a study must be reported as a SAE, except hospitalizations for the following:

- Hospitalizations not intended to treat an acute illness or AE (e.g., social reasons such as pending placement in long-term care facility)
- Surgery or procedure planned before entry into the study (must be documented in the CRF). Note: Hospitalizations that were planned before the signing of the ICF, and where the underlying condition for which the hospitalization was planned has not worsened, will not be considered SAEs. Any AE that results in a prolongation of the originally planned hospitalization is to be reported as a new SAE.

The cause of death of a subject in a study, whether or not the event is expected or associated with the study drug, is considered a SAE.

The first report of an SAE may also be made by telephone. The investigator must provide the minimal information: i.e. study number, subject's date of birth, medication code number, period of intake, nature of the AE, and investigator's attribution.

This report of an SAE by telephone must always be confirmed by a written, more detailed report (the Serious Adverse Event Form) to be completed and signed by the investigator. If initial reporting was done by telephone, the person answering the phone can complete the Serious Adverse Event Form according to the information provided by the investigator. This form needs to be reviewed, completed if applicable, signed, and dated for approval by the investigator.

The start date of the SAE documented on the Serious Adverse Event form must be the same as the start date of the corresponding AE documented on the eCRF. If a change in severity is noted for the existing AE, it must be recorded as a new AE. If a worsened AE meets the criteria for an SAE, the start date of the SAE must be the same as the start date of the worsened AE.

12.3.3. Pregnancy

All initial reports of pregnancy must be reported to the sponsor by the study-site personnel within 24 hours of their knowledge of the event using the appropriate pregnancy notification form. Abnormal pregnancy outcomes (e.g., spontaneous abortion, stillbirth, and congenital anomaly) are considered SAEs and must be reported using the Serious Adverse Event Form. Any subject who becomes pregnant during the study must discontinue further study treatment.

In case the partner of a male subject becomes pregnant, the male subject must abstain from sex with that female partner during the pregnancy. It is highly recommended that pregnancies in partners of male subjects included in the study are reported by the investigational staff within 24 hours of their knowledge of the event using the appropriate pregnancy notification form. In view of the privacy regulations, a separate consent of the partner will be obtained at that time.

Follow-up information regarding the outcome of the pregnancy and any postnatal sequelae in the infant will be required.

12.4. Contacting Sponsor Regarding Safety

The names (and corresponding telephone numbers) of the individuals who should be contacted regarding safety issues or questions regarding the study are listed on the Contact Information page(s), which will be provided as a separate document.

13. PRODUCT QUALITY COMPLAINT HANDLING

A product quality complaint (PQC) is defined as any suspicion of a product defect related to manufacturing, labeling, or packaging, i.e., any dissatisfaction relative to the identity, quality, durability, or reliability of a product, including its labeling or package integrity. A PQC may have an impact on the safety and efficacy of the product. Timely, accurate, and complete reporting and analysis of PQC information from studies are crucial for the protection of subjects, investigators, and the sponsor, and are mandated by regulatory agencies worldwide. The sponsor has established procedures in conformity with regulatory requirements worldwide to ensure appropriate reporting of PQC information; all studies conducted by the sponsor or its affiliates will be conducted in accordance with those procedures.

13.1. Procedures

All initial PQCs must be reported to the sponsor by the study-site personnel as soon as possible after being made aware of the event.

If the defect is combined with a SAE, the study-site personnel must report the PQC to the sponsor according to the SAE reporting timelines (refer to Section 12.3.2, Serious Adverse Events). A sample of the suspected product should be maintained for further investigation if requested by the sponsor.

13.2. Contacting Sponsor Regarding Product Quality

The names (and corresponding telephone numbers) of the individuals who should be contacted regarding product quality issues are listed on the Contact Information page(s), which will be provided as a separate document.

14. STUDY DRUG INFORMATION

14.1. Physical Description of Study Drug(s)

TMC435 supplied for this study is formulated as an oral capsule containing TMC435 sodium salt equivalent to 150 mg TMC435, sodium lauryl sulphate, magnesium stearate (vegetable grade), anhydrous colloidal silica, croscarmellose sodium, and lactose monohydrate. It will be manufactured and provided under the responsibility of the sponsor.

All subjects will receive PegIFNα-2a/RBV treatment in addition to TMC435.

PegIFNα-2a (Pegasys) will be supplied as a syringe containing 0.5 mL solution with 180 μg PegIFNα-2a.

Ribavirin (Copegus) will be supplied as a light pink to pink colored, flat, oval-shaped, film-coated tablet for oral administration. Each tablet contains 200 mg of ribavirin.

14.2. Packaging

The investigational medication (TMC435) will be packaged under responsibility of the sponsor.

For commercial PegIFNα-2a (Pegasys), prefilled syringes from Quality Assurance (QA)-released commercial stock will be delivered (with graduation), packed in a paper box together with plunger and needle and labeled with a study-specific label under the responsibility of the sponsor (see Section 14.3).

Commercial RBV (Copegus) will be provided from the QA-released commercial stock and labeled with a study-specific label under the responsibility of the sponsor (see Section 14.3).

Sites should use the relabeled commercial supplies for Pegasys and Copegus as supplied by the sponsor. In the rare event that centrally provided relabeled supplies cannot be used, locally prescribed commercially available Pegasys or Copegus may be provided to the study subject. Use of prescribed commercial Pegasys or Copegus should be documented in the subject's source notes as well as in the eCRF.

All study drug will be dispensed in child-resistant packaging.

No medication can be repacked without prior approval from the sponsor.

Medication management will be done through the Interactive Web-based Response System (IWRS).

14.3. Labeling

Study drug labels will contain information to meet the applicable regulatory requirements.

No medication can be relabeled without prior approval from the sponsor.

14.4. Preparation, Handling, and Storage

TMC435 capsules must be stored at a room temperature of 15-30°C (59-86°F) and in the original container delivered under the responsibility of the sponsor.

PegIFNα-2a must be stored at 2-8°C (35.6-46.4°F) in a refrigerator and must not be frozen. The prefilled syringes should be kept in the outer carton to protect from light. More details on procedures for handling and storage can be found in the manufacturer’s prescribing information.

RBV should be stored at or below 30°C (86°F). Procedures for handling and storage of RBV are detailed in the manufacturer’s prescribing information.

Regular temperature logging of the medication storage room at the investigational site should be performed. Should a deviation in storage conditions occur, the site must not further dispense the

affected drug, quarantine the drug in IWRS, and must provide the monitor immediately with the following information:

- Study number;
- Reference or batch number(s);
- Kit number;
- Site number;
- Temperature log (including date and duration of the deviation, and the minimum temperature below the range and/or maximum temperature above the range that the product was exposed to);
- Used units (C or °F).

Deviations in storage conditions will be evaluated by the sponsor/stability manager.

14.5. Drug Accountability

The investigator is responsible for ensuring that all study drug received at the site is inventoried and accounted for throughout the study. The dispensing of study drug to the subject, and the return of study drug from the subject (if applicable), must be documented on the drug accountability form. Subjects must be instructed to return all original containers, whether empty or containing study drug. All study drug will be stored and disposed of according to the sponsor's instructions. Study-site personnel must not combine contents of the study drug containers.

All empty, partially used and unused packages for TMC435, PegIFNα-2a and RBV must be returned by the subject for accountability purposes by the site staff. Packages cannot be destroyed before full accountability is performed and without prior approval from the site manager.

Syringes used for administration of PegIFNα-2a must be discarded at home by the subject in a waste container. Waste containers will be provided to each subject upon enrolment in the study. The needle should not be removed from the syringe when discarding the syringe into the waste container. Full waste containers should be brought back to the study center for appropriate destruction. Drug accountability of PegIFNα-2a will be performed on returned empty packages of used syringes and returned unopened packages of unused syringes only.

Study drug must be handled in strict accordance with the protocol and the container label, and must be stored at the study site in a limited-access area or in a locked cabinet under appropriate environmental conditions. Unused study drug, and study drug returned by the subject, must be available for verification by the sponsor's study site monitor during on-site monitoring visits. The return to the sponsor of unused study drug, or used returned study drug for destruction, will be documented on the drug return form. When the study site is an authorized destruction unit and study drug supplies (including full waste containers for PegIFNα-2a) are destroyed on-site, this must be documented on the drug destruction form.

Study drug disposal including disposal of the full waste containers for PegIFNα-2a must comply with local guidelines or hospital waste management policies.

Study drug should be dispensed under the supervision of the investigator or a qualified member of the study-site personnel, or by a hospital/clinic pharmacist. Study drug will be supplied only to subjects participating in the study. Returned study drug must not be dispensed again, even to the same subject. Study drug may not be relabeled or reassigned (after dispensation) for use by other subjects. The investigator agrees neither to dispense the study drug from, nor store it at, any site other than the study sites agreed upon with the sponsor.

15. STUDY-SPECIFIC MATERIALS

The investigator will be provided with the following supplies:

- Investigator's Brochure for TMC435
- Pharmacy manual/study site investigational product manual
- Laboratory manual
- A binder with PRO instruments including instructions for use
- Contact information pages;
- eDC Manual
- Sample ICF

16. ETHICAL ASPECTS

16.1. Study-Specific Design Considerations

Potential subjects will be fully informed of the risks and requirements of the study and, during the study, subjects will be given any new information that may affect their decision to continue participation. They will be told that their consent to participate in the study is voluntary and may be withdrawn at any time with no reason given and without penalty or loss of benefits to which they would otherwise be entitled. Only subjects who are fully able to understand the risks, benefits, and potential AEs of the study, and provide their consent voluntarily will be enrolled.

16.2. Regulatory Ethics Compliance

16.2.1. Investigator Responsibilities

The investigator is responsible for ensuring that the study is performed in accordance with the protocol, current ICH guidelines on Good Clinical Practice (GCP), and applicable regulatory and country-specific requirements.

Good Clinical Practice is an international ethical and scientific quality standard for designing, conducting, recording, and reporting studies that involve the participation of human subjects. Compliance with this standard provides public assurance that the rights, safety, and well-being of study subjects are protected, consistent with the principles that originated in the Declaration of Helsinki, and that the study data are credible.

16.2.2. Independent Ethics Committee or Institutional Review Board

Before the start of the study, the investigator (or sponsor where required) will provide the IEC/IRB with current and complete copies of the following documents (as required by local regulations):

- Final protocol and, if applicable, amendments
- Sponsor-approved ICF (and any other written materials to be provided to the subjects)
- Investigator's Brochure (or equivalent information) and amendments/addenda
- Sponsor-approved subject recruiting materials
- Information on compensation for study-related injuries
- Investigator's curriculum vitae or equivalent information (unless not required, as documented by the IEC/IRB)
- Information regarding funding, name of the sponsor, institutional affiliations, other potential conflicts of interest, and incentives for subjects
- Any other documents that the IEC/IRB requests to fulfill its obligation

This study will be undertaken only after the IEC/IRB has given full approval of the final protocol, amendments (if any, excluding the ones that are purely administrative, with no consequences for subjects, data or study conduct), the ICF, applicable recruiting materials, and subject compensation programs, and the sponsor has received a copy of this approval. This approval letter must be dated and must clearly identify the IEC/IRB and the documents being approved.

During the study the investigator (or sponsor where required) will send the following documents and updates to the IEC/IRB for their review and approval, where appropriate:

- Protocol amendments (excluding the ones that are purely administrative, with no consequences for subjects, data or study conduct)
- Revision(s) to ICF and any other written materials to be provided to subjects
- If applicable, new or revised subject recruiting materials approved by the sponsor
- Revisions to compensation for study-related injuries or payment to subjects for participation in the study, if applicable
- New edition(s) of the Investigator's Brochure and amendments/addenda
- Summaries of the status of the study at intervals stipulated in guidelines of the IEC/IRB (at least annually)
- Reports of AEs that are serious, unlisted/unexpected, and associated with the study drug
- New information that may adversely affect the safety of the subjects or the conduct of the study
- Deviations from or changes to the protocol to eliminate immediate hazards to the subjects

- Report of deaths of subjects under the investigator's care
- Notification if a new investigator is responsible for the study at the site
- Development Safety Update Report and Line Listings, where applicable
- Any other requirements of the IEC/IRB

For all protocol amendments (excluding the ones that are purely administrative, with no consequences for subjects, data or study conduct), the amendment and applicable ICF revisions must be submitted promptly to the IEC/IRB for review and approval before implementation of the change(s).

At least once a year, the IEC/IRB will be asked to review and reapprove this study. The reapproval should be documented in writing (excluding the ones that are purely administrative, with no consequences for subjects, data, or study conduct).

At the end of the study, the investigator (or sponsor where required) will notify the IEC/IRB about the study completion.

16.2.3. Informed Consent

Each subject must give written consent according to local requirements after the nature of the study has been fully explained. The ICF(s) must be signed before performance of any study-related activity. The ICF(s) that is/are used must be approved by both the sponsor and by the reviewing IEC/IRB and be in a language that the subject can read and understand. The informed consent should be in accordance with principles that originated in the Declaration of Helsinki, current ICH and GCP guidelines, applicable regulatory requirements, and sponsor policy.

Before enrollment in the study, the investigator or an authorized member of the study-site personnel must explain to potential subjects the aims, methods, reasonably anticipated benefits, and potential hazards of the study, and any discomfort participation in the study may entail. Subjects will be informed that their participation is voluntary and that they may withdraw consent to participate at any time. They will be informed that choosing not to participate will not affect the care the subject will receive for the treatment of his or her disease. Subjects will be told that alternative treatments are available if they refuse to take part and that such refusal will not prejudice future treatment. Finally, they will be told that the investigator will maintain a subject identification register for the purposes of long-term follow up if needed and that their records may be accessed by health authorities and authorized sponsor personnel without violating the confidentiality of the subject, to the extent permitted by the applicable law(s) or regulations. By signing the ICF the subject is authorizing such access, and agrees to allow his or her study physician to recontact the subject for the purpose of obtaining consent for additional safety evaluations, if needed.

The subject will be given sufficient time to read the ICF and the opportunity to ask questions. After this explanation and before entry into the study, consent should be appropriately recorded by means of the subject's personally dated signature. After having obtained the consent, a copy of the ICF must be given to the subject.

Where local regulations require, a separate ICF may be used for the required DNA component of the study.

If the subject is unable to read or write, an impartial witness should be present for the entire informed consent process (which includes reading and explaining all written information) and should personally date and sign the ICF after the oral consent of the subject is obtained.

16.2.4. Privacy of Personal Data

The collection and processing of personal data from subjects enrolled in this study will be limited to those data that are necessary to fulfill the objectives of the study.

These data must be collected and processed with adequate precautions to ensure confidentiality and compliance with applicable data privacy protection laws and regulations. Appropriate technical and organizational measures to protect the personal data against unauthorized disclosures or access, accidental or unlawful destruction, or accidental loss or alteration must be put in place. Sponsor personnel whose responsibilities require access to personal data agree to keep the identity of subjects confidential.

The informed consent obtained from the subject includes explicit consent for the processing of personal data and for the investigator/institution to allow direct access to his or her original medical records (source data/documents) for study-related monitoring, audit, IEC/IRB review, and regulatory inspection. This consent also addresses the transfer of the data to other entities and to other countries.

The subject has the right to request through the investigator access to his or her personal data and the right to request rectification of any data that are not correct or complete. Reasonable steps will be taken to respond to such a request, taking into consideration the nature of the request, the conditions of the study, and the applicable laws and regulations.

16.2.5. Long-Term Retention of Samples for Additional Future Research

All subjects who consent to participate in the study give consent that any samples collected as per Time and Events Schedule may be stored by the sponsor for up to a maximum of 15 years after the end of the study, unless local regulations require a shorter storage period. Upon explicit consent, samples that remain after study-related analyses may be used by the sponsor for further exploratory research, if applicable. Samples will only be used to understand TMC435, to understand HCV infection, to understand differential drug responders, and to develop tests/assays related to TMC435 and HCV infection. The research may begin at any time during the study or the post-study storage period.

Stored samples will be coded throughout the sample storage and analysis process and will not be labeled with personal identifiers.

Subjects may withdraw their consent for their samples to be used for further exploratory research (refer to Section 10.3, Withdrawal From the Study (Withdrawal From the Use of Samples in Future Research).

16.2.6. Country Selection

This study will only be conducted in those countries where the intent is to launch or otherwise help ensure access to the developed product, unless explicitly addressed as a specific ethical consideration in Section 16.1, Study-Specific Design Considerations.

17. ADMINISTRATIVE REQUIREMENTS

17.1. Protocol Amendments

Neither the investigator nor the sponsor will modify this protocol without a formal amendment by the sponsor. All protocol amendments must be issued by the sponsor, and signed and dated by the investigator. Protocol amendments must not be implemented without prior IEC/IRB approval, or when the relevant competent authority has raised any grounds for non-acceptance, except when necessary to eliminate immediate hazards to the subjects, in which case the amendment must be promptly submitted to the IEC/IRB and relevant competent authority. Documentation of amendment approval by the investigator and IEC/IRB must be provided to the sponsor. When the change(s) involves only logistic or administrative aspects of the study, the IRB (and IEC where required) only needs to be notified.

During the course of the study, in situations where a departure from the protocol is unavoidable, the investigator or other physician in attendance will contact the appropriate sponsor representative (see Contact Information page(s) provided separately). Except in emergency situations, this contact should be made <u>before</u> implementing any departure from the protocol. In all cases, contact with the sponsor must be made as soon as possible to discuss the situation and agree on an appropriate course of action. The data recorded in the CRF and source documents will reflect any departure from the protocol, and the source documents will describe this departure and the circumstances requiring it.

17.2. Regulatory Documentation

17.2.1. Regulatory Approval/Notification

This protocol and any amendment(s) must be submitted to the appropriate regulatory authorities in each respective country, if applicable. A study may not be initiated until all local regulatory requirements are met.

17.2.2. Required Prestudy Documentation

The following documents must be provided to the sponsor before a site will be declared open and screening can start:

- Protocol and amendment(s), if any, signed and dated by the principal investigator
- A copy of the dated and signed (or sealed, where appropriate per local regulations), written IEC/IRB approval of the protocol, amendments, ICF, any recruiting materials, and if applicable, subject compensation programs. This approval must clearly identify the specific protocol by title and number and must be signed (or sealed, where appropriate per local regulations) by the chairman or authorized designee.

- Name and address of the IEC/IRB, including a current list of the IEC/IRB members and their function, with a statement that it is organized and operates according to GCP and the applicable laws and regulations. If accompanied by a letter of explanation, or equivalent, from the IEC/IRB, a general statement may be substituted for this list. If an investigator or a member of the study-site personnel is a member of the IEC/IRB, documentation must be obtained to state that this person did not participate in the deliberations or in the vote/opinion of the study.
- Regulatory authority approval or notification, if applicable
- Signed and dated statement of investigator, if applicable
- Documentation of investigator qualifications (e.g., curriculum vitae)
- Completed investigator financial disclosure form from the principal investigator, where required
- Signed and dated clinical trial agreement, which includes the financial agreement
- Any other documentation required by local regulations

The following documents must be provided to the sponsor before enrollment of the first subject:

- Completed investigator financial disclosure forms from all subinvestigators
- Documentation of subinvestigator qualifications (e.g., curriculum vitae)
- Name and address of any local laboratory conducting tests for the study, and a dated copy of current laboratory normal ranges for these tests, if applicable
- Local laboratory documentation demonstrating competence and test reliability (e.g., accreditation/license), if applicable

17.3. Subject Identification, Enrollment, and Screening Logs

The investigator agrees to complete a subject identification code list to permit easy identification of each subject during and after the study. This document will be reviewed by the sponsor study-site contact for completeness.

The subject identification code list will be treated as confidential and will be filed by the investigator in the study file. To ensure subject confidentiality, no copy will be made. All reports and communications relating to the study will identify subjects by subject identification and date of birth. In cases where the subject is not randomized into the study, the date seen and date of birth will be used.

The investigator must also complete a subject screening and enrolment log, which reports on all subjects who were seen to determine eligibility for inclusion in the study.

17.4. Source Documentation

At a minimum, source documentation must be available for the following to confirm data collected in the CRF: subject identification, eligibility, and study identification; study discussion and date of signed informed consent; dates of visits; results of safety and efficacy parameters as

required by the protocol; record of all AEs and follow-up of AEs; concomitant medication; drug receipt/dispensing/return records; study drug administration information; and date of study completion and reason for early discontinuation of study drug or withdrawal from the study, if applicable.

In addition, the author of an entry in the source documents should be identifiable.

At a minimum, the type and level of detail of source data available for a subject should be consistent with that commonly recorded at the study site as a basis for standard medical care. Specific details required as source data for the study will be reviewed with the investigator before the study and will be described in the monitoring guidelines (or other equivalent document).

Some data will be recorded directly into the CRF and will be considered source data. Data that will be recorded directly into the eCRF are specified in the Source Document Identification Form that will be signed by the investigator at site initiation.

PRO assessments will be recorded by the subjects on paper forms, then sent by study personnel for data entry by the CRO.

17.5. Case Report Form Completion

Case report forms are provided for each subject in electronic format.

Electronic Data Capture (eDC) will be used for this study. The study data will be transcribed by study-site personnel from the source documents onto an electronic CRF, and transmitted in a secure manner to the sponsor within the timeframe agreed upon between the sponsor and the study site. The electronic file will be considered to be the CRF.

Worksheets may be used for the capture of some data to facilitate completion of the CRF. Any such worksheets will become part of the subject's source documentation. All data relating to the study must be recorded in CRFs prepared by the sponsor. Data must be entered into CRFs in English. Study site personnel must complete the CRF as soon as possible after a subject visit, and the forms should be available for review at the next scheduled monitoring visit.

All subjective measurements (e.g., pain scale information or other questionnaires) will be completed by the same individual who made the initial baseline determinations whenever possible. The investigator must verify that all data entries in the CRFs are accurate and correct.

All CRF entries, corrections, and alterations must be made by the investigator or other authorized study-site personnel. If necessary, queries will be generated in the eDC tool The investigator or study-site personnel must adjust the CRF (if applicable) and complete the query.

If corrections to a CRF are needed after the initial entry into the CRF, this can be done in 3 different ways:

- Study site personnel can make corrections in the eDC tool at their own initiative or as a response to an auto query (generated by the eDC tool).
- Study site manager can generate a query for resolution by the study-site personnel.
- Clinical data manager can generate a query for resolution by the study-site personnel.

17.6. Data Quality Assurance/Quality Control

Steps to be taken to ensure the accuracy and reliability of data include the selection of qualified investigators and appropriate study sites, review of protocol procedures with the investigator and study-site personnel before the study, periodic monitoring visits by the sponsor, and direct transmission of clinical laboratory data from a central laboratory into the sponsor's data base. Written instructions will be provided for collection, handling, storage, and shipment of samples.

Guidelines for CRF completion will be provided and reviewed with study-site personnel before the start of the study. The sponsor will review CRFs for accuracy and completeness during on-site monitoring visits and after transmission to the sponsor; any discrepancies will be resolved with the investigator or designee, as appropriate. After upload of the data into the study database they will be verified for accuracy and consistency with the data sources.

17.7. Record Retention

In compliance with the ICH/GCP guidelines, the investigator/institution will maintain all CRFs and all source documents that support the data collected from each subject, as well as all study documents as specified in ICH/GCP Section 8, Essential Documents for the Conduct of a Clinical Trial, and all study documents as specified by the applicable regulatory requirement(s). The investigator/institution will take measures to prevent accidental or premature destruction of these documents.

Essential documents must be retained until at least 2 years after the last approval of a marketing application in an ICH region and until there are no pending or contemplated marketing applications in an ICH region or until at least 2 years have elapsed since the formal discontinuation of clinical development of the investigational product. These documents will be retained for a longer period if required by the applicable regulatory requirements or by an agreement with the sponsor. It is the responsibility of the sponsor to inform the investigator/institution as to when these documents no longer need to be retained.

If the responsible investigator retires, relocates, or for other reasons withdraws from the responsibility of keeping the study records, custody must be transferred to a person who will accept the responsibility. The sponsor must be notified in writing of the name and address of the new custodian. Under no circumstance shall the investigator relocate or dispose of any study documents before having obtained written approval from the sponsor.

If it becomes necessary for the sponsor or the appropriate regulatory authority to review any documentation relating to this study, the investigator/institution must permit access to such reports.

17.8. Monitoring

The sponsor will perform on-site monitoring visits as documented in the Monitoring Guidelines. The monitor will record dates of the visits in a study site visit log that will be kept at the study site. The first post-initiation visit will be made as soon as possible after enrollment has begun. At these visits, the monitor will compare the data entered into the CRFs with the hospital or clinic records (source documents). The nature and location of all source documents will be identified to ensure that all sources of original data required to complete the CRF are known to the sponsor and study-site personnel and are accessible for verification by the sponsor study-site contact. If electronic records are maintained at the study site, the method of verification must be discussed with the study-site personnel.

Direct access to source documentation (medical records) must be allowed for the purpose of verifying that the data recorded in the CRF are consistent with the original source data. Findings from this review of CRFs and source documents will be discussed with the study-site personnel. The sponsor expects that, during monitoring visits, the relevant study-site personnel will be available, the source documentation will be accessible, and a suitable environment will be provided for review of study-related documents. The monitor will meet with the investigator on a regular basis during the study to provide feedback on the study conduct.

17.9. Study Completion/Termination

17.9.1. Study Completion

The study is considered completed with the last visit for the last subject participating in the study. The final data from the study site will be sent to the sponsor (or designee) after completion of the final subject visit at that study site, in the time frame specified in the Clinical Trial Agreement.

17.9.2. Study Termination

The sponsor reserves the right to close the study site or terminate the study at any time for any reason at the sole discretion of the sponsor. Study sites will be closed upon study completion. A study site is considered closed when all required documents and study supplies have been collected or destroyed and a study-site closure visit has been performed.

The investigator may initiate study-site closure at any time, provided there is reasonable cause and sufficient notice is given in advance of the intended termination.

Reasons for the early closure of a study site by the sponsor or investigator may include but are not limited to:

- Failure of the investigator to comply with the protocol, the requirements of the IEC/IRB or local health authorities, the sponsor's procedures, or GCP guidelines
- Inadequate recruitment of subjects by the investigator

- Discontinuation of further study drug development. One possible reason for the discontinuation of the medication may be because the risks for the participants are no longer medically justifiable.

17.10. On-Site Audits

Representatives of the sponsor's clinical quality assurance department may visit the study site at any time during or after completion of the study to conduct an audit of the study in compliance with regulatory guidelines and company policy. These audits will require access to all study records, including source documents, for inspection and comparison with the CRFs. Subject privacy must, however, be respected. The investigator and study-site personnel are responsible for being present and available for consultation during routinely scheduled study-site audit visits conducted by the sponsor or its designees.

Similar auditing procedures may also be conducted by agents of any regulatory body, either as part of a national GCP compliance program or to review the results of this study in support of a regulatory submission. The investigator should immediately notify the sponsor if they have been contacted by a regulatory agency concerning an upcoming inspection.

17.11. Use of Information and Publication

All information, including but not limited to information regarding TMC435 or the sponsor's operations (e.g., patent application, formulas, manufacturing processes, basic scientific data, prior clinical data, formulation information) supplied by the sponsor to the investigator and not previously published, and any data, including pharmacogenomic research data, generated as a result of this study, are considered confidential and remain the sole property of the sponsor. The investigator agrees to maintain this information in confidence and use this information only to accomplish this study, and will not use it for other purposes without the sponsor's prior written consent.

The investigator understands that the information developed in the study will be used by the sponsor in connection with the continued development of TMC435, and thus may be disclosed as required to other clinical investigators or regulatory agencies. To permit the information derived from the clinical studies to be used, the investigator is obligated to provide the sponsor with all data obtained in the study.

The sponsor will create a DSUR and Line Listings (if applicable) in accordance with the ICH guideline E2F with a data lock date of 7 January. This study (TMC435HPC3014) will be included in the first DSUR produced after approval of this protocol, which may be less than 1 year's time from this particular study approval date, and in all subsequent DSURs, as appropriate, until final CSR of the study is available.

The results of the study will be reported in a Clinical Study Report generated by the sponsor and will contain CRF data from all study sites that participated in the study. Recruitment performance or specific expertise related to the nature and the key assessment parameters of the study will be used to determine a coordinating investigator. Results of analyses performed after the Clinical Study Report has been issued will be reported in a separate report and will not

require a revision of the Clinical Study Report. Study subject identifiers will not be used in publication of results. Any work created in connection with performance of the study and contained in the data that can benefit from copyright protection (except any publication by the investigator as provided for below) shall be the property of the sponsor as author and owner of copyright in such work.

Consistent with Good Publication Practices and International Committee of Medical Journal Editors guidelines, the sponsor shall have the right to publish such primary (multicenter) data and information without approval from the investigator. The investigator has the right to publish study site-specific data after the primary data are published. If an investigator wishes to publish information from the study, a copy of the manuscript must be provided to the sponsor for review at least 60 days before submission for publication or presentation. Expedited reviews will be arranged for abstracts, poster presentations, or other materials. If requested by the sponsor in writing, the investigator will withhold such publication for up to an additional 60 days to allow for filing of a patent application. In the event that issues arise regarding scientific integrity or regulatory compliance, the sponsor will review these issues with the investigator. The sponsor will not mandate modifications to scientific content and does not have the right to suppress information. For multicenter study designs and substudy approaches, secondary results generally should not be published before the primary endpoints of a study have been published. Similarly, investigators will recognize the integrity of a multicenter study by not submitting for publication data derived from the individual study site until the combined results from the completed study have been submitted for publication, within 12 months of the availability of the final data (tables, listings, graphs), or the sponsor confirms there will be no multicenter study publication. Authorship of publications resulting from this study will be based on the guidelines on authorship, such as those described in the Uniform Requirements for Manuscripts Submitted to Biomedical Journals, which state that the named authors must have made a significant contribution to the design of the study or analysis and interpretation of the data, provided critical review of the paper, and given final approval of the final version.

Registration of Clinical Studies and Disclosure of Results

The sponsor will register and/or disclose the existence of and the results of clinical studies as required by law.

REFERENCES

1. Aerssen J, Fanning G, Scholliers A, et al. Impact of IL28B genotype and pretreatment serum IP-10 in treatment-naive genotype-1 HCV patients treated with TMC435 in combination with peginterferon alfa-2a and ribavirin in PILLAR study. 46th Annual Meeting of the European Association for the Study of the Liver (EASL 2011).

2. Afdahl N, O'Brien C, Godofsky E, et al. Valopicitabine (NM283), alone or with peginterferon, compared to peginterferon/ribavirin retreatment in patients with HCV-1 infection and prior non-response to PegIFN/RBV: one-year results. Data presented at the Annual Meeting of the European Association for the Study of the Liver (EASL). Barcelona, Spain. 11-15 April 2007.

3. Bacon BR, Gordon SC, Lawitz E, et al.; HCV RESPOND-2 Investigators. Boceprevir for previously treated chronic HCV genotype 1 infection. N Engl J Med. 2011; 364 (13): 1207-1217.

4. Bronowicki JP, Hezode C, Bengtsson L, et al. 100% SVR in IL28B CC Patients Treated with 12 Weeks of Telaprevir, Peginterferon and Ribavirin in the PROVE 2 Trial. 47th Annual Meeting of the European Association for the Study of the Liver (EASL 2012).

5. Clark Ch, Mahoney JS, Clark DJ, Eriksen LR. Screening for depression in a hepatitis C populations: the reliability and validity of the Center for Epidemiologic Studies Depression Scale (CES-D). J Adv Nurs. 2002; 40: 361-369.

6. Clinical Research Report, TMC435350-TiDP16-C202. An open-label trial in genotype 2, 3, 4, 5 and 6 hepatitis C-infected subjects to evaluate the antiviral activity, safety, tolerability and pharmacokinetics of TMC435350 following 7 days once daily dosing as monotherapy. Tibotec Pharmaceuticals (11 May 2010).

7. Clinical Study Report, TMC435-TiDP16-C205. A Phase IIb, randomized, double-blind, placebo-controlled trial to investigate the efficacy, tolerability, safety and pharmacokinetics of TMC435 as part of a treatment regimen including peginterferon alfa-2a and ribavirin in treatment-naïve genotype 1 hepatitis C-infected subjects. Janssen Research & Development (30 March 2012).

8. COBAS® TaqMan® HCV Test, v2.0 For Use With The High Pure System. 2010.

9. Copegus® Summary of Product Characteristics. Hertfordshire, UK: Roche Products Limited, 2012. Available at: http://www.medicines.org.uk/emc/medicine/11755/SPC/Copegus%20200mg%20and%20400mg%20Film-coated%20Tablets#furtherInfo. Accessed 26 Nov 2012.

10. Covance Central Laboratory Services Method Summary HCV viral load using Roche COBAS TaqMan HCV/HPS assay v. 2.0

11. European Association for the Study of the Liver. EASL Clinical Practice Guidelines: management of hepatitis C virus infection. J Hepatol 2011;55 (2): 245-264.

12. Evon DM , Ramcharran D , Belle SH. Prospective Analysis of Depression During Peginterferon and Ribavirin Therapy of Chronic Hepatitis C: Results of the Virahep-C Study. Am J Gastroenterol. 2009; 104:2949–2958.

13. Ferenci P, Laferl H, Scherzer TM, et al. Peginterferon alfa-2a and ribavirin for 24 weeks in hepatitis C type 1 and 4 patients with rapid virological response. Gastroenterology 2008; 135 (2): 451-458.

14. Fried M, Buti M, Dore GJ, et al. TMC435 in Combination with Peginterferon and Ribavirin in Treatment-Naive HCV Genotype 1 Patients: Final Analysis of the PILLAR Phase IIb Study. 62nd Annual Meeting of the American Association for the Study of Liver Diseases (AASLD 2011).

15. Fried MW, Hadziyannis SJ, Shiffman ML, Messinger D, Zeuzem S. Rapid virological response is the most important predictor of sustained virological response across genotypes in patients with chronic hepatitis C virus infection. J Hepatol. 2011; 55 (1): 69-75.

16. Gilles L, Beumont-Mauviel M, Peeters M, SCOTT J Reducing time with HCV treatment-related fatigue: results from the simeprevir (TMC435) phase IIB treatment-naive PILLAR trial. Poster no-269 at IDWEEK 2012 - Joint Meeting of the Infectious Diseases Society of America (IDSA), the Society for Healthcare Epidemiology of America (SHEA), the HIV Medicine Association (HIVMA), and the Pediatric Infectious Diseases Society (PIDS), San Diego, California, USA, October 17-21, 2012.

17. Hézode C, Forestier N, Dusheiko G, et al.; PROVE2 Study Team. Telaprevir and peginterferon with or without ribavirin for chronic HCV infection. N Engl J Med. 2009; 360 (18): 1839-1850.

18. Investigator's Brochure: TMC435 (simeprevir) Edition 7. Janssen Research and Development, June 2013.

19. Investigator's Brochure: TMC435 (simeprevir) Edition 7, Addendum 1. Janssen Research and Development, October 2013

20. Jacobson IM, McHutchison JG, Dusheiko G, et al. Telaprevir for previously untreated chronic hepatitis C virus infection. N Engl J Med. 2011; 364(25): 2405-2416.

21. Jensen DM, Marcellin P, Freilich B, et al. Re-treatment of patients with chronic hepatitis C who do not respond to peginterferon-alpha2b: a randomized trial. Ann Intern Med. 2009; 150 (8): 528-540.

22. Kamal SM. Hepatitis C virus genotype 4 therapy: progress and challenges. Liver International. 2011; ISSN 1478-3223.

23. Kamal SM, El Kamary SS, Shardell MD, et al. Pegylated Interferon Alpha-2b Plus Ribavirin in Patients with Genotype 4 Chronic Hepatitis C: The Role of Rapid and Early Virologic Response. Hepatology. 2007; 46 (6): 1732-1740.

24. Keeffe EB. Chronic hepatitis C: management of treatment failures. Clin Gastroenterol Hepatol. 2005; 3 (10 Suppl. 2): S102-S105.

25. Kleinman L, Zodet W, Kakim Z, et al. Psychometric evaluation of the fatigue severity scale for use in chronic hepatitis C. Qual Life Res. 2000; 9: 499-508.

26. Koike K. Antiviral treatment of hepatitis C: present status and future prospects. J Infect Chemother. 2006; 12 (5): 227-232.

27. Koziel M, Peters M. Viral hepatitis in HIV infection. N Engl J Med. 2007; 356 (14): 1445-1454.

28. Kwo PY, Lawitz EJ, McCone J, Schiff ER, Vierling JM, Pound D, et al. Efficacy of boceprevir, an NS3 protease inhibitor, in combination with peginterferon alfa-2b and ribavirin in treatment-naïve patients with genotype 1 hepatitis C infection (SPRINT-1): an open-label randomised, multi-centre Phase II trial. Lancet. 2010; 2376 (9742): 705-716.

29. Lawitz E, Mangia A, Wyles D, et al. Sofosbuvir for previously untreated chronic hepatitis C infection. N Engl J Med. 2013; 368 (20): 1878-1887.

30. Levey AS, Bosch JP, Breyer Lewis J, et al. A more accurate method to estimate glomerular filtration rate from serum creatinine: a new prediction equation. Ann Int Med. 1999; 130 (6): 461-470.

31. Manns MP, Foster GR, Rockstroh JK, Zeuzem S, Zoulim F, Houghton M. The way forward in HCV treatment--finding the right path. Nat Rev Drug Discov 2007; 6 (12): 991-1000. Erratum in: Nat Rev Drug Discov 2008; 7(5): 458. Nat Rev Drug Discov. 2008; 7 (1): 102.

32. Marcellin P et al., EASL 2013 Poster #865

33. McHutchison JG, Manns M, Patel K, et al.; Adherence to combination therapy enhances sustained response in genotype-1-infected patients with chronic hepatitis C. Gastroenterology 2002;123 (4): 1061-1069.

34. McHutchison JG, Everson GT, Gordon SC, et al.; PROVE1 Study Team. Telaprevir with peginterferon and ribavirin for chronic HCV genotype 1 infection. N Engl J Med. 2009; 360 (18): 1827-1838. Erratum in: N Engl J Med. 2009; 361 (15): 1516.

35. McHutchison JG, Manns MP, Muir AJ, et al.; PROVE3 Study Team. Telaprevir for previously treated chronic HCV infection. N Engl J Med. 2010; 362 (14): 1292-1303. Erratum in: N Engl J Med. 2010; 362 (17): 1647.

36. Moreno C, Hezode C, Marcellin P et al. Simeprevir with peginterferon/ribavirin in treatment-naïve or experienced patients with chronic HCV genotype 4 infection: Interim results of a Phase III trial. European AIDS Clinical Society (EACS) 2013, LBPS9/6.

37. Nelson et al., EASL 2013 poster #881

38. Pegasys® Summary of Product Characteristics. Hertfordshire, UK: Roche Products Limited, 2012. Available at: http://www.ema.europa.eu/ema/index.jsp?curl=pages/medicines/human/medicines/000395/human_med_000974.jsp&mid=WC0b01ac058001d124. Accessed 26 Nov 2012.

39. Perrillo R, Rothstein KD, Rubin R, et al. Comparison of quality of life, work productivity and medical resource utilization of peginterferon alpha 2a vs. the combination of interferon alpha 2b plus ribavirin as initial treatment in patients with chronic hepatitis C. J Viral Hepat. 2004; 11: 157-165.

40. Poordad F, McCone J Jr, Bacon BR, et al.; SPRINT-2 Investigators. Boceprevir for untreated chronic HCV genotype 1 infection. N Engl J Med. 2011; 364 (13): 1195-1206.

41. Poynard T, Colombo S, Bruix J, et al. Peginterferon alfa-2b and ribavirin: effective in patients with hepatitis C who failed interferon-alfa/ribavirin therapy. Gastroenterology. 2009; 136 (5): 1618-1628.e2.

42. Radloff LS. The CES-D scale: A self-report depression scale for research in the general population. Appl Psychol Meas 1977; 1: 385-401.

43. Scherzer TM, Stättermayer AF, Strasser M, et al. Impact of IL28B on treatment outcome in hepatitis C virus G1/4 patients receiving response-guided therapy with peginterferon alpha-2a (40KD)/ribavirin. Hepatology 2011; 54 (5): 1518-1526.

44. Sherman KE, Flamm SL, Afdhai NH, et al. Telaprevir in combination with peginterferon alfa2a and ribavirin for 24 or 48 weeks in treatment-naïve genotype 1 HCV patients who achieved an extended rapid viral response: final results of Phase 3 ILLUMINATE study. Data presented at the 61st Annual Meeting of the American Association for the Study of Liver Diseases (AASLD). Boston, MA, USA. 29 October - 02 November 2010. [abstract LB-2].

45. Shiffman ML, Salvatore J, Hubbard S, et al. Treatment of chronic hepatitis C virus genotype 1 with peginterferon, ribavirin, and epoetin alpha. Hepatology. 2007; 46 (2): 371-379.

46. The Global Burden of Hepatitis C Working Group. Global burden of disease (GBD) for hepatitis C. J Clin Pharmacol. 2004; 44 (1): 20-29.

47. Zeuzem S, Andreone P, Stanislas P, et al. Telaprevir for retreatment of HCV infection. N Engl J Med. 2011; 364 (25): 2417-2428.

ATTACHMENTS

Attachment 1: Metavir Scoring System, 1997

0: No fibrosis

1: Stellate enlargement of portal tracts without septae formation

2: Enlargement of portal tracts with rare septae formation

3: Numerous septae without cirrhosis

4: Cirrhosis

The French METAVIR Cooperative Study Group. Intraobserver and interobserver variations in liver biopsy interpretation in patients with chronic hepatitis C. Hepatology. 1994; 20: 15-20.

Attachment 2: WHO Toxicity Grading Scale for Determining the Severity of Adverse Events, February 2003

ABBREVIATIONS (used in the table):

ULN = Upper Limit of Normal

LLN = Lower Limit of Normal

R_x = Therapy

IV = Intravenous

FEV_1 = forced expiratory volume in 1 second

ESTIMATING SEVERITY GRADE

For abnormalities NOT found elsewhere in the Toxicity Tables use the scale below to estimate grade of severity:

**GRADE 1**	**Mild**	Transient or mild discomfort (< 48 hours); no medical intervention/therapy required.
**GRADE 2**	**Moderate**	Mild to moderate limitation in activity; some assistance may be needed; no or minimal medical intervention/ therapy required.
**GRADE 3**	**Severe**	Marked limitation in activity; some assistance usually required; medical intervention/therapy required; hospitalizations possible.
**GRADE 4**	**Potentially life threatening**[a]	Extreme limitation in activity; significant assistance required; significant medical intervention/therapy required; hospitalization or hospice care probable.

[a] Revised by the sponsor

COMMENTS REGARDING THE USE OF THESE TABLES

- For parameters not included in the following Toxicity Tables, sites should refer to the "Guide For Estimating Severity Grade" located above.
- Criteria are generally grouped by body system. Some protocols may have additional protocol-specific grading criteria, which will supersede the use of these tables for specified criteria.

Item	Grade 1	Grade 2	Grade 3	Grade 4
**Hematology**				
Hemoglobin	9.5-10.5 gm/dL	8.0-9.4 gm/dL	6.5-7.9 gm/dL	<6.5 gm/dL
Absolute Neutrophil Count	1000-1500/mm³	750-999/mm³	500-749/mm³	<500/mm³
Platelets	75000-99000/mm³	50000-74999/mm³	20000-49000/mm³	<20000/mm³
Prothrombin Time (PT)	≥1.01 to ≤1.25 x ULN	>1.25 to ≤1.50 x ULN	>1.50 to ≤3.00 x ULN	>3.00 x ULN
Activated Partial Thromboplastin (aPPT)	≥1.01 to ≤1.66 x ULN	>1.66 to ≤2.33 x ULN	>2.33 to ≤3.00 x ULN	>3.00 x ULN
Fibrinogen	≥0.75 to ≤0.99 x LLN	≥0.50 to <0.75 x LLN	≥0.25 to <0.50 x LLN	<0.25 x LLN
Fibrin Split Product	20-40 mcg/mL	41-50 mcg/mL	51-60 mcg/mL	>60 mcg/mL
Methemoglobin	5.0-9.9%	10.0-14.9%	15.0-19.9%	>20.0%
**Liver Enzymes**				
AST (SGOT)	≥1.25 to ≤2.50 x ULN	>2.50 to ≤5.00 x ULN	>5.00 to ≤10.00 x ULN	>10.00 x ULN
ALT (SGPT)	≥1.25 to ≤2.50 x ULN	>2.50 to ≤5.00 x ULN	>5.00 to ≤10.00 x ULN	>10.00 x ULN
GGT	≥1.25 to ≤2.50 x ULN	>2.50 to ≤5.00 x ULN	>5.00 to ≤10.00 x ULN	>10.00 x ULN
Alkaline Phosphatase	≥1.25 to ≤2.50 x ULN	>2.50 to ≤5.00 x ULN	>5.00 to ≤10.00 x ULN	>10.00 x ULN
Amylase	≥1.1 to ≤1.5 x ULN	>1.5 to ≤2.0 x ULN	>2.0 to ≤5.0 x ULN	>5.0 x ULN
**Chemistries**				
Hyponatremia	130-135 mEq/L	123-129 mEq/L	116-122 mEq/L	<116 mEq/L or mental status changes or seizures
Hypernatremia	146-150 mEq/L	151-157 mEq/L	158-165 mEq/L	>165 mEq/L or mental status changes or seizures
Hypokalemia	3.0-3.4 mEq/L	2.5-2.9 mEq/L	2.0-2.4 mEq/L or intensive replacement Rx required or hospitalization required	<2.0 mEq/L or paresis or ileus or life-threatening arrhythmia
Hyperkalemia	5.6-6.0 mEq/L	6.1-6.5 mEq/L	6.6-7.0 mEq/L	>7.0 mEq/L or life-threatening arrhythmia
Hypoglycemia	55-64 mg/dL	40-54 mg/dL	30-39 mg/dL	<30 mg/dL or mental status changes or coma
Hyperglycemia (note if fasting)	116-160 mg/dL	161-250 mg/dL	251-500 mg/dL	>500 mg/dL or ketoacidosis or seizures
Hypocalcemia (corrected for albumin)	8.4-7.8 mg/dL	7.7-7.0 mg/dL	6.9-6.1 mg/dL	<6.1 mg/dL or life threatening arrhythmia or tetany

Item	Grade 1	Grade 2	Grade 3	Grade 4
Hypercalcemia (corrected for albumin)	10.6-11.5 mg/dL	11.6-12.5 mg/dL	12.6-13.5 mg/dL	>13.5 mg/dL life-threatening arrhythmia
Hypomagnesemia	1.4-1.2 mEq/L	1.1-0.9 mEq/L	0.8-0.6 mEq/L	<0.6 mEq/L or life-threatening arrhythmia
Hypophosphatemia	2.0-2.4 mg/dL	1.5-1.9 mg/dL or replacement Rx required	1.0-1.4 mg/dL intensive Rx or hospitalization required	<1.0 mg/dL or life-threatening arrhythmia
Hyperbilirubinemia	≥1.1 to ≤1.5 x ULN	>1.5 to ≤2.5 x ULN	>2.5 to ≤5.0 x ULN	>5.0 x ULN
Lipase[a]	≥1.1 to ≤1.5 x ULN	>1.5 to ≤3.0 x ULN	>3.0 to ≤5.0 x ULN	>5.0 x ULN
BUN	≥1.25 to ≤2.50 x ULN	>2.50 to ≤5.00 x ULN	>5.00 to ≤10.00 x ULN	>10.00 x ULN
Creatinine	≥1.1 to ≤1.5 x ULN	>1.5 to ≤3.0 x ULN	>3.0 to ≤6.0 x ULN	>6.0 x ULN or required dialysis
**Urinalysis**				
Proteinuria	1+ or <0.3% or <3g/L or 200 mg – 1 gm loss/day	2-3 + or 0.3-1.0% or 3-10 g/L 1-2 gm loss/day	4+ or >1.0% or >10 g/L 2-3.5 gm loss/day	nephrotic syndrome or >3.5 gm loss/day
Hematuria	microscopic only	gross, no clots	gross + clots	obstructive or required transfusion
**Cardiac Dysfunction**				
Cardiac Rhythm	-	asymptomatic, transient signs, no Rx required	recurrent/persistent; no Rx required	requires Rx
Hypertension	transient inc. >20 mm; no Rx	recurrent, chronic, > 20 mm, Rx required	requires acute Rx; no hospitalization	requires hospitalization
Hypotension	transient orthostatic hypotension, no Rx	symptoms correctable with oral fluids Rx	requires IV fluids; no hospitalization required	requires hospitalization
Pericarditis	minimal effusion	mild/moderate asymptomatic effusion, no Rx	symptomatic effusion; pain; ECG changes	tamponade; pericardiocentesis or surgery required
Hemorrhage, Blood Loss	microscopic/occult	mild, no transfusion	gross blood loss; 1-2 units transfused	massive blood loss; >3 units transfused

a. Revised by the sponsor

Item	Grade 1	Grade 2	Grade 3	Grade 4
**Respiratory**				
Cough	Transient; no Rx	treatment associated cough local Rx	uncontrolled	-
Bronchospasm, Acute	transient; no Rx <80-70% FEV_1 (or peak flow)	requires Rx normalizes with bronchodilator; FEV_1 50-70% (or peak flow)	no normalization with bronchodilator; FEV_1 25-50% (or peak flow retractions)	cyanosis: FEV_1 <25% (or peak flow) or intubated
**Gastrointestinal**				
Stomatitis	mild discomfort; no limits on activity	some limits on eating/drinking	eating/talking very limited	requires IV fluids
Nausea	mild discomfort; maintains reasonable intake	moderate discomfort; intake decreased significantly; some activity limited	severe discomfort; no significant intake; activities limited	minimal fluid intake
Vomiting	transient emesis	occasional/moderate vomiting	orthostatic hypotension or IV fluids required	hypotensive shock or hospitalization required for IV fluid therapy
Constipation	mild	moderate	severe	distensions w/vomiting
Diarrhea	transient 3-4 loose stools/day	5-7 loose stools/day	orthostatic hypotension or >7 loose stools/day or required IV fluids	hypotensive shock or hospitalization for IV fluid therapy required
**Neuro & Neuromuscular**				
Neuro-Cerebellar	slight incoordination dysdiadochokinesis	intention tremor, dysmetria, slurred speech; nystagmus	locomotor ataxia	incapacitated
Mood	mild anxiety or depression	moderate anxiety or depression and therapy required	severe anxiety or depression or mania; needs assistance	acute psychosis; incapacitated, requires hospitalization
Neuro Control (ADL = activities of daily living)	mild difficulty concentrating; no Rx; mild confusion/agitation; ADL unaffected	moderate confusion/agitation some limitation of ADL; minimal Rx	severe confusion/agitation needs assistance for ADL; therapy required	toxic psychosis; hospitalization
Muscle Strength	subjective weakness no objective symptoms/ signs	mild objective signs/symptoms no decrease in function	objective weakness function limited	paralysis

Item	Grade 1	Grade 2	Grade 3	Grade 4
**Other Parameters**				
Fever: oral, >12 hours	37.7-38.5 °C or 100.0-101.5 °F	38.6-39.5 °C or 101.6-102.9 °F	39.6-40.5 °C or 103-105 °F	>40 °C or >105 °F
Headache	mild, no Rx therapy	transient, moderate; Rx required	severe; responds to initial narcotic therapy	intractable; required repeated narcotic therapy
Fatigue	no decrease in ADL	normal activity decreased 25-50%	normal activity decreased >50% can't work	unable to care for self
Allergic Reaction	pruritus without rash	localized urticaria	generalized urticaria; angioedema	anaphylaxis
Local Reaction	tenderness or erythema	induration <10 cm or phlebitis or inflammation	induration >10 cm or ulceration	necrosis
Mucocutaneous	erythema; pruritus	diffuse, maculo papular rash, dry desquamation	vesiculation, moist desquamation, or ulceration	exfoliative dermatitis, mucous membrane involvement or erythema, multiforme or suspected Stevens-Johnson or necrosis requiring surgery

Attachment 3: Hepatitis C Symptom & Impact Questionnaire (HCV-SIQ v3)

This questionnaire asks how you were doing over the past 7 days (including today).

Please read each question carefully then mark ☑ the answer that best describes **how you have felt due to your <u>Hepatitis C or its treatment</u> over the past 7 days including today**.

There are no right or wrong answers. If you want to change your answer, cross out your original answer and mark ☑ the answer that best describes your experience.

**PART I: Symptoms**					
Please describe **how severe** each **symptom** listed below was for you **on average over the past 7 days (including today)**. If you did not experience the symptom during the past 7 days, mark ☑ "Not at all".					
	**Not at all**	**A little**	**Somewhat**	**Very**	**Extremely**
**1.** Sore or achy muscles or joints	☐ 0	☐ 1	☐ 2	☐ 3	☐ 4
**2.** Feverish (for example feeling hot, cold, sweating)	☐ 0	☐ 1	☐ 2	☐ 3	☐ 4
**3.** Headache	☐ 0	☐ 1	☐ 2	☐ 3	☐ 4
**4.** Queasy or nauseous	☐ 0	☐ 1	☐ 2	☐ 3	☐ 4
**5.** Stomach pain or cramps	☐ 0	☐ 1	☐ 2	☐ 3	☐ 4
**6.** Shortness of breath	☐ 0	☐ 1	☐ 2	☐ 3	☐ 4
**7.** Tiredness	☐ 0	☐ 1	☐ 2	☐ 3	☐ 4
**8.** Physically weak	☐ 0	☐ 1	☐ 2	☐ 3	☐ 4
**9.** Easily irritated	☐ 0	☐ 1	☐ 2	☐ 3	☐ 4
**10.** Sad or depressed	☐ 0	☐ 1	☐ 2	☐ 3	☐ 4
**11.** Worried or anxious	☐ 0	☐ 1	☐ 2	☐ 3	☐ 4
**12.** Trouble remembering things	☐ 0	☐ 1	☐ 2	☐ 3	☐ 4
**13.** Trouble thinking clearly or concentrating	☐ 0	☐ 1	☐ 2	☐ 3	☐ 4
**14.** Problems getting to sleep or staying asleep	☐ 0	☐ 1	☐ 2	☐ 3	☐ 4
**15.** Dry or itchy skin	☐ 0	☐ 1	☐ 2	☐ 3	☐ 4
**16.** Tender or irritated skin	☐ 0	☐ 1	☐ 2	☐ 3	☐ 4
**17.** Pain or burning near anus	☐ 0	☐ 1	☐ 2	☐ 3	☐ 4
**18.** Hair loss	☐ 0	☐ 1	☐ 2	☐ 3	☐ 4
**19.** Feeling faint or dizzy	☐ 0	☐ 1	☐ 2	☐ 3	☐ 4
**20.** Soreness or swelling where medicine was injected	☐ 0	☐ 1	☐ 2	☐ 3	☐ 4
**21.** Dry mouth	☐ 0	☐ 1	☐ 2	☐ 3	☐ 4
**22.** Ringing or buzzing sound in ears	☐ 0	☐ 1	☐ 2	☐ 3	☐ 4

How many of the past 7 days have you had the following symptoms?	0 days	1-2 days	3-4 days	5-6 days	Every day
**23.** Diarrhea (very loose or liquid stools)	☐0	☐1	☐2	☐3	☐4
**24.** Loss of appetite/did not feel like eating	☐0	☐1	☐2	☐3	☐4

**Over the past 7 days**, did you notice that ...	Yes	No
**25.** Things tasted bad or had little flavor	☐0	☐1
**26.** Your hair or nails felt dry, looked dull, or tended to break easily	☐0	☐1

PART 2: Impact on Daily Life

27.Over the past 7 days, how much did your **health limit you doing things** you needed to do? (mark one answer only)

Not at all limited	A little limited	Somewhat limited	Very limited
**I did everything** I needed to do ☐0	**I did most things** I needed to do ☐1	**I could not do many things** I needed to do ☐2	**I could not do anything** I needed to do ☐3

**28.**Are you in **school or working in a job for pay?**	Yes	No
	☐0 **Please answer questions 29 and 30**	☐1 **<u>Do not</u> answer questions 29 or 30**
**29. Over the past 7 days, how many hours did you miss from work or school** <u>because of your health</u>? Please include time you missed to go to **your** doctor appointments, to get **your** medicines, or because **you did not feel well**. If you were <u>not</u> scheduled to work or go to school during the past 7 days, enter "0".	Hours missed from work or school in past 7 days _________hours	
**30. Over the past 7 days**, **how many hours when you were working or in school did you not get much done** <u>because of your health</u>? If you were <u>not</u> scheduled to work or go to school during the past 7 days, enter"0".	Hours you did not get much done at work or school in past 7 days __________ hours	

Questionnaire Complete

Thank you. Please return this form to the person who gave it to you.

Attachment 4: Work Productivity and Activity Impairment:Hepatitis C Questionnaire (WPAI:Hepatitis C)

Work Productivity and Activity Impairment Questionnaire, Hepatitis C, V2.0 (WPAI:Hepatitis C)

The following questions ask about the effect of your hepatitis C on your ability to work and perform regular activities. *Please fill in the blanks or circle a number, as indicated.*

1) Are you currently employed (working for pay)? _____NO ____ YES

If NO, check "NO" and skip to question 6.

The next questions are about the **past seven days**, not including today.

2) During the past seven days, how many hours did you miss from work because of problems associated with your hepatitis C? *Include hours you missed on sick days, times you went in late, left early, etc., because of* hepatitis C. *Do not include time you missed to participate in this study.*

_____ HOURS

3) During the past seven days, how many hours did you miss from work because of any other reason, such as vacation, holidays, time off to participate in this study?

______HOURS

4) During the past seven days, how many hours did you actually work?

______HOURS *(If "0", skip to question 6)*

5) During the past seven days, how much did hepatitis C affect your productivity while you were working?

Think about days you were limited in the amount or kind of work you could do, days you accomplished less than you would like, or days you could not do your work as carefully as usual. If hepatitis C affected your work only a little, choose a low number. Choose a high number if hepatitis C affected your work a great deal.

Consider only how much hepatitis C affected productivity while you were working.

Hepatitis C had no effect on work	0	1	2	3	4	5	6	7	8	9	10	Hepatitis C completely prevented me from working

CIRCLE A NUMBER

6) During the past seven days, how much did hepatitis C affect your ability to do your regular daily activities, other than work at a job?

By regular activities, we mean the usual activities you do, such as work around the house, shopping, child care, exercising, studying, etc. Think about times you were limited in the amount or kind of activities you could do and times you accomplished less than you would like. If hepatitis C affected your activities only a little, choose a low number. Choose a high number if hepatitis C affected your activities a great deal.

Consider only how much hepatitis C affected your ability to do your regular daily activities, other than work at a job.

Hepatitis C had no effect on my daily activities	0	1	2	3	4	5	6	7	8	9	10	Hepatitis C completely prevented me from doing my daily activities

CIRCLE A NUMBER

WPAI:Hep C V2.0 (US English)

Attachment 5: EuroQol 5-Dimension Questionnaire (EQ-5D)

Health Questionnaire

English version for the UK

UK (English) v.2 © 2009 EuroQol Group. EQ-5D™ is a trade mark of the EuroQol Group

Under each heading, please tick the ONE box that best describes your health TODAY

MOBILITY

I have no problems in walking about ❑
I have slight problems in walking about ❑
I have moderate problems in walking about ❑
I have severe problems in walking about ❑
I am unable to walk about ❑

SELF-CARE

I have no problems washing or dressing myself ❑
I have slight problems washing or dressing myself ❑
I have moderate problems washing or dressing myself ❑
I have severe problems washing or dressing myself ❑
I am unable to wash or dress myself ❑

USUAL ACTIVITIES *(e.g., work, study, housework, family or leisure activities)*

I have no problems doing my usual activities ❑
I have slight problems doing my usual activities ❑
I have moderate problems doing my usual activities ❑
I have severe problems doing my usual activities ❑
I am unable to do my usual activities ❑

PAIN / DISCOMFORT

I have no pain or discomfort ❑
I have slight pain or discomfort ❑
I have moderate pain or discomfort ❑
I have severe pain or discomfort ❑
I have extreme pain or discomfort ❑

ANXIETY / DEPRESSION

I am not anxious or depressed ❑
I am slightly anxious or depressed ❑
I am moderately anxious or depressed ❑
I am severely anxious or depressed ❑
I am extremely anxious or depressed ❑

UK (English) v.2 © 2009 EuroQol Group. EQ-5D™ is a trade mark of the EuroQol Group

- We would like to know how good or bad your health is TODAY.
- This scale is numbered from 0 to 100.
- 100 means the <u>best</u> health you can imagine.
  0 means the <u>worst</u> health you can imagine.
- Mark an X on the scale to indicate how your health is TODAY.
- Now, please write the number you marked on the scale in the box below.

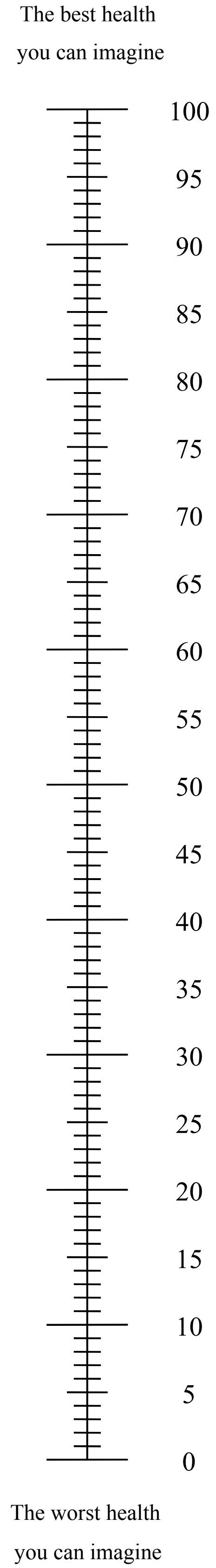

YOUR HEALTH TODAY = | |
|---|

UK (English) v.2 © 2009 EuroQol Group. EQ-5D™ is a trade mark of the EuroQol Group

Attachment 6: Fatigue Severity Scale (FSS)

Below are a series of statements regarding your fatigue. By fatigue we mean a sense of tiredness, lack of energy or total body give-out. Please read each statement and choose a number from 1 to 7, where 1 indicates you completely disagree with the statement and 7 indicates you completely agree. Please answer these questions as they apply to the past TWO WEEKS.

	Completely disagree						Completely agree
1. My motivation is lower when I am fatigued.	1	2	3	4	5	6	7
2. Exercise brings on my fatigue.	1	2	3	4	5	6	7
3. I am easily fatigued.	1	2	3	4	5	6	7
4. Fatigue interferes with my physical functioning.	1	2	3	4	5	6	7
5. Fatigue causes frequent problems for me.	1	2	3	4	5	6	7
6. My fatigue prevents sustained physical functioning.	1	2	3	4	5	6	7
7. Fatigue interferes with carrying out certain duties and responsibilities.	1	2	3	4	5	6	7
8. My fatigue is very disabling.	1	2	3	4	5	6	7
9. Fatigue interferes with my work, family or social life.	1	2	3	4	5	6	7

Attachment 7: Center For Epidemiologic Studies-Depression Scale (CES-D)

Center for Epidemiologic Studies Depression Scale (CES-D), NIMH

Below is a list of the ways you might have felt or behaved. Please tell me how often you have felt this way during the past week.

	During the Past Week			
	Rarely or none of the time (less than 1 day)	Some or a little of the time (1-2 days)	Occasionally or a moderate amount of time (3-4 days)	Most or all of the time (5-7 days)
1. I was bothered by things that usually don't bother me.	☐	☐	☐	☐
2. I did not feel like eating; my appetite was poor.	☐	☐	☐	☐
3. I felt that I could not shake off the blues even with help from my family or friends.	☐	☐	☐	☐
4. I felt I was just as good as other people.	☐	☐	☐	☐
5. I had trouble keeping my mind on what I was doing.	☐	☐	☐	☐
6. I felt depressed.	☐	☐	☐	☐
7. I felt that everything I did was an effort.	☐	☐	☐	☐
8. I felt hopeful about the future.	☐	☐	☐	☐
9. I thought my life had been a failure.	☐	☐	☐	☐
10. I felt fearful.	☐	☐	☐	☐
11. My sleep was restless.	☐	☐	☐	☐
12. I was happy.	☐	☐	☐	☐
13. I talked less than usual.	☐	☐	☐	☐
14. I felt lonely.	☐	☐	☐	☐
15. People were unfriendly.	☐	☐	☐	☐
16. I enjoyed life.	☐	☐	☐	☐
17. I had crying spells.	☐	☐	☐	☐
18. I felt sad.	☐	☐	☐	☐
19. I felt that people dislike me.	☐	☐	☐	☐
20. I could not get "going."	☐	☐	☐	☐

SCORING: zero for answers in the first column, 1 for answers in the second column, 2 for answers in the third column, 3 for answers in the fourth column. The scoring of positive items is reversed. Possible range of scores is zero to 60, with the higher scores indicating the presence of more symptomatology.

Attachment 8: Visit Schedule for Rash Management

This visit schedule summarizes the visits and assessments to be performed in case of rash. At the investigator's discretion, additional visits and assessments can be performed. **Local laboratory blood sample assessments will be documented/collected as described in the text above.**

	Grade 1 Rash	Grade 2 Rash	Grade 3 or 4 Rash
**Day 0[a]**	• Study medication **MAY** be **CONTINUED.**<br>• Unscheduled visit (on site) for initial rash evaluation **REQUIRED**.<br>• Assessment of safety blood sample by local laboratory **REQUIRED**.	• Study medication **MAY** be **CONTINUED**.<br>• Unscheduled visit (on site) for initial rash evaluation **REQUIRED**.<br>• Assessment of safety blood sample by local laboratory **REQUIRED**.<br>• Referral to dermatologist **OPTIONAL** (preferably within 24h after onset of rash, if performed).	• Study medication **MUST** be permanently **DISCONTINUED**. Rechallenge is **NOT ALLOWED**.<br>• Unscheduled visit (on site) for initial rash evaluation as soon as possible after the subject contacts the investigator to report the event **REQUIRED**.<br>• Assessment of safety blood sample by local laboratory **REQUIRED**.<br>• Referral to dermatologist **REQUIRED** (preferably within 24h after onset of rash).<br>• Biopsy **REQUIRED** for grade 4 rash (as soon as possible after onset of rash). Biopsy at the dermatologist's discretion for grade 3 rash.
**Day 1**		•	• Follow-up visit (on site) **REQUIRED**.<br>• Assessment of safety blood sample by local laboratory **REQUIRED**.
**Further Visits**	• Appropriate follow-up visits at the investigator's discretion until resolution of rash or until a clinically stable endpoint is reached.[2]	• Appropriate follow-up visits at the investigator's discretion until resolution of rash or until a clinically stable endpoint is reached.[2]	• Appropriate follow-up **REQUIRED** until resolution of rash or until a clinically stable endpoint is reached.

[a] Note that Day 0 of the rash is the first day of Investigator assessment and not the first day of rash as reported by the subject.
[2] In case rash progresses from a grade 1 or a grade 2 to a higher grade, start follow-up schedule for grade 2, 3 or 4 rash as appropriate.

INVESTIGATOR AGREEMENT

I have read this protocol and agree that it contains all necessary details for carrying out this study. I will conduct the study as outlined herein and will complete the study within the time designated.

I will provide copies of the protocol and all pertinent information to all individuals responsible to me who assist in the conduct of this study. I will discuss this material with them to ensure that they are fully informed regarding the study drug, the conduct of the study, and the obligations of confidentiality.

Coordinating Investigator (where required):

Name (typed or printed): ______________________________

Institution and Address: ______________________________

____

____

____

Signature: ______________________________ Date: ______________________________

(Day Month Year)

Principal (Site) Investigator:

Name (typed or printed): ______________________________

Institution and Address: ______________________________

____

____

____

Telephone Number: ______________________________

Signature: ______________________________ Date: ______________________________

(Day Month Year)

Sponsor's Responsible Medical Officer:

Name (typed or printed): Dr. Michael Von Poncet

Institution: Janssen Therapeutics EMEA

Signature: electronic signature appended at the end of the protocol Date: ______________________________

(Day Month Year)

Note: If the address or telephone number of the investigator changes during the course of the study, written notification will be provided by the investigator to the sponsor, and a protocol amendment will not be required.

LAST PAGE
