# Supplementary material for: An Open-Label Trial of 12-Week Simeprevir plus Peginterferon/Ribavirin (PR) in Treatment-Naïve Patients with Hepatitis C Virus (HCV) Genotype 1 (GT1)
Source: PLoS One. 2016 Jul 18;11(7):e0158526. doi: 10.1371/journal.pone.0158526 (PMC4948848; doi:10.1371/journal.pone.0158526)
Supplement: S1 Dataset — (ZIP) [file pone.0158526.s009.zip › LVIBL01.rtf]

LVIBL01:	Listing of Baseline Polymorphisms; Intent-to-treat (Study TMC435HPC3014)
Treatment Group = Simeprevir 12Wks 150 mg PR12/24 - HCV Genotype = Genotype 1 - Treatment Duration = 12 Weeks Treatment - HCV Geno/Subtype = 1a/other	
Subject ID	Subtype
(LIPA)	Subtype
(Trugene)	Visit	Time Point	Date	Day in Study	Sequenced Region	Region	Polymorphism	
30140001	1a		Baseline	Baseline	17OCT2013	1	NS3/4A	NS3	T40A	
									P67S	
									S91A	
									L153I	
									N174G *	
									S189T	
									S196C	
									F197Y	
									V248I	
									A315A/V	
									V318T	
									S332P	
									P334S	
									V358A	
									K372R	
									A379A/G	
									V382A/T	
									L384M	
									I386V	
									S410A	
									V609I	
									I615V	
								NS4A	V43A	
									Q46K	
30140002	1a		Baseline	Baseline	03OCT2013	1	NS3PART	NS3	V33I	
									T40A	
									V51A/V	
									P67S	
									S91A	
									L153I	
30140003	1a		Baseline	Baseline	11OCT2013	1	NS3/4A	NS3	V33I	
									T40A	
									N49N/S	
									I72L	
									S91A	
									L153I	
									N174G *	
									T185S	
									F197Y	
									V248I	
									V318T	
									S332P	
									P334S	
									V358A	
									I386V	
									S410A	
									V609I	
									I615V	
								NS4A	K34R	
									Q46R	
30140004	1a		Baseline	Baseline	01OCT2013	1	NS3PART	NS3	Q28A/V	
									T40A	
									S66T	
									P67S	
									S91A	
									L153I	
									N174G *	
30140008	1a		Baseline	Baseline	17OCT2013	1	NS3/4A	NS3	L14F/L	
									V29T	
									Q34L	
									T40A	
									V51A	
									Q80K *	
									S91T	
									L153I	
									T178A	
									A192V	
									V248I	
									S332P	
									V358A/V	
									I386V	
									S410A	
									K469K/R	
									V490I/V	
									F557F/L	
									I586T	
									I615V	
								NS4A	I29V	
									K34R	
									Q46R	
30140010	1a		Baseline	Baseline	10OCT2013	1	NS3/4A	NS3	V33I/V	
									T46S	
									P67P/S	
									S91A	
									L153I	
									V248I	
									S332P	
									V358A	
									L384M	
									S410A	
									F418Y	
									F557L	
									I615V	
								NS4A	V43A	
									Q46R	
30140013	1a		Baseline	Baseline	06NOV2013	1	NS3PART	NS3	T40A	
									P67S	
									S91A	
									L153I	
									N174S *	
30140016	1a		Baseline	Baseline	25OCT2013	1	NS3/4A	NS3	I18V	
									T40A	
									S91A	
									L153I	
									A192V	
									V248I	
									S332P	
									I347I/V	
									V358A	
									I386V	
									S410A	
									F418F/Y	
									F557L	
									I586T	
									V609I	
								NS4A	I29V	
									Q46R	
30140017	1a		Baseline	Baseline	08NOV2013	1	NS3/4A	NS3	T40A	
									P67S	
									S91A	
									S122G *	
									L153I	
									N174S *	
									T185S	
									F197Y	
									V248I	
									V318T	
									S332P	
									V358A	
									K372R	
									I386V	
									S410A	
									F418Y	
									I615V	
								NS4A	Q46R	
30140019	1a		Baseline	Baseline	01NOV2013	1	NS3PART	NS3	V29T	
									T40A	
									Q80K *	
									S91A	
									L153I	
30140028	1a		Baseline	Baseline	05NOV2013	1	NS3PART	NS3	I18V	
									V33I/V	
									T40A	
									I64I/L	
									P67S	
									S91A	
									I114V	
									A147A/S	
									L153I	
									D168D/E *	
									N174S *	
30140030	1a		Baseline	Baseline	29OCT2013	1	NS3/4A	NS3	T40A	
									I72V	
									S91A	
									L153I	
									A192A/V	
									V248I	
									I252V	
									V329I/V	
									S332P	
									E357D/E	
									S410A	
									A455S	
									F557L	
									I586I/N/T	
									I615I/V	
								NS4A	K34R	
									Q46R	
30140033	1a		Baseline	Baseline	13NOV2013	1	NS3/4A	NS3	Q28E	
									T40A	
									S66T	
									P67S	
									S91A	
									L153I	
									N174G *	
									F197Y	
									V248I	
									I300V	
									V318T	
									S332P	
									V358A	
									I386V	
									V399T	
									T402L	
									S410A	
									F557L	
									V609I	
									T610I	
									I615V	
								NS4A	Q46R	
30140036	1a		Baseline	Baseline	12NOV2013	1	NS3/4A	NS3	T40A	
									P67S	
									Q89H/Q	
									S91A	
									S122G *	
									L153I	
									N174S *	
									T185S	
									S196A	
									F197Y	
									V248I	
									P264S	
									V318T	
									S332P	
									V358T	
									I386V	
									S410A	
									F418Y	
									I615V	
30140038	1a		Baseline	Baseline	13NOV2013	1	NS3PART	NS3	Q28G	
									T40A	
									I64L	
									P67S	
									S91A	
									L153I	
									N174G *	
30140043	1a		Baseline	Baseline	18NOV2013	1	NS3/4A	NS3	T40A	
									P67S	
									S91A	
									L153I	
									N174G *	
									T185S	
									S196C	
									F197Y	
									V248I	
									A315V	
									V318T	
									S332A	
									V358A	
									I386V	
									V399I	
									T402A	
									S410A	
									V609I	
									I615V	
								NS4A	Q46R	
30140044	1a		Baseline	Baseline	21NOV2013	1	NS3/4A	NS3	T40A	
									T46S	
									S91A	
									L153I	
									N174S *	
									V248I	
									V329I/V	
									S332P	
									V358T	
									S410A	
									F418F/Y	
									F557L	
								NS4A	Q46R	
30140050	1a		Baseline	Baseline	15NOV2013	1	NS3/4A	NS3	I18I/V	
									Q34L	
									V36L *	
									T40A	
									V71I	
									Q80K *	
									P86S	
									S91A	
									L153I	
									A315A/V	
									L317L/M	
									S332P	
									A379A/T	
									I386V	
									S410A	
									T459S	
									H541H/R	
								NS4A	I29V	
									I38V	
									Q46R	
30140052	1a		Baseline	Baseline	12NOV2013	1	NS3/4A	NS3	P67S	
									S91A	
									L153I	
									N174S *	
									V248I	
									S332P	
									P334H	
									I386V	
									S410A	
									F418Y	
									V609L	
									I615V	
								NS4A	I37V	
									Q46R	
30140053	1a		Baseline	Baseline	19NOV2013	1	NS3/4A	NS3	T40A	
									S91A	
									S122G/S *	
									L153I	
									N174N/S *	
									F184F/Y	
									T185S	
									S196C	
									F197Y	
									V248I	
									D249E	
									V318T	
									S332P	
									V358A	
									A383A/G	
									I386V	
									T402A	
									S410A	
									T459S	
									F557F/L	
									I615V	
								NS4A	Q46R	
30140054	1a		Baseline	Baseline	26NOV2013	1	NS3PART	NS3	L14M	
									Q28E	
									P67S	
									S91A	
									S128T	
									A147S	
									L153I	
									N174S *	
30140055	1a		Baseline	Baseline	08NOV2013	1	NS3PART	NS3	T40A	
									S91A	
									I114V	
									L153I	
									N174S *	
30140062	1a		Baseline	Baseline	06DEC2013	1	NS3PART	NS3	N27D	
									T46S	
									S91A	
									L153I	
30140065	1a		Baseline	Baseline	02DEC2013	1	NS3PART	NS3	T40A	
									T46S	
									S91A	
									L153I	
									N174S *	
30140074	1a		Baseline	Baseline	27NOV2013	1	NS3PART	NS3	T40A	
									P67P/S	
									I72M	
									D79E	
									S91A	
									L94M	
									L153I	
									N174S *	
30140075	1a		Baseline	Baseline	27NOV2013	1	NS3PART	NS3	V33I	
									T40A	
									T54S *	
									V55I *	
									S91A	
									L153I	
									N174N/S *	
30140080	1a		Baseline	Baseline	09DEC2013	1	NS3/4A	NS3	T46S	
									S91A	
									T95I	
									L153I	
									N174S *	
									S332P	
									V358A	
									K360R	
									A379A/G	
									I386V	
									S410A	
									F418Y	
									V609I	
								NS4A	Q46R	
30140102	1a		Baseline	Baseline	19NOV2013	1	NS3/4A	NS3	S91A	
									G124A/G	
									L153I	
									I170V *	
									N174S *	
									V248I	
									S332P	
									V358T	
									S410A	
									F418Y	
									F475Y	
									F557L	
									I615I/V	
								NS4A	Q46R	
30140105	1a		Baseline	Baseline	19DEC2013	1	NS3/4A	NS3	I18I/V	
									T40A	
									P67S	
									S91A	
									T98A	
									G124A/G	
									L153I	
									N174S *	
									S189S/T	
									S196C	
									F197Y	
									V248I	
									A315V	
									V318T	
									S332P	
									V358A	
									I386V	
									S410A	
									K469R	
									I615V	
								NS4A	Q46R	
30140107	1a		Baseline	Baseline	12DEC2013	1	NS3/4A	NS3	Q28G	
									T40A	
									L153I	
									A192V	
									S196T	
									F197Y	
									V248I	
									S332P	
									V358A	
									S410A	
									F557L	
									V609I	
									I615V	
								NS4A	I29V	
30140109	1a		Baseline	Baseline	28NOV2013	1	NS3/4A	NS3	V29A	
									T40A	
									I64L	
									Q80K *	
									Q89P	
									S91T	
									V151A	
									L153I	
									A192V	
									V248I	
									S332P	
									K371R	
									I386V	
									S410A	
									K469R	
									I615V	
								NS4A	I29V	
									I37V	
									Q46R	
30140112	1a		Baseline	Baseline	13DEC2013	1	NS3PART	NS3	I18I/V	
									T40A	
									T46S	
									P67S	
									I114I/V	
									G124A	
									A147A/S	
									V151A/V	
									L153I	
									N174G/S *	
30140126	1a		Baseline	Baseline	10DEC2013	1	NS3PART	NS3	T40A	
									I64L	
									P67S	
									V83I	
									S91A	
									L153I	
									N174G *	
30140129	1a		Baseline	Baseline	13DEC2013	1	NS3/4A	NS3	T40A	
									P67S	
									S91A	
									I114V	
									R117H	
									L153I	
									N174S *	
									T185S	
									S196C	
									F197Y	
									V248I	
									A315V	
									V318T	
									S332P	
									V358A	
									I386V	
									S410A	
									F557L	
									I615V	
								NS4A	V30I	
30140136	1a		Baseline	Baseline	03DEC2013	1	NS3/4A	NS3	V33I	
									T40A	
									T61S	
									P67S	
									S91A	
									L153I	
									I170V *	
									N174S *	
									T185S	
									S196C	
									F197Y	
									V248I	
									I300I/V	
									S332P	
									V358A	
									I386V	
									S410A	
									F418F/Y	
									T435A	
									T449I	
									F557L	
									I615V	
								NS4A	Q46R	
30140139	1a		Baseline	Baseline	05DEC2013	1	NS3/4A	NS3	S91A	
									L153I	
									V248I	
									V329I	
									S332P	
									V358A/I/T/V	
									I386V	
									S410A	
									F418Y	
									V490I	
									F557L	
									I586T	
								NS4A	I37V	
									Q46R	
30140144	1a		Baseline	Baseline	12DEC2013	1	NS3/4A	NS3	T40A	
									I64L	
									P67S	
									Q89P	
									S91A	
									L153I	
									N174G *	
									T185S	
									S196C	
									F197Y	
									V248I	
									A315V	
									V318T	
									S332P	
									V358T	
									I386V	
									S410A	
									F418Y	
									F557L	
									I615V	
								NS4A	V30D	
									K34R	
									Q46K	
30140151	1a		Baseline	Baseline	06DEC2013	1	NS3/4A	NS3	T40A	
									T61S	
									R62K	
									I64L	
									P67S	
									Q89P/S	
									S91A	
									S122G/S *	
									L153I	
									N174G *	
									T185S	
									S196C	
									F197Y	
									K244R	
									V248I	
									D249E	
									V306A/V	
									A315A/V	
									V318T	
									S332P	
									I386V	
									S410A	
									F557L	
									V609I	
									I615V	
								NS4A	A14A/V	
									Q46R	
30140152	1a		Baseline	Baseline	02DEC2013	1	NS3PART	NS3	T40A	
									P67S	
									S91A	
									S122G *	
									L153I	
									N174S *	
30140161	1a		Baseline	Baseline	13DEC2013	1	NS3/4A	NS3	V29A	
									V36M/V *	
									T40A	
									Q80K *	
									S91T	
									T98A/T	
									G120G/S	
									S122G/S *	
									L153I	
									N174N/S *	
									K244K/R	
									V248I	
									I286I/V	
									L317M	
									S332P	
									A383S	
									S410A	
									F418Y	
									V490I/V	
									A497A/T	
									H541H/R	
									F557F/L	
									I586I/N	
								NS4A	A14A/T	
									I37V	
									Q46R	
30140164	1a		Baseline	Baseline	10DEC2013	1	NS3/4A	NS3	T40A	
									T61A	
									L153I	
									S263N	
									S332P	
									P334S	
									S410A	
									F418Y	
									I586T	
									V609I	
30140169	1a		Baseline	Baseline	13DEC2013	1	NS3/4A	NS3	I18I/V	
									P67S	
									S91A	
									S122G *	
									L153I	
									N174G *	
									F197Y	
									V248I	
									V318T	
									S332P	
									P334H	
									V358A/V	
									V382T	
									I386V	
									S410A	
									F418Y	
									A477T	
									F557L	
									P574L	
									V609I	
									I615V	
									V630I/V	
								NS4A	A14A/T	
									Q46R	
30140170	1a		Baseline	Baseline	19DEC2013	1	NS3PART	NS3	T40A	
									T46S	
									V83I/V	
									S91A	
									S122G *	
									L153I	
									N174S *	
30140174	1a		Baseline	Baseline	23DEC2013	1	NS3/4A	NS3	T40A	
									S91A	
									L153I	
									N174S *	
									V329I/V	
									S332P	
									V358A	
									A379T	
									L384M	
									S410A	
								NS4A	Q46R	
30140179	1a		Baseline	Baseline	23DEC2013	1	NS3/4A	NS3	T40S	
									T46S	
									A87A/V	
									Q89H	
									S91A	
									L153I	
									N174S *	
									L228I/L	
									V248I	
									S332P	
									V358T	
									L384L/M/V	
									S410A	
									F557L	
								NS4A	T2A/T	
									Q46R	
30140180	1a		Baseline	Baseline	16DEC2013	1	NS3/4A	NS3	Q28E	
									T40A	
									P67S	
									S91A	
									L153I	
									I170V *	
									N174S *	
									T185S	
									S196C	
									F197Y	
									V232G/V	
									V248I	
									A315V	
									V318T	
									S332P	
									V339I	
									A340G	
									V358T	
									I386V	
									S410A	
									F418Y	
									F475Y	
									F557F/L	
									V609I/T	
									I615V	
								NS4A	V30I	
									Q46R	
30140186	1a		Baseline	Baseline	30DEC2013	1	NS3/4A	NS3	V29A	
									T40A/V	
									Q80K *	
									S91A	
									L153I	
									V248I/V	
									I288I/M	
									V329I	
									S332P	
									V358A/I/T/V	
									I386I/V	
									V399A/I/T/V	
									S410A	
									F475Y	
									Y516F	
									I586I/N/T	
									V609I	
								NS4A	I29V	
									Q46R	

! Indicates an insertion; ^ Indicates a deletion
* Indicates polymorphisms at selected NS3 postions (36, 41, 43, 54, 55, 80, 107, 122, 132, 138, 155, 156, 158, 168, 169, 170, 174, 175)
Polymorphism are defined as changes from con1 (AJ238799) and H77 (AF009606) for hcv geno/subtype 1b and 1a/other, respectively.	
[LVIBL01.rtf] [\STAT\Analyses\Programs\FinalAnalysis\Final1\2.TLF\6.Virology\VIR_FA.sas] 23OCT2015, 17:02	

LVIBL01:	Listing of Baseline Polymorphisms; Intent-to-treat (Study TMC435HPC3014)
Treatment Group = Simeprevir 12Wks 150 mg PR12/24 - HCV Genotype = Genotype 1 - Treatment Duration = 12 Weeks Treatment - HCV Geno/Subtype = 1b	
Subject ID	Subtype
(LIPA)	Subtype
(Trugene)	Visit	Time Point	Date	Day in Study	Sequenced Region	Region	Polymorphism	
30140006	1b		Baseline	Baseline	26SEP2013	1	NS3PART	NS3	S7A	
									R26K	
									Y56F	
									T63A	
									I71V	
									S147L	
									V170I *	
30140007	1b		Baseline	Baseline	11OCT2013	1	NS3/4A	NS3	R26K	
									Y56F	
									Q80L *	
									Q86T	
									P89S	
									R117C	
									V170I *	
									S189T	
									I248V	
									T299S	
									S343N	
									L386I	
									I407V	
									M470R	
									D555E	
									V561T	
									T611L	
								NS4A	K34R	
									V43A	
									R46Q	
30140009	1b		Baseline	Baseline	08OCT2013	1	NS3/4A	NS3	S7A	
									R26K	
									S42T	
									V83F/V	
									S147L	
									K213R	
									I248V	
									I288M	
									T299S	
									S343N	
									T344I	
									T358A	
									A379T	
									L386I	
									D405N	
									I407V	
									M470R	
									T611L	
								NS4A	V26C	
									I37V	
									R46Q	
30140011	1b		Baseline	Baseline	25OCT2013	1	NS3/4A	NS3	S7A	
									L14F	
									R26K	
									V35M	
									V48I	
									S61A	
									V132I *	
									I248I/V	
									A263G	
									P264S	
									V339I	
									S343N	
									I407V	
									F418Y	
									M470G	
									T610A	
									T611L	
									I615V	
								NS4A	L10I	
									I37V	
									R46Q	
30140020	1b		Baseline	Baseline	08NOV2013	1	NS3/4A	NS3	R26K	
									H246Y	
									N251S	
									S343N	
									K371R	
									G383S	
									I407V	
									M470G	
									F475Y	
									V609I/V	
									T611L	
								NS4A	I37V	
									R46Q	
30140021	1b		Baseline	Baseline	07NOV2013	1	NS3/4A	NS3	S7A	
									R26K	
									A45G	
									T72I	
									Q86P	
									S122N *	
									V132I *	
									V170I *	
									S189A	
									K244T	
									I248V	
									S343N	
									I356L	
									T358A	
									K371R	
									G383A	
									D405N	
									I407V	
									S459T	
									M470R	
									S510T	
									D555E	
									V561T	
									Q580E	
									T611L	
									I615M	
									Y618L	
								NS4A	I30V	
									I37V	
									V43A	
30140024	1b		Baseline	Baseline	31OCT2013	1	NS3/4A	NS3	R26K	
									V35I	
									T46A	
									V48I	
									T63P	
									T72I	
									Q86P	
									T95S	
									V132I *	
									I248V	
									P264S	
									S297A	
									I303V	
									S343N	
									I407V	
									M470R	
									A544S	
									V561T	
									T610I	
									T611L	
30140025	1b		Baseline	Baseline	12NOV2013	1	NS3/4A	NS3	R26K	
									V48I	
									Y56F	
									T72I	
									Q86P	
									V132I *	
									N251G	
									P334H	
									S343N	
									T344I	
									T358A	
									I407V	
									T430V	
									M470R	
									T611L	
									I615V	
								NS4A	I37V	
30140027	1b		Baseline	Baseline	28OCT2013	1	NS3/4A	NS3	R26K	
									V51A	
									V170I *	
									S174A *	
									P264S	
									T295I	
									T299S	
									E337D	
									A340G	
									S342T	
									S343N	
									T344I	
									G383S	
									I407V	
									M470R	
									D555E	
									V561T	
									T611L	
								NS4A	I37V	
									R46Q	
									E47Q	
30140029	1b		Baseline	Baseline	29OCT2013	1	NS3/4A	NS3	S7A	
									R26K	
									T46A	
									V48I	
									Y56F	
									Q86P	
									H246Y	
									N251S	
									I265V	
									T299S	
									S343N	
									I347V	
									I356L	
									T358V	
									K371R	
									S382L	
									G383S	
									L386I	
									I407V	
									M470R	
									V561T	
									T611L	
								NS4A	I30V	
									R46Q	
30140031	1b		Baseline	Baseline	29OCT2013	1	NS3PART	NS3	I18V	
									R26K	
									V48I	
									P89S	
30140032	1b		Baseline	Baseline	29OCT2013	1	NS3/4A	NS3	S7A	
									I18V	
									R26K	
									V48I	
									Y56F	
									Q86P	
									I248V	
									T299S	
									S343N	
									T344I	
									I347I/V	
									T358A/V	
									L386I	
									I407V	
									M470R	
									V561T	
									I586T	
									T611L	
								NS4A	V43A	
									R46Q	
									E47A	
30140034	1b		Baseline	Baseline	06NOV2013	1	NS3/4A	NS3	R26K	
									V48I	
									R117H	
									V170I/V *	
									H246Y	
									I248V	
									P264S	
									T299S	
									S343N	
									T344I	
									I356L	
									T358A/I/T/V	
									S403A/T	
									I407V	
									M470R	
									S510T	
									S534G	
									T610I	
									T611L	
									A621T	
								NS4A	R46Q	
30140035	1b		Baseline	Baseline	08NOV2013	1	NS3/4A	NS3	R26K	
									Y56F	
									S101N	
									A150V	
									V170I *	
									I248V	
									T299S	
									S343N	
									T344I	
									G383S	
									I407V	
									F418Y	
									M470R	
									T477A	
									S510T	
									S534G	
									T611L	
									Y618L	
									V629I/V	
								NS4A	K34R	
									V43A	
30140039	1b		Baseline	Baseline	12NOV2013	1	NS3/4A	NS3	R26K	
									V48I	
									R117H/R	
									S147L	
									A150V	
									V151A	
									V172A/V	
									S174T *	
									E176G	
									T185S	
									I248V	
									P264S	
									I288M	
									T299S	
									S343N	
									T344I	
									G383A	
									I407V	
									M470Q	
									T611L	
									I615V	
								NS4A	I30V	
									I37L	
									V43I	
30140040	1b		Baseline	Baseline	21NOV2013	1	NS3/4A	NS3	S7A	
									R26K	
									K62R	
									T72I	
									I248V	
									P264S	
									S343N	
									T358A/T	
									G383S	
									I407V	
									M470R	
									V490I	
									T540P	
									T611L	
								NS4A	S22C	
									K34R	
									I37V	
									I38V	
									R46Q	
30140046	1b		Baseline	Baseline	12NOV2013	1	NS3/4A	NS3	R26K	
									P334S	
									S343N	
									T344I	
									K372R	
									L377F	
									G383A/G	
									I407V	
									K589R	
									T591A/T	
									V609I	
									I615A	
								NS4A	I30V	
									R46Q	
30140047	1b		Baseline	Baseline	13NOV2013	1	NS3PART	NS3	R26K	
									N49S	
									N77S	
									Q86P	
									V132I *	
									K136R	
30140048	1b		Baseline	Baseline	21NOV2013	1	NS3/4A	NS3	S7A	
									R26K	
									T72I	
									Q86P	
									V132I *	
									S189A	
									K244T	
									I248V	
									S343N	
									I356L	
									T358V	
									K371K/R	
									G383A	
									D405N	
									I407V	
									S459T	
									M470R	
									D555E	
									F557Y	
									V561T	
									T611L	
									Y618F	
								NS4A	V43A	
30140056	1b		Baseline	Baseline	05NOV2013	1	NS3/4A	NS3	S7A	
									I18V	
									R26K	
									S42T	
									V48I	
									Y56F	
									Q86A	
									V170A *	
									I248V	
									I288M	
									T299S	
									S343N	
									T402S	
									I407V	
									M470R	
									V511I	
									T610I	
									T611L	
								NS4A	I37V	
30140057	1b		Baseline	Baseline	12NOV2013	1	NS3/4A	NS3	S7A	
									I18V	
									R26K	
									Y56F	
									I114V	
									I248V	
									P264S	
									I288M	
									T299S	
									S343N	
									T344I	
									T358N	
									G383S	
									I407V	
									M470R	
									S488T	
									V609T	
									T611L	
								NS4A	R46Q	
30140058	1b		Baseline	Baseline	12NOV2013	1	NS3/4A	NS3	S7A	
									R26K	
									V48I	
									P89S	
									I153V	
									I248V	
									T299S	
									S343N	
									T344I	
									I356L	
									G383S	
									I407V	
									V451L	
									I472V	
									S534G	
									D555E	
									Q572K	
									T591M	
									H593Q	
									T611L	
									A621T	
								NS4A	I30N	
									I37V	
									I38V	
									R46Q	
30140061	1b		Baseline	Baseline	15NOV2013	1	NS3PART	NS3	S7A	
									V51T	
									I71V	
									S122T *	
									V170I *	
30140067	1b		Baseline	Baseline	02DEC2013	1	NS3/4A	NS3	I18V	
									R26K	
									V48I/V	
									I248V	
									G262G/S	
									V339I	
									S343N	
									G383S	
									T402A	
									I407V	
									M470G	
									I586I/T	
									T610I	
									T611L	
								NS4A	I37V	
									V43I/V	
30140068	1b		Baseline	Baseline	19NOV2013	1	NS3/4A	NS3	R26K	
									V48I/V	
									T54S *	
									S61A	
									I71V	
									T72I/T	
									V132I/V *	
									H246H/Y	
									P264P/S	
									I288M	
									S343N	
									T358V	
									G383A	
									I407V	
									M470G	
									S510T	
									S534G	
									F557L	
									I586I/M	
									T610I/T	
									T611L	
									A621T	
								NS4A	I37I/V	
									R46Q	
30140070	1b		Baseline	Baseline	05DEC2013	1	NS3/4A	NS3	R26K	
									V48I	
									S61T	
									P96A/P	
									S122G/S *	
									I248V	
									T299S	
									S343N	
									T344V	
									T358A	
									L377I	
									L386I	
									T402A	
									I407V	
									M470A	
									S534G	
									Q572K	
									T611L	
									T616C	
								NS4A	I37V	
									R46Q	
30140077	1b		Baseline	Baseline	06DEC2013	1	NS3/4A	NS3	R26K	
									V48I	
									I248V	
									T299S	
									S343N	
									T344I	
									T358A	
									S403G/S	
									I407V	
									M470R	
									F557L	
									T611L	
								NS4A	I29V	
									R46Q	
30140078	1b		Baseline	Baseline	02DEC2013	1	NS3/4A	NS3	I3V	
									S7A	
									R26K	
									V48I	
									H246Y	
									N251S	
									P264S	
									T299S	
									A315V	
									V318A	
									S343N	
									I407V	
									M470G	
									D555E	
									T611L	
								NS4A	I29I/V	
									I37I/V	
									R46Q	
30140081	1b		Baseline	Baseline	03DEC2013	1	NS3/4A	NS3	S7A	
									L14F	
									R26K	
									V48I	
									Y56F	
									I248V	
									V256I	
									I288M	
									T299S	
									V339I	
									S343N	
									T344V	
									S382A	
									T402S	
									I407V	
									F418Y	
									M470R	
									N607T	
									T611L	
								NS4A	R46Q	
30140084	1b		Baseline	Baseline	11DEC2013	1	NS3/4A	NS3	S7A	
									R26K	
									V48I	
									L94M	
									V132I/V *	
									A150A/V	
									H246H/Y	
									I248V	
									A263A/G	
									A340I	
									S342P	
									S343T	
									T358I/T	
									L386I	
									I407V	
									M470G	
									S534G	
									T611L	
								NS4A	R46Q	
30140085	1b		Baseline	Baseline	03DEC2013	1	NS3/4A	NS3	L13I	
									R26K	
									V170I *	
									I248V	
									T299S	
									S343N	
									T358A	
									S382L	
									L386I	
									I407V	
									M470G	
									T611L	
									I615V	
								NS4A	A36V	
									I37V	
30140086	1b		Baseline	Baseline	11DEC2013	1	NS3/4A	NS3	R26K	
									V35I	
									S61P	
									A150V	
									V172I	
									A263G	
									P264P/S	
									I288M	
									S343N/T	
									G383A	
									I407V	
									M470T	
									T611L	
									I615V	
								NS4A	K34R	
30140087	1b		Baseline	Baseline	11DEC2013	1	NS3/4A	NS3	S7A	
									R26K	
									V48I	
									Y56F	
									V132I/V *	
									A150A/V	
									I248V	
									P264S	
									I288I/M	
									T299S	
									S343N	
									G383S	
									L386I	
									T402A/T	
									I407V	
									I472I/T	
									V490I	
									D555E	
									I586T	
									T611L	
								NS4A	I37V	
30140088	1b		Baseline	Baseline	11DEC2013	1	NS3/4A	NS3	L14F	
									R26K/R	
									R117Y	
									A150V	
									G237G/S	
									H246Y	
									I248V	
									N251G	
									P264A	
									S343N	
									G383S	
									I407V	
									R469K/R	
									M470R	
									T477A/T	
									V609I	
									T610I	
									T611L	
								NS4A	K34R	
									I38V	
									V43I	
30140091	1b		Baseline	Baseline	04DEC2013	1	NS3/4A	NS3	S7A	
									R26K	
									S61P	
									K68T	
									G120G/S	
									S122G *	
									A150A/V	
									K213R	
									T299S	
									V329I	
									P334S	
									S343N	
									G383S	
									I407V	
									E447D	
									F475Y	
									S510T	
									S534G	
									A553S	
									D555E	
									I586T	
									V609I	
									T610I/T	
									T611L	
									A621T	
								NS4A	T19S	
									K34R	
30140092	1b		Baseline	Baseline	11DEC2013	1	NS3/4A	NS3	S7A	
									L14I	
									R26K	
									E30E/G	
									Y56F	
									V132I *	
									D168E *	
									I248V	
									P264S	
									Y284D/Y	
									I288M	
									T299S	
									A315V	
									P334H	
									S343N	
									I356V	
									L386I	
									T402A	
									I407V	
									T445S	
									T550A	
									D555E	
									V561I	
									I586T	
									T611L	
									I615V	
								NS4A	R46Q	
30140093	1b		Baseline	Baseline	11DEC2013	1	NS3/4A	NS3	R26K	
									V48I	
									S61A	
									T72I	
									V170I *	
									P264S	
									T295V	
									T299S	
									S343N	
									T344I/T	
									I347V	
									G383A/C/G/S	
									T402S	
									I407V	
									T433I	
									M470R	
									F475Y	
									F557L	
									I586T	
									T610I	
									T611L	
								NS4A	I29I/V	
									R46Q	
30140094	1b		Baseline	Baseline	11DEC2013	1	NS3PART	NS3	L14I/L	
									R26K	
									V48I/V	
									V51A/M/T/V	
									T72I/T	
									V132I/V *	
									A150A/V	
									V170I/V *	
									T178A/I/T/V	
30140098	1b		Baseline	Baseline	05DEC2013	1	NS3/4A	NS3	R26K	
									V183T	
									K213R	
									I248V	
									T299S	
									S343N	
									T344I	
									A379A/T	
									G383S	
									L386I	
									D405N	
									I407V	
									F418Y	
									M470S	
									T611L	
									I615V	
								NS4A	I37V	
									R46Q	
30140099	1b		Baseline	Baseline	04DEC2013	1	NS3/4A	NS3	S7A	
									R26K	
									V48I	
									T72I	
									Q86P	
									T108S	
									V132I *	
									S189A	
									F197Y	
									K244T	
									I248V	
									V318T	
									S343N	
									T344N	
									I356L	
									T358V	
									K371R	
									A379T	
									G383A	
									D405N	
									I407V	
									S459T	
									M470R	
									S510T	
									D555E	
									V561T	
									T611L	
									Y618L	
									V630I	
								NS4A	I29L	
									R46Q	
30140100	1b		Baseline	Baseline	04DEC2013	1	NS3/4A	NS3	S7A	
									R26K	
									K213R	
									I248V	
									T299S	
									P334S	
									S343N	
									T344I	
									I407V	
									M470A	
									V609I	
									T611L	
								NS4A	S22C	
									K34R	
									I38V	
30140103	1b		Baseline	Baseline	05DEC2013	1	NS3/4A	NS3	I18V	
									Y56F	
									I71V	
									V170I *	
									I248V	
									T299S	
									S343N	
									T344V	
									I356L	
									I407V	
									F418Y	
									M470G	
									T611L	
								NS4A	R46Q	
30140106	1b		Baseline	Baseline	09DEC2013	1	NS3/4A	NS3	R26K	
									S61T	
									T72I	
									Y105F	
									S122T *	
									V132I *	
									G237S	
									I248V	
									A263S	
									T299S	
									S343N	
									A379T	
									G383S	
									L386I	
									I407V	
									F418Y	
									V451M	
									M470R	
									S510T	
									S534G	
									T611L	
									A621T	
								NS4A	K34R	
									I38V	
									R46Q	
30140108	1b		Baseline	Baseline	10DEC2013	1	NS3/4A	NS3	R26K	
									V48I	
									S61A	
									S122G *	
									H246Y	
									A263G	
									P264S	
									P334S	
									V339I	
									S343N	
									T358V	
									G383S	
									I407V	
									M470G	
									T540P	
									T611L	
								NS4A	I30V	
									I37V	
									R46Q	
30140110	1b		Baseline	Baseline	26NOV2013	1	NS3/4A	NS3	S7A	
									L14F	
									R26K	
									V48I	
									S61A	
									L94I	
									S122N *	
									V132I *	
									H246Y	
									N251S	
									I265V	
									T299S	
									S343N	
									K371R	
									I407V	
									F418Y	
									M470G	
									I586T	
									T610I	
									T611L	
								NS4A	I37V	
									R46Q/R	
30140113	1b		Baseline	Baseline	04DEC2013	1	NS3/4A	NS3	S7A	
									R26K	
									T46S	
									V48I	
									Y56F/Y	
									T72I	
									V132I *	
									S147L/M	
									G237S	
									I248V	
									T299S	
									T312S	
									S343N	
									S382V	
									L386I	
									I407V	
									F418Y	
									M470R	
									P595A	
									V609I	
									T610I	
									T611L	
								NS4A	I37V	
									I38V	
									R46Q	
30140114	1b		Baseline	Baseline	04DEC2013	1	NS3/4A	NS3	R26K	
									A87S	
									A150V	
									D168D/E *	
									I248V	
									N251S	
									V329I/V	
									S343N	
									K371R	
									G383S	
									I407V	
									F418F/Y	
									V451A/V	
									M470G	
									S534G/S	
									I586I/T	
									T611L	
									T616C	
									V630A	
								NS4A	K34R	
									I37V	
									I38V	
30140116	1b		Baseline	Baseline	10DEC2013	1	NS3/4A	NS3	R26K	
									V48I	
									Y56F	
									Q86P	
									I114V	
									V132I *	
									I248V	
									V256I	
									S343N	
									E357A/E	
									I407V	
									M470R	
									T610I	
									T611L	
								NS4A	I37V	
									R46Q	
30140117	1b		Baseline	Baseline	09DEC2013	1	NS3PART	NS3	S7A	
									R26K	
									V48I	
									P89S	
									L94M	
									V132I *	
									S147L	
									A150V	
30140118	1b		Baseline	Baseline	11DEC2013	1	NS3/4A	NS3	L14I	
									R26K	
									V51A	
									I71V	
									Q80L *	
									S102P/S	
									A150V	
									S189T	
									A240T	
									V256I	
									T295V	
									S297A	
									T299S	
									P334S	
									S343N	
									T344A	
									I356L	
									I407V	
									M470R	
									S510T	
									F557Y	
									I586T	
									V605I	
									T611F	
								NS4A	I37V	
30140120	1b		Baseline	Baseline	11DEC2013	1	NS3/4A	NS3	R26K	
									I71I/V	
									V151A/V	
									V170I *	
									T177A/T	
									M179A/T	
									P334H	
									S343N	
									T344I	
									T358A	
									I407V	
									M470P	
									T611L	
								NS4A	I37T	
									R46Q	
30140124	1b		Baseline	Baseline	18DEC2013	1	NS3/4A	NS3	S7A	
									L13I/T	
									S147M	
									I153V	
									I288M	
									T299S	
									I300I/V	
									S343N	
									T358A	
									S382V	
									I407V	
									M470R	
									T477A	
									V524I/V	
									I586I/T	
									T610V	
									T611L	
									I615M/V	
								NS4A	T2A	
									V26I	
									I37V	
									R46Q	
30140130	1b		Baseline	Baseline	04DEC2013	1	NS3/4A	NS3	R26K	
									T63S	
									T72I	
									H246Y	
									I248V	
									G262G/S	
									T299S	
									S343N	
									I356L	
									S382A/L/S/V	
									G383S	
									L386V	
									I407V	
									F418F/Y	
									M470A	
									I472I/V	
									T477A	
									D555E	
									P574S	
									I586T	
									T611L	
								NS4A	L13M	
									T19S	
									S22C	
									I30V	
									I37T	
									R46Q/R	
30140133	1b		Baseline	Baseline	04DEC2013	1	NS3/4A	NS3	S7A	
									R26K	
									Y56F	
									S147L	
									A150V	
									I248V	
									I288M	
									T299S	
									S343N	
									S382V	
									G383S	
									I407V	
									M470R	
									T477A	
									F557L	
									T611L	
								NS4A	I29V	
									K34R	
									I38V	
									V43A	
									R46Q	
30140140	1b		Baseline	Baseline	04DEC2013	1	NS3PART	NS3	R26K	
									V48I	
									P89T	
30140143	1b		Baseline	Baseline	12DEC2013	1	NS3/4A	NS3	R26K	
									V132I *	
									V170I *	
									H246Y	
									I248T	
									V256I	
									S297A	
									T299S	
									P334S	
									N335S	
									S343N	
									T344V	
									I356L	
									I407V	
									F418Y	
									M470G	
									I586V	
									T611L	
								NS4A	I30V	
									A36V	
									R46Q	
30140146		1b	Baseline	Baseline	10DEC2013	1	NS3/4A	NS3	R26K	
									T72I	
									Q86S	
									R117H	
									V132I *	
									V170I *	
									I248V	
									T299S	
									A315A/V	
									P334S	
									S343N	
									S382T	
									I407V	
									M470R	
									S510T	
									V609T	
									T611L	
									V630I	
								NS4A	K34R	
									R46Q	
30140147		1b	Baseline	Baseline	10DEC2013	1	NS3/4A	NS3	R26K	
									K213R	
									I248V	
									V256A	
									A263T	
									I356V	
									G383S	
									I407V	
									M470A	
									T477A	
									T610I	
									T611L	
								NS4A	I37V	
									V43I	
30140148	1b		Baseline	Baseline	17DEC2013	1	NS3PART	NS3	R26K	
									Q86S	
									V170I *	
30140153	1b		Baseline	Baseline	12DEC2013	1	NS3PART	NS3	S7A	
									R26K	
									R117Q	
									V132I *	
									S147L	
30140154	1b		Baseline	Baseline	13DEC2013	1	NS3/4A	NS3	R26K	
									V48I/V	
									K244R	
									I248V	
									I252V	
									T299S	
									S343N	
									T344I/T	
									I347V	
									I356L	
									T358V	
									G383S	
									I407V	
									F418Y	
									M470A	
									F557L	
									T610I	
									T611L	
30140157	1b		Baseline	Baseline	13DEC2013	1	NS3/4A	NS3	L13V	
									R26K	
									T46S	
									V48I	
									N49S	
									N77S	
									Q86P	
									R92Q	
									V132I *	
									V170I *	
									F184Y	
									F197Y	
									H246Y	
									I248V	
									D249E	
									T299S	
									S343N	
									T344D	
									I356L	
									T358A	
									K371R	
									A379T	
									G383A	
									I407V	
									F418Y	
									T433I	
									M470G	
									I586V	
									T610V	
									T611L	
								NS4A	I30V	
									I37V	
30140158	1b		Baseline	Baseline	27DEC2013	1	NS3/4A	NS3	S7A	
									R26K	
									A39S	
									S61T	
									V170I *	
									I248V	
									T299S	
									V339A	
									S342P	
									S343N	
									K372R	
									G383S	
									I407V	
									M470A	
									T477A	
									T611L	
								NS4A	V26T	
									I29V	
									I37V	
									R46Q	
30140159	1b		Baseline	Baseline	13DEC2013	1	NS3/4A	NS3	S7A	
									L13V	
									P67A/P	
									V132I *	
									A150V	
									I248V	
									D249N	
									P264S	
									T299S	
									S343N	
									T344I/T	
									G383A	
									L386M	
									I407V	
									M470A	
									S510T	
									F557F/L	
									T611L	
									I615V	
								NS4A	I37V	
									I38V	
30140166	1b		Baseline	Baseline	10DEC2013	1	NS3/4A	NS3	R26K	
									V48I	
									R117H	
									V132I *	
									A150V	
									I153V	
									I248V	
									T295V	
									T299S	
									P334S	
									S343N	
									T344S	
									G383A	
									I407V	
									M470R	
									F475Y	
									T611L	
									I615V	
								NS4A	K34R	
									R46Q	
30140167	1b		Baseline	Baseline	18DEC2013	1	NS3/4A	NS3	S7A	
									Y56F	
									T72I	
									S147L	
									I153I/V	
									V170I/V *	
									P264S	
									I265V	
									I288M	
									T299S	
									P334S	
									S343N	
									A379T	
									S382V	
									L386I	
									S403N/S	
									I407V	
									F418Y	
									R469K	
									M470R	
									T477A	
									T611L	
									T612S	
								NS4A	I38V	
									V43I/V	
									R46K	
30140168	1b		Baseline	Baseline	19DEC2013	1	NS3/4A	NS3	S7A	
									L14F	
									R26K	
									C52M	
									Y56F	
									T72I	
									P89S	
									M179I	
									F197Y	
									A220S	
									I288M	
									T299S	
									A315V	
									S343T	
									T344N	
									I356L	
									T358A	
									K360R	
									G361E	
									S382V	
									G383S	
									L386V	
									A390P	
									D405N	
									I407V	
									F418Y	
									V451M	
									M470R	
									T477A	
									T505S	
									D555E	
									Y559F	
									V561T	
									I586V	
									T611L	
								NS4A	I30V	
									I37V	
									R46Q	
30140173	1b		Baseline	Baseline	23DEC2013	1	NS3/4A	NS3	S7A	
									I18V	
									R26K	
									Y56F	
									I248I/V	
									I288M	
									T299S	
									S343N	
									T344I/T	
									T358N	
									G383S	
									I407V	
									F418Y	
									T610I	
									T611L	
								NS4A	T20A	
									I30I/V	
									I37V	
30140178	1b		Baseline	Baseline	16DEC2013	1	NS3PART	NS3	R26K	
									N49S	
									Y56F	
									Q86A	
									P89A	
									A150V	
30140181	1b		Baseline	Baseline	16DEC2013	1	NS3/4A	NS3	R26K	
									I248V	
									P264S	
									S343N	
									I407V	
									F418Y	
									M470G	
									T610A	
									T611L	
								NS4A	I29V	
									I37V	
									V43A	
									R46K	
30140184	1b		Baseline	Baseline	19DEC2013	1	NS3/4A	NS3	I18I/V	
									R26K	
									L94L/M	
									S122G *	
									V132I *	
									S147L/S	
									A150V	
									A220A/T	
									I288I/M	
									T299S	
									S343N	
									I356L	
									T358V	
									I407V	
									F418Y	
									M470G	
									S534G	
									T610I	
									T611L	
									A621T	
								NS4A	I30V	
									I37V	
									R46Q	
30140185	1b		Baseline	Baseline	07JAN2014	1	NS3/4A	NS3	R26K	
									V48I	
									L94M	
									V132I *	
									A150V	
									V170I/V *	
									I248V	
									T299S	
									S343N	
									I356L	
									T358A/T	
									I407V	
									F418Y	
									M470G/R	
									T611L	
									Y618F	
								NS4A	K34R	
									R46Q	

! Indicates an insertion; ^ Indicates a deletion
* Indicates polymorphisms at selected NS3 postions (36, 41, 43, 54, 55, 80, 107, 122, 132, 138, 155, 156, 158, 168, 169, 170, 174, 175)
Polymorphism are defined as changes from con1 (AJ238799) and H77 (AF009606) for hcv geno/subtype 1b and 1a/other, respectively.	
[LVIBL01.rtf] [\STAT\Analyses\Programs\FinalAnalysis\Final1\2.TLF\6.Virology\VIR_FA.sas] 23OCT2015, 17:02	

LVIBL01:	Listing of Baseline Polymorphisms; Intent-to-treat (Study TMC435HPC3014)
Treatment Group = Simeprevir 12Wks 150 mg PR12/24 - HCV Genotype = Genotype 1 - Treatment Duration = >12 Weeks Treatment- HCV Geno/Subtype = 1a/other	
Subject ID	Subtype
(LIPA)	Subtype
(Trugene)	Visit	Time Point	Date	Day in Study	Sequenced Region	Region	Polymorphism	
30140014	1a		Baseline	Baseline	25OCT2013	1	NS3PART	NS3	T40A	
									P67S	
									S91A	
									L153I	
									N174S *	
									T178S	
30140022	1a		Baseline	Baseline	04NOV2013	1	NS3/4A	NS3	T40A	
									P67S	
									S91A	
									L153I	
									N174S *	
									S189T	
									F197Y	
									V248I	
									V318T	
									S332P	
									V358A	
									K372R	
									A379T	
									I386V	
									S410A	
								NS4A	Q46R	
30140041	1a		Baseline	Baseline	21NOV2013	1	NS3/4A	NS3	I18I/V	
									T40A	
									P67A/S	
									S91A	
									T98A/T	
									S122G/S *	
									A147A/V	
									L153I	
									N174S *	
									T185S/T	
									S189S/T	
									S196A/T	
									F197Y	
									V248I	
									V318T	
									S332P	
									V358A	
									I386I/V	
									S410A	
									F418Y	
									Q572Q/R	
									P574L	
									I586I/T	
									I615V	
								NS4A	Q46R	
30140051	1a		Baseline	Baseline	22NOV2013	1	NS3/4A	NS3	T40A	
									R62K/R	
									P67S	
									Q89H	
									S91A	
									T95I	
									S122G *	
									L153I	
									N174G *	
									T185S	
									F197Y	
									V248I	
									D249E	
									S299T	
									V318T	
									S332P	
									P334H	
									L377F	
									I386V	
									S410A	
									A604G	
									V609I	
30140059	1a		Baseline	Baseline	12NOV2013	1	NS3/4A	NS3	T40A	
									S91A	
									L153I	
									N174N/S *	
									V248I	
									S332P	
									V358A	
									S410A	
									F418Y	
									F557L	
									I615I/V	
								NS4A	Q46R	
30140064	1a		Baseline	Baseline	02DEC2013	1	NS3PART	NS3	I18V	
									T40A	
									P67S	
									S91A	
									L153I	
									N174G *	
30140066	1a		Baseline	Baseline	04DEC2013	1	NS3/4A	NS3	T40A	
									Q80K *	
									L153I	
									N174S *	
									T178S	
									A192I	
									G237A/G	
									V306A/V	
									V329I/V	
									S332P	
									I386I/V	
									T402A/T	
									S410A	
									F418F/Y	
									T505M/T	
									I586I/S	
									I615V	
								NS4A	I29V	
									I37I/V	
									Q46R	
30140069	1a		Baseline	Baseline	05DEC2013	1	NS3/4A	NS3	T40A	
									P67P/S	
									Q80K *	
									A87S	
									S91A	
									L153I	
									N174S *	
									A240V	
									V248I	
									S332P	
									S342P	
									V358A	
									S410A	
									A455S	
									F557L	
									N607H/N	
									V609I	
									T621A	
								NS4A	V43A	
30140079	1a		Baseline	Baseline	06DEC2013	1	NS3PART	NS3	T46S/T	
									V55A *	
									I64L	
									S91A	
									I114I/V	
									R119K/R	
									L153I	
									N174S *	
30140090	1a		Baseline	Baseline	04DEC2013	1	NS3/4A	NS3	T40A	
									L143V	
									L153I	
									S196T	
									A240V	
									S332P	
									V358A	
									S410A	
									F418Y	
									I586T	
									I615V	
								NS4A	I29I/V	
									Q46Q/R	
30140115	1a		Baseline	Baseline	05DEC2013	1	NS3PART	NS3	P67S	
									S91A	
									I114I/V	
									L153I	
									N174S *	
30140119	1a		Baseline	Baseline	03DEC2013	1	NS3/4A	NS3	T40A	
									S91A	
									L94M	
									L153I	
									D168E *	
									N174S *	
									T185S	
									A192A/V	
									F197Y	
									V248I	
									P264S	
									V318T	
									V319I	
									S332P	
									I386I/V	
									S410A	
									V490I	
									F557L	
									Q572K/Q	
									I615V	
								NS4A	K34R	
									Q46R	
30140123	1a		Baseline	Baseline	12DEC2013	1	NS3/4A	NS3	T40A	
									Q80K *	
									L153I	
									K224T	
									V248I	
									S332P	
									V358A	
									K360R	
									S410A	
									F418Y	
									A477T	
									N556S	
									I615V	
								NS4A	I29V	
									Q46Q/R	
30140137	1a		Baseline	Baseline	05DEC2013	1	NS3/4A	NS3	T40A	
									P67S	
									S91A	
									L153I	
									N174G/S *	
									T178I/T	
									F197Y	
									V248I	
									V318T	
									S332P	
									V358A	
									V382T	
									I386V	
									S410A	
									F418Y	
									A477A/T	
									F557F/L	
									I615V	
								NS4A	Q46R	
30140149	1a		Baseline	Baseline	10DEC2013	1	NS3/4A	NS3	T40A	
									K68N	
									S122G *	
									L153I	
									S332P	
									I386V	
									S410A	
									P470R	
								NS4A	Q46R	
30140150	1a		Baseline	Baseline	04DEC2013	1	NS3PART	NS3	T40A	
									Q80K *	
									L153I	
30140155	1a		Baseline	Baseline	13DEC2013	1	NS3/4A	NS3	T40A	
									P67S	
									S91A	
									L153I	
									T185S	
									F197Y	
									V248I	
									A315V	
									V318T	
									S332P	
									I347V	
									I386V	
									V399I	
									S410A	
									F418Y	
									I615V	
30140177	1a		Baseline	Baseline	23DEC2013	1	NS3/4A	NS3	V29A	
									T40A	
									N49S	
									P67S	
									Q80K *	
									S91T	
									L153I	
									V248I	
									S299T	
									S332P	
									K371R	
									K372R	
									A379T	
									V382T	
									I386V	
									T402A	
									S410A	
									K469R	
								NS4A	I29V	
									V43M	
									Q46R	

! Indicates an insertion; ^ Indicates a deletion
* Indicates polymorphisms at selected NS3 postions (36, 41, 43, 54, 55, 80, 107, 122, 132, 138, 155, 156, 158, 168, 169, 170, 174, 175)
Polymorphism are defined as changes from con1 (AJ238799) and H77 (AF009606) for hcv geno/subtype 1b and 1a/other, respectively.	
[LVIBL01.rtf] [\STAT\Analyses\Programs\FinalAnalysis\Final1\2.TLF\6.Virology\VIR_FA.sas] 23OCT2015, 17:02	

LVIBL01:	Listing of Baseline Polymorphisms; Intent-to-treat (Study TMC435HPC3014)
Treatment Group = Simeprevir 12Wks 150 mg PR12/24 - HCV Genotype = Genotype 1 - Treatment Duration = >12 Weeks Treatment- HCV Geno/Subtype = 1b	
Subject ID	Subtype
(LIPA)	Subtype
(Trugene)	Visit	Time Point	Date	Day in Study	Sequenced Region	Region	Polymorphism	
30140018	1b		Baseline	Baseline	11NOV2013	1	NS3/4A	NS3	R26K	
									V48I	
									Y56F	
									Q86M	
									R117H	
									A150V	
									V170I *	
									I248V	
									T295I	
									T299S	
									V329I	
									V339I	
									A340G	
									S343N	
									I356L	
									T402A	
									D405N	
									I407V	
									M470R	
									S510T	
									T611L	
									A621T	
									S624A	
								NS4A	I29L	
									I37V	
									R46Q	
30140042		1b	Baseline	Baseline	21NOV2013	1	NS3PART	NS3	S7A	
									R26K	
									V48I/V	
									S61A/S	
									R117H	
									S147L	
30140049	1b		Baseline	Baseline	06NOV2013	1	NS3/4A	NS3	S7A	
									R26K	
									Y56F/Y	
									I71V	
									Q86P	
									T185S	
									I248V	
									T299S	
									P334S	
									S343N	
									I356L	
									I407V	
									S534G	
									D555E	
									T591M/V	
									T611L	
									A621T	
								NS4A	I30N	
									I37V	
									I38V	
									R46Q	
30140071	1b		Baseline	Baseline	19NOV2013	1	NS3/4A	NS3	R26K	
									T46S	
									V48I	
									I71V	
									I248V	
									P264P/S	
									V329I/V	
									S343N	
									T358V	
									L386I	
									I407V	
									F418F/Y	
									M470G	
									S510T	
									S534G	
									V609I	
									T611L	
									A621T	
								NS4A	I37V	
									R46Q	
30140073	1b		Baseline	Baseline	27NOV2013	1	NS3/4A	NS3	S7T	
									R26K	
									I248V	
									V256I	
									T299S	
									I407V	
									T433I	
									M470R	
									S510T	
									F557L	
									T611L	
									I615V	
								NS4A	R46K	
30140082	1b		Baseline	Baseline	11DEC2013	1	NS3/4A	NS3	R26K	
									V48I	
									T63P	
									Q86P	
									T95S	
									V132I *	
									I248V	
									V256I	
									P264S	
									S297A	
									I303V	
									S343N	
									I407V	
									F418Y	
									M470R	
									D555E	
									V561T	
									T611L	
								NS4A	R46Q	
30140083	1b		Baseline	Baseline	11DEC2013	1	NS3PART	NS3	S7A	
									R26K	
									T72I/T	
									R117H/R	
									V132I/V *	
									K136K/R	
									T178I/T	
30140089	1b		Baseline	Baseline	11DEC2013	1	NS3/4A	NS3	L14I/L	
									R26K	
									V48I	
									T72I	
									I248V	
									A263G	
									I288M	
									S343N	
									T344S	
									K371R	
									L386I	
									I407V	
									M470R	
									T611L	
								NS4A	I37V	
									R46Q	
30140095	1b		Baseline	Baseline	06DEC2013	1	NS3/4A	NS3	S7A	
									R26K	
									S61A	
									T72I	
									A91A/T	
									R117C	
									H246Y	
									I248T	
									T299S	
									P334S	
									S343N	
									T344I/T	
									I356L	
									T358A	
									S382A/V	
									I407V	
									M470R	
									D555E	
									F557L	
									T611L	
									I615V	
									M620A	
								NS4A	K34R	
									I37V	
									V43A/T	
									R46Q	
30140096	1b		Baseline	Baseline	28NOV2013	1	NS3/4A	NS3	R26K	
									V48I	
									N49S	
									V170I *	
									I248V	
									P264S	
									T299S	
									S343N	
									T358V	
									G383S	
									L386I	
									I407V	
									F418Y	
									M470G	
									T610I/V	
									T611L	
								NS4A	I30N	
									A36V	
									R46Q	
30140111	1b		Baseline	Baseline	04DEC2013	1	NS3/4A	NS3	S7A	
									R26K	
									V48I	
									Y56F	
									S147L	
									I248V	
									I265V	
									T299S	
									S343N	
									E357D	
									S382V	
									L386I	
									I407V	
									F418Y	
									M470R	
									T477A	
									T610I	
									T611L	
								NS4A	R46Q/R	
									E47A/E	
30140122	1b		Baseline	Baseline	13DEC2013	1	NS3/4A	NS3	S7A	
									I18V	
									R26K	
									V48I	
									Y56F	
									T72I	
									P89P/S	
									T98S	
									S147L/Q	
									V170I/V *	
									I248V	
									T299S	
									S343N	
									I347V	
									T358A	
									S382V	
									I407V	
									F418Y	
									M470R	
									T477A	
									T540N	
									D555E	
									V561T	
									T611L	
								NS4A	K34R	
									I38V	
									R46Q	
30140125	1b		Baseline	Baseline	09DEC2013	1	NS3/4A	NS3	R26K	
									T46A	
									V48I	
									V107I/V *	
									V170I *	
									K213K/R	
									P264P/S	
									I288M	
									I300V	
									V339T	
									S342P/S	
									S343N/S	
									T358L	
									I407V	
									F418Y	
									T433I/T	
									M470G	
									S534G/S	
									A544A/T	
									R570K/R	
									T611L	
								NS4A	K34R	
									I37I/V	
									R46Q	
30140132	1b		Baseline	Baseline	13DEC2013	1	NS3/4A	NS3	S7A	
									L14I	
									R26K	
									V48I	
									Y56F	
									T63A/T	
									T72I	
									R117C/R	
									S147L	
									I248V	
									T295I	
									T299S	
									P334S	
									S343N	
									T344I/M	
									S382V	
									I407V	
									F418Y	
									R469K	
									M470R	
									T477A	
									I586T	
									V605I	
									T611F	
									M620T	
								NS4A	I37V	
									R46Q	
30140134	1b		Baseline	Baseline	19DEC2013	1	NS3/4A	NS3	S7A	
									L13L/V	
									R26K	
									Y56F	
									T95X	
									S147M	
									N251S	
									T299S	
									S343N	
									T344I/T	
									E357E/G	
									T358A	
									S382T	
									I407V	
									I542I/M/V	
									T611L	
								NS4A	I37V	
									V43A/T	
									R46Q	
30140142	1b		Baseline	Baseline	13DEC2013	1	NS3/4A	NS3	L14F/L	
									R26K	
									T72A	
									S122T *	
									G237S	
									H246Y	
									I259V	
									V339I	
									S343N	
									T344I/T	
									G383A	
									L384S	
									L386I	
									I407V	
									M470G	
									S510T	
									S534G	
									T611L	
									A621T	
								NS4A	I30V	
									K34R	
									R46Q	
30140163	1b		Baseline	Baseline	10DEC2013	1	NS3/4A	NS3	S7A	
									R26K	
									V48I	
									S61T	
									T63A	
									L94M	
									V170I *	
									Q198E	
									I248V	
									S297A/S	
									T299S	
									P334S	
									S343N	
									T344I/V	
									K371K/R	
									I407V	
									F418F/Y	
									T611L	
									I615V	
								NS4A	V26M	
									I37V	
									R46Q	
30140165	1b		Baseline	Baseline	10DEC2013	1	NS3/4A	NS3	S7A	
									R26K	
									V48I	
									Y56F	
									V132I *	
									A150V	
									I248I/V	
									P264S	
									T299S	
									S343N	
									T344I	
									I356L	
									E357D	
									L386V	
									I407V	
									F418Y	
									T449S/T	
									T610V	
									T611L	
								NS4A	I37V	
									R46Q	
30140171	1b		Baseline	Baseline	26DEC2013	1	NS3/4A	NS3	S7A	
									R26K	
									V48I	
									Y56F	
									P89S	
									S147L	
									I248V	
									T299S	
									S343N	
									T358A	
									S382V	
									L386I	
									I407V	
									F418Y	
									M470R	
									T477A	
									T610I	
									T611L	
								NS4A	R46Q	
30140175	1b		Baseline	Baseline	26DEC2013	1	NS3/4A	NS3	S7A	
									R26K	
									Y56F/Y	
									Q80H *	
									R117C	
									S147L	
									A150A/V	
									I248V	
									T299S	
									S343N	
									T344V	
									S382V	
									L386I	
									D405N	
									I407V	
									F418Y	
									M470R/S/T	
									T611L	
									I615V	
30140176	1b		Baseline	Baseline	19DEC2013	1	NS3PART	NS3	S7A	
									L14F	
									R26K	
									V48I/V	
									L94M	
									V132I *	
									S147L/P/S	
									A150V	
30140182	1b		Baseline	Baseline	06JAN2014	1	NS3/4A	NS3	R26K	
									V36I *	
									V48I	
									S61T	
									I71V	
									P89S	
									T196S	
									A220S	
									P264S	
									I265V	
									T299S	
									S343N	
									T344I	
									T358A	
									I407V	
									F418Y	
									M470R	
									V561A	
									V609I	
									T611L	
								NS4A	T20A	
									I37V	
									R46Q	

! Indicates an insertion; ^ Indicates a deletion
* Indicates polymorphisms at selected NS3 postions (36, 41, 43, 54, 55, 80, 107, 122, 132, 138, 155, 156, 158, 168, 169, 170, 174, 175)
Polymorphism are defined as changes from con1 (AJ238799) and H77 (AF009606) for hcv geno/subtype 1b and 1a/other, respectively.	
[LVIBL01.rtf] [\STAT\Analyses\Programs\FinalAnalysis\Final1\2.TLF\6.Virology\VIR_FA.sas] 23OCT2015, 17:02	
