# Supplementary material for: An Open-Label Trial of 12-Week Simeprevir plus Peginterferon/Ribavirin (PR) in Treatment-Naïve Patients with Hepatitis C Virus (HCV) Genotype 1 (GT1)
Source: PLoS One. 2016 Jul 18;11(7):e0158526. doi: 10.1371/journal.pone.0158526 (PMC4948848; doi:10.1371/journal.pone.0158526)
Supplement: S1 Dataset — (ZIP) [file pone.0158526.s009.zip › TEFSUB01.rtf]

TEFSUB01:	Sustained Virologic Response 12 Weeks After the Planned End of Treatment - Subgroup Analyses; Intent-to-treat (Study TMC435HPC3014)
1) HCV Geno/subtype 
Treatment Group = Simeprevir 12Wks 150 mg PR12/24	
	Genotype 1	
	12 Weeks 
Treatment	>12 Weeks 
Treatment	All Subjects	
Analysis set: intent-to-treata				
	123	40	163	
	
Sustained Virologic Response 12 Weeks after EOT				
1a/other				
n/N (%)	31/ 49 
( 63.3%)	10/ 18 
( 55.6%)	41/ 67 
( 61.2%)	
95% CI	(49.77; 76.76)	(32.60; 78.51)	(49.53; 72.86)	
1b				
n/N (%)	50/ 74 
( 67.6%)	11/ 22 
( 50.0%)	61/ 96 
( 63.5%)	
95% CI	(56.90; 78.23)	(29.11; 70.89)	(53.91; 73.17)	
4a				
n/N (%)	-	-	-	
95% CI	-	-	-	
4d				
n/N (%)	-	-	-	
95% CI	-	-	-	
4other				
n/N (%)	-	-	-	
95% CI	-	-	-	
	


a Number of ITT subjects that reached 12 weeks after planned EOT	
[TEFSUB01.rtf] [\STAT\Analyses\Programs\FinalAnalysis\Final1\2.TLF\2.Efficacy\EFF_FA.sas] 23OCT2015, 18:04	

TEFSUB01:	Sustained Virologic Response 12 Weeks After the Planned End of Treatment - Subgroup Analyses; Intent-to-treat (Study TMC435HPC3014)
2) IL28B Genotype 
Treatment Group = Simeprevir 12Wks 150 mg PR12/24	
	Genotype 1	
	12 Weeks 
Treatment	>12 Weeks 
Treatment	All Subjects	
Analysis set: intent-to-treata				
	123	40	163	
	
Sustained Virologic Response 12 Weeks after EOT				
CC				
n/N (%)	30/ 32 
( 93.8%)	8/  8 
( 100.0%)	38/ 40 
( 95.0%)	
95% CI	(85.36; 100.00)	(100.00; 100.00)	(88.25; 100.00)	
CT				
n/N (%)	40/ 73 
( 54.8%)	9/ 20 
( 45.0%)	49/ 93 
( 52.7%)	
95% CI	(43.38; 66.21)	(23.20; 66.80)	(42.54; 62.84)	
TT				
n/N (%)	11/ 18 
( 61.1%)	4/ 12 
( 33.3%)	15/ 30 
( 50.0%)	
95% CI	(38.59; 83.63)	(6.66; 60.01)	(32.11; 67.89)	
	


a Number of ITT subjects that reached 12 weeks after planned EOT	
[TEFSUB01.rtf] [\STAT\Analyses\Programs\FinalAnalysis\Final1\2.TLF\2.Efficacy\EFF_FA.sas] 23OCT2015, 18:04	

TEFSUB01:	Sustained Virologic Response 12 Weeks After the Planned End of Treatment - Subgroup Analyses; Intent-to-treat (Study TMC435HPC3014)
3) Sex 
Treatment Group = Simeprevir 12Wks 150 mg PR12/24	
	Genotype 1	
	12 Weeks 
Treatment	>12 Weeks 
Treatment	All Subjects	
Analysis set: intent-to-treata				
	123	40	163	
	
Sustained Virologic Response 12 Weeks after EOT				
Female				
n/N (%)	38/ 58 
( 65.5%)	8/ 12 
( 66.7%)	46/ 70 
( 65.7%)	
95% CI	(53.28; 77.75)	(39.99; 93.34)	(54.59; 76.83)	
Male				
n/N (%)	43/ 65 
( 66.2%)	13/ 28 
( 46.4%)	56/ 93 
( 60.2%)	
95% CI	(54.65; 77.66)	(27.96; 64.90)	(50.27; 70.16)	
	


a Number of ITT subjects that reached 12 weeks after planned EOT	
[TEFSUB01.rtf] [\STAT\Analyses\Programs\FinalAnalysis\Final1\2.TLF\2.Efficacy\EFF_FA.sas] 23OCT2015, 18:04	

TEFSUB01:	Sustained Virologic Response 12 Weeks After the Planned End of Treatment - Subgroup Analyses; Intent-to-treat (Study TMC435HPC3014)
4) Race 
Treatment Group = Simeprevir 12Wks 150 mg PR12/24	
	Genotype 1	
	12 Weeks 
Treatment	>12 Weeks 
Treatment	All Subjects	
Analysis set: intent-to-treata				
	123	40	163	
	
Sustained Virologic Response 12 Weeks after EOT				
Caucasian				
n/N (%)	62/ 98 
( 63.3%)	15/ 32 
( 46.9%)	77/130 
( 59.2%)	
95% CI	(53.72; 72.81)	(29.59; 64.16)	(50.78; 67.68)	
Black				
n/N (%)	4/  5 
( 80.0%)	0/  1 
(  0.0%)	4/  6 
( 66.7%)	
95% CI	(44.94; 100.00)	-	(28.95; 100.00)	
Asian				
n/N (%)	3/  3 
( 100.0%)	-	3/  3 
( 100.0%)	
95% CI	(100.00; 100.00)	-	(100.00; 100.00)	
Other				
n/N (%)	0/  1 
(  0.0%)	-	0/  1 
(  0.0%)	
95% CI	-	-	-	
Unknown				
n/N (%)	12/ 16 
( 75.0%)	6/  7 
( 85.7%)	18/ 23 
( 78.3%)	
95% CI	(53.78; 96.22)	(59.79; 100.00)	(61.40; 95.12)	
	


a Number of ITT subjects that reached 12 weeks after planned EOT	
[TEFSUB01.rtf] [\STAT\Analyses\Programs\FinalAnalysis\Final1\2.TLF\2.Efficacy\EFF_FA.sas] 23OCT2015, 18:04	

TEFSUB01:	Sustained Virologic Response 12 Weeks After the Planned End of Treatment - Subgroup Analyses; Intent-to-treat (Study TMC435HPC3014)
6) Country 
Treatment Group = Simeprevir 12Wks 150 mg PR12/24	
	Genotype 1	
	12 Weeks 
Treatment	>12 Weeks 
Treatment	All Subjects	
Analysis set: intent-to-treata				
	123	40	163	
	
Sustained Virologic Response 12 Weeks after EOT				
Austria				
n/N (%)	12/ 17 
( 70.6%)	3/  5 
( 60.0%)	15/ 22 
( 68.2%)	
95% CI	(48.93; 92.25)	(17.06; 100.00)	(48.72; 87.64)	
Belgium				
n/N (%)	7/ 14 
( 50.0%)	1/  2 
( 50.0%)	8/ 16 
( 50.0%)	
95% CI	(23.81; 76.19)	(0.00; 100.00)	(25.50; 74.50)	
France				
n/N (%)	21/ 29 
( 72.4%)	6/  8 
( 75.0%)	27/ 37 
( 73.0%)	
95% CI	(56.15; 88.68)	(44.99; 100.00)	(58.66; 87.28)	
Germany				
n/N (%)	14/ 23 
( 60.9%)	3/  5 
( 60.0%)	17/ 28 
( 60.7%)	
95% CI	(40.92; 80.81)	(17.06; 100.00)	(42.62; 78.80)	
Italy				
n/N (%)	9/ 13 
( 69.2%)	3/  6 
( 50.0%)	12/ 19 
( 63.2%)	
95% CI	(44.14; 94.32)	(9.99; 90.01)	(41.47; 84.85)	
Saudi Arabia				
n/N (%)	-	-	-	
95% CI	-	-	-	
Spain				
n/N (%)	12/ 18 
( 66.7%)	4/ 12 
( 33.3%)	16/ 30 
( 53.3%)	
95% CI	(44.89; 88.44)	(6.66; 60.01)	(35.48; 71.19)	
United Kingdom				
n/N (%)	6/  9 
( 66.7%)	1/  2 
( 50.0%)	7/ 11 
( 63.6%)	
95% CI	(35.87; 97.46)	(0.00; 100.00)	(35.21; 92.06)	
	


a Number of ITT subjects that reached 12 weeks after planned EOT	
[TEFSUB01.rtf] [\STAT\Analyses\Programs\FinalAnalysis\Final1\2.TLF\2.Efficacy\EFF_FA.sas] 23OCT2015, 18:04	

TEFSUB01:	Sustained Virologic Response 12 Weeks After the Planned End of Treatment - Subgroup Analyses; Intent-to-treat (Study TMC435HPC3014)
7) Baseline BMI 
Treatment Group = Simeprevir 12Wks 150 mg PR12/24	
	Genotype 1	
	12 Weeks 
Treatment	>12 Weeks 
Treatment	All Subjects	
Analysis set: intent-to-treata				
	123	40	163	
	
Sustained Virologic Response 12 Weeks after EOT				
<25 kg/m2				
n/N (%)	42/ 60 
( 70.0%)	14/ 19 
( 73.7%)	56/ 79 
( 70.9%)	
95% CI	(58.40; 81.60)	(53.88; 93.48)	(60.87; 80.90)	
>=25 - <30 kg/m2				
n/N (%)	31/ 49 
( 63.3%)	6/ 16 
( 37.5%)	37/ 65 
( 56.9%)	
95% CI	(49.77; 76.76)	(13.78; 61.22)	(44.88; 68.96)	
>=30 kg/m2				
n/N (%)	8/ 14 
( 57.1%)	1/  5 
( 20.0%)	9/ 19 
( 47.4%)	
95% CI	(31.22; 83.07)	(0.00; 55.06)	(24.92; 69.82)	
	


a Number of ITT subjects that reached 12 weeks after planned EOT	
[TEFSUB01.rtf] [\STAT\Analyses\Programs\FinalAnalysis\Final1\2.TLF\2.Efficacy\EFF_FA.sas] 23OCT2015, 18:04	

TEFSUB01:	Sustained Virologic Response 12 Weeks After the Planned End of Treatment - Subgroup Analyses; Intent-to-treat (Study TMC435HPC3014)
8) Baseline HCV RNA 
Treatment Group = Simeprevir 12Wks 150 mg PR12/24	
	Genotype 1	
	12 Weeks 
Treatment	>12 Weeks 
Treatment	All Subjects	
Analysis set: intent-to-treata				
	123	40	163	
	
Sustained Virologic Response 12 Weeks after EOT				
<=800000 IU/mL				
n/N (%)	27/ 33 
( 81.8%)	2/  3 
( 66.7%)	29/ 36 
( 80.6%)	
95% CI	(68.66; 94.98)	(13.32; 100.00)	(67.63; 93.48)	
>800000 IU/mL				
n/N (%)	54/ 90 
( 60.0%)	19/ 37 
( 51.4%)	73/127 
( 57.5%)	
95% CI	(49.88; 70.12)	(35.25; 67.46)	(48.88; 66.08)	
	


a Number of ITT subjects that reached 12 weeks after planned EOT	
[TEFSUB01.rtf] [\STAT\Analyses\Programs\FinalAnalysis\Final1\2.TLF\2.Efficacy\EFF_FA.sas] 23OCT2015, 18:04	

TEFSUB01:	Sustained Virologic Response 12 Weeks After the Planned End of Treatment - Subgroup Analyses; Intent-to-treat (Study TMC435HPC3014)
9) Metavir Score 
Treatment Group = Simeprevir 12Wks 150 mg PR12/24	
	Genotype 1	
	12 Weeks 
Treatment	>12 Weeks 
Treatment	All Subjects	
Analysis set: intent-to-treata				
	123	40	163	
	
Sustained Virologic Response 12 Weeks after EOT				
Score F0-F1				
n/N (%)	69/ 93 
( 74.2%)	13/ 25 
( 52.0%)	82/118 
( 69.5%)	
95% CI	(65.30; 83.09)	(32.42; 71.58)	(61.18; 77.80)	
Score F2				
n/N (%)	11/ 29 
( 37.9%)	8/ 15 
( 53.3%)	19/ 44 
( 43.2%)	
95% CI	(20.27; 55.59)	(28.09; 78.58)	(28.55; 57.82)	
Score F3				
n/N (%)	-	-	-	
Missing				
n/N (%)	1/  1 
( 100.0%)	-	1/  1 
( 100.0%)	
	


a Number of ITT subjects that reached 12 weeks after planned EOT	
[TEFSUB01.rtf] [\STAT\Analyses\Programs\FinalAnalysis\Final1\2.TLF\2.Efficacy\EFF_FA.sas] 23OCT2015, 18:04	

TEFSUB01:	Sustained Virologic Response 12 Weeks After the Planned End of Treatment - Subgroup Analyses; Intent-to-treat (Study TMC435HPC3014)
10) Baseline Q80K Mutation 
Treatment Group = Simeprevir 12Wks 150 mg PR12/24	
	Genotype 1	
	12 Weeks 
Treatment	>12 Weeks 
Treatment	All Subjects	
Analysis set: intent-to-treata				
	123	40	163	
	
Sustained Virologic Response 12 Weeks after EOT				
Q80K				
n/N (%)	6/  6 
( 100.0%)	2/  5 
( 40.0%)	8/ 11 
( 72.7%)	
95% CI	(100.00; 100.00)	(0.00; 82.94)	(46.41; 99.05)	
No Q80K				
n/N (%)	71/113 
( 62.8%)	19/ 35 
( 54.3%)	90/148 
( 60.8%)	
95% CI	(53.92; 71.74)	(37.78; 70.79)	(52.95; 68.68)	
Missing				
n/N (%)	4/  4 
( 100.0%)	-	4/  4 
( 100.0%)	
95% CI	(100.00; 100.00)	-	(100.00; 100.00)	
	


a Number of ITT subjects that reached 12 weeks after planned EOT	
[TEFSUB01.rtf] [\STAT\Analyses\Programs\FinalAnalysis\Final1\2.TLF\2.Efficacy\EFF_FA.sas] 23OCT2015, 18:04	

TEFSUB01:	Sustained Virologic Response 12 Weeks After the Planned End of Treatment - Subgroup Analyses; Intent-to-treat (Study TMC435HPC3014)
11) Mode of HCV Infection 
Treatment Group = Simeprevir 12Wks 150 mg PR12/24	
	Genotype 1	
	12 Weeks 
Treatment	>12 Weeks 
Treatment	All Subjects	
Analysis set: intent-to-treata				
	123	40	163	
	
Sustained Virologic Response 12 Weeks after EOT				
Blood Transfusion				
n/N (%)	9/ 21 
( 42.9%)	7/  9 
( 77.8%)	16/ 30 
( 53.3%)	
95% CI	(21.69; 64.02)	(50.62; 100.00)	(35.48; 71.19)	
Hemophilia-Associated Injections				
n/N (%)	1/  1 
( 100.0%)	-	1/  1 
( 100.0%)	
Heterosexual Contact				
n/N (%)	2/  4 
( 50.0%)	1/  1 
( 100.0%)	3/  5 
( 60.0%)	
95% CI	(1.00; 99.00)	-	(17.06; 100.00)	
Intravenously Injectable Drug Use				
n/N (%)	21/ 29 
( 72.4%)	3/  6 
( 50.0%)	24/ 35 
( 68.6%)	
95% CI	(56.15; 88.68)	(9.99; 90.01)	(53.19; 83.95)	
Mother To Child Transmission				
n/N (%)	1/  2 
( 50.0%)	1/  1 
( 100.0%)	2/  3 
( 66.7%)	
95% CI	(0.00; 100.00)	-	(13.32; 100.00)	
Multiple				
n/N (%)	2/  4 
( 50.0%)	-	2/  4 
( 50.0%)	
95% CI	(1.00; 99.00)	-	(1.00; 99.00)	
Other				
n/N (%)	45/ 62 
( 72.6%)	9/ 23 
( 39.1%)	54/ 85 
( 63.5%)	
95% CI	(61.48; 83.68)	(19.19; 59.08)	(53.30; 73.76)	
	


a Number of ITT subjects that reached 12 weeks after planned EOT	
[TEFSUB01.rtf] [\STAT\Analyses\Programs\FinalAnalysis\Final1\2.TLF\2.Efficacy\EFF_FA.sas] 23OCT2015, 18:04	
