# Supplementary material for: An Open-Label Trial of 12-Week Simeprevir plus Peginterferon/Ribavirin (PR) in Treatment-Naïve Patients with Hepatitis C Virus (HCV) Genotype 1 (GT1)
Source: PLoS One. 2016 Jul 18;11(7):e0158526. doi: 10.1371/journal.pone.0158526 (PMC4948848; doi:10.1371/journal.pone.0158526)
Supplement: S1 Dataset — (ZIP) [file pone.0158526.s009.zip › TEFSUB01A.RTF]

TEFSUB01A:	Sustained Virologic Response 12 Weeks After the Planned End of Treatment (SVR12) - by Stratification Factors; Intent-to-treat (Study TMC435HPC3014)
1) HCV Geno/subtype 
Treatment Group = Simeprevir 12Wks 150 mg PR12/24	
	Genotype 1	
	12 Weeks 
Treatment	>12 Weeks 
Treatment	All Subjects	
Analysis set: intent-to-treata				
	123	40	163	
	
Sustained Virologic Response 12 Weeks after EOT				
1a/other				
n/N (%)	31/ 49 
( 63.3%)	10/ 18 
( 55.6%)	41/ 67 
( 61.2%)	
95% CI	(49.77; 76.76)	(32.60; 78.51)	(49.53; 72.86)	
1b				
n/N (%)	50/ 74 
( 67.6%)	11/ 22 
( 50.0%)	61/ 96 
( 63.5%)	
95% CI	(56.90; 78.23)	(29.11; 70.89)	(53.91; 73.17)	
4a				
n/N (%)	-	-	-	
95% CI	-	-	-	
4d				
n/N (%)	-	-	-	
95% CI	-	-	-	
4other				
n/N (%)	-	-	-	
95% CI	-	-	-	
	


a Number of ITT subjects that reached 12 weeks after planned EOT	
[TEFSUB01A.rtf] [\STAT\Analyses\Programs\FinalAnalysis\Final1\2.TLF\2.Efficacy\EFF_FA.sas] 23OCT2015, 18:04	

TEFSUB01A:	Sustained Virologic Response 12 Weeks After the Planned End of Treatment (SVR12) - by Stratification Factors; Intent-to-treat (Study TMC435HPC3014)
2) IL28B Genotype 
Treatment Group = Simeprevir 12Wks 150 mg PR12/24	
	Genotype 1	
	12 Weeks 
Treatment	>12 Weeks 
Treatment	All Subjects	
Analysis set: intent-to-treata				
	123	40	163	
	
Sustained Virologic Response 12 Weeks after EOT				
CC				
n/N (%)	30/ 32 
( 93.8%)	8/  8 
( 100.0%)	38/ 40 
( 95.0%)	
95% CI	(85.36; 100.00)	(100.00; 100.00)	(88.25; 100.00)	
CT				
n/N (%)	40/ 73 
( 54.8%)	9/ 20 
( 45.0%)	49/ 93 
( 52.7%)	
95% CI	(43.38; 66.21)	(23.20; 66.80)	(42.54; 62.84)	
TT				
n/N (%)	11/ 18 
( 61.1%)	4/ 12 
( 33.3%)	15/ 30 
( 50.0%)	
95% CI	(38.59; 83.63)	(6.66; 60.01)	(32.11; 67.89)	
	


a Number of ITT subjects that reached 12 weeks after planned EOT	
[TEFSUB01A.rtf] [\STAT\Analyses\Programs\FinalAnalysis\Final1\2.TLF\2.Efficacy\EFF_FA.sas] 23OCT2015, 18:04	
