# Supplementary material for: An Open-Label Trial of 12-Week Simeprevir plus Peginterferon/Ribavirin (PR) in Treatment-Naïve Patients with Hepatitis C Virus (HCV) Genotype 1 (GT1)
Source: PLoS One. 2016 Jul 18;11(7):e0158526. doi: 10.1371/journal.pone.0158526 (PMC4948848; doi:10.1371/journal.pone.0158526)
Supplement: S1 Dataset — (ZIP) [file pone.0158526.s009.zip › TEFSUB02 - Merged Detectable.rtf]

TEFSUB02COLLAPSED:	Sustained Virologic Response 12 Weeks After the Planned End of Treatment - Subgroup Analyses for Virologic Response at Week 2; Intent-to-treat (Study TMC435HPC3014)	
	Simeprevir
12 Wks
150 mg
PR 12/24 	
	 Genotype 1 	
	 12 Wks 	 >12 Wks 	 All subjects 	
Virologic Response at Week 2				
<25 undetectable				
n/N (%)	39/51 (76.5 %)	1/2 (50.0 %)	40/53 (75.5 %)	
95% CI	(64.83;88.11)	(0.00;100.00)	(63.89;87.06)	
missing				
n/N (%)	1/1 (100.0 %)	0/1	1/2 (50.0 %)	
95% CI	(0.00;100.00)	(0.00;0.00)	(0.00;100.00)	
not <25 undetectable				
n/N (%)	41/71 (57.7 %)	20/37 (54.1 %)	61/108 (56.5 %)	
95% CI	(46.26;69.24)	(38.00;70.11)	(47.13;65.83)	
	
[TEFSUB02COLLAPSED.RTF] [TMC435\HPC3014\DBR_FINAL_ANALYSIS\RE_FINAL_ANALYSIS\PDEV\TEFSUB02COLLAPSED.SAS] 25MAY2016, 18:35	
