# Supplementary material for: An Open-Label Trial of 12-Week Simeprevir plus Peginterferon/Ribavirin (PR) in Treatment-Naïve Patients with Hepatitis C Virus (HCV) Genotype 1 (GT1)
Source: PLoS One. 2016 Jul 18;11(7):e0158526. doi: 10.1371/journal.pone.0158526 (PMC4948848; doi:10.1371/journal.pone.0158526)
Supplement: S1 Dataset — (ZIP) [file pone.0158526.s009.zip › TEFSUB02.rtf]

TEFSUB02:	Sustained Virologic Response 12 Weeks After the Planned End of Treatment - Subgroup Analyses for Early Response Parameters; Intent-to-treat (Study TMC435HPC3014)
1) Virologic Response at Week 2 
Treatment Group = Simeprevir 12Wks 150 mg PR12/24	
	Genotype 1		
	12 Weeks 
Treatment	>12 Weeks 
Treatment	All Subjects		
Analysis set: intent-to-treata					
	123	40	163		
	
Sustained Virologic Response 12 Weeks after EOT					
< 25 undetectable					
n/N (%)	39/ 51 
( 76.5%)	1/  2 
( 50.0%)	40/ 53 
( 75.5%)		
95% CI	(64.83; 88.11)	(0.00; 100.00)	(63.89; 87.06)		
< 25 detectable					
n/N (%)	41/ 71 
( 57.7%)	4/  7 
( 57.1%)	45/ 78 
( 57.7%)		
95% CI	(46.26; 69.24)	(20.48; 93.80)	(46.73; 68.66)		
>= 25 IU/mL					
n/N (%)	-	16/ 30 
( 53.3%)	16/ 30 
( 53.3%)		
95% CI	-	(35.48; 71.19)	(35.48; 71.19)		
Missing					
n/N (%)	1/  1 
( 100.0%)	0/  1 
(  0.0%)	1/  2 
( 50.0%)		
95% CI	-	-	(0.00; 100.00)		
	


a Number of ITT subjects that reached 12 weeks after planned EOT	
[TEFSUB02.rtf] [\STAT\Analyses\Programs\FinalAnalysis\Final1\2.TLF\2.Efficacy\EFF_FA.sas] 23OCT2015, 18:04	

TEFSUB02:	Sustained Virologic Response 12 Weeks After the Planned End of Treatment - Subgroup Analyses for Early Response Parameters; Intent-to-treat (Study TMC435HPC3014)
2) Virologic Response at Week 4 
Treatment Group = Simeprevir 12Wks 150 mg PR12/24	
	Genotype 1		
	12 Weeks 
Treatment	>12 Weeks 
Treatment	All Subjects		
Analysis set: intent-to-treata					
	123	40	163		
	
Sustained Virologic Response 12 Weeks after EOT					
< 25 undetectable					
n/N (%)	81/123 
( 65.9%)	3/  5 
( 60.0%)	84/128 
( 65.6%)		
95% CI	(57.47; 74.23)	(17.06; 100.00)	(57.40; 73.85)		
< 25 detectable					
n/N (%)	-	18/ 26 
( 69.2%)	18/ 26 
( 69.2%)		
95% CI	-	(51.49; 86.97)	(51.49; 86.97)		
>= 25 IU/mL					
n/N (%)	-	0/  8 
(  0.0%)	0/  8 
(  0.0%)		
Missing					
n/N (%)	-	0/  1 
(  0.0%)	0/  1 
(  0.0%)		
	


a Number of ITT subjects that reached 12 weeks after planned EOT	
[TEFSUB02.rtf] [\STAT\Analyses\Programs\FinalAnalysis\Final1\2.TLF\2.Efficacy\EFF_FA.sas] 23OCT2015, 18:04	

TEFSUB02:	Sustained Virologic Response 12 Weeks After the Planned End of Treatment - Subgroup Analyses for Early Response Parameters; Intent-to-treat (Study TMC435HPC3014)
3) Hgb Reduction from Baseline at Week 12 
Treatment Group = Simeprevir 12Wks 150 mg PR12/24	
	Genotype 1		
	12 Weeks 
Treatment	>12 Weeks 
Treatment	All Subjects		
Analysis set: intent-to-treata					
	123	40	163		
	
Sustained Virologic Response 12 Weeks after EOT					
<10 g/L					
n/N (%)	6/ 10 
( 60.0%)	2/  4 
( 50.0%)	8/ 14 
( 57.1%)		
95% CI	(29.64; 90.36)	(1.00; 99.00)	(31.22; 83.07)		
10-19 g/L					
n/N (%)	17/ 24 
( 70.8%)	5/ 10 
( 50.0%)	22/ 34 
( 64.7%)		
95% CI	(52.65; 89.02)	(19.01; 80.99)	(48.64; 80.77)		
20-29 g/L					
n/N (%)	26/ 41 
( 63.4%)	4/  5 
( 80.0%)	30/ 46 
( 65.2%)		
95% CI	(48.67; 78.16)	(44.94; 100.00)	(51.45; 78.98)		
>=30 g/L					
n/N (%)	27/ 40 
( 67.5%)	9/ 13 
( 69.2%)	36/ 53 
( 67.9%)		
95% CI	(52.99; 82.01)	(44.14; 94.32)	(55.36; 80.49)		
Missing					
n/N (%)	5/  8 
( 62.5%)	1/  8 
( 12.5%)	6/ 16 
( 37.5%)		
95% CI	(28.95; 96.05)	(0.00; 35.42)	(13.78; 61.22)		
	


a Number of ITT subjects that reached 12 weeks after planned EOT	
[TEFSUB02.rtf] [\STAT\Analyses\Programs\FinalAnalysis\Final1\2.TLF\2.Efficacy\EFF_FA.sas] 23OCT2015, 18:04	
