# Supplementary material for: An Open-Label Trial of 12-Week Simeprevir plus Peginterferon/Ribavirin (PR) in Treatment-Naïve Patients with Hepatitis C Virus (HCV) Genotype 1 (GT1)
Source: PLoS One. 2016 Jul 18;11(7):e0158526. doi: 10.1371/journal.pone.0158526 (PMC4948848; doi:10.1371/journal.pone.0158526)
Supplement: S1 Dataset — (ZIP) [file pone.0158526.s009.zip › TEFSVR02aB.RTF]

TEFSVR02aB:	Sustained Virologic Response 4 and 24 Weeks After the Planned End of Treatment (SVR4 and SVR24); Intent-to-treat (Study TMC435HPC3014)
Treatment Group = Simeprevir 12Wks 150 mg PR12/24	
	Genotype 1	
	12 Weeks 
Treatment	>12 Weeks 
Treatment	All Subjects	
Analysis set: intent-to-treat				
	123	40	163	
	
SVR4				
Yes				
n/N (%)	109/123 
( 88.6%)	24/ 40 
( 60.0%)	133/163 
( 81.6%)	
95% CI	(83.01; 94.23)	(44.82; 75.18)	(75.65; 87.54)	
	
SVR24				
Yes				
n/N (%)	79/123 
( 64.2%)	21/ 40 
( 52.5%)	100/163 
( 61.3%)	
95% CI	(55.76; 72.70)	(37.02; 67.98)	(53.87; 68.83)	
	
	
[TEFSVR02aB.rtf] [\STAT\Analyses\Programs\FinalAnalysis\Final1\2.TLF\2.Efficacy\EFF_FA.sas] 23OCT2015, 18:04	
