# Supplementary material for: An Open-Label Trial of 12-Week Simeprevir plus Peginterferon/Ribavirin (PR) in Treatment-Naïve Patients with Hepatitis C Virus (HCV) Genotype 1 (GT1)
Source: PLoS One. 2016 Jul 18;11(7):e0158526. doi: 10.1371/journal.pone.0158526 (PMC4948848; doi:10.1371/journal.pone.0158526)
Supplement: S1 Dataset — (ZIP) [file pone.0158526.s009.zip › TEFSVR03.rtf]

TEFSVR03:	Sustained Virologic Response, 4, 12 and 24 Weeks After the Planned End of Treatment - Details; Intent-to-treat (Study TMC435HPC3014)
Treatment Group = Simeprevir 12Wks 150 mg PR12/24	
	Genotype 1	
	12 Weeks 
Treatment	>12 Weeks 
Treatment	All Subjects	
Analysis set: intent-to-treat	123	40	163	
	
SVR4				
Total with SVR4	109/123 
( 88.6%)	24/ 40 
( 60.0%)	133/163 
( 81.6%)	
Total without SVR4 for any reason	14/123 
( 11.4%)	16/ 40 
( 40.0%)	30/163 
( 18.4%)	
Detectable at EOT	0/123 
(  0.0%)	10/ 40 
( 25.0%)	10/163 
(  6.1%)	
Discontinued at least one study therapy (any other case)	0/123 
(  0.0%)	10/ 40 
( 25.0%)	10/163 
(  6.1%)	
Adverse event	0/123 
(  0.0%)	2/ 40 
(  5.0%)	2/163 
(  1.2%)	
Subject reached a virologic endpoint	0/123 
(  0.0%)	8/ 40 
( 20.0%)	8/163 
(  4.9%)	
Undetectable at EOTa	14/123 
( 11.4%)	6/ 40 
( 15.0%)	20/163 
( 12.3%)	
Completed all study therapy	14/123 
( 11.4%)	3/ 40 
(  7.5%)	17/163 
( 10.4%)	
Discontinued at least one study therapy (any other case)	0/123 
(  0.0%)	3/ 40 
(  7.5%)	3/163 
(  1.8%)	
Adverse event	0/123 
(  0.0%)	2/ 40 
(  5.0%)	2/163 
(  1.2%)	
Other	0/123 
(  0.0%)	0/ 40 
(  0.0%)	0/163 
(  0.0%)	
Subject reached a virologic endpoint	0/123 
(  0.0%)	1/ 40 
(  2.5%)	1/163 
(  0.6%)	
HCV RNA >= 25 at Timepoint of SVR	13/123 
( 10.6%)	6/ 40 
( 15.0%)	19/163 
( 11.7%)	
Completed all study therapy	13/123 
( 10.6%)	3/ 40 
(  7.5%)	16/163 
(  9.8%)	
Discontinued at least one study therapy (any other case)	0/123 
(  0.0%)	3/ 40 
(  7.5%)	3/163 
(  1.8%)	
Adverse event	0/123 
(  0.0%)	2/ 40 
(  5.0%)	2/163 
(  1.2%)	
Other	0/123 
(  0.0%)	0/ 40 
(  0.0%)	0/163 
(  0.0%)	
Subject reached a virologic endpoint	0/123 
(  0.0%)	1/ 40 
(  2.5%)	1/163 
(  0.6%)	
Missing at Timepoint of SVR	1/123 
(  0.8%)	0/ 40 
(  0.0%)	1/163 
(  0.6%)	
Completed all study therapy	1/123 
(  0.8%)	0/ 40 
(  0.0%)	1/163 
(  0.6%)	
	
SVR12				
Total with SVR12	81/123 
( 65.9%)	21/ 40 
( 52.5%)	102/163 
( 62.6%)	
Total without SVR12 for any reason	42/123 
( 34.1%)	19/ 40 
( 47.5%)	61/163 
( 37.4%)	
Detectable at EOT	0/123 
(  0.0%)	10/ 40 
( 25.0%)	10/163 
(  6.1%)	
Discontinued at least one study therapy (any other case)	0/123 
(  0.0%)	10/ 40 
( 25.0%)	10/163 
(  6.1%)	
Adverse event	0/123 
(  0.0%)	2/ 40 
(  5.0%)	2/163 
(  1.2%)	
Subject reached a virologic endpoint	0/123 
(  0.0%)	8/ 40 
( 20.0%)	8/163 
(  4.9%)	
Undetectable at EOTa	42/123 
( 34.1%)	9/ 40 
( 22.5%)	51/163 
( 31.3%)	
Completed all study therapy	42/123 
( 34.1%)	6/ 40 
( 15.0%)	48/163 
( 29.4%)	
Discontinued at least one study therapy (any other case)	0/123 
(  0.0%)	3/ 40 
(  7.5%)	3/163 
(  1.8%)	
Adverse event	0/123 
(  0.0%)	2/ 40 
(  5.0%)	2/163 
(  1.2%)	
Other	0/123 
(  0.0%)	0/ 40 
(  0.0%)	0/163 
(  0.0%)	
Subject reached a virologic endpoint	0/123 
(  0.0%)	1/ 40 
(  2.5%)	1/163 
(  0.6%)	
HCV RNA >= 25 at Timepoint of SVR	39/123 
( 31.7%)	9/ 40 
( 22.5%)	48/163 
( 29.4%)	
Completed all study therapy	39/123 
( 31.7%)	6/ 40 
( 15.0%)	45/163 
( 27.6%)	
Discontinued at least one study therapy (any other case)	0/123 
(  0.0%)	3/ 40 
(  7.5%)	3/163 
(  1.8%)	
Adverse event	0/123 
(  0.0%)	2/ 40 
(  5.0%)	2/163 
(  1.2%)	
Other	0/123 
(  0.0%)	0/ 40 
(  0.0%)	0/163 
(  0.0%)	
Subject reached a virologic endpoint	0/123 
(  0.0%)	1/ 40 
(  2.5%)	1/163 
(  0.6%)	
Missing at Timepoint of SVR	3/123 
(  2.4%)	0/ 40 
(  0.0%)	3/163 
(  1.8%)	
Completed all study therapy	3/123 
(  2.4%)	0/ 40 
(  0.0%)	3/163 
(  1.8%)	
	
SVR24				
Total with SVR24	79/123 
( 64.2%)	21/ 40 
( 52.5%)	100/163 
( 61.3%)	
Total without SVR24 for any reason	44/123 
( 35.8%)	19/ 40 
( 47.5%)	63/163 
( 38.7%)	
Detectable at EOT	0/123 
(  0.0%)	10/ 40 
( 25.0%)	10/163 
(  6.1%)	
Discontinued at least one study therapy (any other case)	0/123 
(  0.0%)	10/ 40 
( 25.0%)	10/163 
(  6.1%)	
Adverse event	0/123 
(  0.0%)	2/ 40 
(  5.0%)	2/163 
(  1.2%)	
Subject reached a virologic endpoint	0/123 
(  0.0%)	8/ 40 
( 20.0%)	8/163 
(  4.9%)	
Undetectable at EOTa	44/123 
( 35.8%)	9/ 40 
( 22.5%)	53/163 
( 32.5%)	
Completed all study therapy	43/123 
( 35.0%)	6/ 40 
( 15.0%)	49/163 
( 30.1%)	
Discontinued at least one study therapy (any other case)	1/123 
(  0.8%)	3/ 40 
(  7.5%)	4/163 
(  2.5%)	
Adverse event	0/123 
(  0.0%)	2/ 40 
(  5.0%)	2/163 
(  1.2%)	
Other	1/123 
(  0.8%)	0/ 40 
(  0.0%)	1/163 
(  0.6%)	
Subject reached a virologic endpoint	0/123 
(  0.0%)	1/ 40 
(  2.5%)	1/163 
(  0.6%)	
HCV RNA >= 25 at Timepoint of SVR	41/123 
( 33.3%)	9/ 40 
( 22.5%)	50/163 
( 30.7%)	
Completed all study therapy	40/123 
( 32.5%)	6/ 40 
( 15.0%)	46/163 
( 28.2%)	
Discontinued at least one study therapy (any other case)	1/123 
(  0.8%)	3/ 40 
(  7.5%)	4/163 
(  2.5%)	
Adverse event	0/123 
(  0.0%)	2/ 40 
(  5.0%)	2/163 
(  1.2%)	
Other	1/123 
(  0.8%)	0/ 40 
(  0.0%)	1/163 
(  0.6%)	
Subject reached a virologic endpoint	0/123 
(  0.0%)	1/ 40 
(  2.5%)	1/163 
(  0.6%)	
Missing at Timepoint of SVR	3/123 
(  2.4%)	0/ 40 
(  0.0%)	3/163 
(  1.8%)	
Completed all study therapy	3/123 
(  2.4%)	0/ 40 
(  0.0%)	3/163 
(  1.8%)	
	

a	Combination of the 2 categories: HCV RNA >= 25 IU/mL at Timepoint of SVR and Missing at Timepoint of SVR	
[TEFSVR03.rtf] [\STAT\Analyses\Programs\FinalAnalysis\Final1\2.TLF\2.Efficacy\EFF_FA.sas] 23OCT2015, 18:04	
