# Supplementary material for: An Open-Label Trial of 12-Week Simeprevir plus Peginterferon/Ribavirin (PR) in Treatment-Naïve Patients with Hepatitis C Virus (HCV) Genotype 1 (GT1)
Source: PLoS One. 2016 Jul 18;11(7):e0158526. doi: 10.1371/journal.pone.0158526 (PMC4948848; doi:10.1371/journal.pone.0158526)
Supplement: S1 Dataset — (ZIP) [file pone.0158526.s009.zip › TEFSVR08_4.rtf]

TEFSVR08_4:	Sustained Virologic Response 4 Weeks After the Planned End of Treatment; Genotype 1 (Study TMC435HPC3014)	
	Simeprevir
12 Wks
150 mg
PR 12/24 	
	 Genotype 1  	
	 12 Wks 	 >12 Wks 	 All subjects 	
SVR4=yes %(n/N)	88.6% (109/123)	60.0% (24/40)	81.6% (133/163)	
95% CI	( 81.6%- 93.6%)	( 43.3%- 75.1%)	( 74.8%- 87.2%)	
				
SVR4=no %(n/N)	10.6% (13/123)	40.0% (16/40)	17.8% (29/163)	
95% CI	( 5.7%- 17.4%)	( 24.9%- 56.7%)	( 12.3%- 24.5%)	
				
SVR4=missing %(n/N)	0.8% (1/123)	(0/40)	0.6% (1/163)	
95% CI	( 0.0%- 4.4%)		( 0.0%- 3.4%)	
				
	
[TEFSVR08_4.RTF] [TMC435\HPC3014\DBR_FINAL_ANALYSIS\RE_FINAL_ANALYSIS\PDEV\TEFSVR08_4.SAS] 27MAY2016, 14:29	
