# Supplementary material for: An Open-Label Trial of 12-Week Simeprevir plus Peginterferon/Ribavirin (PR) in Treatment-Naïve Patients with Hepatitis C Virus (HCV) Genotype 1 (GT1)
Source: PLoS One. 2016 Jul 18;11(7):e0158526. doi: 10.1371/journal.pone.0158526 (PMC4948848; doi:10.1371/journal.pone.0158526)
Supplement: S1 Dataset — (ZIP) [file pone.0158526.s009.zip › TEFSVR08_12.rtf]

TEFSVR08_12:	Sustained Virologic Response 12 Weeks After the Planned End of Treatment; Genotype 1 (Study TMC435HPC3014)	
	Simeprevir
12 Wks
150 mg
PR 12/24 	
	 Genotype 1  	
	 12 Wks 	 >12 Wks 	 All subjects 	
SVR12=yes %(n/N)	65.9% (81/123)	52.5% (21/40)	62.6% (102/163)	
95% CI	( 56.8%- 74.2%)	( 36.1%- 68.5%)	( 54.7%- 70.0%)	
				
SVR12=no %(n/N)	31.7% (39/123)	47.5% (19/40)	35.6% (58/163)	
95% CI	( 23.6%- 40.7%)	( 31.5%- 63.9%)	( 28.3%- 43.4%)	
				
SVR12=missing %(n/N)	2.4% (3/123)	(0/40)	1.8% (3/163)	
95% CI	( 0.5%- 7.0%)		( 0.4%- 5.3%)	
				
	
[TEFSVR08_12.RTF] [TMC435\HPC3014\DBR_FINAL_ANALYSIS\RE_FINAL_ANALYSIS\PDEV\TEFSVR08_12.SAS] 27MAY2016, 14:26	
