# Supplementary material for: An Open-Label Trial of 12-Week Simeprevir plus Peginterferon/Ribavirin (PR) in Treatment-Naïve Patients with Hepatitis C Virus (HCV) Genotype 1 (GT1)
Source: PLoS One. 2016 Jul 18;11(7):e0158526. doi: 10.1371/journal.pone.0158526 (PMC4948848; doi:10.1371/journal.pone.0158526)
Supplement: S1 Dataset — (ZIP) [file pone.0158526.s009.zip › TEFVBT01.rtf]

TEFVBT01:	Viral Breakthrough; Intent-to-treat (Study TMC435HPC3014)
Treatment Group: Simeprevir 12Wks 150 mg PR12/24	
	Genotype 1	
	12 Weeks 
Treatment	>12 Weeks 
Treatment	All Subjects	
Analysis set: intent-to-treat	123	40	163	
	
Viral Breakthrough during triple therapy or PR only phase	0/123 
(  0.0%)	4/ 39 
( 10.3%)	4/162 
(  2.5%)	
	
By treatment period				
Viral Breakthrough during triple therapy phase	0/123 
(  0.0%)	3/ 39 
(  7.7%)	3/162 
(  1.9%)	
Viral Breakthrough during PR only phase	- 
	1/ 29 
(  3.4%)	1/ 29 
(  3.4%)	
	
	
[TEFVBT01.rtf] [\STAT\Analyses\Programs\FinalAnalysis\Final1\2.TLF\2.Efficacy\EFF_FA.sas] 23OCT2015, 18:04	
