# Supplementary material for: An Open-Label Trial of 12-Week Simeprevir plus Peginterferon/Ribavirin (PR) in Treatment-Naïve Patients with Hepatitis C Virus (HCV) Genotype 1 (GT1)
Source: PLoS One. 2016 Jul 18;11(7):e0158526. doi: 10.1371/journal.pone.0158526 (PMC4948848; doi:10.1371/journal.pone.0158526)
Supplement: S1 Dataset — (ZIP) [file pone.0158526.s009.zip › TEFVF01.rtf]

TEFVF01:	On-Treatment and Post-Treatment Failure; Intent-to-treat (Study TMC435HPC3014)
Treatment Group = Simeprevir 12Wks 150 mg PR12/24	
	Genotype 1	
	12 Weeks 
Treatment	>12 Weeks 
Treatment	All Subjects	
Analysis set: intent-to-treat	123	40	163	
	
Failurea	45/123 
( 36.6%)	19/ 40 
( 47.5%)	64/163 
( 39.3%)	
On-treatment failureb	0/123 
(  0.0%)	10/ 40 
( 25.0%)	10/163 
(  6.1%)	
Discontinued PegIFN and RBV	0/123 
(  0.0%)	10/ 40 
( 25.0%)	10/163 
(  6.1%)	
Met a stopping rule at Week 4	0/123 
(  0.0%)	7/ 40 
( 17.5%)	7/163 
(  4.3%)	
Other	0/123 
(  0.0%)	2/ 40 
(  5.0%)	2/163 
(  1.2%)	
Viral breakthrough	0/123 
(  0.0%)	1/ 40 
(  2.5%)	1/163 
(  0.6%)	
Post-treatment failurec	45/123 
( 36.6%)	9/ 40 
( 22.5%)	54/163 
( 33.1%)	
Missing at timepoint of SVR12d	3/123 
(  2.4%)	0/ 40 
(  0.0%)	3/163 
(  1.8%)	
Viral relapse	42/123 
( 34.1%)	9/ 40 
( 22.5%)	51/163 
( 31.3%)	
Completed PegIFN and/or RBV	42/123 
( 34.1%)	6/ 40 
( 15.0%)	48/163 
( 29.4%)	
Discontinued PegIFN and RBV	0/123 
(  0.0%)	3/ 40 
(  7.5%)	3/163 
(  1.8%)	
	

Note: A subject can occur in only one category.
	Stopping rules: HCV RNA >= 25 IU/ml at the week 4 visit, HCV RNA >= 25 IU/mL or detectable at the week 12 visit or
	subject has a viral breakthrough. Subjects who met a stopping rule had to discontinue all treatment.
a	Subject did not achieve SVR12 or achieved SVR12 and had a relapse thereafter.
b	Confirmed detectable HCV RNA levels at actual EOT.
c	Failure but with undetectable (or unconfirmed detectable) HCV RNA levels at EOT
d	Subjects with on-treatment response, without viral relapse, but who fail solely because of missing data at the time point
	of SVR12 (and thereafter)
Subject with CRF ID TMC435HPC3014-0043 achieved HCV RNA <25 iu/ml detected at last study related visit (week 36) 
after previously having experienced a viral relapse. This subject will be further described in the CSR.	
[TEFVF01.rtf] [\STAT\Analyses\Programs\FinalAnalysis\Final1\2.TLF\2.Efficacy\EFF_FA.sas] 23OCT2015, 18:04	
