# Supplementary material for: An Open-Label Trial of 12-Week Simeprevir plus Peginterferon/Ribavirin (PR) in Treatment-Naïve Patients with Hepatitis C Virus (HCV) Genotype 1 (GT1)
Source: PLoS One. 2016 Jul 18;11(7):e0158526. doi: 10.1371/journal.pone.0158526 (PMC4948848; doi:10.1371/journal.pone.0158526)
Supplement: S1 Dataset — (ZIP) [file pone.0158526.s009.zip › TSIDEM01A.RTF]

TSIDEM01A:	Demographic Characteristics; Intent-to-treat (Study TMC435HPC3014)
Treatment Group = Simeprevir 12Wks 150 mg PR12/24	
	Genotype 1	
	12 Weeks 
Treatment	>12 Weeks 
Treatment	All Subjects	
Analysis set: intent-to-treat	123	40	163	
	
Gender				
N	123	40	163	
Female	58 (47.2%)	12 (30.0%)	70 (42.9%)	
Male	65 (52.8%)	28 (70.0%)	93 (57.1%)	
	
Age (years)				
N	123	40	163	
Mean (SD)	45.4 (10.57)	48.2 (9.70)	46.1 (10.40)	
Median	47.0	49.5	47.0	
Range	(23; 68)	(26; 64)	(23; 68)	
	
Age (years)				
N	123	40	163	
≤45 years	52 (42.3%)	15 (37.5%)	67 (41.1%)	
>45 - ≤65 years	68 (55.3%)	25 (62.5%)	93 (57.1%)	
>65 years	3 (2.4%)	0	3 (1.8%)	
	
Race				
Not allowed to ask per local regulations	16	7	23	
N	107	33	140	
Asian	3 (2.8%)	0	3 (2.1%)	
Black or African American	5 (4.7%)	1 (3.0%)	6 (4.3%)	
Multiple	0	0	0	
Native Hawaiian or Other Pacific Islander	1 (0.9%)	0	1 (0.7%)	
White	98 (91.6%)	32 (97.0%)	130 (92.9%)	
	
Ethnicity				
Not allowed to ask per local regulations	16	7	23	
N	107	33	140	
Hispanic or Latino	3 (2.8%)	0	3 (2.1%)	
Not Hispanic or Latino	104 (97.2%)	33 (100.0%)	137 (97.9%)	
	
Region				
N	123	40	163	
Europe	123 (100.0%)	40 (100.0%)	163 (100.0%)	
Middle-east/North-africa	0	0	0	
	
Country				
N	123	40	163	
Austria	17 (13.8%)	5 (12.5%)	22 (13.5%)	
Belgium	14 (11.4%)	2 (5.0%)	16 (9.8%)	
France	29 (23.6%)	8 (20.0%)	37 (22.7%)	
Germany	23 (18.7%)	5 (12.5%)	28 (17.2%)	
Italy	13 (10.6%)	6 (15.0%)	19 (11.7%)	
Saudi Arabia	0	0	0	
Spain	18 (14.6%)	12 (30.0%)	30 (18.4%)	
United Kingdom	9 (7.3%)	2 (5.0%)	11 (6.7%)	
	
Origin				
N	0	0	0	
Europe	0	0	0	
Middle-East/North-Africa	0	0	0	
Other regions	0	0	0	
	
Body weight (kg)				
N	123	40	163	
Mean (SD)	74.10 (15.170)	74.72 (15.740)	74.25 (15.265)	
Median	73.40	76.00	73.50	
Range	(42.5; 110.0)	(37.8; 103.0)	(37.8; 110.0)	
	
Body mass index (kg/m²)				
N	123	40	163	
Mean (SD)	25.44 (4.384)	25.36 (4.138)	25.42 (4.312)	
Median	25.10	25.45	25.10	
Range	(16.6; 38.9)	(15.7; 33.9)	(15.7; 38.9)	
	
Body mass index (kg/m²)				
N	123	40	163	
<25 kg/m²	60 (48.8%)	19 (47.5%)	79 (48.5%)	
≥25 - <30 kg/m²	49 (39.8%)	16 (40.0%)	65 (39.9%)	
≥30 kg/m²	14 (11.4%)	5 (12.5%)	19 (11.7%)	
	

N = number of subjects with data	
[TSIDEM01A.rtf] [\STAT\Analyses\Programs\FinalAnalysis\Final1\2.TLF\1.General\GEN_FA.sas] 23OCT2015, 16:53	
