# Supplementary material for: An Open-Label Trial of 12-Week Simeprevir plus Peginterferon/Ribavirin (PR) in Treatment-Naïve Patients with Hepatitis C Virus (HCV) Genotype 1 (GT1)
Source: PLoS One. 2016 Jul 18;11(7):e0158526. doi: 10.1371/journal.pone.0158526 (PMC4948848; doi:10.1371/journal.pone.0158526)
Supplement: S1 Dataset — (ZIP) [file pone.0158526.s009.zip › TSIDEM01B.RTF]

TSIDEM01B:	Demographic Characteristics by Subgroups of Interest; Intent-to-treat (Study TMC435HPC3014)
Treatment Group = Simeprevir 12Wks 150 mg PR12/24 
HCV Geno/Subtype = 1a/other	
	Genotype 1	
	12 Weeks 
Treatment	>12 Weeks 
Treatment	All Subjects	
Analysis set: intent-to-treat	49	18	67	
	
Gender				
N	49	18	67	
Female	20 (40.8%)	5 (27.8%)	25 (37.3%)	
Male	29 (59.2%)	13 (72.2%)	42 (62.7%)	
	
Age (years)				
N	49	18	67	
Mean	44.8	45.6	45.0	
SE	1.36	2.05	1.13	
SD	9.55	8.70	9.27	
95% C.I. *	(42.01; 47.50)	(41.29; 49.94)	(42.72; 47.25)	
Min	25	26	25	
Q1	40.0	43.0	40.0	
Median	47.0	46.0	47.0	
Q3	50.0	52.0	51.0	
Max	66	59	66	
	
Age (years)				
N	49	18	67	
≤45 years	20 (40.8%)	7 (38.9%)	27 (40.3%)	
>45 - ≤65 years	28 (57.1%)	11 (61.1%)	39 (58.2%)	
>65 years	1 (2.0%)	0	1 (1.5%)	
	
Race				
Not allowed to ask per local regulations	7	4	11	
N	42	14	56	
Asian	1 (2.4%)	0	1 (1.8%)	
Black or African American	2 (4.8%)	0	2 (3.6%)	
Native Hawaiian or Other Pacific Islander	1 (2.4%)	0	1 (1.8%)	
White	38 (90.5%)	14 (100.0%)	52 (92.9%)	
	
Ethnicity				
Not allowed to ask per local regulations	7	4	11	
N	42	14	56	
Not Hispanic or Latino	42 (100.0%)	14 (100.0%)	56 (100.0%)	
	
Region				
N	49	18	67	
Europe	49 (100.0%)	18 (100.0%)	67 (100.0%)	
	
Country				
N	49	18	67	
Austria	13 (26.5%)	3 (16.7%)	16 (23.9%)	
Belgium	5 (10.2%)	1 (5.6%)	6 (9.0%)	
France	14 (28.6%)	5 (27.8%)	19 (28.4%)	
Germany	7 (14.3%)	3 (16.7%)	10 (14.9%)	
Italy	2 (4.1%)	1 (5.6%)	3 (4.5%)	
Spain	4 (8.2%)	4 (22.2%)	8 (11.9%)	
United Kingdom	4 (8.2%)	1 (5.6%)	5 (7.5%)	
	
Body weight (kg)				
N	49	18	67	
Mean	73.74	76.96	74.61	
SE	2.263	3.567	1.907	
SD	15.843	15.133	15.607	
95% C.I. *	(69.194; 78.295)	(69.436; 84.487)	(70.802; 78.416)	
Min	42.5	51.0	42.5	
Q1	61.00	65.00	63.00	
Median	73.50	76.00	74.00	
Q3	85.00	86.50	86.50	
Max	110.0	103.0	110.0	
	
Body mass index (kg/m²)				
N	49	18	67	
Mean	24.98	25.96	25.24	
SE	0.586	0.787	0.478	
SD	4.099	3.340	3.909	
95% C.I. *	(23.806; 26.161)	(24.295; 27.616)	(24.291; 26.198)	
Min	16.6	19.0	16.6	
Q1	21.70	23.60	22.10	
Median	24.70	25.90	24.80	
Q3	27.70	29.00	28.10	
Max	33.9	31.1	33.9	
	
Body mass index (kg/m²)				
N	49	18	67	
<25 kg/m²	26 (53.1%)	8 (44.4%)	34 (50.7%)	
≥25 - <30 kg/m²	17 (34.7%)	8 (44.4%)	25 (37.3%)	
≥30 kg/m²	6 (12.2%)	2 (11.1%)	8 (11.9%)	
	

* Confidence interval for mean
N = number of subjects with data	
[TSIDEM01B.rtf] [\STAT\Analyses\Programs\FinalAnalysis\Final1\2.TLF\1.General\GEN_FA.sas] 23OCT2015, 16:53	

TSIDEM01B:	Demographic Characteristics by Subgroups of Interest; Intent-to-treat (Study TMC435HPC3014)
Treatment Group = Simeprevir 12Wks 150 mg PR12/24 
HCV Geno/Subtype = 1b	
	Genotype 1	
	12 Weeks 
Treatment	>12 Weeks 
Treatment	All Subjects	
Analysis set: intent-to-treat	74	22	96	
	
Gender				
N	74	22	96	
Female	38 (51.4%)	7 (31.8%)	45 (46.9%)	
Male	36 (48.6%)	15 (68.2%)	51 (53.1%)	
	
Age (years)				
N	74	22	96	
Mean	45.8	50.2	46.8	
SE	1.31	2.17	1.13	
SD	11.24	10.17	11.11	
95% C.I. *	(43.21; 48.41)	(45.72; 54.74)	(44.57; 49.07)	
Min	23	30	23	
Q1	38.0	43.0	39.0	
Median	47.0	51.5	47.5	
Q3	53.0	57.0	56.0	
Max	68	64	68	
	
Age (years)				
N	74	22	96	
≤45 years	32 (43.2%)	8 (36.4%)	40 (41.7%)	
>45 - ≤65 years	40 (54.1%)	14 (63.6%)	54 (56.3%)	
>65 years	2 (2.7%)	0	2 (2.1%)	
	
Race				
Not allowed to ask per local regulations	9	3	12	
N	65	19	84	
Asian	2 (3.1%)	0	2 (2.4%)	
Black or African American	3 (4.6%)	1 (5.3%)	4 (4.8%)	
White	60 (92.3%)	18 (94.7%)	78 (92.9%)	
	
Ethnicity				
Not allowed to ask per local regulations	9	3	12	
N	65	19	84	
Hispanic or Latino	3 (4.6%)	0	3 (3.6%)	
Not Hispanic or Latino	62 (95.4%)	19 (100.0%)	81 (96.4%)	
	
Region				
N	74	22	96	
Europe	74 (100.0%)	22 (100.0%)	96 (100.0%)	
	
Country				
N	74	22	96	
Austria	4 (5.4%)	2 (9.1%)	6 (6.3%)	
Belgium	9 (12.2%)	1 (4.5%)	10 (10.4%)	
France	15 (20.3%)	3 (13.6%)	18 (18.8%)	
Germany	16 (21.6%)	2 (9.1%)	18 (18.8%)	
Italy	11 (14.9%)	5 (22.7%)	16 (16.7%)	
Spain	14 (18.9%)	8 (36.4%)	22 (22.9%)	
United Kingdom	5 (6.8%)	1 (4.5%)	6 (6.3%)	
	
Body weight (kg)				
N	74	22	96	
Mean	74.33	72.88	74.00	
SE	1.722	3.483	1.541	
SD	14.814	16.335	15.099	
95% C.I. *	(70.898; 77.762)	(65.635; 80.120)	(70.937; 77.056)	
Min	47.0	37.8	37.8	
Q1	65.00	57.30	64.60	
Median	72.50	74.00	72.50	
Q3	82.20	85.00	83.50	
Max	107.0	99.2	107.0	
	
Body mass index (kg/m²)				
N	74	22	96	
Mean	25.75	24.88	25.55	
SE	0.531	1.005	0.468	
SD	4.564	4.715	4.589	
95% C.I. *	(24.691; 26.806)	(22.787; 26.968)	(24.619; 26.479)	
Min	17.2	15.7	15.7	
Q1	22.50	21.30	22.05	
Median	25.15	25.10	25.15	
Q3	28.30	27.60	28.05	
Max	38.9	33.9	38.9	
	
Body mass index (kg/m²)				
N	74	22	96	
<25 kg/m²	34 (45.9%)	11 (50.0%)	45 (46.9%)	
≥25 - <30 kg/m²	32 (43.2%)	8 (36.4%)	40 (41.7%)	
≥30 kg/m²	8 (10.8%)	3 (13.6%)	11 (11.5%)	
	

* Confidence interval for mean
N = number of subjects with data	
[TSIDEM01B.rtf] [\STAT\Analyses\Programs\FinalAnalysis\Final1\2.TLF\1.General\GEN_FA.sas] 23OCT2015, 16:53	

TSIDEM01B:	Demographic Characteristics by Subgroups of Interest; Intent-to-treat (Study TMC435HPC3014)
Treatment Group = Simeprevir 12Wks 150 mg PR12/24 
IL28b = CC	
	Genotype 1	
	12 Weeks 
Treatment	>12 Weeks 
Treatment	All Subjects	
Analysis set: intent-to-treat	32	8	40	
	
Gender				
N	32	8	40	
Female	15 (46.9%)	3 (37.5%)	18 (45.0%)	
Male	17 (53.1%)	5 (62.5%)	22 (55.0%)	
	
Age (years)				
N	32	8	40	
Mean	45.3	45.8	45.4	
SE	1.88	3.96	1.68	
SD	10.65	11.21	10.61	
95% C.I. *	(41.51; 49.18)	(36.38; 55.12)	(42.03; 48.82)	
Min	24	32	24	
Q1	36.5	35.0	36.0	
Median	48.5	47.0	48.5	
Q3	52.5	53.0	52.5	
Max	64	64	64	
	
Age (years)				
N	32	8	40	
≤45 years	13 (40.6%)	4 (50.0%)	17 (42.5%)	
>45 - ≤65 years	19 (59.4%)	4 (50.0%)	23 (57.5%)	
	
Race				
Not allowed to ask per local regulations	7	3	10	
N	25	5	30	
Asian	3 (12.0%)	0	3 (10.0%)	
Black or African American	0	0	0	
Multiple	0	0	0	
White	22 (88.0%)	5 (100.0%)	27 (90.0%)	
	
Ethnicity				
Not allowed to ask per local regulations	7	3	10	
N	25	5	30	
Not Hispanic or Latino	25 (100.0%)	5 (100.0%)	30 (100.0%)	
	
Region				
N	32	8	40	
Europe	32 (100.0%)	8 (100.0%)	40 (100.0%)	
Middle-east/North-africa	0	0	0	
	
Country				
N	32	8	40	
Austria	3 (9.4%)	0	3 (7.5%)	
Belgium	1 (3.1%)	0	1 (2.5%)	
France	9 (28.1%)	3 (37.5%)	12 (30.0%)	
Germany	6 (18.8%)	2 (25.0%)	8 (20.0%)	
Italy	6 (18.8%)	0	6 (15.0%)	
Saudi Arabia	0	0	0	
Spain	4 (12.5%)	2 (25.0%)	6 (15.0%)	
United Kingdom	3 (9.4%)	1 (12.5%)	4 (10.0%)	
	
Origin				
N	0	0	0	
Europe	0	0	0	
Middle-East/North-Africa	0	0	0	
Other regions	0	0	0	
	
Body weight (kg)				
N	32	8	40	
Mean	71.20	70.89	71.14	
SE	2.738	3.915	2.306	
SD	15.487	11.073	14.583	
95% C.I. *	(65.613; 76.780)	(61.631; 80.144)	(66.471; 75.799)	
Min	46.0	51.0	46.0	
Q1	56.90	64.00	58.90	
Median	71.15	73.05	71.15	
Q3	83.60	77.75	81.10	
Max	99.0	86.5	99.0	
	
Body mass index (kg/m²)				
N	32	8	40	
Mean	24.34	25.15	24.51	
SE	0.579	0.906	0.495	
SD	3.274	2.562	3.131	
95% C.I. *	(23.163; 25.524)	(23.008; 27.292)	(23.504; 25.506)	
Min	19.2	21.5	19.2	
Q1	21.65	23.55	21.90	
Median	24.10	24.75	24.55	
Q3	27.10	26.95	27.10	
Max	29.8	29.2	29.8	
	
Body mass index (kg/m²)				
N	32	8	40	
<25 kg/m²	17 (53.1%)	5 (62.5%)	22 (55.0%)	
≥25 - <30 kg/m²	15 (46.9%)	3 (37.5%)	18 (45.0%)	
≥30 kg/m²	0	0	0	
	

* Confidence interval for mean
N = number of subjects with data	
[TSIDEM01B.rtf] [\STAT\Analyses\Programs\FinalAnalysis\Final1\2.TLF\1.General\GEN_FA.sas] 23OCT2015, 16:53	

TSIDEM01B:	Demographic Characteristics by Subgroups of Interest; Intent-to-treat (Study TMC435HPC3014)
Treatment Group = Simeprevir 12Wks 150 mg PR12/24 
IL28b = CT	
	Genotype 1	
	12 Weeks 
Treatment	>12 Weeks 
Treatment	All Subjects	
Analysis set: intent-to-treat	73	20	93	
	
Gender				
N	73	20	93	
Female	36 (49.3%)	4 (20.0%)	40 (43.0%)	
Male	37 (50.7%)	16 (80.0%)	53 (57.0%)	
	
Age (years)				
N	73	20	93	
Mean	45.2	47.7	45.7	
SE	1.33	2.01	1.13	
SD	11.39	9.01	10.93	
95% C.I. *	(42.52; 47.84)	(43.48; 51.92)	(43.47; 47.97)	
Min	23	26	23	
Q1	38.0	44.0	40.0	
Median	47.0	48.0	47.0	
Q3	53.0	53.5	53.0	
Max	68	62	68	
	
Age (years)				
N	73	20	93	
≤45 years	32 (43.8%)	8 (40.0%)	40 (43.0%)	
>45 - ≤65 years	38 (52.1%)	12 (60.0%)	50 (53.8%)	
>65 years	3 (4.1%)	0	3 (3.2%)	
	
Race				
Not allowed to ask per local regulations	7	4	11	
N	66	16	82	
Asian	0	0	0	
Black or African American	5 (7.6%)	0	5 (6.1%)	
Native Hawaiian or Other Pacific Islander	1 (1.5%)	0	1 (1.2%)	
White	60 (90.9%)	16 (100.0%)	76 (92.7%)	
	
Ethnicity				
Not allowed to ask per local regulations	7	4	11	
N	66	16	82	
Hispanic or Latino	3 (4.5%)	0	3 (3.7%)	
Not Hispanic or Latino	63 (95.5%)	16 (100.0%)	79 (96.3%)	
	
Region				
N	73	20	93	
Europe	73 (100.0%)	20 (100.0%)	93 (100.0%)	
Middle-east/North-africa	0	0	0	
	
Country				
N	73	20	93	
Austria	13 (17.8%)	3 (15.0%)	16 (17.2%)	
Belgium	8 (11.0%)	2 (10.0%)	10 (10.8%)	
France	16 (21.9%)	5 (25.0%)	21 (22.6%)	
Germany	10 (13.7%)	2 (10.0%)	12 (12.9%)	
Italy	7 (9.6%)	3 (15.0%)	10 (10.8%)	
Saudi Arabia	0	0	0	
Spain	14 (19.2%)	5 (25.0%)	19 (20.4%)	
United Kingdom	5 (6.8%)	0	5 (5.4%)	
	
Origin				
N	0	0	0	
Europe	0	0	0	
Middle-East/North-Africa	0	0	0	
	
Body weight (kg)				
N	73	20	93	
Mean	74.68	76.90	75.16	
SE	1.800	4.020	1.648	
SD	15.379	17.976	15.896	
95% C.I. *	(71.093; 78.269)	(68.487; 85.313)	(71.884; 78.432)	
Min	42.5	37.8	37.8	
Q1	67.00	65.95	67.00	
Median	73.00	76.00	73.40	
Q3	84.10	92.50	87.00	
Max	110.0	103.0	110.0	
	
Body mass index (kg/m²)				
N	73	20	93	
Mean	25.57	25.27	25.50	
SE	0.537	1.042	0.475	
SD	4.585	4.658	4.577	
95% C.I. *	(24.499; 26.638)	(23.090; 27.450)	(24.562; 26.447)	
Min	16.6	15.7	15.7	
Q1	22.20	22.50	22.20	
Median	24.80	25.25	24.90	
Q3	28.30	29.40	28.40	
Max	36.8	33.9	36.8	
	
Body mass index (kg/m²)				
N	73	20	93	
<25 kg/m²	37 (50.7%)	10 (50.0%)	47 (50.5%)	
≥25 - <30 kg/m²	25 (34.2%)	6 (30.0%)	31 (33.3%)	
≥30 kg/m²	11 (15.1%)	4 (20.0%)	15 (16.1%)	
	

* Confidence interval for mean
N = number of subjects with data	
[TSIDEM01B.rtf] [\STAT\Analyses\Programs\FinalAnalysis\Final1\2.TLF\1.General\GEN_FA.sas] 23OCT2015, 16:53	

TSIDEM01B:	Demographic Characteristics by Subgroups of Interest; Intent-to-treat (Study TMC435HPC3014)
Treatment Group = Simeprevir 12Wks 150 mg PR12/24 
IL28b = TT	
	Genotype 1	
	12 Weeks 
Treatment	>12 Weeks 
Treatment	All Subjects	
Analysis set: intent-to-treat	18	12	30	
	
Gender				
N	18	12	30	
Female	7 (38.9%)	5 (41.7%)	12 (40.0%)	
Male	11 (61.1%)	7 (58.3%)	18 (60.0%)	
	
Age (years)				
N	18	12	30	
Mean	46.3	50.5	48.0	
SE	1.57	2.93	1.52	
SD	6.67	10.15	8.33	
95% C.I. *	(43.02; 49.65)	(44.05; 56.95)	(44.89; 51.11)	
Min	32	30	30	
Q1	43.0	44.5	43.0	
Median	47.0	53.5	47.5	
Q3	48.0	57.0	56.0	
Max	62	64	64	
	
Age (years)				
N	18	12	30	
≤45 years	7 (38.9%)	3 (25.0%)	10 (33.3%)	
>45 - ≤65 years	11 (61.1%)	9 (75.0%)	20 (66.7%)	
	
Race				
Not allowed to ask per local regulations	2	0	2	
N	16	12	28	
Black or African American	0	1 (8.3%)	1 (3.6%)	
White	16 (100.0%)	11 (91.7%)	27 (96.4%)	
	
Ethnicity				
Not allowed to ask per local regulations	2	0	2	
N	16	12	28	
Not Hispanic or Latino	16 (100.0%)	12 (100.0%)	28 (100.0%)	
	
Region				
N	18	12	30	
Europe	18 (100.0%)	12 (100.0%)	30 (100.0%)	
Middle-east/North-africa	0	0	0	
	
Country				
N	18	12	30	
Austria	1 (5.6%)	2 (16.7%)	3 (10.0%)	
Belgium	5 (27.8%)	0	5 (16.7%)	
France	4 (22.2%)	0	4 (13.3%)	
Germany	7 (38.9%)	1 (8.3%)	8 (26.7%)	
Italy	0	3 (25.0%)	3 (10.0%)	
Saudi Arabia	0	0	0	
Spain	0	5 (41.7%)	5 (16.7%)	
United Kingdom	1 (5.6%)	1 (8.3%)	2 (6.7%)	
	
Origin				
N	0	0	0	
Europe	0	0	0	
Middle-East/North-Africa	0	0	0	
	
Body weight (kg)				
N	18	12	30	
Mean	76.88	73.63	75.58	
SE	3.223	4.297	2.558	
SD	13.674	14.884	14.010	
95% C.I. *	(70.084; 83.683)	(64.168; 83.082)	(70.349; 80.811)	
Min	55.7	50.0	50.0	
Q1	66.80	62.00	66.80	
Median	76.00	74.20	76.00	
Q3	80.90	85.20	85.00	
Max	106.0	94.0	106.0	
	
Body mass index (kg/m²)				
N	18	12	30	
Mean	26.89	25.66	26.40	
SE	1.173	1.256	0.858	
SD	4.976	4.350	4.698	
95% C.I. *	(24.420; 29.369)	(22.895; 28.422)	(24.646; 28.154)	
Min	18.4	19.0	18.4	
Q1	24.30	21.60	23.50	
Median	26.55	26.90	26.60	
Q3	29.40	28.00	28.20	
Max	38.9	33.5	38.9	
	
Body mass index (kg/m²)				
N	18	12	30	
<25 kg/m²	6 (33.3%)	4 (33.3%)	10 (33.3%)	
≥25 - <30 kg/m²	9 (50.0%)	7 (58.3%)	16 (53.3%)	
≥30 kg/m²	3 (16.7%)	1 (8.3%)	4 (13.3%)	
	

* Confidence interval for mean
N = number of subjects with data	
[TSIDEM01B.rtf] [\STAT\Analyses\Programs\FinalAnalysis\Final1\2.TLF\1.General\GEN_FA.sas] 23OCT2015, 16:53	
