# Supplementary material for: An Open-Label Trial of 12-Week Simeprevir plus Peginterferon/Ribavirin (PR) in Treatment-Naïve Patients with Hepatitis C Virus (HCV) Genotype 1 (GT1)
Source: PLoS One. 2016 Jul 18;11(7):e0158526. doi: 10.1371/journal.pone.0158526 (PMC4948848; doi:10.1371/journal.pone.0158526)
Supplement: S1 Dataset — (ZIP) [file pone.0158526.s009.zip › TSIDEM02.rtf]

TSIDEM02:	Baseline Disease Characteristics; Intent-to-treat (Study TMC435HPC3014)
Treatment Group = Simeprevir 12Wks 150 mg PR12/24	
	Genotype 1	
	12 Weeks 
Treatment	>12 Weeks 
Treatment	All Subjects	
Analysis set: intent-to-treat	123	40	163	
	
Baseline HCV RNA level (IU/mL)				
N	123	40	163	
Mean	4637310.6	6935125.0	5201191.4	
SE	578472.10	1071422.10	513825.45	
SD	6415565.90	6776268.37	6560084.25	
95% C.I. *	(3492167.28; 5782453.86)	(4767969.24; 9102280.76)	(4186532.18; 6215850.65)	
Min	4500	156000	4500	
Q1	604000.0	2065000.0	909000.0	
Median	2400000.0	4475000.0	3000000.0	
Q3	6300000.0	9290000.0	7260000.0	
Max	49300000	28000000	49300000	
	
Baseline log10 HCV RNA level				
N	123	40	163	
Mean	6.26	6.62	6.35	
SE	0.064	0.080	0.054	
SD	0.713	0.503	0.684	
95% C.I. *	(6.136; 6.391)	(6.456; 6.778)	(6.245; 6.456)	
Min	3.7	5.2	3.7	
Q1	5.78	6.31	5.96	
Median	6.38	6.65	6.48	
Q3	6.80	6.97	6.86	
Max	7.7	7.4	7.7	
	
Baseline HCV RNA level (IU/mL)				
N	123	40	163	
<400000 IU/mL	21 (17.1%)	2 (5.0%)	23 (14.1%)	
≥400000 - ≤800000 IU/mL	12 (9.8%)	1 (2.5%)	13 (8.0%)	
>800000 IU/mL	90 (73.2%)	37 (92.5%)	127 (77.9%)	
	
Metavir fibrosis score a				
N	122	40	162	
Score F0-F1	93 (76.2%)	25 (62.5%)	118 (72.8%)	
Score F2	29 (23.8%)	15 (37.5%)	44 (27.2%)	
Score F3	0	0	0	
	
Baseline ALT level (U/L)				
N	123	40	163	
Mean	62.7	61.0	62.3	
SE	3.50	4.88	2.89	
SD	38.79	30.85	36.91	
95% C.I. *	(55.82; 69.67)	(51.08; 70.82)	(56.60; 68.02)	
Min	16	21	16	
Q1	36.0	38.0	37.0	
Median	54.0	53.5	54.0	
Q3	79.0	73.5	76.0	
Max	228	159	228	
	
Baseline ALT toxicity grade				
N	123	40	163	
Grade 0	54 (43.9%)	17 (42.5%)	71 (43.6%)	
Grade 1	54 (43.9%)	18 (45.0%)	72 (44.2%)	
Grade 2	13 (10.6%)	5 (12.5%)	18 (11.0%)	
Grade 3	2 (1.6%)	0	2 (1.2%)	
	
HCV geno/subtype (coalesce) b				
N	123	40	163	
1a	49 (39.8%)	18 (45.0%)	67 (41.1%)	
1b	74 (60.2%)	22 (55.0%)	96 (58.9%)	
4	0	0	0	
4a	0	0	0	
4a/4c/4d	0	0	0	
4c	0	0	0	
4d	0	0	0	
4e	0	0	0	
4f	0	0	0	
4k	0	0	0	
4n	0	0	0	
4q	0	0	0	
4r	0	0	0	
	
HCV geno/subtype (ns5b)				
N	0	0	0	
4a	0	0	0	
4c	0	0	0	
4d	0	0	0	
4e	0	0	0	
4k	0	0	0	
4n	0	0	0	
4q	0	0	0	
4r	0	0	0	
	
HCV geno/subtype (Trugene assay)					
N	4	1	5	
1a	2 (50.0%)	0	2 (40.0%)	
1b	2 (50.0%)	1 (100.0%)	3 (60.0%)	
4	0	0	0	
4a	0	0	0	
	
HCV geno/subtype (LIPA 2.0 assay)				
N	119	39	158	
1a	47 (39.5%)	18 (46.2%)	65 (41.1%)	
1b	72 (60.5%)	21 (53.8%)	93 (58.9%)	
4	0	0	0	
4a/4c/4d	0	0	0	
4e	0	0	0	
4f	0	0	0	
4h	0	0	0	
	
HCV geno/subtype as stratified				
N	123	40	163	
1a	49 (39.8%)	18 (45.0%)	67 (41.1%)	
1b	74 (60.2%)	22 (55.0%)	96 (58.9%)	
4	0	0	0	
	
Il28b genotype				
N	123	40	163	
CC	32 (26.0%)	8 (20.0%)	40 (24.5%)	
CT	73 (59.3%)	20 (50.0%)	93 (57.1%)	
TT	18 (14.6%)	12 (30.0%)	30 (18.4%)	
	
Duration of HCV infection (years)				
N	56	17	73	
Mean	20.31	19.02	20.01	
SE	1.890	3.643	1.669	
SD	14.147	15.022	14.259	
95% C.I. *	(16.520; 24.097)	(11.300; 26.747)	(16.683; 23.337)	
Min	0.8	1.9	0.8	
Q1	4.45	2.90	4.00	
Median	21.70	19.70	20.80	
Q3	29.90	29.90	29.90	
Max	52.0	47.0	52.0	
	
Time since diagnosis (years)				
N	123	40	163	
Mean	8.52	8.61	8.54	
SE	0.737	1.397	0.651	
SD	8.178	8.833	8.316	
95% C.I. *	(7.062; 9.982)	(5.780; 11.430)	(7.256; 9.829)	
Min	0.4	0.5	0.4	
Q1	1.50	1.10	1.40	
Median	5.00	3.20	4.40	
Q3	15.80	18.40	16.00	
Max	28.9	26.9	28.9	
	
Il28b genotype as stratified				
N	123	40	163	
CC	32 (26.0%)	8 (20.0%)	40 (24.5%)	
CT	73 (59.3%)	20 (50.0%)	93 (57.1%)	
TT	18 (14.6%)	12 (30.0%)	30 (18.4%)	
	
Mode of hepatitis C infection				
N	123	40	163	
Blood transfusion	21 (17.1%)	9 (22.5%)	30 (18.4%)	
Hemophilia-associated injections	1 (0.8%)	0	1 (0.6%)	
Heterosexual contact	4 (3.3%)	1 (2.5%)	5 (3.1%)	
Intravenously injectable drug use	29 (23.6%)	6 (15.0%)	35 (21.5%)	
Mother to child transmission	2 (1.6%)	1 (2.5%)	3 (1.8%)	
Multiple	4 (3.3%)	0	4 (2.5%)	
Other	62 (50.4%)	23 (57.5%)	85 (52.1%)	
	

* Confidence interval for mean
N = number of subjects with data
a	Results from the Metavir scoring system and the non-invasive methods.
b	HCV Geno/Subtype (Coalesce) is based on the NS5B assay, and if not available on LIPA HCV II or Trugene results.	
[TSIDEM02.rtf] [\STAT\Analyses\Programs\FinalAnalysis\Final1\2.TLF\1.General\GEN_FA.sas] 23OCT2015, 16:53	
