# Supplementary material for: An Open-Label Trial of 12-Week Simeprevir plus Peginterferon/Ribavirin (PR) in Treatment-Naïve Patients with Hepatitis C Virus (HCV) Genotype 1 (GT1)
Source: PLoS One. 2016 Jul 18;11(7):e0158526. doi: 10.1371/journal.pone.0158526 (PMC4948848; doi:10.1371/journal.pone.0158526)
Supplement: S1 Dataset — (ZIP) [file pone.0158526.s009.zip › TSIDS01.rtf]

TSIDS01:	Subjects Screened and Treated; All Subjects (Study TMC435HPC3014)	
	Simeprevir
12Wks
150 mg
PR12/24	
	Unknown	Genotype 1	All Subjects	
Screened	5	183	277	
Screen Failure	5 (100.0%)	18 (9.8%)	45 (16.2%)	
Enrolled/Not treated		2 (1.1%)	2 (0.7%)	
Treated		163 (89.1%)	230 (83.0%)	
	
[TSIDS01.rtf] [\STAT\Analyses\Programs\FinalAnalysis\Final1\2.TLF\1.General\GEN_FA.sas] 23OCT2015, 16:53	
