# Supplementary material for: An Open-Label Trial of 12-Week Simeprevir plus Peginterferon/Ribavirin (PR) in Treatment-Naïve Patients with Hepatitis C Virus (HCV) Genotype 1 (GT1)
Source: PLoS One. 2016 Jul 18;11(7):e0158526. doi: 10.1371/journal.pone.0158526 (PMC4948848; doi:10.1371/journal.pone.0158526)
Supplement: S1 Dataset — (ZIP) [file pone.0158526.s009.zip › TSIDS05A.RTF]

TSIDS05A:	Completions and Discontinuations of Study Medication and Reasons for Discontinuation; Intent-to-treat (Study TMC435HPC3014)
Treatment Group = Simeprevir 12Wks 150 mg PR12/24	
	Genotype 1	
	12 Weeks 
Treatment	>12 Weeks 
Treatment	All Subjects	
Analysis set: intent-to-treat	123	40	163	
	
Simeprevir				
N	123	40	163	
Completed	122 
(99.2%)	29 
(72.5%)	151 
(92.6%)	
Discontinued	1 
(0.8%)	11 
(27.5%)	12 
(7.4%)	
Adverse event a		3 
(7.5%)	3 
(1.8%)	
Subject non-compliant	1 
(0.8%)		1 
(0.6%)	
Subject reached a virologic endpoint b		8 
(20.0%)	8 
(4.9%)	
Ribavirin				
N	123	40	163	
Completed	122 
(99.2%)	26 
(65.0%)	148 
(90.8%)	
Discontinued	1 
(0.8%)	14 
(35.0%)	15 
(9.2%)	
Adverse event a		4 
(10.0%)	4 
(2.5%)	
Subject non-compliant	1 
(0.8%)	1 
(2.5%)	2 
(1.2%)	
Subject reached a virologic endpoint b		9 
(22.5%)	9 
(5.5%)	
PegIFN				
N	123	40	163	
Completed	123 
(100.0%)	27 
(67.5%)	150 
(92.0%)	
Discontinued		13 
(32.5%)	13 
(8.0%)	
Adverse event a		4 
(10.0%)	4 
(2.5%)	
Subject non-compliant				
Subject reached a virologic endpoint b		9 
(22.5%)	9 
(5.5%)	
	

a	Adverse event: This category may include subjects who stopped Simeprevir because they had to stop RBV and/or PegIFN
	due to an adverse event.
b	Subject reached a virologic endpoint: subject met a virologic stopping rule.
Information presented in the table is based upon 'Treatment Termination' CRF page (investigator's evaluation).	
[TSIDS05A.rtf] [\STAT\Analyses\Programs\FinalAnalysis\Final1\2.TLF\1.General\GEN_FA.sas] 23OCT2015, 16:53	
