# Supplementary material for: An Open-Label Trial of 12-Week Simeprevir plus Peginterferon/Ribavirin (PR) in Treatment-Naïve Patients with Hepatitis C Virus (HCV) Genotype 1 (GT1)
Source: PLoS One. 2016 Jul 18;11(7):e0158526. doi: 10.1371/journal.pone.0158526 (PMC4948848; doi:10.1371/journal.pone.0158526)
Supplement: S1 Dataset — (ZIP) [file pone.0158526.s009.zip › TSIDS05B.RTF]

TSIDS05B:	Completions and Discontinuations of the Trial and Reasons for Discontinuation; Intent-to-treat (Study TMC435HPC3014)
Treatment Group = Simeprevir 12Wks 150 mg PR12/24	
	Genotype 1	
	12 Weeks 
Treatment	>12 Weeks 
Treatment	All Subjects	
Trial				
N	123	40	163	
Completed	114 
(92.7%)	33 
(82.5%)	147 
(90.2%)	
Discontinued	9 
(7.3%)	7 
(17.5%)	16 
(9.8%)	
Lost to follow-up	5 
(4.1%)	2 
(5.0%)	7 
(4.3%)	
Other	1 
(0.8%)		1 
(0.6%)	
Subject entered another investigational trial	2 
(1.6%)	1 
(2.5%)	3 
(1.8%)	
Subject non-compliant				
Withdrawal by subject	1 
(0.8%)	4 
(10.0%)	5 
(3.1%)	
	

Information presented in the table is based upon 'Treatment Termination' CRF page (investigator's evaluation).	
[TSIDS05B.rtf] [\STAT\Analyses\Programs\FinalAnalysis\Final1\2.TLF\1.General\GEN_FA.sas] 23OCT2015, 16:53	
