# Supplementary material for: An Open-Label Trial of 12-Week Simeprevir plus Peginterferon/Ribavirin (PR) in Treatment-Naïve Patients with Hepatitis C Virus (HCV) Genotype 1 (GT1)
Source: PLoS One. 2016 Jul 18;11(7):e0158526. doi: 10.1371/journal.pone.0158526 (PMC4948848; doi:10.1371/journal.pone.0158526)
Supplement: S1 Dataset — (ZIP) [file pone.0158526.s009.zip › TVIBL07.rtf]

TVIBL07: Number (%) of Subjects with Baseline Q80K Polymorphism; Intent-to-treat
Treatment Group = Simeprevir 12Wks 150 mg PR12/24
i) Overall	
	Genotype 1	
	12 Weeks 
Treatment	>12 Weeks 
Treatment	All Subjects	
Analysis set: intent-to-treat	123	40	163	
	
All HCV geno/subtypes	123	40	163	
Subjects with sequencing data	119	40	159	
Q80K	6 
( 5.0%)	5 
( 12.5%)	11 
( 6.9%)	
No Q80K	113 
( 95.0%)	35 
( 87.5%)	148 
( 93.1%)	
	
HCV geno/subtype 1a/other	49	18	67	
Subjects with sequencing data	47	18	65	
Q80K	6 
( 12.8%)	5 
( 27.8%)	11 
( 16.9%)	
No Q80K	41 
( 87.2%)	13 
( 72.2%)	54 
( 83.1%)	
	
HCV geno/subtype 1b	74	22	96	
Subjects with sequencing data	72	22	94	
Q80K				
No Q80K	72 
( 100.0%)	22 
( 100.0%)	94 
( 100.0%)	
	
HCV geno/subtype 4a				
Subjects with sequencing data				
Q80K				
No Q80K				
	
HCV geno/subtype 4d				
Subjects with sequencing data				
Q80K				
No Q80K				
	
HCV geno/subtype 4other				
	

Polymorphisms are defined as changes from con1 (AJ238799) and H77 (AF009606) for HCV geno/subtype 1b and 1a/other, respectively	
[TVIBL07.rtf] [\STAT\Analyses\Programs\FinalAnalysis\Final1\2.TLF\6.Virology\VIR_FA.sas] 23OCT2015, 17:02	

TVIBL07: Number (%) of Subjects with Baseline Q80K Polymorphism; Intent-to-treat
Treatment Group = Simeprevir 12Wks 150 mg PR12/24
ii) By Country	
	Genotype 1	
	12 Weeks 
Treatment	>12 Weeks 
Treatment	All Subjects	
Analysis set: intent-to-treat	123	40	163	
	
Austria				
All HCV geno/subtypes	17	5	22	
Subjects with sequencing data	17	5	22	
Q80K	2 
( 11.8%)		2 
( 9.1%)	
No Q80K	15 
( 88.2%)	5 
( 100.0%)	20 
( 90.9%)	
HCV geno/subtype 1a/other	13	3	16	
Subjects with sequencing data	13	3	16	
Q80K	2 
( 15.4%)		2 
( 12.5%)	
No Q80K	11 
( 84.6%)	3 
( 100.0%)	14 
( 87.5%)	
HCV geno/subtype 1b	4	2	6	
Subjects with sequencing data	4	2	6	
Q80K				
No Q80K	4 
( 100.0%)	2 
( 100.0%)	6 
( 100.0%)	
HCV geno/subtype 4a				
Subjects with sequencing data				
Q80K				
No Q80K				
HCV geno/subtype 4d				
Subjects with sequencing data				
Q80K				
No Q80K				
HCV geno/subtype 4other				
	
Belgium				
All HCV geno/subtypes	14	2	16	
Subjects with sequencing data	13	2	15	
Q80K	1 
( 7.7%)	1 
( 50.0%)	2 
( 13.3%)	
No Q80K	12 
( 92.3%)	1 
( 50.0%)	13 
( 86.7%)	
HCV geno/subtype 1a/other	5	1	6	
Subjects with sequencing data	4	1	5	
Q80K	1 
( 25.0%)	1 
( 100.0%)	2 
( 40.0%)	
No Q80K	3 
( 75.0%)		3 
( 60.0%)	
HCV geno/subtype 1b	9	1	10	
Subjects with sequencing data	9	1	10	
Q80K				
No Q80K	9 
( 100.0%)	1 
( 100.0%)	10 
( 100.0%)	
HCV geno/subtype 4a				
Subjects with sequencing data				
Q80K				
No Q80K				
HCV geno/subtype 4other				
	
France				
All HCV geno/subtypes	29	8	37	
Subjects with sequencing data	27	8	35	
Q80K		2 
( 25.0%)	2 
( 5.7%)	
No Q80K	27 
( 100.0%)	6 
( 75.0%)	33 
( 94.3%)	
HCV geno/subtype 1a/other	14	5	19	
Subjects with sequencing data	13	5	18	
Q80K		2 
( 40.0%)	2 
( 11.1%)	
No Q80K	13 
( 100.0%)	3 
( 60.0%)	16 
( 88.9%)	
HCV geno/subtype 1b	15	3	18	
Subjects with sequencing data	14	3	17	
Q80K				
No Q80K	14 
( 100.0%)	3 
( 100.0%)	17 
( 100.0%)	
HCV geno/subtype 4a				
Subjects with sequencing data				
Q80K				
No Q80K				
HCV geno/subtype 4d				
Subjects with sequencing data				
Q80K				
No Q80K				
HCV geno/subtype 4other				
	
Germany				
All HCV geno/subtypes	23	5	28	
Subjects with sequencing data	22	5	27	
Q80K	1 
( 4.5%)		1 
( 3.7%)	
No Q80K	21 
( 95.5%)	5 
( 100.0%)	26 
( 96.3%)	
HCV geno/subtype 1a/other	7	3	10	
Subjects with sequencing data	7	3	10	
Q80K	1 
( 14.3%)		1 
( 10.0%)	
No Q80K	6 
( 85.7%)	3 
( 100.0%)	9 
( 90.0%)	
HCV geno/subtype 1b	16	2	18	
Subjects with sequencing data	15	2	17	
Q80K				
No Q80K	15 
( 100.0%)	2 
( 100.0%)	17 
( 100.0%)	
	
Italy				
All HCV geno/subtypes	13	6	19	
Subjects with sequencing data	13	6	19	
Q80K	1 
( 7.7%)		1 
( 5.3%)	
No Q80K	12 
( 92.3%)	6 
( 100.0%)	18 
( 94.7%)	
HCV geno/subtype 1a/other	2	1	3	
Subjects with sequencing data	2	1	3	
Q80K	1 
( 50.0%)		1 
( 33.3%)	
No Q80K	1 
( 50.0%)	1 
( 100.0%)	2 
( 66.7%)	
HCV geno/subtype 1b	11	5	16	
Subjects with sequencing data	11	5	16	
Q80K				
No Q80K	11 
( 100.0%)	5 
( 100.0%)	16 
( 100.0%)	
HCV geno/subtype 4a				
Subjects with sequencing data				
Q80K				
No Q80K				
HCV geno/subtype 4d				
Subjects with sequencing data				
Q80K				
No Q80K				
HCV geno/subtype 4other				
	
Saudi Arabia				
All HCV geno/subtypes				
Subjects with sequencing data				
Q80K				
No Q80K				
HCV geno/subtype 4a				
Subjects with sequencing data				
Q80K				
No Q80K				
HCV geno/subtype 4d				
Subjects with sequencing data				
Q80K				
No Q80K				
HCV geno/subtype 4other				
	
Spain				
All HCV geno/subtypes	18	12	30	
Subjects with sequencing data	18	12	30	
Q80K		1 
( 8.3%)	1 
( 3.3%)	
No Q80K	18 
( 100.0%)	11 
( 91.7%)	29 
( 96.7%)	
HCV geno/subtype 1a/other	4	4	8	
Subjects with sequencing data	4	4	8	
Q80K		1 
( 25.0%)	1 
( 12.5%)	
No Q80K	4 
( 100.0%)	3 
( 75.0%)	7 
( 87.5%)	
HCV geno/subtype 1b	14	8	22	
Subjects with sequencing data	14	8	22	
Q80K				
No Q80K	14 
( 100.0%)	8 
( 100.0%)	22 
( 100.0%)	
HCV geno/subtype 4a				
Subjects with sequencing data				
Q80K				
No Q80K				
HCV geno/subtype 4d				
Subjects with sequencing data				
Q80K				
No Q80K				
HCV geno/subtype 4other				
	
United Kingdom				
All HCV geno/subtypes	9	2	11	
Subjects with sequencing data	9	2	11	
Q80K	1 
( 11.1%)	1 
( 50.0%)	2 
( 18.2%)	
No Q80K	8 
( 88.9%)	1 
( 50.0%)	9 
( 81.8%)	
HCV geno/subtype 1a/other	4	1	5	
Subjects with sequencing data	4	1	5	
Q80K	1 
( 25.0%)	1 
( 100.0%)	2 
( 40.0%)	
No Q80K	3 
( 75.0%)		3 
( 60.0%)	
HCV geno/subtype 1b	5	1	6	
Subjects with sequencing data	5	1	6	
Q80K				
No Q80K	5 
( 100.0%)	1 
( 100.0%)	6 
( 100.0%)	
	

Polymorphisms are defined as changes from con1 (AJ238799) and H77 (AF009606) for HCV geno/subtype 1b and 1a/other, respectively	
[TVIBL07.rtf] [\STAT\Analyses\Programs\FinalAnalysis\Final1\2.TLF\6.Virology\VIR_FA.sas] 23OCT2015, 17:02	
