# Supplementary material for: An Open-Label Trial of 12-Week Simeprevir plus Peginterferon/Ribavirin (PR) in Treatment-Naïve Patients with Hepatitis C Virus (HCV) Genotype 1 (GT1)
Source: PLoS One. 2016 Jul 18;11(7):e0158526. doi: 10.1371/journal.pone.0158526 (PMC4948848; doi:10.1371/journal.pone.0158526)
Supplement: S1 Dataset — (ZIP) [file pone.0158526.s009.zip › TVIBL09.rtf]

TVIBL09: Number (%) of Subjects by Baseline Polymorphism at NS3 Positions 43, 80, 122, 155, 156 and 168; Intent-to-treat
HCV Geno/Subtype =  Total	
	Genotype 1	
n/N (%)	12 Weeks 
Treatment	>12 Weeks 
Treatment	All Subjects	
Analysis set: intent-to-treat	123	40	163	
	
HCV geno/subtype Total	123	40	163	
Subjects with sequencing data	119	40	159	
	
No Polymorphism at positions of interest #	93/119 (78.2%)	29/40 (72.5%)	122/159 (76.7%)	
Any Polymorphism at positions of interest #	26/119 (21.8%)	11/40 (27.5%)	37/159 (23.3%)	
T122N				
T122S				
S122G	11/119 (9.2%)	3/40 (7.5%)	14/159 (8.8%)	
Q80K	5/119 (4.2%)	5/40 (12.5%)	10/159 (6.3%)	
D168E	3/119 (2.5%)	1/40 (2.5%)	4/159 (2.5%)	
S122T	2/119 (1.7%)	1/40 (2.5%)	3/159 (1.9%)	
Q80L	2/119 (1.7%)		2/159 (1.3%)	
S122N	2/119 (1.7%)		2/159 (1.3%)	
Q80H		1/40 (2.5%)	1/159 (0.6%)	
Q80K+S122G	1/119 (0.8%)		1/159 (0.6%)	
	

# Any baseline polymorphism on NS3 postions 43, 80, 122, 155, 156, 168 are considered.
n = Number of subjects with polymorphism = numerator; N = Total number of subjects = denominator
Polymorphisms are defined as changes from con1 (AJ238799) and H77 (AF009606) for HCV geno/subtype 1b and 1a/other, 
	respectively	
[TVIBL09.rtf] [\STAT\Analyses\Programs\FinalAnalysis\Final1\2.TLF\6.Virology\VIR_FA.sas] 23OCT2015, 17:02	

TVIBL09: Number (%) of Subjects by Baseline Polymorphism at NS3 Positions 43, 80, 122, 155, 156 and 168; Intent-to-treat
HCV Geno/Subtype = 1a/other	
	Genotype 1	
n/N (%)	12 Weeks 
Treatment	>12 Weeks 
Treatment	All Subjects	
Analysis set: intent-to-treat	49	18	67	
	
HCV geno/subtype 1a/other	49	18	67	
Subjects with sequencing data	47	18	65	
	
No Polymorphism at positions of interest #	33/47 (70.2%)	9/18 (50.0%)	42/65 (64.6%)	
Any Polymorphism at positions of interest #	14/47 (29.8%)	9/18 (50.0%)	23/65 (35.4%)	
Q80K	5/47 (10.6%)	5/18 (27.8%)	10/65 (15.4%)	
S122G	7/47 (14.9%)	3/18 (16.7%)	10/65 (15.4%)	
D168E	1/47 (2.1%)	1/18 (5.6%)	2/65 (3.1%)	
Q80K+S122G	1/47 (2.1%)		1/65 (1.5%)	
	

# Any baseline polymorphism on NS3 postions 43, 80, 122, 155, 156, 168 are considered.
n = Number of subjects with polymorphism = numerator; N = Total number of subjects = denominator
Polymorphisms are defined as changes from con1 (AJ238799) and H77 (AF009606) for HCV geno/subtype 1b and 1a/other, 
	respectively	
[TVIBL09.rtf] [\STAT\Analyses\Programs\FinalAnalysis\Final1\2.TLF\6.Virology\VIR_FA.sas] 23OCT2015, 17:02	

TVIBL09: Number (%) of Subjects by Baseline Polymorphism at NS3 Positions 43, 80, 122, 155, 156 and 168; Intent-to-treat
HCV Geno/Subtype = 1b	
	Genotype 1	
n/N (%)	12 Weeks 
Treatment	>12 Weeks 
Treatment	All Subjects	
Analysis set: intent-to-treat	74	22	96	
	
HCV geno/subtype 1b	74	22	96	
Subjects with sequencing data	72	22	94	
	
No Polymorphism at positions of interest #	60/72 (83.3%)	20/22 (90.9%)	80/94 (85.1%)	
Any Polymorphism at positions of interest #	12/72 (16.7%)	2/22 (9.1%)	14/94 (14.9%)	
S122G	4/72 (5.6%)		4/94 (4.3%)	
S122T	2/72 (2.8%)	1/22 (4.5%)	3/94 (3.2%)	
D168E	2/72 (2.8%)		2/94 (2.1%)	
Q80L	2/72 (2.8%)		2/94 (2.1%)	
S122N	2/72 (2.8%)		2/94 (2.1%)	
Q80H		1/22 (4.5%)	1/94 (1.1%)	
	

# Any baseline polymorphism on NS3 postions 43, 80, 122, 155, 156, 168 are considered.
n = Number of subjects with polymorphism = numerator; N = Total number of subjects = denominator
Polymorphisms are defined as changes from con1 (AJ238799) and H77 (AF009606) for HCV geno/subtype 1b and 1a/other, 
	respectively	
[TVIBL09.rtf] [\STAT\Analyses\Programs\FinalAnalysis\Final1\2.TLF\6.Virology\VIR_FA.sas] 23OCT2015, 17:02	
