# Supplementary material for: An Open-Label Trial of 12-Week Simeprevir plus Peginterferon/Ribavirin (PR) in Treatment-Naïve Patients with Hepatitis C Virus (HCV) Genotype 1 (GT1)
Source: PLoS One. 2016 Jul 18;11(7):e0158526. doi: 10.1371/journal.pone.0158526 (PMC4948848; doi:10.1371/journal.pone.0158526)
Supplement: S1 Dataset — (ZIP) [file pone.0158526.s009.zip › TVIFAI05A.RTF]

TVIFAI05A: Number (%) of Subjects with Emerging Mutations (NS3 Positions 36,41,43,54,55,80,107,122,132,138,155,156,158,168,169,170,174,175) in Subjects with Failure - at Time of Failure - by Baseline Q80K Polymorphism; Intent-to-treat
   
  
 Treatment Group = Simeprevir 12Wks 150 mg PR12/24 
Genotype 1	
	12 Weeks 
Treatment	>12 Weeks 
Treatment	All Subjects	
	HCV Geno/Subtype	HCV Geno/Subtype	HCV Geno/Subtype	
n/N (%)	1a/other	1b	Total	1a/other	1b	Total	1a/other	1b	Total	
Analysis set: intent-to treat	49	74	123	18	22	40	67	96	163	
	
Failure	19	26	45	8	11	19	27	37	64	
	
Sequencing data										
Available #	11	15	26	3	3	6	14	18	32	
Not Available	8	11	19	5	8	13	13	19	32	
	
No Emerging Mutation	1/11 
( 9.1%)		1/26 
( 3.8%)				1/14 
( 7.1%)		1/32 
( 3.1%)	
Without Q80K at baseline	1/11 
( 9.1%)		1/26 
( 3.8%)				1/14 
( 7.1%)		1/32 
( 3.1%)	
	
Any Emerging Mutation	10/11 
( 90.9%)	15/15 
( 100.0%)	25/26 
( 96.2%)	3/3 
( 100.0%)	3/3 
( 100.0%)	6/6 
( 100.0%)	13/14 
( 92.9%)	18/18 
( 100.0%)	31/32 
( 96.9%)	
Without Q80K at baseline	10/11 
( 90.9%)	15/15 
( 100.0%)	25/26 
( 96.2%)	2/3 
( 66.7%)	3/3 
( 100.0%)	5/6 
( 83.3%)	12/14 
( 85.7%)	18/18 
( 100.0%)	30/32 
( 93.8%)	
D168V	2/11 
( 18.2%)	8/15 
( 53.3%)	10/26 
( 38.5%)	1/3 
( 33.3%)		1/6 
( 16.7%)	3/14 
( 21.4%)	8/18 
( 44.4%)	11/32 
( 34.4%)	
D168A	2/11 
( 18.2%)	1/15 
( 6.7%)	3/26 
( 11.5%)				2/14 
( 14.3%)	1/18 
( 5.6%)	3/32 
( 9.4%)	
R155K	3/11 
( 27.3%)		3/26 
( 11.5%)				3/14 
( 21.4%)		3/32 
( 9.4%)	
Q80R+D168E/V		1/15 
( 6.7%)	1/26 
( 3.8%)		1/3 
( 33.3%)	1/6 
( 16.7%)		2/18 
( 11.1%)	2/32 
( 6.3%)	
R155Q+D168V		2/15 
( 13.3%)	2/26 
( 7.7%)					2/18 
( 11.1%)	2/32 
( 6.3%)	
D168A+V170I		1/15 
( 6.7%)	1/26 
( 3.8%)					1/18 
( 5.6%)	1/32 
( 3.1%)	
D168E/V					1/3 
( 33.3%)	1/6 
( 16.7%)		1/18 
( 5.6%)	1/32 
( 3.1%)	
D168H		1/15 
( 6.7%)	1/26 
( 3.8%)					1/18 
( 5.6%)	1/32 
( 3.1%)	
Q80K+R155K+D168A	1/11 
( 9.1%)		1/26 
( 3.8%)				1/14 
( 7.1%)		1/32 
( 3.1%)	
Q80L+R155K	1/11 
( 9.1%)		1/26 
( 3.8%)				1/14 
( 7.1%)		1/32 
( 3.1%)	
Q80R+D168E					1/3 
( 33.3%)	1/6 
( 16.7%)		1/18 
( 5.6%)	1/32 
( 3.1%)	
R155K+D168E+L175F				1/3 
( 33.3%)		1/6 
( 16.7%)	1/14 
( 7.1%)		1/32 
( 3.1%)	
S122T		1/15 
( 6.7%)	1/26 
( 3.8%)					1/18 
( 5.6%)	1/32 
( 3.1%)	
T54S+R155K	1/11 
( 9.1%)		1/26 
( 3.8%)				1/14 
( 7.1%)		1/32 
( 3.1%)	
With Q80K at baseline				1/3 
( 33.3%)		1/6 
( 16.7%)	1/14 
( 7.1%)		1/32 
( 3.1%)	
R155K				1/3 
( 33.3%)		1/6 
( 16.7%)	1/14 
( 7.1%)		1/32 
( 3.1%)	
	

n = number of subjects with mutation = numerator; N = total number of subjects with sequencing data at baseline and at the particular time point = denominator
#: Only subjects with baseline and post-baseline sequencing data are considered.
Polymorphisms are defined as changes from con1 (AJ238799) and H77 (AF009606) for hcv geno/subtype 1b and 1a/other,respectively.
Emerging mutations profiles containing mutations at position 132, 170, 174 and 175 will be shown on separate lines  for HCV geno/subtype 1a and HCV geno/subtype
1b due to different wildtype amino acids (i.e. I132 and V132, I170 and V170, N174 and S174, L175 and M175 respectively.)
Failures: all subjects not achieving SVR12 or who relapsed after timepoint of SVR12.	
[TVIFAI05A.rtf] [\STAT\Analyses\Programs\FinalAnalysis\Final1\2.TLF\6.Virology\VIR_FA.sas] 23OCT2015, 17:02	
