# Supplementary material for: An Open-Label Trial of 12-Week Simeprevir plus Peginterferon/Ribavirin (PR) in Treatment-Naïve Patients with Hepatitis C Virus (HCV) Genotype 1 (GT1)
Source: PLoS One. 2016 Jul 18;11(7):e0158526. doi: 10.1371/journal.pone.0158526 (PMC4948848; doi:10.1371/journal.pone.0158526)
Supplement: S1 Dataset — (ZIP) [file pone.0158526.s009.zip › Patient-reported Outcomes/TPROCED03.rtf]

TPROCED03:	Descriptive Statistics of the Changes from Baseline in the CES-D Depression Score per Analysis Timepoint - Available Data Approach(;) Intent-to-Treat (Study TMC435HPC3014)
Treatment Group = Simeprevir 12Wks 150 mg PR12/24 
Phase = Overall Study Period 
1) Overall	
	12 Weeks 
Treatment	>12 Weeks 
Treatment	All Subjects		
Week 4					
N	117	38	155		
Mean	5.2	4.4	5.0		
SE	0.87	1.31	0.73		
SD	9.40	8.06	9.07		
95% C.I. *	(3.44; 6.88)	(1.72; 7.02)	(3.53; 6.41)		
Min	-9	-13	-13		
Q1	-1.0	0.0	-1.0		
Median	3.0	4.5	3.0		
Q3	9.0	9.0	9.0		
Max	34	23	34		
	
Week 8					
N	116	34	150		
Mean	4.2	4.3	4.2		
SE	0.81	1.76	0.74		
SD	8.72	10.25	9.05		
95% C.I. *	(2.57; 5.78)	(0.68; 7.84)	(2.73; 5.65)		
Min	-14	-20	-20		
Q1	-1.4	-1.0	-1.0		
Median	3.5	3.5	3.5		
Q3	8.3	8.1	8.2		
Max	30	39	39		
	
Week 12					
N	120	30	150		
Mean	6.1	5.5	6.0		
SE	0.95	2.03	0.86		
SD	10.41	11.13	10.53		
95% C.I. *	(4.17; 7.94)	(1.39; 9.70)	(4.26; 7.65)		
Min	-13	-12	-13		
Q1	0.0	0.0	0.0		
Median	4.0	3.5	4.0		
Q3	13.5	8.0	13.0		
Max	40	40	40		
	
Week 16					
N	115	33	148		
Mean	-0.1	4.1	0.8		
SE	0.76	1.58	0.70		
SD	8.10	9.06	8.48		
95% C.I. *	(-1.64; 1.35)	(0.88; 7.31)	(-0.58; 2.18)		
Min	-19	-12	-19		
Q1	-5.0	0.0	-5.0		
Median	-1.0	3.0	0.0		
Q3	4.0	7.0	5.0		
Max	33	29	33		
	
Week 20					
N		28	28		
Mean		3.6	3.6		
SE		1.64	1.64		
SD		8.70	8.70		
95% C.I. *		(0.23; 6.98)	(0.23; 6.98)		
Min		-12	-12		
Q1		-2.5	-2.5		
Median		4.9	4.9		
Q3		8.5	8.5		
Max		21	21		
	
Week 24					
N	110	29	139		
Mean	-1.7	2.7	-0.8		
SE	0.72	1.50	0.66		
SD	7.50	8.08	7.81		
95% C.I. *	(-3.16; -0.33)	(-0.33; 5.81)	(-2.12; 0.50)		
Min	-26	-14	-26		
Q1	-6.0	-2.0	-5.0		
Median	-1.6	3.8	-1.0		
Q3	2.0	9.0	3.0		
Max	19	14	19		
	
Week 36					
N	8		8		
Mean	-4.5		-4.5		
SE	2.16		2.16		
SD	6.12		6.12		
95% C.I. *	(-9.61; 0.61)		(-9.61; 0.61)		
Min	-19		-19		
Q1	-4.5		-4.5		
Median	-3.0		-3.0		
Q3	-1.0		-1.0		
Max	0		0		
	

* Confidence interval for mean
Subjects with planned end of treatment at Week 12 do not have EQ-5Q, CES-D, FSS or WPAI results at Week 20.
The CES-D score ranges from 0 to 60, with higher scores indicating worse outcome.	
[TPROCED03.rtf] [\STAT\Analyses\Programs\Primary Analysis\Final4\2.TLF\7.PRO_PA\PRO_PA.sas] 15JAN2015, 16:51	

TPROCED03:	Descriptive Statistics of the Changes from Baseline in the CES-D Depression Score per Analysis Timepoint - Available Data Approach(;) Intent-to-Treat (Study TMC435HPC3014)
Treatment Group = Simeprevir 12Wks 150 mg PR12/24 
Phase = Overall Study Period 
2) By SVR12	
	SVR12 No	SVR12 Yes		
	12 Weeks 
Treatment	All Subjects	12 Weeks 
Treatment	All Subjects		
Week 4						
N	43	43	74	74		
Mean	3.5	3.5	6.2	6.2		
SE	1.25	1.25	1.16	1.16		
SD	8.19	8.19	9.96	9.96		
95% C.I. *	(0.94; 5.98)	(0.94; 5.98)	(3.85; 8.46)	(3.85; 8.46)		
Min	-9	-9	-9	-9		
Q1	-2.0	-2.0	-1.0	-1.0		
Median	3.0	3.0	3.5	3.5		
Q3	7.0	7.0	9.2	9.2		
Max	28	28	34	34		
	
Week 8						
N	43	43	73	73		
Mean	3.9	3.9	4.4	4.4		
SE	1.45	1.45	0.97	0.97		
SD	9.52	9.52	8.27	8.27		
95% C.I. *	(0.94; 6.80)	(0.94; 6.80)	(2.42; 6.28)	(2.42; 6.28)		
Min	-13	-13	-14	-14		
Q1	-2.0	-2.0	-1.0	-1.0		
Median	4.0	4.0	3.0	3.0		
Q3	8.0	8.0	8.4	8.4		
Max	27	27	30	30		
	
Week 12						
N	43	43	77	77		
Mean	6.2	6.2	6.0	6.0		
SE	1.57	1.57	1.20	1.20		
SD	10.32	10.32	10.53	10.53		
95% C.I. *	(2.98; 9.33)	(2.98; 9.33)	(3.61; 8.39)	(3.61; 8.39)		
Min	-10	-10	-13	-13		
Q1	-2.0	-2.0	0.0	0.0		
Median	5.0	5.0	4.0	4.0		
Q3	14.0	14.0	12.2	12.2		
Max	28	28	40	40		
	
Week 16						
N	41	41	74	74		
Mean	1.1	1.1	-0.8	-0.8		
SE	1.54	1.54	0.80	0.80		
SD	9.89	9.89	6.90	6.90		
95% C.I. *	(-2.01; 4.23)	(-2.01; 4.23)	(-2.43; 0.76)	(-2.43; 0.76)		
Min	-15	-15	-19	-19		
Q1	-5.6	-5.6	-4.7	-4.7		
Median	-2.0	-2.0	-1.0	-1.0		
Q3	5.0	5.0	2.0	2.0		
Max	33	33	19	19		
	
Week 24						
N	37	37	73	73		
Mean	-0.7	-0.7	-2.3	-2.3		
SE	1.16	1.16	0.90	0.90		
SD	7.03	7.03	7.72	7.72		
95% C.I. *	(-3.06; 1.63)	(-3.06; 1.63)	(-4.07; -0.46)	(-4.07; -0.46)		
Min	-13	-13	-26	-26		
Q1	-5.0	-5.0	-6.0	-6.0		
Median	-2.0	-2.0	-1.0	-1.0		
Q3	2.1	2.1	2.0	2.0		
Max	19	19	18	18		
	
Week 36						
N	3	3	5	5		
Mean	-3.0	-3.0	-5.4	-5.4		
SE	0.58	0.58	3.53	3.53		
SD	1.00	1.00	7.89	7.89		
95% C.I. *	(-5.48; -0.52)	(-5.48; -0.52)	(-15.20; 4.40)	(-15.20; 4.40)		
Min	-4	-4	-19	-19		
Q1	-4.0	-4.0	-5.0	-5.0		
Median	-3.0	-3.0	-3.0	-3.0		
Q3	-2.0	-2.0	0.0	0.0		
Max	-2	-2	0	0		
	

* Confidence interval for mean
Subjects with planned end of treatment at Week 12 do not have EQ-5Q, CES-D, FSS or WPAI results at Week 20.
The CES-D score ranges from 0 to 60, with higher scores indicating worse outcome.	
[TPROCED03.rtf] [\STAT\Analyses\Programs\Primary Analysis\Final4\2.TLF\7.PRO_PA\PRO_PA.sas] 15JAN2015, 16:51	

TPROCED03:	Descriptive Statistics of the Changes from Baseline in the CES-D Depression Score per Analysis Timepoint - Available Data Approach(;) Intent-to-Treat (Study TMC435HPC3014)
Treatment Group = Simeprevir 12Wks 150 mg PR12/24 
Phase = Overall Study Period 
3) By Region	
	Europe		
	12 Weeks 
Treatment	>12 Weeks 
Treatment	All Subjects		
Week 4					
N	117	38	155		
Mean	5.2	4.4	5.0		
SE	0.87	1.31	0.73		
SD	9.40	8.06	9.07		
95% C.I. *	(3.44; 6.88)	(1.72; 7.02)	(3.53; 6.41)		
Min	-9	-13	-13		
Q1	-1.0	0.0	-1.0		
Median	3.0	4.5	3.0		
Q3	9.0	9.0	9.0		
Max	34	23	34		
	
Week 8					
N	116	34	150		
Mean	4.2	4.3	4.2		
SE	0.81	1.76	0.74		
SD	8.72	10.25	9.05		
95% C.I. *	(2.57; 5.78)	(0.68; 7.84)	(2.73; 5.65)		
Min	-14	-20	-20		
Q1	-1.4	-1.0	-1.0		
Median	3.5	3.5	3.5		
Q3	8.3	8.1	8.2		
Max	30	39	39		
	
Week 12					
N	120	30	150		
Mean	6.1	5.5	6.0		
SE	0.95	2.03	0.86		
SD	10.41	11.13	10.53		
95% C.I. *	(4.17; 7.94)	(1.39; 9.70)	(4.26; 7.65)		
Min	-13	-12	-13		
Q1	0.0	0.0	0.0		
Median	4.0	3.5	4.0		
Q3	13.5	8.0	13.0		
Max	40	40	40		
	
Week 16					
N	115	33	148		
Mean	-0.1	4.1	0.8		
SE	0.76	1.58	0.70		
SD	8.10	9.06	8.48		
95% C.I. *	(-1.64; 1.35)	(0.88; 7.31)	(-0.58; 2.18)		
Min	-19	-12	-19		
Q1	-5.0	0.0	-5.0		
Median	-1.0	3.0	0.0		
Q3	4.0	7.0	5.0		
Max	33	29	33		
	
Week 20					
N		28	28		
Mean		3.6	3.6		
SE		1.64	1.64		
SD		8.70	8.70		
95% C.I. *		(0.23; 6.98)	(0.23; 6.98)		
Min		-12	-12		
Q1		-2.5	-2.5		
Median		4.9	4.9		
Q3		8.5	8.5		
Max		21	21		
	
Week 24					
N	110	29	139		
Mean	-1.7	2.7	-0.8		
SE	0.72	1.50	0.66		
SD	7.50	8.08	7.81		
95% C.I. *	(-3.16; -0.33)	(-0.33; 5.81)	(-2.12; 0.50)		
Min	-26	-14	-26		
Q1	-6.0	-2.0	-5.0		
Median	-1.6	3.8	-1.0		
Q3	2.0	9.0	3.0		
Max	19	14	19		
	
Week 36					
N	8		8		
Mean	-4.5		-4.5		
SE	2.16		2.16		
SD	6.12		6.12		
95% C.I. *	(-9.61; 0.61)		(-9.61; 0.61)		
Min	-19		-19		
Q1	-4.5		-4.5		
Median	-3.0		-3.0		
Q3	-1.0		-1.0		
Max	0		0		
	

* Confidence interval for mean
Subjects with planned end of treatment at Week 12 do not have EQ-5Q, CES-D, FSS or WPAI results at Week 20.
The CES-D score ranges from 0 to 60, with higher scores indicating worse outcome.	
[TPROCED03.rtf] [\STAT\Analyses\Programs\Primary Analysis\Final4\2.TLF\7.PRO_PA\PRO_PA.sas] 15JAN2015, 16:51	
