# Supplementary material for: An Open-Label Trial of 12-Week Simeprevir plus Peginterferon/Ribavirin (PR) in Treatment-Naïve Patients with Hepatitis C Virus (HCV) Genotype 1 (GT1)
Source: PLoS One. 2016 Jul 18;11(7):e0158526. doi: 10.1371/journal.pone.0158526 (PMC4948848; doi:10.1371/journal.pone.0158526)
Supplement: S1 Dataset — (ZIP) [file pone.0158526.s009.zip › Patient-reported Outcomes/TPROEQ04.rtf]

TPROEQ04:	Descriptive Statistics of the Changes in the EQ-5D Visual Analogue Scale (VAS) per Analysis Timepoint - Available Data Approach(;) Intent-to-Treat (Study TMC435HPC3014)
Treatment Group = Simeprevir 12Wks 150 mg PR12/24 
Phase = Overall Study Period 
1) Overall	
	12 Weeks 
Treatment	>12 Weeks 
Treatment	All Subjects		
Week 4					
N	101	33	134		
Mean	-9.6	-12.8	-10.4		
SE	2.16	3.40	1.83		
SD	21.71	19.51	21.17		
95% C.I. *	(-13.91; -5.34)	(-19.77; -5.93)	(-14.03; -6.80)		
Min	-95	-75	-95		
Q1	-20.0	-20.0	-20.0		
Median	-5.0	-10.0	-5.0		
Q3	0.0	0.0	0.0		
Max	72	20	72		
	
Week 8					
N	99	31	130		
Mean	-9.6	-8.8	-9.4		
SE	2.05	3.54	1.77		
SD	20.44	19.73	20.20		
95% C.I. *	(-13.65; -5.50)	(-16.08; -1.60)	(-12.91; -5.89)		
Min	-60	-75	-75		
Q1	-20.0	-15.0	-20.0		
Median	-10.0	0.0	-8.5		
Q3	0.0	5.0	0.0		
Max	67	20	67		
	
Week 12					
N	107	28	135		
Mean	-7.3	-13.8	-8.6		
SE	2.06	3.30	1.78		
SD	21.34	17.48	20.71		
95% C.I. *	(-11.37; -3.19)	(-20.56; -7.01)	(-12.15; -5.10)		
Min	-80	-45	-80		
Q1	-20.0	-25.0	-20.0		
Median	-5.0	-10.0	-5.0		
Q3	0.0	1.5	0.0		
Max	65	10	65		
	
Week 16					
N	107	29	136		
Mean	1.1	-11.7	-1.6		
SE	1.71	3.48	1.60		
SD	17.70	18.75	18.61		
95% C.I. *	(-2.30; 4.49)	(-18.82; -4.56)	(-4.79; 1.52)		
Min	-45	-75	-75		
Q1	-9.0	-20.0	-10.0		
Median	0.0	-10.0	0.0		
Q3	5.0	0.0	5.0		
Max	70	10	70		
	
Week 20					
N		27	27		
Mean		-13.5	-13.5		
SE		3.85	3.85		
SD		20.00	20.00		
95% C.I. *		(-21.39; -5.57)	(-21.39; -5.57)		
Min		-75	-75		
Q1		-25.0	-25.0		
Median		-10.0	-10.0		
Q3		0.0	0.0		
Max		15	15		
	
Week 24					
N	105	27	132		
Mean	2.2	-11.1	-0.6		
SE	1.74	3.64	1.63		
SD	17.85	18.90	18.78		
95% C.I. *	(-1.29; 5.62)	(-18.59; -3.64)	(-3.79; 2.68)		
Min	-70	-65	-70		
Q1	-5.0	-25.0	-10.0		
Median	0.0	-5.0	0.0		
Q3	5.0	2.0	5.0		
Max	70	15	70		
	
Week 36					
N	8		8		
Mean	7.5		7.5		
SE	4.76		4.76		
SD	13.46		13.46		
95% C.I. *	(-3.75; 18.75)		(-3.75; 18.75)		
Min	-3		-3		
Q1	1.5		1.5		
Median	5.0		5.0		
Q3	5.0		5.0		
Max	40		40		
	

* Confidence interval for mean
Subjects with planned end of treatment at Week 12 do not have EQ-5Q, CES-D, FSS or WPAI results at Week 20.
Result of the EQ-5D Visual Analogue Scale (VAS) ranges from a 0 to 100 score, indicating the worst and best imaginable health states, respectively.	
[TPROEQ04.rtf] [\STAT\Analyses\Programs\Primary Analysis\Final4\2.TLF\7.PRO_PA\PRO_PA.sas] 15JAN2015, 16:51	

TPROEQ04:	Descriptive Statistics of the Changes in the EQ-5D Visual Analogue Scale (VAS) per Analysis Timepoint - Available Data Approach(;) Intent-to-Treat (Study TMC435HPC3014)
Treatment Group = Simeprevir 12Wks 150 mg PR12/24 
Phase = Overall Study Period 
2) By SVR12	
	SVR12 No	SVR12 Yes		
	12 Weeks 
Treatment	All Subjects	12 Weeks 
Treatment	All Subjects		
Week 4						
N	34	34	67	67		
Mean	-6.4	-6.4	-11.3	-11.3		
SE	2.32	2.32	3.03	3.03		
SD	13.53	13.53	24.79	24.79		
95% C.I. *	(-11.10; -1.66)	(-11.10; -1.66)	(-17.32; -5.22)	(-17.32; -5.22)		
Min	-35	-35	-95	-95		
Q1	-15.0	-15.0	-25.0	-25.0		
Median	-5.0	-5.0	-5.0	-5.0		
Q3	0.0	0.0	0.0	0.0		
Max	25	25	72	72		
	
Week 8						
N	36	36	63	63		
Mean	-10.6	-10.6	-9.0	-9.0		
SE	2.46	2.46	2.92	2.92		
SD	14.74	14.74	23.17	23.17		
95% C.I. *	(-15.60; -5.63)	(-15.60; -5.63)	(-14.82; -3.15)	(-14.82; -3.15)		
Min	-40	-40	-60	-60		
Q1	-20.0	-20.0	-20.0	-20.0		
Median	-10.0	-10.0	-5.0	-5.0		
Q3	-1.0	-1.0	0.0	0.0		
Max	25	25	67	67		
	
Week 12						
N	36	36	71	71		
Mean	-7.6	-7.6	-7.1	-7.1		
SE	2.78	2.78	2.78	2.78		
SD	16.69	16.69	23.46	23.46		
95% C.I. *	(-13.20; -1.91)	(-13.20; -1.91)	(-12.69; -1.59)	(-12.69; -1.59)		
Min	-40	-40	-80	-80		
Q1	-20.0	-20.0	-15.0	-15.0		
Median	-7.5	-7.5	-5.0	-5.0		
Q3	5.0	5.0	0.0	0.0		
Max	30	30	65	65		
	
Week 16						
N	37	37	70	70		
Mean	1.8	1.8	0.7	0.7		
SE	2.30	2.30	2.33	2.33		
SD	13.98	13.98	19.46	19.46		
95% C.I. *	(-2.85; 6.47)	(-2.85; 6.47)	(-3.93; 5.36)	(-3.93; 5.36)		
Min	-35	-35	-45	-45		
Q1	-10.0	-10.0	-5.0	-5.0		
Median	5.0	5.0	0.0	0.0		
Q3	8.0	8.0	5.0	5.0		
Max	30	30	70	70		
	
Week 24						
N	34	34	71	71		
Mean	-0.6	-0.6	3.5	3.5		
SE	1.99	1.99	2.39	2.39		
SD	11.60	11.60	20.11	20.11		
95% C.I. *	(-4.63; 3.46)	(-4.63; 3.46)	(-1.28; 8.24)	(-1.28; 8.24)		
Min	-30	-30	-70	-70		
Q1	-5.0	-5.0	-5.0	-5.0		
Median	0.0	0.0	0.0	0.0		
Q3	5.0	5.0	10.0	10.0		
Max	35	35	70	70		
	
Week 36						
N	3	3	5	5		
Mean	4.3	4.3	9.4	9.4		
SE	0.67	0.67	7.80	7.80		
SD	1.15	1.15	17.44	17.44		
95% C.I. *	(1.46; 7.20)	(1.46; 7.20)	(-12.26; 31.06)	(-12.26; 31.06)		
Min	3	3	-3	-3		
Q1	3.0	3.0	0.0	0.0		
Median	5.0	5.0	5.0	5.0		
Q3	5.0	5.0	5.0	5.0		
Max	5	5	40	40		
	

* Confidence interval for mean
Subjects with planned end of treatment at Week 12 do not have EQ-5Q, CES-D, FSS or WPAI results at Week 20.
Result of the EQ-5D Visual Analogue Scale (VAS) ranges from a 0 to 100 score, indicating the worst and best imaginable health states, respectively.	
[TPROEQ04.rtf] [\STAT\Analyses\Programs\Primary Analysis\Final4\2.TLF\7.PRO_PA\PRO_PA.sas] 15JAN2015, 16:51	

TPROEQ04:	Descriptive Statistics of the Changes in the EQ-5D Visual Analogue Scale (VAS) per Analysis Timepoint - Available Data Approach(;) Intent-to-Treat (Study TMC435HPC3014)
Treatment Group = Simeprevir 12Wks 150 mg PR12/24 
Phase = Overall Study Period 
3) By Region	
	Europe		
	12 Weeks 
Treatment	>12 Weeks 
Treatment	All Subjects		
Week 4					
N	101	33	134		
Mean	-9.6	-12.8	-10.4		
SE	2.16	3.40	1.83		
SD	21.71	19.51	21.17		
95% C.I. *	(-13.91; -5.34)	(-19.77; -5.93)	(-14.03; -6.80)		
Min	-95	-75	-95		
Q1	-20.0	-20.0	-20.0		
Median	-5.0	-10.0	-5.0		
Q3	0.0	0.0	0.0		
Max	72	20	72		
	
Week 8					
N	99	31	130		
Mean	-9.6	-8.8	-9.4		
SE	2.05	3.54	1.77		
SD	20.44	19.73	20.20		
95% C.I. *	(-13.65; -5.50)	(-16.08; -1.60)	(-12.91; -5.89)		
Min	-60	-75	-75		
Q1	-20.0	-15.0	-20.0		
Median	-10.0	0.0	-8.5		
Q3	0.0	5.0	0.0		
Max	67	20	67		
	
Week 12					
N	107	28	135		
Mean	-7.3	-13.8	-8.6		
SE	2.06	3.30	1.78		
SD	21.34	17.48	20.71		
95% C.I. *	(-11.37; -3.19)	(-20.56; -7.01)	(-12.15; -5.10)		
Min	-80	-45	-80		
Q1	-20.0	-25.0	-20.0		
Median	-5.0	-10.0	-5.0		
Q3	0.0	1.5	0.0		
Max	65	10	65		
	
Week 16					
N	107	29	136		
Mean	1.1	-11.7	-1.6		
SE	1.71	3.48	1.60		
SD	17.70	18.75	18.61		
95% C.I. *	(-2.30; 4.49)	(-18.82; -4.56)	(-4.79; 1.52)		
Min	-45	-75	-75		
Q1	-9.0	-20.0	-10.0		
Median	0.0	-10.0	0.0		
Q3	5.0	0.0	5.0		
Max	70	10	70		
	
Week 20					
N		27	27		
Mean		-13.5	-13.5		
SE		3.85	3.85		
SD		20.00	20.00		
95% C.I. *		(-21.39; -5.57)	(-21.39; -5.57)		
Min		-75	-75		
Q1		-25.0	-25.0		
Median		-10.0	-10.0		
Q3		0.0	0.0		
Max		15	15		
	
Week 24					
N	105	27	132		
Mean	2.2	-11.1	-0.6		
SE	1.74	3.64	1.63		
SD	17.85	18.90	18.78		
95% C.I. *	(-1.29; 5.62)	(-18.59; -3.64)	(-3.79; 2.68)		
Min	-70	-65	-70		
Q1	-5.0	-25.0	-10.0		
Median	0.0	-5.0	0.0		
Q3	5.0	2.0	5.0		
Max	70	15	70		
	
Week 36					
N	8		8		
Mean	7.5		7.5		
SE	4.76		4.76		
SD	13.46		13.46		
95% C.I. *	(-3.75; 18.75)		(-3.75; 18.75)		
Min	-3		-3		
Q1	1.5		1.5		
Median	5.0		5.0		
Q3	5.0		5.0		
Max	40		40		
	

* Confidence interval for mean
Subjects with planned end of treatment at Week 12 do not have EQ-5Q, CES-D, FSS or WPAI results at Week 20.
Result of the EQ-5D Visual Analogue Scale (VAS) ranges from a 0 to 100 score, indicating the worst and best imaginable health states, respectively.	
[TPROEQ04.rtf] [\STAT\Analyses\Programs\Primary Analysis\Final4\2.TLF\7.PRO_PA\PRO_PA.sas] 15JAN2015, 16:51	
