# Supplementary material for: An Open-Label Trial of 12-Week Simeprevir plus Peginterferon/Ribavirin (PR) in Treatment-Naïve Patients with Hepatitis C Virus (HCV) Genotype 1 (GT1)
Source: PLoS One. 2016 Jul 18;11(7):e0158526. doi: 10.1371/journal.pone.0158526 (PMC4948848; doi:10.1371/journal.pone.0158526)
Supplement: S1 Dataset — (ZIP) [file pone.0158526.s009.zip › Patient-reported Outcomes/TPROEQ10.rtf]

TPROEQ10:	Descriptive Statistics of the Changes in the EQ-5D Valuation Index per Analysis Timepoint; Intent-to-Treat (Study TMC435HPC3014)
Treatment Group = Simeprevir 12Wks 150 mg PR12/24 
Phase = Overall Study Period 
1) Overall	
	12 Weeks 
Treatment	>12 Weeks 
Treatment	All Subjects		
Week 4					
N	117	36	153		
Mean	-0.1154	-0.1325	-0.1194		
SE	0.01952	0.02770	0.01626		
SD	0.21118	0.16619	0.20112		
95% C.I. *	(-0.15406; -0.07672)	(-0.18873; -0.07627)	(-0.15154; -0.08729)		
Min	-0.719	-0.475	-0.719		
Q1	-0.2340	-0.2690	-0.2340		
Median	-0.0520	-0.0665	-0.0520		
Q3	0.0000	0.0000	0.0000		
Max	0.656	0.163	0.656		
	
Week 8					
N	112	35	147		
Mean	-0.1136	-0.1439	-0.1208		
SE	0.02023	0.03378	0.01736		
SD	0.21404	0.19984	0.21047		
95% C.I. *	(-0.15369; -0.07354)	(-0.21259; -0.07530)	(-0.15515; -0.08653)		
Min	-0.809	-0.923	-0.923		
Q1	-0.2310	-0.2330	-0.2320		
Median	-0.0985	-0.1110	-0.1010		
Q3	0.0000	0.0000	0.0000		
Max	0.656	0.163	0.656		
	
Week 12					
N	115	30	145		
Mean	-0.1255	-0.1793	-0.1366		
SE	0.02094	0.03117	0.01786		
SD	0.22453	0.17072	0.21508		
95% C.I. *	(-0.16698; -0.08403)	(-0.24308; -0.11559)	(-0.17195; -0.10134)		
Min	-0.949	-0.693	-0.949		
Q1	-0.2590	-0.2720	-0.2600		
Median	-0.0940	-0.1525	-0.1110		
Q3	0.0000	-0.0080	0.0000		
Max	0.698	0.059	0.698		
	
Week 16					
N	115	32	147		
Mean	-0.0039	-0.1310	-0.0316		
SE	0.01849	0.03423	0.01679		
SD	0.19834	0.19363	0.20357		
95% C.I. *	(-0.04059; 0.03269)	(-0.20078; -0.06116)	(-0.06478; 0.00159)		
Min	-0.559	-0.613	-0.613		
Q1	-0.0980	-0.1850	-0.1230		
Median	0.0000	-0.0725	0.0000		
Q3	0.0690	0.0000	0.0280		
Max	0.698	0.240	0.698		
	
Week 20					
N		28	28		
Mean		-0.1580	-0.1580		
SE		0.04157	0.04157		
SD		0.21995	0.21995		
95% C.I. *		(-0.24325; -0.07268)	(-0.24325; -0.07268)		
Min		-0.661	-0.661		
Q1		-0.2955	-0.2955		
Median		-0.0420	-0.0420		
Q3		0.0000	0.0000		
Max		0.121	0.121		
	
Week 24					
N	112	28	140		
Mean	0.0148	-0.1046	-0.0091		
SE	0.01540	0.02812	0.01410		
SD	0.16303	0.14881	0.16680		
95% C.I. *	(-0.01573; 0.04532)	(-0.16231; -0.04691)	(-0.03696; 0.01879)		
Min	-0.409	-0.467	-0.467		
Q1	-0.0060	-0.2245	-0.0715		
Median	0.0000	-0.0355	0.0000		
Q3	0.1025	0.0000	0.0540		
Max	0.819	0.121	0.819		
	
Week 36					
N	8		8		
Mean	0.0504		0.0504		
SE	0.04894		0.04894		
SD	0.13842		0.13842		
95% C.I. *	(-0.06534; 0.16609)		(-0.06534; 0.16609)		
Min	-0.114		-0.114		
Q1	0.0000		0.0000		
Median	0.0000		0.0000		
Q3	0.0820		0.0820		
Max	0.353		0.353		
	

* Confidence interval for mean
Subjects with planned end of treatment at Week 12 do not have EQ-5Q, CES-D, FSS or WPAI results at Week 20.
Result from the EQ-5D Valuation Index range from -0.594 to 1 with higher scores indicating better outcome.	
[TPROEQ10.rtf] [\STAT\Analyses\Programs\Primary Analysis\Final4\2.TLF\7.PRO_PA\PRO_PA.sas] 15JAN2015, 16:51	

TPROEQ10:	Descriptive Statistics of the Changes in the EQ-5D Valuation Index per Analysis Timepoint; Intent-to-Treat (Study TMC435HPC3014)
Treatment Group = Simeprevir 12Wks 150 mg PR12/24 
Phase = Overall Study Period 
2) By SVR12	
	SVR12 No	SVR12 Yes		
	12 Weeks 
Treatment	All Subjects	12 Weeks 
Treatment	All Subjects		
Week 4						
N	41	41	76	76		
Mean	-0.1318	-0.1318	-0.1065	-0.1065		
SE	0.03338	0.03338	0.02417	0.02417		
SD	0.21373	0.21373	0.21068	0.21068		
95% C.I. *	(-0.19927; -0.06434)	(-0.19927; -0.06434)	(-0.15468; -0.05840)	(-0.15468; -0.05840)		
Min	-0.719	-0.719	-0.665	-0.665		
Q1	-0.2340	-0.2340	-0.2480	-0.2480		
Median	-0.0840	-0.0840	-0.0435	-0.0435		
Q3	0.0000	0.0000	0.0000	0.0000		
Max	0.485	0.485	0.656	0.656		
	
Week 8						
N	41	41	71	71		
Mean	-0.1307	-0.1307	-0.1038	-0.1038		
SE	0.03321	0.03321	0.02560	0.02560		
SD	0.21262	0.21262	0.21575	0.21575		
95% C.I. *	(-0.19777; -0.06355)	(-0.19777; -0.06355)	(-0.15484; -0.05271)	(-0.15484; -0.05271)		
Min	-0.809	-0.809	-0.624	-0.624		
Q1	-0.2320	-0.2320	-0.2220	-0.2220		
Median	-0.1290	-0.1290	-0.0650	-0.0650		
Q3	-0.0250	-0.0250	0.0000	0.0000		
Max	0.485	0.485	0.656	0.656		
	
Week 12						
N	40	40	75	75		
Mean	-0.1361	-0.1361	-0.1198	-0.1198		
SE	0.03683	0.03683	0.02555	0.02555		
SD	0.23292	0.23292	0.22131	0.22131		
95% C.I. *	(-0.21062; -0.06163)	(-0.21062; -0.06163)	(-0.17076; -0.06892)	(-0.17076; -0.06892)		
Min	-0.949	-0.949	-0.911	-0.911		
Q1	-0.2460	-0.2460	-0.2640	-0.2640		
Median	-0.0940	-0.0940	-0.0940	-0.0940		
Q3	0.0000	0.0000	0.0000	0.0000		
Max	0.485	0.485	0.698	0.698		
	
Week 16						
N	41	41	74	74		
Mean	-0.0335	-0.0335	0.0124	0.0124		
SE	0.03429	0.03429	0.02151	0.02151		
SD	0.21957	0.21957	0.18507	0.18507		
95% C.I. *	(-0.10279; 0.03582)	(-0.10279; 0.03582)	(-0.03046; 0.05530)	(-0.03046; 0.05530)		
Min	-0.534	-0.534	-0.559	-0.559		
Q1	-0.1300	-0.1300	0.0000	0.0000		
Median	0.0000	0.0000	0.0000	0.0000		
Q3	0.0690	0.0690	0.0590	0.0590		
Max	0.485	0.485	0.698	0.698		
	
Week 24						
N	37	37	75	75		
Mean	-0.0251	-0.0251	0.0345	0.0345		
SE	0.02616	0.02616	0.01875	0.01875		
SD	0.15911	0.15911	0.16238	0.16238		
95% C.I. *	(-0.07810; 0.02800)	(-0.07810; 0.02800)	(-0.00291; 0.07181)	(-0.00291; 0.07181)		
Min	-0.409	-0.409	-0.365	-0.365		
Q1	-0.1140	-0.1140	0.0000	0.0000		
Median	0.0000	0.0000	0.0000	0.0000		
Q3	0.0590	0.0590	0.1210	0.1210		
Max	0.302	0.302	0.819	0.819		
	
Week 36						
N	3	3	5	5		
Mean	0.0030	0.0030	0.0788	0.0788		
SE	0.06843	0.06843	0.06901	0.06901		
SD	0.11853	0.11853	0.15431	0.15431		
95% C.I. *	(-0.29144; 0.29744)	(-0.29144; 0.29744)	(-0.11280; 0.27040)	(-0.11280; 0.27040)		
Min	-0.114	-0.114	0.000	0.000		
Q1	-0.1140	-0.1140	0.0000	0.0000		
Median	0.0000	0.0000	0.0000	0.0000		
Q3	0.1230	0.1230	0.0410	0.0410		
Max	0.123	0.123	0.353	0.353		
	

* Confidence interval for mean
Subjects with planned end of treatment at Week 12 do not have EQ-5Q, CES-D, FSS or WPAI results at Week 20.
Result from the EQ-5D Valuation Index range from -0.594 to 1 with higher scores indicating better outcome.	
[TPROEQ10.rtf] [\STAT\Analyses\Programs\Primary Analysis\Final4\2.TLF\7.PRO_PA\PRO_PA.sas] 15JAN2015, 16:51	

TPROEQ10:	Descriptive Statistics of the Changes in the EQ-5D Valuation Index per Analysis Timepoint; Intent-to-Treat (Study TMC435HPC3014)
Treatment Group = Simeprevir 12Wks 150 mg PR12/24 
Phase = Overall Study Period 
3) By Region	
	Europe		
	12 Weeks 
Treatment	>12 Weeks 
Treatment	All Subjects		
Week 4					
N	117	36	153		
Mean	-0.1154	-0.1325	-0.1194		
SE	0.01952	0.02770	0.01626		
SD	0.21118	0.16619	0.20112		
95% C.I. *	(-0.15406; -0.07672)	(-0.18873; -0.07627)	(-0.15154; -0.08729)		
Min	-0.719	-0.475	-0.719		
Q1	-0.2340	-0.2690	-0.2340		
Median	-0.0520	-0.0665	-0.0520		
Q3	0.0000	0.0000	0.0000		
Max	0.656	0.163	0.656		
	
Week 8					
N	112	35	147		
Mean	-0.1136	-0.1439	-0.1208		
SE	0.02023	0.03378	0.01736		
SD	0.21404	0.19984	0.21047		
95% C.I. *	(-0.15369; -0.07354)	(-0.21259; -0.07530)	(-0.15515; -0.08653)		
Min	-0.809	-0.923	-0.923		
Q1	-0.2310	-0.2330	-0.2320		
Median	-0.0985	-0.1110	-0.1010		
Q3	0.0000	0.0000	0.0000		
Max	0.656	0.163	0.656		
	
Week 12					
N	115	30	145		
Mean	-0.1255	-0.1793	-0.1366		
SE	0.02094	0.03117	0.01786		
SD	0.22453	0.17072	0.21508		
95% C.I. *	(-0.16698; -0.08403)	(-0.24308; -0.11559)	(-0.17195; -0.10134)		
Min	-0.949	-0.693	-0.949		
Q1	-0.2590	-0.2720	-0.2600		
Median	-0.0940	-0.1525	-0.1110		
Q3	0.0000	-0.0080	0.0000		
Max	0.698	0.059	0.698		
	
Week 16					
N	115	32	147		
Mean	-0.0039	-0.1310	-0.0316		
SE	0.01849	0.03423	0.01679		
SD	0.19834	0.19363	0.20357		
95% C.I. *	(-0.04059; 0.03269)	(-0.20078; -0.06116)	(-0.06478; 0.00159)		
Min	-0.559	-0.613	-0.613		
Q1	-0.0980	-0.1850	-0.1230		
Median	0.0000	-0.0725	0.0000		
Q3	0.0690	0.0000	0.0280		
Max	0.698	0.240	0.698		
	
Week 20					
N		28	28		
Mean		-0.1580	-0.1580		
SE		0.04157	0.04157		
SD		0.21995	0.21995		
95% C.I. *		(-0.24325; -0.07268)	(-0.24325; -0.07268)		
Min		-0.661	-0.661		
Q1		-0.2955	-0.2955		
Median		-0.0420	-0.0420		
Q3		0.0000	0.0000		
Max		0.121	0.121		
	
Week 24					
N	112	28	140		
Mean	0.0148	-0.1046	-0.0091		
SE	0.01540	0.02812	0.01410		
SD	0.16303	0.14881	0.16680		
95% C.I. *	(-0.01573; 0.04532)	(-0.16231; -0.04691)	(-0.03696; 0.01879)		
Min	-0.409	-0.467	-0.467		
Q1	-0.0060	-0.2245	-0.0715		
Median	0.0000	-0.0355	0.0000		
Q3	0.1025	0.0000	0.0540		
Max	0.819	0.121	0.819		
	
Week 36					
N	8		8		
Mean	0.0504		0.0504		
SE	0.04894		0.04894		
SD	0.13842		0.13842		
95% C.I. *	(-0.06534; 0.16609)		(-0.06534; 0.16609)		
Min	-0.114		-0.114		
Q1	0.0000		0.0000		
Median	0.0000		0.0000		
Q3	0.0820		0.0820		
Max	0.353		0.353		
	

* Confidence interval for mean
Subjects with planned end of treatment at Week 12 do not have EQ-5Q, CES-D, FSS or WPAI results at Week 20.
Result from the EQ-5D Valuation Index range from -0.594 to 1 with higher scores indicating better outcome.	
[TPROEQ10.rtf] [\STAT\Analyses\Programs\Primary Analysis\Final4\2.TLF\7.PRO_PA\PRO_PA.sas] 15JAN2015, 16:51	
