# Supplementary material for: An Open-Label Trial of 12-Week Simeprevir plus Peginterferon/Ribavirin (PR) in Treatment-Naïve Patients with Hepatitis C Virus (HCV) Genotype 1 (GT1)
Source: PLoS One. 2016 Jul 18;11(7):e0158526. doi: 10.1371/journal.pone.0158526 (PMC4948848; doi:10.1371/journal.pone.0158526)
Supplement: S1 Dataset — (ZIP) [file pone.0158526.s009.zip › Patient-reported Outcomes/TPROFSS03.rtf]

TPROFSS03:	Descriptive Statistics of the Changes from Baseline in the Total Fatigue Severity Score per Analysis Timepoint - Available Data Approach(;) Intent-to-Treat (Study TMC435HPC3014)
Treatment Group = Simeprevir 12Wks 150 mg PR12/24 
Phase = Overall Study Period 
1) Overall	
	12 Weeks 
Treatment	>12 Weeks 
Treatment	All Subjects		
Week 4					
N	118	37	155		
Mean	0.902	0.868	0.894		
SE	0.1691	0.3491	0.1528		
SD	1.8369	2.1237	1.9021		
95% C.I. *	(0.5673; 1.2371)	(0.1598; 1.5760)	(0.5922; 1.1958)		
Min	-4.00	-4.00	-4.00		
Q1	-0.250	-0.333	-0.333		
Median	0.556	0.556	0.556		
Q3	2.000	2.333	2.111		
Max	5.44	4.33	5.44		
	
Week 8					
N	119	35	154		
Mean	1.128	0.946	1.087		
SE	0.1735	0.2752	0.1477		
SD	1.8928	1.6282	1.8325		
95% C.I. *	(0.7847; 1.4719)	(0.3867; 1.5053)	(0.7951; 1.3786)		
Min	-4.00	-2.22	-4.00		
Q1	-0.222	0.000	-0.111		
Median	0.778	0.444	0.722		
Q3	2.667	2.444	2.556		
Max	5.78	4.44	5.78		
	
Week 12					
N	115	30	145		
Mean	1.236	1.315	1.252		
SE	0.1827	0.3071	0.1578		
SD	1.9593	1.6823	1.9000		
95% C.I. *	(0.8739; 1.5978)	(0.6866; 1.9430)	(0.9403; 1.5641)		
Min	-4.00	-1.44	-4.00		
Q1	-0.111	0.111	0.000		
Median	1.111	0.778	1.000		
Q3	2.667	2.444	2.667		
Max	5.56	4.67	5.56		
	
Week 16					
N	116	32	148		
Mean	0.021	0.960	0.224		
SE	0.1544	0.3053	0.1410		
SD	1.6631	1.7271	1.7156		
95% C.I. *	(-0.2850; 0.3267)	(0.3370; 1.5823)	(-0.0549; 0.5025)		
Min	-4.44	-2.33	-4.44		
Q1	-0.889	-0.111	-0.667		
Median	0.000	0.833	0.111		
Q3	0.778	2.056	0.944		
Max	4.33	5.22	5.22		
	
Week 20					
N		28	28		
Mean		1.017	1.017		
SE		0.2948	0.2948		
SD		1.5597	1.5597		
95% C.I. *		(0.4121; 1.6217)	(0.4121; 1.6217)		
Min		-1.44	-1.44		
Q1		0.056	0.056		
Median		0.611	0.611		
Q3		1.667	1.667		
Max		4.56	4.56		
	
Week 24					
N	106	29	135		
Mean	-0.352	0.454	-0.178		
SE	0.1610	0.3595	0.1502		
SD	1.6575	1.9358	1.7453		
95% C.I. *	(-0.6708; -0.0323)	(-0.2823; 1.1904)	(-0.4756; 0.1186)		
Min	-4.67	-3.67	-4.67		
Q1	-1.444	-0.889	-1.111		
Median	-0.222	0.222	-0.222		
Q3	0.556	1.889	0.667		
Max	3.67	4.44	4.44		
	
Week 36					
N	7		7		
Mean	-0.651		-0.651		
SE	0.7408		0.7408		
SD	1.9599		1.9599		
95% C.I. *	(-2.4634; 1.1618)		(-2.4634; 1.1618)		
Min	-4.00		-4.00		
Q1	-1.556		-1.556		
Median	-0.667		-0.667		
Q3	0.444		0.444		
Max	2.33		2.33		
	

* Confidence interval for mean
Subjects with planned end of treatment at Week 12 do not have EQ-5Q, CES-D, FSS or WPAI results at Week 20.
The FSS score ranges from 1 to 7, with higher scores indicating more fatigue.	
[TPROFSS03.rtf] [\STAT\Analyses\Programs\Primary Analysis\Final4\2.TLF\7.PRO_PA\PRO_PA.sas] 15JAN2015, 16:51	

TPROFSS03:	Descriptive Statistics of the Changes from Baseline in the Total Fatigue Severity Score per Analysis Timepoint - Available Data Approach(;) Intent-to-Treat (Study TMC435HPC3014)
Treatment Group = Simeprevir 12Wks 150 mg PR12/24 
Phase = Overall Study Period 
2) By SVR12	
	SVR12 No	SVR12 Yes		
	12 Weeks 
Treatment	All Subjects	12 Weeks 
Treatment	All Subjects		
Week 4						
N	41	41	77	77		
Mean	0.684	0.684	1.018	1.018		
SE	0.2879	0.2879	0.2091	0.2091		
SD	1.8434	1.8434	1.8348	1.8348		
95% C.I. *	(0.1021; 1.2658)	(0.1021; 1.2658)	(0.6019; 1.4349)	(0.6019; 1.4349)		
Min	-3.33	-3.33	-4.00	-4.00		
Q1	-0.333	-0.333	-0.111	-0.111		
Median	0.444	0.444	0.778	0.778		
Q3	1.889	1.889	2.333	2.333		
Max	4.11	4.11	5.44	5.44		
	
Week 8						
N	42	42	77	77		
Mean	1.222	1.222	1.077	1.077		
SE	0.3042	0.3042	0.2119	0.2119		
SD	1.9715	1.9715	1.8596	1.8596		
95% C.I. *	(0.6079; 1.8366)	(0.6079; 1.8366)	(0.6549; 1.4991)	(0.6549; 1.4991)		
Min	-3.11	-3.11	-4.00	-4.00		
Q1	-0.222	-0.222	-0.222	-0.222		
Median	0.889	0.889	0.778	0.778		
Q3	2.667	2.667	2.667	2.667		
Max	5.78	5.78	5.00	5.00		
	
Week 12						
N	41	41	74	74		
Mean	1.318	1.318	1.190	1.190		
SE	0.3256	0.3256	0.2208	0.2208		
SD	2.0846	2.0846	1.8995	1.8995		
95% C.I. *	(0.6604; 1.9764)	(0.6604; 1.9764)	(0.7501; 1.6302)	(0.7501; 1.6302)		
Min	-3.22	-3.22	-4.00	-4.00		
Q1	-0.222	-0.222	-0.111	-0.111		
Median	1.444	1.444	1.000	1.000		
Q3	2.667	2.667	2.778	2.778		
Max	5.56	5.56	5.22	5.22		
	
Week 16						
N	42	42	74	74		
Mean	0.128	0.128	-0.040	-0.040		
SE	0.2343	0.2343	0.2031	0.2031		
SD	1.5188	1.5188	1.7468	1.7468		
95% C.I. *	(-0.3450; 0.6016)	(-0.3450; 0.6016)	(-0.4449; 0.3645)	(-0.4449; 0.3645)		
Min	-3.22	-3.22	-4.44	-4.44		
Q1	-0.667	-0.667	-1.000	-1.000		
Median	-0.111	-0.111	0.000	0.000		
Q3	0.778	0.778	0.889	0.889		
Max	3.89	3.89	4.33	4.33		
	
Week 24						
N	37	37	69	69		
Mean	-0.066	-0.066	-0.505	-0.505		
SE	0.3000	0.3000	0.1869	0.1869		
SD	1.8251	1.8251	1.5525	1.5525		
95% C.I. *	(-0.6746; 0.5424)	(-0.6746; 0.5424)	(-0.8776; -0.1317)	(-0.8776; -0.1317)		
Min	-4.67	-4.67	-4.00	-4.00		
Q1	-0.667	-0.667	-1.556	-1.556		
Median	-0.111	-0.111	-0.222	-0.222		
Q3	0.889	0.889	0.333	0.333		
Max	3.44	3.44	3.67	3.67		
	
Week 36						
N	2	2	5	5		
Mean	1.389	1.389	-1.467	-1.467		
SE	0.9444	0.9444	0.6934	0.6934		
SD	1.3356	1.3356	1.5504	1.5504		
95% C.I. *	(-10.6114; 13.3892)	(-10.6114; 13.3892)	(-3.3917; 0.4584)	(-3.3917; 0.4584)		
Min	0.44	0.44	-4.00	-4.00		
Q1	0.444	0.444	-1.556	-1.556		
Median	1.389	1.389	-1.222	-1.222		
Q3	2.333	2.333	-0.667	-0.667		
Max	2.33	2.33	0.11	0.11		
	

* Confidence interval for mean
Subjects with planned end of treatment at Week 12 do not have EQ-5Q, CES-D, FSS or WPAI results at Week 20.
The FSS score ranges from 1 to 7, with higher scores indicating more fatigue.	
[TPROFSS03.rtf] [\STAT\Analyses\Programs\Primary Analysis\Final4\2.TLF\7.PRO_PA\PRO_PA.sas] 15JAN2015, 16:51	

TPROFSS03:	Descriptive Statistics of the Changes from Baseline in the Total Fatigue Severity Score per Analysis Timepoint - Available Data Approach(;) Intent-to-Treat (Study TMC435HPC3014)
Treatment Group = Simeprevir 12Wks 150 mg PR12/24 
Phase = Overall Study Period 
3) By Region	
	Europe		
	12 Weeks 
Treatment	>12 Weeks 
Treatment	All Subjects		
Week 4					
N	118	37	155		
Mean	0.902	0.868	0.894		
SE	0.1691	0.3491	0.1528		
SD	1.8369	2.1237	1.9021		
95% C.I. *	(0.5673; 1.2371)	(0.1598; 1.5760)	(0.5922; 1.1958)		
Min	-4.00	-4.00	-4.00		
Q1	-0.250	-0.333	-0.333		
Median	0.556	0.556	0.556		
Q3	2.000	2.333	2.111		
Max	5.44	4.33	5.44		
	
Week 8					
N	119	35	154		
Mean	1.128	0.946	1.087		
SE	0.1735	0.2752	0.1477		
SD	1.8928	1.6282	1.8325		
95% C.I. *	(0.7847; 1.4719)	(0.3867; 1.5053)	(0.7951; 1.3786)		
Min	-4.00	-2.22	-4.00		
Q1	-0.222	0.000	-0.111		
Median	0.778	0.444	0.722		
Q3	2.667	2.444	2.556		
Max	5.78	4.44	5.78		
	
Week 12					
N	115	30	145		
Mean	1.236	1.315	1.252		
SE	0.1827	0.3071	0.1578		
SD	1.9593	1.6823	1.9000		
95% C.I. *	(0.8739; 1.5978)	(0.6866; 1.9430)	(0.9403; 1.5641)		
Min	-4.00	-1.44	-4.00		
Q1	-0.111	0.111	0.000		
Median	1.111	0.778	1.000		
Q3	2.667	2.444	2.667		
Max	5.56	4.67	5.56		
	
Week 16					
N	116	32	148		
Mean	0.021	0.960	0.224		
SE	0.1544	0.3053	0.1410		
SD	1.6631	1.7271	1.7156		
95% C.I. *	(-0.2850; 0.3267)	(0.3370; 1.5823)	(-0.0549; 0.5025)		
Min	-4.44	-2.33	-4.44		
Q1	-0.889	-0.111	-0.667		
Median	0.000	0.833	0.111		
Q3	0.778	2.056	0.944		
Max	4.33	5.22	5.22		
	
Week 20					
N		28	28		
Mean		1.017	1.017		
SE		0.2948	0.2948		
SD		1.5597	1.5597		
95% C.I. *		(0.4121; 1.6217)	(0.4121; 1.6217)		
Min		-1.44	-1.44		
Q1		0.056	0.056		
Median		0.611	0.611		
Q3		1.667	1.667		
Max		4.56	4.56		
	
Week 24					
N	106	29	135		
Mean	-0.352	0.454	-0.178		
SE	0.1610	0.3595	0.1502		
SD	1.6575	1.9358	1.7453		
95% C.I. *	(-0.6708; -0.0323)	(-0.2823; 1.1904)	(-0.4756; 0.1186)		
Min	-4.67	-3.67	-4.67		
Q1	-1.444	-0.889	-1.111		
Median	-0.222	0.222	-0.222		
Q3	0.556	1.889	0.667		
Max	3.67	4.44	4.44		
	
Week 36					
N	7		7		
Mean	-0.651		-0.651		
SE	0.7408		0.7408		
SD	1.9599		1.9599		
95% C.I. *	(-2.4634; 1.1618)		(-2.4634; 1.1618)		
Min	-4.00		-4.00		
Q1	-1.556		-1.556		
Median	-0.667		-0.667		
Q3	0.444		0.444		
Max	2.33		2.33		
	

* Confidence interval for mean
Subjects with planned end of treatment at Week 12 do not have EQ-5Q, CES-D, FSS or WPAI results at Week 20.
The FSS score ranges from 1 to 7, with higher scores indicating more fatigue.	
[TPROFSS03.rtf] [\STAT\Analyses\Programs\Primary Analysis\Final4\2.TLF\7.PRO_PA\PRO_PA.sas] 15JAN2015, 16:51	
