# Supplementary material for: An Open-Label Trial of 12-Week Simeprevir plus Peginterferon/Ribavirin (PR) in Treatment-Naïve Patients with Hepatitis C Virus (HCV) Genotype 1 (GT1)
Source: PLoS One. 2016 Jul 18;11(7):e0158526. doi: 10.1371/journal.pone.0158526 (PMC4948848; doi:10.1371/journal.pone.0158526)
Supplement: S1 Dataset — (ZIP) [file pone.0158526.s009.zip › Patient-reported Outcomes/TPROWP03.rtf]

TPROWP03:	Descriptive Statistics of the Change from Baseline in WPAI Scores per Analysis Timepoint  Available Data Approach; Intent-to-Treat (Study TMC435HPC3014)
Treatment Group = Simeprevir 12Wks 150 mg PR12/24 
Phase = Overall Study Period 
1) Overall 
1a. WPAI Total Work Impairment	
	12 Weeks 
Treatment	>12 Weeks 
Treatment	All Subjects		
Week 4					
N	46	16	62		
Mean	22.4	25.1	23.1		
SE	4.43	7.28	3.76		
SD	30.06	29.12	29.60		
95% C.I. *	(13.52; 31.38)	(9.56; 40.60)	(15.61; 30.65)		
Min	-40	0	-40		
Q1	0.0	0.0	0.0		
Median	18.0	10.0	18.0		
Q3	39.9	50.0	40.0		
Max	100	73	100		
	
Week 8					
N	46	18	64		
Mean	22.4	22.4	22.4		
SE	4.00	7.05	3.46		
SD	27.11	29.93	27.69		
95% C.I. *	(14.33; 30.44)	(7.55; 37.31)	(15.48; 29.31)		
Min	-18	-10	-18		
Q1	0.0	0.0	0.0		
Median	10.0	0.0	10.0		
Q3	40.0	50.0	41.4		
Max	100	92	100		
	
Week 12					
N	45	11	56		
Mean	25.5	31.8	26.7		
SE	4.14	9.89	3.82		
SD	27.77	32.79	28.62		
95% C.I. *	(17.15; 33.84)	(9.79; 53.85)	(19.08; 34.40)		
Min	-40	-10	-40		
Q1	5.0	0.0	0.0		
Median	20.0	20.0	20.0		
Q3	40.0	63.8	48.8		
Max	100	81	100		
	
Week 16					
N	45	11	56		
Mean	3.3	24.3	7.4		
SE	3.24	10.62	3.47		
SD	21.70	35.21	25.94		
95% C.I. *	(-3.24; 9.80)	(0.60; 47.91)	(0.46; 14.35)		
Min	-40	-10	-40		
Q1	0.0	0.0	0.0		
Median	0.0	0.0	0.0		
Q3	0.0	70.0	15.0		
Max	100	87	100		
	
Week 20					
N		10	10		
Mean		36.2	36.2		
SE		12.65	12.65		
SD		40.00	40.00		
95% C.I. *		(7.54; 64.78)	(7.54; 64.78)		
Min		0	0		
Q1		0.0	0.0		
Median		20.0	20.0		
Q3		76.7	76.7		
Max		100	100		
	
Week 24					
N	51	13	64		
Mean	-3.5	25.0	2.3		
SE	2.89	9.55	3.30		
SD	20.66	34.45	26.42		
95% C.I. *	(-9.33; 2.30)	(4.14; 45.78)	(-4.33; 8.87)		
Min	-65	-10	-65		
Q1	0.0	0.0	0.0		
Median	0.0	10.0	0.0		
Q3	0.0	40.0	0.0		
Max	80	100	100		
	
Week 36					
N	4		4		
Mean	17.5		17.5		
SE	14.36		14.36		
SD	28.72		28.72		
95% C.I. *	(-28.20; 63.20)		(-28.20; 63.20)		
Min	0		0		
Q1	0.0		0.0		
Median	5.0		5.0		
Q3	35.0		35.0		
Max	60		60		
	

* Confidence interval for mean
Subjects with planned end of treatment at Week 12 do not have EQ-5Q, CES-D, FSS or WPAI results at Week 20.
The WPAI Total Work Impairment Score ranges from 0 to 100, with higher scores indicating worse outcome. 
The WPAI Total Work Impairment Score is derived from the WPAI absenteeism score (questions 2 and 4 fromthe WPAI questionnaire) and WPAI presenteeism score (question 5 of the WPAI questionnaire).	
[TPROWP03.rtf] [\STAT\Analyses\Programs\Primary Analysis\Final4\2.TLF\7.PRO_PA\PRO_PA.sas] 15JAN2015, 16:51	

TPROWP03:	Descriptive Statistics of the Change from Baseline in WPAI Scores per Analysis Timepoint  Available Data Approach; Intent-to-Treat (Study TMC435HPC3014)
Treatment Group = Simeprevir 12Wks 150 mg PR12/24 
Phase = Overall Study Period 
1) Overall 
1b. WPAI Daily Activity Impairment	
	12 Weeks 
Treatment	>12 Weeks 
Treatment	All Subjects		
Week 4					
N	103	33	136		
Mean	15.9	21.8	17.4		
SE	2.51	5.36	2.30		
SD	25.45	30.77	26.84		
95% C.I. *	(10.95; 20.90)	(10.91; 32.73)	(12.80; 21.90)		
Min	-50	-40	-50		
Q1	0.0	0.0	0.0		
Median	10.0	10.0	10.0		
Q3	30.0	50.0	30.0		
Max	90	80	90		
	
Week 8					
N	100	30	130		
Mean	20.0	20.0	20.0		
SE	2.77	5.53	2.48		
SD	27.74	30.29	28.23		
95% C.I. *	(14.50; 25.50)	(8.69; 31.31)	(15.10; 24.90)		
Min	-50	-40	-50		
Q1	0.0	0.0	0.0		
Median	20.0	10.0	15.0		
Q3	40.0	40.0	40.0		
Max	100	80	100		
	
Week 12					
N	100	23	123		
Mean	18.9	25.2	20.1		
SE	2.93	5.72	2.61		
SD	29.33	27.45	28.99		
95% C.I. *	(13.08; 24.72)	(13.35; 37.09)	(14.91; 25.26)		
Min	-50	-20	-50		
Q1	0.0	0.0	0.0		
Median	10.0	20.0	10.0		
Q3	35.0	50.0	40.0		
Max	100	80	100		
	
Week 16					
N	96	24	120		
Mean	1.5	21.3	5.4		
SE	2.82	6.18	2.66		
SD	27.61	30.26	29.13		
95% C.I. *	(-4.14; 7.05)	(8.47; 34.03)	(0.15; 10.68)		
Min	-70	-10	-70		
Q1	-10.0	0.0	-5.0		
Median	0.0	5.0	0.0		
Q3	10.0	40.0	10.0		
Max	80	80	80		
	
Week 20					
N		26	26		
Mean		21.2	21.2		
SE		5.39	5.39		
SD		27.47	27.47		
95% C.I. *		(10.06; 32.25)	(10.06; 32.25)		
Min		-20	-20		
Q1		0.0	0.0		
Median		15.0	15.0		
Q3		40.0	40.0		
Max		80	80		
	
Week 24					
N	95	24	119		
Mean	-6.2	20.0	-0.9		
SE	2.72	6.92	2.74		
SD	26.54	33.88	29.94		
95% C.I. *	(-11.62; -0.80)	(5.69; 34.31)	(-6.36; 4.51)		
Min	-90	-40	-90		
Q1	-10.0	0.0	-10.0		
Median	0.0	10.0	0.0		
Q3	0.0	50.0	0.0		
Max	80	80	80		
	
Week 36					
N	8		8		
Mean	0.0		0.0		
SE	11.65		11.65		
SD	32.95		32.95		
95% C.I. *	(-27.55; 27.55)		(-27.55; 27.55)		
Min	-70		-70		
Q1	0.0		0.0		
Median	0.0		0.0		
Q3	10.0		10.0		
Max	50		50		
	

* Confidence interval for mean
Subjects with planned end of treatment at Week 12 do not have EQ-5Q, CES-D, FSS or WPAI results at Week 20.
The WPAI Daily Activities Impairment Score ranges from 0 to 100, with higher scores indicating worse outcome.
The WPAI Daily Activities Impairment Score is derived from impact on daily activities (question 6 from theWPAI questionnaire)	
[TPROWP03.rtf] [\STAT\Analyses\Programs\Primary Analysis\Final4\2.TLF\7.PRO_PA\PRO_PA.sas] 15JAN2015, 16:51	

TPROWP03:	Descriptive Statistics of the Change from Baseline in WPAI Scores per Analysis Timepoint  Available Data Approach; Intent-to-Treat (Study TMC435HPC3014)
Treatment Group = Simeprevir 12Wks 150 mg PR12/24 
Phase = Overall Study Period 
2) By SVR12 
2a. WPAI Total Work Impairment	
	SVR12 No	SVR12 Yes		
	12 Weeks 
Treatment	All Subjects	12 Weeks 
Treatment	All Subjects		
Week 4						
N	22	22	24	24		
Mean	26.9	26.9	18.4	18.4		
SE	7.70	7.70	4.75	4.75		
SD	36.09	36.09	23.28	23.28		
95% C.I. *	(10.91; 42.91)	(10.91; 42.91)	(8.53; 28.19)	(8.53; 28.19)		
Min	-40	-40	0	0		
Q1	0.0	0.0	0.0	0.0		
Median	20.4	20.4	13.3	13.3		
Q3	48.3	48.3	20.0	20.0		
Max	100	100	80	80		
	
Week 8						
N	22	22	24	24		
Mean	21.7	21.7	23.0	23.0		
SE	5.52	5.52	5.87	5.87		
SD	25.87	25.87	28.75	28.75		
95% C.I. *	(10.24; 33.18)	(10.24; 33.18)	(10.86; 35.14)	(10.86; 35.14)		
Min	-18	-18	0	0		
Q1	0.0	0.0	0.0	0.0		
Median	17.0	17.0	10.0	10.0		
Q3	42.9	42.9	40.0	40.0		
Max	66	66	100	100		
	
Week 12						
N	22	22	23	23		
Mean	29.1	29.1	22.0	22.0		
SE	6.05	6.05	5.70	5.70		
SD	28.40	28.40	27.33	27.33		
95% C.I. *	(16.52; 41.70)	(16.52; 41.70)	(10.22; 33.86)	(10.22; 33.86)		
Min	-40	-40	0	0		
Q1	10.0	10.0	0.0	0.0		
Median	31.0	31.0	10.0	10.0		
Q3	50.0	50.0	30.0	30.0		
Max	76	76	100	100		
	
Week 16						
N	22	22	23	23		
Mean	7.2	7.2	-0.4	-0.4		
SE	6.13	6.13	2.31	2.31		
SD	28.76	28.76	11.09	11.09		
95% C.I. *	(-5.56; 19.93)	(-5.56; 19.93)	(-5.24; 4.35)	(-5.24; 4.35)		
Min	-40	-40	-20	-20		
Q1	0.0	0.0	0.0	0.0		
Median	0.0	0.0	0.0	0.0		
Q3	20.0	20.0	0.0	0.0		
Max	100	100	30	30		
	
Week 24						
N	24	24	27	27		
Mean	-3.5	-3.5	-3.5	-3.5		
SE	5.70	5.70	2.21	2.21		
SD	27.92	27.92	11.47	11.47		
95% C.I. *	(-15.33; 8.25)	(-15.33; 8.25)	(-8.03; 1.04)	(-8.03; 1.04)		
Min	-65	-65	-54	-54		
Q1	-20.0	-20.0	0.0	0.0		
Median	0.0	0.0	0.0	0.0		
Q3	0.0	0.0	0.0	0.0		
Max	80	80	10	10		
	
Week 36						
N	2	2	2	2		
Mean	35.0	35.0	0.0	0.0		
SE	25.00	25.00	0.00	0.00		
SD	35.36	35.36	0.00	0.00		
95% C.I. *	(-282.66; 352.66)	(-282.66; 352.66)	(0.00; 0.00)	(0.00; 0.00)		
Min	10	10	0	0		
Q1	10.0	10.0	0.0	0.0		
Median	35.0	35.0	0.0	0.0		
Q3	60.0	60.0	0.0	0.0		
Max	60	60	0	0		
	

* Confidence interval for mean
Subjects with planned end of treatment at Week 12 do not have EQ-5Q, CES-D, FSS or WPAI results at Week 20.
The WPAI Total Work Impairment Score ranges from 0 to 100, with higher scores indicating worse outcome. 
The WPAI Total Work Impairment Score is derived from the WPAI absenteeism score (questions 2 and 4 fromthe WPAI questionnaire) and WPAI presenteeism score (question 5 of the WPAI questionnaire).	
[TPROWP03.rtf] [\STAT\Analyses\Programs\Primary Analysis\Final4\2.TLF\7.PRO_PA\PRO_PA.sas] 15JAN2015, 16:51	

TPROWP03:	Descriptive Statistics of the Change from Baseline in WPAI Scores per Analysis Timepoint  Available Data Approach; Intent-to-Treat (Study TMC435HPC3014)
Treatment Group = Simeprevir 12Wks 150 mg PR12/24 
Phase = Overall Study Period 
2) By SVR12 
2b. WPAI Daily Activity Impairment	
	SVR12 No	SVR12 Yes		
	12 Weeks 
Treatment	All Subjects	12 Weeks 
Treatment	All Subjects		
Week 4						
N	37	37	66	66		
Mean	15.1	15.1	16.4	16.4		
SE	3.63	3.63	3.36	3.36		
SD	22.06	22.06	27.32	27.32		
95% C.I. *	(7.78; 22.49)	(7.78; 22.49)	(9.65; 23.08)	(9.65; 23.08)		
Min	-50	-50	-30	-30		
Q1	0.0	0.0	0.0	0.0		
Median	10.0	10.0	10.0	10.0		
Q3	20.0	20.0	30.0	30.0		
Max	70	70	90	90		
	
Week 8						
N	35	35	65	65		
Mean	19.7	19.7	20.2	20.2		
SE	3.59	3.59	3.83	3.83		
SD	21.21	21.21	30.85	30.85		
95% C.I. *	(12.43; 27.00)	(12.43; 27.00)	(12.51; 27.80)	(12.51; 27.80)		
Min	-10	-10	-50	-50		
Q1	0.0	0.0	0.0	0.0		
Median	20.0	20.0	20.0	20.0		
Q3	30.0	30.0	40.0	40.0		
Max	80	80	100	100		
	
Week 12						
N	34	34	66	66		
Mean	22.9	22.9	16.8	16.8		
SE	4.50	4.50	3.79	3.79		
SD	26.23	26.23	30.79	30.79		
95% C.I. *	(13.79; 32.09)	(13.79; 32.09)	(9.25; 24.39)	(9.25; 24.39)		
Min	-20	-20	-50	-50		
Q1	0.0	0.0	0.0	0.0		
Median	20.0	20.0	10.0	10.0		
Q3	40.0	40.0	30.0	30.0		
Max	70	70	100	100		
	
Week 16						
N	36	36	60	60		
Mean	3.1	3.1	0.5	0.5		
SE	3.82	3.82	3.90	3.90		
SD	22.91	22.91	30.22	30.22		
95% C.I. *	(-4.69; 10.81)	(-4.69; 10.81)	(-7.31; 8.31)	(-7.31; 8.31)		
Min	-50	-50	-70	-70		
Q1	-10.0	-10.0	-10.0	-10.0		
Median	0.0	0.0	0.0	0.0		
Q3	10.0	10.0	10.0	10.0		
Max	80	80	80	80		
	
Week 24						
N	32	32	63	63		
Mean	-3.1	-3.1	-7.8	-7.8		
SE	4.22	4.22	3.51	3.51		
SD	23.89	23.89	27.85	27.85		
95% C.I. *	(-11.74; 5.49)	(-11.74; 5.49)	(-14.79; -0.76)	(-14.79; -0.76)		
Min	-60	-60	-90	-90		
Q1	-10.0	-10.0	-10.0	-10.0		
Median	0.0	0.0	0.0	0.0		
Q3	0.0	0.0	0.0	0.0		
Max	80	80	50	50		
	
Week 36						
N	3	3	5	5		
Mean	20.0	20.0	-12.0	-12.0		
SE	15.28	15.28	14.63	14.63		
SD	26.46	26.46	32.71	32.71		
95% C.I. *	(-45.72; 85.72)	(-45.72; 85.72)	(-52.62; 28.62)	(-52.62; 28.62)		
Min	0	0	-70	-70		
Q1	0.0	0.0	0.0	0.0		
Median	10.0	10.0	0.0	0.0		
Q3	50.0	50.0	0.0	0.0		
Max	50	50	10	10		
	

* Confidence interval for mean
Subjects with planned end of treatment at Week 12 do not have EQ-5Q, CES-D, FSS or WPAI results at Week 20.
The WPAI Daily Activities Impairment Score ranges from 0 to 100, with higher scores indicating worse outcome.
The WPAI Daily Activities Impairment Score is derived from impact on daily activities (question 6 from theWPAI questionnaire)	
[TPROWP03.rtf] [\STAT\Analyses\Programs\Primary Analysis\Final4\2.TLF\7.PRO_PA\PRO_PA.sas] 15JAN2015, 16:51	

TPROWP03:	Descriptive Statistics of the Change from Baseline in WPAI Scores per Analysis Timepoint  Available Data Approach; Intent-to-Treat (Study TMC435HPC3014)
Treatment Group = Simeprevir 12Wks 150 mg PR12/24 
Phase = Overall Study Period 
3) By Region 
3a. WPAI Total Work Impairment	
	Europe		
	12 Weeks 
Treatment	>12 Weeks 
Treatment	All Subjects		
Week 4					
N	46	16	62		
Mean	22.4	25.1	23.1		
SE	4.43	7.28	3.76		
SD	30.06	29.12	29.60		
95% C.I. *	(13.52; 31.38)	(9.56; 40.60)	(15.61; 30.65)		
Min	-40	0	-40		
Q1	0.0	0.0	0.0		
Median	18.0	10.0	18.0		
Q3	39.9	50.0	40.0		
Max	100	73	100		
	
Week 8					
N	46	18	64		
Mean	22.4	22.4	22.4		
SE	4.00	7.05	3.46		
SD	27.11	29.93	27.69		
95% C.I. *	(14.33; 30.44)	(7.55; 37.31)	(15.48; 29.31)		
Min	-18	-10	-18		
Q1	0.0	0.0	0.0		
Median	10.0	0.0	10.0		
Q3	40.0	50.0	41.4		
Max	100	92	100		
	
Week 12					
N	45	11	56		
Mean	25.5	31.8	26.7		
SE	4.14	9.89	3.82		
SD	27.77	32.79	28.62		
95% C.I. *	(17.15; 33.84)	(9.79; 53.85)	(19.08; 34.40)		
Min	-40	-10	-40		
Q1	5.0	0.0	0.0		
Median	20.0	20.0	20.0		
Q3	40.0	63.8	48.8		
Max	100	81	100		
	
Week 16					
N	45	11	56		
Mean	3.3	24.3	7.4		
SE	3.24	10.62	3.47		
SD	21.70	35.21	25.94		
95% C.I. *	(-3.24; 9.80)	(0.60; 47.91)	(0.46; 14.35)		
Min	-40	-10	-40		
Q1	0.0	0.0	0.0		
Median	0.0	0.0	0.0		
Q3	0.0	70.0	15.0		
Max	100	87	100		
	
Week 20					
N		10	10		
Mean		36.2	36.2		
SE		12.65	12.65		
SD		40.00	40.00		
95% C.I. *		(7.54; 64.78)	(7.54; 64.78)		
Min		0	0		
Q1		0.0	0.0		
Median		20.0	20.0		
Q3		76.7	76.7		
Max		100	100		
	
Week 24					
N	51	13	64		
Mean	-3.5	25.0	2.3		
SE	2.89	9.55	3.30		
SD	20.66	34.45	26.42		
95% C.I. *	(-9.33; 2.30)	(4.14; 45.78)	(-4.33; 8.87)		
Min	-65	-10	-65		
Q1	0.0	0.0	0.0		
Median	0.0	10.0	0.0		
Q3	0.0	40.0	0.0		
Max	80	100	100		
	
Week 36					
N	4		4		
Mean	17.5		17.5		
SE	14.36		14.36		
SD	28.72		28.72		
95% C.I. *	(-28.20; 63.20)		(-28.20; 63.20)		
Min	0		0		
Q1	0.0		0.0		
Median	5.0		5.0		
Q3	35.0		35.0		
Max	60		60		
	

* Confidence interval for mean
Subjects with planned end of treatment at Week 12 do not have EQ-5Q, CES-D, FSS or WPAI results at Week 20.
The WPAI Total Work Impairment Score ranges from 0 to 100, with higher scores indicating worse outcome. 
The WPAI Total Work Impairment Score is derived from the WPAI absenteeism score (questions 2 and 4 fromthe WPAI questionnaire) and WPAI presenteeism score (question 5 of the WPAI questionnaire).	
[TPROWP03.rtf] [\STAT\Analyses\Programs\Primary Analysis\Final4\2.TLF\7.PRO_PA\PRO_PA.sas] 15JAN2015, 16:51	

TPROWP03:	Descriptive Statistics of the Change from Baseline in WPAI Scores per Analysis Timepoint  Available Data Approach; Intent-to-Treat (Study TMC435HPC3014)
Treatment Group = Simeprevir 12Wks 150 mg PR12/24 
Phase = Overall Study Period 
3) By Region 
3b. WPAI Daily Activity Impairment	
	Europe		
	12 Weeks 
Treatment	>12 Weeks 
Treatment	All Subjects		
Week 4					
N	103	33	136		
Mean	15.9	21.8	17.4		
SE	2.51	5.36	2.30		
SD	25.45	30.77	26.84		
95% C.I. *	(10.95; 20.90)	(10.91; 32.73)	(12.80; 21.90)		
Min	-50	-40	-50		
Q1	0.0	0.0	0.0		
Median	10.0	10.0	10.0		
Q3	30.0	50.0	30.0		
Max	90	80	90		
	
Week 8					
N	100	30	130		
Mean	20.0	20.0	20.0		
SE	2.77	5.53	2.48		
SD	27.74	30.29	28.23		
95% C.I. *	(14.50; 25.50)	(8.69; 31.31)	(15.10; 24.90)		
Min	-50	-40	-50		
Q1	0.0	0.0	0.0		
Median	20.0	10.0	15.0		
Q3	40.0	40.0	40.0		
Max	100	80	100		
	
Week 12					
N	100	23	123		
Mean	18.9	25.2	20.1		
SE	2.93	5.72	2.61		
SD	29.33	27.45	28.99		
95% C.I. *	(13.08; 24.72)	(13.35; 37.09)	(14.91; 25.26)		
Min	-50	-20	-50		
Q1	0.0	0.0	0.0		
Median	10.0	20.0	10.0		
Q3	35.0	50.0	40.0		
Max	100	80	100		
	
Week 16					
N	96	24	120		
Mean	1.5	21.3	5.4		
SE	2.82	6.18	2.66		
SD	27.61	30.26	29.13		
95% C.I. *	(-4.14; 7.05)	(8.47; 34.03)	(0.15; 10.68)		
Min	-70	-10	-70		
Q1	-10.0	0.0	-5.0		
Median	0.0	5.0	0.0		
Q3	10.0	40.0	10.0		
Max	80	80	80		
	
Week 20					
N		26	26		
Mean		21.2	21.2		
SE		5.39	5.39		
SD		27.47	27.47		
95% C.I. *		(10.06; 32.25)	(10.06; 32.25)		
Min		-20	-20		
Q1		0.0	0.0		
Median		15.0	15.0		
Q3		40.0	40.0		
Max		80	80		
	
Week 24					
N	95	24	119		
Mean	-6.2	20.0	-0.9		
SE	2.72	6.92	2.74		
SD	26.54	33.88	29.94		
95% C.I. *	(-11.62; -0.80)	(5.69; 34.31)	(-6.36; 4.51)		
Min	-90	-40	-90		
Q1	-10.0	0.0	-10.0		
Median	0.0	10.0	0.0		
Q3	0.0	50.0	0.0		
Max	80	80	80		
	
Week 36					
N	8		8		
Mean	0.0		0.0		
SE	11.65		11.65		
SD	32.95		32.95		
95% C.I. *	(-27.55; 27.55)		(-27.55; 27.55)		
Min	-70		-70		
Q1	0.0		0.0		
Median	0.0		0.0		
Q3	10.0		10.0		
Max	50		50		
	

* Confidence interval for mean
Subjects with planned end of treatment at Week 12 do not have EQ-5Q, CES-D, FSS or WPAI results at Week 20.
The WPAI Daily Activities Impairment Score ranges from 0 to 100, with higher scores indicating worse outcome.
The WPAI Daily Activities Impairment Score is derived from impact on daily activities (question 6 from theWPAI questionnaire)	
[TPROWP03.rtf] [\STAT\Analyses\Programs\Primary Analysis\Final4\2.TLF\7.PRO_PA\PRO_PA.sas] 15JAN2015, 16:51	
