# Supplementary material for: An Open-Label Trial of 12-Week Simeprevir plus Peginterferon/Ribavirin (PR) in Treatment-Naïve Patients with Hepatitis C Virus (HCV) Genotype 1 (GT1)
Source: PLoS One. 2016 Jul 18;11(7):e0158526. doi: 10.1371/journal.pone.0158526 (PMC4948848; doi:10.1371/journal.pone.0158526)
Supplement: S1 Dataset — (ZIP) [file pone.0158526.s009.zip › Patient-reported Outcomes/TPROWP03A.rtf]

TPROWP03A:	Descriptive Statistics of the Change from Baseline in WPAI Daily Activities Impairment Score per Analysis Timepoint  Available Data Approach; Intent-to-Treat (Study TMC435HPC3014)
Treatment Group = Simeprevir 12Wks 150 mg PR12/24 
Phase = Overall Study Period 
1) Overall	
	12 Weeks 
Treatment	>12 Weeks 
Treatment	All Subjects		
Week 4					
N	103	33	136		
Mean (SD)	15.9 (25.45)	21.8 (30.77)	17.4 (26.84)		
Median	10.0	10.0	10.0		
Range	(-50; 90)	(-40; 80)	(-50; 90)		
	
Week 8					
N	100	30	130		
Mean (SD)	20.0 (27.74)	20.0 (30.29)	20.0 (28.23)		
Median	20.0	10.0	15.0		
Range	(-50; 100)	(-40; 80)	(-50; 100)		
	
Week 12					
N	100	23	123		
Mean (SD)	18.9 (29.33)	25.2 (27.45)	20.1 (28.99)		
Median	10.0	20.0	10.0		
Range	(-50; 100)	(-20; 80)	(-50; 100)		
	
Week 16					
N	96	24	120		
Mean (SD)	1.5 (27.61)	21.3 (30.26)	5.4 (29.13)		
Median	0.0	5.0	0.0		
Range	(-70; 80)	(-10; 80)	(-70; 80)		
	
Week 20					
N		26	26		
Mean (SD)		21.2 (27.47)	21.2 (27.47)		
Median		15.0	15.0		
Range		(-20; 80)	(-20; 80)		
	
Week 24					
N	95	24	119		
Mean (SD)	-6.2 (26.54)	20.0 (33.88)	-0.9 (29.94)		
Median	0.0	10.0	0.0		
Range	(-90; 80)	(-40; 80)	(-90; 80)		
	
Week 36					
N	8		8		
Mean (SD)	0.0 (32.95)		0.0 (32.95)		
Median	0.0		0.0		
Range	(-70; 50)		(-70; 50)		
	

Subjects with planned end of treatment at Week 12 do not have EQ-5Q, CES-D, FSS or WPAI results at Week 20.
The WPAI Daily Activities Impairment Score ranges from 0 to 100, with higher scores indicating worse outcome.
The WPAI Daily Activities Impairment Score is derived from impact on daily activities (question 6 from theWPAI questionnaire)	
[TPROWP03A.rtf] [\STAT\Analyses\Programs\Primary Analysis\Final4\2.TLF\7.PRO_PA\PRO_PA.sas] 15JAN2015, 16:51	

TPROWP03A:	Descriptive Statistics of the Change from Baseline in WPAI Daily Activities Impairment Score per Analysis Timepoint  Available Data Approach; Intent-to-Treat (Study TMC435HPC3014)
Treatment Group = Simeprevir 12Wks 150 mg PR12/24 
Phase = Overall Study Period 
2) By SVR12	
	SVR12 No	SVR12 Yes		
	12 Weeks 
Treatment	All Subjects	12 Weeks 
Treatment	All Subjects		
Week 4						
N	37	37	66	66		
Mean (SD)	15.1 (22.06)	15.1 (22.06)	16.4 (27.32)	16.4 (27.32)		
Median	10.0	10.0	10.0	10.0		
Range	(-50; 70)	(-50; 70)	(-30; 90)	(-30; 90)		
	
Week 8						
N	35	35	65	65		
Mean (SD)	19.7 (21.21)	19.7 (21.21)	20.2 (30.85)	20.2 (30.85)		
Median	20.0	20.0	20.0	20.0		
Range	(-10; 80)	(-10; 80)	(-50; 100)	(-50; 100)		
	
Week 12						
N	34	34	66	66		
Mean (SD)	22.9 (26.23)	22.9 (26.23)	16.8 (30.79)	16.8 (30.79)		
Median	20.0	20.0	10.0	10.0		
Range	(-20; 70)	(-20; 70)	(-50; 100)	(-50; 100)		
	
Week 16						
N	36	36	60	60		
Mean (SD)	3.1 (22.91)	3.1 (22.91)	0.5 (30.22)	0.5 (30.22)		
Median	0.0	0.0	0.0	0.0		
Range	(-50; 80)	(-50; 80)	(-70; 80)	(-70; 80)		
	
Week 24						
N	32	32	63	63		
Mean (SD)	-3.1 (23.89)	-3.1 (23.89)	-7.8 (27.85)	-7.8 (27.85)		
Median	0.0	0.0	0.0	0.0		
Range	(-60; 80)	(-60; 80)	(-90; 50)	(-90; 50)		
	
Week 36						
N	3	3	5	5		
Mean (SD)	20.0 (26.46)	20.0 (26.46)	-12.0 (32.71)	-12.0 (32.71)		
Median	10.0	10.0	0.0	0.0		
Range	(0; 50)	(0; 50)	(-70; 10)	(-70; 10)		
	

Subjects with planned end of treatment at Week 12 do not have EQ-5Q, CES-D, FSS or WPAI results at Week 20.
The WPAI Daily Activities Impairment Score ranges from 0 to 100, with higher scores indicating worse outcome.
The WPAI Daily Activities Impairment Score is derived from impact on daily activities (question 6 from theWPAI questionnaire)	
[TPROWP03A.rtf] [\STAT\Analyses\Programs\Primary Analysis\Final4\2.TLF\7.PRO_PA\PRO_PA.sas] 15JAN2015, 16:51	

TPROWP03A:	Descriptive Statistics of the Change from Baseline in WPAI Daily Activities Impairment Score per Analysis Timepoint  Available Data Approach; Intent-to-Treat (Study TMC435HPC3014)
Treatment Group = Simeprevir 12Wks 150 mg PR12/24 
Phase = Overall Study Period 
3) By Region	
	Europe		
	12 Weeks 
Treatment	>12 Weeks 
Treatment	All Subjects		
Week 4					
N	103	33	136		
Mean (SD)	15.9 (25.45)	21.8 (30.77)	17.4 (26.84)		
Median	10.0	10.0	10.0		
Range	(-50; 90)	(-40; 80)	(-50; 90)		
	
Week 8					
N	100	30	130		
Mean (SD)	20.0 (27.74)	20.0 (30.29)	20.0 (28.23)		
Median	20.0	10.0	15.0		
Range	(-50; 100)	(-40; 80)	(-50; 100)		
	
Week 12					
N	100	23	123		
Mean (SD)	18.9 (29.33)	25.2 (27.45)	20.1 (28.99)		
Median	10.0	20.0	10.0		
Range	(-50; 100)	(-20; 80)	(-50; 100)		
	
Week 16					
N	96	24	120		
Mean (SD)	1.5 (27.61)	21.3 (30.26)	5.4 (29.13)		
Median	0.0	5.0	0.0		
Range	(-70; 80)	(-10; 80)	(-70; 80)		
	
Week 20					
N		26	26		
Mean (SD)		21.2 (27.47)	21.2 (27.47)		
Median		15.0	15.0		
Range		(-20; 80)	(-20; 80)		
	
Week 24					
N	95	24	119		
Mean (SD)	-6.2 (26.54)	20.0 (33.88)	-0.9 (29.94)		
Median	0.0	10.0	0.0		
Range	(-90; 80)	(-40; 80)	(-90; 80)		
	
Week 36					
N	8		8		
Mean (SD)	0.0 (32.95)		0.0 (32.95)		
Median	0.0		0.0		
Range	(-70; 50)		(-70; 50)		
	

Subjects with planned end of treatment at Week 12 do not have EQ-5Q, CES-D, FSS or WPAI results at Week 20.
The WPAI Daily Activities Impairment Score ranges from 0 to 100, with higher scores indicating worse outcome.
The WPAI Daily Activities Impairment Score is derived from impact on daily activities (question 6 from theWPAI questionnaire)	
[TPROWP03A.rtf] [\STAT\Analyses\Programs\Primary Analysis\Final4\2.TLF\7.PRO_PA\PRO_PA.sas] 15JAN2015, 16:51	
