# Supplementary material for: An Open-Label Trial of 12-Week Simeprevir plus Peginterferon/Ribavirin (PR) in Treatment-Naïve Patients with Hepatitis C Virus (HCV) Genotype 1 (GT1)
Source: PLoS One. 2016 Jul 18;11(7):e0158526. doi: 10.1371/journal.pone.0158526 (PMC4948848; doi:10.1371/journal.pone.0158526)
Supplement: S1 Dataset — (ZIP) [file pone.0158526.s009.zip › Regression analyses/QCTEFSVR12MLRLRUM.RTF]

TMC435HPC3014 IA4: Multivariate Logistic Regression
Outcome=SVR12 (Population=Genotype 1 - 12Wks)

	Univariate Analysis	Initial Multivariate Analysis
Events/Total = 79/120	Final Multivariate Analysis
Events/Total = 80/122
C Index = 0.831	
Factor	N Obs
Used	Odds Ratio
(95% CI)	Wald
P-value	Odds Ratio
(95% CI)	Wald
P-value	Odds Ratio
(95% CI)	Wald
P-value	
BL Log10 HCV RNA  (IU/mL)	123	0.58 (0.33,1.04)	0.0665	0.36 (0.14,0.95)	0.0386	0.30 (0.13,0.68)	0.0038	
BL Q80K Mutation=Y	119	99E4 (0.00, I)	0.9791		.		.	
Baseline Albumin (g/L)	123	1.10 (0.97,1.26)	0.1405	1.12 (0.93,1.34)	0.2287		.	
Baseline BMI (kg/m²)	123	0.94 (0.86,1.02)	0.1574	0.93 (0.83,1.04)	0.1948		.	
Baseline Hemoglobin (g/L)	123	1.00 (0.97,1.03)	0.8589	1.00 (0.96,1.05)	0.9221		.	
Baseline Platelets (x10E9/L)	122	1.00 (0.99,1.01)	0.8522	1.00 (0.99,1.01)	0.3908		.	
HCV Subtype=1b	123	1.21 (0.57,2.58)	0.6225	1.83 (0.66,5.10)	0.2455		.	
IL28B=CC	123	11.8 (2.65,52.2)	0.0012	24.6 (4.33, 140)	0.0003	26.3 (4.96, 139)	0.0001	
Metavir Fibrosis Score=F0-F1	122	4.70 (1.95,11.4)	0.0006	6.65 (2.08,21.2)	0.0014	7.02 (2.33,21.2)	0.0005	
Race=white, missing=other	123	0.54 (0.20,1.49)	0.2352	0.47 (0.13,1.74)	0.2566		.	
Sex=F	123	0.97 (0.46,2.05)	0.9407		.		.	
Wk2 Viral Response=undetectable	122	2.38 (1.07,5.29)	0.0339	2.28 (0.77,6.70)	0.1355		.	
