# Supplementary material for: An Open-Label Trial of 12-Week Simeprevir plus Peginterferon/Ribavirin (PR) in Treatment-Naïve Patients with Hepatitis C Virus (HCV) Genotype 1 (GT1)
Source: PLoS One. 2016 Jul 18;11(7):e0158526. doi: 10.1371/journal.pone.0158526 (PMC4948848; doi:10.1371/journal.pone.0158526)
Supplement: S1 Dataset — (ZIP) [file pone.0158526.s009.zip › Regression analyses/QCTEFSVR12MLRnonCCLRUM.RTF]

TMC435HPC3014 IA4: Multivariate Logistic Regression
Outcome=SVR12 (Population=Genotype 1 - 12Wks - IL28B=CT/TT)

	Univariate Analysis	Initial Multivariate Analysis
Events/Total = 51/90	Final Multivariate Analysis
Events/Total = 51/91
C Index = 0.783	
Factor	N Obs
Used	Odds Ratio
(95% CI)	Wald
P-value	Odds Ratio
(95% CI)	Wald
P-value	Odds Ratio
(95% CI)	Wald
P-value	
BL Log10 HCV RNA  (IU/mL)	91	0.24 (0.10,0.55)	0.0008	0.25 (0.08,0.73)	0.0111	0.21 (0.09,0.53)	0.0009	
Baseline Albumin (g/L)	91	1.09 (0.93,1.26)	0.2833	1.12 (0.92,1.37)	0.2461		.	
Baseline BMI (kg/m²)	91	0.96 (0.88,1.05)	0.3999	0.93 (0.83,1.04)	0.2184		.	
Baseline Hemoglobin (g/L)	91	0.99 (0.96,1.02)	0.6036	1.00 (0.96,1.05)	0.9852		.	
Baseline Platelets (x10E9/L)	90	1.00 (1.00,1.01)	0.4876	1.00 (0.99,1.01)	0.7905		.	
HCV Subtype=1b	91	2.39 (0.99,5.80)	0.0537	2.28 (0.74,7.02)	0.1504		.	
Metavir Fibrosis Score=F0-F1	91	6.80 (2.23,20.8)	0.0008	7.91 (2.07,30.2)	0.0025	7.99 (2.31,27.6)	0.0010	
Race=white, missing=other	91	0.82 (0.27,2.54)	0.7358	0.63 (0.15,2.57)	0.5151		.	
Sex=F	91	0.98 (0.43,2.25)	0.9666		.		.	
Wk2 Viral Response=undetectable	91	2.97 (1.22,7.19)	0.0161	2.54 (0.80,8.03)	0.1129		.	
