# Supplementary material for: An Open-Label Trial of 12-Week Simeprevir plus Peginterferon/Ribavirin (PR) in Treatment-Naïve Patients with Hepatitis C Virus (HCV) Genotype 1 (GT1)
Source: PLoS One. 2016 Jul 18;11(7):e0158526. doi: 10.1371/journal.pone.0158526 (PMC4948848; doi:10.1371/journal.pone.0158526)
Supplement: S1 Dataset — (ZIP) [file pone.0158526.s009.zip › Regression analyses/QCTEFVRLMLRLRUM.RTF]

TMC435HPC3014 IA4: Multivariate Logistic Regression
Outcome=Relapse (Population=Genotype 1 - 12Wks)

	Univariate Analysis	Initial Multivariate Analysis
Events/Total = 40/119	Final Multivariate Analysis
Events/Total = 41/121
C Index = 0.819	
Factor	N Obs
Used	Odds Ratio
(95% CI)	Wald
P-value	Odds Ratio
(95% CI)	Wald
P-value	Odds Ratio
(95% CI)	Wald
P-value	
BL Log10 HCV RNA  (IU/mL)	122	2.06 (1.12,3.80)	0.0208	4.05 (1.51,10.9)	0.0055	4.40 (1.87,10.4)	0.0007	
BL Q80K Mutation=Y	118	0.00 (0.00,****)	0.9683		.		.	
Baseline Albumin (g/L)	122	0.92 (0.80,1.05)	0.2080	0.87 (0.73,1.04)	0.1264		.	
Baseline BMI (kg/m²)	122	1.01 (0.93,1.10)	0.7546	0.97 (0.87,1.09)	0.6165		.	
Baseline Hemoglobin (g/L)	122	1.01 (0.98,1.05)	0.3604	1.03 (0.97,1.09)	0.3796		.	
Baseline Platelets (x10E9/L)	121	1.00 (1.00,1.01)	0.5504	1.00 (0.99,1.01)	0.5994		.	
HCV Subtype=1b	122	0.88 (0.41,1.88)	0.7333	0.75 (0.27,2.04)	0.5697		.	
IL28B=CC	122	0.09 (0.02,0.39)	0.0013	0.04 (0.01,0.23)	0.0003	0.04 (0.01,0.20)	<.0001	
Metavir Fibrosis Score=F0-F1	121	0.30 (0.13,0.72)	0.0068	0.24 (0.08,0.76)	0.0151	0.23 (0.08,0.66)	0.0065	
Race=white, missing=other	122	1.39 (0.53,3.65)	0.5069	1.23 (0.35,4.31)	0.7511		.	
Sex=F	122	0.93 (0.44,1.97)	0.8503	1.45 (0.34,6.23)	0.6135		.	
Wk2 Viral Response=undetectable	121	0.46 (0.20,1.02)	0.0563	0.54 (0.19,1.55)	0.2519		.	
