# Supplementary material for: An Open-Label Trial of 12-Week Simeprevir plus Peginterferon/Ribavirin (PR) in Treatment-Naïve Patients with Hepatitis C Virus (HCV) Genotype 1 (GT1)
Source: PLoS One. 2016 Jul 18;11(7):e0158526. doi: 10.1371/journal.pone.0158526 (PMC4948848; doi:10.1371/journal.pone.0158526)
Supplement: S1 Dataset — (ZIP) [file pone.0158526.s009.zip › Regression analyses/QCTEFVRLMLRnonCCLRUM.RTF]

TMC435HPC3014 IA4: Multivariate Logistic Regression
Outcome=Relapse (Population=Genotype 1 - 12Wks - IL28B=CT/TT)

	Univariate Analysis	Initial Multivariate Analysis
Events/Total = 38/89	Final Multivariate Analysis
Events/Total = 39/90
C Index = 0.769	
Factor	N Obs
Used	Odds Ratio
(95% CI)	Wald
P-value	Odds Ratio
(95% CI)	Wald
P-value	Odds Ratio
(95% CI)	Wald
P-value	
BL Log10 HCV RNA  (IU/mL)	90	4.55 (1.92,10.8)	0.0006	4.39 (1.57,12.3)	0.0048	4.72 (1.93,11.5)	0.0007	
Baseline Albumin (g/L)	90	0.94 (0.81,1.09)	0.4123	0.89 (0.74,1.07)	0.2291		.	
Baseline BMI (kg/m²)	90	0.99 (0.90,1.08)	0.8050	0.97 (0.86,1.09)	0.6192		.	
Baseline Hemoglobin (g/L)	90	1.01 (0.98,1.05)	0.4688	1.02 (0.96,1.09)	0.4537		.	
Baseline Platelets (x10E9/L)	89	1.00 (0.99,1.01)	0.7646	1.00 (0.99,1.01)	0.6145		.	
HCV Subtype=1b	90	0.54 (0.22,1.32)	0.1780	0.77 (0.26,2.25)	0.6331		.	
Metavir Fibrosis Score=F0-F1	90	0.25 (0.09,0.71)	0.0089	0.24 (0.07,0.81)	0.0217	0.24 (0.08,0.74)	0.0127	
Race=white, missing=other	90	0.85 (0.28,2.59)	0.7755	0.84 (0.21,3.29)	0.8013		.	
Sex=F	90	1.07 (0.46,2.46)	0.8759	1.81 (0.38,8.53)	0.4551		.	
Wk2 Viral Response=undetectable	90	0.38 (0.16,0.92)	0.0316	0.50 (0.17,1.49)	0.2132		.	
