# Supplementary material for: An Open-Label Trial of 12-Week Simeprevir plus Peginterferon/Ribavirin (PR) in Treatment-Naïve Patients with Hepatitis C Virus (HCV) Genotype 1 (GT1)
Source: PLoS One. 2016 Jul 18;11(7):e0158526. doi: 10.1371/journal.pone.0158526 (PMC4948848; doi:10.1371/journal.pone.0158526)
Supplement: S1 Dataset — (ZIP) [file pone.0158526.s009.zip › Safety data/tsfae01tdg112.rtf]

TSFAE01TDG112:	Adverse Event Summary Table; Intent-to-treat (Study TMC435HPC3014) HCVGTGR1='Genotype 1' and planeot='12 Wks'	
	Simeprevir
12 Wks
150 mg
PR 12/24 	
	SMV + PR 	Ent Trt 	PR Only 	Follow-Up 	Overall 	
Analysis set: intent-to-treat	123	123	2	122	123	
						
Any AE	117 (95.1%)	117 (95.1%)		31 (25.4%)	117 (95.1%)	
Any SAE	4 (3.3%)	4 (3.3%)		3 (2.5%)	7 (5.7%)	
At least possibly related to any Study Therapy	110 (89.4%)	110 (89.4%)		7 (5.7%)	111 (90.2%)	
At least possibly related to SMV	68 (55.3%)	68 (55.3%)			68 (55.3%)	
At least possibly related to Ribavirin	84 (68.3%)	84 (68.3%)		3 (2.5%)	85 (69.1%)	
At least possibly related to PegIFN	105 (85.4%)	105 (85.4%)		5 (4.1%)	106 (86.2%)	
Worst grade 1 AE	54 (43.9%)	54 (43.9%)		18 (14.8%)	49 (39.8%)	
Worst grade 2 AE	37 (30.1%)	37 (30.1%)		9 (7.4%)	39 (31.7%)	
Worst grade 3 AE	23 (18.7%)	23 (18.7%)		2 (1.6%)	24 (19.5%)	
Worst grade 4 AE	3 (2.4%)	3 (2.4%)		2 (1.6%)	5 (4.1%)	
Worst grade 1 or 2 AE	91 (74.0%)	91 (74.0%)		27 (22.1%)	88 (71.5%)	
Worst grade 3 or 4 AE	26 (21.1%)	26 (21.1%)		4 (3.3%)	29 (23.6%)	
At least possibly related to SMV	6 (4.9%)	6 (4.9%)			6 (4.9%)	
	
[TSFAE01TDG112.RTF] [TMC435\HPC3014\DBR_FINAL_ANALYSIS\RE_FINAL_ANALYSIS\PROD\TSFAE01TD.SAS] 02NOV2015, 11:23	
