# Supplementary material for: An Open-Label Trial of 12-Week Simeprevir plus Peginterferon/Ribavirin (PR) in Treatment-Naïve Patients with Hepatitis C Virus (HCV) Genotype 1 (GT1)
Source: PLoS One. 2016 Jul 18;11(7):e0158526. doi: 10.1371/journal.pone.0158526 (PMC4948848; doi:10.1371/journal.pone.0158526)
Supplement: S1 Dataset — (ZIP) [file pone.0158526.s009.zip › Safety data/tsfae01tdg1all.rtf]

TSFAE01TDG1ALL:	Adverse Event Summary Table; Intent-to-treat (Study TMC435HPC3014) HCVGTGR1='Genotype 1'	
	Simeprevir
12 Wks
150 mg
PR 12/24 	
	SMV + PR 	Ent Trt 	PR Only 	Follow-Up 	Overall 	
Analysis set: intent-to-treat	163	163	32	160	163	
						
Any AE	154 (94.5%)	154 (94.5%)	20 (62.5%)	38 (23.8%)	154 (94.5%)	
Any SAE	4 (2.5%)	6 (3.7%)	2 (6.3%)	4 (2.5%)	10 (6.1%)	
At least possibly related to SMV		1 (0.6%)	1 (3.1%)		1 (0.6%)	
At least possibly related to any Study Therapy	146 (89.6%)	146 (89.6%)	19 (59.4%)	8 (5.0%)	147 (90.2%)	
At least possibly related to SMV	87 (53.4%)	88 (54.0%)	4 (12.5%)		88 (54.0%)	
At least possibly related to Ribavirin	110 (67.5%)	114 (69.9%)	13 (40.6%)	3 (1.9%)	115 (70.6%)	
At least possibly related to PegIFN	138 (84.7%)	138 (84.7%)	12 (37.5%)	6 (3.8%)	139 (85.3%)	
Worst grade 1 AE	70 (42.9%)	67 (41.1%)	11 (34.4%)	22 (13.8%)	61 (37.4%)	
Worst grade 2 AE	52 (31.9%)	53 (32.5%)	7 (21.9%)	9 (5.6%)	54 (33.1%)	
Worst grade 3 AE	26 (16.0%)	28 (17.2%)	2 (6.3%)	4 (2.5%)	31 (19.0%)	
Worst grade 4 AE	6 (3.7%)	6 (3.7%)		3 (1.9%)	8 (4.9%)	
Worst grade 1 or 2 AE	122 (74.8%)	120 (73.6%)	18 (56.3%)	31 (19.4%)	115 (70.6%)	
Worst grade 3 or 4 AE	32 (19.6%)	34 (20.9%)	2 (6.3%)	7 (4.4%)	39 (23.9%)	
At least possibly related to SMV	7 (4.3%)	8 (4.9%)	1 (3.1%)		8 (4.9%)	
AE leading to permanent stop(a)	3 (1.8%)	4 (2.5%)	1 (3.1%)		4 (2.5%)	
SMV(b)	3 (1.8%)	3 (1.8%)			3 (1.8%)	
SMV, PegIFN and RBV	3 (1.8%)	3 (1.8%)			3 (1.8%)	
PegIFN or RBV		1 (0.6%)	1 (3.1%)		1 (0.6%)	
PegIFN and RBV		1 (0.6%)	1 (3.1%)		1 (0.6%)	
	
[TSFAE01TDG1ALL.RTF] [TMC435\HPC3014\DBR_FINAL_ANALYSIS\RE_FINAL_ANALYSIS\PROD\TSFAE01TD.SAS] 02NOV2015, 11:23	
