# Supplementary material for: An Open-Label Trial of 12-Week Simeprevir plus Peginterferon/Ribavirin (PR) in Treatment-Naïve Patients with Hepatitis C Virus (HCV) Genotype 1 (GT1)
Source: PLoS One. 2016 Jul 18;11(7):e0158526. doi: 10.1371/journal.pone.0158526 (PMC4948848; doi:10.1371/journal.pone.0158526)
Supplement: S1 Dataset — (ZIP) [file pone.0158526.s009.zip › Safety data/tsfae01tdg1gt12.rtf]

TSFAE01TDG1GT12:	Adverse Event Summary Table; Intent-to-treat (Study TMC435HPC3014) HCVGTGR1='Genotype 1' and (planeot='24 Wks' or planeot='48 Wks')	
	Simeprevir
12 Wks
150 mg
PR 12/24 	
	SMV + PR 	Ent Trt 	PR Only 	Follow-Up 	Overall 	
Analysis set: intent-to-treat	40	40	30	38	40	
						
Any AE	37 (92.5%)	37 (92.5%)	20 (66.7%)	7 (18.4%)	37 (92.5%)	
Any SAE		2 (5.0%)	2 (6.7%)	1 (2.6%)	3 (7.5%)	
At least possibly related to SMV		1 (2.5%)	1 (3.3%)		1 (2.5%)	
At least possibly related to any Study Therapy	36 (90.0%)	36 (90.0%)	19 (63.3%)	1 (2.6%)	36 (90.0%)	
At least possibly related to SMV	19 (47.5%)	20 (50.0%)	4 (13.3%)		20 (50.0%)	
At least possibly related to Ribavirin	26 (65.0%)	30 (75.0%)	13 (43.3%)		30 (75.0%)	
At least possibly related to PegIFN	33 (82.5%)	33 (82.5%)	12 (40.0%)	1 (2.6%)	33 (82.5%)	
Worst grade 1 AE	16 (40.0%)	13 (32.5%)	11 (36.7%)	4 (10.5%)	12 (30.0%)	
Worst grade 2 AE	15 (37.5%)	16 (40.0%)	7 (23.3%)		15 (37.5%)	
Worst grade 3 AE	3 (7.5%)	5 (12.5%)	2 (6.7%)	2 (5.3%)	7 (17.5%)	
Worst grade 4 AE	3 (7.5%)	3 (7.5%)		1 (2.6%)	3 (7.5%)	
Worst grade 1 or 2 AE	31 (77.5%)	29 (72.5%)	18 (60.0%)	4 (10.5%)	27 (67.5%)	
Worst grade 3 or 4 AE	6 (15.0%)	8 (20.0%)	2 (6.7%)	3 (7.9%)	10 (25.0%)	
At least possibly related to SMV	1 (2.5%)	2 (5.0%)	1 (3.3%)		2 (5.0%)	
AE leading to permanent stop(a)	3 (7.5%)	4 (10.0%)	1 (3.3%)		4 (10.0%)	
SMV(b)	3 (7.5%)	3 (7.5%)			3 (7.5%)	
SMV, PegIFN and RBV	3 (7.5%)	3 (7.5%)			3 (7.5%)	
PegIFN or RBV		1 (2.5%)	1 (3.3%)		1 (2.5%)	
PegIFN and RBV		1 (2.5%)	1 (3.3%)		1 (2.5%)	
	
[TSFAE01TDG1GT12.RTF] [TMC435\HPC3014\DBR_FINAL_ANALYSIS\RE_FINAL_ANALYSIS\PROD\TSFAE01TD.SAS] 02NOV2015, 11:23	
