# Supplementary material for: An Open-Label Trial of 12-Week Simeprevir plus Peginterferon/Ribavirin (PR) in Treatment-Naïve Patients with Hepatitis C Virus (HCV) Genotype 1 (GT1)
Source: PLoS One. 2016 Jul 18;11(7):e0158526. doi: 10.1371/journal.pone.0158526 (PMC4948848; doi:10.1371/journal.pone.0158526)
Supplement: S1 Dataset — (ZIP) [file pone.0158526.s009.zip › Safety data/tsfae02tdg112.rtf]

TSFAE02TDG112:	Number (pcnt) of Genotype 1 Subjects with Adverse Events, Intent-to-treat, Study TMC435HPC3014 Trt Dur 12 Wks	
	Simeprevir
12 Wks
150 mg
PR 12/24 	
	SMV + PR 	Ent Trt 	PR Only 	Follow-Up 	Overall 	
Analysis set: Intent-to-treat	123	123	2	122	123	
Any AE	117 (95.1%)	117 (95.1%)	0	31 (25.4%)	117 (95.1%)	
General disorders and administration site conditions	91 (74.0%)	91 (74.0%)	0	1 (0.8%)	91 (74.0%)	
Influenza like illness	47 (38.2%)	47 (38.2%)	0	0	47 (38.2%)	
Fatigue	38 (30.9%)	38 (30.9%)	0	0	38 (30.9%)	
Asthenia	29 (23.6%)	29 (23.6%)	0	0	29 (23.6%)	
Pyrexia	12 (9.8%)	12 (9.8%)	0	0	12 (9.8%)	
Irritability	9 (7.3%)	9 (7.3%)	0	0	9 (7.3%)	
Injection site erythema	5 (4.1%)	5 (4.1%)	0	0	5 (4.1%)	
Chest discomfort	2 (1.6%)	2 (1.6%)	0	0	2 (1.6%)	
Feeling cold	2 (1.6%)	2 (1.6%)	0	0	2 (1.6%)	
Injection site haematoma	1 (0.8%)	1 (0.8%)	0	0	1 (0.8%)	
Injection site pain	1 (0.8%)	1 (0.8%)	0	0	1 (0.8%)	
Injection site rash	1 (0.8%)	1 (0.8%)	0	0	1 (0.8%)	
Malaise	1 (0.8%)	1 (0.8%)	0	0	1 (0.8%)	
Mucosal dryness	1 (0.8%)	1 (0.8%)	0	0	1 (0.8%)	
Mucosal inflammation	1 (0.8%)	1 (0.8%)	0	0	1 (0.8%)	
Non-cardiac chest pain	1 (0.8%)	1 (0.8%)	0	1 (0.8%)	2 (1.6%)	
Oedema	1 (0.8%)	1 (0.8%)	0	0	1 (0.8%)	
Pain	1 (0.8%)	1 (0.8%)	0	0	1 (0.8%)	
Skin and subcutaneous tissue disorders	68 (55.3%)	69 (56.1%)	0	14 (11.5%)	73 (59.3%)	
Pruritus	41 (33.3%)	43 (35.0%)	0	4 (3.3%)	46 (37.4%)	
Dry skin	23 (18.7%)	24 (19.5%)	0	1 (0.8%)	25 (20.3%)	
Rash	17 (13.8%)	17 (13.8%)	0	3 (2.5%)	19 (15.4%)	
Alopecia	5 (4.1%)	9 (7.3%)	0	5 (4.1%)	14 (11.4%)	
Erythema	3 (2.4%)	3 (2.4%)	0	0	3 (2.4%)	
Eczema	2 (1.6%)	2 (1.6%)	0	0	2 (1.6%)	
Hyperhidrosis	2 (1.6%)	2 (1.6%)	0	0	2 (1.6%)	
Psoriasis	2 (1.6%)	2 (1.6%)	0	1 (0.8%)	3 (2.4%)	
Dermatitis	1 (0.8%)	1 (0.8%)	0	0	1 (0.8%)	
Dermatosis	1 (0.8%)	1 (0.8%)	0	0	1 (0.8%)	
Generalised erythema	1 (0.8%)	1 (0.8%)	0	0	1 (0.8%)	
Nail discolouration	1 (0.8%)	1 (0.8%)	0	0	1 (0.8%)	
Pruritus generalised	1 (0.8%)	1 (0.8%)	0	0	1 (0.8%)	
Rash macular	1 (0.8%)	1 (0.8%)	0	0	1 (0.8%)	
Rash pruritic	1 (0.8%)	1 (0.8%)	0	0	1 (0.8%)	
Skin discolouration	1 (0.8%)	1 (0.8%)	0	0	1 (0.8%)	
Skin fissures	1 (0.8%)	1 (0.8%)	0	0	1 (0.8%)	
Skin irritation	1 (0.8%)	1 (0.8%)	0	0	1 (0.8%)	
Skin mass	1 (0.8%)	1 (0.8%)	0	0	1 (0.8%)	
Solar dermatitis	1 (0.8%)	1 (0.8%)	0	0	1 (0.8%)	
Rosacea	0	0	0	1 (0.8%)	1 (0.8%)	
Gastrointestinal disorders	49 (39.8%)	49 (39.8%)	0	0	49 (39.8%)	
Nausea	15 (12.2%)	15 (12.2%)	0	0	15 (12.2%)	
Diarrhoea	9 (7.3%)	9 (7.3%)	0	0	9 (7.3%)	
Abdominal pain upper	6 (4.9%)	7 (5.7%)	0	0	7 (5.7%)	
Dry mouth	7 (5.7%)	7 (5.7%)	0	0	7 (5.7%)	
Constipation	5 (4.1%)	5 (4.1%)	0	0	5 (4.1%)	
Dyspepsia	5 (4.1%)	5 (4.1%)	0	0	5 (4.1%)	
Abdominal pain	4 (3.3%)	4 (3.3%)	0	0	4 (3.3%)	
Gastrooesophageal reflux disease	4 (3.3%)	4 (3.3%)	0	0	4 (3.3%)	
Vomiting	4 (3.3%)	4 (3.3%)	0	0	4 (3.3%)	
Anal pruritus	2 (1.6%)	2 (1.6%)	0	0	2 (1.6%)	
Dysphagia	2 (1.6%)	2 (1.6%)	0	0	2 (1.6%)	
Gingival bleeding	2 (1.6%)	2 (1.6%)	0	0	2 (1.6%)	
Mouth ulceration	2 (1.6%)	2 (1.6%)	0	0	2 (1.6%)	
Toothache	2 (1.6%)	2 (1.6%)	0	0	2 (1.6%)	
Abdominal discomfort	1 (0.8%)	1 (0.8%)	0	0	1 (0.8%)	
Abdominal distension	1 (0.8%)	1 (0.8%)	0	0	1 (0.8%)	
Cheilitis	1 (0.8%)	1 (0.8%)	0	0	1 (0.8%)	
Gastric ulcer	1 (0.8%)	1 (0.8%)	0	0	1 (0.8%)	
Gingival pain	1 (0.8%)	1 (0.8%)	0	0	1 (0.8%)	
Haemorrhoids	0	1 (0.8%)	0	0	1 (0.8%)	
Tongue ulceration	1 (0.8%)	1 (0.8%)	0	0	1 (0.8%)	
Nervous system disorders	43 (35.0%)	45 (36.6%)	0	5 (4.1%)	46 (37.4%)	
Headache	30 (24.4%)	32 (26.0%)	0	2 (1.6%)	33 (26.8%)	
Dysgeusia	8 (6.5%)	8 (6.5%)	0	0	8 (6.5%)	
Dizziness	7 (5.7%)	7 (5.7%)	0	1 (0.8%)	8 (6.5%)	
Disturbance in attention	4 (3.3%)	4 (3.3%)	0	0	4 (3.3%)	
Sciatica	1 (0.8%)	2 (1.6%)	0	1 (0.8%)	3 (2.4%)	
Ageusia	1 (0.8%)	1 (0.8%)	0	0	1 (0.8%)	
Hyperaesthesia	1 (0.8%)	1 (0.8%)	0	0	1 (0.8%)	
Memory impairment	1 (0.8%)	1 (0.8%)	0	0	1 (0.8%)	
Migraine	1 (0.8%)	1 (0.8%)	0	0	1 (0.8%)	
Paraesthesia	1 (0.8%)	1 (0.8%)	0	0	1 (0.8%)	
Poor quality sleep	1 (0.8%)	1 (0.8%)	0	0	1 (0.8%)	
Presyncope	1 (0.8%)	1 (0.8%)	0	0	1 (0.8%)	
Somnolence	1 (0.8%)	1 (0.8%)	0	0	1 (0.8%)	
Carpal tunnel syndrome	0	0	0	1 (0.8%)	1 (0.8%)	
Dizziness postural	0	0	0	1 (0.8%)	1 (0.8%)	
Neuralgia	0	0	0	1 (0.8%)	1 (0.8%)	
Tremor	0	0	0	1 (0.8%)	1 (0.8%)	
Psychiatric disorders	44 (35.8%)	45 (36.6%)	0	4 (3.3%)	48 (39.0%)	
Insomnia	21 (17.1%)	22 (17.9%)	0	0	22 (17.9%)	
Depression	10 (8.1%)	10 (8.1%)	0	2 (1.6%)	12 (9.8%)	
Sleep disorder	10 (8.1%)	10 (8.1%)	0	0	10 (8.1%)	
Anxiety	4 (3.3%)	4 (3.3%)	0	0	4 (3.3%)	
Depressed mood	3 (2.4%)	3 (2.4%)	0	0	3 (2.4%)	
Mood swings	3 (2.4%)	3 (2.4%)	0	0	3 (2.4%)	
Affect lability	2 (1.6%)	2 (1.6%)	0	0	2 (1.6%)	
Aggression	2 (1.6%)	2 (1.6%)	0	0	2 (1.6%)	
Emotional disorder	2 (1.6%)	2 (1.6%)	0	0	2 (1.6%)	
Alcohol withdrawal syndrome	1 (0.8%)	1 (0.8%)	0	1 (0.8%)	1 (0.8%)	
Anger	1 (0.8%)	1 (0.8%)	0	0	1 (0.8%)	
Middle insomnia	1 (0.8%)	1 (0.8%)	0	0	1 (0.8%)	
Mood altered	1 (0.8%)	1 (0.8%)	0	0	1 (0.8%)	
Nicotine dependence	1 (0.8%)	1 (0.8%)	0	0	1 (0.8%)	
Stress	1 (0.8%)	1 (0.8%)	0	0	1 (0.8%)	
Psychotic disorder	0	0	0	1 (0.8%)	1 (0.8%)	
Schizophrenia, paranoid type	0	0	0	1 (0.8%)	1 (0.8%)	
Musculoskeletal and connective tissue disorders	35 (28.5%)	35 (28.5%)	0	3 (2.5%)	36 (29.3%)	
Arthralgia	15 (12.2%)	15 (12.2%)	0	1 (0.8%)	16 (13.0%)	
Myalgia	11 (8.9%)	11 (8.9%)	0	1 (0.8%)	12 (9.8%)	
Back pain	5 (4.1%)	5 (4.1%)	0	1 (0.8%)	6 (4.9%)	
Muscle spasms	3 (2.4%)	3 (2.4%)	0	0	3 (2.4%)	
Sensation of heaviness	1 (0.8%)	2 (1.6%)	0	0	2 (1.6%)	
Axillary mass	1 (0.8%)	1 (0.8%)	0	0	1 (0.8%)	
Muscle twitching	1 (0.8%)	1 (0.8%)	0	0	1 (0.8%)	
Musculoskeletal pain	1 (0.8%)	1 (0.8%)	0	1 (0.8%)	2 (1.6%)	
Neck pain	1 (0.8%)	1 (0.8%)	0	0	1 (0.8%)	
Pain in extremity	1 (0.8%)	1 (0.8%)	0	0	1 (0.8%)	
Polyarthritis	1 (0.8%)	1 (0.8%)	0	0	1 (0.8%)	
Rheumatic disorder	1 (0.8%)	1 (0.8%)	0	0	1 (0.8%)	
Blood and lymphatic system disorders	30 (24.4%)	30 (24.4%)	0	0	30 (24.4%)	
Neutropenia	24 (19.5%)	24 (19.5%)	0	0	24 (19.5%)	
Anaemia	12 (9.8%)	12 (9.8%)	0	0	12 (9.8%)	
Leukopenia	6 (4.9%)	6 (4.9%)	0	0	6 (4.9%)	
Thrombocytopenia	4 (3.3%)	4 (3.3%)	0	0	4 (3.3%)	
Lymphadenopathy	0	1 (0.8%)	0	0	1 (0.8%)	
Lymphopenia	1 (0.8%)	1 (0.8%)	0	0	1 (0.8%)	
Respiratory, thoracic and mediastinal disorders	27 (22.0%)	29 (23.6%)	0	2 (1.6%)	30 (24.4%)	
Dyspnoea	13 (10.6%)	13 (10.6%)	0	0	13 (10.6%)	
Cough	8 (6.5%)	8 (6.5%)	0	1 (0.8%)	9 (7.3%)	
Dyspnoea exertional	6 (4.9%)	6 (4.9%)	0	1 (0.8%)	7 (5.7%)	
Epistaxis	3 (2.4%)	3 (2.4%)	0	0	3 (2.4%)	
Asthma	1 (0.8%)	2 (1.6%)	0	0	2 (1.6%)	
Laryngeal ulceration	1 (0.8%)	1 (0.8%)	0	0	1 (0.8%)	
Oropharyngeal pain	1 (0.8%)	1 (0.8%)	0	0	1 (0.8%)	
Rhinitis allergic	0	1 (0.8%)	0	0	1 (0.8%)	
Metabolism and nutrition disorders	23 (18.7%)	23 (18.7%)	0	1 (0.8%)	24 (19.5%)	
Decreased appetite	18 (14.6%)	18 (14.6%)	0	0	18 (14.6%)	
Gout	1 (0.8%)	2 (1.6%)	0	0	2 (1.6%)	
Increased appetite	2 (1.6%)	2 (1.6%)	0	0	2 (1.6%)	
Hyperamylasaemia	1 (0.8%)	1 (0.8%)	0	0	1 (0.8%)	
Hyperlipasaemia	1 (0.8%)	1 (0.8%)	0	0	1 (0.8%)	
Hyperproteinaemia	0	0	0	1 (0.8%)	1 (0.8%)	
Infections and infestations	20 (16.3%)	22 (17.9%)	0	4 (3.3%)	25 (20.3%)	
Bronchitis	3 (2.4%)	3 (2.4%)	0	0	3 (2.4%)	
Influenza	3 (2.4%)	3 (2.4%)	0	0	3 (2.4%)	
Herpes simplex	2 (1.6%)	2 (1.6%)	0	0	2 (1.6%)	
Acarodermatitis	1 (0.8%)	1 (0.8%)	0	0	1 (0.8%)	
Acute tonsillitis	1 (0.8%)	1 (0.8%)	0	0	1 (0.8%)	
Furuncle	1 (0.8%)	1 (0.8%)	0	0	1 (0.8%)	
Gingivitis	1 (0.8%)	1 (0.8%)	0	0	1 (0.8%)	
Helicobacter gastritis	1 (0.8%)	1 (0.8%)	0	0	1 (0.8%)	
Mastitis	1 (0.8%)	1 (0.8%)	0	0	1 (0.8%)	
Nasopharyngitis	1 (0.8%)	1 (0.8%)	0	1 (0.8%)	2 (1.6%)	
Oral candidiasis	1 (0.8%)	1 (0.8%)	0	0	1 (0.8%)	
Otitis media	1 (0.8%)	1 (0.8%)	0	0	1 (0.8%)	
Pericoronitis	1 (0.8%)	1 (0.8%)	0	0	1 (0.8%)	
Respiratory tract infection	0	1 (0.8%)	0	0	1 (0.8%)	
Rhinitis	1 (0.8%)	1 (0.8%)	0	0	1 (0.8%)	
Sinusitis	1 (0.8%)	1 (0.8%)	0	0	1 (0.8%)	
Subcutaneous abscess	1 (0.8%)	1 (0.8%)	0	0	1 (0.8%)	
Tooth abscess	1 (0.8%)	1 (0.8%)	0	1 (0.8%)	2 (1.6%)	
Tracheitis	1 (0.8%)	1 (0.8%)	0	0	1 (0.8%)	
Urinary tract infection	0	1 (0.8%)	0	0	1 (0.8%)	
Pneumonia	0	0	0	1 (0.8%)	1 (0.8%)	
Tooth infection	0	0	0	1 (0.8%)	1 (0.8%)	
Investigations	18 (14.6%)	18 (14.6%)	0	2 (1.6%)	19 (15.4%)	
Blood bilirubin increased	7 (5.7%)	7 (5.7%)	0	0	7 (5.7%)	
Weight decreased	4 (3.3%)	4 (3.3%)	0	0	4 (3.3%)	
Alanine aminotransferase increased	2 (1.6%)	2 (1.6%)	0	0	2 (1.6%)	
Aspartate aminotransferase increased	2 (1.6%)	2 (1.6%)	0	0	2 (1.6%)	
Gamma-glutamyltransferase increased	2 (1.6%)	2 (1.6%)	0	0	2 (1.6%)	
Haemoglobin decreased	2 (1.6%)	2 (1.6%)	0	0	2 (1.6%)	
Blood creatine phosphokinase increased	1 (0.8%)	1 (0.8%)	0	1 (0.8%)	2 (1.6%)	
Blood lactate dehydrogenase increased	1 (0.8%)	1 (0.8%)	0	0	1 (0.8%)	
Body temperature increased	1 (0.8%)	1 (0.8%)	0	0	1 (0.8%)	
General physical condition abnormal	1 (0.8%)	1 (0.8%)	0	0	1 (0.8%)	
Neutrophil count decreased	1 (0.8%)	1 (0.8%)	0	0	1 (0.8%)	
Weight increased	1 (0.8%)	1 (0.8%)	0	0	1 (0.8%)	
Amylase increased	0	0	0	1 (0.8%)	1 (0.8%)	
Lipase increased	0	0	0	1 (0.8%)	1 (0.8%)	
Ear and labyrinth disorders	12 (9.8%)	12 (9.8%)	0	0	12 (9.8%)	
Vertigo	8 (6.5%)	8 (6.5%)	0	0	8 (6.5%)	
Tinnitus	4 (3.3%)	4 (3.3%)	0	0	4 (3.3%)	
Hypoacusis	1 (0.8%)	1 (0.8%)	0	0	1 (0.8%)	
Eye disorders	8 (6.5%)	9 (7.3%)	0	0	9 (7.3%)	
Dry eye	2 (1.6%)	2 (1.6%)	0	0	2 (1.6%)	
Chalazion	0	1 (0.8%)	0	0	1 (0.8%)	
Conjunctival irritation	1 (0.8%)	1 (0.8%)	0	0	1 (0.8%)	
Conjunctival ulcer	1 (0.8%)	1 (0.8%)	0	0	1 (0.8%)	
Eye irritation	1 (0.8%)	1 (0.8%)	0	0	1 (0.8%)	
Ocular hyperaemia	1 (0.8%)	1 (0.8%)	0	0	1 (0.8%)	
Vision blurred	1 (0.8%)	1 (0.8%)	0	0	1 (0.8%)	
Visual impairment	1 (0.8%)	1 (0.8%)	0	0	1 (0.8%)	
Reproductive system and breast disorders	7 (5.7%)	7 (5.7%)	0	1 (0.8%)	8 (6.5%)	
Menstrual disorder	1 (0.8%)	1 (0.8%)	0	0	1 (0.8%)	
Metrorrhagia	1 (0.8%)	1 (0.8%)	0	0	1 (0.8%)	
Pelvic discomfort	1 (0.8%)	1 (0.8%)	0	0	1 (0.8%)	
Testicular necrosis	1 (0.8%)	1 (0.8%)	0	0	1 (0.8%)	
Vaginal discharge	1 (0.8%)	1 (0.8%)	0	0	1 (0.8%)	
Vaginal haemorrhage	1 (0.8%)	1 (0.8%)	0	0	1 (0.8%)	
Vulvovaginal pruritus	1 (0.8%)	1 (0.8%)	0	0	1 (0.8%)	
Menorrhagia	0	0	0	1 (0.8%)	1 (0.8%)	
Cardiac disorders	5 (4.1%)	5 (4.1%)	0	0	5 (4.1%)	
Tachycardia	4 (3.3%)	4 (3.3%)	0	0	4 (3.3%)	
Palpitations	1 (0.8%)	1 (0.8%)	0	0	1 (0.8%)	
Renal and urinary disorders	5 (4.1%)	5 (4.1%)	0	0	5 (4.1%)	
Chromaturia	2 (1.6%)	2 (1.6%)	0	0	2 (1.6%)	
Cystitis-like symptom	1 (0.8%)	1 (0.8%)	0	0	1 (0.8%)	
Proteinuria	1 (0.8%)	1 (0.8%)	0	0	1 (0.8%)	
Renal colic	1 (0.8%)	1 (0.8%)	0	0	1 (0.8%)	
Vascular disorders	4 (3.3%)	4 (3.3%)	0	1 (0.8%)	4 (3.3%)	
Haematoma	2 (1.6%)	2 (1.6%)	0	0	2 (1.6%)	
Hot flush	1 (0.8%)	1 (0.8%)	0	0	1 (0.8%)	
Hypertension	0	1 (0.8%)	0	1 (0.8%)	2 (1.6%)	
Peripheral coldness	1 (0.8%)	1 (0.8%)	0	0	1 (0.8%)	
Hepatobiliary disorders	3 (2.4%)	3 (2.4%)	0	0	3 (2.4%)	
Hyperbilirubinaemia	3 (2.4%)	3 (2.4%)	0	0	3 (2.4%)	
Injury, poisoning and procedural complications	3 (2.4%)	3 (2.4%)	0	0	3 (2.4%)	
Fall	1 (0.8%)	1 (0.8%)	0	0	1 (0.8%)	
Joint injury	1 (0.8%)	1 (0.8%)	0	0	1 (0.8%)	
Limb injury	1 (0.8%)	1 (0.8%)	0	0	1 (0.8%)	
Endocrine disorders	0	1 (0.8%)	0	1 (0.8%)	2 (1.6%)	
Hyperthyroidism	0	1 (0.8%)	0	0	1 (0.8%)	
Thyroiditis	0	0	0	1 (0.8%)	1 (0.8%)	
	
[TSFAE02TDG112.RTF] [TMC435\HPC3014\DBR_FINAL_ANALYSIS\RE_FINAL_ANALYSIS\PROD\TSFAE02TDG1.SAS] 02NOV2015, 11:22	
