# Supplementary material for: An Open-Label Trial of 12-Week Simeprevir plus Peginterferon/Ribavirin (PR) in Treatment-Naïve Patients with Hepatitis C Virus (HCV) Genotype 1 (GT1)
Source: PLoS One. 2016 Jul 18;11(7):e0158526. doi: 10.1371/journal.pone.0158526 (PMC4948848; doi:10.1371/journal.pone.0158526)
Supplement: S1 Dataset — (ZIP) [file pone.0158526.s009.zip › Safety data/tsfae02tdg1gt12.rtf]

TSFAE02TDG1GT12:	Number (pcnt) of Genotype 1 Subjects with Adverse Events, Intent-to-treat, Study TMC435HPC3014 Trt Dur >12 Wks	
	Simeprevir
12 Wks
150 mg
PR 12/24 	
	SMV + PR 	Ent Trt 	PR Only 	Follow-Up 	Overall 	
Analysis set: Intent-to-treat	40	40	30	38	40	
Any AE	37 (92.5%)	37 (92.5%)	20 (66.7%)	7 (18.4%)	37 (92.5%)	
General disorders and administration site conditions	31 (77.5%)	32 (80.0%)	3 (10.0%)	1 (2.6%)	32 (80.0%)	
Asthenia	13 (32.5%)	14 (35.0%)	1 (3.3%)	0	14 (35.0%)	
Influenza like illness	11 (27.5%)	12 (30.0%)	1 (3.3%)	0	12 (30.0%)	
Fatigue	6 (15.0%)	7 (17.5%)	1 (3.3%)	0	7 (17.5%)	
Pyrexia	7 (17.5%)	7 (17.5%)	0	0	7 (17.5%)	
Irritability	4 (10.0%)	4 (10.0%)	0	1 (2.6%)	5 (12.5%)	
Injection site erythema	3 (7.5%)	3 (7.5%)	0	0	3 (7.5%)	
Skin and subcutaneous tissue disorders	18 (45.0%)	21 (52.5%)	9 (30.0%)	2 (5.3%)	23 (57.5%)	
Pruritus	11 (27.5%)	12 (30.0%)	0	1 (2.6%)	13 (32.5%)	
Rash	6 (15.0%)	7 (17.5%)	3 (10.0%)	0	7 (17.5%)	
Alopecia	1 (2.5%)	5 (12.5%)	4 (13.3%)	0	5 (12.5%)	
Dry skin	5 (12.5%)	5 (12.5%)	0	0	5 (12.5%)	
Erythema	2 (5.0%)	3 (7.5%)	1 (3.3%)	0	3 (7.5%)	
Dermatitis	2 (5.0%)	2 (5.0%)	0	0	2 (5.0%)	
Generalised erythema	0	1 (2.5%)	1 (3.3%)	0	1 (2.5%)	
Rash erythematous	1 (2.5%)	1 (2.5%)	0	0	1 (2.5%)	
Rash macular	1 (2.5%)	1 (2.5%)	0	0	1 (2.5%)	
Rash maculo-papular	1 (2.5%)	1 (2.5%)	0	0	1 (2.5%)	
Rash papular	0	1 (2.5%)	1 (3.3%)	0	1 (2.5%)	
Skin exfoliation	0	1 (2.5%)	1 (3.3%)	0	1 (2.5%)	
Toxic skin eruption	0	1 (2.5%)	1 (3.3%)	0	1 (2.5%)	
Night sweats	0	0	0	1 (2.6%)	1 (2.5%)	
Gastrointestinal disorders	16 (40.0%)	17 (42.5%)	1 (3.3%)	2 (5.3%)	18 (45.0%)	
Diarrhoea	5 (12.5%)	5 (12.5%)	0	0	5 (12.5%)	
Dyspepsia	3 (7.5%)	3 (7.5%)	0	0	3 (7.5%)	
Dry mouth	1 (2.5%)	2 (5.0%)	1 (3.3%)	0	2 (5.0%)	
Gastrooesophageal reflux disease	2 (5.0%)	2 (5.0%)	0	0	2 (5.0%)	
Nausea	2 (5.0%)	2 (5.0%)	0	0	2 (5.0%)	
Vomiting	2 (5.0%)	2 (5.0%)	0	0	2 (5.0%)	
Abdominal distension	1 (2.5%)	1 (2.5%)	0	0	1 (2.5%)	
Abdominal pain upper	1 (2.5%)	1 (2.5%)	0	0	1 (2.5%)	
Gastritis	1 (2.5%)	1 (2.5%)	0	1 (2.6%)	1 (2.5%)	
Stomatitis	1 (2.5%)	1 (2.5%)	0	0	1 (2.5%)	
Faecaloma	0	0	0	1 (2.6%)	1 (2.5%)	
Blood and lymphatic system disorders	10 (25.0%)	13 (32.5%)	4 (13.3%)	0	13 (32.5%)	
Neutropenia	7 (17.5%)	8 (20.0%)	1 (3.3%)	0	8 (20.0%)	
Anaemia	4 (10.0%)	7 (17.5%)	4 (13.3%)	0	7 (17.5%)	
Thrombocytopenia	1 (2.5%)	1 (2.5%)	0	0	1 (2.5%)	
Nervous system disorders	11 (27.5%)	13 (32.5%)	3 (10.0%)	0	13 (32.5%)	
Headache	9 (22.5%)	9 (22.5%)	1 (3.3%)	0	9 (22.5%)	
Dizziness	2 (5.0%)	2 (5.0%)	0	0	2 (5.0%)	
Dysgeusia	2 (5.0%)	2 (5.0%)	0	0	2 (5.0%)	
Amnesia	0	1 (2.5%)	1 (3.3%)	0	1 (2.5%)	
Dizziness postural	1 (2.5%)	1 (2.5%)	0	0	1 (2.5%)	
Epilepsy	0	1 (2.5%)	0	0	1 (2.5%)	
Sciatica	0	1 (2.5%)	1 (3.3%)	0	1 (2.5%)	
Infections and infestations	8 (20.0%)	11 (27.5%)	4 (13.3%)	1 (2.6%)	11 (27.5%)	
Oral candidiasis	1 (2.5%)	2 (5.0%)	1 (3.3%)	0	2 (5.0%)	
Bronchitis	1 (2.5%)	1 (2.5%)	0	0	1 (2.5%)	
Cystitis	1 (2.5%)	1 (2.5%)	0	0	1 (2.5%)	
Ear infection	0	1 (2.5%)	1 (3.3%)	0	1 (2.5%)	
Gastroenteritis	1 (2.5%)	1 (2.5%)	0	0	1 (2.5%)	
Influenza	1 (2.5%)	1 (2.5%)	0	0	1 (2.5%)	
Nasopharyngitis	1 (2.5%)	1 (2.5%)	0	0	1 (2.5%)	
Onychomycosis	0	1 (2.5%)	1 (3.3%)	0	1 (2.5%)	
Orchitis	0	1 (2.5%)	0	0	1 (2.5%)	
Respiratory tract infection	1 (2.5%)	1 (2.5%)	0	0	1 (2.5%)	
Upper respiratory tract infection	1 (2.5%)	1 (2.5%)	0	0	1 (2.5%)	
Urinary tract infection	0	1 (2.5%)	1 (3.3%)	0	1 (2.5%)	
Vulvovaginal candidiasis	1 (2.5%)	1 (2.5%)	0	0	1 (2.5%)	
Otitis media	0	0	0	1 (2.6%)	1 (2.5%)	
Musculoskeletal and connective tissue disorders	9 (22.5%)	9 (22.5%)	0	1 (2.6%)	9 (22.5%)	
Myalgia	5 (12.5%)	5 (12.5%)	0	0	5 (12.5%)	
Arthralgia	3 (7.5%)	3 (7.5%)	0	1 (2.6%)	4 (10.0%)	
Back pain	2 (5.0%)	2 (5.0%)	0	0	2 (5.0%)	
Growing pains	1 (2.5%)	1 (2.5%)	0	0	1 (2.5%)	
Musculoskeletal stiffness	1 (2.5%)	1 (2.5%)	0	0	1 (2.5%)	
Psychiatric disorders	9 (22.5%)	9 (22.5%)	1 (3.3%)	0	9 (22.5%)	
Sleep disorder	4 (10.0%)	4 (10.0%)	0	0	4 (10.0%)	
Anxiety	2 (5.0%)	3 (7.5%)	1 (3.3%)	0	3 (7.5%)	
Depression	2 (5.0%)	3 (7.5%)	1 (3.3%)	0	3 (7.5%)	
Insomnia	3 (7.5%)	3 (7.5%)	0	0	3 (7.5%)	
Depressed mood	1 (2.5%)	1 (2.5%)	0	0	1 (2.5%)	
Feeling of despair	0	1 (2.5%)	1 (3.3%)	0	1 (2.5%)	
Mood altered	1 (2.5%)	1 (2.5%)	0	0	1 (2.5%)	
Psychotic disorder	0	1 (2.5%)	1 (3.3%)	0	1 (2.5%)	
Respiratory, thoracic and mediastinal disorders	7 (17.5%)	7 (17.5%)	1 (3.3%)	0	7 (17.5%)	
Dyspnoea	4 (10.0%)	4 (10.0%)	0	0	4 (10.0%)	
Asthma	0	1 (2.5%)	1 (3.3%)	0	1 (2.5%)	
Cough	1 (2.5%)	1 (2.5%)	0	0	1 (2.5%)	
Epistaxis	1 (2.5%)	1 (2.5%)	0	0	1 (2.5%)	
Oropharyngeal pain	1 (2.5%)	1 (2.5%)	0	0	1 (2.5%)	
Metabolism and nutrition disorders	5 (12.5%)	5 (12.5%)	0	0	5 (12.5%)	
Decreased appetite	4 (10.0%)	4 (10.0%)	0	0	4 (10.0%)	
Hyponatraemia	1 (2.5%)	1 (2.5%)	0	0	1 (2.5%)	
Eye disorders	4 (10.0%)	4 (10.0%)	0	0	4 (10.0%)	
Glare	1 (2.5%)	1 (2.5%)	0	0	1 (2.5%)	
Photophobia	1 (2.5%)	1 (2.5%)	0	0	1 (2.5%)	
Retinal pigmentation	1 (2.5%)	1 (2.5%)	0	0	1 (2.5%)	
Vitreous haemorrhage	1 (2.5%)	1 (2.5%)	0	0	1 (2.5%)	
Investigations	4 (10.0%)	4 (10.0%)	0	1 (2.6%)	5 (12.5%)	
Haemoglobin decreased	2 (5.0%)	2 (5.0%)	0	0	2 (5.0%)	
Weight decreased	2 (5.0%)	2 (5.0%)	0	0	2 (5.0%)	
Blood bilirubin increased	1 (2.5%)	1 (2.5%)	0	0	1 (2.5%)	
Amylase increased	0	0	0	1 (2.6%)	1 (2.5%)	
Ear and labyrinth disorders	2 (5.0%)	2 (5.0%)	0	1 (2.6%)	3 (7.5%)	
Deafness	1 (2.5%)	1 (2.5%)	0	1 (2.6%)	2 (5.0%)	
Ear discomfort	1 (2.5%)	1 (2.5%)	0	0	1 (2.5%)	
Endocrine disorders	1 (2.5%)	2 (5.0%)	1 (3.3%)	0	2 (5.0%)	
Hypothyroidism	1 (2.5%)	2 (5.0%)	1 (3.3%)	0	2 (5.0%)	
Renal and urinary disorders	2 (5.0%)	2 (5.0%)	0	0	2 (5.0%)	
Pollakiuria	1 (2.5%)	1 (2.5%)	0	0	1 (2.5%)	
Urinary incontinence	1 (2.5%)	1 (2.5%)	0	0	1 (2.5%)	
Cardiac disorders	1 (2.5%)	1 (2.5%)	0	1 (2.6%)	2 (5.0%)	
Palpitations	1 (2.5%)	1 (2.5%)	0	0	1 (2.5%)	
Cardiomyopathy	0	0	0	1 (2.6%)	1 (2.5%)	
Myocarditis	0	0	0	1 (2.6%)	1 (2.5%)	
Immune system disorders	0	1 (2.5%)	1 (3.3%)	0	1 (2.5%)	
Seasonal allergy	0	1 (2.5%)	1 (3.3%)	0	1 (2.5%)	
Reproductive system and breast disorders	1 (2.5%)	1 (2.5%)	0	0	1 (2.5%)	
Erectile dysfunction	1 (2.5%)	1 (2.5%)	0	0	1 (2.5%)	
	
[TSFAE02TDG1GT12.RTF] [TMC435\HPC3014\DBR_FINAL_ANALYSIS\RE_FINAL_ANALYSIS\PROD\TSFAE02TDG1.SAS] 02NOV2015, 11:22	
