# Supplementary material for: An Open-Label Trial of 12-Week Simeprevir plus Peginterferon/Ribavirin (PR) in Treatment-Naïve Patients with Hepatitis C Virus (HCV) Genotype 1 (GT1)
Source: PLoS One. 2016 Jul 18;11(7):e0158526. doi: 10.1371/journal.pone.0158526 (PMC4948848; doi:10.1371/journal.pone.0158526)
Supplement: S1 Dataset — (ZIP) [file pone.0158526.s009.zip › Safety data/tsfae05tdg112.rtf]

TSFAE05TDG112:	TSFAE05TDG112: Number (pcnt) of Genotype 1 Subjects with Adverse Events by Worst WHO Toxicity Grade, Intent-to-treat, Study TMC435HPC3014 Trt Dur 12 Wks	
	Simeprevir
12 Wks
150 mg
PR 12/24 	
	SMV + PR 	Ent Trt 	PR Only 	Follow-Up 	Overall 	
Analysis set: Intent-to-treat	123	123	2	122	123	
Any Grade 1 AE	54 (43.9%)	54 (43.9%)	0	18 (14.8%)	49 (39.8%)	
General disorders and administration site conditions	60 (48.8%)	60 (48.8%)	0	1 (0.8%)	60 (48.8%)	
Influenza like illness	37 (30.1%)	37 (30.1%)	0	0	37 (30.1%)	
Fatigue	28 (22.8%)	28 (22.8%)	0	0	28 (22.8%)	
Asthenia	16 (13.0%)	16 (13.0%)	0	0	16 (13.0%)	
Pyrexia	9 (7.3%)	9 (7.3%)	0	0	9 (7.3%)	
Irritability	7 (5.7%)	7 (5.7%)	0	0	7 (5.7%)	
Injection site erythema	4 (3.3%)	4 (3.3%)	0	0	4 (3.3%)	
Chest discomfort	2 (1.6%)	2 (1.6%)	0	0	2 (1.6%)	
Feeling cold	2 (1.6%)	2 (1.6%)	0	0	2 (1.6%)	
Non-cardiac chest pain	1 (0.8%)	1 (0.8%)	0	1 (0.8%)	2 (1.6%)	
Injection site haematoma	1 (0.8%)	1 (0.8%)	0	0	1 (0.8%)	
Injection site rash	1 (0.8%)	1 (0.8%)	0	0	1 (0.8%)	
Malaise	1 (0.8%)	1 (0.8%)	0	0	1 (0.8%)	
Mucosal dryness	1 (0.8%)	1 (0.8%)	0	0	1 (0.8%)	
Mucosal inflammation	1 (0.8%)	1 (0.8%)	0	0	1 (0.8%)	
Skin and subcutaneous tissue disorders	59 (48.0%)	60 (48.8%)	0	10 (8.2%)	60 (48.8%)	
Pruritus	37 (30.1%)	39 (31.7%)	0	4 (3.3%)	42 (34.1%)	
Dry skin	22 (17.9%)	23 (18.7%)	0	1 (0.8%)	24 (19.5%)	
Rash	14 (11.4%)	14 (11.4%)	0	2 (1.6%)	15 (12.2%)	
Alopecia	5 (4.1%)	9 (7.3%)	0	3 (2.5%)	12 (9.8%)	
Erythema	3 (2.4%)	3 (2.4%)	0	0	3 (2.4%)	
Eczema	2 (1.6%)	2 (1.6%)	0	0	2 (1.6%)	
Hyperhidrosis	2 (1.6%)	2 (1.6%)	0	0	2 (1.6%)	
Psoriasis	1 (0.8%)	1 (0.8%)	0	1 (0.8%)	2 (1.6%)	
Dermatitis	1 (0.8%)	1 (0.8%)	0	0	1 (0.8%)	
Dermatosis	1 (0.8%)	1 (0.8%)	0	0	1 (0.8%)	
Generalised erythema	1 (0.8%)	1 (0.8%)	0	0	1 (0.8%)	
Nail discolouration	1 (0.8%)	1 (0.8%)	0	0	1 (0.8%)	
Pruritus generalised	1 (0.8%)	1 (0.8%)	0	0	1 (0.8%)	
Rash macular	1 (0.8%)	1 (0.8%)	0	0	1 (0.8%)	
Rash pruritic	1 (0.8%)	1 (0.8%)	0	0	1 (0.8%)	
Skin discolouration	1 (0.8%)	1 (0.8%)	0	0	1 (0.8%)	
Skin fissures	1 (0.8%)	1 (0.8%)	0	0	1 (0.8%)	
Skin irritation	1 (0.8%)	1 (0.8%)	0	0	1 (0.8%)	
Skin mass	1 (0.8%)	1 (0.8%)	0	0	1 (0.8%)	
Solar dermatitis	1 (0.8%)	1 (0.8%)	0	0	1 (0.8%)	
Nervous system disorders	33 (26.8%)	35 (28.5%)	0	3 (2.5%)	34 (27.6%)	
Headache	22 (17.9%)	24 (19.5%)	0	1 (0.8%)	24 (19.5%)	
Dizziness	7 (5.7%)	7 (5.7%)	0	1 (0.8%)	8 (6.5%)	
Dysgeusia	7 (5.7%)	7 (5.7%)	0	0	7 (5.7%)	
Disturbance in attention	4 (3.3%)	4 (3.3%)	0	0	4 (3.3%)	
Sciatica	1 (0.8%)	2 (1.6%)	0	1 (0.8%)	3 (2.4%)	
Carpal tunnel syndrome	0	0	0	1 (0.8%)	1 (0.8%)	
Dizziness postural	0	0	0	1 (0.8%)	1 (0.8%)	
Hyperaesthesia	1 (0.8%)	1 (0.8%)	0	0	1 (0.8%)	
Migraine	1 (0.8%)	1 (0.8%)	0	0	1 (0.8%)	
Paraesthesia	1 (0.8%)	1 (0.8%)	0	0	1 (0.8%)	
Poor quality sleep	1 (0.8%)	1 (0.8%)	0	0	1 (0.8%)	
Presyncope	1 (0.8%)	1 (0.8%)	0	0	1 (0.8%)	
Somnolence	1 (0.8%)	1 (0.8%)	0	0	1 (0.8%)	
Tremor	0	0	0	1 (0.8%)	1 (0.8%)	
Gastrointestinal disorders	34 (27.6%)	33 (26.8%)	0	0	33 (26.8%)	
Nausea	11 (8.9%)	11 (8.9%)	0	0	11 (8.9%)	
Diarrhoea	7 (5.7%)	7 (5.7%)	0	0	7 (5.7%)	
Abdominal pain upper	5 (4.1%)	6 (4.9%)	0	0	6 (4.9%)	
Constipation	4 (3.3%)	4 (3.3%)	0	0	4 (3.3%)	
Dry mouth	4 (3.3%)	4 (3.3%)	0	0	4 (3.3%)	
Dyspepsia	4 (3.3%)	4 (3.3%)	0	0	4 (3.3%)	
Gastrooesophageal reflux disease	4 (3.3%)	4 (3.3%)	0	0	4 (3.3%)	
Vomiting	4 (3.3%)	4 (3.3%)	0	0	4 (3.3%)	
Abdominal pain	3 (2.4%)	3 (2.4%)	0	0	3 (2.4%)	
Anal pruritus	2 (1.6%)	2 (1.6%)	0	0	2 (1.6%)	
Dysphagia	2 (1.6%)	2 (1.6%)	0	0	2 (1.6%)	
Gingival bleeding	2 (1.6%)	2 (1.6%)	0	0	2 (1.6%)	
Mouth ulceration	2 (1.6%)	2 (1.6%)	0	0	2 (1.6%)	
Toothache	2 (1.6%)	2 (1.6%)	0	0	2 (1.6%)	
Cheilitis	1 (0.8%)	1 (0.8%)	0	0	1 (0.8%)	
Gingival pain	1 (0.8%)	1 (0.8%)	0	0	1 (0.8%)	
Tongue ulceration	1 (0.8%)	1 (0.8%)	0	0	1 (0.8%)	
Psychiatric disorders	29 (23.6%)	30 (24.4%)	0	1 (0.8%)	31 (25.2%)	
Insomnia	18 (14.6%)	19 (15.4%)	0	0	19 (15.4%)	
Depression	7 (5.7%)	7 (5.7%)	0	1 (0.8%)	8 (6.5%)	
Sleep disorder	4 (3.3%)	4 (3.3%)	0	0	4 (3.3%)	
Anxiety	3 (2.4%)	3 (2.4%)	0	0	3 (2.4%)	
Depressed mood	3 (2.4%)	3 (2.4%)	0	0	3 (2.4%)	
Affect lability	2 (1.6%)	2 (1.6%)	0	0	2 (1.6%)	
Aggression	1 (0.8%)	1 (0.8%)	0	0	1 (0.8%)	
Emotional disorder	1 (0.8%)	1 (0.8%)	0	0	1 (0.8%)	
Middle insomnia	1 (0.8%)	1 (0.8%)	0	0	1 (0.8%)	
Mood altered	1 (0.8%)	1 (0.8%)	0	0	1 (0.8%)	
Mood swings	1 (0.8%)	1 (0.8%)	0	0	1 (0.8%)	
Nicotine dependence	1 (0.8%)	1 (0.8%)	0	0	1 (0.8%)	
Stress	1 (0.8%)	1 (0.8%)	0	0	1 (0.8%)	
Musculoskeletal and connective tissue disorders	27 (22.0%)	27 (22.0%)	0	3 (2.5%)	28 (22.8%)	
Arthralgia	13 (10.6%)	13 (10.6%)	0	1 (0.8%)	14 (11.4%)	
Myalgia	8 (6.5%)	8 (6.5%)	0	1 (0.8%)	9 (7.3%)	
Back pain	4 (3.3%)	4 (3.3%)	0	1 (0.8%)	5 (4.1%)	
Muscle spasms	3 (2.4%)	3 (2.4%)	0	0	3 (2.4%)	
Musculoskeletal pain	1 (0.8%)	1 (0.8%)	0	1 (0.8%)	2 (1.6%)	
Sensation of heaviness	1 (0.8%)	2 (1.6%)	0	0	2 (1.6%)	
Axillary mass	1 (0.8%)	1 (0.8%)	0	0	1 (0.8%)	
Muscle twitching	1 (0.8%)	1 (0.8%)	0	0	1 (0.8%)	
Neck pain	1 (0.8%)	1 (0.8%)	0	0	1 (0.8%)	
Rheumatic disorder	1 (0.8%)	1 (0.8%)	0	0	1 (0.8%)	
Respiratory, thoracic and mediastinal disorders	20 (16.3%)	22 (17.9%)	0	2 (1.6%)	23 (18.7%)	
Dyspnoea	10 (8.1%)	10 (8.1%)	0	0	10 (8.1%)	
Cough	7 (5.7%)	7 (5.7%)	0	1 (0.8%)	8 (6.5%)	
Dyspnoea exertional	5 (4.1%)	5 (4.1%)	0	1 (0.8%)	6 (4.9%)	
Epistaxis	3 (2.4%)	3 (2.4%)	0	0	3 (2.4%)	
Asthma	0	1 (0.8%)	0	0	1 (0.8%)	
Rhinitis allergic	0	1 (0.8%)	0	0	1 (0.8%)	
Metabolism and nutrition disorders	17 (13.8%)	16 (13.0%)	0	1 (0.8%)	17 (13.8%)	
Decreased appetite	15 (12.2%)	15 (12.2%)	0	0	15 (12.2%)	
Hyperamylasaemia	1 (0.8%)	1 (0.8%)	0	0	1 (0.8%)	
Hyperproteinaemia	0	0	0	1 (0.8%)	1 (0.8%)	
Increased appetite	1 (0.8%)	1 (0.8%)	0	0	1 (0.8%)	
Infections and infestations	10 (8.1%)	11 (8.9%)	0	2 (1.6%)	12 (9.8%)	
Bronchitis	2 (1.6%)	2 (1.6%)	0	0	2 (1.6%)	
Nasopharyngitis	1 (0.8%)	1 (0.8%)	0	1 (0.8%)	2 (1.6%)	
Gingivitis	1 (0.8%)	1 (0.8%)	0	0	1 (0.8%)	
Herpes simplex	1 (0.8%)	1 (0.8%)	0	0	1 (0.8%)	
Influenza	1 (0.8%)	1 (0.8%)	0	0	1 (0.8%)	
Oral candidiasis	1 (0.8%)	1 (0.8%)	0	0	1 (0.8%)	
Otitis media	1 (0.8%)	1 (0.8%)	0	0	1 (0.8%)	
Rhinitis	1 (0.8%)	1 (0.8%)	0	0	1 (0.8%)	
Tooth abscess	1 (0.8%)	1 (0.8%)	0	0	1 (0.8%)	
Tooth infection	0	0	0	1 (0.8%)	1 (0.8%)	
Urinary tract infection	0	1 (0.8%)	0	0	1 (0.8%)	
Ear and labyrinth disorders	11 (8.9%)	11 (8.9%)	0	0	11 (8.9%)	
Vertigo	7 (5.7%)	7 (5.7%)	0	0	7 (5.7%)	
Tinnitus	4 (3.3%)	4 (3.3%)	0	0	4 (3.3%)	
Hypoacusis	1 (0.8%)	1 (0.8%)	0	0	1 (0.8%)	
Eye disorders	7 (5.7%)	8 (6.5%)	0	0	8 (6.5%)	
Dry eye	2 (1.6%)	2 (1.6%)	0	0	2 (1.6%)	
Chalazion	0	1 (0.8%)	0	0	1 (0.8%)	
Conjunctival irritation	1 (0.8%)	1 (0.8%)	0	0	1 (0.8%)	
Conjunctival ulcer	1 (0.8%)	1 (0.8%)	0	0	1 (0.8%)	
Eye irritation	1 (0.8%)	1 (0.8%)	0	0	1 (0.8%)	
Ocular hyperaemia	1 (0.8%)	1 (0.8%)	0	0	1 (0.8%)	
Vision blurred	1 (0.8%)	1 (0.8%)	0	0	1 (0.8%)	
Investigations	8 (6.5%)	8 (6.5%)	0	0	8 (6.5%)	
Blood bilirubin increased	4 (3.3%)	4 (3.3%)	0	0	4 (3.3%)	
Weight decreased	3 (2.4%)	3 (2.4%)	0	0	3 (2.4%)	
Body temperature increased	1 (0.8%)	1 (0.8%)	0	0	1 (0.8%)	
General physical condition abnormal	1 (0.8%)	1 (0.8%)	0	0	1 (0.8%)	
Blood and lymphatic system disorders	7 (5.7%)	7 (5.7%)	0	0	7 (5.7%)	
Anaemia	8 (6.5%)	8 (6.5%)	0	0	8 (6.5%)	
Leukopenia	6 (4.9%)	6 (4.9%)	0	0	6 (4.9%)	
Neutropenia	4 (3.3%)	4 (3.3%)	0	0	4 (3.3%)	
Thrombocytopenia	3 (2.4%)	3 (2.4%)	0	0	3 (2.4%)	
Lymphadenopathy	0	1 (0.8%)	0	0	1 (0.8%)	
Lymphopenia	1 (0.8%)	1 (0.8%)	0	0	1 (0.8%)	
Renal and urinary disorders	5 (4.1%)	5 (4.1%)	0	0	5 (4.1%)	
Chromaturia	2 (1.6%)	2 (1.6%)	0	0	2 (1.6%)	
Cystitis-like symptom	1 (0.8%)	1 (0.8%)	0	0	1 (0.8%)	
Proteinuria	1 (0.8%)	1 (0.8%)	0	0	1 (0.8%)	
Renal colic	1 (0.8%)	1 (0.8%)	0	0	1 (0.8%)	
Vascular disorders	4 (3.3%)	4 (3.3%)	0	1 (0.8%)	4 (3.3%)	
Haematoma	2 (1.6%)	2 (1.6%)	0	0	2 (1.6%)	
Hypertension	0	1 (0.8%)	0	1 (0.8%)	2 (1.6%)	
Hot flush	1 (0.8%)	1 (0.8%)	0	0	1 (0.8%)	
Peripheral coldness	1 (0.8%)	1 (0.8%)	0	0	1 (0.8%)	
Cardiac disorders	3 (2.4%)	3 (2.4%)	0	0	3 (2.4%)	
Tachycardia	2 (1.6%)	2 (1.6%)	0	0	2 (1.6%)	
Palpitations	1 (0.8%)	1 (0.8%)	0	0	1 (0.8%)	
Reproductive system and breast disorders	3 (2.4%)	3 (2.4%)	0	0	3 (2.4%)	
Metrorrhagia	1 (0.8%)	1 (0.8%)	0	0	1 (0.8%)	
Pelvic discomfort	1 (0.8%)	1 (0.8%)	0	0	1 (0.8%)	
Vaginal discharge	1 (0.8%)	1 (0.8%)	0	0	1 (0.8%)	
Endocrine disorders	0	1 (0.8%)	0	0	1 (0.8%)	
Hyperthyroidism	0	1 (0.8%)	0	0	1 (0.8%)	
Hepatobiliary disorders	1 (0.8%)	1 (0.8%)	0	0	1 (0.8%)	
Hyperbilirubinaemia	1 (0.8%)	1 (0.8%)	0	0	1 (0.8%)	
Injury, poisoning and procedural complications	1 (0.8%)	1 (0.8%)	0	0	1 (0.8%)	
Joint injury	1 (0.8%)	1 (0.8%)	0	0	1 (0.8%)	
Any Grade 2 AE	37 (30.1%)	37 (30.1%)	0	9 (7.4%)	39 (31.7%)	
General disorders and administration site conditions	29 (23.6%)	29 (23.6%)	0	0	29 (23.6%)	
Asthenia	11 (8.9%)	11 (8.9%)	0	0	11 (8.9%)	
Fatigue	10 (8.1%)	10 (8.1%)	0	0	10 (8.1%)	
Influenza like illness	10 (8.1%)	10 (8.1%)	0	0	10 (8.1%)	
Pyrexia	3 (2.4%)	3 (2.4%)	0	0	3 (2.4%)	
Irritability	2 (1.6%)	2 (1.6%)	0	0	2 (1.6%)	
Injection site erythema	1 (0.8%)	1 (0.8%)	0	0	1 (0.8%)	
Injection site pain	1 (0.8%)	1 (0.8%)	0	0	1 (0.8%)	
Oedema	1 (0.8%)	1 (0.8%)	0	0	1 (0.8%)	
Pain	1 (0.8%)	1 (0.8%)	0	0	1 (0.8%)	
Gastrointestinal disorders	15 (12.2%)	16 (13.0%)	0	0	16 (13.0%)	
Nausea	4 (3.3%)	4 (3.3%)	0	0	4 (3.3%)	
Dry mouth	3 (2.4%)	3 (2.4%)	0	0	3 (2.4%)	
Diarrhoea	2 (1.6%)	2 (1.6%)	0	0	2 (1.6%)	
Abdominal discomfort	1 (0.8%)	1 (0.8%)	0	0	1 (0.8%)	
Abdominal distension	1 (0.8%)	1 (0.8%)	0	0	1 (0.8%)	
Abdominal pain	1 (0.8%)	1 (0.8%)	0	0	1 (0.8%)	
Abdominal pain upper	1 (0.8%)	1 (0.8%)	0	0	1 (0.8%)	
Constipation	1 (0.8%)	1 (0.8%)	0	0	1 (0.8%)	
Dyspepsia	1 (0.8%)	1 (0.8%)	0	0	1 (0.8%)	
Gastric ulcer	1 (0.8%)	1 (0.8%)	0	0	1 (0.8%)	
Haemorrhoids	0	1 (0.8%)	0	0	1 (0.8%)	
Infections and infestations	9 (7.3%)	10 (8.1%)	0	2 (1.6%)	12 (9.8%)	
Influenza	2 (1.6%)	2 (1.6%)	0	0	2 (1.6%)	
Acarodermatitis	1 (0.8%)	1 (0.8%)	0	0	1 (0.8%)	
Acute tonsillitis	1 (0.8%)	1 (0.8%)	0	0	1 (0.8%)	
Bronchitis	1 (0.8%)	1 (0.8%)	0	0	1 (0.8%)	
Furuncle	1 (0.8%)	1 (0.8%)	0	0	1 (0.8%)	
Helicobacter gastritis	1 (0.8%)	1 (0.8%)	0	0	1 (0.8%)	
Herpes simplex	1 (0.8%)	1 (0.8%)	0	0	1 (0.8%)	
Mastitis	1 (0.8%)	1 (0.8%)	0	0	1 (0.8%)	
Pneumonia	0	0	0	1 (0.8%)	1 (0.8%)	
Respiratory tract infection	0	1 (0.8%)	0	0	1 (0.8%)	
Sinusitis	1 (0.8%)	1 (0.8%)	0	0	1 (0.8%)	
Subcutaneous abscess	1 (0.8%)	1 (0.8%)	0	0	1 (0.8%)	
Tooth abscess	0	0	0	1 (0.8%)	1 (0.8%)	
Tracheitis	1 (0.8%)	1 (0.8%)	0	0	1 (0.8%)	
Nervous system disorders	10 (8.1%)	10 (8.1%)	0	2 (1.6%)	12 (9.8%)	
Headache	8 (6.5%)	8 (6.5%)	0	1 (0.8%)	9 (7.3%)	
Ageusia	1 (0.8%)	1 (0.8%)	0	0	1 (0.8%)	
Dysgeusia	1 (0.8%)	1 (0.8%)	0	0	1 (0.8%)	
Memory impairment	1 (0.8%)	1 (0.8%)	0	0	1 (0.8%)	
Neuralgia	0	0	0	1 (0.8%)	1 (0.8%)	
Psychiatric disorders	12 (9.8%)	12 (9.8%)	0	0	12 (9.8%)	
Sleep disorder	5 (4.1%)	5 (4.1%)	0	0	5 (4.1%)	
Insomnia	3 (2.4%)	3 (2.4%)	0	0	3 (2.4%)	
Depression	2 (1.6%)	2 (1.6%)	0	0	2 (1.6%)	
Mood swings	2 (1.6%)	2 (1.6%)	0	0	2 (1.6%)	
Aggression	1 (0.8%)	1 (0.8%)	0	0	1 (0.8%)	
Anger	1 (0.8%)	1 (0.8%)	0	0	1 (0.8%)	
Anxiety	1 (0.8%)	1 (0.8%)	0	0	1 (0.8%)	
Emotional disorder	1 (0.8%)	1 (0.8%)	0	0	1 (0.8%)	
Skin and subcutaneous tissue disorders	8 (6.5%)	8 (6.5%)	0	4 (3.3%)	12 (9.8%)	
Pruritus	4 (3.3%)	4 (3.3%)	0	0	4 (3.3%)	
Rash	2 (1.6%)	2 (1.6%)	0	1 (0.8%)	3 (2.4%)	
Alopecia	0	0	0	2 (1.6%)	2 (1.6%)	
Dry skin	1 (0.8%)	1 (0.8%)	0	0	1 (0.8%)	
Psoriasis	1 (0.8%)	1 (0.8%)	0	0	1 (0.8%)	
Rosacea	0	0	0	1 (0.8%)	1 (0.8%)	
Blood and lymphatic system disorders	8 (6.5%)	8 (6.5%)	0	0	8 (6.5%)	
Neutropenia	6 (4.9%)	6 (4.9%)	0	0	6 (4.9%)	
Anaemia	4 (3.3%)	4 (3.3%)	0	0	4 (3.3%)	
Metabolism and nutrition disorders	6 (4.9%)	7 (5.7%)	0	0	7 (5.7%)	
Decreased appetite	3 (2.4%)	3 (2.4%)	0	0	3 (2.4%)	
Gout	1 (0.8%)	2 (1.6%)	0	0	2 (1.6%)	
Hyperlipasaemia	1 (0.8%)	1 (0.8%)	0	0	1 (0.8%)	
Increased appetite	1 (0.8%)	1 (0.8%)	0	0	1 (0.8%)	
Musculoskeletal and connective tissue disorders	7 (5.7%)	7 (5.7%)	0	0	7 (5.7%)	
Myalgia	3 (2.4%)	3 (2.4%)	0	0	3 (2.4%)	
Arthralgia	1 (0.8%)	1 (0.8%)	0	0	1 (0.8%)	
Back pain	1 (0.8%)	1 (0.8%)	0	0	1 (0.8%)	
Pain in extremity	1 (0.8%)	1 (0.8%)	0	0	1 (0.8%)	
Polyarthritis	1 (0.8%)	1 (0.8%)	0	0	1 (0.8%)	
Respiratory, thoracic and mediastinal disorders	7 (5.7%)	7 (5.7%)	0	0	7 (5.7%)	
Dyspnoea	3 (2.4%)	3 (2.4%)	0	0	3 (2.4%)	
Asthma	1 (0.8%)	1 (0.8%)	0	0	1 (0.8%)	
Cough	1 (0.8%)	1 (0.8%)	0	0	1 (0.8%)	
Dyspnoea exertional	1 (0.8%)	1 (0.8%)	0	0	1 (0.8%)	
Laryngeal ulceration	1 (0.8%)	1 (0.8%)	0	0	1 (0.8%)	
Oropharyngeal pain	1 (0.8%)	1 (0.8%)	0	0	1 (0.8%)	
Investigations	7 (5.7%)	7 (5.7%)	0	0	6 (4.9%)	
Alanine aminotransferase increased	2 (1.6%)	2 (1.6%)	0	0	2 (1.6%)	
Aspartate aminotransferase increased	2 (1.6%)	2 (1.6%)	0	0	2 (1.6%)	
Blood bilirubin increased	2 (1.6%)	2 (1.6%)	0	0	2 (1.6%)	
Gamma-glutamyltransferase increased	2 (1.6%)	2 (1.6%)	0	0	2 (1.6%)	
Haemoglobin decreased	2 (1.6%)	2 (1.6%)	0	0	2 (1.6%)	
Blood lactate dehydrogenase increased	1 (0.8%)	1 (0.8%)	0	0	1 (0.8%)	
Lipase increased	0	0	0	1 (0.8%)	1 (0.8%)	
Weight decreased	1 (0.8%)	1 (0.8%)	0	0	1 (0.8%)	
Weight increased	1 (0.8%)	1 (0.8%)	0	0	1 (0.8%)	
Reproductive system and breast disorders	3 (2.4%)	3 (2.4%)	0	1 (0.8%)	4 (3.3%)	
Menorrhagia	0	0	0	1 (0.8%)	1 (0.8%)	
Menstrual disorder	1 (0.8%)	1 (0.8%)	0	0	1 (0.8%)	
Vaginal haemorrhage	1 (0.8%)	1 (0.8%)	0	0	1 (0.8%)	
Vulvovaginal pruritus	1 (0.8%)	1 (0.8%)	0	0	1 (0.8%)	
Injury, poisoning and procedural complications	2 (1.6%)	2 (1.6%)	0	0	2 (1.6%)	
Fall	1 (0.8%)	1 (0.8%)	0	0	1 (0.8%)	
Limb injury	1 (0.8%)	1 (0.8%)	0	0	1 (0.8%)	
Cardiac disorders	1 (0.8%)	1 (0.8%)	0	0	1 (0.8%)	
Tachycardia	1 (0.8%)	1 (0.8%)	0	0	1 (0.8%)	
Eye disorders	1 (0.8%)	1 (0.8%)	0	0	1 (0.8%)	
Visual impairment	1 (0.8%)	1 (0.8%)	0	0	1 (0.8%)	
Hepatobiliary disorders	1 (0.8%)	1 (0.8%)	0	0	1 (0.8%)	
Hyperbilirubinaemia	1 (0.8%)	1 (0.8%)	0	0	1 (0.8%)	
Any Grade 3 AE	23 (18.7%)	23 (18.7%)	0	2 (1.6%)	24 (19.5%)	
Blood and lymphatic system disorders	13 (10.6%)	13 (10.6%)	0	0	13 (10.6%)	
Neutropenia	12 (9.8%)	12 (9.8%)	0	0	12 (9.8%)	
Thrombocytopenia	1 (0.8%)	1 (0.8%)	0	0	1 (0.8%)	
Investigations	3 (2.4%)	3 (2.4%)	0	1 (0.8%)	4 (3.3%)	
Blood creatine phosphokinase increased	1 (0.8%)	1 (0.8%)	0	1 (0.8%)	2 (1.6%)	
Blood bilirubin increased	1 (0.8%)	1 (0.8%)	0	0	1 (0.8%)	
Neutrophil count decreased	1 (0.8%)	1 (0.8%)	0	0	1 (0.8%)	
Psychiatric disorders	2 (1.6%)	2 (1.6%)	0	3 (2.5%)	4 (3.3%)	
Depression	1 (0.8%)	1 (0.8%)	0	1 (0.8%)	2 (1.6%)	
Alcohol withdrawal syndrome	1 (0.8%)	1 (0.8%)	0	1 (0.8%)	1 (0.8%)	
Psychotic disorder	0	0	0	1 (0.8%)	1 (0.8%)	
Schizophrenia, paranoid type	0	0	0	1 (0.8%)	1 (0.8%)	
General disorders and administration site conditions	2 (1.6%)	2 (1.6%)	0	0	2 (1.6%)	
Asthenia	2 (1.6%)	2 (1.6%)	0	0	2 (1.6%)	
Cardiac disorders	1 (0.8%)	1 (0.8%)	0	0	1 (0.8%)	
Tachycardia	1 (0.8%)	1 (0.8%)	0	0	1 (0.8%)	
Ear and labyrinth disorders	1 (0.8%)	1 (0.8%)	0	0	1 (0.8%)	
Vertigo	1 (0.8%)	1 (0.8%)	0	0	1 (0.8%)	
Hepatobiliary disorders	1 (0.8%)	1 (0.8%)	0	0	1 (0.8%)	
Hyperbilirubinaemia	1 (0.8%)	1 (0.8%)	0	0	1 (0.8%)	
Infections and infestations	1 (0.8%)	1 (0.8%)	0	0	1 (0.8%)	
Pericoronitis	1 (0.8%)	1 (0.8%)	0	0	1 (0.8%)	
Musculoskeletal and connective tissue disorders	1 (0.8%)	1 (0.8%)	0	0	1 (0.8%)	
Arthralgia	1 (0.8%)	1 (0.8%)	0	0	1 (0.8%)	
Reproductive system and breast disorders	1 (0.8%)	1 (0.8%)	0	0	1 (0.8%)	
Testicular necrosis	1 (0.8%)	1 (0.8%)	0	0	1 (0.8%)	
Skin and subcutaneous tissue disorders	1 (0.8%)	1 (0.8%)	0	0	1 (0.8%)	
Rash	1 (0.8%)	1 (0.8%)	0	0	1 (0.8%)	
Any Grade 4 AE	3 (2.4%)	3 (2.4%)	0	2 (1.6%)	5 (4.1%)	
Blood and lymphatic system disorders	2 (1.6%)	2 (1.6%)	0	0	2 (1.6%)	
Neutropenia	2 (1.6%)	2 (1.6%)	0	0	2 (1.6%)	
Endocrine disorders	0	0	0	1 (0.8%)	1 (0.8%)	
Thyroiditis	0	0	0	1 (0.8%)	1 (0.8%)	
Investigations	0	0	0	1 (0.8%)	1 (0.8%)	
Amylase increased	0	0	0	1 (0.8%)	1 (0.8%)	
Psychiatric disorders	1 (0.8%)	1 (0.8%)	0	0	1 (0.8%)	
Sleep disorder	1 (0.8%)	1 (0.8%)	0	0	1 (0.8%)	
Any Grade 3-4 AE	26 (21.1%)	26 (21.1%)	0	4 (3.3%)	29 (23.6%)	
Blood and lymphatic system disorders	15 (12.2%)	15 (12.2%)	0	0	15 (12.2%)	
Neutropenia	14 (11.4%)	14 (11.4%)	0	0	14 (11.4%)	
Thrombocytopenia	1 (0.8%)	1 (0.8%)	0	0	1 (0.8%)	
Investigations	3 (2.4%)	3 (2.4%)	0	2 (1.6%)	5 (4.1%)	
Blood creatine phosphokinase increased	1 (0.8%)	1 (0.8%)	0	1 (0.8%)	2 (1.6%)	
Amylase increased	0	0	0	1 (0.8%)	1 (0.8%)	
Blood bilirubin increased	1 (0.8%)	1 (0.8%)	0	0	1 (0.8%)	
Neutrophil count decreased	1 (0.8%)	1 (0.8%)	0	0	1 (0.8%)	
Psychiatric disorders	3 (2.4%)	3 (2.4%)	0	3 (2.5%)	5 (4.1%)	
Depression	1 (0.8%)	1 (0.8%)	0	1 (0.8%)	2 (1.6%)	
Alcohol withdrawal syndrome	1 (0.8%)	1 (0.8%)	0	1 (0.8%)	1 (0.8%)	
Psychotic disorder	0	0	0	1 (0.8%)	1 (0.8%)	
Schizophrenia, paranoid type	0	0	0	1 (0.8%)	1 (0.8%)	
Sleep disorder	1 (0.8%)	1 (0.8%)	0	0	1 (0.8%)	
General disorders and administration site conditions	2 (1.6%)	2 (1.6%)	0	0	2 (1.6%)	
Asthenia	2 (1.6%)	2 (1.6%)	0	0	2 (1.6%)	
Cardiac disorders	1 (0.8%)	1 (0.8%)	0	0	1 (0.8%)	
Tachycardia	1 (0.8%)	1 (0.8%)	0	0	1 (0.8%)	
Ear and labyrinth disorders	1 (0.8%)	1 (0.8%)	0	0	1 (0.8%)	
Vertigo	1 (0.8%)	1 (0.8%)	0	0	1 (0.8%)	
Endocrine disorders	0	0	0	1 (0.8%)	1 (0.8%)	
Thyroiditis	0	0	0	1 (0.8%)	1 (0.8%)	
Hepatobiliary disorders	1 (0.8%)	1 (0.8%)	0	0	1 (0.8%)	
Hyperbilirubinaemia	1 (0.8%)	1 (0.8%)	0	0	1 (0.8%)	
Infections and infestations	1 (0.8%)	1 (0.8%)	0	0	1 (0.8%)	
Pericoronitis	1 (0.8%)	1 (0.8%)	0	0	1 (0.8%)	
Musculoskeletal and connective tissue disorders	1 (0.8%)	1 (0.8%)	0	0	1 (0.8%)	
Arthralgia	1 (0.8%)	1 (0.8%)	0	0	1 (0.8%)	
Reproductive system and breast disorders	1 (0.8%)	1 (0.8%)	0	0	1 (0.8%)	
Testicular necrosis	1 (0.8%)	1 (0.8%)	0	0	1 (0.8%)	
Skin and subcutaneous tissue disorders	1 (0.8%)	1 (0.8%)	0	0	1 (0.8%)	
Rash	1 (0.8%)	1 (0.8%)	0	0	1 (0.8%)	
	
[TSFAE05TDG112.RTF] [TMC435\HPC3014\DBR_FINAL_ANALYSIS\RE_FINAL_ANALYSIS\PROD\TSFAE05TDG112.SAS] 02NOV2015, 11:20	
