# Supplementary material for: An Open-Label Trial of 12-Week Simeprevir plus Peginterferon/Ribavirin (PR) in Treatment-Naïve Patients with Hepatitis C Virus (HCV) Genotype 1 (GT1)
Source: PLoS One. 2016 Jul 18;11(7):e0158526. doi: 10.1371/journal.pone.0158526 (PMC4948848; doi:10.1371/journal.pone.0158526)
Supplement: S1 Dataset — (ZIP) [file pone.0158526.s009.zip › Safety data/tsfae05tdg1all.rtf]

TSFAE05TDG1ALL:	TSFAE05TDG1GTALL: Number (pcnt) of Genotype 1 Subjects with Adverse Events by Worst WHO Toxicity Grade, Intent-to-treat, Study TMC435HPC3014 All Subjects	
	Simeprevir
12 Wks
150 mg
PR 12/24 	
	SMV + PR 	Ent Trt 	PR Only 	Follow-Up 	Overall 	
Analysis set: Intent-to-treat	163	163	32	160	163	
Any Grade 1 AE	70 (42.9%)	67 (41.1%)	11 (34.4%)	22 (13.8%)	61 (37.4%)	
General disorders and administration site conditions	84 (51.5%)	85 (52.1%)	3 (9.4%)	2 (1.3%)	85 (52.1%)	
Influenza like illness	48 (29.4%)	49 (30.1%)	1 (3.1%)	0	49 (30.1%)	
Fatigue	32 (19.6%)	33 (20.2%)	1 (3.1%)	0	33 (20.2%)	
Asthenia	25 (15.3%)	26 (16.0%)	1 (3.1%)	0	26 (16.0%)	
Pyrexia	15 (9.2%)	15 (9.2%)	0	0	15 (9.2%)	
Irritability	10 (6.1%)	10 (6.1%)	0	1 (0.6%)	11 (6.7%)	
Injection site erythema	6 (3.7%)	6 (3.7%)	0	0	6 (3.7%)	
Chest discomfort	2 (1.2%)	2 (1.2%)	0	0	2 (1.2%)	
Feeling cold	2 (1.2%)	2 (1.2%)	0	0	2 (1.2%)	
Non-cardiac chest pain	1 (0.6%)	1 (0.6%)	0	1 (0.6%)	2 (1.2%)	
Injection site haematoma	1 (0.6%)	1 (0.6%)	0	0	1 (0.6%)	
Injection site rash	1 (0.6%)	1 (0.6%)	0	0	1 (0.6%)	
Malaise	1 (0.6%)	1 (0.6%)	0	0	1 (0.6%)	
Mucosal dryness	1 (0.6%)	1 (0.6%)	0	0	1 (0.6%)	
Mucosal inflammation	1 (0.6%)	1 (0.6%)	0	0	1 (0.6%)	
Skin and subcutaneous tissue disorders	72 (44.2%)	75 (46.0%)	7 (21.9%)	12 (7.5%)	77 (47.2%)	
Pruritus	46 (28.2%)	49 (30.1%)	0	5 (3.1%)	53 (32.5%)	
Dry skin	26 (16.0%)	27 (16.6%)	0	1 (0.6%)	28 (17.2%)	
Rash	17 (10.4%)	17 (10.4%)	1 (3.1%)	2 (1.3%)	18 (11.0%)	
Alopecia	6 (3.7%)	14 (8.6%)	4 (12.5%)	3 (1.9%)	17 (10.4%)	
Erythema	5 (3.1%)	5 (3.1%)	0	0	5 (3.1%)	
Dermatitis	3 (1.8%)	3 (1.8%)	0	0	3 (1.8%)	
Eczema	2 (1.2%)	2 (1.2%)	0	0	2 (1.2%)	
Generalised erythema	1 (0.6%)	2 (1.2%)	1 (3.1%)	0	2 (1.2%)	
Hyperhidrosis	2 (1.2%)	2 (1.2%)	0	0	2 (1.2%)	
Psoriasis	1 (0.6%)	1 (0.6%)	0	1 (0.6%)	2 (1.2%)	
Dermatosis	1 (0.6%)	1 (0.6%)	0	0	1 (0.6%)	
Nail discolouration	1 (0.6%)	1 (0.6%)	0	0	1 (0.6%)	
Night sweats	0	0	0	1 (0.6%)	1 (0.6%)	
Pruritus generalised	1 (0.6%)	1 (0.6%)	0	0	1 (0.6%)	
Rash erythematous	1 (0.6%)	1 (0.6%)	0	0	1 (0.6%)	
Rash macular	1 (0.6%)	1 (0.6%)	0	0	1 (0.6%)	
Rash maculo-papular	1 (0.6%)	1 (0.6%)	0	0	1 (0.6%)	
Rash papular	0	1 (0.6%)	1 (3.1%)	0	1 (0.6%)	
Rash pruritic	1 (0.6%)	1 (0.6%)	0	0	1 (0.6%)	
Skin discolouration	1 (0.6%)	1 (0.6%)	0	0	1 (0.6%)	
Skin fissures	1 (0.6%)	1 (0.6%)	0	0	1 (0.6%)	
Skin irritation	1 (0.6%)	1 (0.6%)	0	0	1 (0.6%)	
Skin mass	1 (0.6%)	1 (0.6%)	0	0	1 (0.6%)	
Solar dermatitis	1 (0.6%)	1 (0.6%)	0	0	1 (0.6%)	
Toxic skin eruption	0	1 (0.6%)	1 (3.1%)	0	1 (0.6%)	
Gastrointestinal disorders	50 (30.7%)	50 (30.7%)	1 (3.1%)	1 (0.6%)	50 (30.7%)	
Nausea	13 (8.0%)	13 (8.0%)	0	0	13 (8.0%)	
Diarrhoea	12 (7.4%)	12 (7.4%)	0	0	12 (7.4%)	
Abdominal pain upper	6 (3.7%)	7 (4.3%)	0	0	7 (4.3%)	
Dyspepsia	7 (4.3%)	7 (4.3%)	0	0	7 (4.3%)	
Dry mouth	5 (3.1%)	6 (3.7%)	1 (3.1%)	0	6 (3.7%)	
Gastrooesophageal reflux disease	6 (3.7%)	6 (3.7%)	0	0	6 (3.7%)	
Vomiting	6 (3.7%)	6 (3.7%)	0	0	6 (3.7%)	
Constipation	4 (2.5%)	4 (2.5%)	0	0	4 (2.5%)	
Abdominal pain	3 (1.8%)	3 (1.8%)	0	0	3 (1.8%)	
Anal pruritus	2 (1.2%)	2 (1.2%)	0	0	2 (1.2%)	
Dysphagia	2 (1.2%)	2 (1.2%)	0	0	2 (1.2%)	
Gingival bleeding	2 (1.2%)	2 (1.2%)	0	0	2 (1.2%)	
Mouth ulceration	2 (1.2%)	2 (1.2%)	0	0	2 (1.2%)	
Toothache	2 (1.2%)	2 (1.2%)	0	0	2 (1.2%)	
Abdominal distension	1 (0.6%)	1 (0.6%)	0	0	1 (0.6%)	
Cheilitis	1 (0.6%)	1 (0.6%)	0	0	1 (0.6%)	
Gastritis	1 (0.6%)	1 (0.6%)	0	1 (0.6%)	1 (0.6%)	
Gingival pain	1 (0.6%)	1 (0.6%)	0	0	1 (0.6%)	
Stomatitis	1 (0.6%)	1 (0.6%)	0	0	1 (0.6%)	
Tongue ulceration	1 (0.6%)	1 (0.6%)	0	0	1 (0.6%)	
Nervous system disorders	42 (25.8%)	45 (27.6%)	2 (6.3%)	3 (1.9%)	44 (27.0%)	
Headache	29 (17.8%)	31 (19.0%)	1 (3.1%)	1 (0.6%)	31 (19.0%)	
Dizziness	9 (5.5%)	9 (5.5%)	0	1 (0.6%)	10 (6.1%)	
Dysgeusia	9 (5.5%)	9 (5.5%)	0	0	9 (5.5%)	
Disturbance in attention	4 (2.5%)	4 (2.5%)	0	0	4 (2.5%)	
Sciatica	1 (0.6%)	2 (1.2%)	0	1 (0.6%)	3 (1.8%)	
Dizziness postural	1 (0.6%)	1 (0.6%)	0	1 (0.6%)	2 (1.2%)	
Amnesia	0	1 (0.6%)	1 (3.1%)	0	1 (0.6%)	
Carpal tunnel syndrome	0	0	0	1 (0.6%)	1 (0.6%)	
Epilepsy	0	1 (0.6%)	0	0	1 (0.6%)	
Hyperaesthesia	1 (0.6%)	1 (0.6%)	0	0	1 (0.6%)	
Migraine	1 (0.6%)	1 (0.6%)	0	0	1 (0.6%)	
Paraesthesia	1 (0.6%)	1 (0.6%)	0	0	1 (0.6%)	
Poor quality sleep	1 (0.6%)	1 (0.6%)	0	0	1 (0.6%)	
Presyncope	1 (0.6%)	1 (0.6%)	0	0	1 (0.6%)	
Somnolence	1 (0.6%)	1 (0.6%)	0	0	1 (0.6%)	
Tremor	0	0	0	1 (0.6%)	1 (0.6%)	
Psychiatric disorders	37 (22.7%)	37 (22.7%)	0	1 (0.6%)	38 (23.3%)	
Insomnia	21 (12.9%)	22 (13.5%)	0	0	22 (13.5%)	
Depression	9 (5.5%)	9 (5.5%)	0	1 (0.6%)	10 (6.1%)	
Sleep disorder	7 (4.3%)	7 (4.3%)	0	0	7 (4.3%)	
Anxiety	5 (3.1%)	5 (3.1%)	0	0	5 (3.1%)	
Depressed mood	4 (2.5%)	4 (2.5%)	0	0	4 (2.5%)	
Affect lability	2 (1.2%)	2 (1.2%)	0	0	2 (1.2%)	
Mood altered	2 (1.2%)	2 (1.2%)	0	0	2 (1.2%)	
Aggression	1 (0.6%)	1 (0.6%)	0	0	1 (0.6%)	
Emotional disorder	1 (0.6%)	1 (0.6%)	0	0	1 (0.6%)	
Middle insomnia	1 (0.6%)	1 (0.6%)	0	0	1 (0.6%)	
Mood swings	1 (0.6%)	1 (0.6%)	0	0	1 (0.6%)	
Nicotine dependence	1 (0.6%)	1 (0.6%)	0	0	1 (0.6%)	
Stress	1 (0.6%)	1 (0.6%)	0	0	1 (0.6%)	
Musculoskeletal and connective tissue disorders	35 (21.5%)	35 (21.5%)	0	4 (2.5%)	36 (22.1%)	
Arthralgia	15 (9.2%)	15 (9.2%)	0	2 (1.3%)	17 (10.4%)	
Myalgia	12 (7.4%)	12 (7.4%)	0	1 (0.6%)	13 (8.0%)	
Back pain	6 (3.7%)	6 (3.7%)	0	1 (0.6%)	7 (4.3%)	
Muscle spasms	3 (1.8%)	3 (1.8%)	0	0	3 (1.8%)	
Musculoskeletal pain	1 (0.6%)	1 (0.6%)	0	1 (0.6%)	2 (1.2%)	
Sensation of heaviness	1 (0.6%)	2 (1.2%)	0	0	2 (1.2%)	
Axillary mass	1 (0.6%)	1 (0.6%)	0	0	1 (0.6%)	
Growing pains	1 (0.6%)	1 (0.6%)	0	0	1 (0.6%)	
Muscle twitching	1 (0.6%)	1 (0.6%)	0	0	1 (0.6%)	
Musculoskeletal stiffness	1 (0.6%)	1 (0.6%)	0	0	1 (0.6%)	
Neck pain	1 (0.6%)	1 (0.6%)	0	0	1 (0.6%)	
Rheumatic disorder	1 (0.6%)	1 (0.6%)	0	0	1 (0.6%)	
Respiratory, thoracic and mediastinal disorders	26 (16.0%)	27 (16.6%)	0	2 (1.3%)	28 (17.2%)	
Dyspnoea	13 (8.0%)	13 (8.0%)	0	0	13 (8.0%)	
Cough	8 (4.9%)	8 (4.9%)	0	1 (0.6%)	9 (5.5%)	
Dyspnoea exertional	5 (3.1%)	5 (3.1%)	0	1 (0.6%)	6 (3.7%)	
Epistaxis	4 (2.5%)	4 (2.5%)	0	0	4 (2.5%)	
Asthma	0	1 (0.6%)	0	0	1 (0.6%)	
Oropharyngeal pain	1 (0.6%)	1 (0.6%)	0	0	1 (0.6%)	
Rhinitis allergic	0	1 (0.6%)	0	0	1 (0.6%)	
Infections and infestations	17 (10.4%)	20 (12.3%)	3 (9.4%)	2 (1.3%)	21 (12.9%)	
Bronchitis	3 (1.8%)	3 (1.8%)	0	0	3 (1.8%)	
Nasopharyngitis	2 (1.2%)	2 (1.2%)	0	1 (0.6%)	3 (1.8%)	
Oral candidiasis	2 (1.2%)	3 (1.8%)	1 (3.1%)	0	3 (1.8%)	
Influenza	2 (1.2%)	2 (1.2%)	0	0	2 (1.2%)	
Urinary tract infection	0	2 (1.2%)	1 (3.1%)	0	2 (1.2%)	
Cystitis	1 (0.6%)	1 (0.6%)	0	0	1 (0.6%)	
Ear infection	0	1 (0.6%)	1 (3.1%)	0	1 (0.6%)	
Gingivitis	1 (0.6%)	1 (0.6%)	0	0	1 (0.6%)	
Herpes simplex	1 (0.6%)	1 (0.6%)	0	0	1 (0.6%)	
Orchitis	0	1 (0.6%)	0	0	1 (0.6%)	
Otitis media	1 (0.6%)	1 (0.6%)	0	0	1 (0.6%)	
Respiratory tract infection	1 (0.6%)	1 (0.6%)	0	0	1 (0.6%)	
Rhinitis	1 (0.6%)	1 (0.6%)	0	0	1 (0.6%)	
Tooth abscess	1 (0.6%)	1 (0.6%)	0	0	1 (0.6%)	
Tooth infection	0	0	0	1 (0.6%)	1 (0.6%)	
Upper respiratory tract infection	1 (0.6%)	1 (0.6%)	0	0	1 (0.6%)	
Vulvovaginal candidiasis	1 (0.6%)	1 (0.6%)	0	0	1 (0.6%)	
Metabolism and nutrition disorders	20 (12.3%)	19 (11.7%)	0	1 (0.6%)	20 (12.3%)	
Decreased appetite	18 (11.0%)	18 (11.0%)	0	0	18 (11.0%)	
Hyperamylasaemia	1 (0.6%)	1 (0.6%)	0	0	1 (0.6%)	
Hyperproteinaemia	0	0	0	1 (0.6%)	1 (0.6%)	
Increased appetite	1 (0.6%)	1 (0.6%)	0	0	1 (0.6%)	
Blood and lymphatic system disorders	12 (7.4%)	13 (8.0%)	2 (6.3%)	0	13 (8.0%)	
Anaemia	11 (6.7%)	12 (7.4%)	2 (6.3%)	0	12 (7.4%)	
Neutropenia	7 (4.3%)	7 (4.3%)	0	0	7 (4.3%)	
Leukopenia	6 (3.7%)	6 (3.7%)	0	0	6 (3.7%)	
Thrombocytopenia	4 (2.5%)	4 (2.5%)	0	0	4 (2.5%)	
Lymphadenopathy	0	1 (0.6%)	0	0	1 (0.6%)	
Lymphopenia	1 (0.6%)	1 (0.6%)	0	0	1 (0.6%)	
Ear and labyrinth disorders	13 (8.0%)	13 (8.0%)	0	0	13 (8.0%)	
Vertigo	7 (4.3%)	7 (4.3%)	0	0	7 (4.3%)	
Tinnitus	4 (2.5%)	4 (2.5%)	0	0	4 (2.5%)	
Deafness	1 (0.6%)	1 (0.6%)	0	0	1 (0.6%)	
Ear discomfort	1 (0.6%)	1 (0.6%)	0	0	1 (0.6%)	
Hypoacusis	1 (0.6%)	1 (0.6%)	0	0	1 (0.6%)	
Eye disorders	10 (6.1%)	11 (6.7%)	0	0	11 (6.7%)	
Dry eye	2 (1.2%)	2 (1.2%)	0	0	2 (1.2%)	
Chalazion	0	1 (0.6%)	0	0	1 (0.6%)	
Conjunctival irritation	1 (0.6%)	1 (0.6%)	0	0	1 (0.6%)	
Conjunctival ulcer	1 (0.6%)	1 (0.6%)	0	0	1 (0.6%)	
Eye irritation	1 (0.6%)	1 (0.6%)	0	0	1 (0.6%)	
Glare	1 (0.6%)	1 (0.6%)	0	0	1 (0.6%)	
Ocular hyperaemia	1 (0.6%)	1 (0.6%)	0	0	1 (0.6%)	
Photophobia	1 (0.6%)	1 (0.6%)	0	0	1 (0.6%)	
Retinal pigmentation	1 (0.6%)	1 (0.6%)	0	0	1 (0.6%)	
Vision blurred	1 (0.6%)	1 (0.6%)	0	0	1 (0.6%)	
Investigations	10 (6.1%)	10 (6.1%)	0	0	10 (6.1%)	
Blood bilirubin increased	5 (3.1%)	5 (3.1%)	0	0	5 (3.1%)	
Weight decreased	4 (2.5%)	4 (2.5%)	0	0	4 (2.5%)	
Body temperature increased	1 (0.6%)	1 (0.6%)	0	0	1 (0.6%)	
General physical condition abnormal	1 (0.6%)	1 (0.6%)	0	0	1 (0.6%)	
Haemoglobin decreased	1 (0.6%)	1 (0.6%)	0	0	1 (0.6%)	
Renal and urinary disorders	6 (3.7%)	6 (3.7%)	0	0	6 (3.7%)	
Chromaturia	2 (1.2%)	2 (1.2%)	0	0	2 (1.2%)	
Cystitis-like symptom	1 (0.6%)	1 (0.6%)	0	0	1 (0.6%)	
Pollakiuria	1 (0.6%)	1 (0.6%)	0	0	1 (0.6%)	
Proteinuria	1 (0.6%)	1 (0.6%)	0	0	1 (0.6%)	
Renal colic	1 (0.6%)	1 (0.6%)	0	0	1 (0.6%)	
Cardiac disorders	4 (2.5%)	4 (2.5%)	0	0	4 (2.5%)	
Palpitations	2 (1.2%)	2 (1.2%)	0	0	2 (1.2%)	
Tachycardia	2 (1.2%)	2 (1.2%)	0	0	2 (1.2%)	
Vascular disorders	4 (2.5%)	4 (2.5%)	0	1 (0.6%)	4 (2.5%)	
Haematoma	2 (1.2%)	2 (1.2%)	0	0	2 (1.2%)	
Hypertension	0	1 (0.6%)	0	1 (0.6%)	2 (1.2%)	
Hot flush	1 (0.6%)	1 (0.6%)	0	0	1 (0.6%)	
Peripheral coldness	1 (0.6%)	1 (0.6%)	0	0	1 (0.6%)	
Reproductive system and breast disorders	3 (1.8%)	3 (1.8%)	0	0	3 (1.8%)	
Metrorrhagia	1 (0.6%)	1 (0.6%)	0	0	1 (0.6%)	
Pelvic discomfort	1 (0.6%)	1 (0.6%)	0	0	1 (0.6%)	
Vaginal discharge	1 (0.6%)	1 (0.6%)	0	0	1 (0.6%)	
Endocrine disorders	1 (0.6%)	2 (1.2%)	0	0	2 (1.2%)	
Hyperthyroidism	0	1 (0.6%)	0	0	1 (0.6%)	
Hypothyroidism	1 (0.6%)	1 (0.6%)	0	0	1 (0.6%)	
Hepatobiliary disorders	1 (0.6%)	1 (0.6%)	0	0	1 (0.6%)	
Hyperbilirubinaemia	1 (0.6%)	1 (0.6%)	0	0	1 (0.6%)	
Injury, poisoning and procedural complications	1 (0.6%)	1 (0.6%)	0	0	1 (0.6%)	
Joint injury	1 (0.6%)	1 (0.6%)	0	0	1 (0.6%)	
Any Grade 2 AE	52 (31.9%)	53 (32.5%)	7 (21.9%)	9 (5.6%)	54 (33.1%)	
General disorders and administration site conditions	36 (22.1%)	36 (22.1%)	0	0	36 (22.1%)	
Asthenia	15 (9.2%)	15 (9.2%)	0	0	15 (9.2%)	
Fatigue	12 (7.4%)	12 (7.4%)	0	0	12 (7.4%)	
Influenza like illness	10 (6.1%)	10 (6.1%)	0	0	10 (6.1%)	
Pyrexia	4 (2.5%)	4 (2.5%)	0	0	4 (2.5%)	
Irritability	3 (1.8%)	3 (1.8%)	0	0	3 (1.8%)	
Injection site erythema	2 (1.2%)	2 (1.2%)	0	0	2 (1.2%)	
Injection site pain	1 (0.6%)	1 (0.6%)	0	0	1 (0.6%)	
Oedema	1 (0.6%)	1 (0.6%)	0	0	1 (0.6%)	
Pain	1 (0.6%)	1 (0.6%)	0	0	1 (0.6%)	
Gastrointestinal disorders	15 (9.2%)	16 (9.8%)	0	1 (0.6%)	17 (10.4%)	
Nausea	4 (2.5%)	4 (2.5%)	0	0	4 (2.5%)	
Dry mouth	3 (1.8%)	3 (1.8%)	0	0	3 (1.8%)	
Diarrhoea	2 (1.2%)	2 (1.2%)	0	0	2 (1.2%)	
Abdominal discomfort	1 (0.6%)	1 (0.6%)	0	0	1 (0.6%)	
Abdominal distension	1 (0.6%)	1 (0.6%)	0	0	1 (0.6%)	
Abdominal pain	1 (0.6%)	1 (0.6%)	0	0	1 (0.6%)	
Abdominal pain upper	1 (0.6%)	1 (0.6%)	0	0	1 (0.6%)	
Constipation	1 (0.6%)	1 (0.6%)	0	0	1 (0.6%)	
Dyspepsia	1 (0.6%)	1 (0.6%)	0	0	1 (0.6%)	
Faecaloma	0	0	0	1 (0.6%)	1 (0.6%)	
Gastric ulcer	1 (0.6%)	1 (0.6%)	0	0	1 (0.6%)	
Haemorrhoids	0	1 (0.6%)	0	0	1 (0.6%)	
Skin and subcutaneous tissue disorders	13 (8.0%)	13 (8.0%)	1 (3.1%)	4 (2.5%)	17 (10.4%)	
Pruritus	6 (3.7%)	6 (3.7%)	0	0	6 (3.7%)	
Rash	5 (3.1%)	5 (3.1%)	1 (3.1%)	1 (0.6%)	6 (3.7%)	
Alopecia	0	0	0	2 (1.3%)	2 (1.2%)	
Dry skin	2 (1.2%)	2 (1.2%)	0	0	2 (1.2%)	
Erythema	0	1 (0.6%)	1 (3.1%)	0	1 (0.6%)	
Psoriasis	1 (0.6%)	1 (0.6%)	0	0	1 (0.6%)	
Rash macular	1 (0.6%)	1 (0.6%)	0	0	1 (0.6%)	
Rosacea	0	0	0	1 (0.6%)	1 (0.6%)	
Skin exfoliation	0	1 (0.6%)	1 (3.1%)	0	1 (0.6%)	
Nervous system disorders	12 (7.4%)	13 (8.0%)	1 (3.1%)	2 (1.3%)	15 (9.2%)	
Headache	10 (6.1%)	10 (6.1%)	0	1 (0.6%)	11 (6.7%)	
Ageusia	1 (0.6%)	1 (0.6%)	0	0	1 (0.6%)	
Dysgeusia	1 (0.6%)	1 (0.6%)	0	0	1 (0.6%)	
Memory impairment	1 (0.6%)	1 (0.6%)	0	0	1 (0.6%)	
Neuralgia	0	0	0	1 (0.6%)	1 (0.6%)	
Sciatica	0	1 (0.6%)	1 (3.1%)	0	1 (0.6%)	
Infections and infestations	10 (6.1%)	12 (7.4%)	1 (3.1%)	3 (1.9%)	14 (8.6%)	
Influenza	2 (1.2%)	2 (1.2%)	0	0	2 (1.2%)	
Acarodermatitis	1 (0.6%)	1 (0.6%)	0	0	1 (0.6%)	
Acute tonsillitis	1 (0.6%)	1 (0.6%)	0	0	1 (0.6%)	
Bronchitis	1 (0.6%)	1 (0.6%)	0	0	1 (0.6%)	
Furuncle	1 (0.6%)	1 (0.6%)	0	0	1 (0.6%)	
Gastroenteritis	1 (0.6%)	1 (0.6%)	0	0	1 (0.6%)	
Helicobacter gastritis	1 (0.6%)	1 (0.6%)	0	0	1 (0.6%)	
Herpes simplex	1 (0.6%)	1 (0.6%)	0	0	1 (0.6%)	
Mastitis	1 (0.6%)	1 (0.6%)	0	0	1 (0.6%)	
Onychomycosis	0	1 (0.6%)	1 (3.1%)	0	1 (0.6%)	
Otitis media	0	0	0	1 (0.6%)	1 (0.6%)	
Pneumonia	0	0	0	1 (0.6%)	1 (0.6%)	
Respiratory tract infection	0	1 (0.6%)	0	0	1 (0.6%)	
Sinusitis	1 (0.6%)	1 (0.6%)	0	0	1 (0.6%)	
Subcutaneous abscess	1 (0.6%)	1 (0.6%)	0	0	1 (0.6%)	
Tooth abscess	0	0	0	1 (0.6%)	1 (0.6%)	
Tracheitis	1 (0.6%)	1 (0.6%)	0	0	1 (0.6%)	
Psychiatric disorders	13 (8.0%)	13 (8.0%)	0	0	13 (8.0%)	
Sleep disorder	6 (3.7%)	6 (3.7%)	0	0	6 (3.7%)	
Depression	2 (1.2%)	3 (1.8%)	1 (3.1%)	0	3 (1.8%)	
Insomnia	3 (1.8%)	3 (1.8%)	0	0	3 (1.8%)	
Anxiety	1 (0.6%)	2 (1.2%)	1 (3.1%)	0	2 (1.2%)	
Mood swings	2 (1.2%)	2 (1.2%)	0	0	2 (1.2%)	
Aggression	1 (0.6%)	1 (0.6%)	0	0	1 (0.6%)	
Anger	1 (0.6%)	1 (0.6%)	0	0	1 (0.6%)	
Emotional disorder	1 (0.6%)	1 (0.6%)	0	0	1 (0.6%)	
Feeling of despair	0	1 (0.6%)	1 (3.1%)	0	1 (0.6%)	
Blood and lymphatic system disorders	10 (6.1%)	12 (7.4%)	2 (6.3%)	0	12 (7.4%)	
Neutropenia	7 (4.3%)	8 (4.9%)	1 (3.1%)	0	8 (4.9%)	
Anaemia	5 (3.1%)	7 (4.3%)	2 (6.3%)	0	7 (4.3%)	
Metabolism and nutrition disorders	7 (4.3%)	8 (4.9%)	0	0	8 (4.9%)	
Decreased appetite	4 (2.5%)	4 (2.5%)	0	0	4 (2.5%)	
Gout	1 (0.6%)	2 (1.2%)	0	0	2 (1.2%)	
Hyperlipasaemia	1 (0.6%)	1 (0.6%)	0	0	1 (0.6%)	
Increased appetite	1 (0.6%)	1 (0.6%)	0	0	1 (0.6%)	
Musculoskeletal and connective tissue disorders	8 (4.9%)	8 (4.9%)	0	0	8 (4.9%)	
Myalgia	4 (2.5%)	4 (2.5%)	0	0	4 (2.5%)	
Arthralgia	2 (1.2%)	2 (1.2%)	0	0	2 (1.2%)	
Back pain	1 (0.6%)	1 (0.6%)	0	0	1 (0.6%)	
Pain in extremity	1 (0.6%)	1 (0.6%)	0	0	1 (0.6%)	
Polyarthritis	1 (0.6%)	1 (0.6%)	0	0	1 (0.6%)	
Respiratory, thoracic and mediastinal disorders	7 (4.3%)	8 (4.9%)	1 (3.1%)	0	8 (4.9%)	
Dyspnoea	3 (1.8%)	3 (1.8%)	0	0	3 (1.8%)	
Asthma	1 (0.6%)	2 (1.2%)	1 (3.1%)	0	2 (1.2%)	
Cough	1 (0.6%)	1 (0.6%)	0	0	1 (0.6%)	
Dyspnoea exertional	1 (0.6%)	1 (0.6%)	0	0	1 (0.6%)	
Laryngeal ulceration	1 (0.6%)	1 (0.6%)	0	0	1 (0.6%)	
Oropharyngeal pain	1 (0.6%)	1 (0.6%)	0	0	1 (0.6%)	
Investigations	8 (4.9%)	8 (4.9%)	0	0	7 (4.3%)	
Haemoglobin decreased	3 (1.8%)	3 (1.8%)	0	0	3 (1.8%)	
Alanine aminotransferase increased	2 (1.2%)	2 (1.2%)	0	0	2 (1.2%)	
Aspartate aminotransferase increased	2 (1.2%)	2 (1.2%)	0	0	2 (1.2%)	
Blood bilirubin increased	2 (1.2%)	2 (1.2%)	0	0	2 (1.2%)	
Gamma-glutamyltransferase increased	2 (1.2%)	2 (1.2%)	0	0	2 (1.2%)	
Blood lactate dehydrogenase increased	1 (0.6%)	1 (0.6%)	0	0	1 (0.6%)	
Lipase increased	0	0	0	1 (0.6%)	1 (0.6%)	
Weight decreased	1 (0.6%)	1 (0.6%)	0	0	1 (0.6%)	
Weight increased	1 (0.6%)	1 (0.6%)	0	0	1 (0.6%)	
Reproductive system and breast disorders	4 (2.5%)	4 (2.5%)	0	1 (0.6%)	5 (3.1%)	
Erectile dysfunction	1 (0.6%)	1 (0.6%)	0	0	1 (0.6%)	
Menorrhagia	0	0	0	1 (0.6%)	1 (0.6%)	
Menstrual disorder	1 (0.6%)	1 (0.6%)	0	0	1 (0.6%)	
Vaginal haemorrhage	1 (0.6%)	1 (0.6%)	0	0	1 (0.6%)	
Vulvovaginal pruritus	1 (0.6%)	1 (0.6%)	0	0	1 (0.6%)	
Eye disorders	2 (1.2%)	2 (1.2%)	0	0	2 (1.2%)	
Visual impairment	1 (0.6%)	1 (0.6%)	0	0	1 (0.6%)	
Vitreous haemorrhage	1 (0.6%)	1 (0.6%)	0	0	1 (0.6%)	
Injury, poisoning and procedural complications	2 (1.2%)	2 (1.2%)	0	0	2 (1.2%)	
Fall	1 (0.6%)	1 (0.6%)	0	0	1 (0.6%)	
Limb injury	1 (0.6%)	1 (0.6%)	0	0	1 (0.6%)	
Cardiac disorders	1 (0.6%)	1 (0.6%)	0	0	1 (0.6%)	
Tachycardia	1 (0.6%)	1 (0.6%)	0	0	1 (0.6%)	
Endocrine disorders	0	1 (0.6%)	1 (3.1%)	0	1 (0.6%)	
Hypothyroidism	0	1 (0.6%)	1 (3.1%)	0	1 (0.6%)	
Hepatobiliary disorders	1 (0.6%)	1 (0.6%)	0	0	1 (0.6%)	
Hyperbilirubinaemia	1 (0.6%)	1 (0.6%)	0	0	1 (0.6%)	
Immune system disorders	0	1 (0.6%)	1 (3.1%)	0	1 (0.6%)	
Seasonal allergy	0	1 (0.6%)	1 (3.1%)	0	1 (0.6%)	
Renal and urinary disorders	1 (0.6%)	1 (0.6%)	0	0	1 (0.6%)	
Urinary incontinence	1 (0.6%)	1 (0.6%)	0	0	1 (0.6%)	
Any Grade 3 AE	26 (16.0%)	28 (17.2%)	2 (6.3%)	4 (2.5%)	31 (19.0%)	
Blood and lymphatic system disorders	14 (8.6%)	14 (8.6%)	0	0	14 (8.6%)	
Neutropenia	13 (8.0%)	13 (8.0%)	0	0	13 (8.0%)	
Thrombocytopenia	1 (0.6%)	1 (0.6%)	0	0	1 (0.6%)	
Investigations	4 (2.5%)	4 (2.5%)	0	2 (1.3%)	6 (3.7%)	
Blood creatine phosphokinase increased	1 (0.6%)	1 (0.6%)	0	1 (0.6%)	2 (1.2%)	
Amylase increased	0	0	0	1 (0.6%)	1 (0.6%)	
Blood bilirubin increased	1 (0.6%)	1 (0.6%)	0	0	1 (0.6%)	
Neutrophil count decreased	1 (0.6%)	1 (0.6%)	0	0	1 (0.6%)	
Weight decreased	1 (0.6%)	1 (0.6%)	0	0	1 (0.6%)	
Psychiatric disorders	2 (1.2%)	3 (1.8%)	1 (3.1%)	3 (1.9%)	5 (3.1%)	
Depression	1 (0.6%)	1 (0.6%)	0	1 (0.6%)	2 (1.2%)	
Psychotic disorder	0	1 (0.6%)	1 (3.1%)	1 (0.6%)	2 (1.2%)	
Alcohol withdrawal syndrome	1 (0.6%)	1 (0.6%)	0	1 (0.6%)	1 (0.6%)	
Schizophrenia, paranoid type	0	0	0	1 (0.6%)	1 (0.6%)	
Ear and labyrinth disorders	1 (0.6%)	1 (0.6%)	0	1 (0.6%)	2 (1.2%)	
Deafness	0	0	0	1 (0.6%)	1 (0.6%)	
Vertigo	1 (0.6%)	1 (0.6%)	0	0	1 (0.6%)	
General disorders and administration site conditions	2 (1.2%)	2 (1.2%)	0	0	2 (1.2%)	
Asthenia	2 (1.2%)	2 (1.2%)	0	0	2 (1.2%)	
Skin and subcutaneous tissue disorders	1 (0.6%)	2 (1.2%)	1 (3.1%)	0	2 (1.2%)	
Rash	1 (0.6%)	2 (1.2%)	1 (3.1%)	0	2 (1.2%)	
Cardiac disorders	1 (0.6%)	1 (0.6%)	0	0	1 (0.6%)	
Cardiomyopathy	0	0	0	1 (0.6%)	1 (0.6%)	
Tachycardia	1 (0.6%)	1 (0.6%)	0	0	1 (0.6%)	
Hepatobiliary disorders	1 (0.6%)	1 (0.6%)	0	0	1 (0.6%)	
Hyperbilirubinaemia	1 (0.6%)	1 (0.6%)	0	0	1 (0.6%)	
Infections and infestations	1 (0.6%)	1 (0.6%)	0	0	1 (0.6%)	
Pericoronitis	1 (0.6%)	1 (0.6%)	0	0	1 (0.6%)	
Metabolism and nutrition disorders	1 (0.6%)	1 (0.6%)	0	0	1 (0.6%)	
Hyponatraemia	1 (0.6%)	1 (0.6%)	0	0	1 (0.6%)	
Musculoskeletal and connective tissue disorders	1 (0.6%)	1 (0.6%)	0	0	1 (0.6%)	
Arthralgia	1 (0.6%)	1 (0.6%)	0	0	1 (0.6%)	
Reproductive system and breast disorders	1 (0.6%)	1 (0.6%)	0	0	1 (0.6%)	
Testicular necrosis	1 (0.6%)	1 (0.6%)	0	0	1 (0.6%)	
Any Grade 4 AE	6 (3.7%)	6 (3.7%)	0	3 (1.9%)	8 (4.9%)	
Blood and lymphatic system disorders	4 (2.5%)	4 (2.5%)	0	0	4 (2.5%)	
Neutropenia	4 (2.5%)	4 (2.5%)	0	0	4 (2.5%)	
Cardiac disorders	0	0	0	1 (0.6%)	1 (0.6%)	
Myocarditis	0	0	0	1 (0.6%)	1 (0.6%)	
Endocrine disorders	0	0	0	1 (0.6%)	1 (0.6%)	
Thyroiditis	0	0	0	1 (0.6%)	1 (0.6%)	
Investigations	0	0	0	1 (0.6%)	1 (0.6%)	
Amylase increased	0	0	0	1 (0.6%)	1 (0.6%)	
Psychiatric disorders	1 (0.6%)	1 (0.6%)	0	0	1 (0.6%)	
Sleep disorder	1 (0.6%)	1 (0.6%)	0	0	1 (0.6%)	
Respiratory, thoracic and mediastinal disorders	1 (0.6%)	1 (0.6%)	0	0	1 (0.6%)	
Dyspnoea	1 (0.6%)	1 (0.6%)	0	0	1 (0.6%)	
Any Grade 3-4 AE	32 (19.6%)	34 (20.9%)	2 (6.3%)	7 (4.4%)	39 (23.9%)	
Blood and lymphatic system disorders	18 (11.0%)	18 (11.0%)	0	0	18 (11.0%)	
Neutropenia	17 (10.4%)	17 (10.4%)	0	0	17 (10.4%)	
Thrombocytopenia	1 (0.6%)	1 (0.6%)	0	0	1 (0.6%)	
Investigations	4 (2.5%)	4 (2.5%)	0	3 (1.9%)	7 (4.3%)	
Amylase increased	0	0	0	2 (1.3%)	2 (1.2%)	
Blood creatine phosphokinase increased	1 (0.6%)	1 (0.6%)	0	1 (0.6%)	2 (1.2%)	
Blood bilirubin increased	1 (0.6%)	1 (0.6%)	0	0	1 (0.6%)	
Neutrophil count decreased	1 (0.6%)	1 (0.6%)	0	0	1 (0.6%)	
Weight decreased	1 (0.6%)	1 (0.6%)	0	0	1 (0.6%)	
Psychiatric disorders	3 (1.8%)	4 (2.5%)	1 (3.1%)	3 (1.9%)	6 (3.7%)	
Depression	1 (0.6%)	1 (0.6%)	0	1 (0.6%)	2 (1.2%)	
Psychotic disorder	0	1 (0.6%)	1 (3.1%)	1 (0.6%)	2 (1.2%)	
Alcohol withdrawal syndrome	1 (0.6%)	1 (0.6%)	0	1 (0.6%)	1 (0.6%)	
Schizophrenia, paranoid type	0	0	0	1 (0.6%)	1 (0.6%)	
Sleep disorder	1 (0.6%)	1 (0.6%)	0	0	1 (0.6%)	
Cardiac disorders	1 (0.6%)	1 (0.6%)	0	1 (0.6%)	2 (1.2%)	
Cardiomyopathy	0	0	0	1 (0.6%)	1 (0.6%)	
Myocarditis	0	0	0	1 (0.6%)	1 (0.6%)	
Tachycardia	1 (0.6%)	1 (0.6%)	0	0	1 (0.6%)	
Ear and labyrinth disorders	1 (0.6%)	1 (0.6%)	0	1 (0.6%)	2 (1.2%)	
Deafness	0	0	0	1 (0.6%)	1 (0.6%)	
Vertigo	1 (0.6%)	1 (0.6%)	0	0	1 (0.6%)	
General disorders and administration site conditions	2 (1.2%)	2 (1.2%)	0	0	2 (1.2%)	
Asthenia	2 (1.2%)	2 (1.2%)	0	0	2 (1.2%)	
Skin and subcutaneous tissue disorders	1 (0.6%)	2 (1.2%)	1 (3.1%)	0	2 (1.2%)	
Rash	1 (0.6%)	2 (1.2%)	1 (3.1%)	0	2 (1.2%)	
Endocrine disorders	0	0	0	1 (0.6%)	1 (0.6%)	
Thyroiditis	0	0	0	1 (0.6%)	1 (0.6%)	
Hepatobiliary disorders	1 (0.6%)	1 (0.6%)	0	0	1 (0.6%)	
Hyperbilirubinaemia	1 (0.6%)	1 (0.6%)	0	0	1 (0.6%)	
Infections and infestations	1 (0.6%)	1 (0.6%)	0	0	1 (0.6%)	
Pericoronitis	1 (0.6%)	1 (0.6%)	0	0	1 (0.6%)	
Metabolism and nutrition disorders	1 (0.6%)	1 (0.6%)	0	0	1 (0.6%)	
Hyponatraemia	1 (0.6%)	1 (0.6%)	0	0	1 (0.6%)	
Musculoskeletal and connective tissue disorders	1 (0.6%)	1 (0.6%)	0	0	1 (0.6%)	
Arthralgia	1 (0.6%)	1 (0.6%)	0	0	1 (0.6%)	
Reproductive system and breast disorders	1 (0.6%)	1 (0.6%)	0	0	1 (0.6%)	
Testicular necrosis	1 (0.6%)	1 (0.6%)	0	0	1 (0.6%)	
Respiratory, thoracic and mediastinal disorders	1 (0.6%)	1 (0.6%)	0	0	1 (0.6%)	
Dyspnoea	1 (0.6%)	1 (0.6%)	0	0	1 (0.6%)	
	
[TSFAE05TDG1ALL.RTF] [TMC435\HPC3014\DBR_FINAL_ANALYSIS\RE_FINAL_ANALYSIS\PROD\TSFAE05TDG1ALL.SAS] 02NOV2015, 11:21	
