# Supplementary material for: An Open-Label Trial of 12-Week Simeprevir plus Peginterferon/Ribavirin (PR) in Treatment-Naïve Patients with Hepatitis C Virus (HCV) Genotype 1 (GT1)
Source: PLoS One. 2016 Jul 18;11(7):e0158526. doi: 10.1371/journal.pone.0158526 (PMC4948848; doi:10.1371/journal.pone.0158526)
Supplement: S1 Dataset — (ZIP) [file pone.0158526.s009.zip › Safety data/tsfae15tdg112.rtf]

TSFAE15TDG112:	Number (pcnt) of Genotype 1 Subjects with Adverse Events of Special/Clinical Interest by Preferred Term, Intent-to-treat, Study TMC435HPC3014, Trt Dur 12 Wks 	
	Simeprevir
12 Wks
150 mg
PR 12/24 	
	SMV + PR 	Ent Trt 	PR Only 	Follow-Up 	Overall 	
Analysis set: intent-to-treat	123	123	2	122	123	
Any AE	117 (95.1%)	117 (95.1%)	0	31 (25.4%)	117 (95.1%)	
PRURITUS (ANY TYPE)	42 (34.1%)	44 (35.8%)	0	4 (3.3%)	47 (38.2%)	
Pruritus	41 (33.3%)	43 (35.0%)	0	4 (3.3%)	46 (37.4%)	
Pruritus generalised	1 (0.8%)	1 (0.8%)	0	0	1 (0.8%)	
Rash pruritic	1 (0.8%)	1 (0.8%)	0	0	1 (0.8%)	
UPPER GI	26 (21.1%)	27 (22.0%)	0	0	27 (22.0%)	
Nausea	15 (12.2%)	15 (12.2%)	0	0	15 (12.2%)	
Abdominal pain upper	6 (4.9%)	7 (5.7%)	0	0	7 (5.7%)	
Dyspepsia	5 (4.1%)	5 (4.1%)	0	0	5 (4.1%)	
Vomiting	4 (3.3%)	4 (3.3%)	0	0	4 (3.3%)	
NEUTRO	25 (20.3%)	25 (20.3%)	0	0	25 (20.3%)	
Neutropenia	24 (19.5%)	24 (19.5%)	0	0	24 (19.5%)	
Neutrophil count decreased	1 (0.8%)	1 (0.8%)	0	0	1 (0.8%)	
RASH (ANY TYPE)	21 (17.1%)	21 (17.1%)	0	3 (2.5%)	22 (17.9%)	
Rash	17 (13.8%)	17 (13.8%)	0	3 (2.5%)	19 (15.4%)	
Erythema	3 (2.4%)	3 (2.4%)	0	0	3 (2.4%)	
Generalised erythema	1 (0.8%)	1 (0.8%)	0	0	1 (0.8%)	
Rash macular	1 (0.8%)	1 (0.8%)	0	0	1 (0.8%)	
DYSPNEA	19 (15.4%)	19 (15.4%)	0	1 (0.8%)	20 (16.3%)	
Dyspnoea	13 (10.6%)	13 (10.6%)	0	0	13 (10.6%)	
Dyspnoea exertional	6 (4.9%)	6 (4.9%)	0	1 (0.8%)	7 (5.7%)	
ANEMIA	14 (11.4%)	14 (11.4%)	0	0	14 (11.4%)	
Anaemia	12 (9.8%)	12 (9.8%)	0	0	12 (9.8%)	
Haemoglobin decreased	2 (1.6%)	2 (1.6%)	0	0	2 (1.6%)	
INCREASED BILIRUBIN	10 (8.1%)	10 (8.1%)	0	0	10 (8.1%)	
Blood bilirubin increased	7 (5.7%)	7 (5.7%)	0	0	7 (5.7%)	
Hyperbilirubinaemia	3 (2.4%)	3 (2.4%)	0	0	3 (2.4%)	
PHOTOSENSITIVITY	1 (0.8%)	1 (0.8%)	0	0	1 (0.8%)	
Solar dermatitis	1 (0.8%)	1 (0.8%)	0	0	1 (0.8%)	
Rash FDA						
Y	27 (22.0%)	27 (22.0%)	0	3 (2.5%)	28 (22.8%)	
Rash	17 (13.8%)	17 (13.8%)	0	3 (2.5%)	19 (15.4%)	
Erythema	3 (2.4%)	3 (2.4%)	0	0	3 (2.4%)	
Eczema	2 (1.6%)	2 (1.6%)	0	0	2 (1.6%)	
Dermatitis	1 (0.8%)	1 (0.8%)	0	0	1 (0.8%)	
Dermatosis	1 (0.8%)	1 (0.8%)	0	0	1 (0.8%)	
Generalised erythema	1 (0.8%)	1 (0.8%)	0	0	1 (0.8%)	
Rash macular	1 (0.8%)	1 (0.8%)	0	0	1 (0.8%)	
Rash pruritic	1 (0.8%)	1 (0.8%)	0	0	1 (0.8%)	
Solar dermatitis	1 (0.8%)	1 (0.8%)	0	0	1 (0.8%)	
	
[TSFAE15TDG112.RTF] [TMC435\HPC3014\DBR_FINAL_ANALYSIS\RE_FINAL_ANALYSIS\PROD\TSFAE15TDG112.SAS] 02NOV2015, 11:19	
