# Supplementary material for: An Open-Label Trial of 12-Week Simeprevir plus Peginterferon/Ribavirin (PR) in Treatment-Naïve Patients with Hepatitis C Virus (HCV) Genotype 1 (GT1)
Source: PLoS One. 2016 Jul 18;11(7):e0158526. doi: 10.1371/journal.pone.0158526 (PMC4948848; doi:10.1371/journal.pone.0158526)
Supplement: S1 Dataset — (ZIP) [file pone.0158526.s009.zip › Safety data/tsfae15tdg1all.rtf]

TSFAE15TDG1ALL:	Number (pcnt) of Genotype 1 Subjects with Adverse Events of Special/Clinical Interest by Preferred Term, Intent-to-treat, Study TMC435HPC3014, Trt Dur 12 Wks 	
	Simeprevir
12 Wks
150 mg
PR 12/24 	
	SMV + PR 	Ent Trt 	PR Only 	Follow-Up 	Overall 	
Analysis set: intent-to-treat	163	163	32	160	163	
Any AE	154 (94.5%)	154 (94.5%)	20 (62.5%)	38 (23.8%)	154 (94.5%)	
PRURITUS (ANY TYPE)	53 (32.5%)	56 (34.4%)	0	5 (3.1%)	60 (36.8%)	
Pruritus	52 (31.9%)	55 (33.7%)	0	5 (3.1%)	59 (36.2%)	
Pruritus generalised	1 (0.6%)	1 (0.6%)	0	0	1 (0.6%)	
Rash pruritic	1 (0.6%)	1 (0.6%)	0	0	1 (0.6%)	
UPPER GI	34 (20.9%)	35 (21.5%)	0	0	35 (21.5%)	
Nausea	17 (10.4%)	17 (10.4%)	0	0	17 (10.4%)	
Abdominal pain upper	7 (4.3%)	8 (4.9%)	0	0	8 (4.9%)	
Dyspepsia	8 (4.9%)	8 (4.9%)	0	0	8 (4.9%)	
Vomiting	6 (3.7%)	6 (3.7%)	0	0	6 (3.7%)	
RASH (ANY TYPE)	31 (19.0%)	34 (20.9%)	6 (18.8%)	3 (1.9%)	35 (21.5%)	
Rash	23 (14.1%)	24 (14.7%)	3 (9.4%)	3 (1.9%)	26 (16.0%)	
Erythema	5 (3.1%)	6 (3.7%)	1 (3.1%)	0	6 (3.7%)	
Generalised erythema	1 (0.6%)	2 (1.2%)	1 (3.1%)	0	2 (1.2%)	
Rash macular	2 (1.2%)	2 (1.2%)	0	0	2 (1.2%)	
Rash erythematous	1 (0.6%)	1 (0.6%)	0	0	1 (0.6%)	
Rash maculo-papular	1 (0.6%)	1 (0.6%)	0	0	1 (0.6%)	
Rash papular	0	1 (0.6%)	1 (3.1%)	0	1 (0.6%)	
Skin exfoliation	0	1 (0.6%)	1 (3.1%)	0	1 (0.6%)	
Toxic skin eruption	0	1 (0.6%)	1 (3.1%)	0	1 (0.6%)	
NEUTRO	32 (19.6%)	33 (20.2%)	1 (3.1%)	0	33 (20.2%)	
Neutropenia	31 (19.0%)	32 (19.6%)	1 (3.1%)	0	32 (19.6%)	
Neutrophil count decreased	1 (0.6%)	1 (0.6%)	0	0	1 (0.6%)	
ANEMIA	20 (12.3%)	23 (14.1%)	4 (12.5%)	0	23 (14.1%)	
Anaemia	16 (9.8%)	19 (11.7%)	4 (12.5%)	0	19 (11.7%)	
Haemoglobin decreased	4 (2.5%)	4 (2.5%)	0	0	4 (2.5%)	
DYSPNEA	23 (14.1%)	23 (14.1%)	0	1 (0.6%)	24 (14.7%)	
Dyspnoea	17 (10.4%)	17 (10.4%)	0	0	17 (10.4%)	
Dyspnoea exertional	6 (3.7%)	6 (3.7%)	0	1 (0.6%)	7 (4.3%)	
INCREASED BILIRUBIN	11 (6.7%)	11 (6.7%)	0	0	11 (6.7%)	
Blood bilirubin increased	8 (4.9%)	8 (4.9%)	0	0	8 (4.9%)	
Hyperbilirubinaemia	3 (1.8%)	3 (1.8%)	0	0	3 (1.8%)	
PHOTOSENSITIVITY	1 (0.6%)	1 (0.6%)	0	0	1 (0.6%)	
Solar dermatitis	1 (0.6%)	1 (0.6%)	0	0	1 (0.6%)	
Rash FDA						
Y	39 (23.9%)	42 (25.8%)	6 (18.8%)	3 (1.9%)	43 (26.4%)	
Rash	23 (14.1%)	24 (14.7%)	3 (9.4%)	3 (1.9%)	26 (16.0%)	
Erythema	5 (3.1%)	6 (3.7%)	1 (3.1%)	0	6 (3.7%)	
Dermatitis	3 (1.8%)	3 (1.8%)	0	0	3 (1.8%)	
Eczema	2 (1.2%)	2 (1.2%)	0	0	2 (1.2%)	
Generalised erythema	1 (0.6%)	2 (1.2%)	1 (3.1%)	0	2 (1.2%)	
Rash macular	2 (1.2%)	2 (1.2%)	0	0	2 (1.2%)	
Dermatosis	1 (0.6%)	1 (0.6%)	0	0	1 (0.6%)	
Rash erythematous	1 (0.6%)	1 (0.6%)	0	0	1 (0.6%)	
Rash maculo-papular	1 (0.6%)	1 (0.6%)	0	0	1 (0.6%)	
Rash papular	0	1 (0.6%)	1 (3.1%)	0	1 (0.6%)	
Rash pruritic	1 (0.6%)	1 (0.6%)	0	0	1 (0.6%)	
Skin exfoliation	0	1 (0.6%)	1 (3.1%)	0	1 (0.6%)	
Solar dermatitis	1 (0.6%)	1 (0.6%)	0	0	1 (0.6%)	
Toxic skin eruption	0	1 (0.6%)	1 (3.1%)	0	1 (0.6%)	
	
[TSFAE15TDG1ALL.RTF] [TMC435\HPC3014\DBR_FINAL_ANALYSIS\RE_FINAL_ANALYSIS\PROD\TSFAE15TDG1ALL.SAS] 02NOV2015, 11:20	
