# Supplementary material for: An Open-Label Trial of 12-Week Simeprevir plus Peginterferon/Ribavirin (PR) in Treatment-Naïve Patients with Hepatitis C Virus (HCV) Genotype 1 (GT1)
Source: PLoS One. 2016 Jul 18;11(7):e0158526. doi: 10.1371/journal.pone.0158526 (PMC4948848; doi:10.1371/journal.pone.0158526)
Supplement: S1 Dataset — (ZIP) [file pone.0158526.s009.zip › Safety data/tsfae15tdg1gt12.rtf]

TSFAE15TDG1GT12:	Number (pcnt) of Genotype 1 Subjects with Adverse Events of Special/Clinical Interest by Preferred Term, Intent-to-treat, Study TMC435HPC3014, Trt Dur gt 12 Wks 	
	Simeprevir
12 Wks
150 mg
PR 12/24 	
	SMV + PR 	Ent Trt 	PR Only 	Follow-Up 	Overall 	
Analysis set: intent-to-treat	40	40	30	38	40	
Any AE	37 (92.5%)	37 (92.5%)	20 (66.7%)	7 (18.4%)	37 (92.5%)	
RASH (ANY TYPE)	10 (25.0%)	13 (32.5%)	6 (20.0%)	0	13 (32.5%)	
Rash	6 (15.0%)	7 (17.5%)	3 (10.0%)	0	7 (17.5%)	
Erythema	2 (5.0%)	3 (7.5%)	1 (3.3%)	0	3 (7.5%)	
Generalised erythema	0	1 (2.5%)	1 (3.3%)	0	1 (2.5%)	
Rash erythematous	1 (2.5%)	1 (2.5%)	0	0	1 (2.5%)	
Rash macular	1 (2.5%)	1 (2.5%)	0	0	1 (2.5%)	
Rash maculo-papular	1 (2.5%)	1 (2.5%)	0	0	1 (2.5%)	
Rash papular	0	1 (2.5%)	1 (3.3%)	0	1 (2.5%)	
Skin exfoliation	0	1 (2.5%)	1 (3.3%)	0	1 (2.5%)	
Toxic skin eruption	0	1 (2.5%)	1 (3.3%)	0	1 (2.5%)	
PRURITUS (ANY TYPE)	11 (27.5%)	12 (30.0%)	0	1 (2.6%)	13 (32.5%)	
Pruritus	11 (27.5%)	12 (30.0%)	0	1 (2.6%)	13 (32.5%)	
ANEMIA	6 (15.0%)	9 (22.5%)	4 (13.3%)	0	9 (22.5%)	
Anaemia	4 (10.0%)	7 (17.5%)	4 (13.3%)	0	7 (17.5%)	
Haemoglobin decreased	2 (5.0%)	2 (5.0%)	0	0	2 (5.0%)	
NEUTRO	7 (17.5%)	8 (20.0%)	1 (3.3%)	0	8 (20.0%)	
Neutropenia	7 (17.5%)	8 (20.0%)	1 (3.3%)	0	8 (20.0%)	
UPPER GI	8 (20.0%)	8 (20.0%)	0	0	8 (20.0%)	
Dyspepsia	3 (7.5%)	3 (7.5%)	0	0	3 (7.5%)	
Nausea	2 (5.0%)	2 (5.0%)	0	0	2 (5.0%)	
Vomiting	2 (5.0%)	2 (5.0%)	0	0	2 (5.0%)	
Abdominal pain upper	1 (2.5%)	1 (2.5%)	0	0	1 (2.5%)	
DYSPNEA	4 (10.0%)	4 (10.0%)	0	0	4 (10.0%)	
Dyspnoea	4 (10.0%)	4 (10.0%)	0	0	4 (10.0%)	
INCREASED BILIRUBIN	1 (2.5%)	1 (2.5%)	0	0	1 (2.5%)	
Blood bilirubin increased	1 (2.5%)	1 (2.5%)	0	0	1 (2.5%)	
Rash FDA						
Y	12 (30.0%)	15 (37.5%)	6 (20.0%)	0	15 (37.5%)	
Rash	6 (15.0%)	7 (17.5%)	3 (10.0%)	0	7 (17.5%)	
Erythema	2 (5.0%)	3 (7.5%)	1 (3.3%)	0	3 (7.5%)	
Dermatitis	2 (5.0%)	2 (5.0%)	0	0	2 (5.0%)	
Generalised erythema	0	1 (2.5%)	1 (3.3%)	0	1 (2.5%)	
Rash erythematous	1 (2.5%)	1 (2.5%)	0	0	1 (2.5%)	
Rash macular	1 (2.5%)	1 (2.5%)	0	0	1 (2.5%)	
Rash maculo-papular	1 (2.5%)	1 (2.5%)	0	0	1 (2.5%)	
Rash papular	0	1 (2.5%)	1 (3.3%)	0	1 (2.5%)	
Skin exfoliation	0	1 (2.5%)	1 (3.3%)	0	1 (2.5%)	
Toxic skin eruption	0	1 (2.5%)	1 (3.3%)	0	1 (2.5%)	
	
[TSFAE15TDG1GT12.RTF] [TMC435\HPC3014\DBR_FINAL_ANALYSIS\RE_FINAL_ANALYSIS\PROD\TSFAE15TDG1GT12.SAS] 02NOV2015, 11:20	
