# Supplementary material for: An Open-Label Trial of 12-Week Simeprevir plus Peginterferon/Ribavirin (PR) in Treatment-Naïve Patients with Hepatitis C Virus (HCV) Genotype 1 (GT1)
Source: PLoS One. 2016 Jul 18;11(7):e0158526. doi: 10.1371/journal.pone.0158526 (PMC4948848; doi:10.1371/journal.pone.0158526)
Supplement: S1 Dataset — (ZIP) [file pone.0158526.s009.zip › Safety data/TSFLAB01-GT.rtf]

TSFLAB01-GT:	Actual Laboratory Parameters values by Analysis Timepoint (Entire Treatment Phase); Intent-to-treat (Study TMC435HPC3014)	
Simeprevir
12 Wks
150 mg
PR 12/24 	
	 Genotype 1 	
	 12 Wks
(N=123) 	 >12 Wks
(N=40) 	 All subjects
(N=163) 	
Hemoglobin (g/L)				
Baseline				
N	123	40	163	
Mean	148.85	152.93	149.85	
Std. Err.	1.112	2.117	0.993	
Std. Dev.	12.335	13.387	12.682	
95% C.I.	(146.644; 151.047)	(148.644; 157.206)	(147.885; 151.808)	
Minimum	119.0	121.0	119.0	
First quartile	140.00	142.50	140.00	
Median	147.00	154.50	149.00	
Third quartile	157.00	161.50	159.00	
Maximum	180.0	179.0	180.0	
Week 01				
N	118	34	152	
Mean	148.11	153.32	149.28	
Std. Err.	1.143	2.475	1.056	
Std. Dev.	12.411	14.432	13.024	
95% C.I.	(145.847; 150.373)	(148.288; 158.359)	(147.189; 151.363)	
Minimum	120.0	117.0	117.0	
First quartile	139.00	141.00	139.00	
Median	146.50	156.50	148.50	
Third quartile	158.00	165.00	159.00	
Maximum	176.0	178.0	178.0	
Week 02				
N	105	34	139	
Mean	137.93	137.62	137.86	
Std. Err.	1.558	2.735	1.349	
Std. Dev.	15.967	15.945	15.904	
95% C.I.	(134.843; 141.023)	(132.054; 143.181)	(135.189; 140.523)	
Minimum	106.0	102.0	102.0	
First quartile	127.00	125.00	126.00	
Median	138.00	139.00	138.00	
Third quartile	150.00	149.00	150.00	
Maximum	179.0	171.0	179.0	
Week 04				
N	121	39	160	
Mean	129.00	129.00	129.00	
Std. Err.	1.501	3.203	1.372	
Std. Dev.	16.507	20.003	17.357	
95% C.I.	(126.029; 131.971)	(122.516; 135.484)	(126.290; 131.710)	
Minimum	92.0	96.0	92.0	
First quartile	116.00	108.00	115.00	
Median	128.00	129.00	128.50	
Third quartile	140.00	140.00	140.00	
Maximum	177.0	170.0	177.0	
Week 08				
N	122	34	156	
Mean	125.02	124.59	124.93	
Std. Err.	1.277	3.501	1.251	
Std. Dev.	14.100	20.416	15.620	
95% C.I.	(122.497; 127.552)	(117.465; 131.712)	(122.459; 127.400)	
Minimum	94.0	90.0	90.0	
First quartile	116.00	108.00	114.00	
Median	124.00	123.00	124.00	
Third quartile	134.00	146.00	137.00	
Maximum	157.0	160.0	160.0	
Week 12				
N	115	28	143	
Mean	123.12	121.75	122.85	
Std. Err.	1.380	3.597	1.309	
Std. Dev.	14.802	19.031	15.655	
95% C.I.	(120.387; 125.856)	(114.370; 129.130)	(120.265; 125.441)	
Minimum	85.0	93.0	85.0	
First quartile	114.00	106.00	112.00	
Median	123.00	115.50	123.00	
Third quartile	134.00	139.50	135.00	
Maximum	167.0	151.0	167.0	
Week 16				
N	34	28	62	
Mean	136.71	119.18	128.79	
Std. Err.	2.265	3.420	2.260	
Std. Dev.	13.206	18.099	17.795	
95% C.I.	(132.098; 141.314)	(112.161; 126.196)	(124.271; 133.309)	
Minimum	105.0	95.0	95.0	
First quartile	130.00	103.50	114.00	
Median	136.50	114.50	133.00	
Third quartile	147.00	138.00	142.00	
Maximum	158.0	153.0	158.0	
Week 20				
N	0	28	28	
Mean	-	119.11	119.11	
Std. Err.	-	3.483	3.483	
Std. Dev.	-	18.428	18.428	
95% C.I.	-	(111.962; 126.253)	(111.962; 126.253)	
Minimum	-	92.0	92.0	
First quartile	-	105.50	105.50	
Median	-	116.00	116.00	
Third quartile	-	136.50	136.50	
Maximum	-	152.0	152.0	
Week 24				
N	0	27	27	
Mean	-	120.07	120.07	
Std. Err.	-	3.424	3.424	
Std. Dev.	-	17.789	17.789	
95% C.I.	-	(113.037; 127.111)	(113.037; 127.111)	
Minimum	-	91.0	91.0	
First quartile	-	106.00	106.00	
Median	-	123.00	123.00	
Third quartile	-	133.00	133.00	
Maximum	-	148.0	148.0	
Week 28				
N	0	5	5	
Mean	-	124.20	124.20	
Std. Err.	-	9.635	9.635	
Std. Dev.	-	21.545	21.545	
95% C.I.	-	(97.448; 150.952)	(97.448; 150.952)	
Minimum	-	92.0	92.0	
First quartile	-	113.00	113.00	
Median	-	132.00	132.00	
Third quartile	-	142.00	142.00	
Maximum	-	142.0	142.0	
Week 36				
N	0	1	1	
Mean	-	92.00	92.00	
95% C.I.	-	(.; .)	(.; .)	
Minimum	-	92.0	92.0	
First quartile	-	92.00	92.00	
Median	-	92.00	92.00	
Third quartile	-	92.00	92.00	
Maximum	-	92.0	92.0	
Week 48				
N	0	1	1	
Mean	-	89.00	89.00	
95% C.I.	-	(.; .)	(.; .)	
Minimum	-	89.0	89.0	
First quartile	-	89.00	89.00	
Median	-	89.00	89.00	
Third quartile	-	89.00	89.00	
Maximum	-	89.0	89.0	
EOT				
N	123	39	162	
Mean	123.02	123.69	123.19	
Std. Err.	1.271	3.029	1.205	
Std. Dev.	14.097	18.918	15.334	
95% C.I.	(120.508; 125.541)	(117.560; 129.825)	(120.806; 125.564)	
Minimum	85.0	91.0	85.0	
First quartile	114.00	107.00	112.00	
Median	123.00	127.00	123.00	
Third quartile	133.00	140.00	134.00	
Maximum	153.0	157.0	157.0	
Neutrophils and Precursors (x10E9/L)				
Baseline				
N	123	40	163	
Mean	3.81	4.25	3.92	
Std. Err.	0.163	0.359	0.152	
Std. Dev.	1.811	2.269	1.935	
95% C.I.	(3.487; 4.134)	(3.522; 4.974)	(3.619; 4.217)	
Minimum	1.1	1.6	1.1	
First quartile	2.56	2.71	2.62	
Median	3.54	3.71	3.55	
Third quartile	4.49	4.78	4.63	
Maximum	13.0	12.6	13.0	
Week 01				
N	118	34	152	
Mean	2.07	2.56	2.18	
Std. Err.	0.116	0.198	0.101	
Std. Dev.	1.258	1.156	1.250	
95% C.I.	(1.838; 2.297)	(2.157; 2.964)	(1.977; 2.378)	
Minimum	0.6	0.9	0.6	
First quartile	1.28	1.57	1.36	
Median	1.70	2.28	1.82	
Third quartile	2.45	3.54	2.66	
Maximum	7.9	5.3	7.9	
Week 02				
N	104	34	138	
Mean	1.98	2.31	2.06	
Std. Err.	0.105	0.239	0.099	
Std. Dev.	1.069	1.395	1.161	
95% C.I.	(1.767; 2.183)	(1.820; 2.793)	(1.861; 2.252)	
Minimum	0.6	0.5	0.5	
First quartile	1.16	1.32	1.22	
Median	1.69	2.02	1.82	
Third quartile	2.38	2.89	2.62	
Maximum	5.6	6.8	6.8	
Week 04				
N	121	39	160	
Mean	1.77	1.93	1.81	
Std. Err.	0.089	0.166	0.078	
Std. Dev.	0.978	1.038	0.992	
95% C.I.	(1.592; 1.944)	(1.596; 2.269)	(1.653; 1.963)	
Minimum	0.6	0.6	0.6	
First quartile	1.10	1.07	1.10	
Median	1.50	1.85	1.55	
Third quartile	2.09	2.49	2.23	
Maximum	6.6	5.4	6.6	
Week 08				
N	122	34	156	
Mean	1.66	2.16	1.76	
Std. Err.	0.082	0.222	0.081	
Std. Dev.	0.903	1.292	1.018	
95% C.I.	(1.494; 1.818)	(1.705; 2.607)	(1.604; 1.926)	
Minimum	0.5	0.5	0.5	
First quartile	1.05	1.23	1.10	
Median	1.49	1.89	1.51	
Third quartile	1.98	2.82	2.16	
Maximum	6.1	7.2	7.2	
Week 12				
N	115	28	143	
Mean	1.70	1.56	1.67	
Std. Err.	0.089	0.133	0.076	
Std. Dev.	0.958	0.705	0.914	
95% C.I.	(1.518; 1.872)	(1.291; 1.837)	(1.519; 1.821)	
Minimum	0.5	0.5	0.5	
First quartile	0.98	0.97	0.98	
Median	1.47	1.56	1.47	
Third quartile	2.06	2.23	2.10	
Maximum	4.8	3.1	4.8	
Week 16				
N	34	28	62	
Mean	3.36	1.83	2.67	
Std. Err.	0.367	0.220	0.243	
Std. Dev.	2.138	1.164	1.913	
95% C.I.	(2.613; 4.105)	(1.379; 2.282)	(2.183; 3.154)	
Minimum	0.4	0.5	0.4	
First quartile	2.18	1.14	1.63	
Median	2.78	1.71	2.20	
Third quartile	3.60	2.10	3.13	
Maximum	12.5	6.0	12.5	
Week 20				
N	0	28	28	
Mean	-	1.88	1.88	
Std. Err.	-	0.213	0.213	
Std. Dev.	-	1.128	1.128	
95% C.I.	-	(1.444; 2.318)	(1.444; 2.318)	
Minimum	-	0.5	0.5	
First quartile	-	1.07	1.07	
Median	-	1.76	1.76	
Third quartile	-	2.32	2.32	
Maximum	-	5.9	5.9	
Week 24				
N	0	27	27	
Mean	-	1.85	1.85	
Std. Err.	-	0.217	0.217	
Std. Dev.	-	1.126	1.126	
95% C.I.	-	(1.402; 2.293)	(1.402; 2.293)	
Minimum	-	0.5	0.5	
First quartile	-	0.95	0.95	
Median	-	1.54	1.54	
Third quartile	-	2.64	2.64	
Maximum	-	5.1	5.1	
Week 28				
N	0	5	5	
Mean	-	2.69	2.69	
Std. Err.	-	1.022	1.022	
Std. Dev.	-	2.286	2.286	
95% C.I.	-	(-0.146; 5.530)	(-0.146; 5.530)	
Minimum	-	1.2	1.2	
First quartile	-	1.29	1.29	
Median	-	1.73	1.73	
Third quartile	-	2.59	2.59	
Maximum	-	6.7	6.7	
Week 36				
N	0	1	1	
Mean	-	0.77	0.77	
95% C.I.	-	(.; .)	(.; .)	
Minimum	-	0.8	0.8	
First quartile	-	0.77	0.77	
Median	-	0.77	0.77	
Third quartile	-	0.77	0.77	
Maximum	-	0.8	0.8	
Week 48				
N	0	1	1	
Mean	-	0.91	0.91	
95% C.I.	-	(.; .)	(.; .)	
Minimum	-	0.9	0.9	
First quartile	-	0.91	0.91	
Median	-	0.91	0.91	
Third quartile	-	0.91	0.91	
Maximum	-	0.9	0.9	
EOT				
N	123	39	162	
Mean	1.66	1.91	1.72	
Std. Err.	0.080	0.172	0.074	
Std. Dev.	0.893	1.077	0.943	
95% C.I.	(1.496; 1.815)	(1.558; 2.256)	(1.570; 1.863)	
Minimum	0.5	0.5	0.5	
First quartile	0.98	1.08	0.99	
Median	1.49	1.68	1.53	
Third quartile	1.92	2.57	2.15	
Maximum	4.8	5.1	5.1	
Platelets (x10E9/L)				
Baseline				
N	122	40	162	
Mean	243.85	238.15	242.44	
Std. Err.	5.140	11.256	4.751	
Std. Dev.	56.772	71.190	60.465	
95% C.I.	(233.677; 254.028)	(215.382; 260.918)	(233.063; 251.826)	
Minimum	131.0	127.0	127.0	
First quartile	198.00	186.00	196.00	
Median	235.00	223.00	231.00	
Third quartile	286.00	290.50	286.00	
Maximum	388.0	452.0	452.0	
Week 01				
N	117	34	151	
Mean	185.56	187.18	185.93	
Std. Err.	4.388	10.330	4.102	
Std. Dev.	47.466	60.234	50.408	
95% C.I.	(176.873; 194.256)	(166.160; 208.193)	(177.822; 194.033)	
Minimum	86.0	67.0	67.0	
First quartile	146.00	144.00	144.00	
Median	180.00	184.50	181.00	
Third quartile	220.00	228.00	222.00	
Maximum	313.0	328.0	328.0	
Week 02				
N	105	34	139	
Mean	186.12	193.09	187.83	
Std. Err.	5.749	11.693	5.185	
Std. Dev.	58.912	68.183	61.125	
95% C.I.	(174.723; 197.525)	(169.298; 216.878)	(177.576; 198.079)	
Minimum	71.0	60.0	60.0	
First quartile	144.00	148.00	144.00	
Median	176.00	190.00	181.00	
Third quartile	219.00	245.00	225.00	
Maximum	424.0	384.0	424.0	
Week 04				
N	119	39	158	
Mean	187.16	204.59	191.46	
Std. Err.	5.290	12.776	5.094	
Std. Dev.	57.704	79.788	64.034	
95% C.I.	(176.685; 197.635)	(178.726; 230.454)	(181.400; 201.524)	
Minimum	59.0	76.0	59.0	
First quartile	141.00	155.00	143.00	
Median	183.00	180.00	182.00	
Third quartile	222.00	238.00	227.00	
Maximum	340.0	423.0	423.0	
Week 08				
N	119	34	153	
Mean	168.24	189.74	173.01	
Std. Err.	5.107	13.251	4.975	
Std. Dev.	55.715	77.266	61.533	
95% C.I.	(158.121; 178.349)	(162.776; 216.695)	(163.185; 182.841)	
Minimum	48.0	110.0	48.0	
First quartile	126.00	134.00	128.00	
Median	158.00	156.50	158.00	
Third quartile	204.00	220.00	208.00	
Maximum	366.0	432.0	432.0	
Week 12				
N	113	28	141	
Mean	166.32	172.64	167.57	
Std. Err.	4.927	12.935	4.692	
Std. Dev.	52.378	68.443	55.719	
95% C.I.	(156.556; 176.081)	(146.103; 199.182)	(158.297; 176.852)	
Minimum	59.0	99.0	59.0	
First quartile	131.00	121.50	130.00	
Median	160.00	151.50	156.00	
Third quartile	202.00	211.00	202.00	
Maximum	309.0	401.0	401.0	
Week 16				
N	34	28	62	
Mean	228.09	182.04	207.29	
Std. Err.	9.189	13.115	8.249	
Std. Dev.	53.581	69.396	64.950	
95% C.I.	(209.393; 246.783)	(155.127; 208.944)	(190.796; 223.785)	
Minimum	131.0	76.0	76.0	
First quartile	190.00	132.00	153.00	
Median	226.00	162.00	213.50	
Third quartile	270.00	231.00	248.00	
Maximum	345.0	372.0	372.0	
Week 20				
N	0	28	28	
Mean	-	175.32	175.32	
Std. Err.	-	13.583	13.583	
Std. Dev.	-	71.872	71.872	
95% C.I.	-	(147.452; 203.191)	(147.452; 203.191)	
Minimum	-	85.0	85.0	
First quartile	-	131.00	131.00	
Median	-	163.50	163.50	
Third quartile	-	206.50	206.50	
Maximum	-	450.0	450.0	
Week 24				
N	0	27	27	
Mean	-	163.78	163.78	
Std. Err.	-	11.721	11.721	
Std. Dev.	-	60.902	60.902	
95% C.I.	-	(139.686; 187.870)	(139.686; 187.870)	
Minimum	-	86.0	86.0	
First quartile	-	111.00	111.00	
Median	-	150.00	150.00	
Third quartile	-	212.00	212.00	
Maximum	-	352.0	352.0	
Week 28				
N	0	5	5	
Mean	-	177.00	177.00	
Std. Err.	-	13.183	13.183	
Std. Dev.	-	29.479	29.479	
95% C.I.	-	(140.397; 213.603)	(140.397; 213.603)	
Minimum	-	152.0	152.0	
First quartile	-	165.00	165.00	
Median	-	168.00	168.00	
Third quartile	-	172.00	172.00	
Maximum	-	228.0	228.0	
Week 36				
N	0	1	1	
Mean	-	143.00	143.00	
95% C.I.	-	(.; .)	(.; .)	
Minimum	-	143.0	143.0	
First quartile	-	143.00	143.00	
Median	-	143.00	143.00	
Third quartile	-	143.00	143.00	
Maximum	-	143.0	143.0	
Week 48				
N	0	1	1	
Mean	-	149.00	149.00	
95% C.I.	-	(.; .)	(.; .)	
Minimum	-	149.0	149.0	
First quartile	-	149.00	149.00	
Median	-	149.00	149.00	
Third quartile	-	149.00	149.00	
Maximum	-	149.0	149.0	
EOT				
N	122	39	161	
Mean	166.35	166.33	166.35	
Std. Err.	4.716	10.585	4.381	
Std. Dev.	52.090	66.104	55.586	
95% C.I.	(157.016; 175.689)	(144.905; 187.762)	(157.696; 174.999)	
Minimum	59.0	76.0	59.0	
First quartile	131.00	125.00	130.00	
Median	157.00	152.00	156.00	
Third quartile	202.00	200.00	200.00	
Maximum	309.0	450.0	450.0	
Direct Bilirubin (umol/L)				
Baseline				
N	123	40	163	
Mean	2.77	3.45	2.94	
Std. Err.	0.093	0.263	0.098	
Std. Dev.	1.031	1.663	1.246	
95% C.I.	(2.588; 2.956)	(2.918; 3.982)	(2.746; 3.131)	
Minimum	1.0	1.0	1.0	
First quartile	2.00	2.00	2.00	
Median	3.00	3.00	3.00	
Third quartile	3.00	4.00	4.00	
Maximum	6.0	9.0	9.0	
Week 01				
N	120	37	157	
Mean	4.92	5.54	5.06	
Std. Err.	0.235	0.361	0.200	
Std. Dev.	2.578	2.193	2.500	
95% C.I.	(4.451; 5.383)	(4.809; 6.272)	(4.670; 5.458)	
Minimum	1.0	2.0	1.0	
First quartile	3.00	4.00	3.00	
Median	4.00	5.00	4.00	
Third quartile	6.00	7.00	7.00	
Maximum	15.0	10.0	15.0	
Week 02				
N	111	36	147	
Mean	5.14	5.69	5.27	
Std. Err.	0.229	0.349	0.194	
Std. Dev.	2.418	2.095	2.348	
95% C.I.	(4.680; 5.590)	(4.986; 6.403)	(4.889; 5.655)	
Minimum	1.0	3.0	1.0	
First quartile	3.00	4.00	4.00	
Median	5.00	5.00	5.00	
Third quartile	6.00	7.00	7.00	
Maximum	18.0	11.0	18.0	
Week 04				
N	122	39	161	
Mean	5.20	5.03	5.16	
Std. Err.	0.224	0.374	0.192	
Std. Dev.	2.478	2.334	2.438	
95% C.I.	(4.752; 5.641)	(4.269; 5.782)	(4.776; 5.535)	
Minimum	1.0	2.0	1.0	
First quartile	3.00	3.00	3.00	
Median	5.00	5.00	5.00	
Third quartile	6.00	6.00	6.00	
Maximum	18.0	12.0	18.0	
Week 08				
N	123	35	158	
Mean	5.37	4.51	5.18	
Std. Err.	0.243	0.365	0.207	
Std. Dev.	2.696	2.161	2.605	
95% C.I.	(4.893; 5.855)	(3.772; 5.257)	(4.774; 5.593)	
Minimum	2.0	2.0	2.0	
First quartile	3.00	3.00	3.00	
Median	5.00	4.00	5.00	
Third quartile	7.00	6.00	7.00	
Maximum	17.0	10.0	17.0	
Week 12				
N	116	26	142	
Mean	4.94	4.50	4.86	
Std. Err.	0.243	0.389	0.211	
Std. Dev.	2.619	1.985	2.514	
95% C.I.	(4.458; 5.421)	(3.698; 5.302)	(4.442; 5.276)	
Minimum	2.0	2.0	2.0	
First quartile	3.00	3.00	3.00	
Median	4.00	4.00	4.00	
Third quartile	6.00	6.00	6.00	
Maximum	14.0	9.0	14.0	
Week 16				
N	34	29	63	
Mean	1.94	2.90	2.38	
Std. Err.	0.152	0.229	0.146	
Std. Dev.	0.886	1.235	1.156	
95% C.I.	(1.632; 2.250)	(2.427; 3.366)	(2.090; 2.672)	
Minimum	1.0	1.0	1.0	
First quartile	1.00	2.00	2.00	
Median	2.00	3.00	2.00	
Third quartile	2.00	3.00	3.00	
Maximum	4.0	6.0	6.0	
Week 20				
N	0	29	29	
Mean	-	2.69	2.69	
Std. Err.	-	0.244	0.244	
Std. Dev.	-	1.312	1.312	
95% C.I.	-	(2.191; 3.189)	(2.191; 3.189)	
Minimum	-	1.0	1.0	
First quartile	-	2.00	2.00	
Median	-	3.00	3.00	
Third quartile	-	3.00	3.00	
Maximum	-	5.0	5.0	
Week 24				
N	0	28	28	
Mean	-	2.75	2.75	
Std. Err.	-	0.315	0.315	
Std. Dev.	-	1.669	1.669	
95% C.I.	-	(2.103; 3.397)	(2.103; 3.397)	
Minimum	-	1.0	1.0	
First quartile	-	1.00	1.00	
Median	-	3.00	3.00	
Third quartile	-	3.00	3.00	
Maximum	-	7.0	7.0	
Week 28				
N	0	5	5	
Mean	-	1.60	1.60	
Std. Err.	-	0.245	0.245	
Std. Dev.	-	0.548	0.548	
95% C.I.	-	(0.920; 2.280)	(0.920; 2.280)	
Minimum	-	1.0	1.0	
First quartile	-	1.00	1.00	
Median	-	2.00	2.00	
Third quartile	-	2.00	2.00	
Maximum	-	2.0	2.0	
Week 36				
N	0	1	1	
Mean	-	3.00	3.00	
95% C.I.	-	(.; .)	(.; .)	
Minimum	-	3.0	3.0	
First quartile	-	3.00	3.00	
Median	-	3.00	3.00	
Third quartile	-	3.00	3.00	
Maximum	-	3.0	3.0	
Week 48				
N	0	1	1	
Mean	-	2.00	2.00	
95% C.I.	-	(.; .)	(.; .)	
Minimum	-	2.0	2.0	
First quartile	-	2.00	2.00	
Median	-	2.00	2.00	
Third quartile	-	2.00	2.00	
Maximum	-	2.0	2.0	
EOT				
N	123	39	162	
Mean	5.17	3.49	4.77	
Std. Err.	0.245	0.350	0.212	
Std. Dev.	2.721	2.187	2.695	
95% C.I.	(4.685; 5.656)	(2.778; 4.196)	(4.347; 5.184)	
Minimum	2.0	1.0	1.0	
First quartile	3.00	2.00	3.00	
Median	5.00	3.00	4.00	
Third quartile	7.00	5.00	6.00	
Maximum	15.0	11.0	15.0	
Indirect Bilirubin (umol/L)				
Baseline				
N	123	40	163	
Mean	7.14	7.83	7.31	
Std. Err.	0.328	0.768	0.311	
Std. Dev.	3.642	4.856	3.970	
95% C.I.	(6.488; 7.788)	(6.272; 9.378)	(6.693; 7.921)	
Minimum	3.0	3.0	3.0	
First quartile	5.00	4.00	5.00	
Median	6.00	7.00	6.00	
Third quartile	9.00	9.00	9.00	
Maximum	22.0	26.0	26.0	
Week 01				
N	120	37	157	
Mean	13.68	18.65	14.85	
Std. Err.	0.994	3.164	1.072	
Std. Dev.	10.884	19.243	13.427	
95% C.I.	(11.716; 15.651)	(12.233; 25.065)	(12.737; 16.970)	
Minimum	3.0	3.0	3.0	
First quartile	7.00	9.00	8.00	
Median	11.00	11.00	11.00	
Third quartile	18.00	19.00	18.00	
Maximum	91.0	91.0	91.0	
Week 02				
N	111	36	147	
Mean	13.39	14.81	13.73	
Std. Err.	0.710	1.469	0.645	
Std. Dev.	7.476	8.815	7.818	
95% C.I.	(11.981; 14.794)	(11.823; 17.788)	(12.460; 15.009)	
Minimum	3.0	6.0	3.0	
First quartile	8.00	9.50	8.00	
Median	12.00	12.50	12.00	
Third quartile	17.00	17.00	17.00	
Maximum	47.0	47.0	47.0	
Week 04				
N	122	39	161	
Mean	11.93	12.15	11.99	
Std. Err.	0.580	1.113	0.514	
Std. Dev.	6.401	6.949	6.516	
95% C.I.	(10.787; 13.082)	(9.901; 14.407)	(10.973; 13.002)	
Minimum	2.0	5.0	2.0	
First quartile	8.00	7.00	8.00	
Median	10.00	10.00	10.00	
Third quartile	15.00	15.00	15.00	
Maximum	44.0	42.0	44.0	
Week 08				
N	123	35	158	
Mean	11.65	11.80	11.68	
Std. Err.	0.569	1.306	0.527	
Std. Dev.	6.312	7.726	6.625	
95% C.I.	(10.524; 12.777)	(9.146; 14.454)	(10.643; 12.725)	
Minimum	2.0	4.0	2.0	
First quartile	8.00	7.00	8.00	
Median	10.00	9.00	10.00	
Third quartile	15.00	15.00	15.00	
Maximum	43.0	33.0	43.0	
Week 12				
N	116	26	142	
Mean	10.93	10.58	10.87	
Std. Err.	0.551	1.169	0.497	
Std. Dev.	5.934	5.961	5.919	
95% C.I.	(9.840; 12.022)	(8.169; 12.985)	(9.884; 11.848)	
Minimum	3.0	4.0	3.0	
First quartile	6.00	7.00	6.00	
Median	10.00	9.00	10.00	
Third quartile	14.00	12.00	14.00	
Maximum	34.0	32.0	34.0	
Week 16				
N	34	29	63	
Mean	5.09	7.69	6.29	
Std. Err.	0.314	0.863	0.458	
Std. Dev.	1.832	4.645	3.639	
95% C.I.	(4.449; 5.727)	(5.923; 9.457)	(5.369; 7.202)	
Minimum	3.0	3.0	3.0	
First quartile	4.00	5.00	4.00	
Median	5.00	6.00	5.00	
Third quartile	5.00	8.00	7.00	
Maximum	12.0	23.0	23.0	
Week 20				
N	0	29	29	
Mean	-	6.72	6.72	
Std. Err.	-	0.726	0.726	
Std. Dev.	-	3.909	3.909	
95% C.I.	-	(5.237; 8.211)	(5.237; 8.211)	
Minimum	-	3.0	3.0	
First quartile	-	4.00	4.00	
Median	-	6.00	6.00	
Third quartile	-	8.00	8.00	
Maximum	-	19.0	19.0	
Week 24				
N	0	28	28	
Mean	-	7.54	7.54	
Std. Err.	-	0.977	0.977	
Std. Dev.	-	5.167	5.167	
95% C.I.	-	(5.532; 9.539)	(5.532; 9.539)	
Minimum	-	2.0	2.0	
First quartile	-	5.00	5.00	
Median	-	6.00	6.00	
Third quartile	-	8.50	8.50	
Maximum	-	22.0	22.0	
Week 28				
N	0	5	5	
Mean	-	3.80	3.80	
Std. Err.	-	0.583	0.583	
Std. Dev.	-	1.304	1.304	
95% C.I.	-	(2.181; 5.419)	(2.181; 5.419)	
Minimum	-	2.0	2.0	
First quartile	-	3.00	3.00	
Median	-	4.00	4.00	
Third quartile	-	5.00	5.00	
Maximum	-	5.0	5.0	
Week 36				
N	0	1	1	
Mean	-	4.00	4.00	
95% C.I.	-	(.; .)	(.; .)	
Minimum	-	4.0	4.0	
First quartile	-	4.00	4.00	
Median	-	4.00	4.00	
Third quartile	-	4.00	4.00	
Maximum	-	4.0	4.0	
Week 48				
N	0	1	1	
Mean	-	6.00	6.00	
95% C.I.	-	(.; .)	(.; .)	
Minimum	-	6.0	6.0	
First quartile	-	6.00	6.00	
Median	-	6.00	6.00	
Third quartile	-	6.00	6.00	
Maximum	-	6.0	6.0	
EOT				
N	123	39	162	
Mean	11.24	8.90	10.68	
Std. Err.	0.519	0.843	0.449	
Std. Dev.	5.758	5.266	5.717	
95% C.I.	(10.216; 12.272)	(7.191; 10.604)	(9.792; 11.566)	
Minimum	3.0	3.0	3.0	
First quartile	7.00	5.00	6.00	
Median	11.00	7.00	10.00	
Third quartile	14.00	11.00	14.00	
Maximum	34.0	22.0	34.0	
Bilirubin (umol/L)				
Baseline				
N	123	40	163	
Mean	9.89	11.28	10.23	
Std. Err.	0.404	0.971	0.388	
Std. Dev.	4.480	6.139	4.954	
95% C.I.	(9.087; 10.686)	(9.312; 13.238)	(9.461; 10.993)	
Minimum	4.0	4.0	4.0	
First quartile	7.00	7.00	7.00	
Median	9.00	10.00	9.00	
Third quartile	12.00	13.00	13.00	
Maximum	28.0	32.0	32.0	
Week 01				
N	121	38	159	
Mean	18.49	23.79	19.75	
Std. Err.	1.138	3.314	1.181	
Std. Dev.	12.515	20.429	14.894	
95% C.I.	(16.235; 20.740)	(17.075; 30.504)	(17.422; 22.088)	
Minimum	4.0	5.0	4.0	
First quartile	10.00	14.00	12.00	
Median	15.00	16.50	15.00	
Third quartile	24.00	27.00	24.00	
Maximum	95.0	100.0	100.0	
Week 02				
N	112	36	148	
Mean	18.63	20.50	19.09	
Std. Err.	0.881	1.681	0.782	
Std. Dev.	9.325	10.087	9.515	
95% C.I.	(16.888; 20.380)	(17.087; 23.913)	(17.542; 20.633)	
Minimum	4.0	9.0	4.0	
First quartile	11.00	14.00	12.00	
Median	18.00	18.50	18.00	
Third quartile	24.50	23.50	24.00	
Maximum	55.0	54.0	55.0	
Week 04				
N	122	39	161	
Mean	17.13	17.18	17.14	
Std. Err.	0.749	1.402	0.659	
Std. Dev.	8.268	8.757	8.361	
95% C.I.	(15.649; 18.613)	(14.341; 20.018)	(15.841; 18.444)	
Minimum	4.0	7.0	4.0	
First quartile	11.00	11.00	11.00	
Median	15.50	16.00	16.00	
Third quartile	21.00	21.00	21.00	
Maximum	51.0	54.0	54.0	
Week 08				
N	123	35	158	
Mean	17.02	16.31	16.87	
Std. Err.	0.740	1.616	0.676	
Std. Dev.	8.203	9.563	8.496	
95% C.I.	(15.560; 18.489)	(13.029; 19.599)	(15.532; 18.202)	
Minimum	4.0	6.0	4.0	
First quartile	11.00	10.00	11.00	
Median	15.00	13.00	15.00	
Third quartile	22.00	20.00	21.00	
Maximum	52.0	43.0	52.0	
Week 12				
N	123	29	152	
Mean	16.30	15.14	16.08	
Std. Err.	0.750	1.441	0.665	
Std. Dev.	8.313	7.759	8.198	
95% C.I.	(14.817; 17.785)	(12.187; 18.089)	(14.765; 17.393)	
Minimum	5.0	6.0	5.0	
First quartile	10.00	10.00	10.00	
Median	15.00	14.00	15.00	
Third quartile	21.00	17.00	21.00	
Maximum	48.0	41.0	48.0	
Week 16				
N	34	29	63	
Mean	7.03	10.59	8.67	
Std. Err.	0.438	1.067	0.585	
Std. Dev.	2.552	5.748	4.645	
95% C.I.	(6.139; 7.920)	(8.400; 12.773)	(7.497; 9.837)	
Minimum	4.0	4.0	4.0	
First quartile	6.00	7.00	6.00	
Median	6.00	9.00	7.00	
Third quartile	8.00	11.00	10.00	
Maximum	16.0	29.0	29.0	
Week 20				
N	0	29	29	
Mean	-	9.41	9.41	
Std. Err.	-	0.918	0.918	
Std. Dev.	-	4.946	4.946	
95% C.I.	-	(7.532; 11.295)	(7.532; 11.295)	
Minimum	-	4.0	4.0	
First quartile	-	6.00	6.00	
Median	-	9.00	9.00	
Third quartile	-	11.00	11.00	
Maximum	-	24.0	24.0	
Week 24				
N	0	28	28	
Mean	-	10.29	10.29	
Std. Err.	-	1.254	1.254	
Std. Dev.	-	6.638	6.638	
95% C.I.	-	(7.712; 12.860)	(7.712; 12.860)	
Minimum	-	4.0	4.0	
First quartile	-	6.00	6.00	
Median	-	8.00	8.00	
Third quartile	-	11.50	11.50	
Maximum	-	29.0	29.0	
Week 28				
N	0	5	5	
Mean	-	5.20	5.20	
Std. Err.	-	0.970	0.970	
Std. Dev.	-	2.168	2.168	
95% C.I.	-	(2.508; 7.892)	(2.508; 7.892)	
Minimum	-	2.0	2.0	
First quartile	-	4.00	4.00	
Median	-	6.00	6.00	
Third quartile	-	7.00	7.00	
Maximum	-	7.0	7.0	
Week 36				
N	0	1	1	
Mean	-	7.00	7.00	
95% C.I.	-	(.; .)	(.; .)	
Minimum	-	7.0	7.0	
First quartile	-	7.00	7.00	
Median	-	7.00	7.00	
Third quartile	-	7.00	7.00	
Maximum	-	7.0	7.0	
Week 48				
N	0	1	1	
Mean	-	8.00	8.00	
95% C.I.	-	(.; .)	(.; .)	
Minimum	-	8.0	8.0	
First quartile	-	8.00	8.00	
Median	-	8.00	8.00	
Third quartile	-	8.00	8.00	
Maximum	-	8.0	8.0	
EOT				
N	123	39	162	
Mean	16.56	12.38	15.56	
Std. Err.	0.738	1.122	0.636	
Std. Dev.	8.181	7.006	8.094	
95% C.I.	(15.101; 18.021)	(10.114; 14.656)	(14.300; 16.811)	
Minimum	5.0	4.0	4.0	
First quartile	10.00	7.00	8.00	
Median	15.00	9.00	14.50	
Third quartile	21.00	18.00	21.00	
Maximum	48.0	29.0	48.0	
	
[TSFLAB01-GT.RTF] [TMC435\HPC3014\DBR_FINAL_ANALYSIS\RE_FINAL_ANALYSIS\PROD\TSFLAB01-GT.SAS] 02NOV2015, 12:19	
