# Supplementary material for: An Open-Label Trial of 12-Week Simeprevir plus Peginterferon/Ribavirin (PR) in Treatment-Naïve Patients with Hepatitis C Virus (HCV) Genotype 1 (GT1)
Source: PLoS One. 2016 Jul 18;11(7):e0158526. doi: 10.1371/journal.pone.0158526 (PMC4948848; doi:10.1371/journal.pone.0158526)
Supplement: S1 Dataset — (ZIP) [file pone.0158526.s009.zip › Safety data/TSFLAB02-GT.rtf]

TSFLAB02-GT:	Change from Baseline in Laboratory Parameters by Analysis Timepoint (Entire Treatment Phase); Intent-to-treat (Study TMC435HPC3014)	
Simeprevir
12 Wks
150 mg
PR 12/24 	
	 Genotype 1 	
	 12 Wks
(N=123) 	 >12 Wks
(N=40) 	 All subjects
(N=163) 	
Hemoglobin (g/L)				
Week 01				
N	118	34	152	
Mean	-0.43	-0.44	-0.43	
Std. Err.	0.679	1.064	0.577	
Std. Dev.	7.376	6.205	7.111	
95% C.I.	(-1.777; 0.913)	(-2.606; 1.724)	(-1.574; 0.705)	
Minimum	-29.0	-22.0	-29.0	
First quartile	-4.00	-4.00	-4.00	
Median	0.00	-1.00	0.00	
Third quartile	5.00	3.00	4.50	
Maximum	14.0	11.0	14.0	
Week 02				
N	105	34	139	
Mean	-10.96	-13.94	-11.69	
Std. Err.	1.121	1.862	0.965	
Std. Dev.	11.491	10.860	11.374	
95% C.I.	(-13.186; -8.738)	(-17.730; -10.152)	(-13.598; -9.783)	
Minimum	-45.0	-38.0	-45.0	
First quartile	-18.00	-22.00	-19.00	
Median	-9.00	-15.00	-10.00	
Third quartile	-2.00	-7.00	-3.00	
Maximum	8.0	16.0	16.0	
Week 04				
N	121	39	160	
Mean	-20.03	-24.05	-21.01	
Std. Err.	1.204	2.596	1.113	
Std. Dev.	13.241	16.211	14.075	
95% C.I.	(-22.416; -17.650)	(-29.306; -18.796)	(-23.210; -18.815)	
Minimum	-47.0	-63.0	-63.0	
First quartile	-30.00	-35.00	-31.00	
Median	-18.00	-22.00	-18.00	
Third quartile	-11.00	-12.00	-11.00	
Maximum	6.0	7.0	7.0	
Week 08				
N	122	34	156	
Mean	-23.90	-26.91	-24.56	
Std. Err.	1.073	2.802	1.038	
Std. Dev.	11.849	16.340	12.962	
95% C.I.	(-26.025; -21.778)	(-32.613; -21.211)	(-26.608; -22.508)	
Minimum	-55.0	-64.0	-64.0	
First quartile	-30.00	-37.00	-31.50	
Median	-22.50	-28.50	-23.00	
Third quartile	-16.00	-15.00	-16.00	
Maximum	2.0	13.0	13.0	
Week 12				
N	115	28	143	
Mean	-25.67	-27.46	-26.02	
Std. Err.	1.126	2.861	1.062	
Std. Dev.	12.076	15.140	12.695	
95% C.I.	(-27.900; -23.439)	(-33.335; -21.594)	(-28.120; -23.922)	
Minimum	-57.0	-58.0	-58.0	
First quartile	-34.00	-38.00	-35.00	
Median	-24.00	-28.00	-25.00	
Third quartile	-18.00	-15.50	-17.00	
Maximum	6.0	5.0	6.0	
Week 16				
N	34	28	62	
Mean	-13.65	-29.96	-21.02	
Std. Err.	1.799	2.831	1.909	
Std. Dev.	10.491	14.983	15.031	
95% C.I.	(-17.307; -9.987)	(-35.774; -24.155)	(-24.833; -17.199)	
Minimum	-42.0	-72.0	-72.0	
First quartile	-19.00	-39.50	-28.00	
Median	-11.00	-28.00	-17.50	
Third quartile	-8.00	-21.00	-10.00	
Maximum	4.0	-4.0	4.0	
Week 20				
N	0	28	28	
Mean	-	-30.04	-30.04	
Std. Err.	-	2.957	2.957	
Std. Dev.	-	15.648	15.648	
95% C.I.	-	(-36.103; -23.968)	(-36.103; -23.968)	
Minimum	-	-75.0	-75.0	
First quartile	-	-39.00	-39.00	
Median	-	-27.50	-27.50	
Third quartile	-	-18.50	-18.50	
Maximum	-	2.0	2.0	
Week 24				
N	0	27	27	
Mean	-	-30.04	-30.04	
Std. Err.	-	2.928	2.928	
Std. Dev.	-	15.215	15.215	
95% C.I.	-	(-36.056; -24.018)	(-36.056; -24.018)	
Minimum	-	-74.0	-74.0	
First quartile	-	-35.00	-35.00	
Median	-	-29.00	-29.00	
Third quartile	-	-18.00	-18.00	
Maximum	-	-3.0	-3.0	
Week 28				
N	0	5	5	
Mean	-	-22.40	-22.40	
Std. Err.	-	9.320	9.320	
Std. Dev.	-	20.840	20.840	
95% C.I.	-	(-48.276; 3.476)	(-48.276; 3.476)	
Minimum	-	-59.0	-59.0	
First quartile	-	-19.00	-19.00	
Median	-	-14.00	-14.00	
Third quartile	-	-12.00	-12.00	
Maximum	-	-8.0	-8.0	
Week 36				
N	0	1	1	
Mean	-	-59.00	-59.00	
95% C.I.	-	(.; .)	(.; .)	
Minimum	-	-59.0	-59.0	
First quartile	-	-59.00	-59.00	
Median	-	-59.00	-59.00	
Third quartile	-	-59.00	-59.00	
Maximum	-	-59.0	-59.0	
Week 48				
N	0	1	1	
Mean	-	-62.00	-62.00	
95% C.I.	-	(.; .)	(.; .)	
Minimum	-	-62.0	-62.0	
First quartile	-	-62.00	-62.00	
Median	-	-62.00	-62.00	
Third quartile	-	-62.00	-62.00	
Maximum	-	-62.0	-62.0	
EOT				
N	123	39	162	
Mean	-25.82	-29.36	-26.67	
Std. Err.	1.053	2.372	0.986	
Std. Dev.	11.682	14.814	12.550	
95% C.I.	(-27.906; -23.736)	(-34.161; -24.557)	(-28.620; -24.726)	
Minimum	-57.0	-74.0	-74.0	
First quartile	-33.00	-35.00	-34.00	
Median	-25.00	-27.00	-25.00	
Third quartile	-18.00	-18.00	-18.00	
Maximum	0.0	-3.0	0.0	
Neutrophils and Precursors (x10E9/L)				
Week 01				
N	118	34	152	
Mean	-1.72	-1.82	-1.74	
Std. Err.	0.127	0.301	0.119	
Std. Dev.	1.375	1.755	1.463	
95% C.I.	(-1.969; -1.467)	(-2.431; -1.206)	(-1.975; -1.506)	
Minimum	-8.3	-8.6	-8.6	
First quartile	-2.31	-2.38	-2.32	
Median	-1.63	-1.50	-1.62	
Third quartile	-0.87	-0.74	-0.87	
Maximum	3.7	0.4	3.7	
Week 02				
N	104	34	138	
Mean	-1.94	-1.94	-1.94	
Std. Err.	0.151	0.378	0.146	
Std. Dev.	1.543	2.205	1.721	
95% C.I.	(-2.237; -1.637)	(-2.712; -1.173)	(-2.228; -1.649)	
Minimum	-9.3	-9.9	-9.9	
First quartile	-2.58	-2.46	-2.52	
Median	-1.82	-1.72	-1.80	
Third quartile	-0.91	-1.35	-0.92	
Maximum	1.5	3.3	3.3	
Week 04				
N	121	39	160	
Mean	-2.04	-2.38	-2.13	
Std. Err.	0.122	0.317	0.120	
Std. Dev.	1.338	1.981	1.520	
95% C.I.	(-2.285; -1.803)	(-3.027; -1.743)	(-2.365; -1.890)	
Minimum	-7.2	-10.1	-10.1	
First quartile	-2.72	-2.92	-2.74	
Median	-1.90	-1.90	-1.90	
Third quartile	-1.16	-1.26	-1.19	
Maximum	0.6	0.2	0.6	
Week 08				
N	122	34	156	
Mean	-2.16	-2.09	-2.15	
Std. Err.	0.126	0.317	0.120	
Std. Dev.	1.389	1.847	1.495	
95% C.I.	(-2.411; -1.913)	(-2.732; -1.443)	(-2.382; -1.910)	
Minimum	-8.9	-8.8	-8.9	
First quartile	-2.84	-2.97	-2.86	
Median	-1.99	-1.92	-1.98	
Third quartile	-1.37	-0.83	-1.13	
Maximum	0.6	0.5	0.6	
Week 12				
N	115	28	143	
Mean	-2.17	-2.54	-2.24	
Std. Err.	0.152	0.349	0.140	
Std. Dev.	1.631	1.848	1.676	
95% C.I.	(-2.468; -1.865)	(-3.261; -1.828)	(-2.518; -1.964)	
Minimum	-9.8	-10.1	-10.1	
First quartile	-2.91	-2.94	-2.91	
Median	-2.04	-2.06	-2.04	
Third quartile	-1.01	-1.45	-1.14	
Maximum	1.0	-0.8	1.0	
Week 16				
N	34	28	62	
Mean	-0.82	-2.45	-1.56	
Std. Err.	0.363	0.278	0.255	
Std. Dev.	2.115	1.472	2.011	
95% C.I.	(-1.560; -0.084)	(-3.019; -1.878)	(-2.067; -1.046)	
Minimum	-8.1	-6.6	-8.1	
First quartile	-1.51	-3.03	-2.56	
Median	-0.77	-2.12	-1.39	
Third quartile	0.58	-1.45	-0.61	
Maximum	3.6	0.0	3.6	
Week 20				
N	0	28	28	
Mean	-	-2.40	-2.40	
Std. Err.	-	0.403	0.403	
Std. Dev.	-	2.133	2.133	
95% C.I.	-	(-3.225; -1.571)	(-3.225; -1.571)	
Minimum	-	-9.9	-9.9	
First quartile	-	-2.97	-2.97	
Median	-	-2.20	-2.20	
Third quartile	-	-1.13	-1.13	
Maximum	-	1.4	1.4	
Week 24				
N	0	27	27	
Mean	-	-2.58	-2.58	
Std. Err.	-	0.308	0.308	
Std. Dev.	-	1.599	1.599	
95% C.I.	-	(-3.209; -1.944)	(-3.209; -1.944)	
Minimum	-	-7.4	-7.4	
First quartile	-	-3.21	-3.21	
Median	-	-2.25	-2.25	
Third quartile	-	-1.45	-1.45	
Maximum	-	0.0	0.0	
Week 28				
N	0	5	5	
Mean	-	-2.30	-2.30	
Std. Err.	-	1.047	1.047	
Std. Dev.	-	2.342	2.342	
95% C.I.	-	(-5.208; 0.608)	(-5.208; 0.608)	
Minimum	-	-5.9	-5.9	
First quartile	-	-2.60	-2.60	
Median	-	-2.39	-2.39	
Third quartile	-	-1.03	-1.03	
Maximum	-	0.4	0.4	
Week 36				
N	0	1	1	
Mean	-	-1.45	-1.45	
95% C.I.	-	(.; .)	(.; .)	
Minimum	-	-1.5	-1.5	
First quartile	-	-1.45	-1.45	
Median	-	-1.45	-1.45	
Third quartile	-	-1.45	-1.45	
Maximum	-	-1.5	-1.5	
Week 48				
N	0	1	1	
Mean	-	-1.31	-1.31	
95% C.I.	-	(.; .)	(.; .)	
Minimum	-	-1.3	-1.3	
First quartile	-	-1.31	-1.31	
Median	-	-1.31	-1.31	
Third quartile	-	-1.31	-1.31	
Maximum	-	-1.3	-1.3	
EOT				
N	123	39	162	
Mean	-2.15	-2.41	-2.22	
Std. Err.	0.148	0.269	0.130	
Std. Dev.	1.641	1.683	1.649	
95% C.I.	(-2.448; -1.862)	(-2.955; -1.864)	(-2.472; -1.960)	
Minimum	-9.8	-7.4	-9.8	
First quartile	-2.91	-2.99	-2.91	
Median	-2.05	-2.09	-2.06	
Third quartile	-1.07	-1.28	-1.13	
Maximum	1.0	0.0	1.0	
Platelets (x10E9/L)				
Week 01				
N	117	34	151	
Mean	-57.59	-41.97	-54.07	
Std. Err.	3.829	6.766	3.367	
Std. Dev.	41.419	39.453	41.376	
95% C.I.	(-65.174; -50.006)	(-55.736; -28.205)	(-60.726; -47.420)	
Minimum	-193.0	-124.0	-193.0	
First quartile	-85.00	-68.00	-79.00	
Median	-51.00	-39.00	-49.00	
Third quartile	-31.00	-12.00	-27.00	
Maximum	34.0	33.0	34.0	
Week 02				
N	105	34	139	
Mean	-58.18	-50.29	-56.25	
Std. Err.	5.628	8.969	4.777	
Std. Dev.	57.675	52.295	56.324	
95% C.I.	(-69.342; -47.019)	(-68.541; -32.048)	(-65.698; -46.806)	
Minimum	-188.0	-168.0	-188.0	
First quartile	-93.00	-68.00	-89.00	
Median	-57.00	-40.00	-54.00	
Third quartile	-29.00	-20.00	-27.00	
Maximum	236.0	34.0	236.0	
Week 04				
N	119	39	158	
Mean	-55.70	-33.79	-50.29	
Std. Err.	4.850	8.935	4.319	
Std. Dev.	52.909	55.801	54.290	
95% C.I.	(-65.302; -46.093)	(-51.884; -15.706)	(-58.822; -41.760)	
Minimum	-205.0	-157.0	-205.0	
First quartile	-87.00	-61.00	-83.00	
Median	-61.00	-35.00	-54.50	
Third quartile	-23.00	16.00	-19.00	
Maximum	104.0	70.0	104.0	
Week 08				
N	119	34	153	
Mean	-76.50	-54.65	-71.64	
Std. Err.	5.007	10.669	4.601	
Std. Dev.	54.615	62.208	56.911	
95% C.I.	(-86.410; -66.581)	(-76.353; -32.942)	(-80.731; -62.550)	
Minimum	-217.0	-190.0	-217.0	
First quartile	-103.00	-95.00	-102.00	
Median	-77.00	-48.00	-76.00	
Third quartile	-50.00	-3.00	-38.00	
Maximum	120.0	49.0	120.0	
Week 12				
N	113	28	141	
Mean	-77.62	-73.14	-76.73	
Std. Err.	4.934	12.203	4.619	
Std. Dev.	52.447	64.574	54.845	
95% C.I.	(-87.395; -67.844)	(-98.182; -48.104)	(-85.862; -67.599)	
Minimum	-250.0	-210.0	-250.0	
First quartile	-111.00	-118.00	-112.00	
Median	-77.00	-60.00	-75.00	
Third quartile	-46.00	-34.00	-44.00	
Maximum	62.0	58.0	62.0	
Week 16				
N	34	28	62	
Mean	-20.91	-66.32	-41.42	
Std. Err.	8.171	11.465	7.379	
Std. Dev.	47.643	60.667	58.104	
95% C.I.	(-37.535; -4.288)	(-89.846; -42.797)	(-56.175; -26.664)	
Minimum	-113.0	-200.0	-200.0	
First quartile	-55.00	-96.50	-69.00	
Median	-27.00	-64.00	-36.50	
Third quartile	8.00	-26.00	-9.00	
Maximum	116.0	79.0	116.0	
Week 20				
N	0	28	28	
Mean	-	-73.04	-73.04	
Std. Err.	-	12.798	12.798	
Std. Dev.	-	67.722	67.722	
95% C.I.	-	(-99.296; -46.776)	(-99.296; -46.776)	
Minimum	-	-239.0	-239.0	
First quartile	-	-114.50	-114.50	
Median	-	-70.50	-70.50	
Third quartile	-	-32.00	-32.00	
Maximum	-	58.0	58.0	
Week 24				
N	0	27	27	
Mean	-	-82.19	-82.19	
Std. Err.	-	13.464	13.464	
Std. Dev.	-	69.963	69.963	
95% C.I.	-	(-109.861; -54.509)	(-109.861; -54.509)	
Minimum	-	-240.0	-240.0	
First quartile	-	-129.00	-129.00	
Median	-	-70.00	-70.00	
Third quartile	-	-38.00	-38.00	
Maximum	-	69.0	69.0	
Week 28				
N	0	5	5	
Mean	-	-81.00	-81.00	
Std. Err.	-	21.615	21.615	
Std. Dev.	-	48.332	48.332	
95% C.I.	-	(-141.012; -20.988)	(-141.012; -20.988)	
Minimum	-	-133.0	-133.0	
First quartile	-	-99.00	-99.00	
Median	-	-95.00	-95.00	
Third quartile	-	-75.00	-75.00	
Maximum	-	-3.0	-3.0	
Week 36				
N	0	1	1	
Mean	-	-84.00	-84.00	
95% C.I.	-	(.; .)	(.; .)	
Minimum	-	-84.0	-84.0	
First quartile	-	-84.00	-84.00	
Median	-	-84.00	-84.00	
Third quartile	-	-84.00	-84.00	
Maximum	-	-84.0	-84.0	
Week 48				
N	0	1	1	
Mean	-	-78.00	-78.00	
95% C.I.	-	(.; .)	(.; .)	
Minimum	-	-78.0	-78.0	
First quartile	-	-78.00	-78.00	
Median	-	-78.00	-78.00	
Third quartile	-	-78.00	-78.00	
Maximum	-	-78.0	-78.0	
EOT				
N	122	39	161	
Mean	-77.50	-72.05	-76.18	
Std. Err.	4.727	11.114	4.466	
Std. Dev.	52.212	69.405	56.667	
95% C.I.	(-86.858; -68.142)	(-94.550; -49.553)	(-85.000; -67.360)	
Minimum	-250.0	-240.0	-250.0	
First quartile	-111.00	-111.00	-111.00	
Median	-75.50	-70.00	-75.00	
Third quartile	-45.00	-29.00	-39.00	
Maximum	62.0	69.0	69.0	
Direct Bilirubin (umol/L)				
Week 01				
N	120	37	157	
Mean	2.15	2.05	2.13	
Std. Err.	0.225	0.315	0.187	
Std. Dev.	2.469	1.914	2.344	
95% C.I.	(1.704; 2.596)	(1.416; 2.692)	(1.758; 2.497)	
Minimum	-2.0	-1.0	-2.0	
First quartile	0.50	1.00	1.00	
Median	2.00	2.00	2.00	
Third quartile	3.00	3.00	3.00	
Maximum	12.0	7.0	12.0	
Week 02				
N	111	36	147	
Mean	2.36	2.28	2.34	
Std. Err.	0.225	0.263	0.181	
Std. Dev.	2.369	1.579	2.197	
95% C.I.	(1.915; 2.806)	(1.744; 2.812)	(1.982; 2.698)	
Minimum	-2.0	-1.0	-2.0	
First quartile	1.00	1.00	1.00	
Median	2.00	2.00	2.00	
Third quartile	4.00	3.00	4.00	
Maximum	15.0	6.0	15.0	
Week 04				
N	122	39	161	
Mean	2.43	1.54	2.22	
Std. Err.	0.208	0.257	0.172	
Std. Dev.	2.300	1.603	2.181	
95% C.I.	(2.022; 2.847)	(1.019; 2.058)	(1.878; 2.557)	
Minimum	-1.0	-2.0	-2.0	
First quartile	1.00	1.00	1.00	
Median	2.00	1.00	2.00	
Third quartile	3.00	2.00	3.00	
Maximum	15.0	6.0	15.0	
Week 08				
N	123	35	158	
Mean	2.60	1.20	2.29	
Std. Err.	0.237	0.259	0.198	
Std. Dev.	2.623	1.530	2.489	
95% C.I.	(2.133; 3.070)	(0.674; 1.726)	(1.900; 2.682)	
Minimum	-2.0	-2.0	-2.0	
First quartile	1.00	0.00	1.00	
Median	2.00	1.00	2.00	
Third quartile	4.00	2.00	3.00	
Maximum	14.0	4.0	14.0	
Week 12				
N	116	26	142	
Mean	2.16	1.46	2.03	
Std. Err.	0.232	0.295	0.198	
Std. Dev.	2.497	1.503	2.358	
95% C.I.	(1.696; 2.614)	(0.855; 2.069)	(1.637; 2.419)	
Minimum	-2.0	-1.0	-2.0	
First quartile	0.00	0.00	0.00	
Median	2.00	1.00	1.00	
Third quartile	4.00	2.00	4.00	
Maximum	11.0	5.0	11.0	
Week 16				
N	34	29	63	
Mean	-0.88	-0.28	-0.60	
Std. Err.	0.238	0.204	0.162	
Std. Dev.	1.387	1.099	1.289	
95% C.I.	(-1.366; -0.398)	(-0.694; 0.142)	(-0.928; -0.278)	
Minimum	-5.0	-3.0	-5.0	
First quartile	-2.00	-1.00	-1.00	
Median	-1.00	0.00	-1.00	
Third quartile	0.00	0.00	0.00	
Maximum	2.0	2.0	2.0	
Week 20				
N	0	29	29	
Mean	-	-0.48	-0.48	
Std. Err.	-	0.256	0.256	
Std. Dev.	-	1.379	1.379	
95% C.I.	-	(-1.007; 0.042)	(-1.007; 0.042)	
Minimum	-	-3.0	-3.0	
First quartile	-	-1.00	-1.00	
Median	-	-1.00	-1.00	
Third quartile	-	0.00	0.00	
Maximum	-	3.0	3.0	
Week 24				
N	0	28	28	
Mean	-	-0.39	-0.39	
Std. Err.	-	0.214	0.214	
Std. Dev.	-	1.133	1.133	
95% C.I.	-	(-0.832; 0.047)	(-0.832; 0.047)	
Minimum	-	-3.0	-3.0	
First quartile	-	-1.00	-1.00	
Median	-	-1.00	-1.00	
Third quartile	-	0.00	0.00	
Maximum	-	2.0	2.0	
Week 28				
N	0	5	5	
Mean	-	-1.00	-1.00	
Std. Err.	-	0.316	0.316	
Std. Dev.	-	0.707	0.707	
95% C.I.	-	(-1.878; -0.122)	(-1.878; -0.122)	
Minimum	-	-2.0	-2.0	
First quartile	-	-1.00	-1.00	
Median	-	-1.00	-1.00	
Third quartile	-	-1.00	-1.00	
Maximum	-	0.0	0.0	
Week 36				
N	0	1	1	
Mean	-	0.00	0.00	
95% C.I.	-	(.; .)	(.; .)	
Minimum	-	0.0	0.0	
First quartile	-	0.00	0.00	
Median	-	0.00	0.00	
Third quartile	-	0.00	0.00	
Maximum	-	0.0	0.0	
Week 48				
N	0	1	1	
Mean	-	-1.00	-1.00	
95% C.I.	-	(.; .)	(.; .)	
Minimum	-	-1.0	-1.0	
First quartile	-	-1.00	-1.00	
Median	-	-1.00	-1.00	
Third quartile	-	-1.00	-1.00	
Maximum	-	-1.0	-1.0	
EOT				
N	123	39	162	
Mean	2.40	0.00	1.82	
Std. Err.	0.235	0.238	0.204	
Std. Dev.	2.604	1.487	2.592	
95% C.I.	(1.934; 2.863)	(-0.482; 0.482)	(1.419; 2.223)	
Minimum	-2.0	-3.0	-3.0	
First quartile	0.00	-1.00	0.00	
Median	2.00	0.00	1.00	
Third quartile	4.00	1.00	3.00	
Maximum	12.0	4.0	12.0	
Indirect Bilirubin (umol/L)				
Week 01				
N	120	37	157	
Mean	6.62	10.97	7.64	
Std. Err.	0.891	2.666	0.934	
Std. Dev.	9.764	16.215	11.698	
95% C.I.	(4.852; 8.382)	(5.567; 16.379)	(5.799; 9.487)	
Minimum	-6.0	-4.0	-6.0	
First quartile	1.00	3.00	1.00	
Median	4.00	5.00	5.00	
Third quartile	11.00	11.00	11.00	
Maximum	76.0	80.0	80.0	
Week 02				
N	111	36	147	
Mean	6.18	7.08	6.40	
Std. Err.	0.612	0.997	0.522	
Std. Dev.	6.451	5.983	6.332	
95% C.I.	(4.967; 7.394)	(5.059; 9.108)	(5.369; 7.433)	
Minimum	-5.0	-2.0	-5.0	
First quartile	2.00	4.00	2.00	
Median	5.00	5.50	5.00	
Third quartile	9.00	9.00	9.00	
Maximum	32.0	28.0	32.0	
Week 04				
N	122	39	161	
Mean	4.81	4.23	4.67	
Std. Err.	0.471	0.686	0.393	
Std. Dev.	5.207	4.283	4.992	
95% C.I.	(3.878; 5.745)	(2.843; 5.619)	(3.894; 5.448)	
Minimum	-6.0	-3.0	-6.0	
First quartile	1.00	1.00	1.00	
Median	4.00	4.00	4.00	
Third quartile	8.00	7.00	7.00	
Maximum	30.0	16.0	30.0	
Week 08				
N	123	35	158	
Mean	4.51	3.86	4.37	
Std. Err.	0.442	0.877	0.394	
Std. Dev.	4.900	5.186	4.955	
95% C.I.	(3.638; 5.387)	(2.076; 5.638)	(3.588; 5.146)	
Minimum	-4.0	-8.0	-8.0	
First quartile	1.00	1.00	1.00	
Median	4.00	3.00	4.00	
Third quartile	7.00	6.00	7.00	
Maximum	28.0	21.0	28.0	
Week 12				
N	116	26	142	
Mean	3.68	3.27	3.61	
Std. Err.	0.461	0.522	0.388	
Std. Dev.	4.967	2.662	4.626	
95% C.I.	(2.768; 4.595)	(2.194; 4.344)	(2.838; 4.373)	
Minimum	-8.0	-1.0	-8.0	
First quartile	0.00	1.00	0.00	
Median	3.00	3.00	3.00	
Third quartile	6.50	5.00	6.00	
Maximum	21.0	9.0	21.0	
Week 16				
N	34	29	63	
Mean	-2.09	0.07	-1.10	
Std. Err.	0.714	0.572	0.483	
Std. Dev.	4.166	3.081	3.834	
95% C.I.	(-3.542; -0.635)	(-1.103; 1.241)	(-2.061; -0.130)	
Minimum	-18.0	-5.0	-18.0	
First quartile	-3.00	-3.00	-3.00	
Median	-2.00	0.00	-1.00	
Third quartile	0.00	2.00	1.00	
Maximum	5.0	8.0	8.0	
Week 20				
N	0	29	29	
Mean	-	-0.90	-0.90	
Std. Err.	-	0.630	0.630	
Std. Dev.	-	3.395	3.395	
95% C.I.	-	(-2.188; 0.395)	(-2.188; 0.395)	
Minimum	-	-10.0	-10.0	
First quartile	-	-3.00	-3.00	
Median	-	0.00	0.00	
Third quartile	-	1.00	1.00	
Maximum	-	7.0	7.0	
Week 24				
N	0	28	28	
Mean	-	-0.21	-0.21	
Std. Err.	-	0.706	0.706	
Std. Dev.	-	3.735	3.735	
95% C.I.	-	(-1.663; 1.234)	(-1.663; 1.234)	
Minimum	-	-9.0	-9.0	
First quartile	-	-2.50	-2.50	
Median	-	0.00	0.00	
Third quartile	-	2.00	2.00	
Maximum	-	7.0	7.0	
Week 28				
N	0	5	5	
Mean	-	-2.00	-2.00	
Std. Err.	-	0.707	0.707	
Std. Dev.	-	1.581	1.581	
95% C.I.	-	(-3.963; -0.037)	(-3.963; -0.037)	
Minimum	-	-4.0	-4.0	
First quartile	-	-3.00	-3.00	
Median	-	-2.00	-2.00	
Third quartile	-	-1.00	-1.00	
Maximum	-	0.0	0.0	
Week 36				
N	0	1	1	
Mean	-	-4.00	-4.00	
95% C.I.	-	(.; .)	(.; .)	
Minimum	-	-4.0	-4.0	
First quartile	-	-4.00	-4.00	
Median	-	-4.00	-4.00	
Third quartile	-	-4.00	-4.00	
Maximum	-	-4.0	-4.0	
Week 48				
N	0	1	1	
Mean	-	-2.00	-2.00	
95% C.I.	-	(.; .)	(.; .)	
Minimum	-	-2.0	-2.0	
First quartile	-	-2.00	-2.00	
Median	-	-2.00	-2.00	
Third quartile	-	-2.00	-2.00	
Maximum	-	-2.0	-2.0	
EOT				
N	123	39	162	
Mean	4.11	0.97	3.35	
Std. Err.	0.440	0.702	0.388	
Std. Dev.	4.880	4.386	4.939	
95% C.I.	(3.235; 4.977)	(-0.447; 2.396)	(2.586; 4.118)	
Minimum	-8.0	-9.0	-9.0	
First quartile	1.00	-2.00	0.00	
Median	3.00	0.00	3.00	
Third quartile	7.00	3.00	6.00	
Maximum	21.0	13.0	21.0	
Bilirubin (umol/L)				
Week 01				
N	121	38	159	
Mean	8.72	12.63	9.65	
Std. Err.	1.025	2.769	1.027	
Std. Dev.	11.275	17.071	12.946	
95% C.I.	(6.690; 10.748)	(7.020; 18.243)	(7.626; 11.682)	
Minimum	-6.0	-5.0	-6.0	
First quartile	1.00	4.00	2.00	
Median	6.00	7.00	7.00	
Third quartile	14.00	15.00	14.00	
Maximum	76.0	85.0	85.0	
Week 02				
N	112	36	148	
Mean	8.61	9.36	8.79	
Std. Err.	0.780	1.147	0.652	
Std. Dev.	8.253	6.883	7.926	
95% C.I.	(7.062; 10.152)	(7.032; 11.690)	(7.503; 10.078)	
Minimum	-5.0	-3.0	-5.0	
First quartile	2.50	5.00	3.50	
Median	7.00	8.00	8.00	
Third quartile	13.00	12.50	13.00	
Maximum	37.0	28.0	37.0	
Week 04				
N	122	39	161	
Mean	7.27	5.77	6.91	
Std. Err.	0.633	0.868	0.525	
Std. Dev.	6.991	5.422	6.660	
95% C.I.	(6.017; 8.524)	(4.012; 7.527)	(5.870; 7.943)	
Minimum	-6.0	-3.0	-6.0	
First quartile	3.00	2.00	2.00	
Median	6.00	5.00	6.00	
Third quartile	11.00	9.00	10.00	
Maximum	34.0	22.0	34.0	
Week 08				
N	123	35	158	
Mean	7.14	5.06	6.68	
Std. Err.	0.615	1.045	0.535	
Std. Dev.	6.826	6.183	6.726	
95% C.I.	(5.920; 8.357)	(2.933; 7.181)	(5.620; 7.734)	
Minimum	-5.0	-10.0	-10.0	
First quartile	2.00	1.00	2.00	
Median	6.00	4.00	5.50	
Third quartile	12.00	8.00	10.00	
Maximum	35.0	25.0	35.0	
Week 12				
N	123	29	152	
Mean	6.41	4.34	6.02	
Std. Err.	0.675	0.708	0.565	
Std. Dev.	7.483	3.810	6.972	
95% C.I.	(5.079; 7.750)	(2.895; 5.794)	(4.902; 7.137)	
Minimum	-10.0	-5.0	-10.0	
First quartile	1.00	2.00	1.00	
Median	6.00	4.00	5.00	
Third quartile	11.00	6.00	10.00	
Maximum	33.0	12.0	33.0	
Week 16				
N	34	29	63	
Mean	-2.97	-0.21	-1.70	
Std. Err.	0.918	0.711	0.615	
Std. Dev.	5.351	3.830	4.878	
95% C.I.	(-4.838; -1.103)	(-1.664; 1.250)	(-2.927; -0.470)	
Minimum	-23.0	-7.0	-23.0	
First quartile	-5.00	-3.00	-4.00	
Median	-3.00	-1.00	-1.00	
Third quartile	1.00	2.00	2.00	
Maximum	7.0	9.0	9.0	
Week 20				
N	0	29	29	
Mean	-	-1.38	-1.38	
Std. Err.	-	0.801	0.801	
Std. Dev.	-	4.313	4.313	
95% C.I.	-	(-3.020; 0.261)	(-3.020; 0.261)	
Minimum	-	-12.0	-12.0	
First quartile	-	-3.00	-3.00	
Median	-	-1.00	-1.00	
Third quartile	-	1.00	1.00	
Maximum	-	8.0	8.0	
Week 24				
N	0	28	28	
Mean	-	-0.61	-0.61	
Std. Err.	-	0.812	0.812	
Std. Dev.	-	4.298	4.298	
95% C.I.	-	(-2.274; 1.059)	(-2.274; 1.059)	
Minimum	-	-10.0	-10.0	
First quartile	-	-3.00	-3.00	
Median	-	-1.00	-1.00	
Third quartile	-	2.00	2.00	
Maximum	-	9.0	9.0	
Week 28				
N	0	5	5	
Mean	-	-3.20	-3.20	
Std. Err.	-	0.860	0.860	
Std. Dev.	-	1.924	1.924	
95% C.I.	-	(-5.588; -0.812)	(-5.588; -0.812)	
Minimum	-	-6.0	-6.0	
First quartile	-	-4.00	-4.00	
Median	-	-3.00	-3.00	
Third quartile	-	-2.00	-2.00	
Maximum	-	-1.0	-1.0	
Week 36				
N	0	1	1	
Mean	-	-4.00	-4.00	
95% C.I.	-	(.; .)	(.; .)	
Minimum	-	-4.0	-4.0	
First quartile	-	-4.00	-4.00	
Median	-	-4.00	-4.00	
Third quartile	-	-4.00	-4.00	
Maximum	-	-4.0	-4.0	
Week 48				
N	0	1	1	
Mean	-	-3.00	-3.00	
95% C.I.	-	(.; .)	(.; .)	
Minimum	-	-3.0	-3.0	
First quartile	-	-3.00	-3.00	
Median	-	-3.00	-3.00	
Third quartile	-	-3.00	-3.00	
Maximum	-	-3.0	-3.0	
EOT				
N	123	39	162	
Mean	6.67	0.97	5.30	
Std. Err.	0.664	0.841	0.575	
Std. Dev.	7.360	5.254	7.317	
95% C.I.	(5.361; 7.989)	(-0.729; 2.678)	(4.167; 6.438)	
Minimum	-10.0	-10.0	-10.0	
First quartile	1.00	-3.00	0.00	
Median	6.00	0.00	4.00	
Third quartile	11.00	4.00	10.00	
Maximum	33.0	16.0	33.0	
	
[TSFLAB02-GT.RTF] [TMC435\HPC3014\DBR_FINAL_ANALYSIS\RE_FINAL_ANALYSIS\PROD\TSFLAB02-GT.SAS] 02NOV2015, 12:10	
