# Supplementary material for: An Open-Label Trial of 12-Week Simeprevir plus Peginterferon/Ribavirin (PR) in Treatment-Naïve Patients with Hepatitis C Virus (HCV) Genotype 1 (GT1)
Source: PLoS One. 2016 Jul 18;11(7):e0158526. doi: 10.1371/journal.pone.0158526 (PMC4948848; doi:10.1371/journal.pone.0158526)
Supplement: S1 Dataset — (ZIP) [file pone.0158526.s009.zip › LSIDEM02.rtf]

LSIDEM02:	Listing of Baseline Disease Characteristics; Intent-to-treat (Study TMC435HPC3014)
Treatment Group = Simeprevir 12Wks 150 mg PR12/24	
	Baseline HCV RNA		Baseline ALT	HCV Geno
/Subtype	IL28b Genotype		
Subject ID	IU/mL
/log10 IU/mL
/Category IU/mL	Metavir
Scorea	U/L
/Toxicity Grade	Coalesceb
/Trugene
/LIPA
/As Stratified	As Analysed
/As Stratified	Duration
of HCV
Infection (yrs)	Time Since
Diagnosis (yrs)	Mode
of HCV
Infection	
HCV Genotype: Genotype 1 - Treatment Duration: 12 Weeks Treatment	
30140001	6300000
/  6.7993
/>800000	F2	82
/Grade 1	1a
/
/1a
/1a	CT
/CT	20.8	11.9	Other	
30140002	122000
/  5.0864
/<400000	F0-F1	98
/Grade 2	1a
/
/1a
/1a	CT
/CT	15.8	15.8	Intravenously injectable drug use	
30140003	4520000
/  6.6551
/>800000	F0-F1	19
/Grade 0	1a
/
/1a
/1a	TT
/TT	23.8	0.6	Intravenously injectable drug use	
30140004	1450000
/  6.1614
/>800000	F2	178
/Grade 2	1a
/
/1a
/1a	CT
/CT	16.8	1.5	Intravenously injectable drug use	
30140006	8570000
/  6.9330
/>800000	F0-F1	51
/Grade 0	1b
/
/1b
/1b	TT
/TT		0.7	Other	
30140007	1670000
/  6.2227
/>800000	F0-F1	50
/Grade 1	1b
/
/1b
/1b	TT
/TT	23.8	0.7	Blood transfusion	
30140008	4480000
/  6.6513
/>800000	F0-F1	46
/Grade 1	1a
/
/1a
/1a	CC
/CC	43.8	10.8	Other	
30140009	2400000
/  6.3802
/>800000	F2	31
/Grade 0	1b
/
/1b
/1b	CT
/CT		6.9	Blood transfusion	
30140010	852000
/  5.9304
/>800000	F0-F1	55
/Grade 1	1a
/
/1a
/1a	CT
/CT		0.4	Other	
30140011	6560000
/  6.8169
/>800000	F0-F1	84
/Grade 1	1b
/
/1b
/1b	TT
/TT	27.8	0.5	Blood transfusion	
30140012	1840000
/  6.2648
/>800000	F0-F1	39
/Grade 0	1a
/1a
/
/1a	CT
/CT	3.8	3.8	Other	
30140013	1230000
/  6.0899
/>800000	F0-F1	82
/Grade 1	1a
/
/1a
/1a	CT
/CT		1.4	Multiple	
30140016	4500
/  3.6532
/<400000	F0-F1	51
/Grade 0	1a
/
/1a
/1a	CC
/CC		1.3	Other	
30140017	2830000
/  6.4518
/>800000	F2	81
/Grade 1	1a
/
/1a
/1a	TT
/TT		17.9	Intravenously injectable drug use	
30140019	4500000
/  6.6532
/>800000	F0-F1	64
/Grade 1	1a
/
/1a
/1a	CC
/CC	0.8	0.6	Other	
30140020	3950000
/  6.5966
/>800000	F0-F1	46
/Grade 0	1b
/
/1b
/1b	CT
/CT	28.9	28.9	Multiple	
30140021	5980000
/  6.7767
/>800000	F0-F1	73
/Grade 1	1b
/
/1b
/1b	CT
/CT		17.9	Intravenously injectable drug use	
30140024	491000
/  5.6911
/≥400000 - ≤800000	F0-F1	75
/Grade 1	1b
/
/1b
/1b	TT
/TT		22.8	Other	
30140025	1620000
/  6.2095
/>800000	F0-F1	26
/Grade 0	1b
/
/1b
/1b	TT
/TT		0.8	Other	
30140027	565000
/  5.7520
/≥400000 - ≤800000	F0-F1	65
/Grade 1	1b
/
/1b
/1b	TT
/TT	1.8	0.9	Intravenously injectable drug use	
30140028	1020000
/  6.0086
/>800000	F0-F1	56
/Grade 1	1a
/
/1a
/1a	CT
/CT	27.8	0.8	Blood transfusion	
30140029	196000
/  5.2923
/<400000	F2	61
/Grade 1	1b
/
/1b
/1b	TT
/TT		5.1	Other	
30140030	4390000
/  6.6425
/>800000	F0-F1	19
/Grade 0	1a
/
/1a
/1a	CC
/CC		8.4	Heterosexual contact	
30140031	3340000
/  6.5237
/>800000	F2	71
/Grade 1	1b
/
/1b
/1b	CC
/CC		2	Other	
30140032	10600000
/  7.0253
/>800000	F0-F1	41
/Grade 0	1b
/
/1b
/1b	CT
/CT		0.6	Other	
30140033	324000
/  5.5105
/<400000	F2	41
/Grade 0	1a
/
/1a
/1a	CT
/CT	30.9	11.9	Intravenously injectable drug use	
30140034	387000
/  5.5877
/<400000	F0-F1	28
/Grade 0	1b
/
/1b
/1b	TT
/TT		4.4	Other	
30140035	11700000
/  7.0682
/>800000	F0-F1	43
/Grade 0	1b
/
/1b
/1b	CT
/CT		1.1	Other	
30140036	829000
/  5.9186
/>800000	F0-F1	46
/Grade 0	1a
/
/1a
/1a	CT
/CT	33.9	2.4	Intravenously injectable drug use	
30140038	1250000
/  6.0969
/>800000	F0-F1	32
/Grade 0	1a
/
/1a
/1a	CT
/CT		0.7	Intravenously injectable drug use	
30140039	1620000
/  6.2095
/>800000	F0-F1	45
/Grade 1	1b
/
/1b
/1b	CT
/CT	22.8	16.9	Other	
30140040	1770000
/  6.2480
/>800000	F0-F1	43
/Grade 0	1b
/
/1b
/1b	CT
/CT	23.9	4.3	Blood transfusion	
30140043	1480000
/  6.1703
/>800000	F2	76
/Grade 1	1a
/
/1a
/1a	CT
/CT	40.9	16.6	Other	
30140044	11100000
/  7.0453
/>800000	F0-F1	55
/Grade 1	1a
/
/1a
/1a	CT
/CT		21.7	Blood transfusion	
30140046	46300
/  4.6656
/<400000	F0-F1	70
/Grade 1	1b
/
/1b
/1b	CT
/CT	1.1	0.9	Intravenously injectable drug use	
30140047	166000
/  5.2201
/<400000	F0-F1	108
/Grade 1	1b
/
/1b
/1b	CT
/CT	2.9	2.7	Intravenously injectable drug use	
30140048	6810000
/  6.8331
/>800000	F0-F1	140
/Grade 2	1b
/
/1b
/1b	CT
/CT		0.6	Other	
30140050	17200000
/  7.2355
/>800000	F0-F1	90
/Grade 1	1a
/
/1a
/1a	CC
/CC		1.1	Other	
30140052	459000
/  5.6618
/≥400000 - ≤800000	F0-F1	70
/Grade 1	1a
/
/1a
/1a	CT
/CT	10.9	8.6	Intravenously injectable drug use	
30140053	6510000
/  6.8136
/>800000	F0-F1	115
/Grade 2	1a
/
/1a
/1a	CC
/CC		2.4	Heterosexual contact	
30140054	484000
/  5.6848
/≥400000 - ≤800000	F0-F1	184
/Grade 3	1a
/
/1a
/1a	CC
/CC	4.9	1.7	Intravenously injectable drug use	
30140055	110000
/  5.0414
/<400000	F0-F1	54
/Grade 1	1a
/
/1a
/1a	CC
/CC	13.6	9.1	Intravenously injectable drug use	
30140056	49300000
/  7.6928
/>800000	F0-F1	60
/Grade 1	1b
/
/1b
/1b	CC
/CC		6.4	Intravenously injectable drug use	
30140057	418000
/  5.6212
/≥400000 - ≤800000	F0-F1	149
/Grade 2	1b
/
/1b
/1b	TT
/TT	36.9	12.9	Blood transfusion	
30140058	1640000
/  6.2148
/>800000	F0-F1	59
/Grade 1	1b
/
/1b
/1b	TT
/TT		1.1	Other	
30140061	45200
/  4.6551
/<400000	F0-F1	59
/Grade 1	1b
/
/1b
/1b	CT
/CT	29.9	22.9	Hemophilia-associated injections	
30140062	2350000
/  6.3711
/>800000	F0-F1	71
/Grade 1	1a
/
/1a
/1a	CC
/CC		18.9	Other	
30140065	3060000
/  6.4857
/>800000	F0-F1	39
/Grade 0	1a
/
/1a
/1a	CT
/CT	28.9	2.9	Blood transfusion	
30140067	5930000
/  6.7731
/>800000	F0-F1	34
/Grade 0	1b
/
/1b
/1b	CT
/CT	36.9	21.9	Blood transfusion	
30140068	4910000
/  6.6911
/>800000	F0-F1	27
/Grade 0	1b
/
/1b
/1b	CT
/CT	41.9	15.6	Intravenously injectable drug use	
30140070	7260000
/  6.8609
/>800000	F0-F1	29
/Grade 0	1b
/
/1b
/1b	CT
/CT		0.8	Other	
30140072	11700
/  4.0682
/<400000	F0-F1	30
/Grade 0	1b
/
/1b
/1b	CT
/CT		4.9	Other	
30140074	105000
/  5.0212
/<400000	F0-F1	37
/Grade 0	1a
/
/1a
/1a	TT
/TT		15.9	Intravenously injectable drug use	
30140075	6520000
/  6.8142
/>800000	F0-F1	22
/Grade 0	1a
/
/1a
/1a	CC
/CC		19.9	Multiple	
30140076	1350000
/  6.1303
/>800000	F0-F1	33
/Grade 0	1a
/1a
/
/1a	CT
/CT	21.9	1.4	Blood transfusion	
30140077	3870000
/  6.5877
/>800000	F0-F1	22
/Grade 0	1b
/
/1b
/1b	CT
/CT	15.9	15.9	Other	
30140078	1140000
/  6.0569
/>800000	F2	36
/Grade 0	1b
/
/1b
/1b	CC
/CC	18.9	18.9	Other	
30140080	2310000
/  6.3636
/>800000	F0-F1	16
/Grade 0	1a
/
/1a
/1a	CT
/CT	1.6	1.6	Other	
30140081	1500000
/  6.1761
/>800000	F2	79
/Grade 1	1b
/
/1b
/1b	CT
/CT	24.9	16	Blood transfusion	
30140084	432000
/  5.6355
/≥400000 - ≤800000	F0-F1	55
/Grade 1	1b
/
/1b
/1b	CT
/CT		5.9	Other	
30140085	1590000
/  6.2014
/>800000	F0-F1	79
/Grade 1	1b
/
/1b
/1b	TT
/TT	42.9	18.9	Other	
30140086	212000
/  5.3263
/<400000	F2	56
/Grade 1	1b
/
/1b
/1b	CT
/CT		1.9	Other	
30140087	5640000
/  6.7513
/>800000	F2	45
/Grade 0	1b
/
/1b
/1b	CC
/CC		1.6	Other	
30140088	15400000
/  7.1875
/>800000	F0-F1	92
/Grade 1	1b
/
/1b
/1b	CC
/CC		20.9	Multiple	
30140091	15500000
/  7.1903
/>800000	F0-F1	44
/Grade 0	1b
/
/1b
/1b	CC
/CC		0.9	Other	
30140092	382000
/  5.5821
/<400000	F2	162
/Grade 2	1b
/
/1b
/1b	CT
/CT		1.8	Other	
30140093	9550000
/  6.9800
/>800000	F2	134
/Grade 2	1b
/
/1b
/1b	CT
/CT		8.9	Other	
30140094	3900000
/  6.5911
/>800000	F0-F1	56
/Grade 1	1b
/
/1b
/1b	CT
/CT		11.9	Mother to child transmission	
30140098	1510000
/  6.1790
/>800000	F0-F1	25
/Grade 0	1b
/
/1b
/1b	CT
/CT		2.4	Other	
30140099	909000
/  5.9586
/>800000	F2	37
/Grade 0	1b
/
/1b
/1b	CT
/CT	2.9	2.9	Heterosexual contact	
30140100	2410000
/  6.3820
/>800000	F0-F1	25
/Grade 0	1b
/
/1b
/1b	CT
/CT		0.5	Other	
30140101	381000
/  5.5809
/<400000	F0-F1	42
/Grade 0	1b
/
/1b
/1b	CT
/CT	27.9	22.9	Blood transfusion	
30140102	4770000
/  6.6785
/>800000	F0-F1	88
/Grade 1	1a
/
/1a
/1a	CT
/CT	21.5	9.9	Intravenously injectable drug use	
30140103	1380000
/  6.1399
/>800000	F0-F1	27
/Grade 0	1b
/
/1b
/1b	CT
/CT		28.9	Other	
30140105	12400000
/  7.0934
/>800000	F2	97
/Grade 2	1a
/
/1a
/1a	CT
/CT		20	Intravenously injectable drug use	
30140106	411000
/  5.6138
/≥400000 - ≤800000	F0-F1	55
/Grade 1	1b
/
/1b
/1b	TT
/TT	33.9	18.9	Blood transfusion	
30140107	5480000
/  6.7388
/>800000	F0-F1	66
/Grade 1	1a
/
/1a
/1a	TT
/TT		8.9	Intravenously injectable drug use	
30140108	720000
/  5.8573
/≥400000 - ≤800000	F0-F1	34
/Grade 0	1b
/
/1b
/1b	TT
/TT		1.3	Other	
30140109	1560000
/  6.1931
/>800000	F2	47
/Grade 0	1a
/
/1a
/1a	CT
/CT		16.9	Intravenously injectable drug use	
30140110	15500000
/  7.1903
/>800000	F2	102
/Grade 1	1b
/
/1b
/1b	CT
/CT	14.9	0.7	Other	
30140112	7340000
/  6.8657
/>800000		120
/Grade 2	1a
/
/1a
/1a	CC
/CC		10	Other	
30140113	2840000
/  6.4533
/>800000	F2	37
/Grade 0	1b
/
/1b
/1b	CT
/CT	2.7	2.4	Blood transfusion	
30140114	9870000
/  6.9943
/>800000	F0-F1	81
/Grade 1	1b
/
/1b
/1b	CC
/CC	33.9	4.1	Blood transfusion	
30140116	5150000
/  6.7118
/>800000	F0-F1	65
/Grade 1	1b
/
/1b
/1b	CT
/CT		0.6	Other	
30140117	1030000
/  6.0128
/>800000	F0-F1	45
/Grade 0	1b
/
/1b
/1b	CT
/CT		4.1	Other	
30140118	125000
/  5.0969
/<400000	F2	61
/Grade 1	1b
/
/1b
/1b	CT
/CT		0.8	Other	
30140120	89500
/  4.9518
/<400000	F0-F1	64
/Grade 1	1b
/
/1b
/1b	CC
/CC		6.9	Other	
30140124	1760000
/  6.2455
/>800000	F0-F1	36
/Grade 0	1b
/
/1b
/1b	CT
/CT		5	Other	
30140126	27100000
/  7.4330
/>800000	F0-F1	42
/Grade 0	1a
/
/1a
/1a	CT
/CT		19.9	Intravenously injectable drug use	
30140129	3680000
/  6.5658
/>800000	F2	80
/Grade 1	1a
/
/1a
/1a	CT
/CT		2	Other	
30140130	6960000
/  6.8426
/>800000	F2	80
/Grade 1	1b
/
/1b
/1b	CC
/CC	29.9	0.6	Blood transfusion	
30140133	2820000
/  6.4502
/>800000	F0-F1	25
/Grade 0	1b
/
/1b
/1b	CT
/CT		25.9	Other	
30140136	2810000
/  6.4487
/>800000	F2	104
/Grade 1	1a
/
/1a
/1a	CT
/CT	29.9	2.9	Blood transfusion	
30140139	8910000
/  6.9499
/>800000	F2	168
/Grade 2	1a
/
/1a
/1a	CC
/CC		11.9	Other	
30140140	552000
/  5.7419
/≥400000 - ≤800000	F0-F1	48
/Grade 1	1b
/
/1b
/1b	CT
/CT	1.9	1.9	Other	
30140143	1010000
/  6.0043
/>800000	F0-F1	35
/Grade 0	1b
/
/1b
/1b	CT
/CT		14.9	Other	
30140144	6220000
/  6.7938
/>800000	F0-F1	54
/Grade 1	1a
/
/1a
/1a	CT
/CT		24.9	Intravenously injectable drug use	
30140146	604000
/  5.7810
/≥400000 - ≤800000	F0-F1	56
/Grade 1	1b
/1b
/
/1b	CT
/CT		1.5	Other	
30140147	896000
/  5.9523
/>800000	F0-F1	31
/Grade 0	1b
/1b
/
/1b	CT
/CT	36.9	1.3	Intravenously injectable drug use	
30140148	6010000
/  6.7789
/>800000	F2	152
/Grade 2	1b
/
/1b
/1b	CT
/CT	40	6.2	Blood transfusion	
30140151	8230000
/  6.9154
/>800000	F0-F1	24
/Grade 0	1a
/
/1a
/1a	CC
/CC	29.9	27.9	Intravenously injectable drug use	
30140152	4200000
/  6.6232
/>800000	F0-F1	40
/Grade 0	1a
/
/1a
/1a	CT
/CT	15.9	12.9	Blood transfusion	
30140153	632000
/  5.8007
/≥400000 - ≤800000	F0-F1	32
/Grade 0	1b
/
/1b
/1b	CT
/CT		2.1	Intravenously injectable drug use	
30140154	16000000
/  7.2041
/>800000	F0-F1	79
/Grade 1	1b
/
/1b
/1b	CT
/CT		12.1	Other	
30140157	147000
/  5.1673
/<400000	F0-F1	26
/Grade 0	1b
/
/1b
/1b	CC
/CC	3	2.1	Other	
30140158	311000
/  5.4928
/<400000	F0-F1	56
/Grade 1	1b
/
/1b
/1b	CT
/CT		1.7	Other	
30140159	12200000
/  7.0864
/>800000	F0-F1	26
/Grade 0	1b
/
/1b
/1b	CC
/CC		20	Other	
30140161	14100000
/  7.1492
/>800000	F0-F1	62
/Grade 1	1a
/
/1a
/1a	CC
/CC		3	Other	
30140164	2700000
/  6.4314
/>800000	F0-F1	60
/Grade 1	1a
/
/1a
/1a	CC
/CC	11.9	11.9	Intravenously injectable drug use	
30140166	192000
/  5.2833
/<400000	F0-F1	55
/Grade 1	1b
/
/1b
/1b	CT
/CT	1.9	1.9	Other	
30140167	2420000
/  6.3838
/>800000	F2	28
/Grade 0	1b
/
/1b
/1b	CC
/CC	52	13.3	Blood transfusion	
30140168	227000
/  5.3560
/<400000	F2	228
/Grade 3	1b
/
/1b
/1b	CC
/CC		15	Mother to child transmission	
30140169	15900000
/  7.2014
/>800000	F0-F1	24
/Grade 0	1a
/
/1a
/1a	CT
/CT	11	10.2	Heterosexual contact	
30140170	3480000
/  6.5416
/>800000	F0-F1	49
/Grade 0	1a
/
/1a
/1a	CC
/CC		26	Other	
30140173	4130000
/  6.6160
/>800000	F0-F1	146
/Grade 2	1b
/
/1b
/1b	CT
/CT	7	2	Other	
30140174	510000
/  5.7076
/≥400000 - ≤800000	F0-F1	31
/Grade 0	1a
/
/1a
/1a	TT
/TT		5	Intravenously injectable drug use	
30140178	818000
/  5.9128
/>800000	F0-F1	122
/Grade 2	1b
/
/1b
/1b	CT
/CT	4	1.7	Blood transfusion	
30140179	10700000
/  7.0294
/>800000	F0-F1	35
/Grade 0	1a
/
/1a
/1a	CC
/CC		16.8	Other	
30140180	393000
/  5.5944
/<400000	F0-F1	54
/Grade 1	1a
/
/1a
/1a	CT
/CT	1.1	0.8	Other	
30140181	9410000
/  6.9736
/>800000	F2	81
/Grade 1	1b
/
/1b
/1b	CT
/CT	1.2	1.1	Other	
30140184	9270000
/  6.9671
/>800000	F0-F1	30
/Grade 0	1b
/
/1b
/1b	CC
/CC		13	Other	
30140185	5060000
/  6.7042
/>800000	F2	51
/Grade 1	1b
/
/1b
/1b	CT
/CT		8	Other	
30140186	20100000
/  7.3032
/>800000	F0-F1	41
/Grade 0	1a
/
/1a
/1a	CC
/CC		17	Intravenously injectable drug use	
HCV Genotype: Genotype 1 - Treatment Duration: 24 Weeks Treatment	
30140014	379000
/  5.5786
/<400000	F0-F1	50
/Grade 1	1a
/
/1a
/1a	CT
/CT		1.1	Heterosexual contact	
30140018	941000
/  5.9736
/>800000	F0-F1	29
/Grade 0	1b
/
/1b
/1b	TT
/TT	11.9	11.4	Other	
30140022	10200000
/  7.0086
/>800000	F2	60
/Grade 1	1a
/
/1a
/1a	CT
/CT	10.8	1.6	Intravenously injectable drug use	
30140041	9530000
/  6.9791
/>800000	F0-F1	61
/Grade 1	1a
/
/1a
/1a	CT
/CT		16.3	Other	
30140042	2260000
/  6.3541
/>800000	F0-F1	33
/Grade 0	1b
/1b
/
/1b	TT
/TT		1.5	Other	
30140049	505000
/  5.7033
/≥400000 - ≤800000	F0-F1	33
/Grade 0	1b
/
/1b
/1b	TT
/TT		0.6	Other	
30140051	1460000
/  6.1644
/>800000	F0-F1	159
/Grade 2	1a
/
/1a
/1a	CT
/CT	2.9	1	Other	
30140059	5770000
/  6.7612
/>800000	F2	37
/Grade 0	1a
/
/1a
/1a	CT
/CT		0.7	Other	
30140064	8570000
/  6.9330
/>800000	F0-F1	121
/Grade 2	1a
/
/1a
/1a	CC
/CC	33.4	1.9	Mother to child transmission	
30140066	7500000
/  6.8751
/>800000	F0-F1	25
/Grade 0	1a
/
/1a
/1a	CC
/CC	29.9	18.9	Intravenously injectable drug use	
30140069	9380000
/  6.9722
/>800000	F2	67
/Grade 1	1a
/
/1a
/1a	CT
/CT		1.1	Intravenously injectable drug use	
30140071	28000000
/  7.4472
/>800000	F0-F1	88
/Grade 1	1b
/
/1b
/1b	CC
/CC		1.2	Other	
30140073	3330000
/  6.5224
/>800000	F2	32
/Grade 0	1b
/
/1b
/1b	CT
/CT	8.9	8.9	Other	
30140079	24800000
/  7.3945
/>800000	F0-F1	33
/Grade 0	1a
/
/1a
/1a	CC
/CC	26.9	23.9	Blood transfusion	
30140082	5080000
/  6.7059
/>800000	F2	63
/Grade 1	1b
/
/1b
/1b	TT
/TT	1.9	1.7	Intravenously injectable drug use	
30140083	9200000
/  6.9638
/>800000	F2	61
/Grade 1	1b
/
/1b
/1b	CT
/CT	1.9	1.9	Other	
30140089	1980000
/  6.2967
/>800000	F0-F1	73
/Grade 1	1b
/
/1b
/1b	TT
/TT	27.9	1.9	Blood transfusion	
30140090	19000000
/  7.2788
/>800000	F2	43
/Grade 1	1a
/
/1a
/1a	CT
/CT		26.9	Blood transfusion	
30140096	3650000
/  6.5623
/>800000	F0-F1	51
/Grade 0	1b
/
/1b
/1b	CT
/CT		19.9	Other	
30140111	11600000
/  7.0645
/>800000	F2	67
/Grade 1	1b
/
/1b
/1b	CT
/CT		16.9	Blood transfusion	
30140115	4620000
/  6.6646
/>800000	F0-F1	21
/Grade 0	1a
/
/1a
/1a	CC
/CC		4.2	Blood transfusion	
30140119	1730000
/  6.2380
/>800000	F0-F1	38
/Grade 0	1a
/
/1a
/1a	CC
/CC	34.9	0.8	Blood transfusion	
30140122	3000000
/  6.4771
/>800000	F2	120
/Grade 2	1b
/
/1b
/1b	CT
/CT		0.7	Other	
30140123	8400000
/  6.9243
/>800000	F0-F1	101
/Grade 1	1a
/
/1a
/1a	CT
/CT		4.9	Other	
30140125	3360000
/  6.5263
/>800000	F0-F1	68
/Grade 1	1b
/
/1b
/1b	CT
/CT		0.7	Other	
30140132	3160000
/  6.4997
/>800000	F0-F1	54
/Grade 1	1b
/
/1b
/1b	TT
/TT		21	Other	
30140134	3850000
/  6.5855
/>800000	F2	66
/Grade 1	1b
/
/1b
/1b	CT
/CT		4.1	Other	
30140137	2150000
/  6.3324
/>800000	F0-F1	41
/Grade 0	1a
/
/1a
/1a	TT
/TT		13.9	Other	
30140142	156000
/  5.1931
/<400000	F0-F1	38
/Grade 0	1b
/
/1b
/1b	TT
/TT		0.7	Other	
30140149	974000
/  5.9886
/>800000	F2	39
/Grade 0	1a
/
/1a
/1a	CT
/CT		17.9	Other	
30140150	19400000
/  7.2878
/>800000	F2	116
/Grade 2	1a
/
/1a
/1a	CT
/CT		20.9	Intravenously injectable drug use	
30140155	1520000
/  6.1818
/>800000	F0-F1	48
/Grade 0	1a
/
/1a
/1a	TT
/TT	20	20	Other	
30140163	7340000
/  6.8657
/>800000	F0-F1	53
/Grade 1	1b
/
/1b
/1b	CT
/CT	2.3	2.3	Other	
30140165	18400000
/  7.2648
/>800000	F0-F1	76
/Grade 1	1b
/
/1b
/1b	CT
/CT	19.7	19.7	Blood transfusion	
30140171	7530000
/  6.8768
/>800000	F0-F1	23
/Grade 0	1b
/
/1b
/1b	TT
/TT	47	9.3	Blood transfusion	
30140175	4330000
/  6.6365
/>800000	F2	93
/Grade 1	1b
/
/1b
/1b	TT
/TT		0.5	Other	
30140176	3630000
/  6.5599
/>800000	F0-F1	43
/Grade 0	1b
/
/1b
/1b	CC
/CC		20	Blood transfusion	
30140177	13700000
/  7.1367
/>800000	F2	74
/Grade 1	1a
/
/1a
/1a	CC
/CC		0.9	Intravenously injectable drug use	
30140182	1360000
/  6.1335
/>800000	F0-F1	92
/Grade 2	1b
/
/1b
/1b	TT
/TT	2.2	1.5	Other	
HCV Genotype: Genotype 1 - Treatment Duration: 48 Weeks Treatment	
30140095	5660000
/  6.7528
/>800000	F2	48
/Grade 0	1b
/
/1b
/1b	CT
/CT	40.9	20.9	Other	
