# Supplementary material for: An Open-Label Trial of 12-Week Simeprevir plus Peginterferon/Ribavirin (PR) in Treatment-Naïve Patients with Hepatitis C Virus (HCV) Genotype 1 (GT1)
Source: PLoS One. 2016 Jul 18;11(7):e0158526. doi: 10.1371/journal.pone.0158526 (PMC4948848; doi:10.1371/journal.pone.0158526)
Supplement: S1 Fig — (DOCX) [file pone.0158526.s010.docx]

**S1 Figure**: Forest plot showing the results of the final multivariate logistic regression analyses of factors associated with **[A]** SVR12 (*n=*91) and **[B]** viral relapse (*n=*90) in patients with *IL28B* CT or TT genotype receiving 12 weeks of treatment

**A**

**
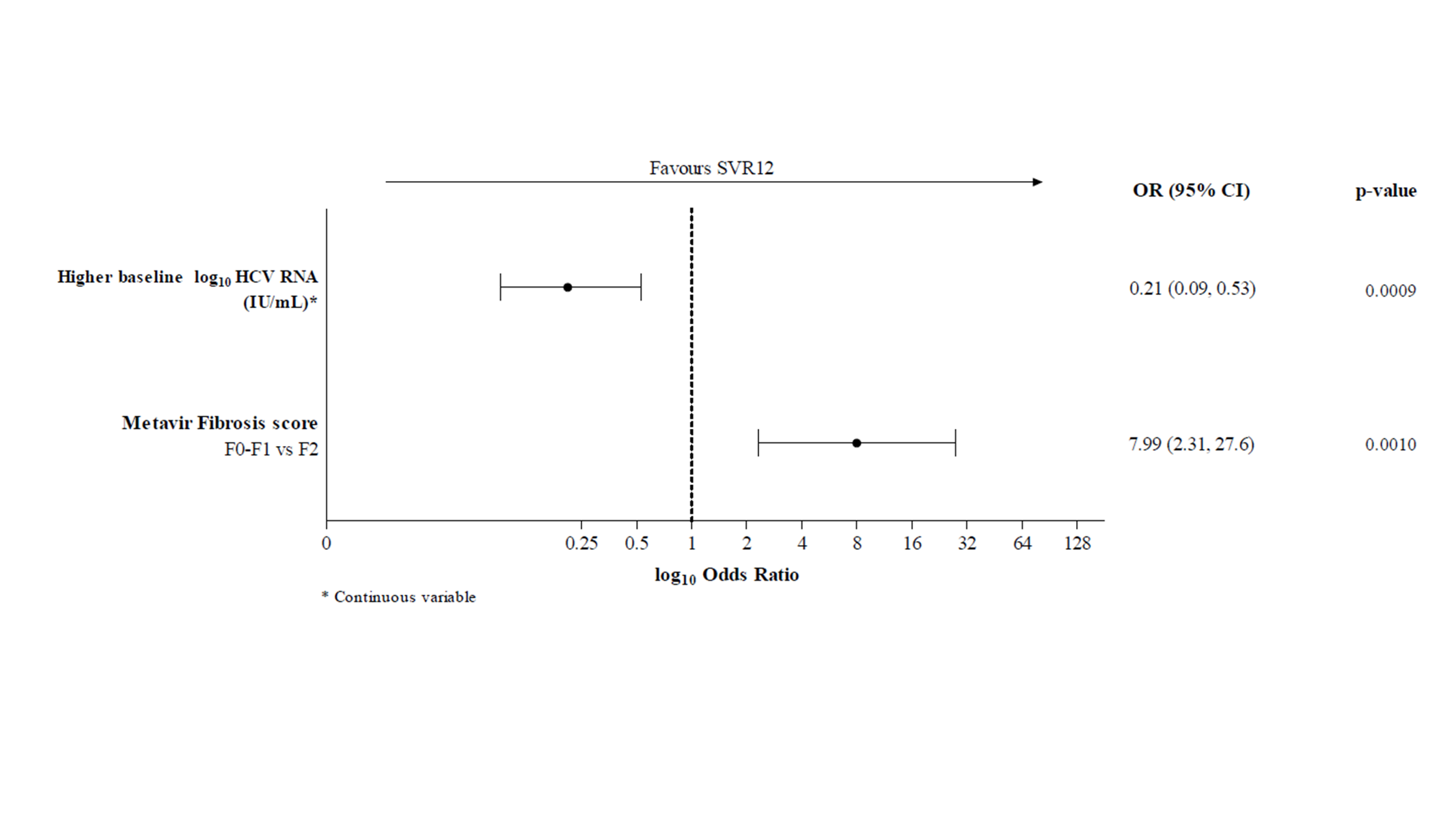
**

**B**

**
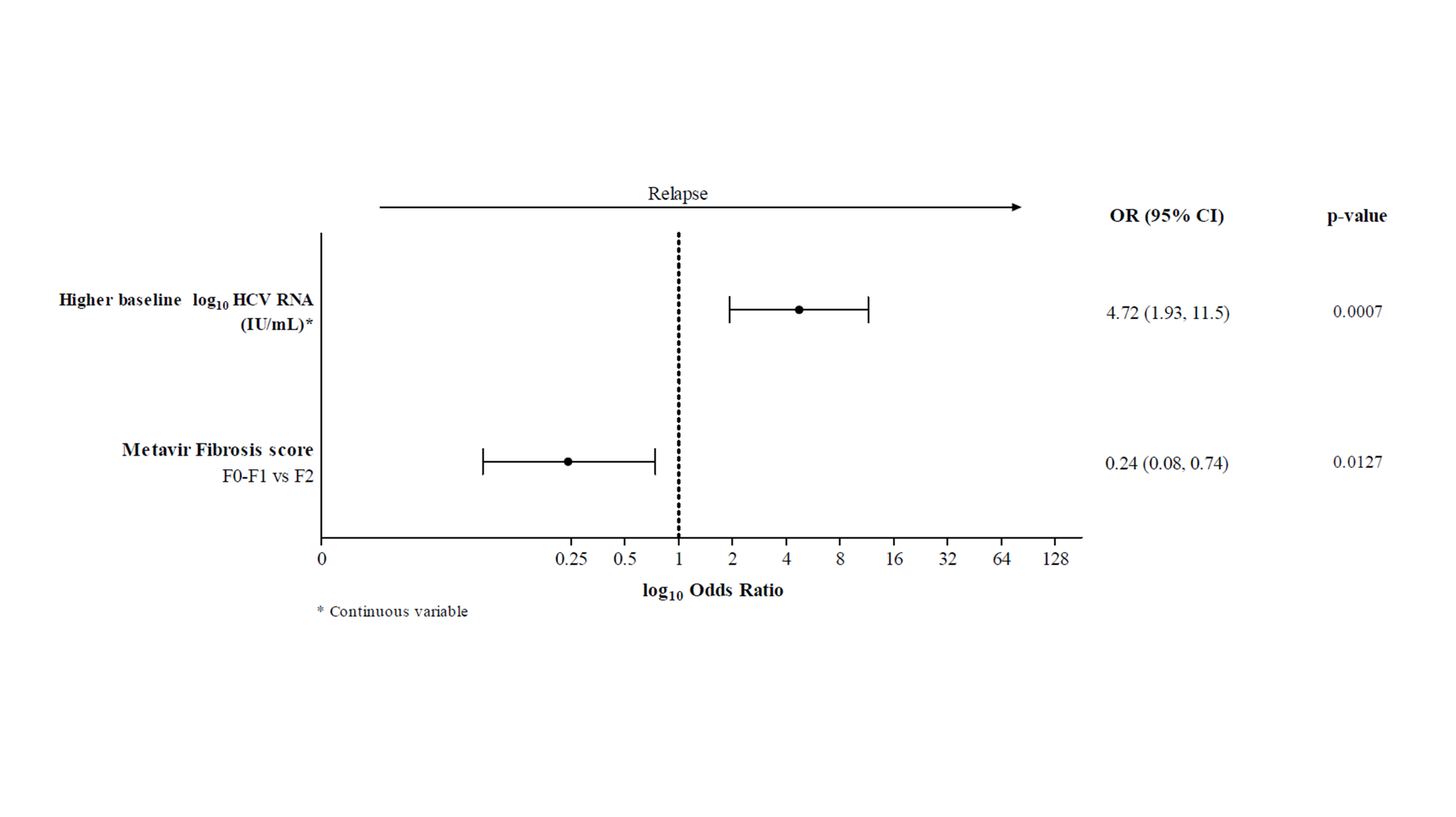
**
